# Supplementary material for: Structural Competency: A Faculty Development Workshop Series for Anti-racism in Medical Education
Source: MedEdPORTAL. 2025 Feb 7;21:11492. doi: 10.15766/mep_2374-8265.11492 (PMC11802914; doi:10.15766/mep_2374-8265.11492)
Supplement: Supplementary file 1 — 1 - Introduction to SC.pptx1 - Facilitator Guide.docx1 - SC Rubric Handout.docx1 - Sample SC Learning Goals.docx2 - Resident Reports & Case-Based Presentations.pptx2 - Facilitator Guide.docx2 - Structural Differential Handout.docx2 - Small-Group Handout.docx3 - Demystifying SC.pptx3 - Facilitator Guide.docx3 - SC One-Minute Preceptor Handout.docx3 - SC SNAPPS Handout.docx3 - Role-Play Scenarios.docx4 - SC Hospital-Based Teaching.pptx4 - Facilitator Guide.docx4 - Daily Inpatient Checklist.docx4 - SC Discharge Checklist.docx4 - Small-Group Scenarios.docxPre- and Postsurveys.docx [file mep_2374-8265.11492-s001.zip › E. 2 - Resident Reports & Case-Based Presentations.pptx]

## Slide 1
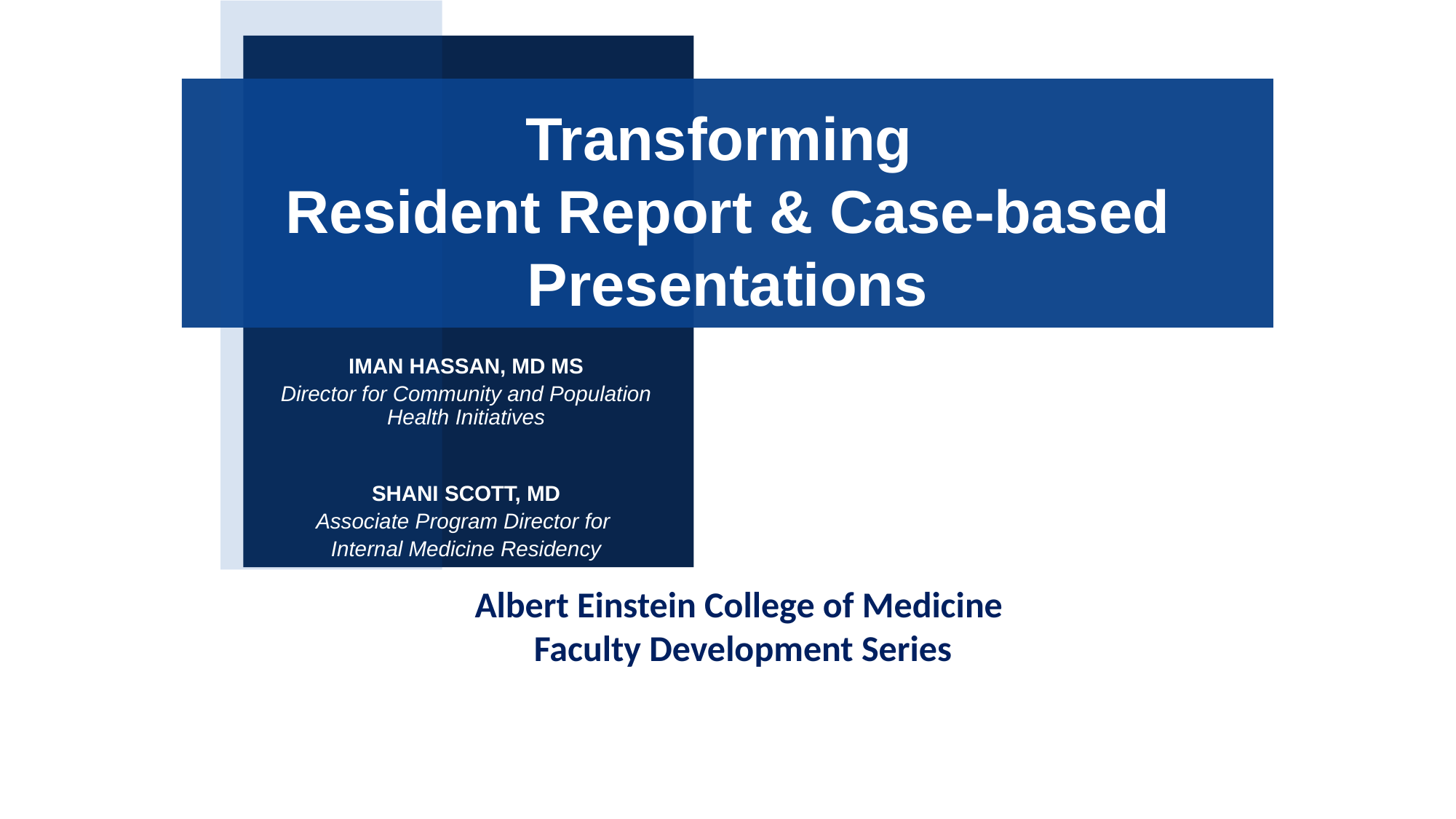

Transforming
Resident Report & Case-based Presentations
IMAN HASSAN, MD MS
Director for Community and Population Health Initiatives
SHANI SCOTT, MD
Associate Program Director for
Internal Medicine Residency
Albert Einstein College of Medicine
Faculty Development Series

## Slide 2
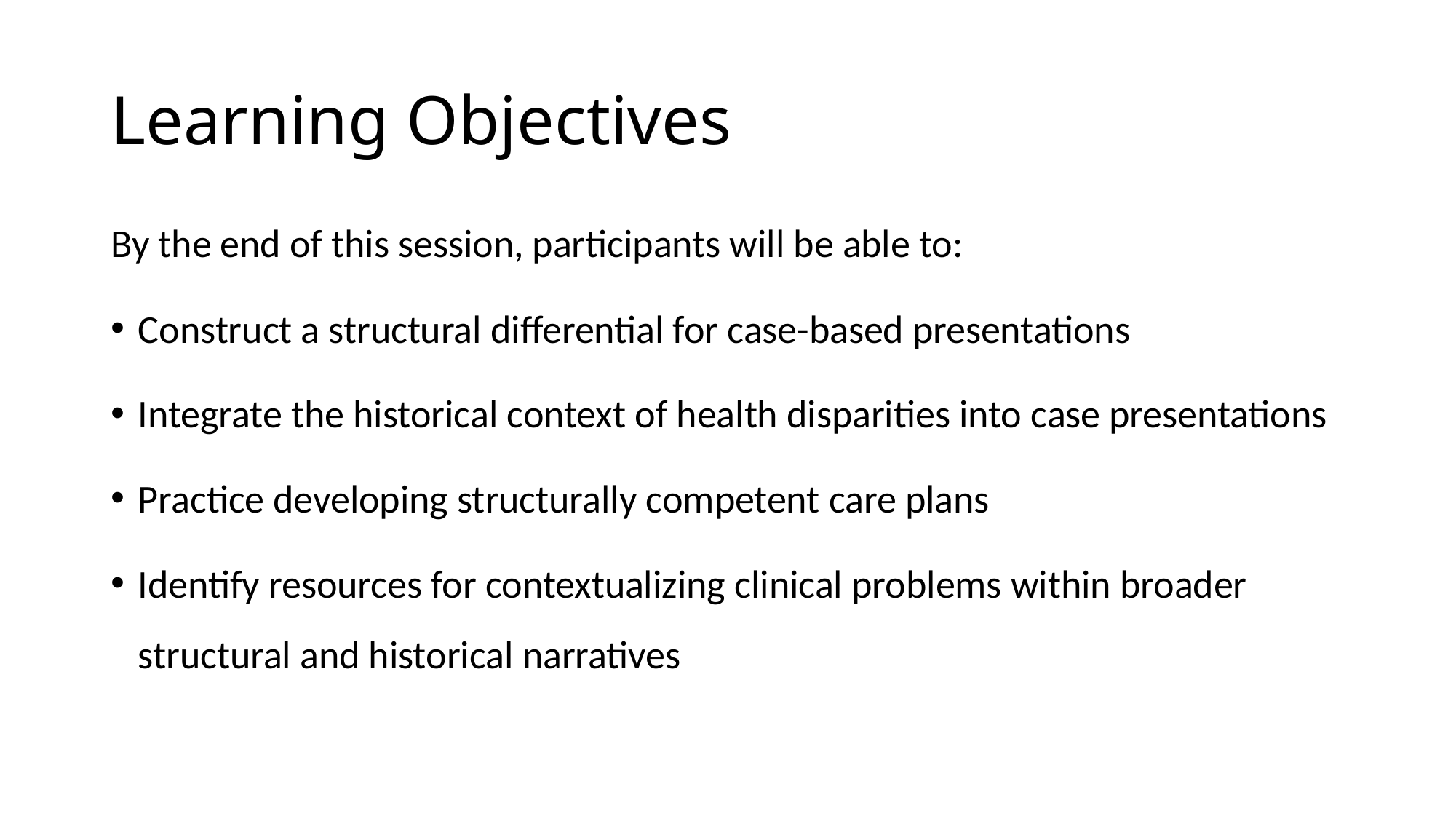

# Learning Objectives
By the end of this session, participants will be able to:
Construct a structural differential for case-based presentations
Integrate the historical context of health disparities into case presentations
Practice developing structurally competent care plans
Identify resources for contextualizing clinical problems within broader structural and historical narratives

## Slide 3
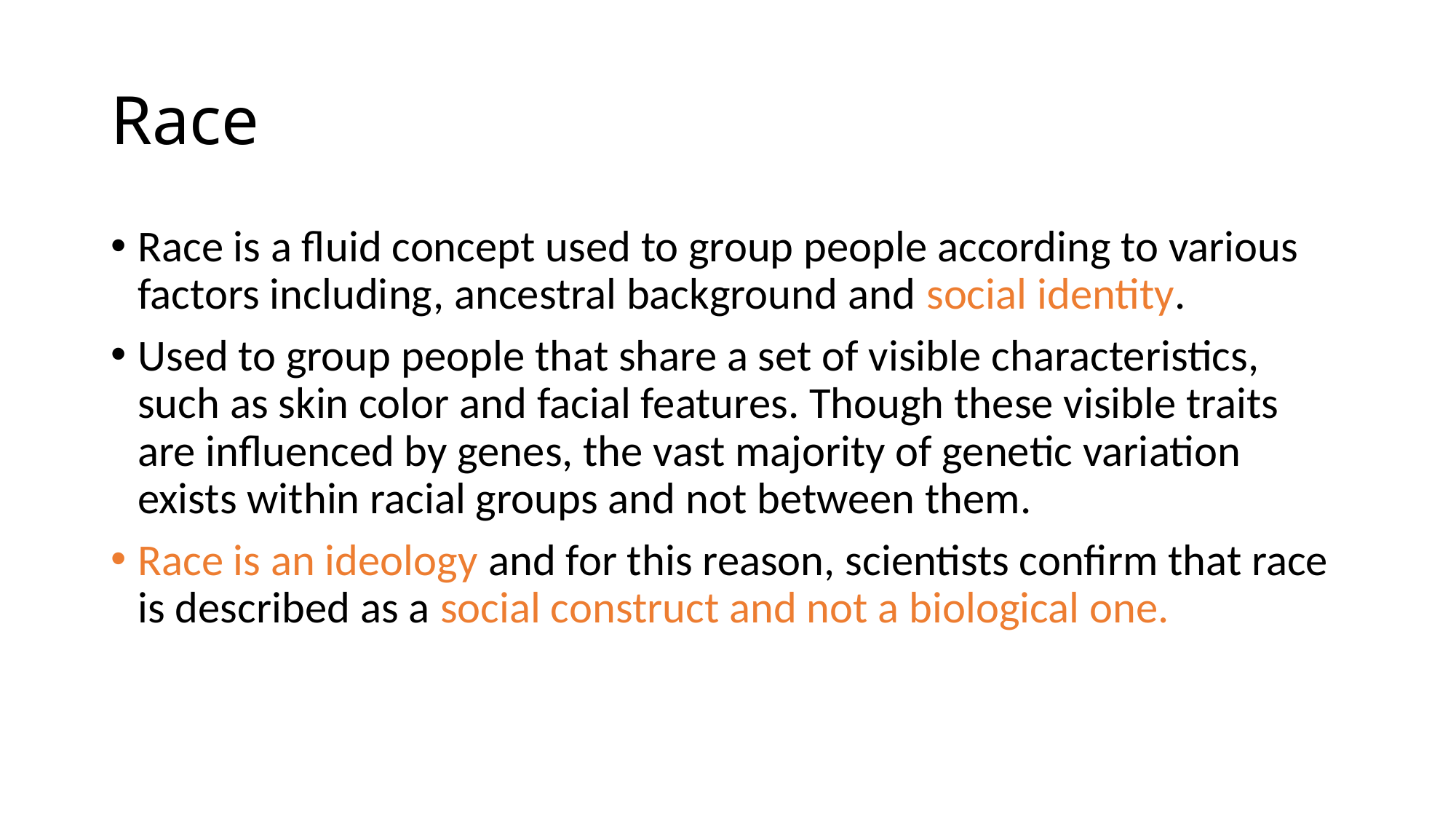

# Race
Race is a fluid concept used to group people according to various factors including, ancestral background and social identity.
Used to group people that share a set of visible characteristics, such as skin color and facial features. Though these visible traits are influenced by genes, the vast majority of genetic variation exists within racial groups and not between them.
Race is an ideology and for this reason, scientists confirm that race is described as a social construct and not a biological one.

## Slide 4
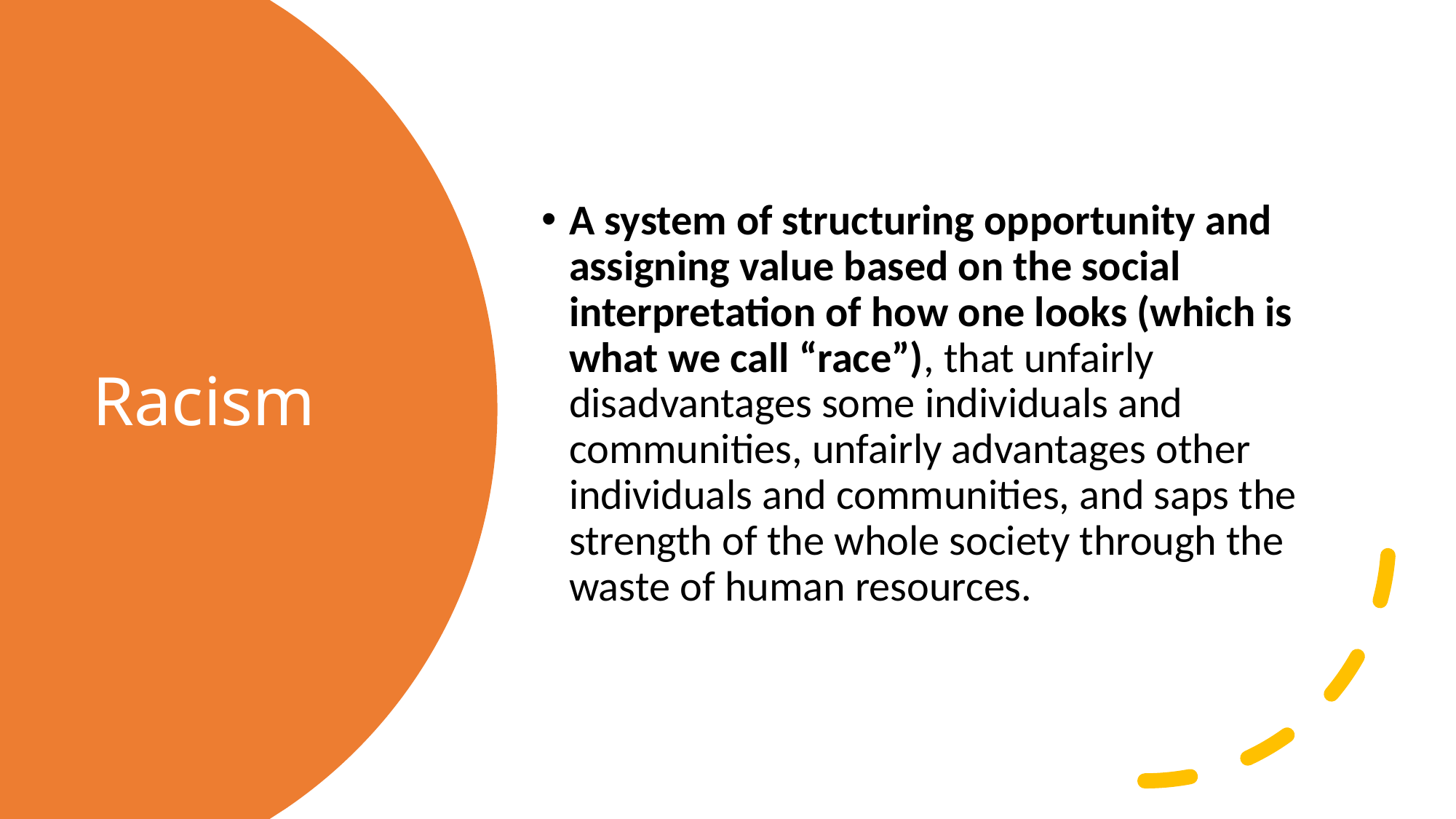

A system of structuring opportunity and assigning value based on the social interpretation of how one looks (which is what we call “race”), that unfairly disadvantages some individuals and communities, unfairly advantages other individuals and communities, and saps the strength of the whole society through the waste of human resources.
# Racism

## Slide 5
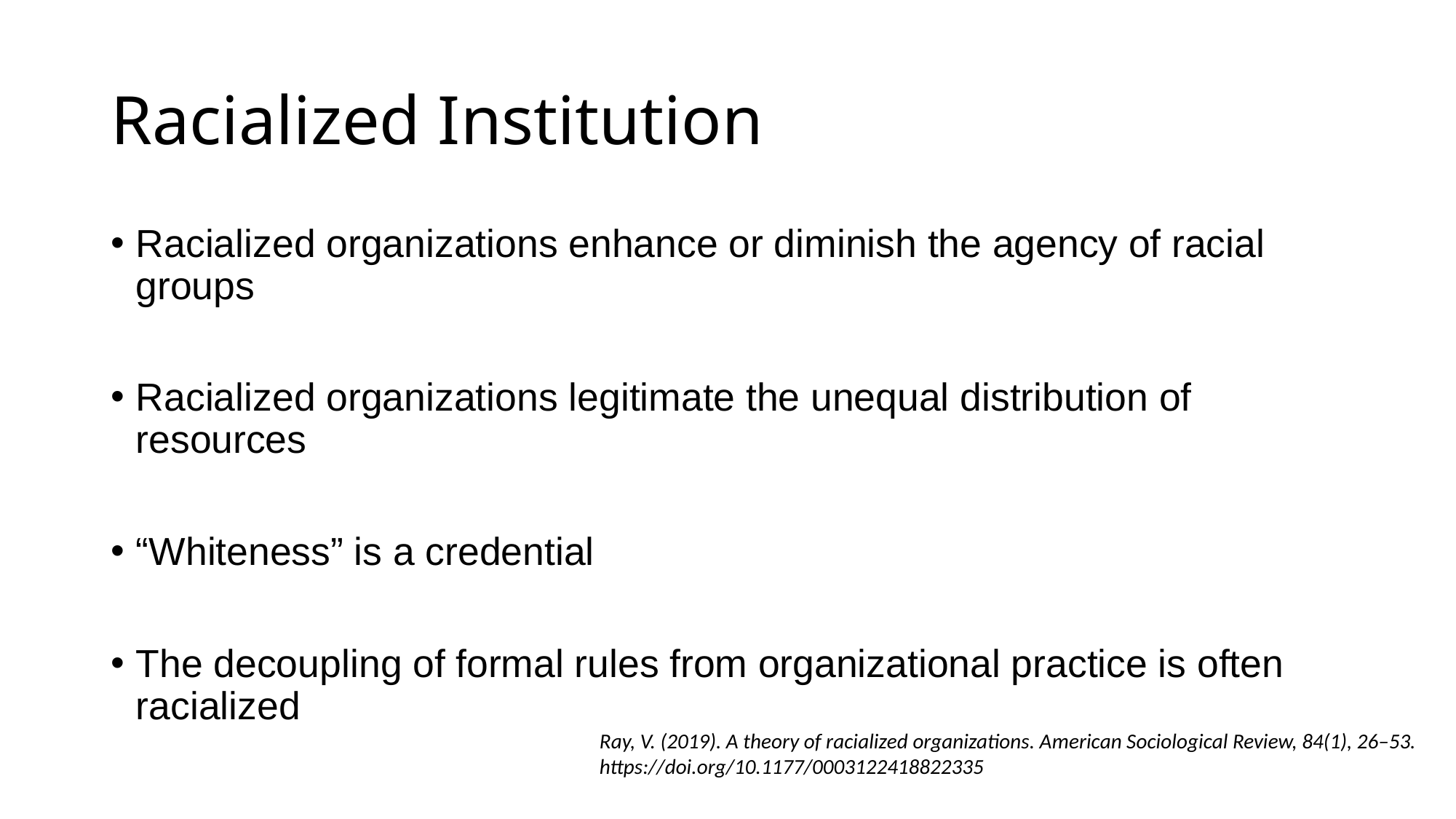

# Racialized Institution
Racialized organizations enhance or diminish the agency of racial groups
Racialized organizations legitimate the unequal distribution of resources
“Whiteness” is a credential
The decoupling of formal rules from organizational practice is often racialized
Ray, V. (2019). A theory of racialized organizations. American Sociological Review, 84(1), 26–53. https://doi.org/10.1177/0003122418822335

## Slide 6
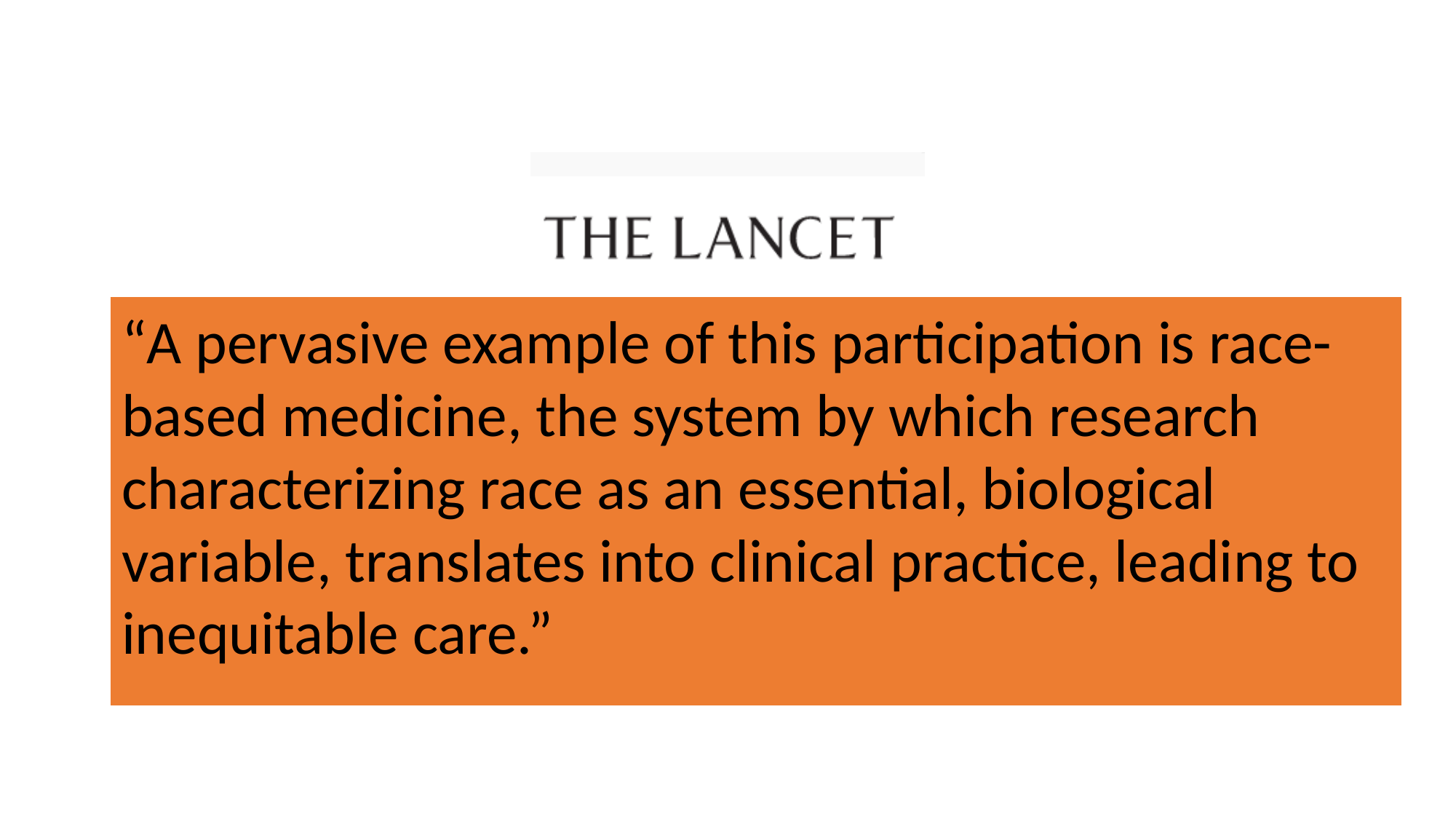

“A pervasive example of this participation is race-based medicine, the system by which research characterizing race as an essential, biological variable, translates into clinical practice, leading to inequitable care.”

## Slide 7
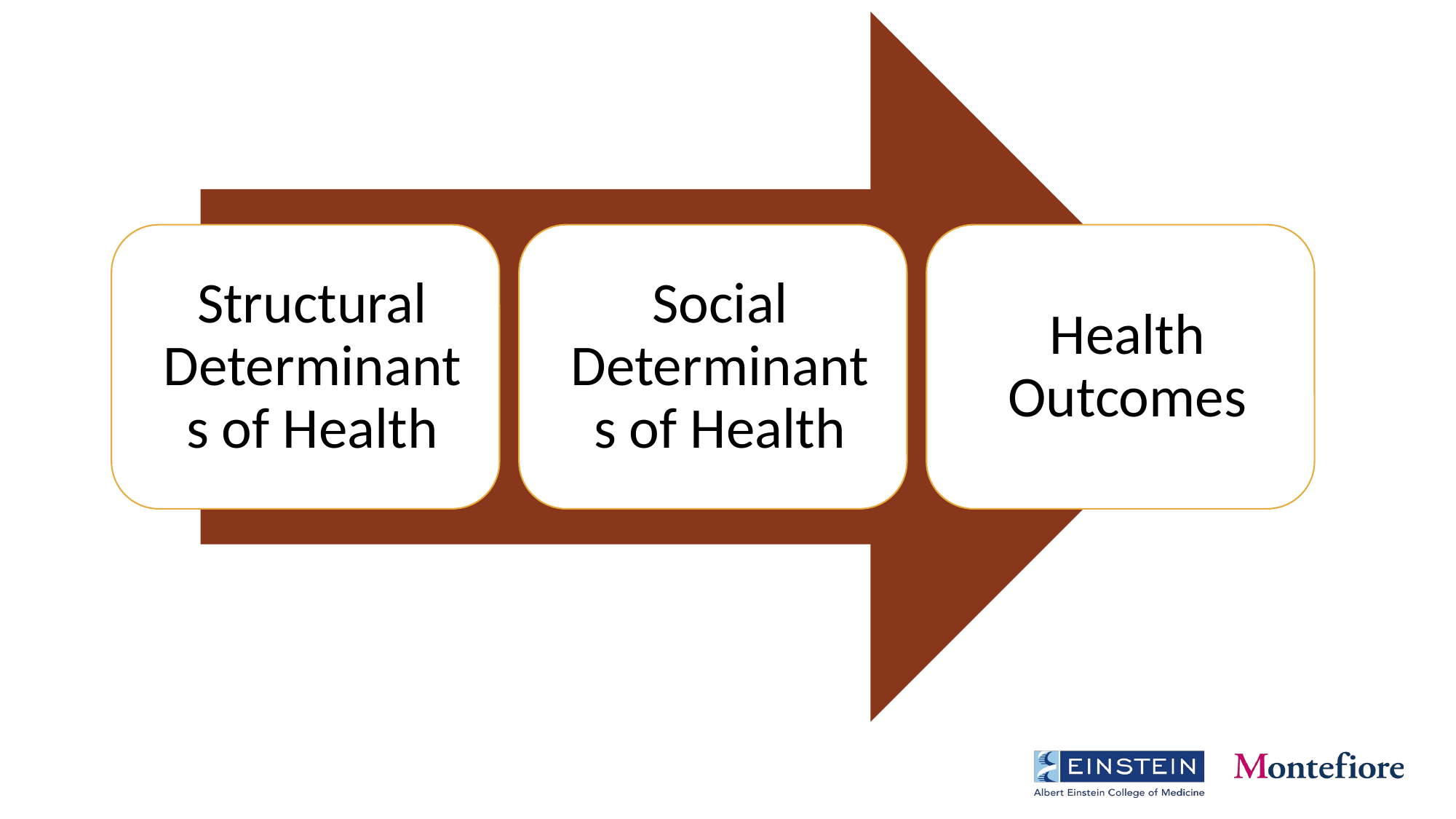

## Slide 8
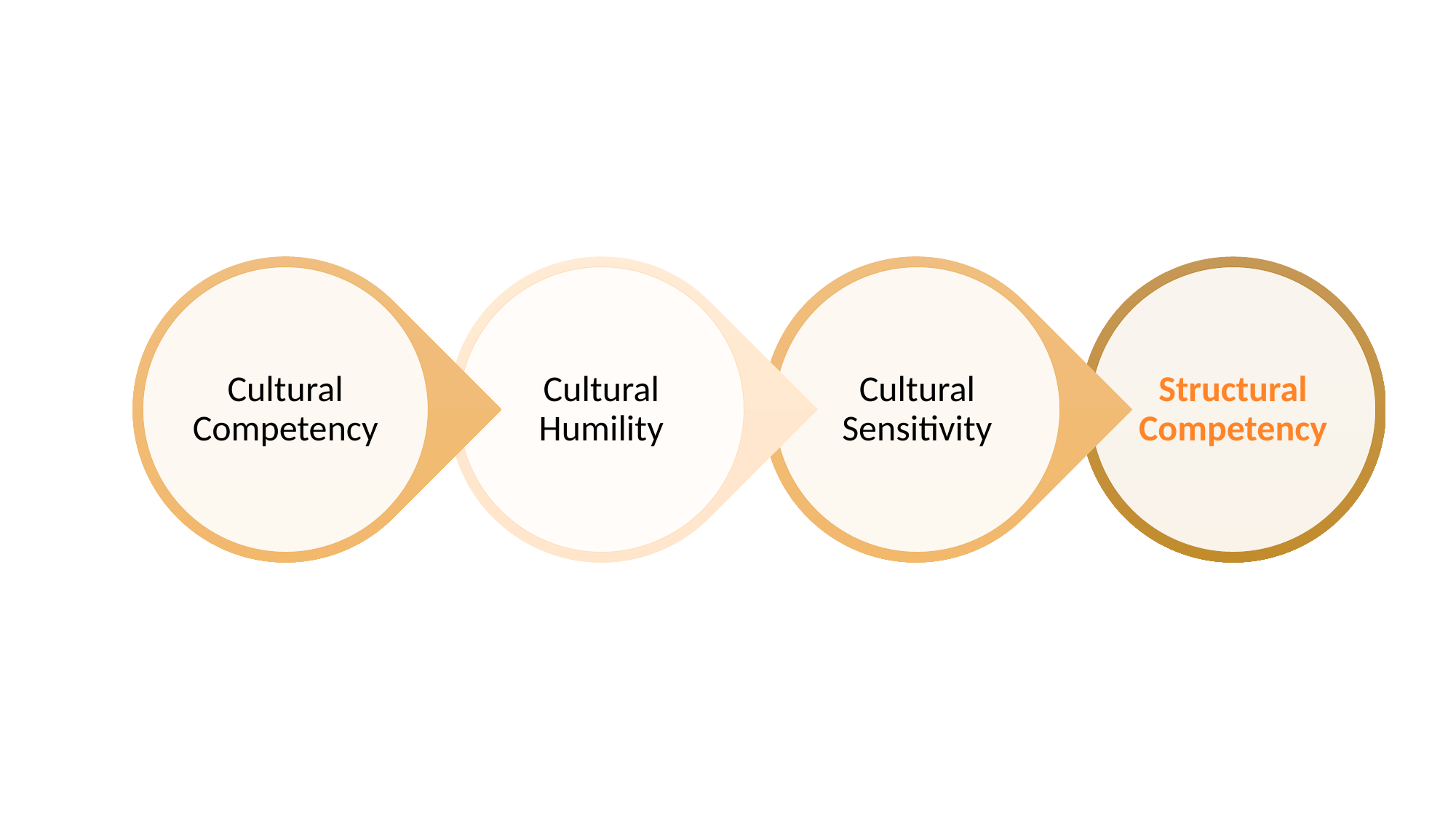

## Slide 9
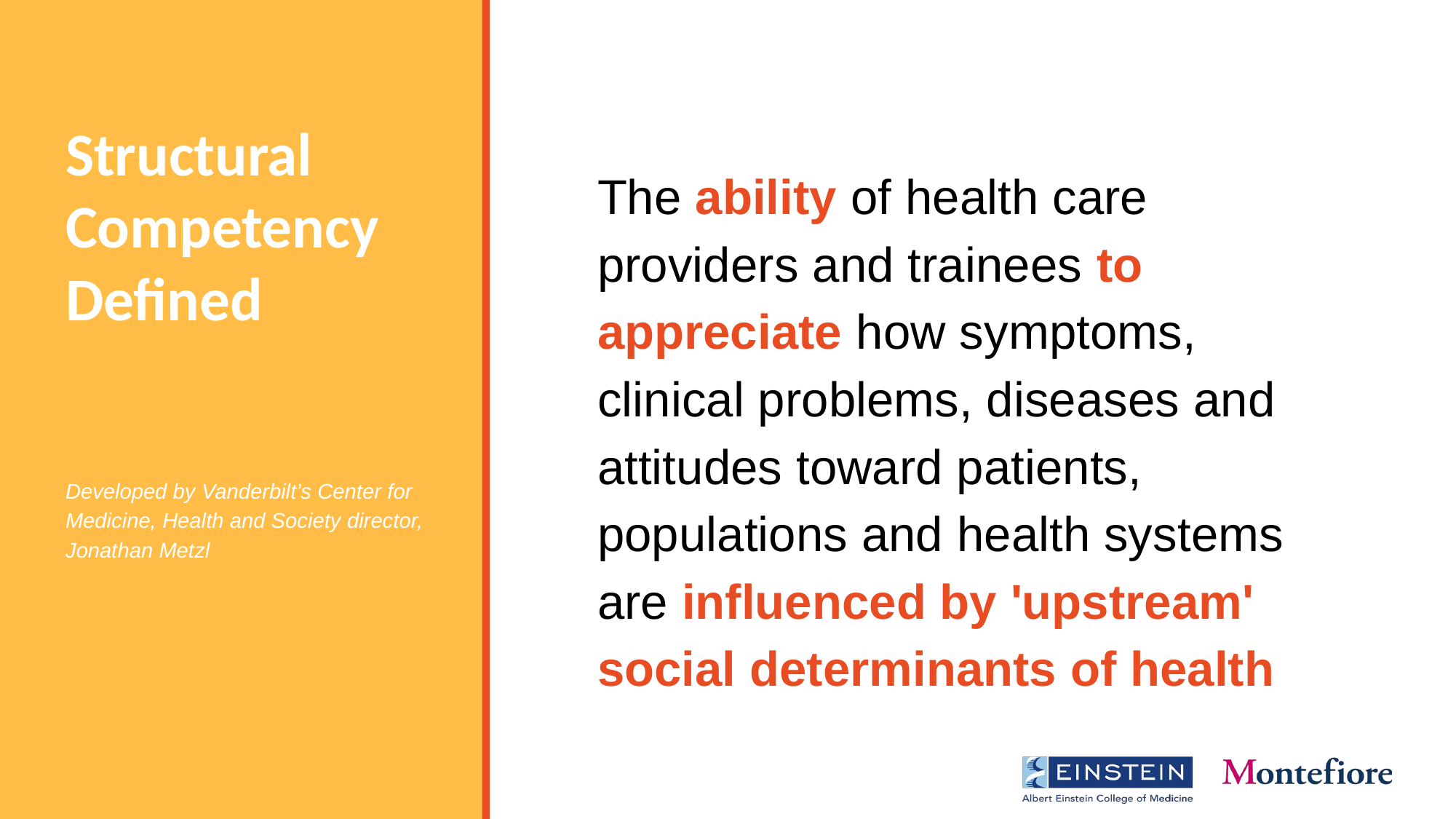

# Structural Competency Defined
The ability of health care providers and trainees to appreciate how symptoms, clinical problems, diseases and attitudes toward patients, populations and health systems are influenced by 'upstream' social determinants of health
Developed by Vanderbilt’s Center for Medicine, Health and Society director, Jonathan Metzl

## Slide 10
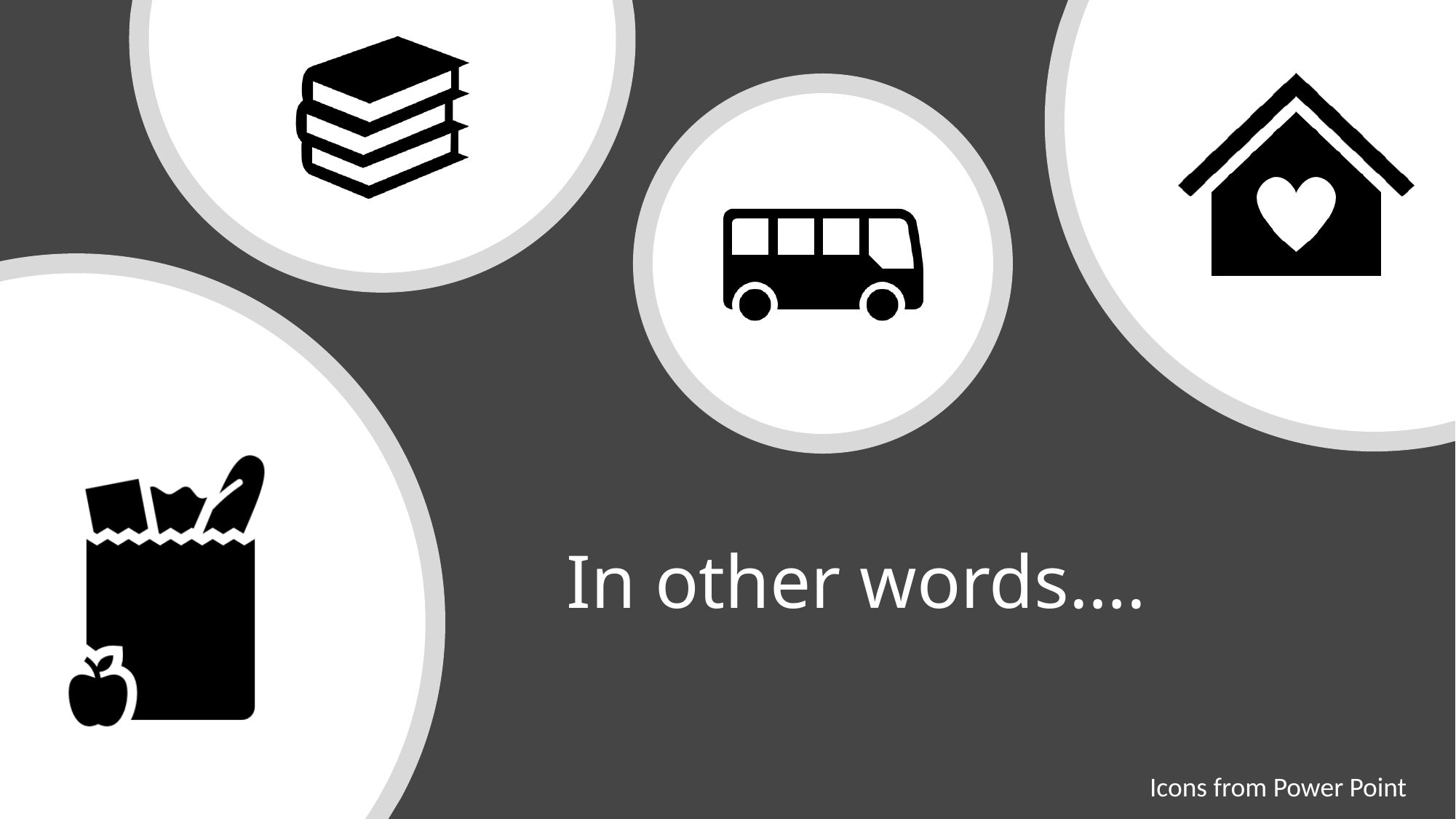

In other words….
Icons from Power Point

## Slide 11
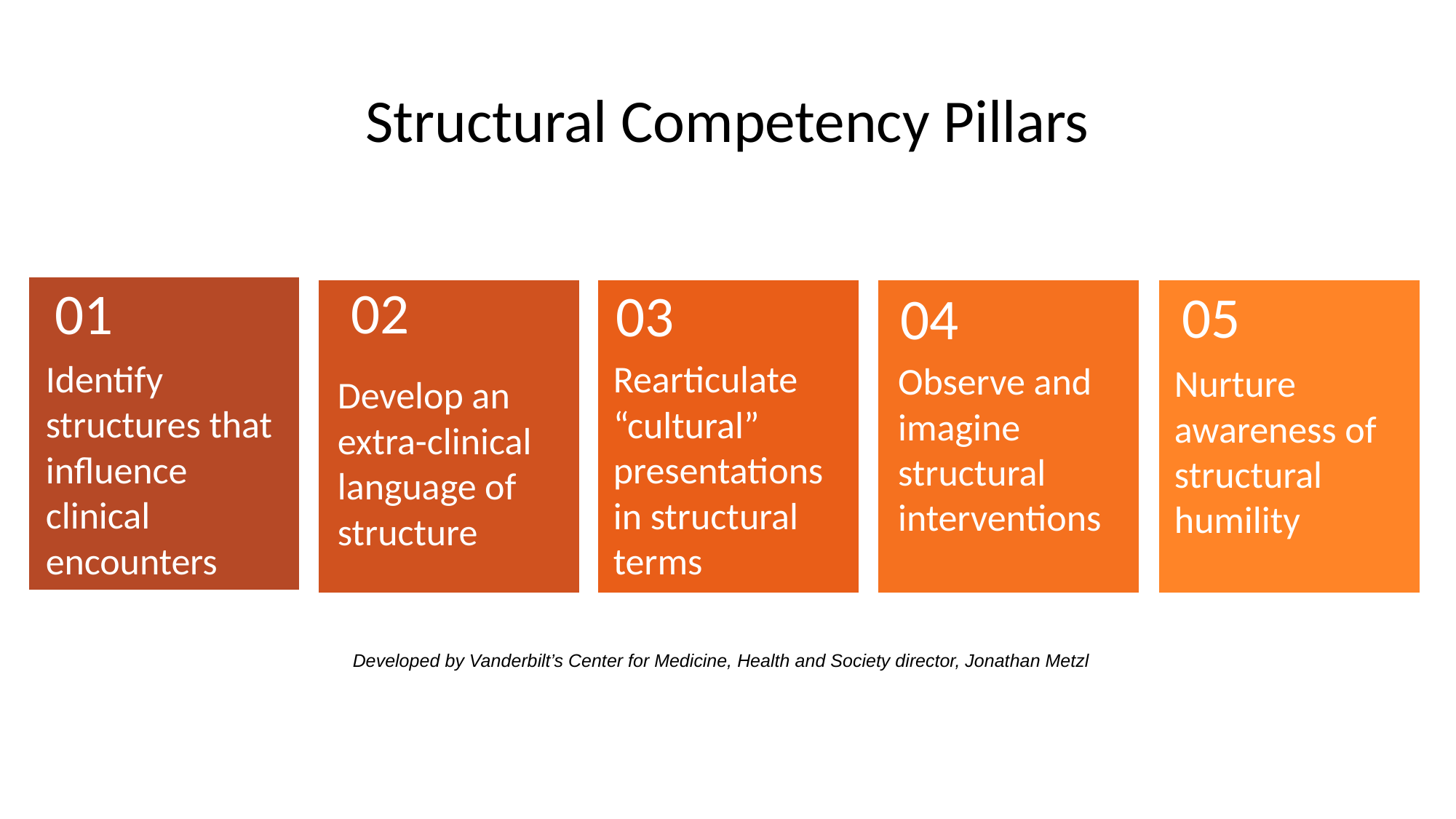

# Structural Competency Pillars
Identify structures that influence clinical encounters
Rearticulate “cultural” presentations in structural terms
Observe and imagine structural interventions
Nurture awareness of structural humility
Develop an extra-clinical language of structure
Developed by Vanderbilt’s Center for Medicine, Health and Society director, Jonathan Metzl

## Slide 12
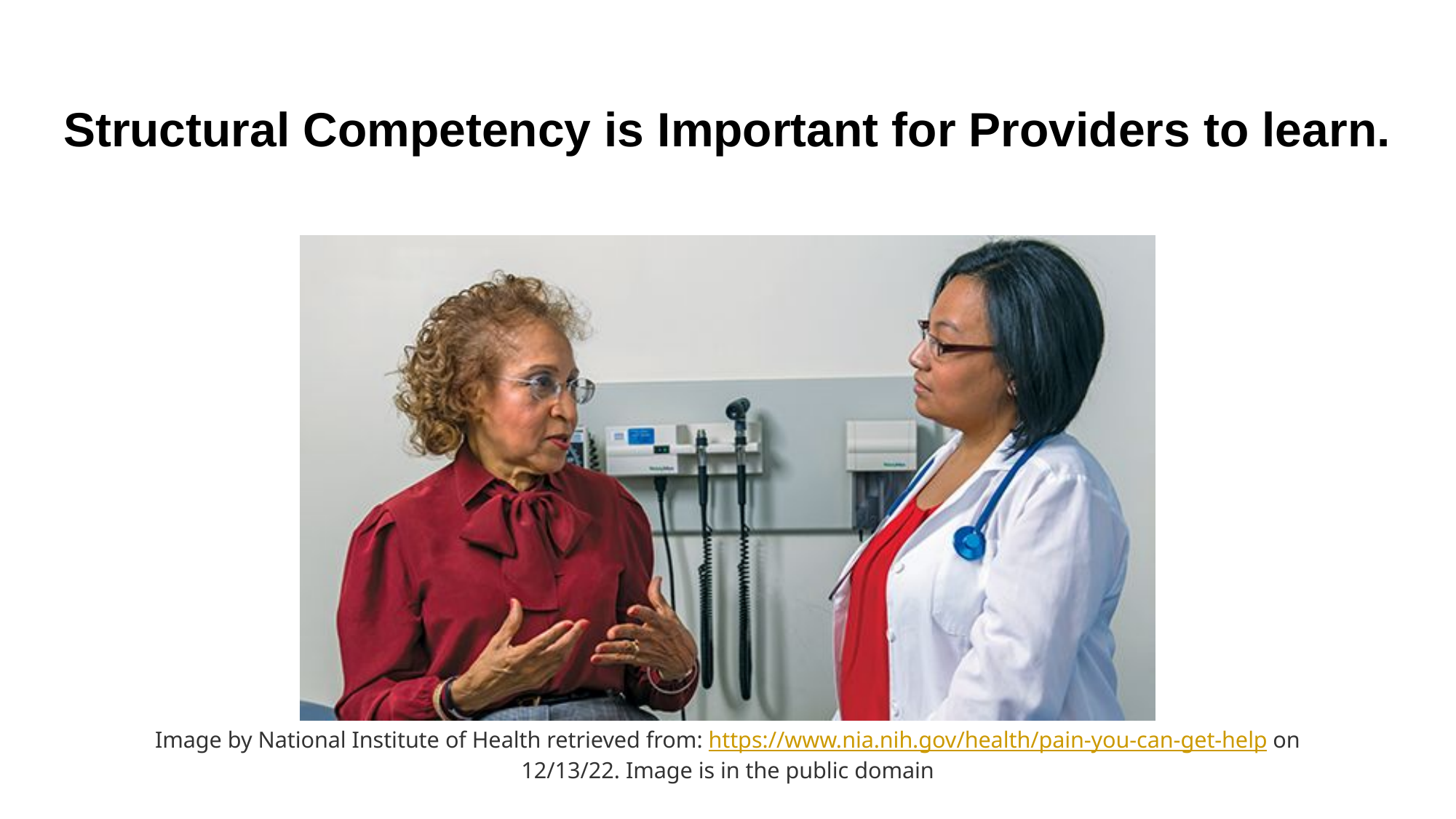

# Structural Competency is Important for Providers to learn.
Image by National Institute of Health retrieved from: https://www.nia.nih.gov/health/pain-you-can-get-help on 12/13/22. Image is in the public domain

## Slide 13
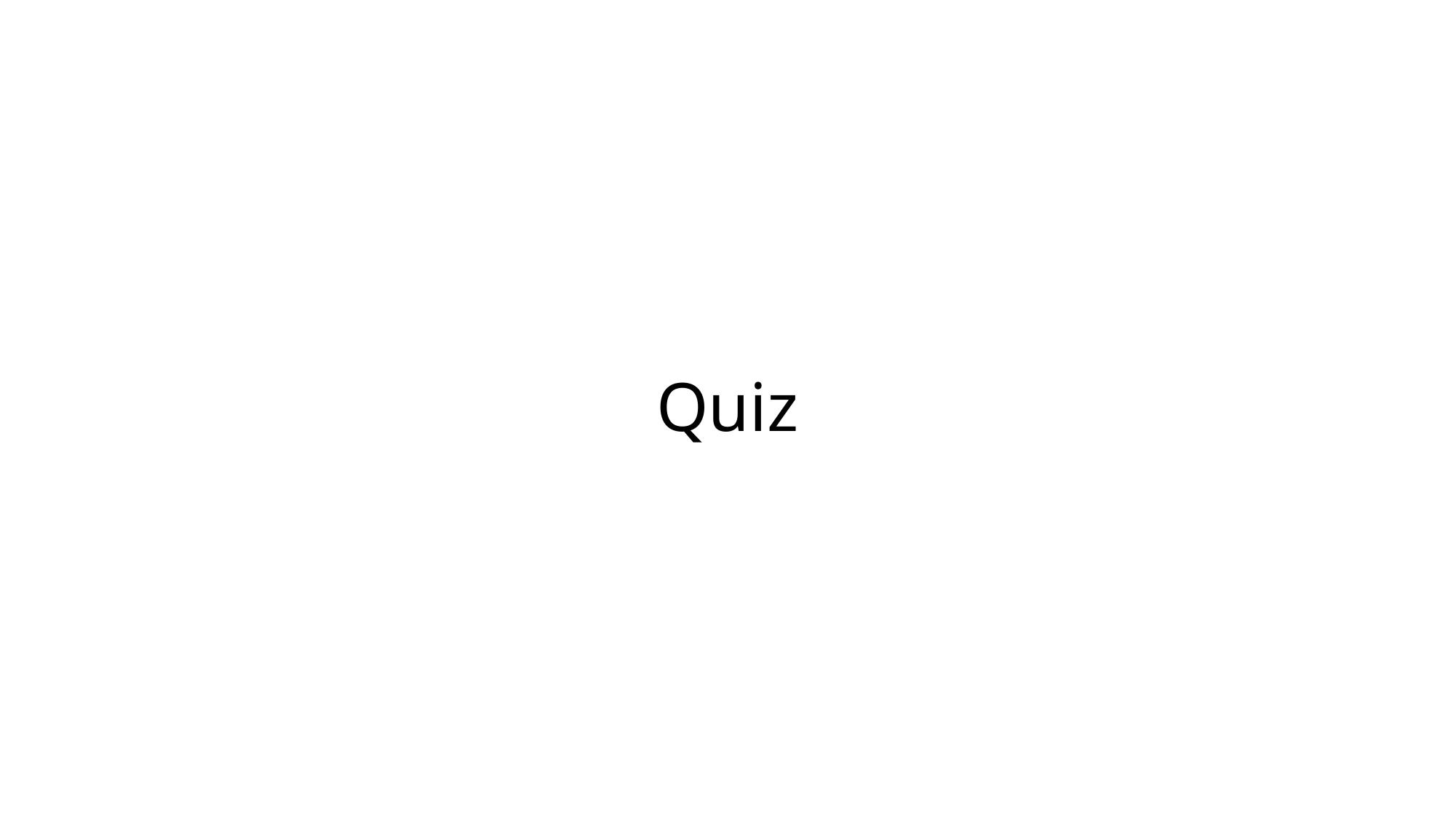

# Quiz

## Slide 14
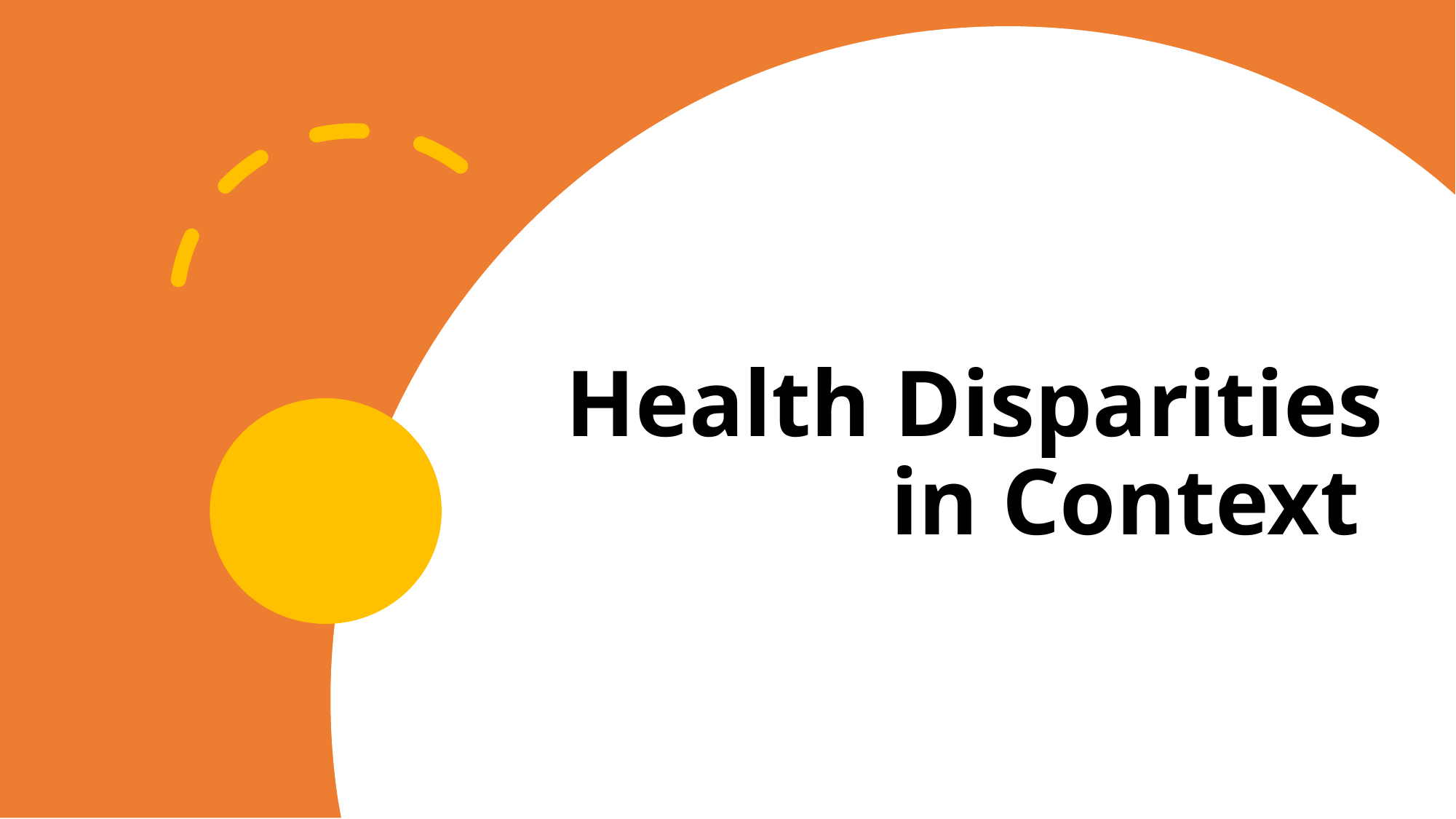

# Health Disparities in Context

## Slide 15
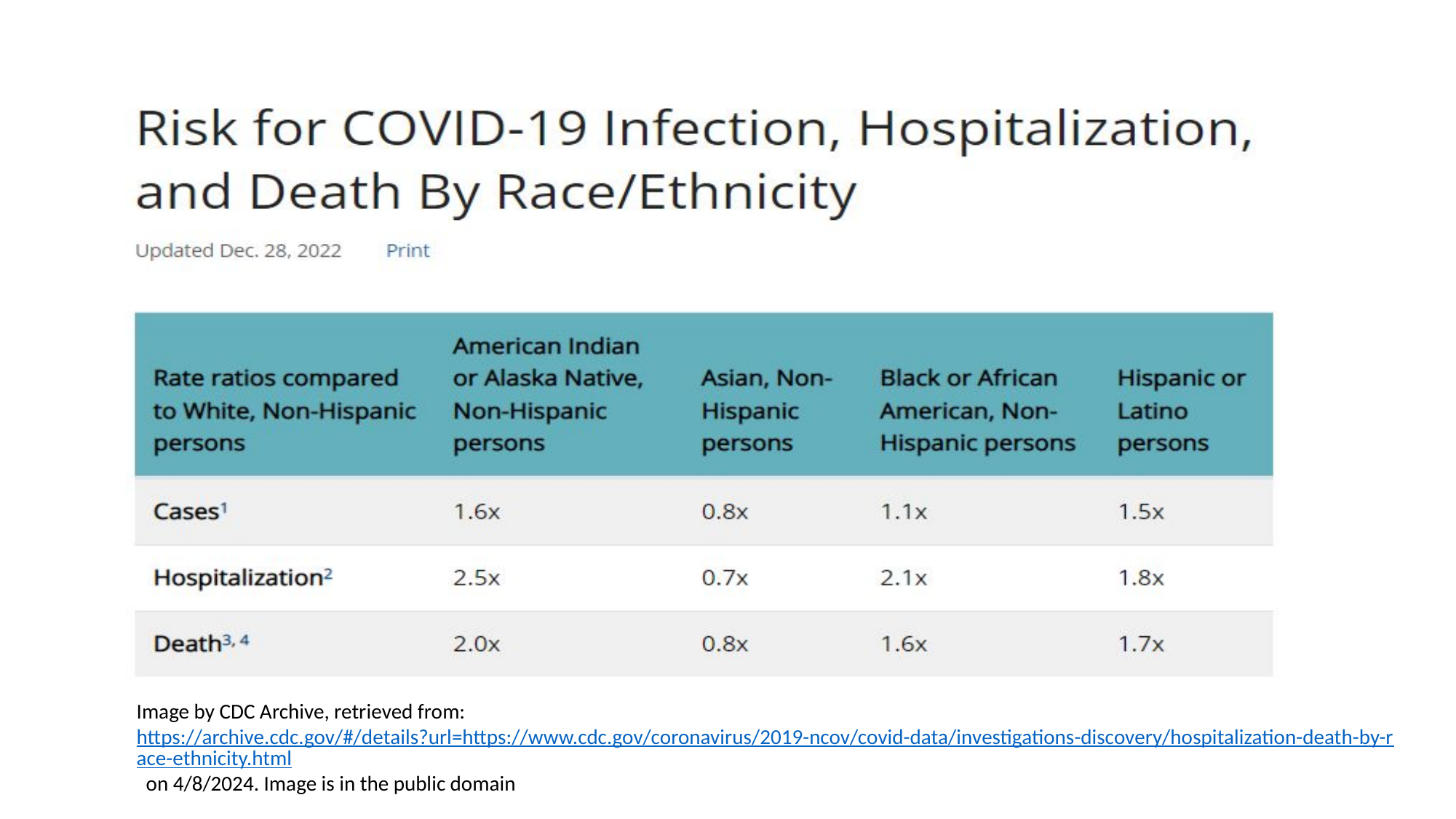

Image by CDC Archive, retrieved from: https://archive.cdc.gov/#/details?url=https://www.cdc.gov/coronavirus/2019-ncov/covid-data/investigations-discovery/hospitalization-death-by-race-ethnicity.html on 4/8/2024. Image is in the public domain

## Slide 16
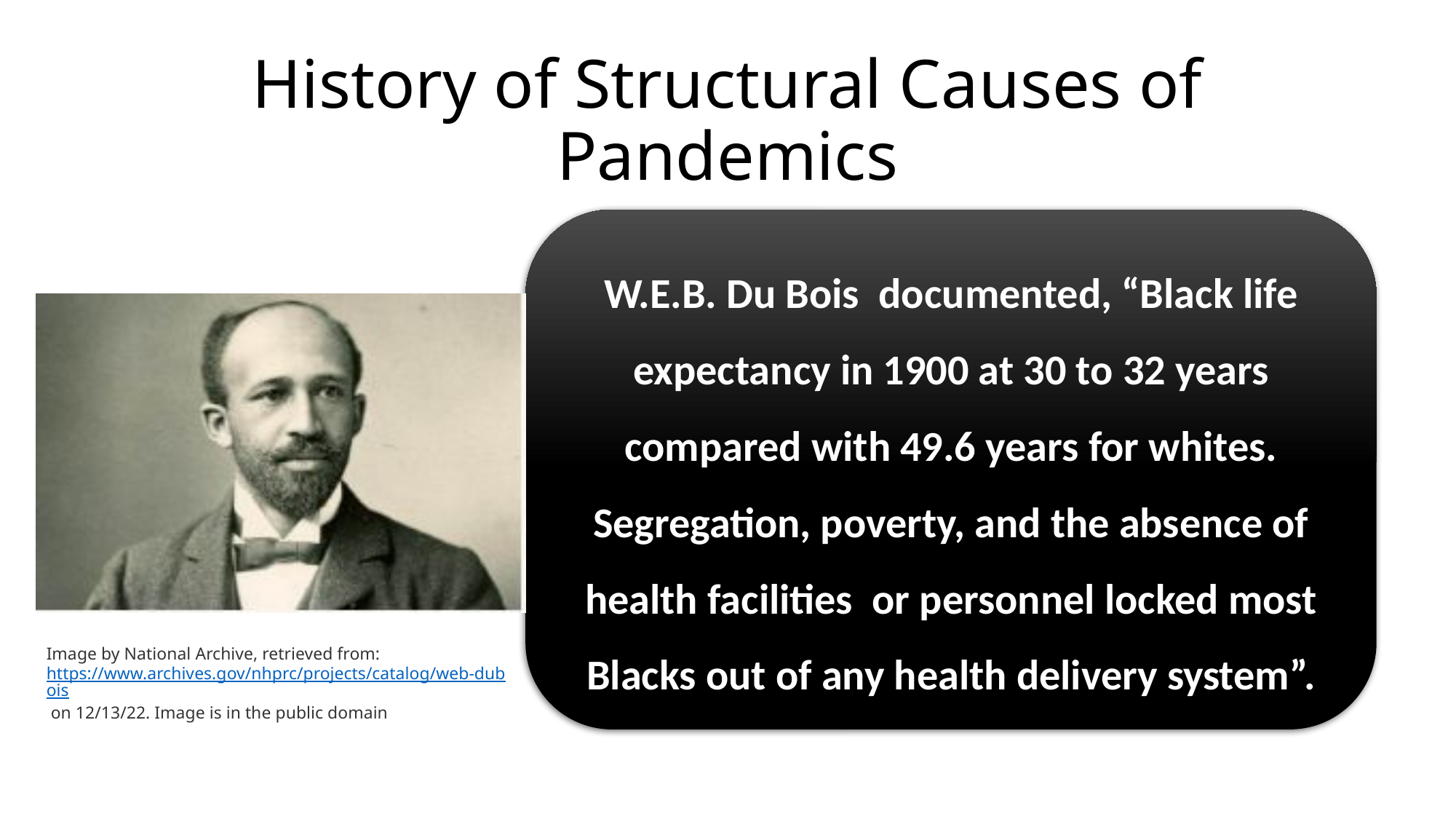

# History of Structural Causes of Pandemics
W.E.B. Du Bois documented, “Black life expectancy in 1900 at 30 to 32 years compared with 49.6 years for whites. Segregation, poverty, and the absence of health facilities or personnel locked most Blacks out of any health delivery system”.
Image by National Archive, retrieved from: https://www.archives.gov/nhprc/projects/catalog/web-dubois on 12/13/22. Image is in the public domain

## Slide 17
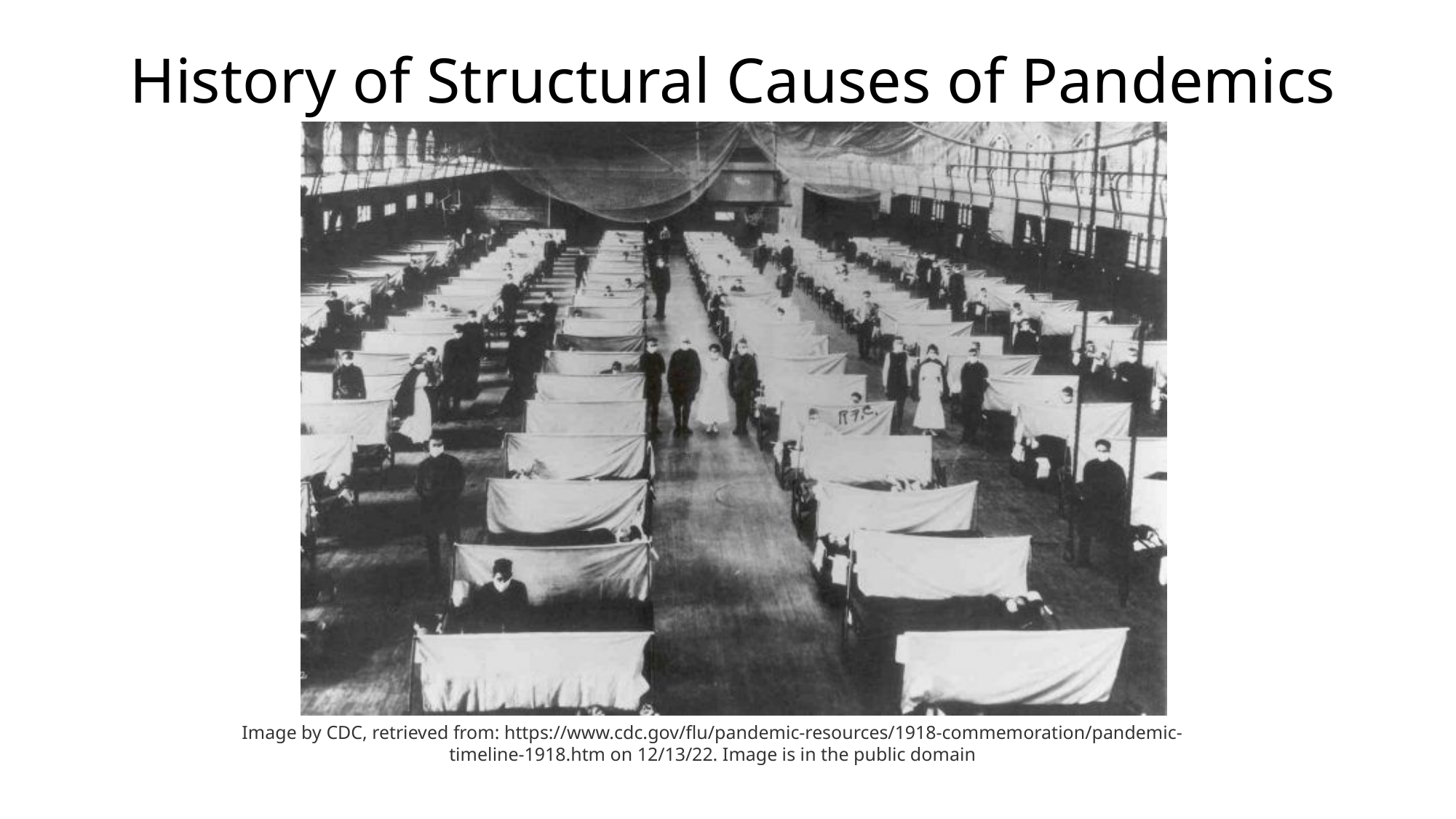

# History of Structural Causes of Pandemics
Image by CDC, retrieved from: https://www.cdc.gov/flu/pandemic-resources/1918-commemoration/pandemic-timeline-1918.htm on 12/13/22. Image is in the public domain

## Slide 18
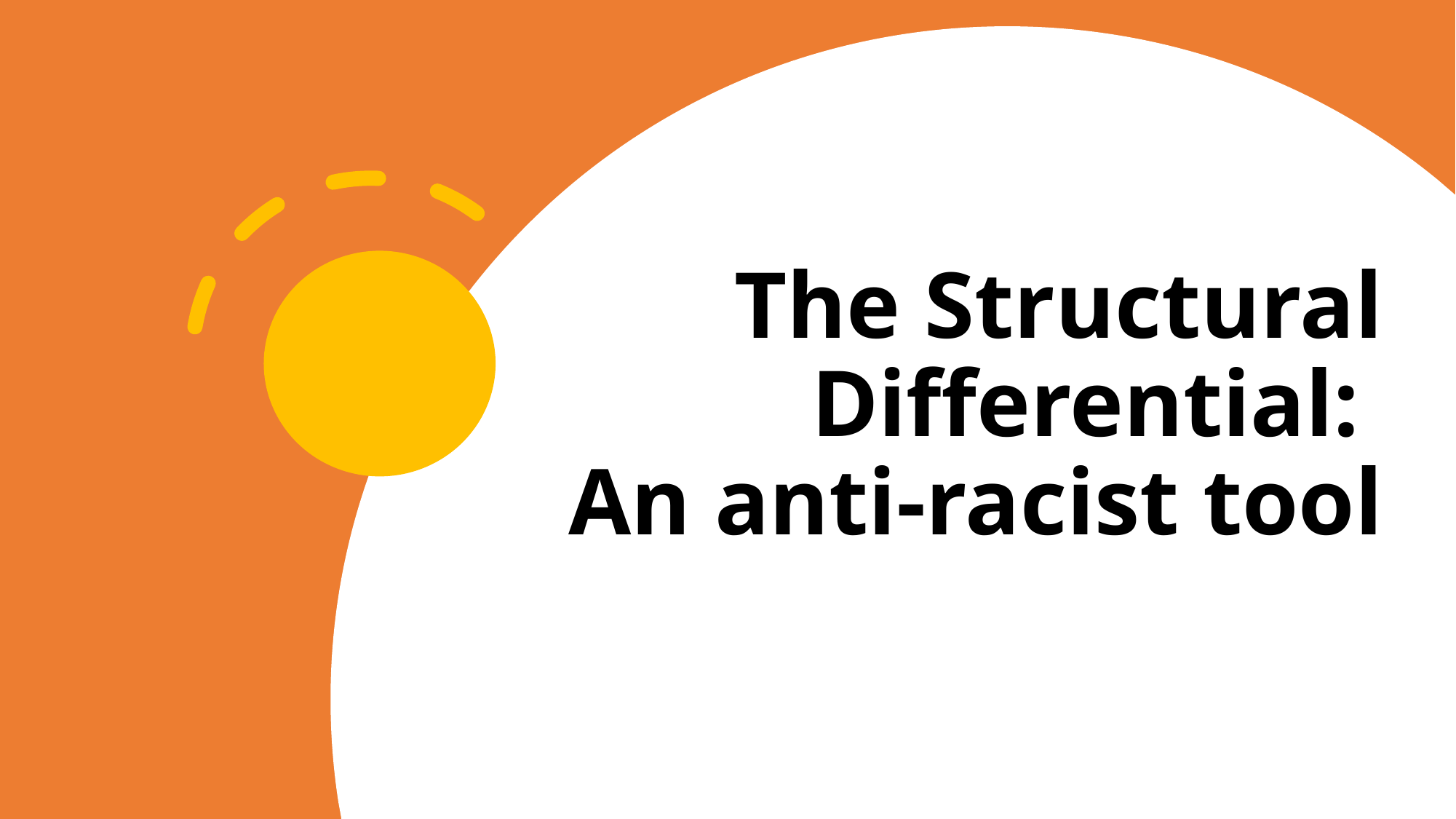

# The Structural Differential: An anti-racist tool

## Slide 19
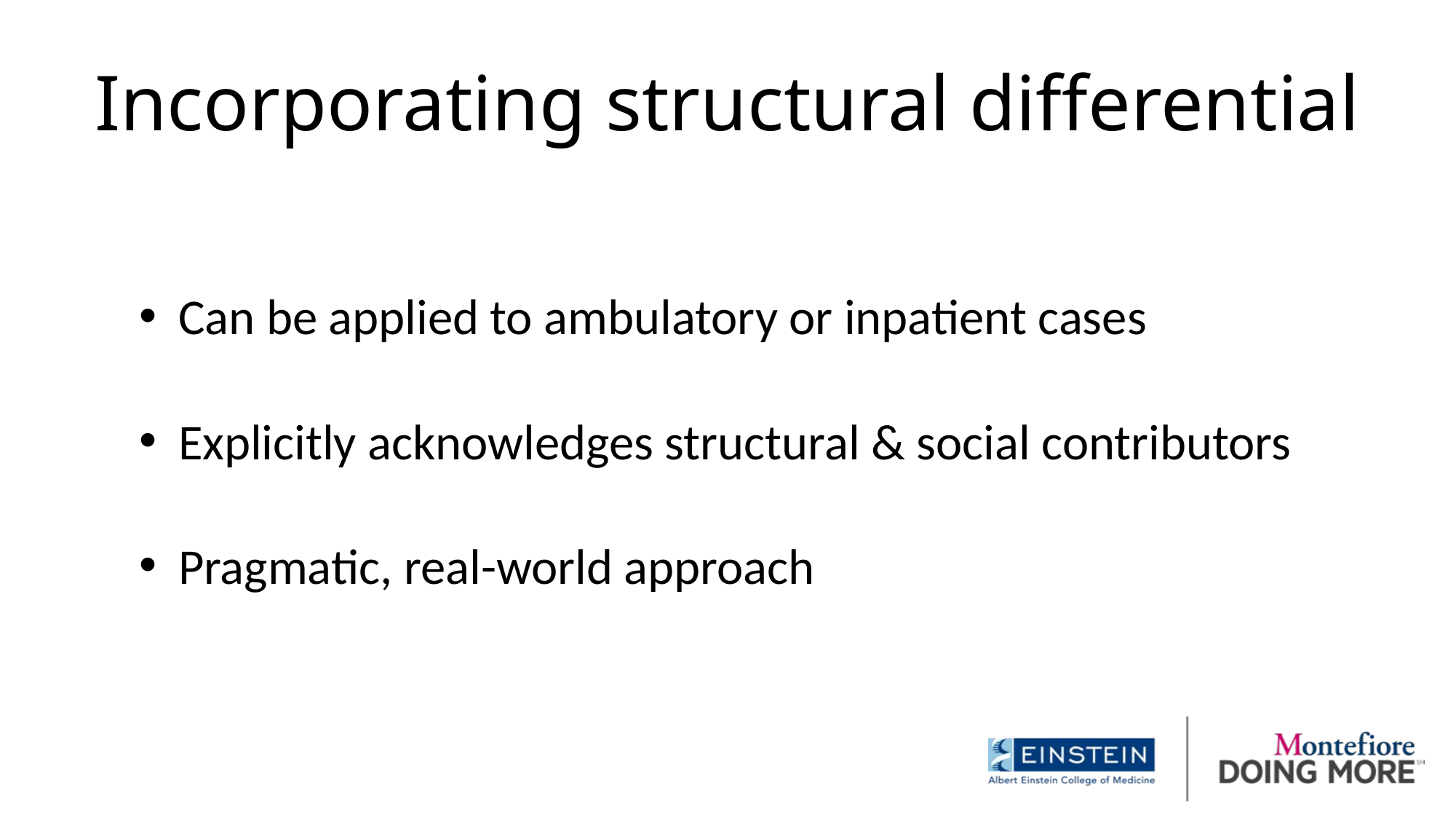

# Incorporating structural differential
Can be applied to ambulatory or inpatient cases
Explicitly acknowledges structural & social contributors
Pragmatic, real-world approach

## Slide 20
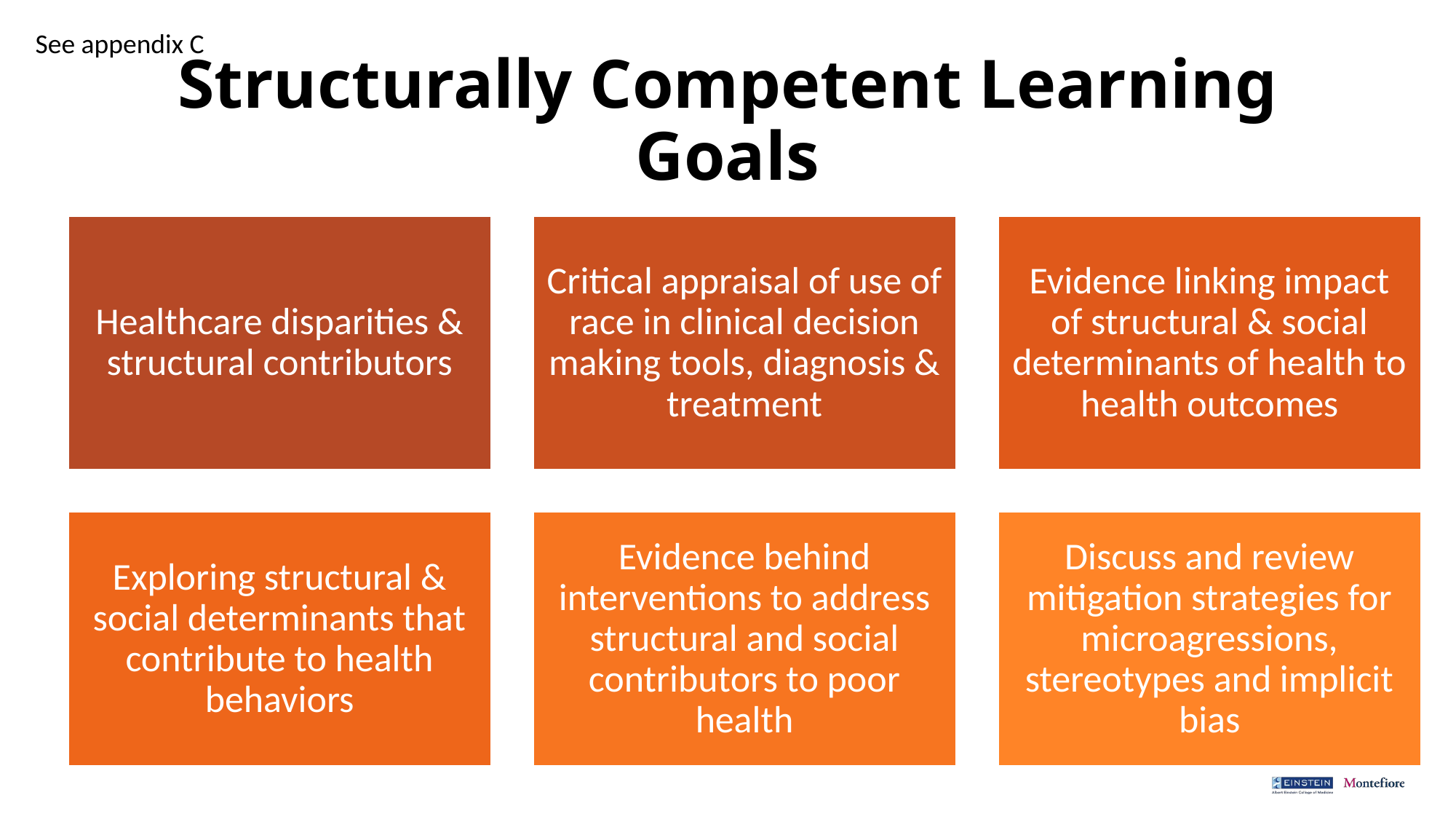

See appendix C
# Structurally Competent Learning Goals

## Slide 21
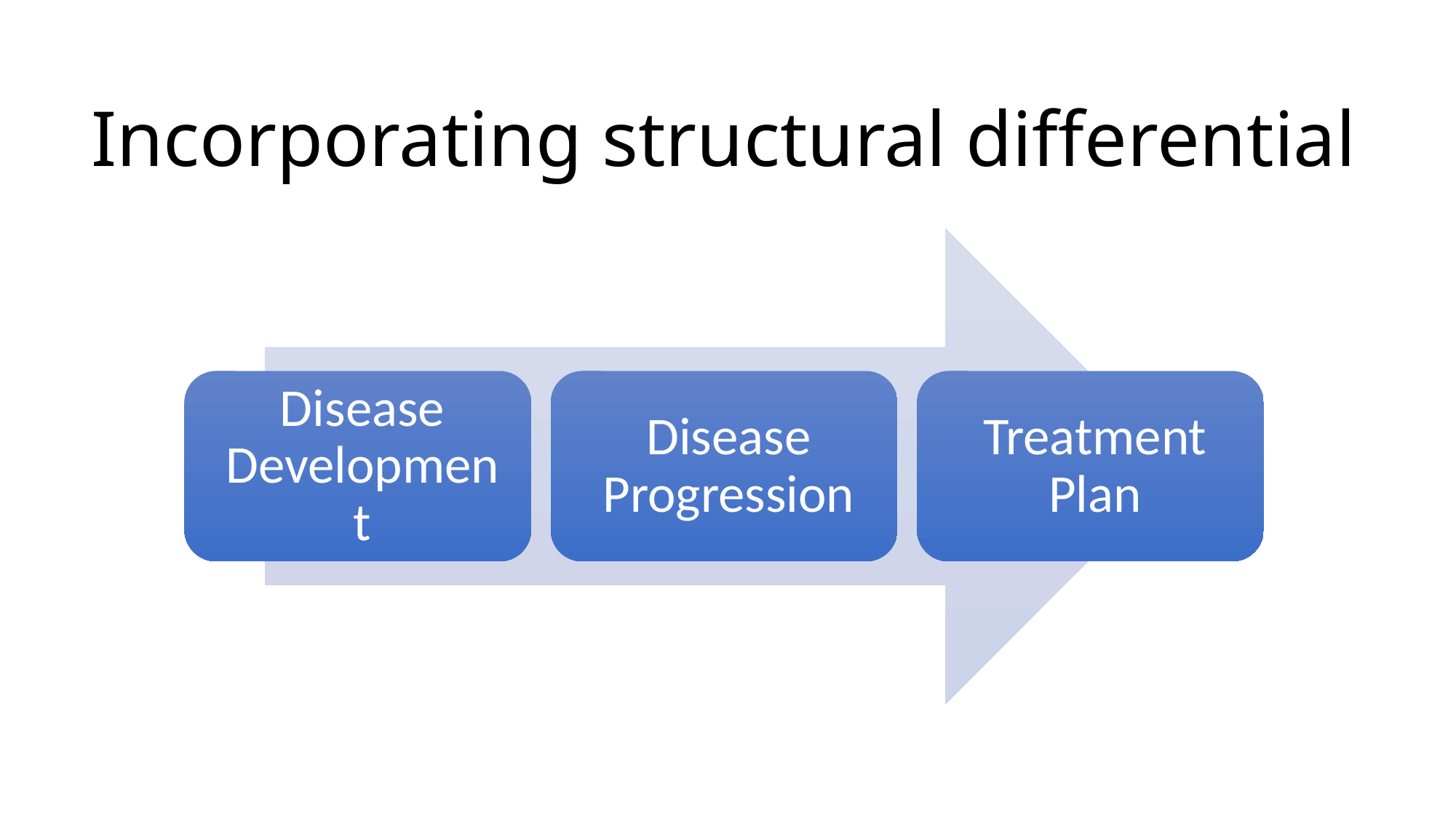

Incorporating structural differential

## Slide 22
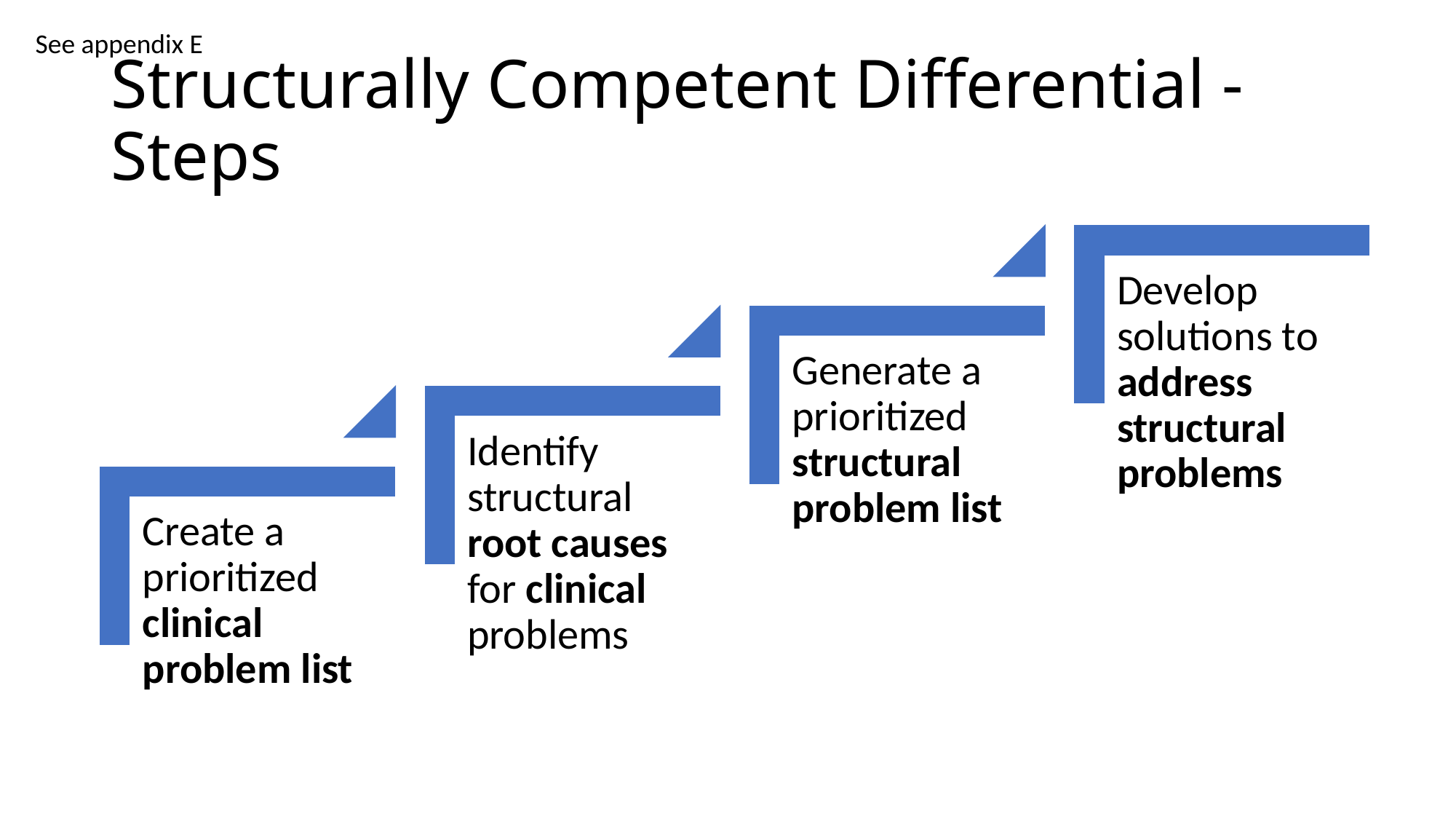

See appendix E
# Structurally Competent Differential - Steps

## Slide 23
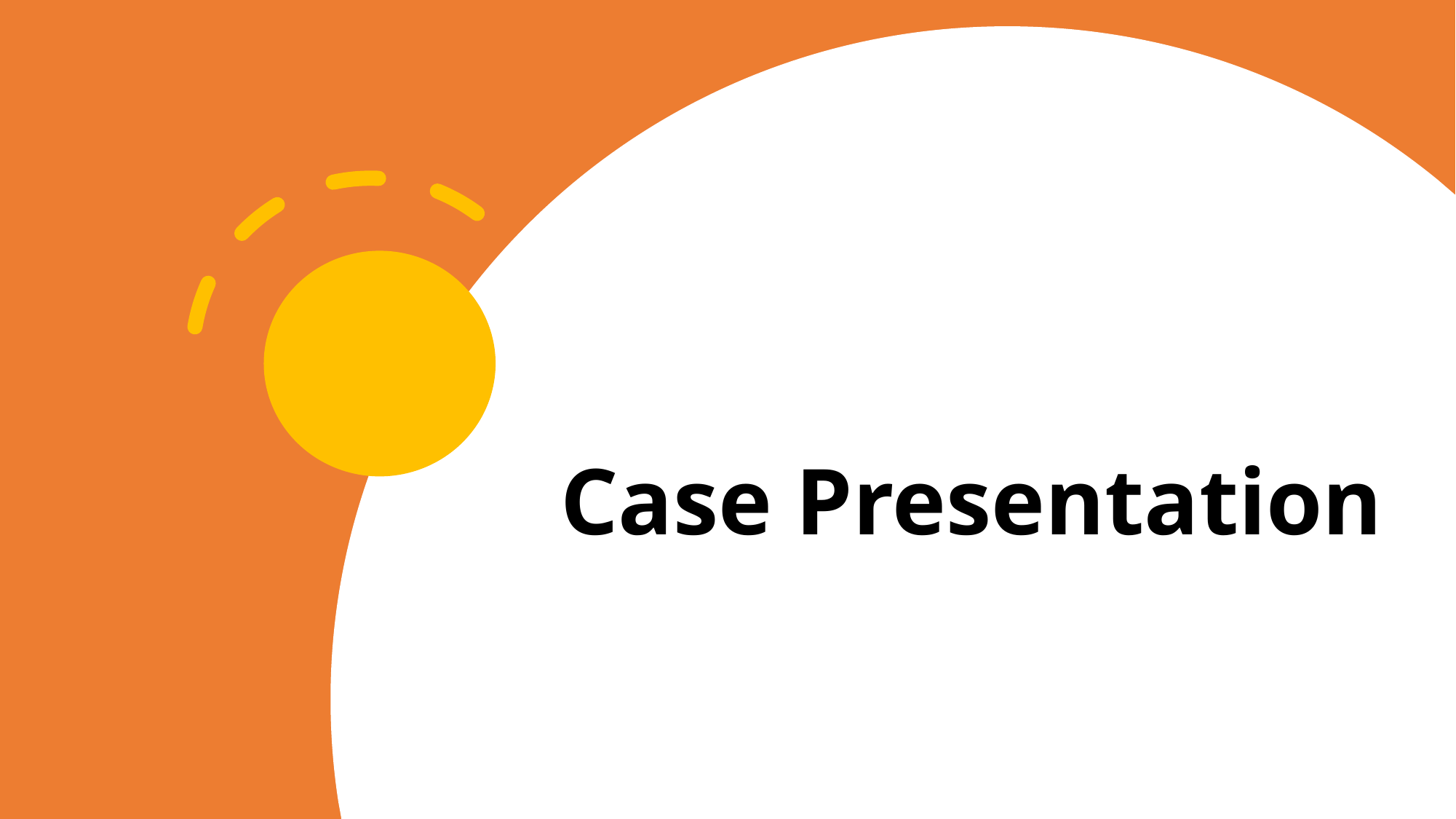

# Case Presentation

## Slide 24
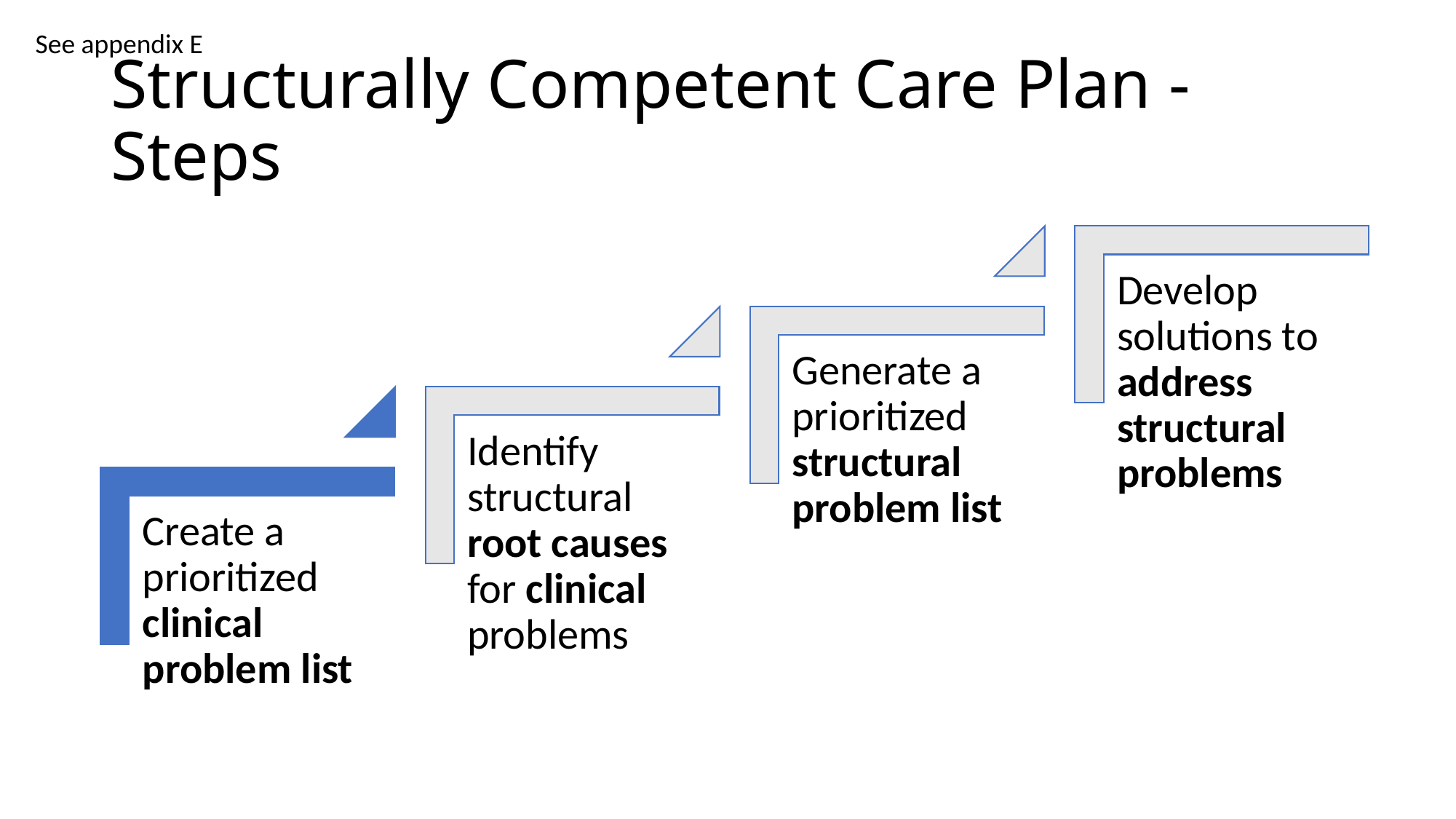

See appendix E
# Structurally Competent Care Plan - Steps

## Slide 25
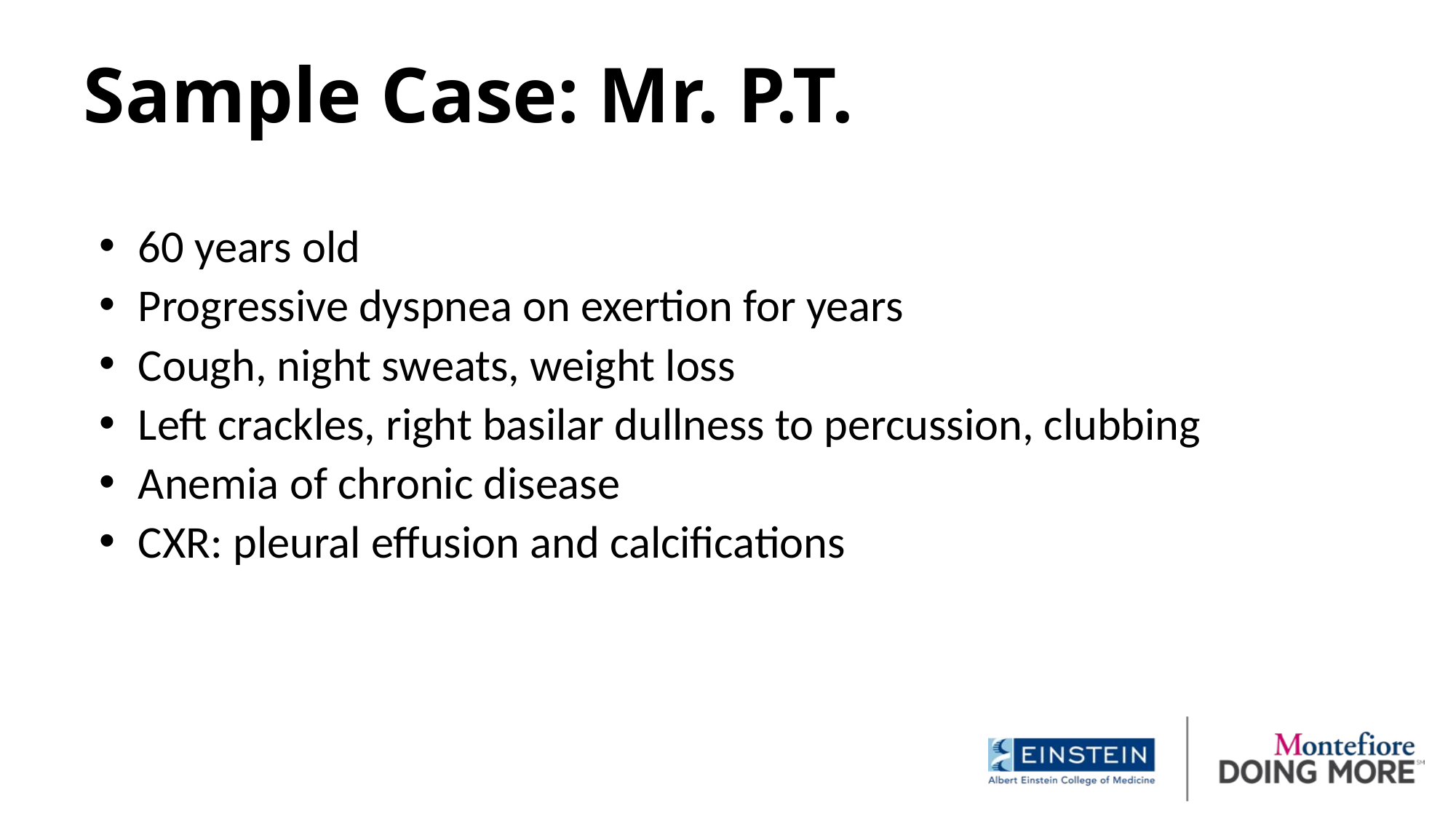

# Sample Case: Mr. P.T.
60 years old
Progressive dyspnea on exertion for years
Cough, night sweats, weight loss
Left crackles, right basilar dullness to percussion, clubbing
Anemia of chronic disease
CXR: pleural effusion and calcifications

## Slide 26
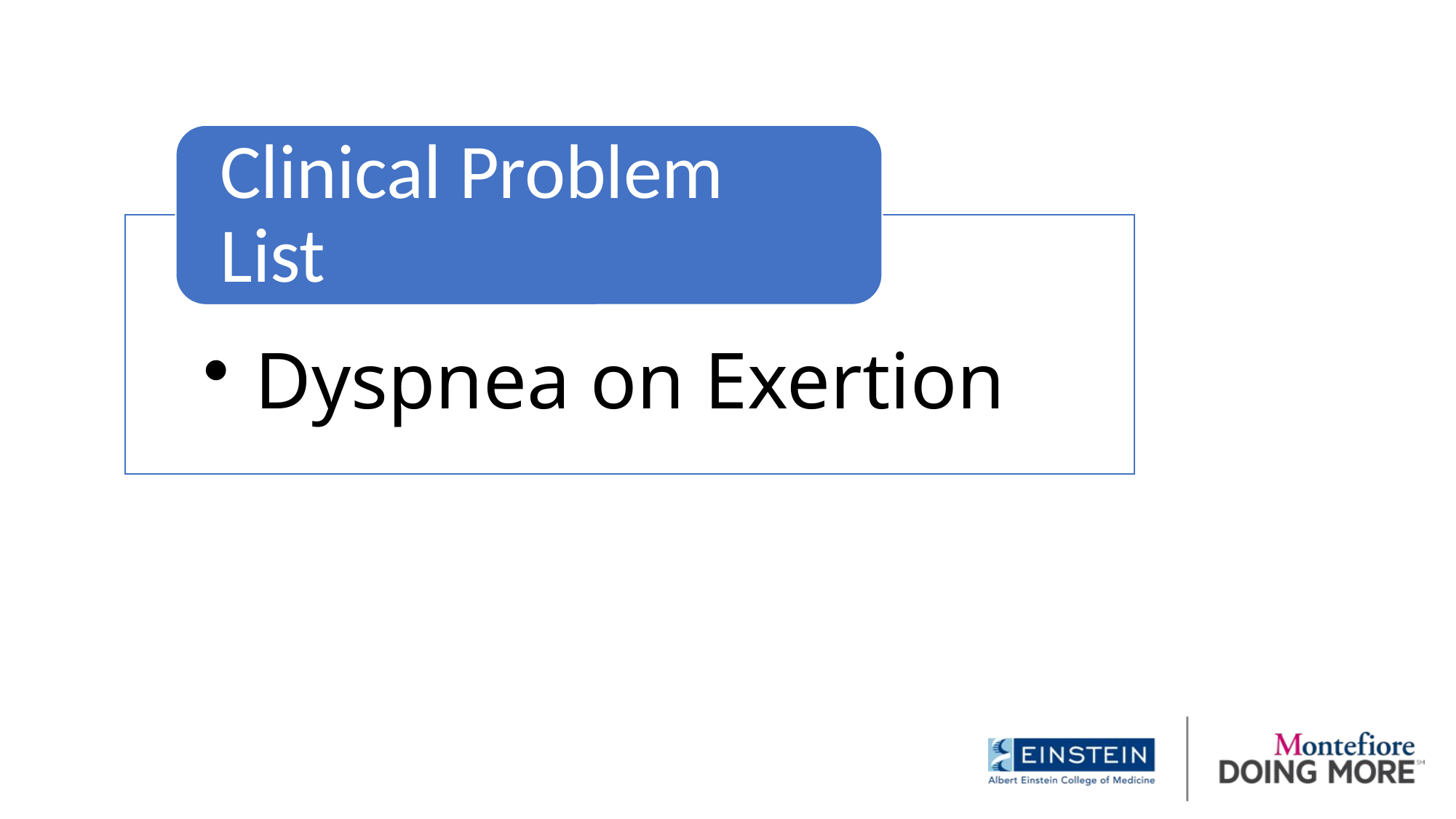

## Slide 27
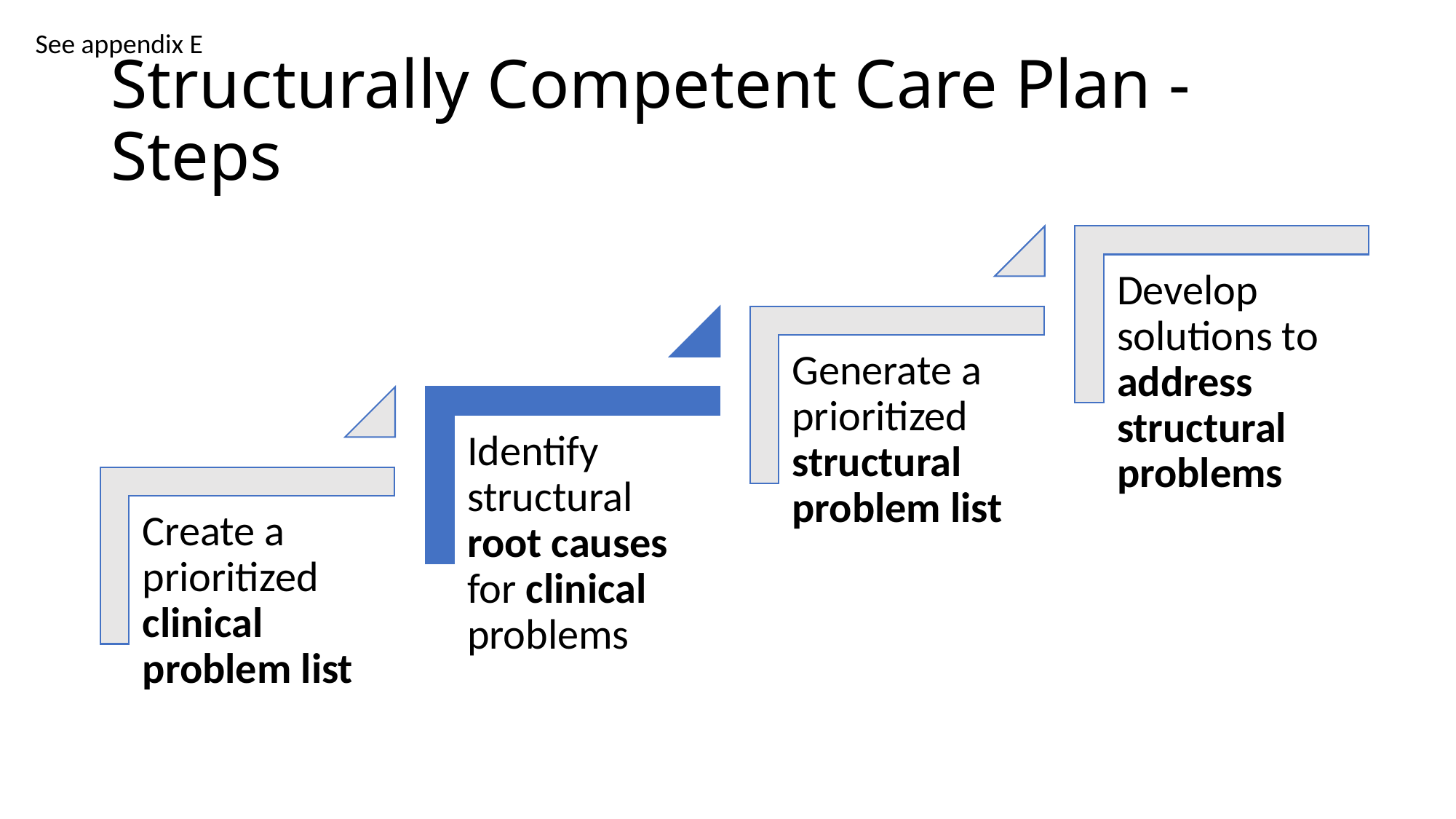

See appendix E
# Structurally Competent Care Plan - Steps

## Slide 28
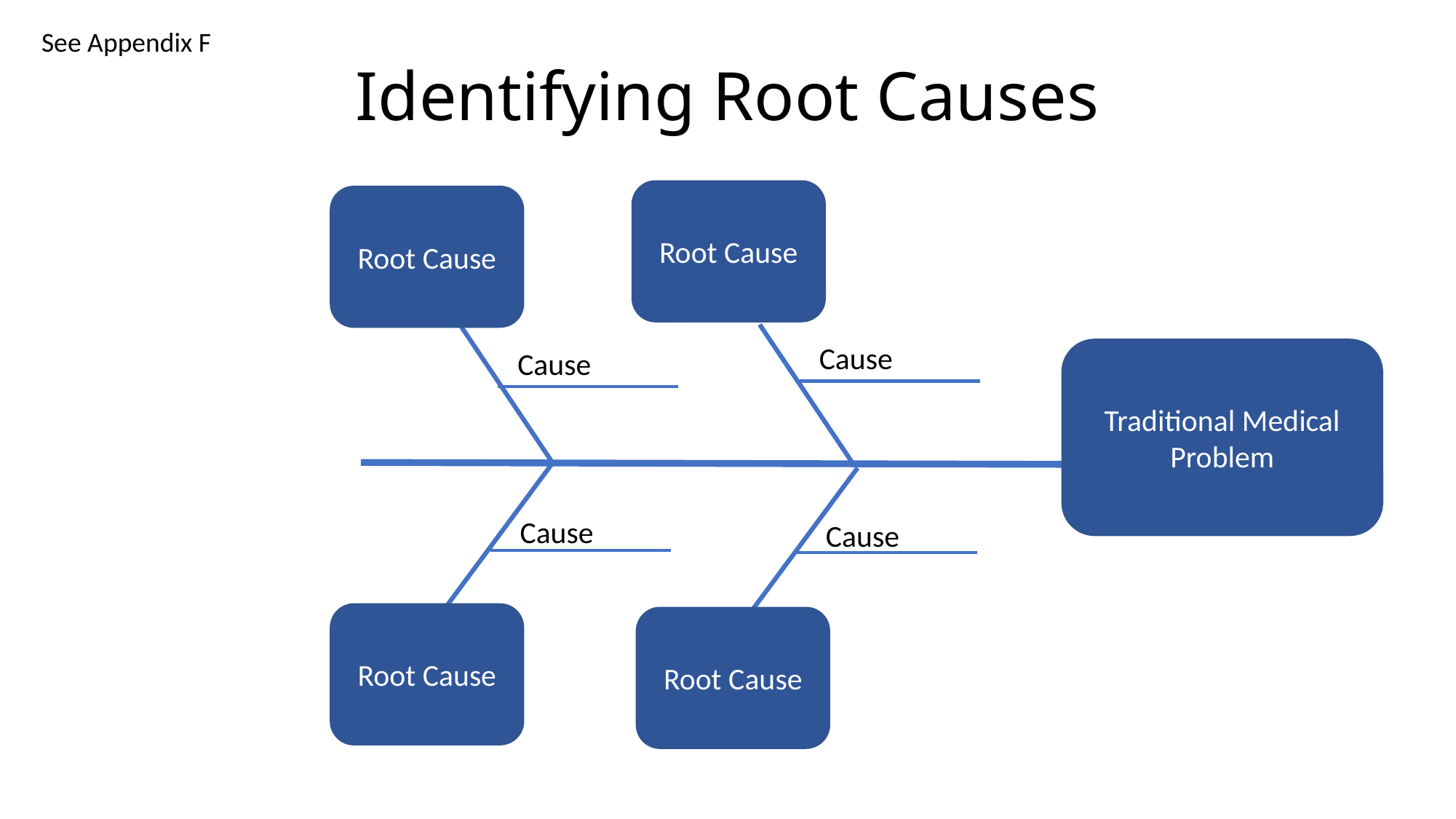

See Appendix F
# Identifying Root Causes
Root Cause
Cause
Root Cause
Cause
Traditional Medical Problem
Cause
Cause
Root Cause
Root Cause

## Slide 29
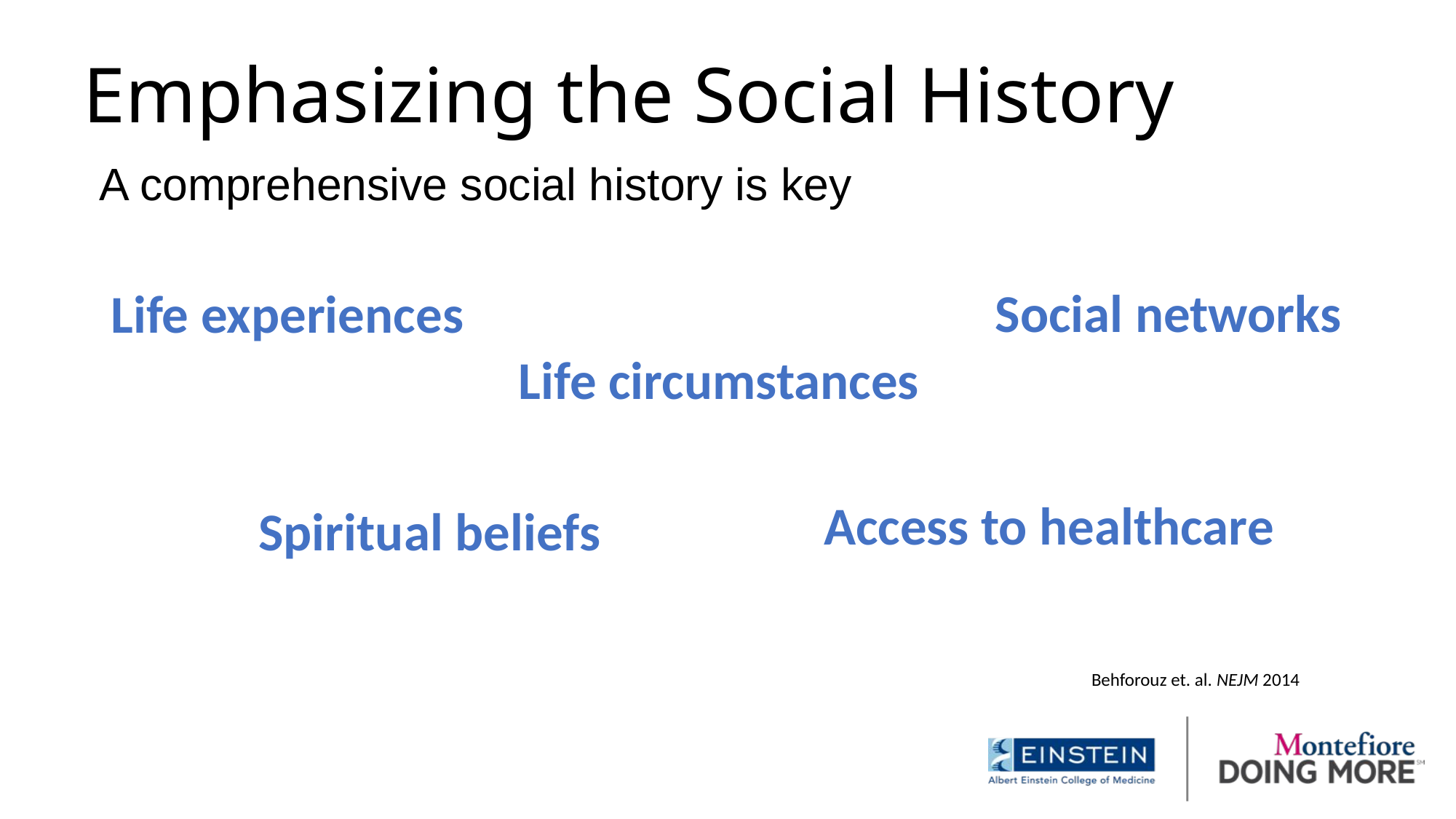

# Emphasizing the Social History
A comprehensive social history is key
Social networks
Life experiences
Life circumstances
Access to healthcare
Spiritual beliefs
Behforouz et. al. NEJM 2014

## Slide 30
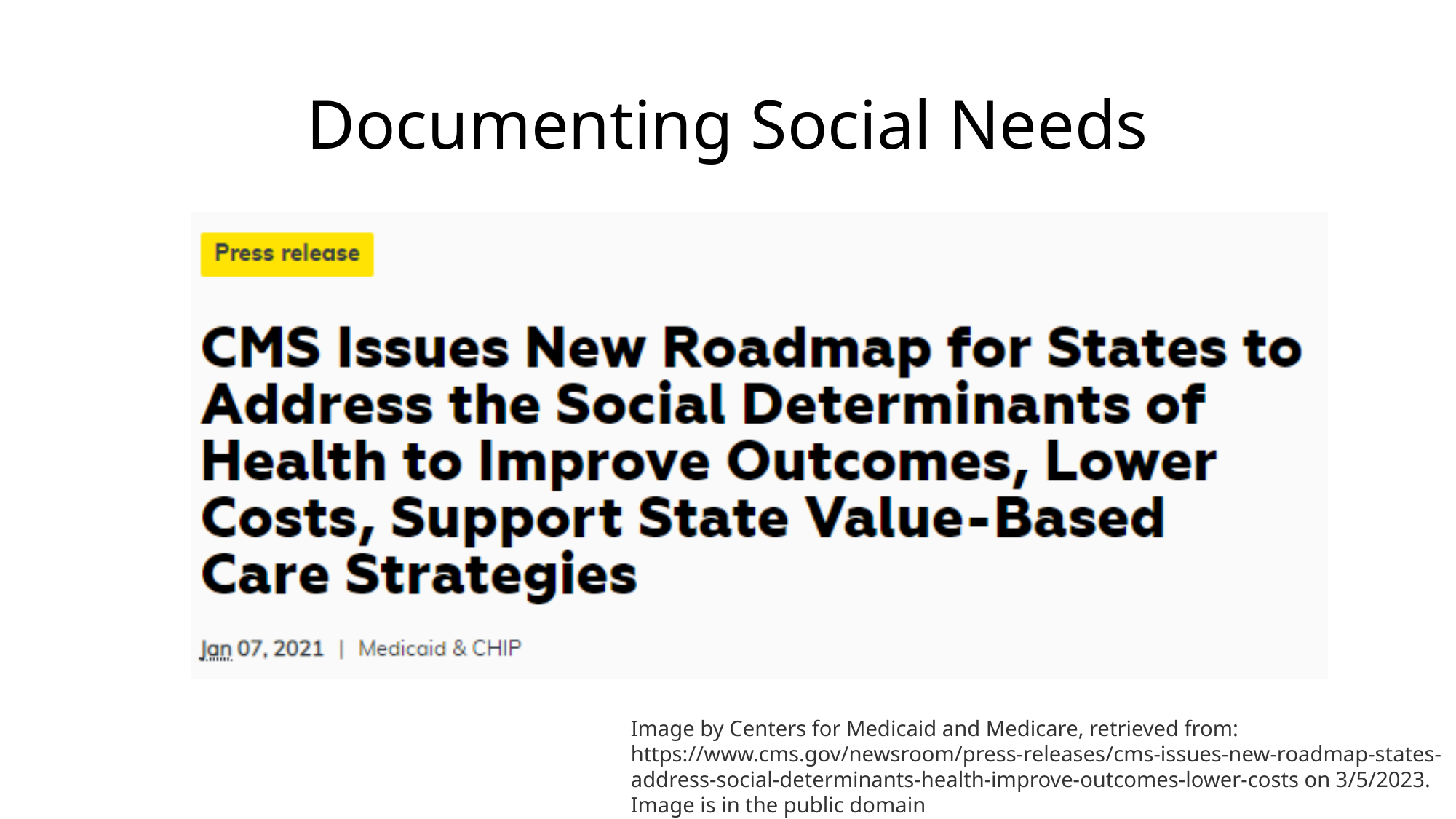

# Documenting Social Needs
Image by Centers for Medicaid and Medicare, retrieved from: https://www.cms.gov/newsroom/press-releases/cms-issues-new-roadmap-states-address-social-determinants-health-improve-outcomes-lower-costs on 3/5/2023. Image is in the public domain

## Slide 31
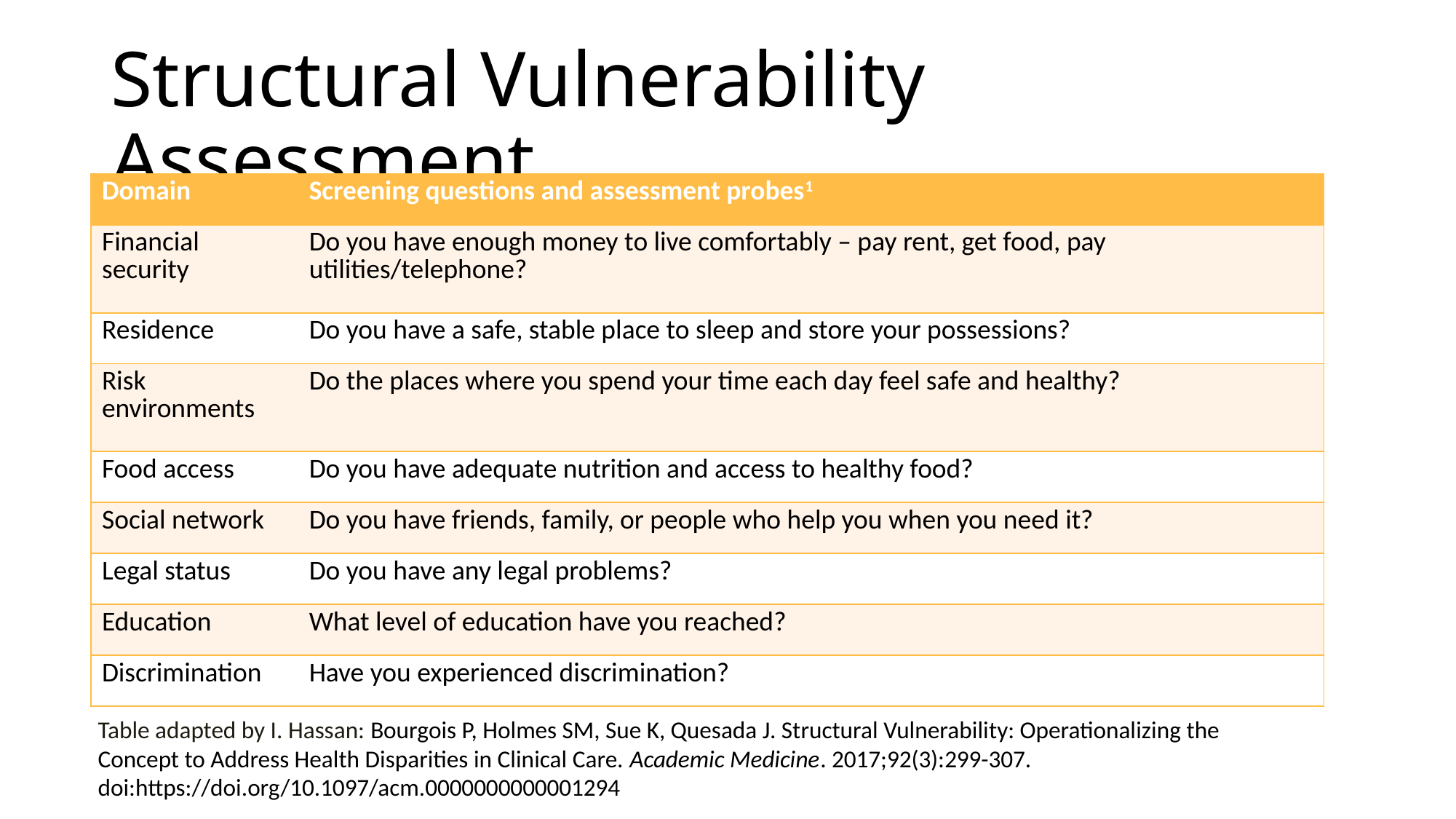

# Structural Vulnerability Assessment
| Domain | Screening questions and assessment probes1 |
| --- | --- |
| Financial security | Do you have enough money to live comfortably – pay rent, get food, pay utilities/telephone? |
| Residence | Do you have a safe, stable place to sleep and store your possessions? |
| Risk environments | Do the places where you spend your time each day feel safe and healthy? |
| Food access | Do you have adequate nutrition and access to healthy food? |
| Social network | Do you have friends, family, or people who help you when you need it? |
| Legal status | Do you have any legal problems? |
| Education | What level of education have you reached? |
| Discrimination | Have you experienced discrimination? |
Table adapted by I. Hassan: Bourgois P, Holmes SM, Sue K, Quesada J. Structural Vulnerability: Operationalizing the Concept to Address Health Disparities in Clinical Care. Academic Medicine. 2017;92(3):299-307. doi:https://doi.org/10.1097/acm.0000000000001294
‌

## Slide 32
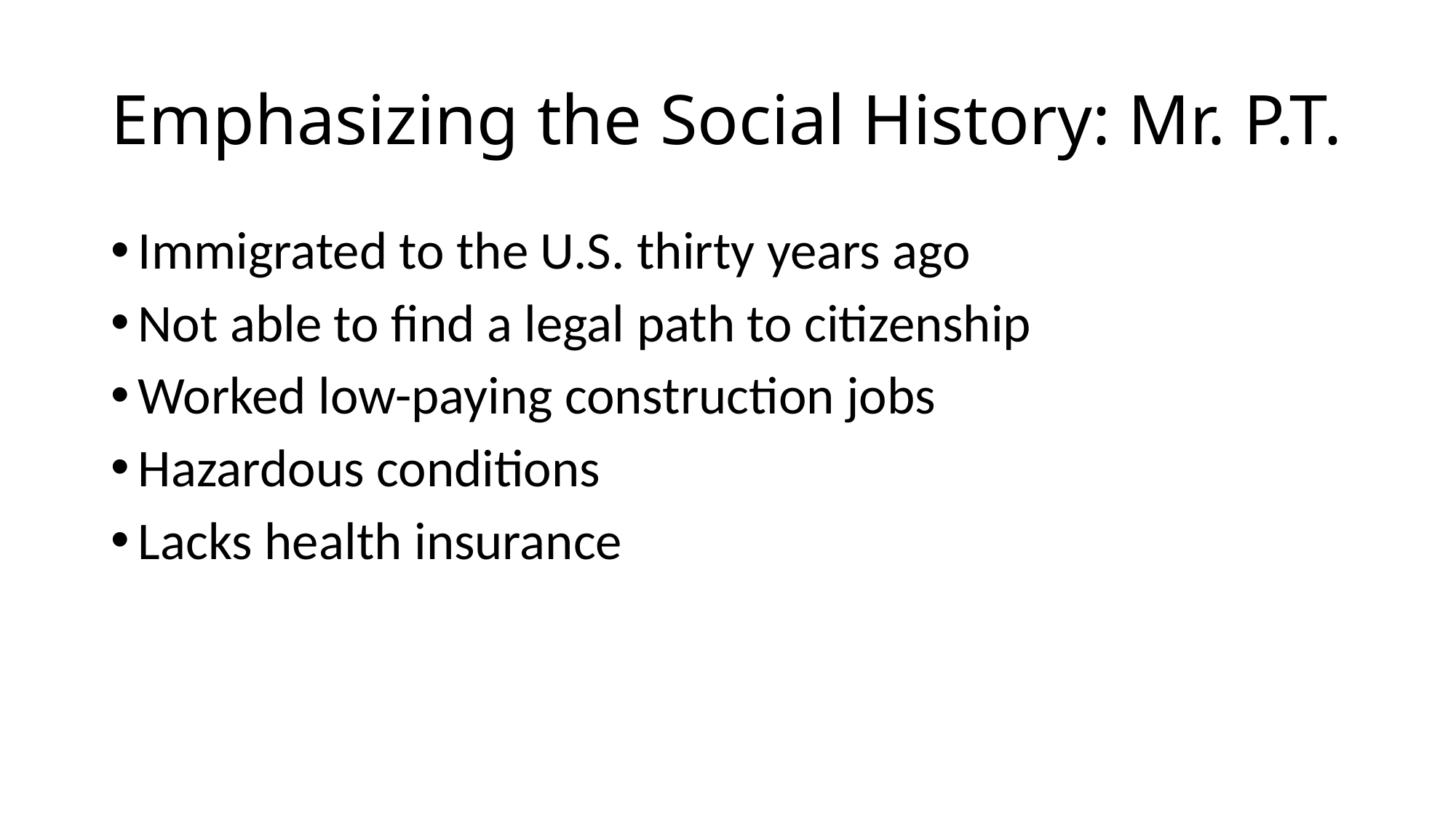

# Emphasizing the Social History: Mr. P.T.
Immigrated to the U.S. thirty years ago
Not able to find a legal path to citizenship
Worked low-paying construction jobs
Hazardous conditions
Lacks health insurance

## Slide 33
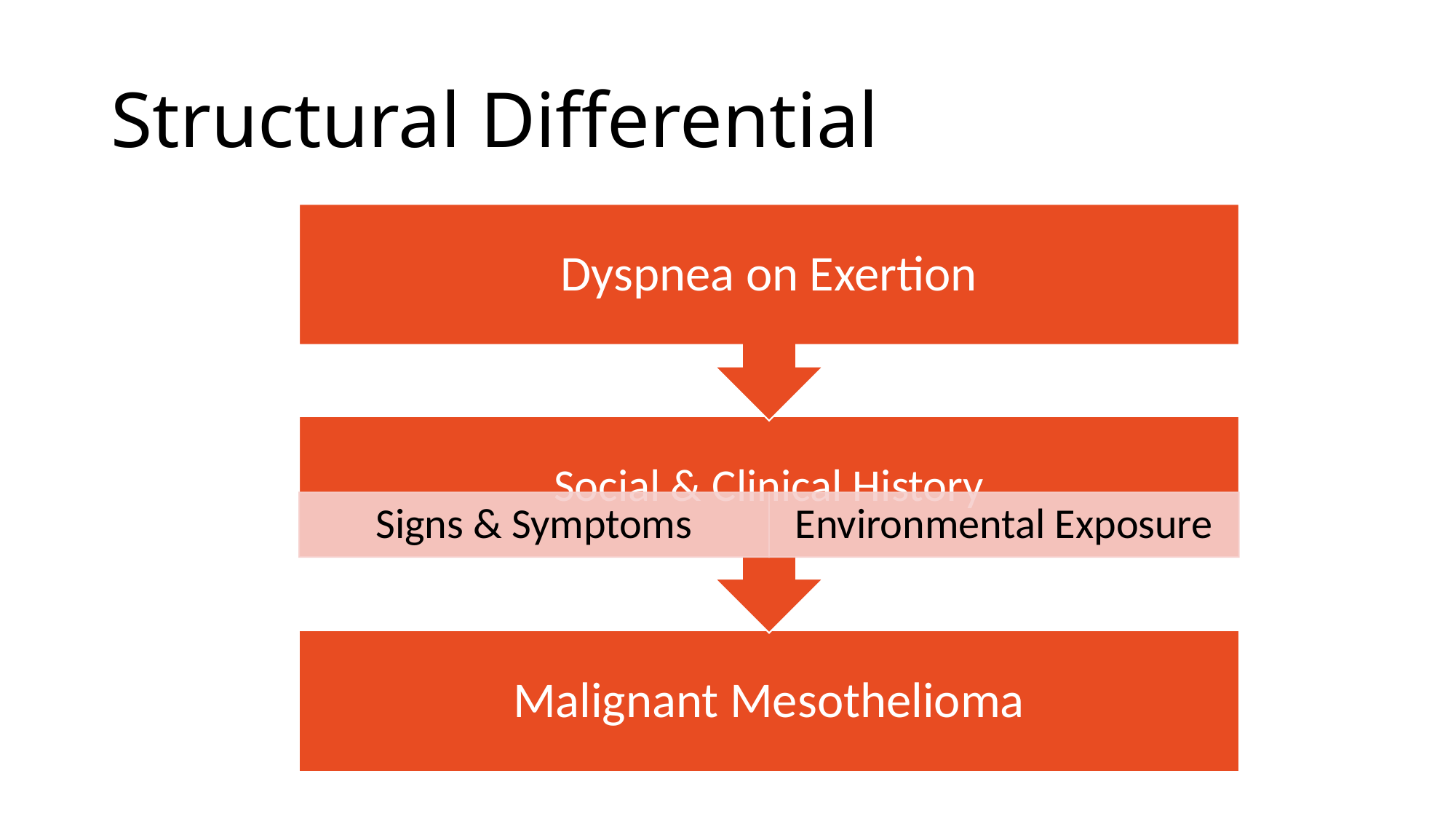

# Structural Differential

## Slide 34
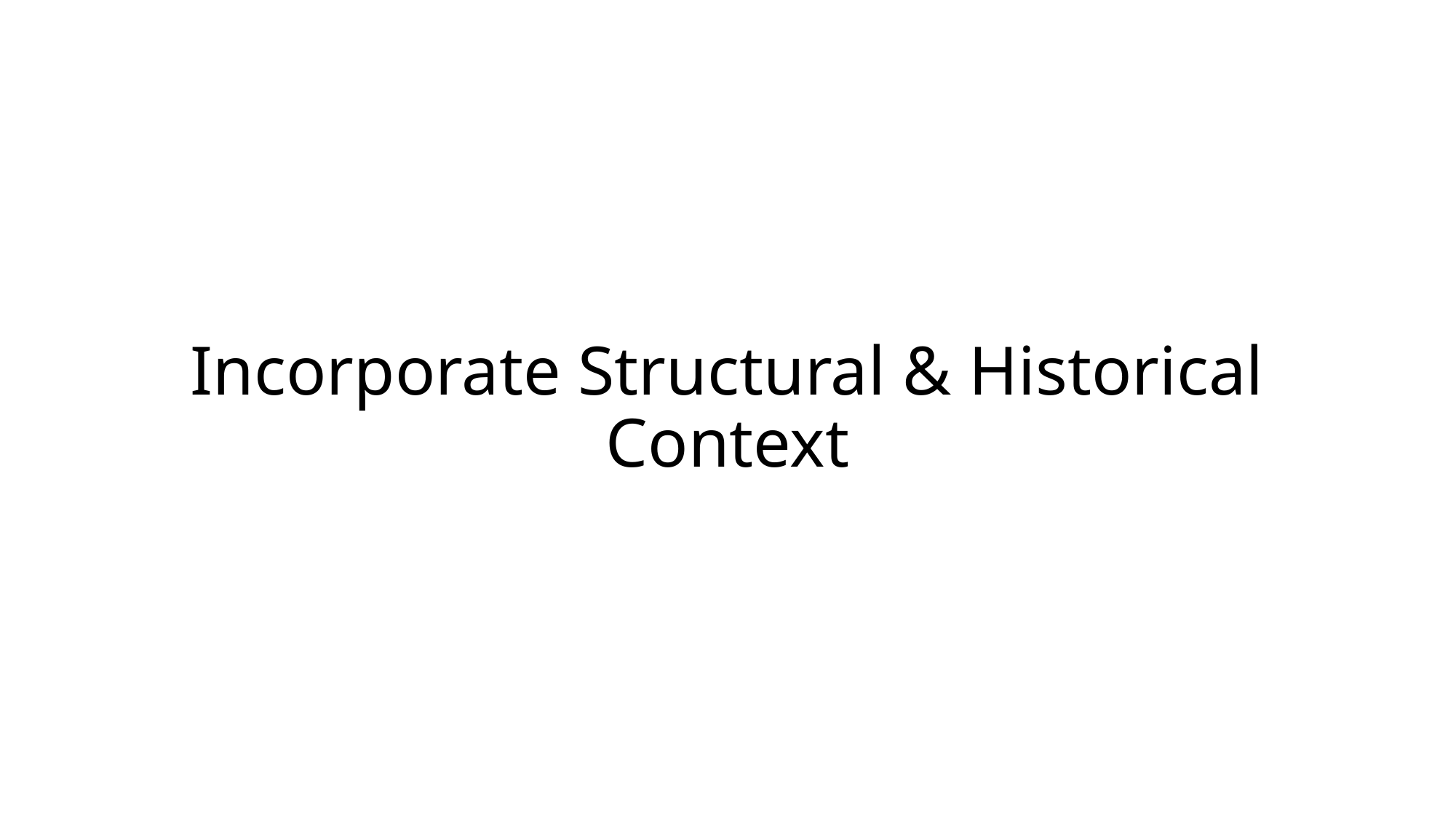

# Incorporate Structural & Historical Context

## Slide 35
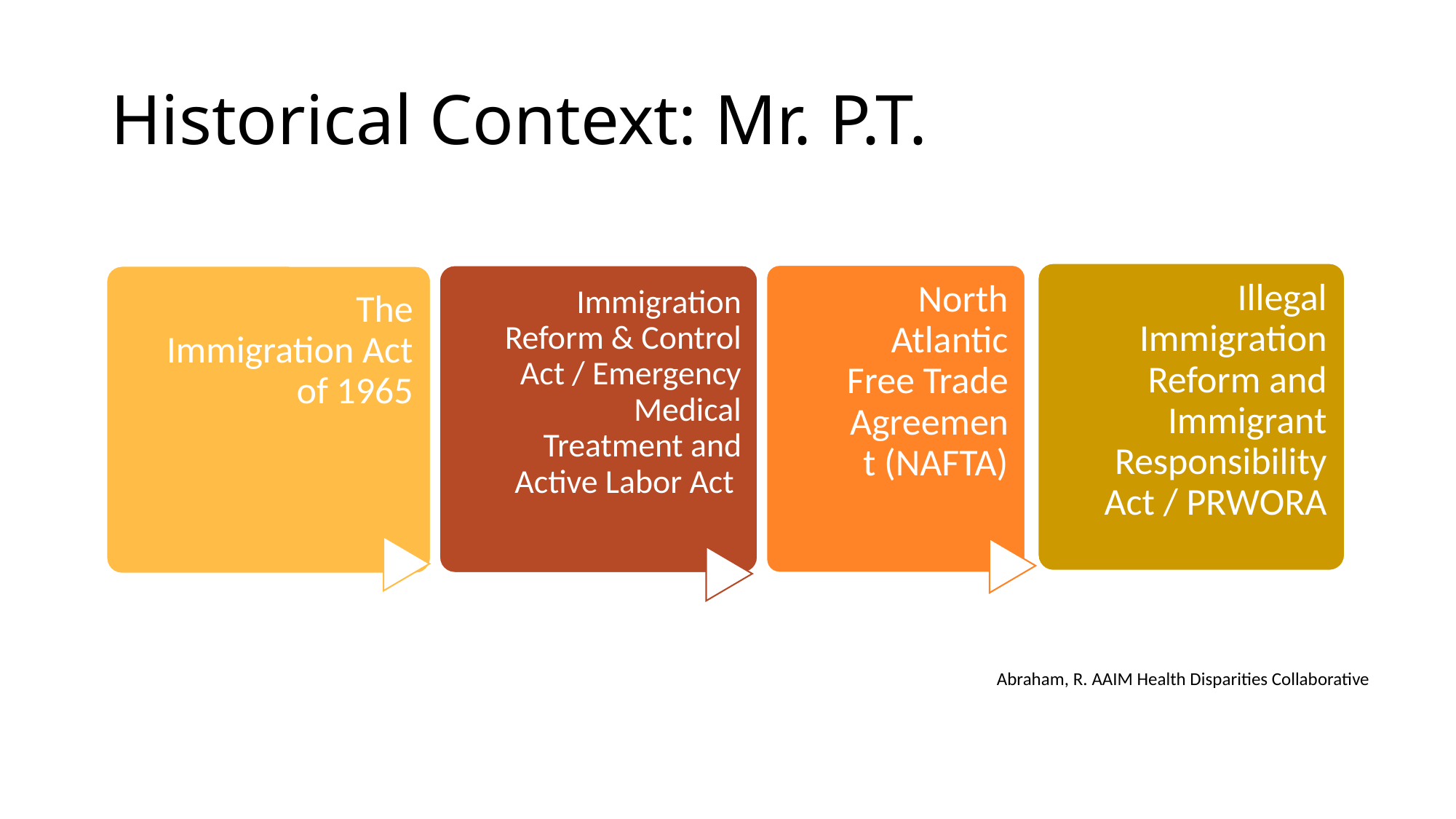

# Historical Context: Mr. P.T.
Abraham, R. AAIM Health Disparities Collaborative

## Slide 36
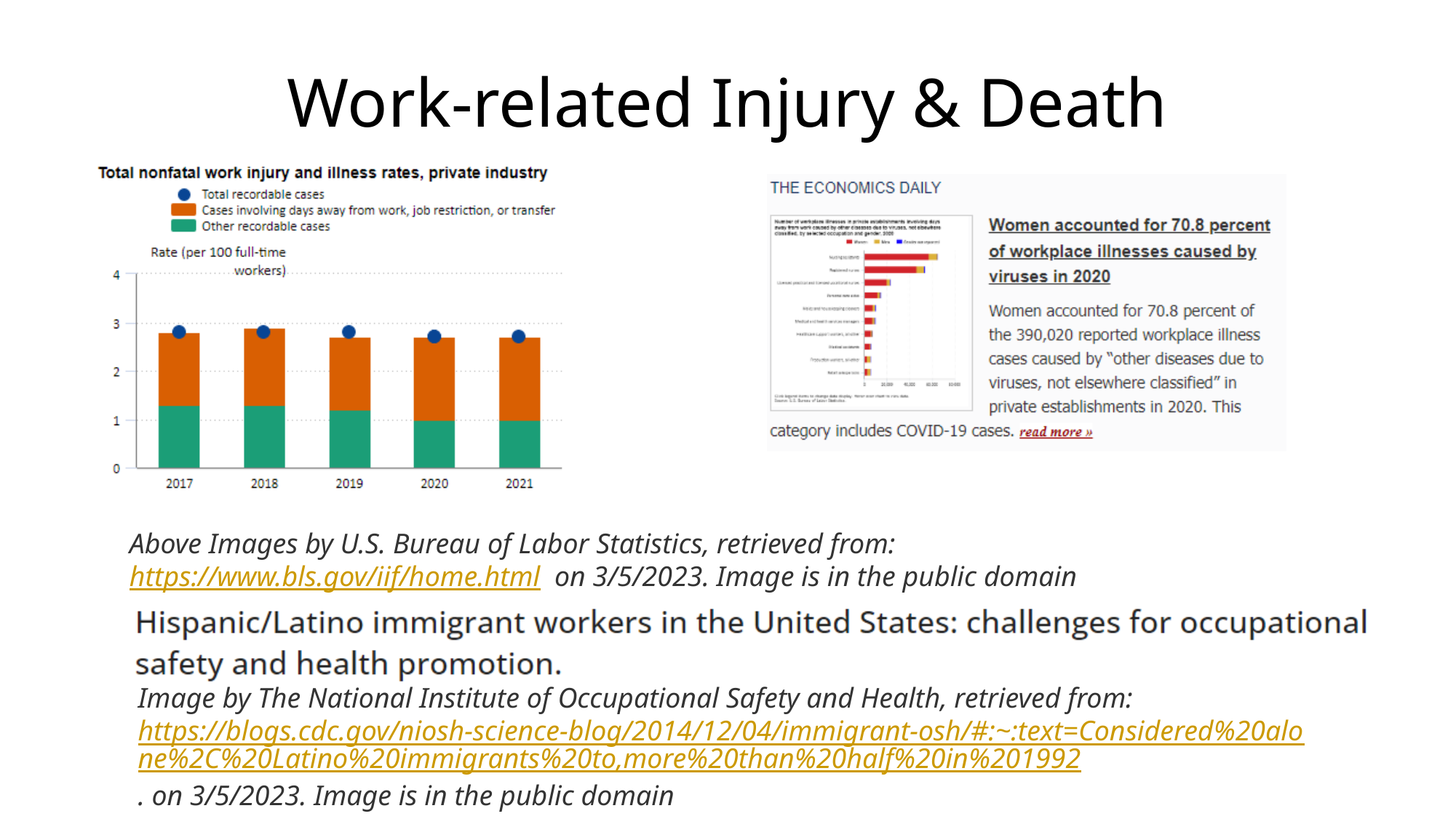

# Work-related Injury & Death
Above Images by U.S. Bureau of Labor Statistics, retrieved from: https://www.bls.gov/iif/home.html on 3/5/2023. Image is in the public domain
Image by The National Institute of Occupational Safety and Health, retrieved from: https://blogs.cdc.gov/niosh-science-blog/2014/12/04/immigrant-osh/#:~:text=Considered%20alone%2C%20Latino%20immigrants%20to,more%20than%20half%20in%201992. on 3/5/2023. Image is in the public domain

## Slide 37
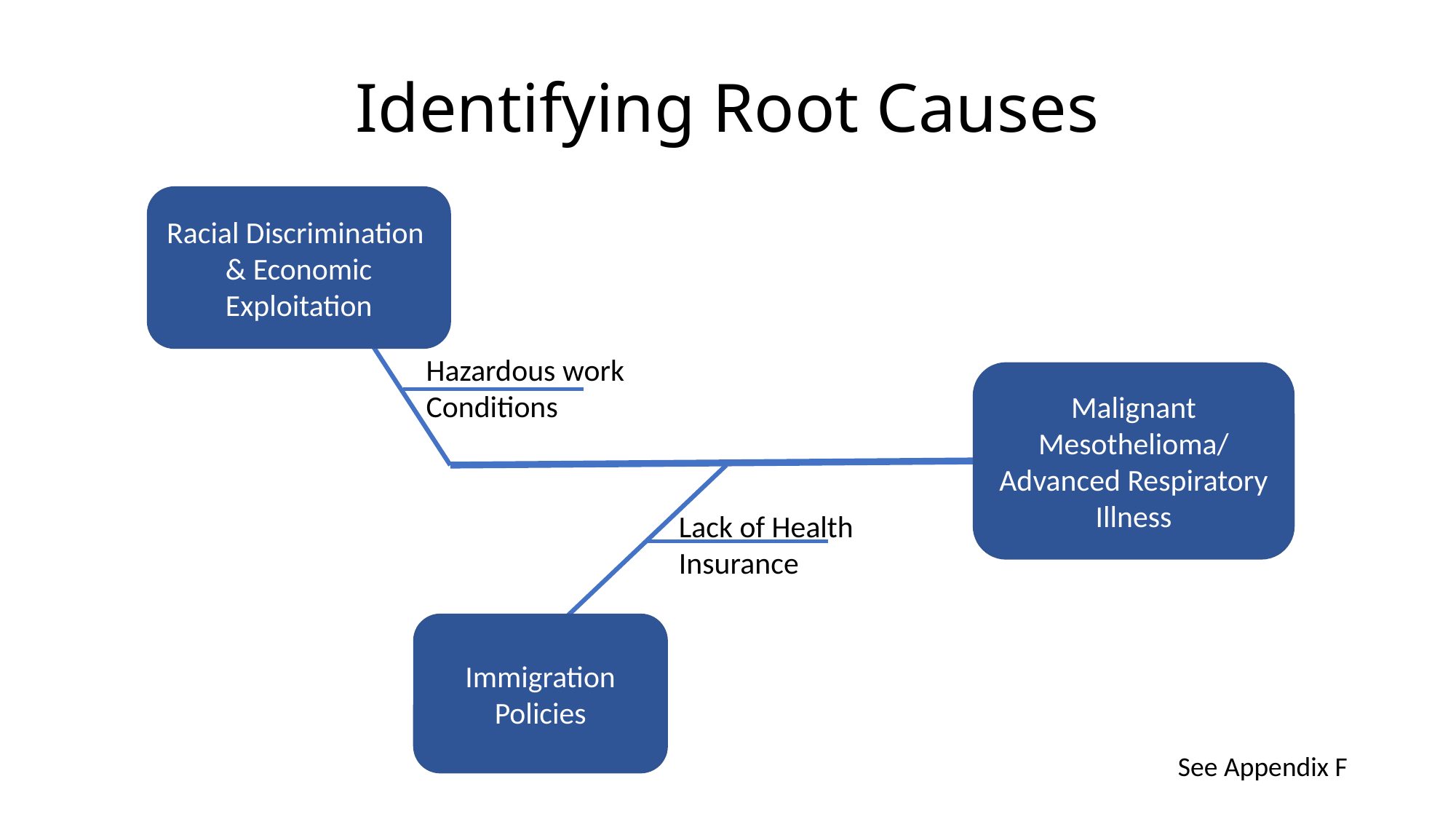

# Identifying Root Causes
Racial Discrimination & Economic Exploitation
Hazardous work
Conditions
Malignant Mesothelioma/
Advanced Respiratory Illness
Lack of Health Insurance
Immigration Policies
See Appendix F

## Slide 38
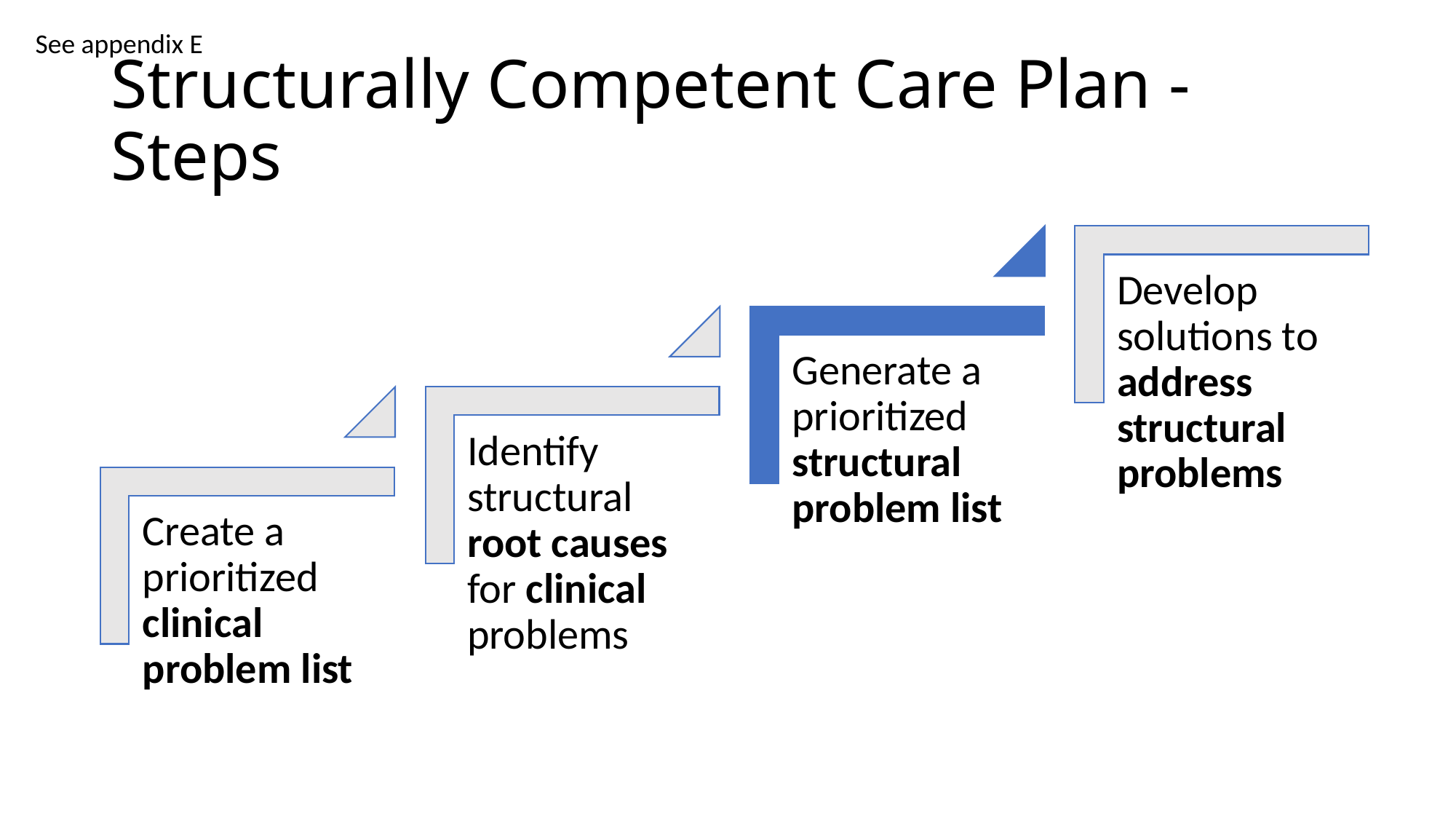

See appendix E
# Structurally Competent Care Plan - Steps

## Slide 39
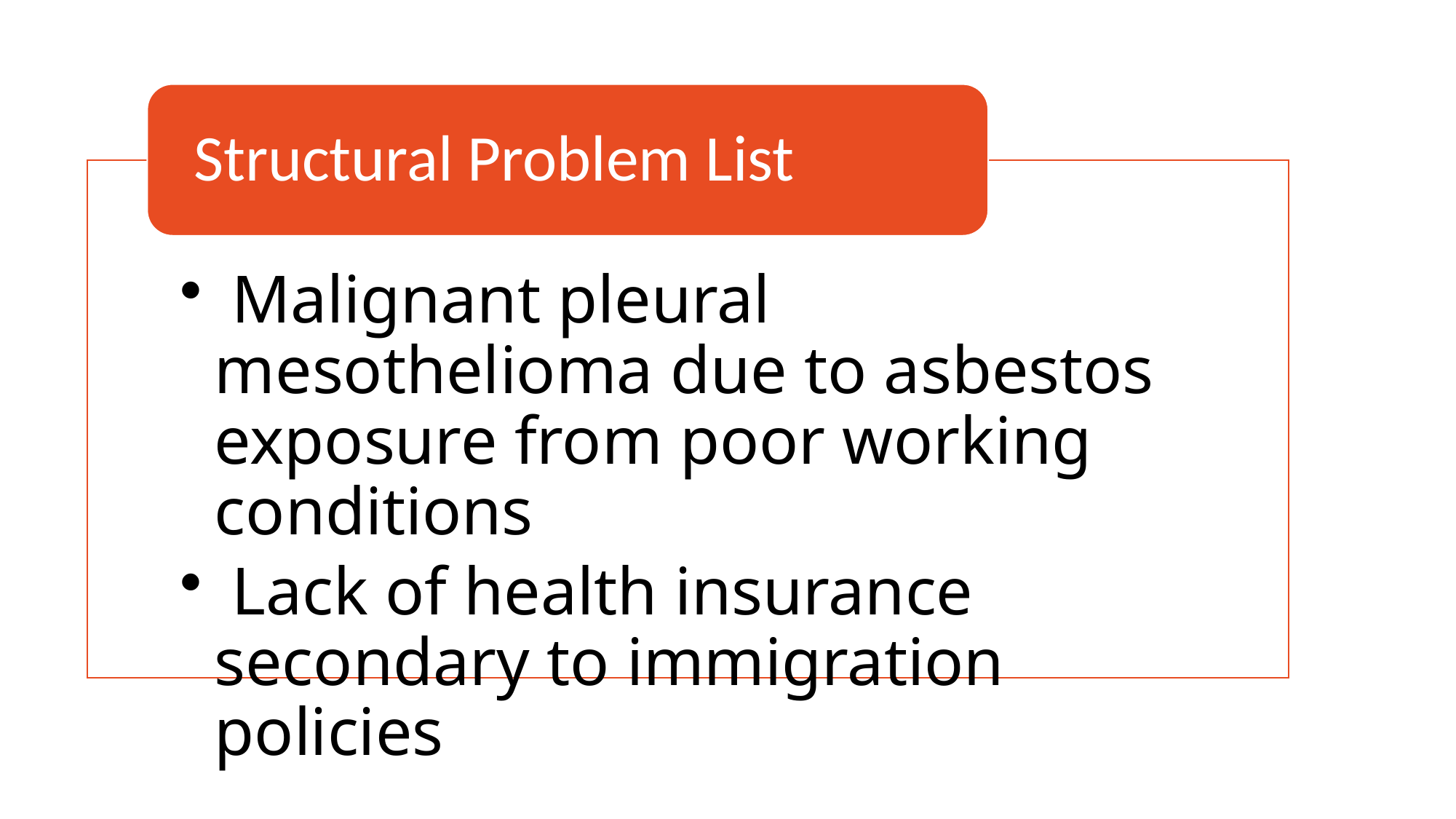

## Slide 40
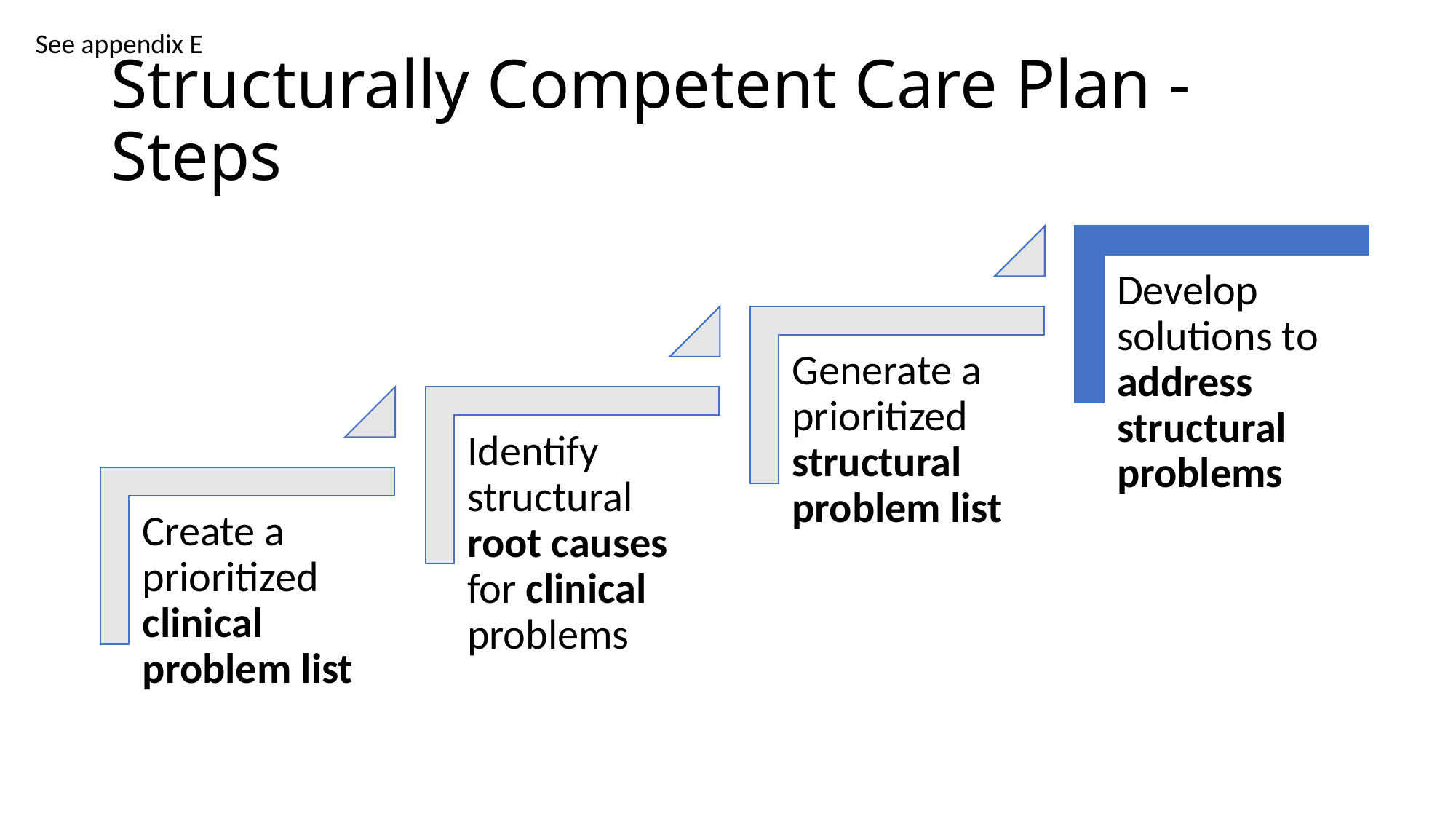

See appendix E
# Structurally Competent Care Plan - Steps

## Slide 41
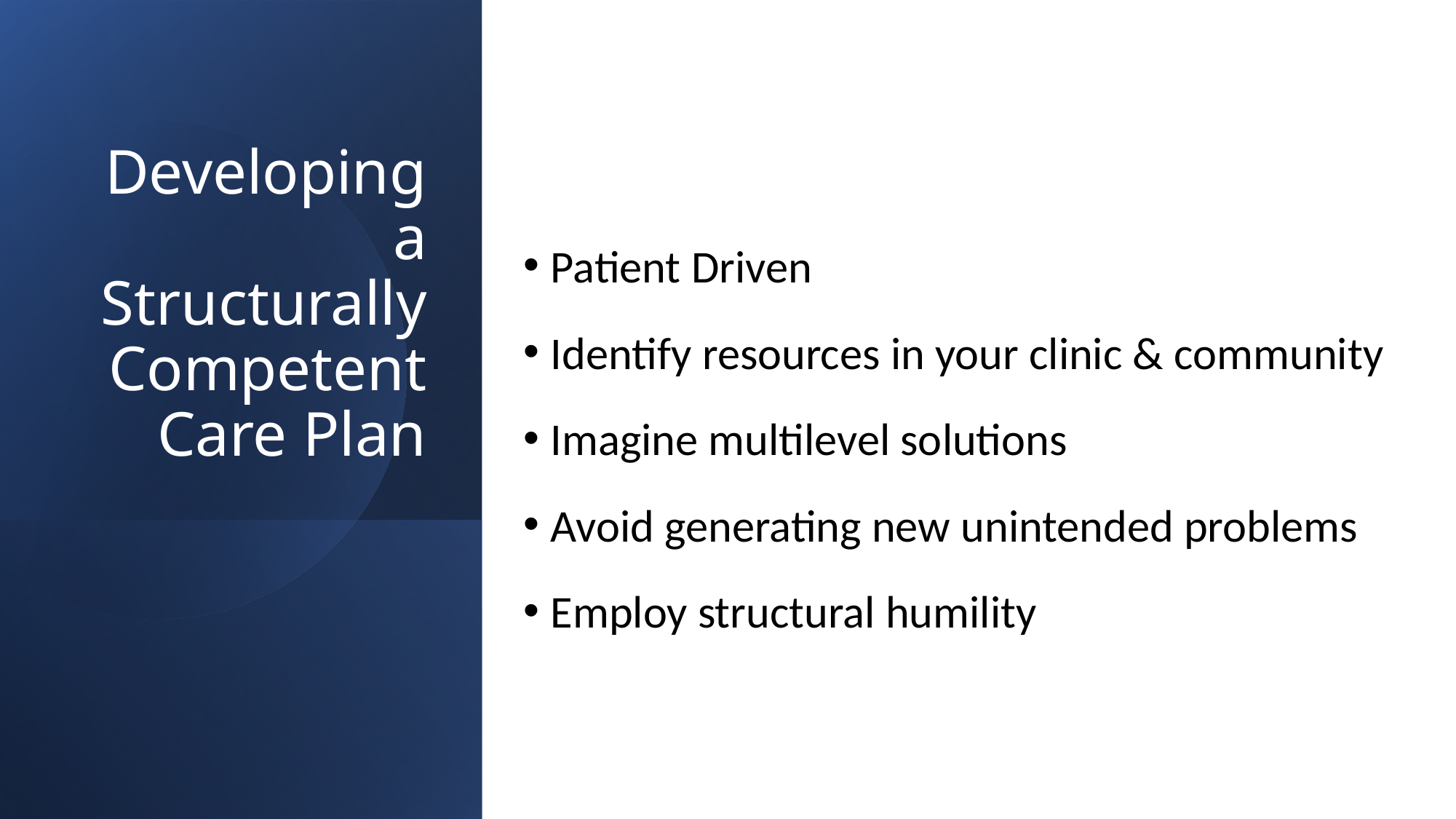

# Developing a Structurally Competent Care Plan
Patient Driven
Identify resources in your clinic & community
Imagine multilevel solutions
Avoid generating new unintended problems
Employ structural humility

## Slide 42
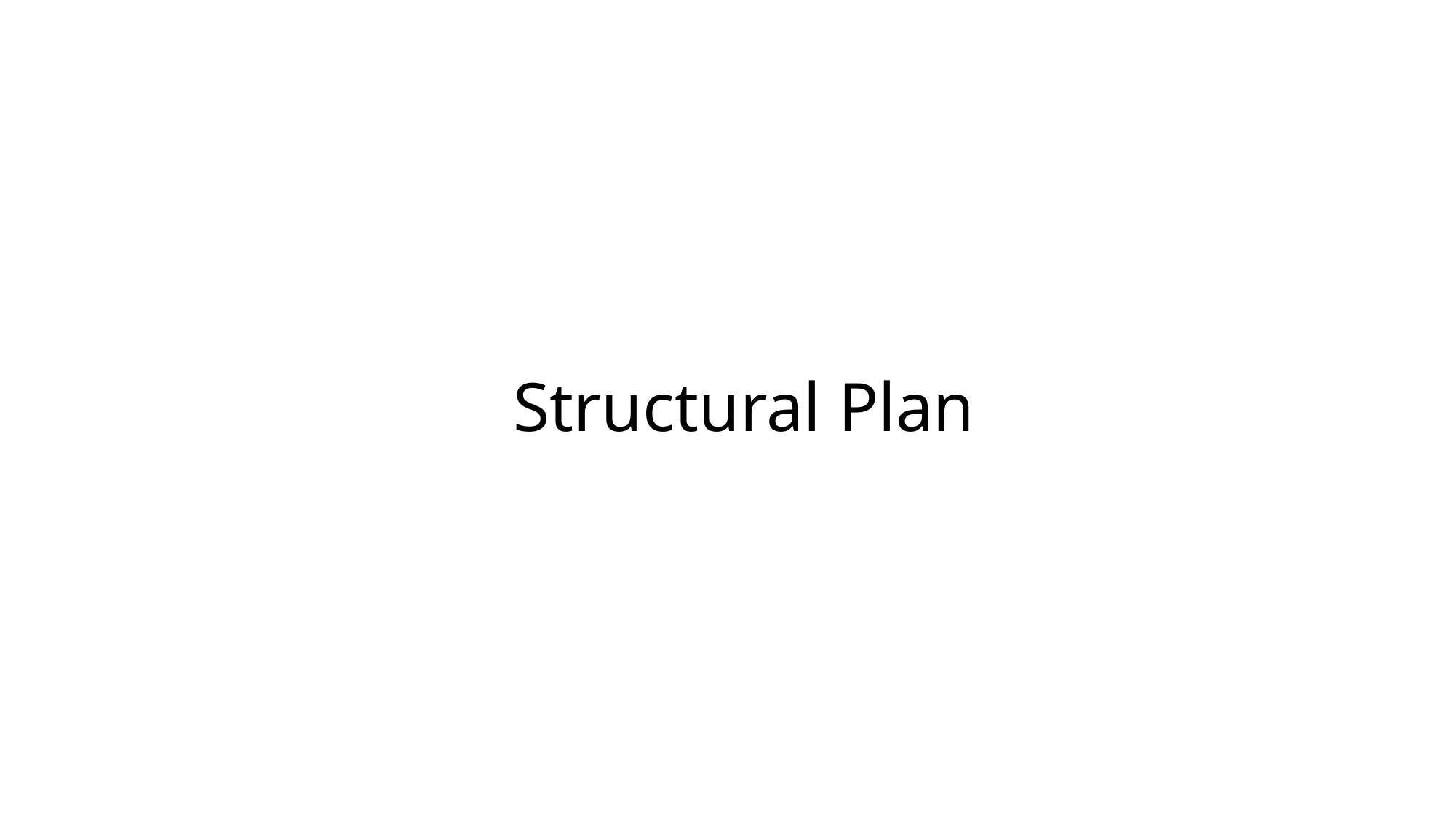

# Structural Plan

## Slide 43
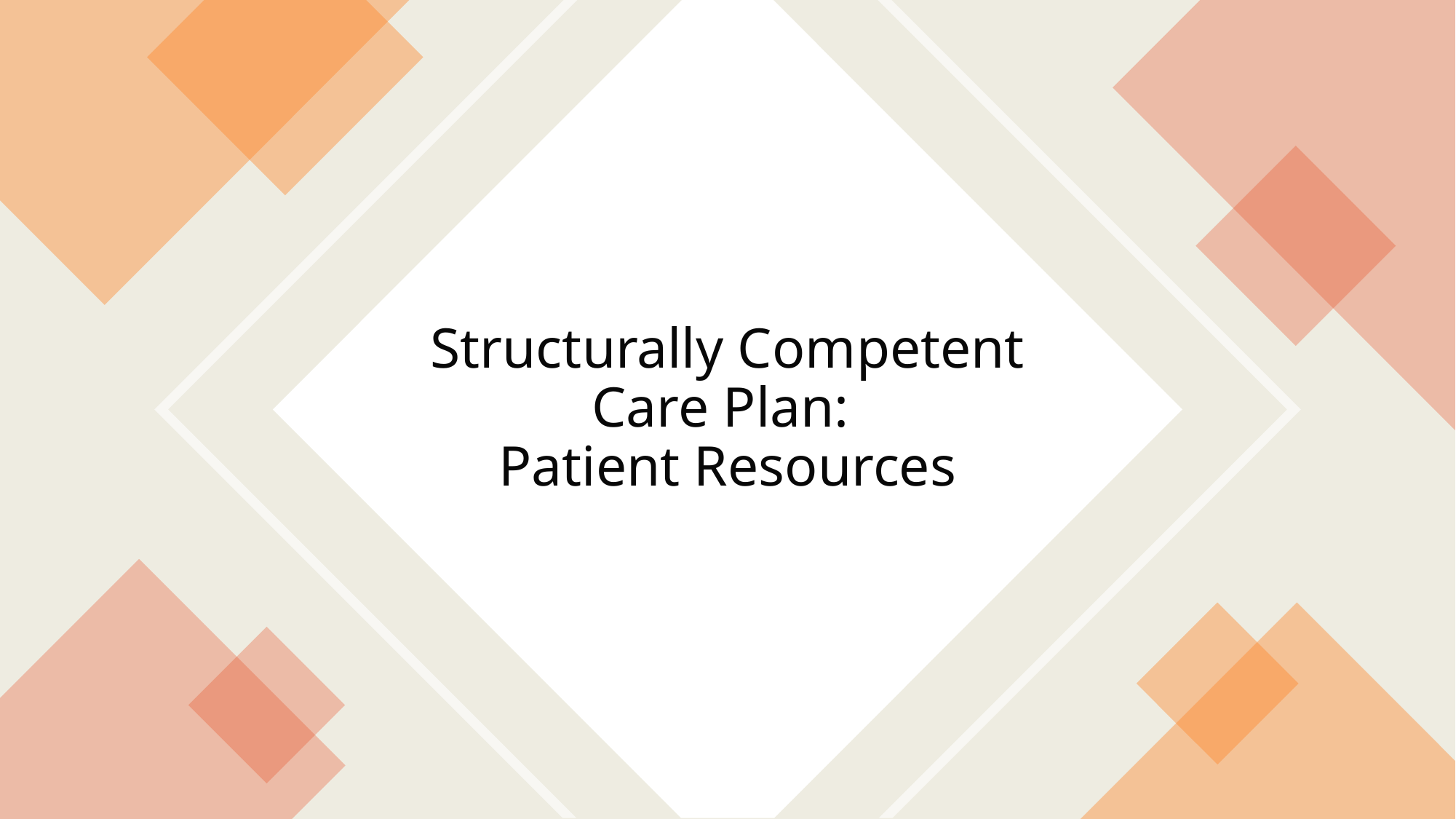

# Structurally Competent Care Plan: Patient Resources

## Slide 44
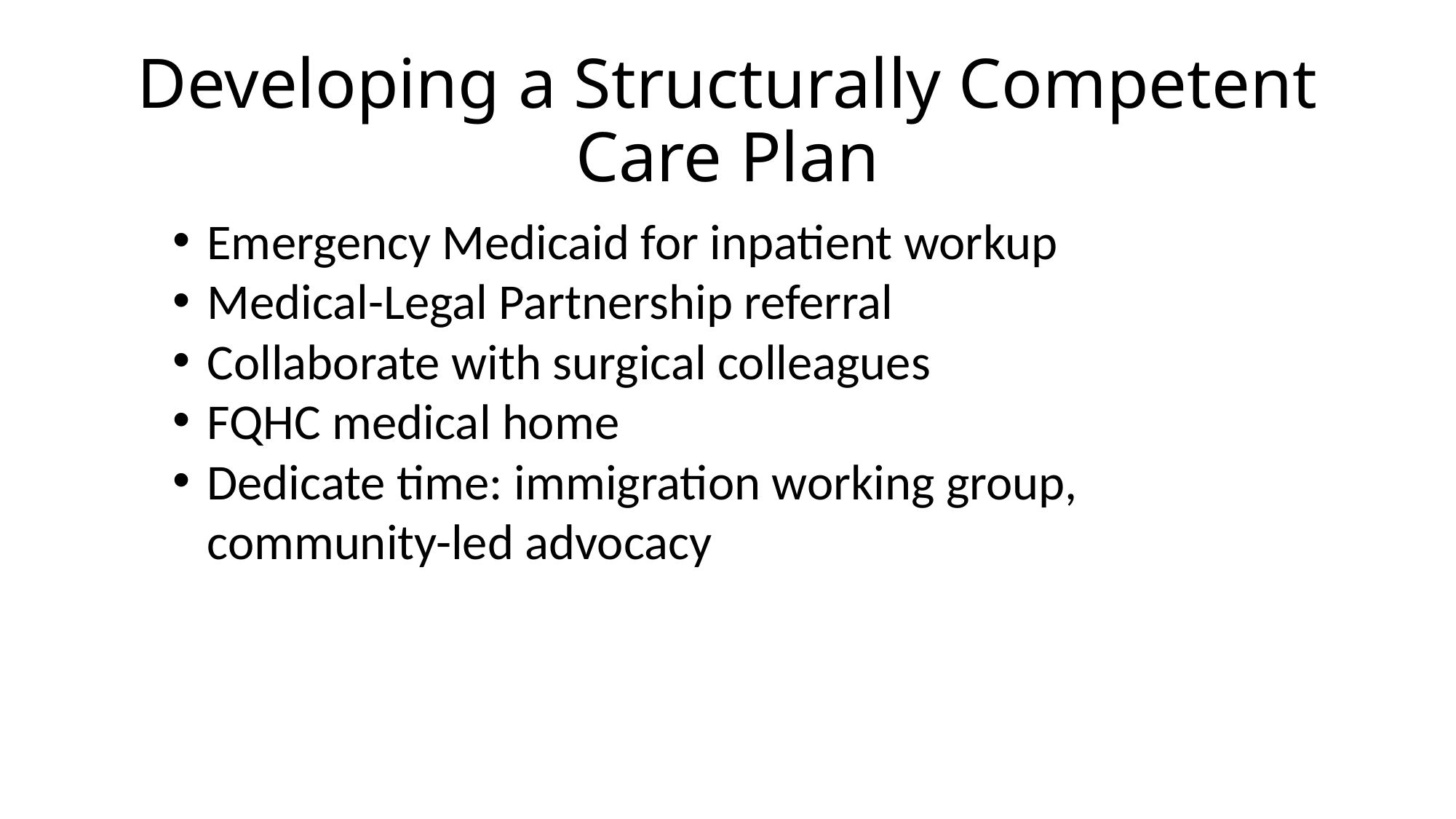

# Developing a Structurally Competent Care Plan
Emergency Medicaid for inpatient workup
Medical-Legal Partnership referral
Collaborate with surgical colleagues
FQHC medical home
Dedicate time: immigration working group, community-led advocacy

## Slide 45
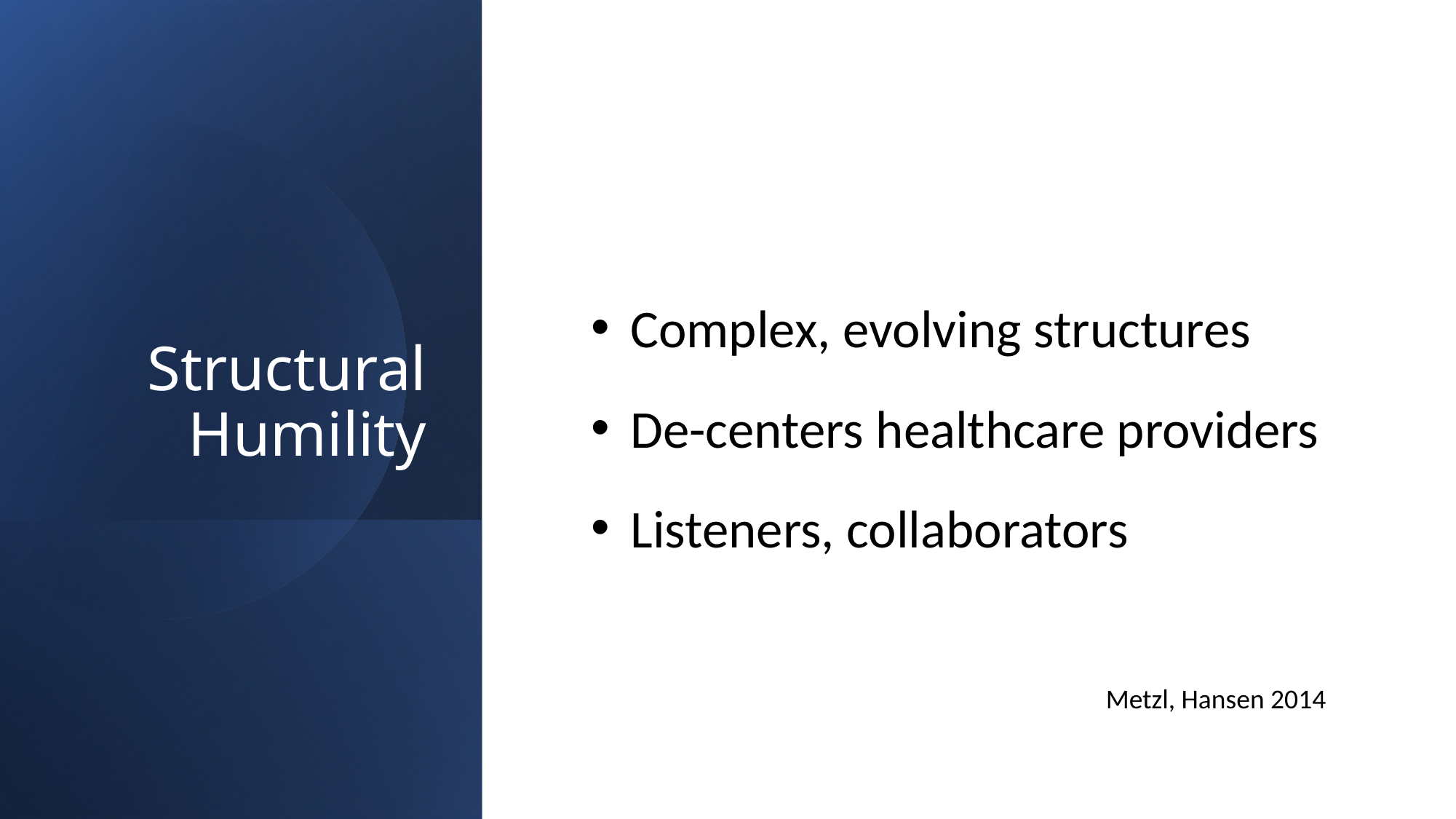

# Structural Humility
 Complex, evolving structures
 De-centers healthcare providers
 Listeners, collaborators
Metzl, Hansen 2014

## Slide 46
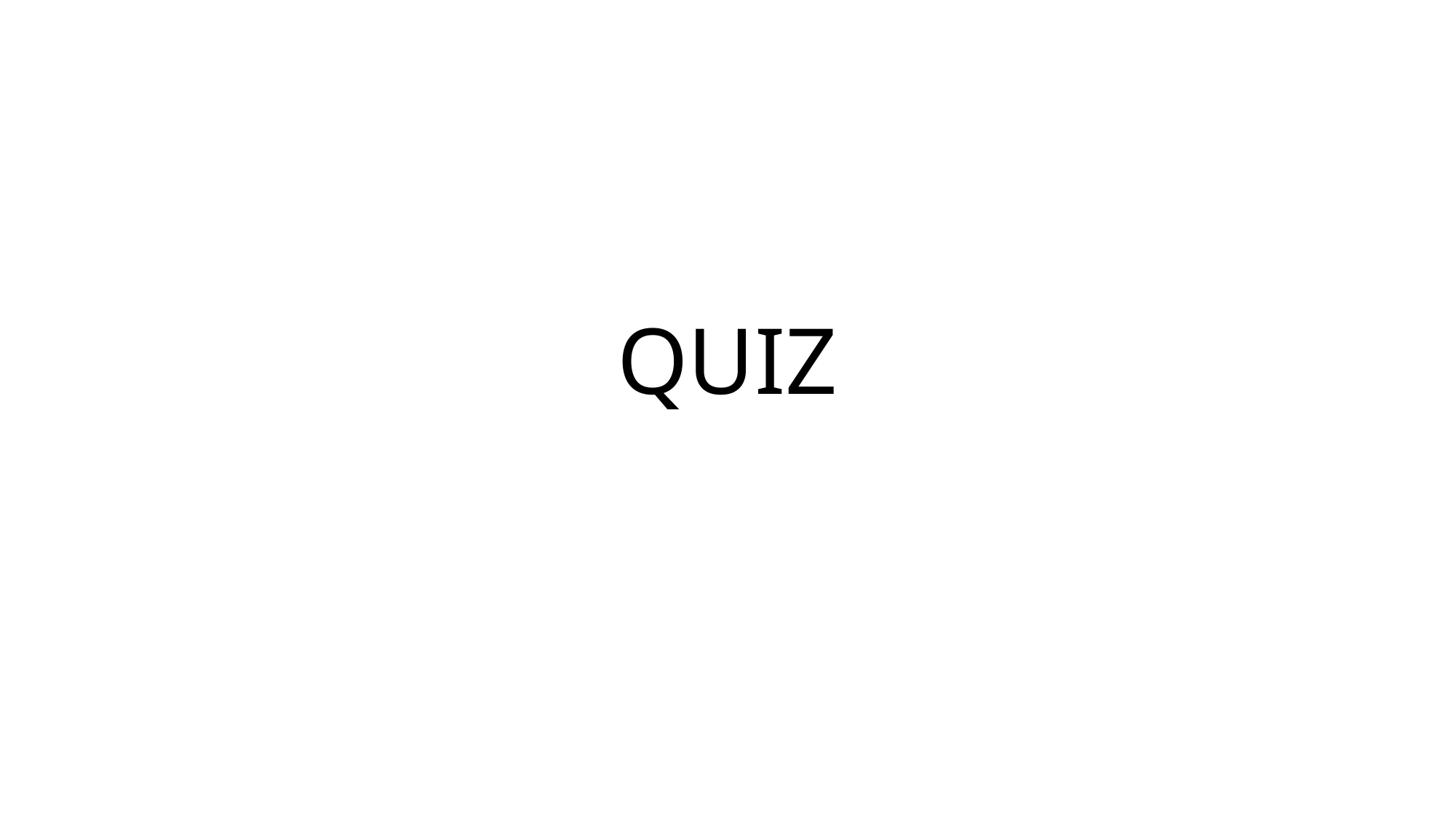

# QUIZ

## Slide 47
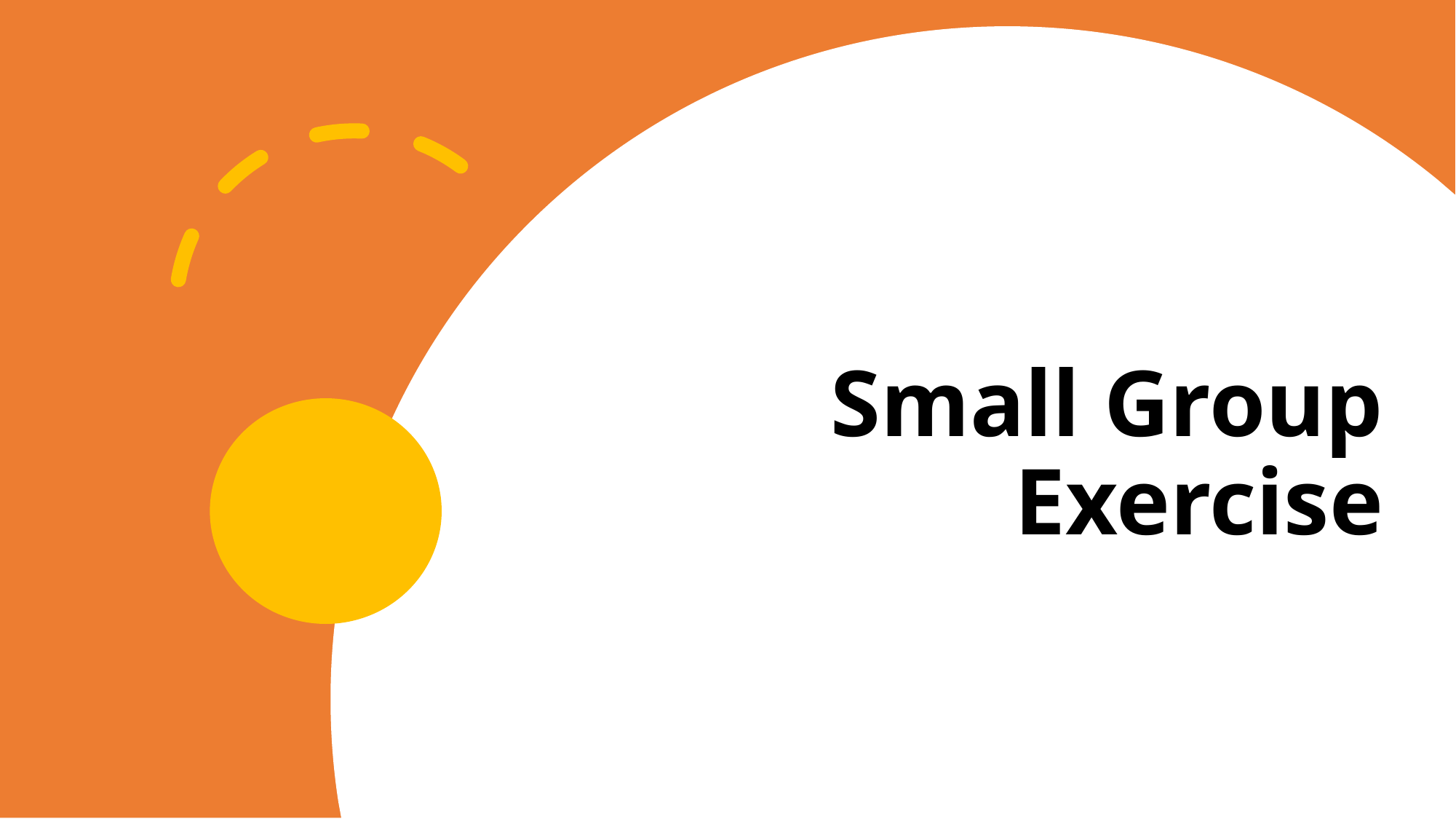

# Small Group Exercise

## Slide 48
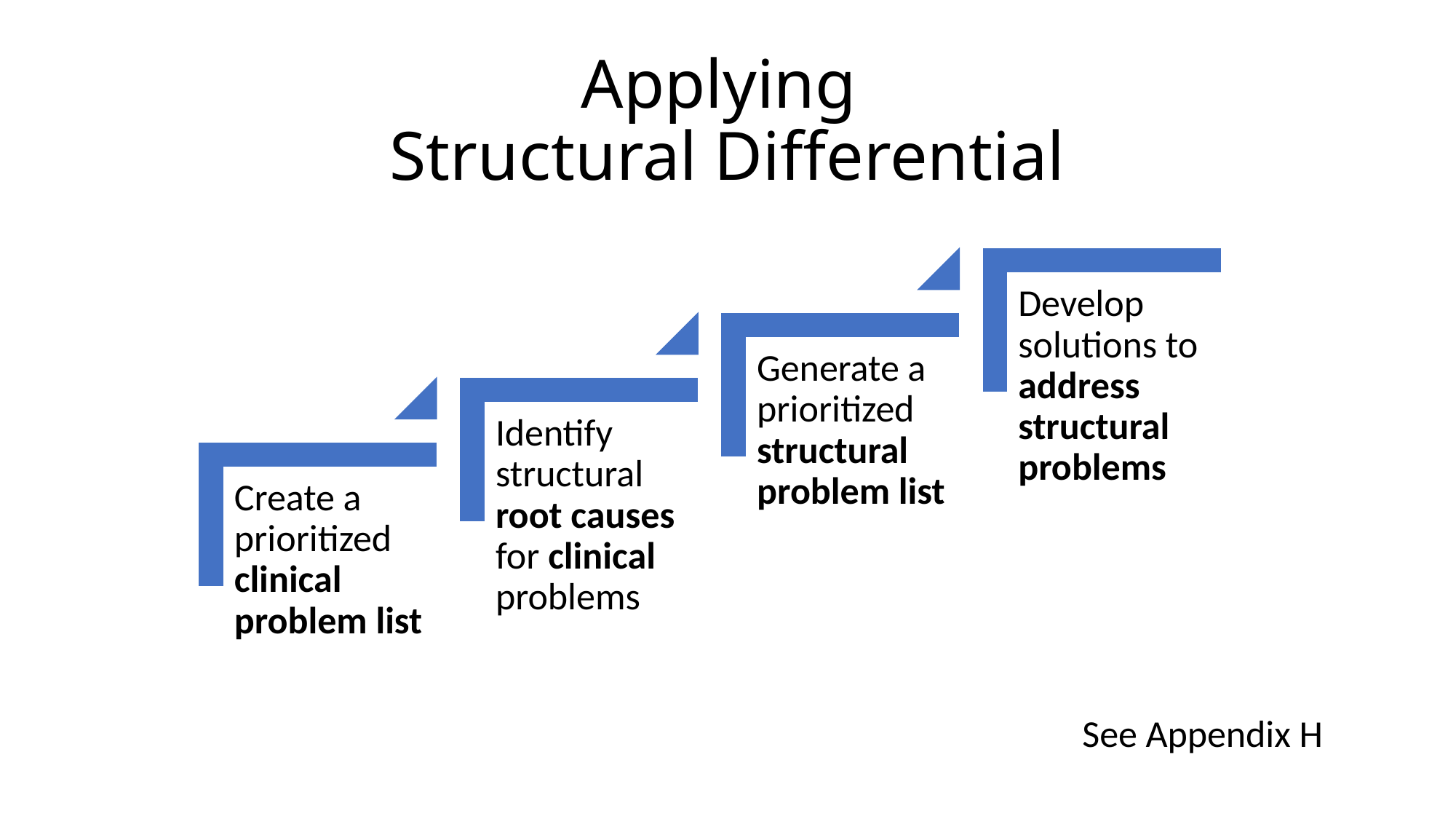

# Applying Structural Differential
See Appendix H

## Slide 49
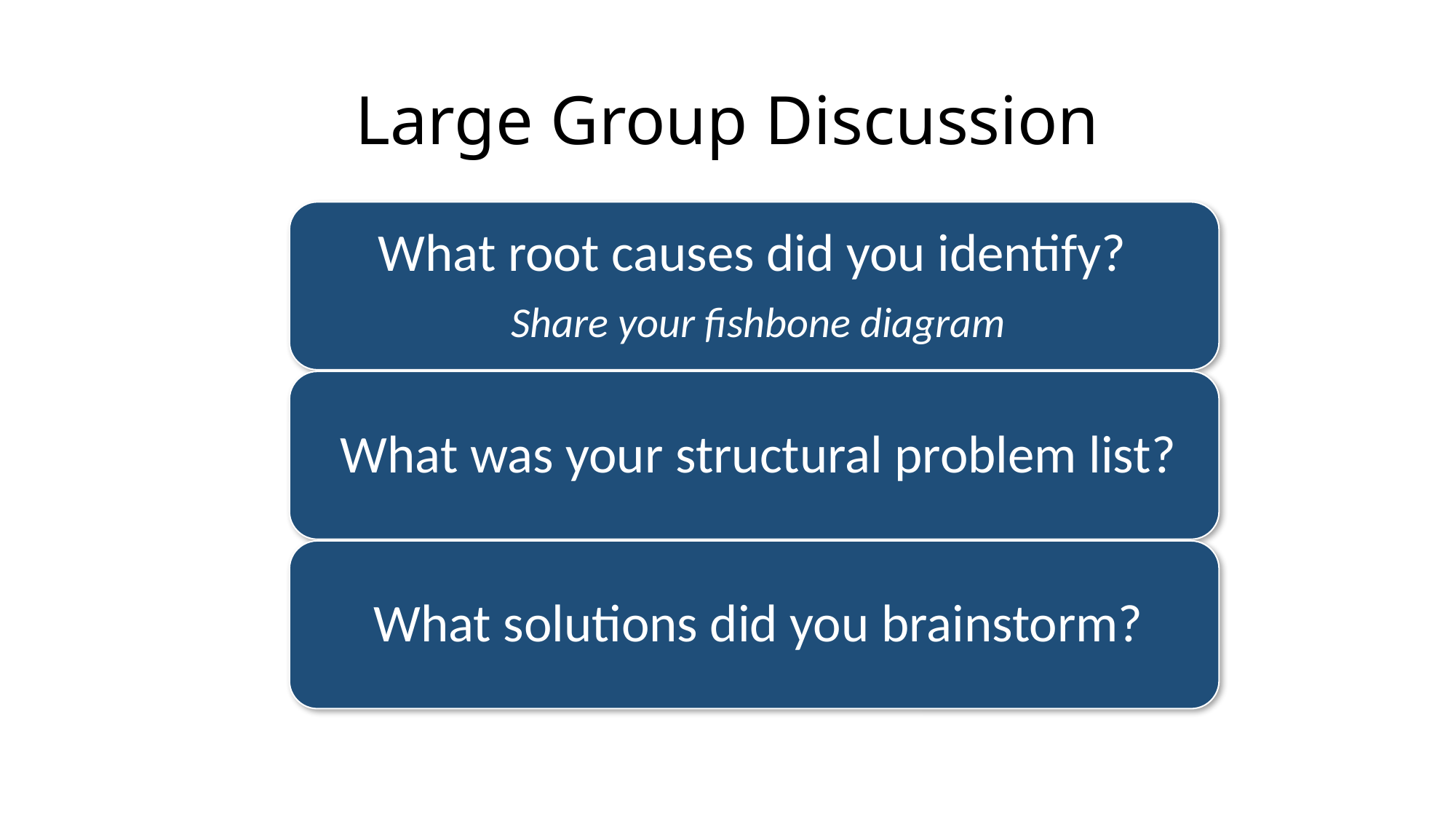

# Large Group Discussion

## Slide 50
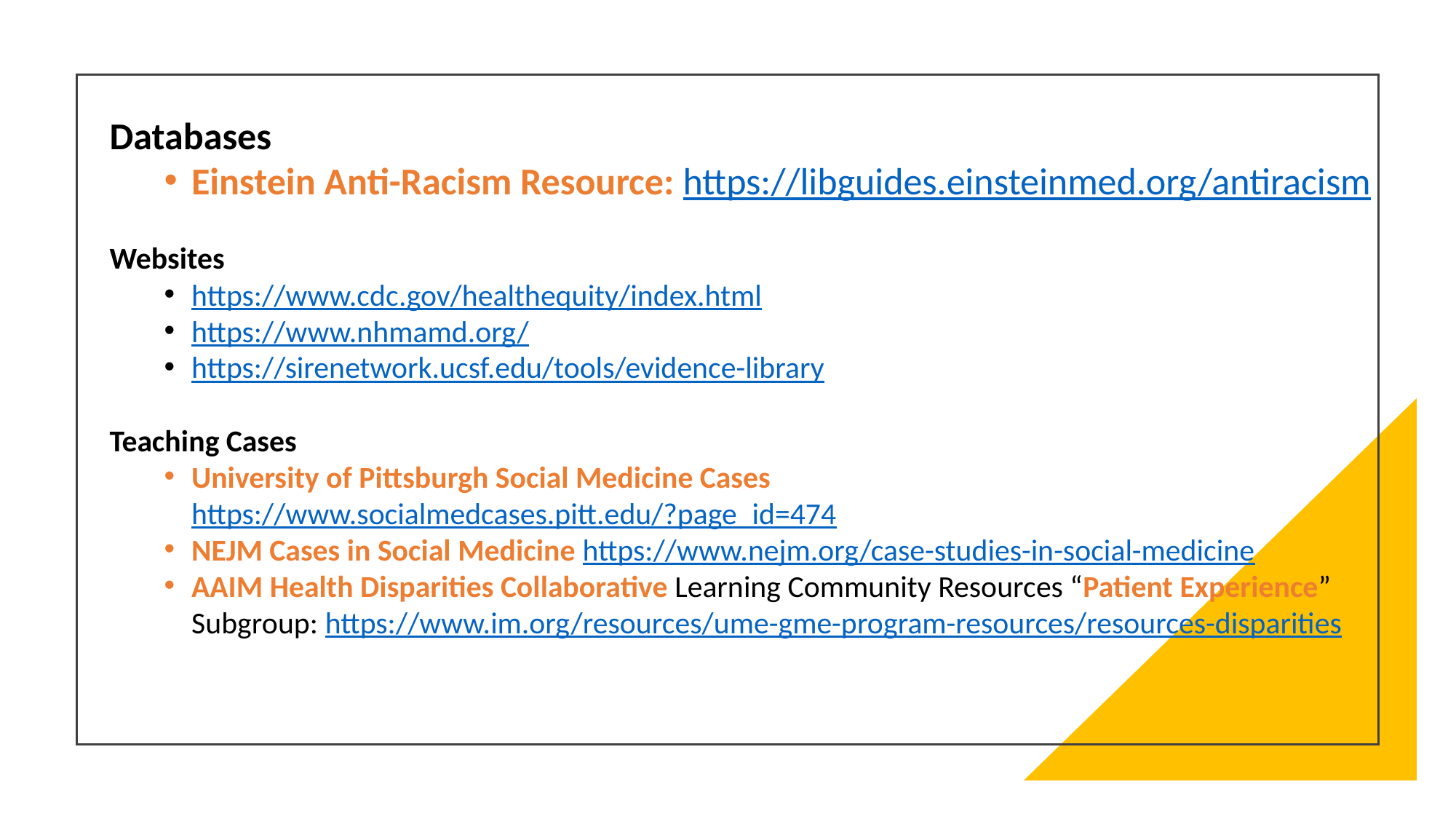

Databases
Einstein Anti-Racism Resource: https://libguides.einsteinmed.org/antiracism
Websites
https://www.cdc.gov/healthequity/index.html
https://www.nhmamd.org/
https://sirenetwork.ucsf.edu/tools/evidence-library
Teaching Cases
University of Pittsburgh Social Medicine Cases https://www.socialmedcases.pitt.edu/?page_id=474
NEJM Cases in Social Medicine https://www.nejm.org/case-studies-in-social-medicine
AAIM Health Disparities Collaborative Learning Community Resources “Patient Experience” Subgroup: https://www.im.org/resources/ume-gme-program-resources/resources-disparities

## Slide 51
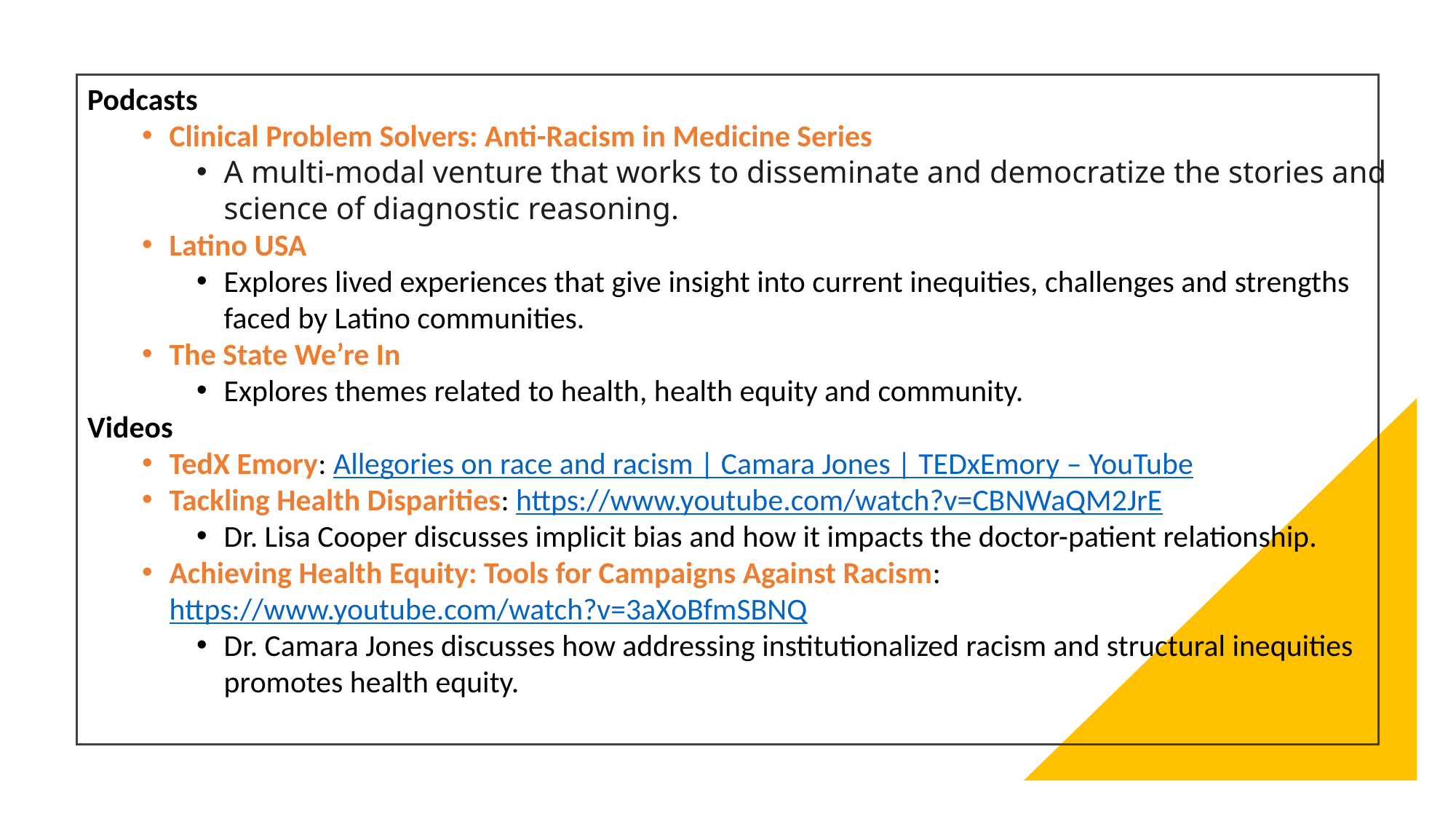

Podcasts
Clinical Problem Solvers: Anti-Racism in Medicine Series
A multi-modal venture that works to disseminate and democratize the stories and science of diagnostic reasoning.
Latino USA
Explores lived experiences that give insight into current inequities, challenges and strengths faced by Latino communities.
The State We’re In
Explores themes related to health, health equity and community.
Videos
TedX Emory: Allegories on race and racism | Camara Jones | TEDxEmory – YouTube
Tackling Health Disparities: https://www.youtube.com/watch?v=CBNWaQM2JrE
Dr. Lisa Cooper discusses implicit bias and how it impacts the doctor-patient relationship.
Achieving Health Equity: Tools for Campaigns Against Racism: https://www.youtube.com/watch?v=3aXoBfmSBNQ
Dr. Camara Jones discusses how addressing institutionalized racism and structural inequities promotes health equity.
