# Supplementary material for: Structural Competency: A Faculty Development Workshop Series for Anti-racism in Medical Education
Source: MedEdPORTAL. 2025 Feb 7;21:11492. doi: 10.15766/mep_2374-8265.11492 (PMC11802914; doi:10.15766/mep_2374-8265.11492)
Supplement: Supplementary file 1 — 1 - Introduction to SC.pptx1 - Facilitator Guide.docx1 - SC Rubric Handout.docx1 - Sample SC Learning Goals.docx2 - Resident Reports & Case-Based Presentations.pptx2 - Facilitator Guide.docx2 - Structural Differential Handout.docx2 - Small-Group Handout.docx3 - Demystifying SC.pptx3 - Facilitator Guide.docx3 - SC One-Minute Preceptor Handout.docx3 - SC SNAPPS Handout.docx3 - Role-Play Scenarios.docx4 - SC Hospital-Based Teaching.pptx4 - Facilitator Guide.docx4 - Daily Inpatient Checklist.docx4 - SC Discharge Checklist.docx4 - Small-Group Scenarios.docxPre- and Postsurveys.docx [file mep_2374-8265.11492-s001.zip › F. 2 - Facilitator Guide.docx]

**Appendix F WS 2- SLIDES AND TALKING POINTS**

| **Slide 1**  Welcome Slide Time Check: 00:00 | **Big Picture**: Set a welcoming learning environment. |
| --- | --- |
| 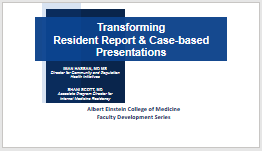 |  |
| *Suggested script/talking points:* Welcome the faculty participants and set a reassuring tone. | |

| **Slide 2**  Learning Objective Overview | **Big Picture:** Brief discussion on what will be discussed over the next ninety minutes |
| --- | --- |
| 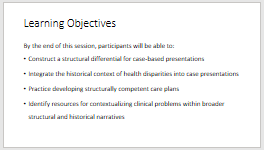 |  |
| *Suggested script/talking points:* Take them through each bullet point briefly. Emphasize that they are going to have specific opportunities to participate and that there will be time for questions at the end. | |

| **Slide 3**  Defining Race | **Big Picture:** Overview of key terms needed for participants to engage in discussions regarding structural competency |
| --- | --- |
| 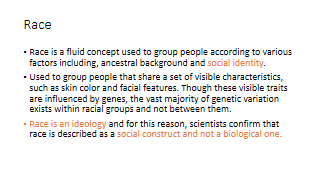 |  |
| *Suggested script/talking points:*  Our anecdotal understanding of race is not accurate.  Society has normalized using race as a means of grouping people based upon visual characteristics such as skin melanin, hair texture or facial features. The National Institute on Genomics and Academy of Science states race is accurately described as a social construct and a not biologic one.  Genetic ties to race are a debunked theory that has historic roots in the racist practice of eugenics | |

| **Slide 4**  Defining Racism | **Big Picture**: Overview of key terms needed for participants to engage in discussions regarding structural competency |
| --- | --- |
| 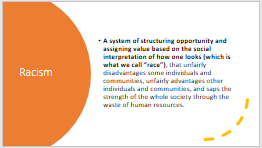 |  |
| *Suggested script/talking points:*  *Definition coined by Dr. Camara P. Jones - Past president of the American Public Health Association, PCSM Alumna Einstein Montefiore Family Medicine*  Racism is a system, not an individual character flaw or a personal moral failing.  It is a system of power that structures opportunity (education, housing, jobs, justice) and assigns value (worthy or unworthy, full of potential or full of menace) based on so-called “race”, the social interpretation of how we look (set of visual characteristics).  This system routinely disadvantages groups/class of people and reciprocally advantages other groups class of people. | |
| **Slide 5**  Defining Racialized Institutions | **Big Picture:** Explain how institutions are constituted of people and permeated with attitudes and beliefs including those rooted in racism. |
| 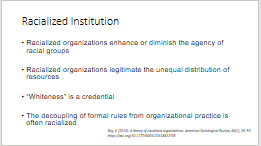 |  |
| *Suggested script/talking points:*  Institutions are constituted of people and thus are permeated with their attitudes and beliefs including those that are rooted in racism. This in turn becomes reflected in the policies, culture and practices creating racialized institutions.  The bullets detail how racism manifests – as which groups can exert agency, reliably have access to resources (financial, visibility, project opportunity, collective empathy, information etc.) | |
| **Slide 6**  Medicine exemplifies structural racism | **Big Picture:** Increase awareness of how healthcare and associated institutions of higher medical education are interwoven with structural racism |
| 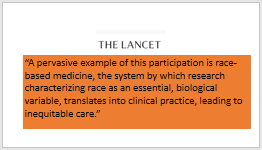 |  |
| *Suggested script/talking points:*  Although clinicians often imagine themselves as beneficent caregivers, it is increasingly clear that medicine is not a stand-alone institution immune to racial inequities, but rather is an institution of structural racism. A pervasive example of this participation is race-based medicine, the system by which research characterizing race as an essential, biological variable, translates into clinical practice, leading to inequitable care.  *When viewing the presentation in presenter mode, please note that the Lancet picture displayed on this slide corresponds to the article referenced in the slide’s content. This visual cue is intended to help you quickly identify the source of the information and provide additional context during your presentation.* | |
|  |  |
|  |  |
|  | |
| **Slide 7**  Influence of Structural and Social Determinants of health (SSDoH) on Health Outcomes | **Big Picture:** SSDoH impact health outcomes. *Repeated slides across workshop presentations are intentionally* included as a review to solidify key concepts, ensuring that participants reinforce their understanding and retention of the most critical information. |
| 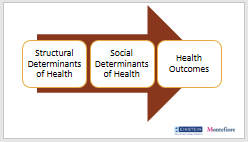 |  |
| *Suggested script/talking points:*  Structural Determinants of Health and the “social and political mechanism[s] that generate, configure and maintain social hierarchies”, for example, racism, labor markets and political institutions. These in turn impact the conditions in which people are *born, grow, work, live, and age,* also known as the Social Determinants of Health. Examples include economic stability, neighborhood and physical environment, education, food, community and social context, and health care system. Social determinants of health contribute to health outcomes and to the health disparities we see across social hierarchies, ex. across races/ethnicities. | |
| **Slide 8**  Evolution to Structural Competency | **Big Picture**: Brief discussion on the conceptual development of structural competency. *Repeated slides across workshop presentations are intentionally* included as a review to solidify key concepts, ensuring that participants reinforce their understanding and retention of the most critical information. |
| 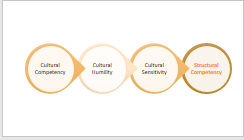 |  |
| - *Suggested script/talking points:* Understanding the patient's life context is crucial for building a robust therapeutic relationship and devising effective health management plans. Historically, terms like "cultural competence" have been used to emphasize the importance of integrating a patient's culture into clinical reasoning. However, these terms have evolved over time because they can inadvertently perpetuate bias and harmful stereotypes, potentially pathologizing cultural norms. This evolution reflects a growing awareness of the limitations and potential pitfalls of focusing solely on cultural factors. - Progressing toward structural competency represents an important shift. It empowers health professionals to consider the broader social, economic, and political structures that impact patient health. By understanding these factors, healthcare providers can create more accurate health recommendations and set achievable health outcome goals that align with the patient's unique life context. This approach moves beyond individual cultural considerations, aiming to address systemic issues and promote equity in healthcare. | |
| **Slide 9**  Definition of Structural Competency | **Big Picture:** Introduce the definition of Structural Competency*. Repeated slides across workshop presentations are intentionally* included as a review to solidify key concepts, ensuring that participants reinforce their understanding and retention of the most critical information. |
| 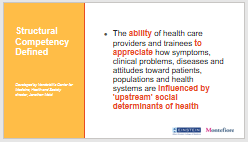 |  |
| *Suggested script/talking points:* Structural competency refers to the trained ability to recognize and respond to health and illness as the downstream effects of broad social, economic, and political structures. Unlike cultural competency, which focuses on individual beliefs and behaviors, structural competency emphasizes understanding how systemic factors—such as policies, institutional practices, and social norms—shape health outcomes. This concept encourages healthcare professionals to consider these larger structural influences when diagnosing, treating, and preventing illness, ultimately aiming to address and reduce health disparities. | |
| **Slide 10**  Definition of Structural Competency | **Big Picture**: Rearticulation of structural competency*. Repeated slides across workshop presentations are intentionally* included as a review to solidify key concepts, ensuring that participants reinforce their understanding and retention of the most critical information. |
| 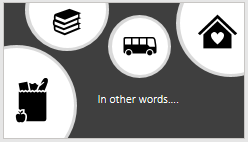 |  |
| *Suggested script/talking points:*  In other words structural competency is the ability of a healthcare professional to understand that a patient’s access to quality housing, education, food, jobs, and transportation are upstream influences on the clinical manifestation of disease within a person and the relationship between the patient, provider, and the health system they reside within. | |
| **Slide 11**  Structural Competency Pillars | **Big Picture:** Brief discussion on structural competency pillars. *Repeated slides across workshop presentations* are intentionally included as a review to solidify key concepts, ensuring that participants reinforce their understanding and retention of the most critical information. |
| 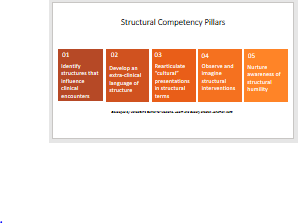 |  |
| *Suggested script/talking points:*  When teaching through the framework of structural competency health professionals should evaluate their learners base on these five pillars  Pillar 1: Name structures that contribute to health outcomes, education, housing ect  Pillar 2: Understand structure via sociology, urban planning, and economics.  Pillar 3: Recasting case presentations to acknowledge these structural barriers to health  Pillar 4: Develop interventions to address health infrastructures  Pillar 5: Nurture a critical awareness of structural humility | |
|  |  |
|  |  |
| **Slide 12**  Importance of Structural Competency | **Big Picture**: Structural competency is a vital skill for providers. *Repeated slides across workshop presentations are intentionally* included as a review to solidify key concepts, ensuring that participants reinforce their understanding and retention of the most critical information. |
| 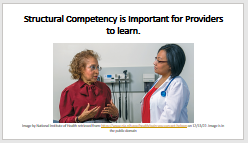 |  |
| *Suggested script/talking points:*  Structural competency is a critical skill to provide effective health services, cultivate a sense of fulfillment in health care professionals, and advocate to create structural change to support healthier patient communities. | |
|  | |
| **Slide 13**  Quiz | **Big Picture**: Check understanding of structural competency |
| 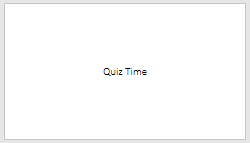 |  |
| *Suggested script/talking points:*  The following quiz was administered via Zoom Poll:  Name “upstream” contributors to health outcomes (check all that apply)   1. **Neighborhood Redlining** 2. Alcohol consumption 3. **Lack of green spaces** 4. Cigarette Smoking 5. Member of minority race/ethnicity | |
| **Slide 14**  Health Disparities in Context | **Big Picture**: To build provider awareness of structural contributors to health outcomes, frame health disparities as the result of historic neglect of underserved communities |
| 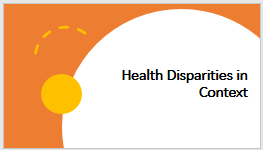 |  |
| *Suggested script/talking points:*  Including a historical context in medical education is crucial to addressing health inequities. The disparities we observe in chronic disease morbidity and mortality have deep roots dating back to the establishment of the medical profession. Healthcare professionals must become aware of this aspect of medical history that currently hinders their ability to meet the needs of marginalized communities. | |
| **Slide 15**  Example of Historic Context of Health Disparities | **Big Picture**: Example of historic roots of health disparities |
| 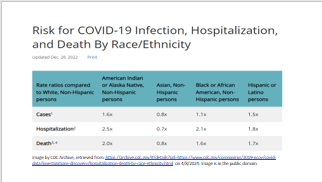 |  |
| *Suggested script/talking points:*  As an example, let’s review COVID-19 pandemic and the disparities in mortality rates in minority communities. This commonly adopted content area presents an opportunity for faculty to trace pandemics back through history in order to illustrate how to contextualize health outcomes in the appropriate historical/social lens.  Faculty must consciously remind learners that the disparities they see in practice are not NEW, rather disparities are cyclical manifestations of unaddressed SSDoH. | |
|  | |
| **Slide 16**  Structural causes of pandemics | **Big Picture**: Teaching sessions can use historical references to highlight how structural inequities contributed to prior pandemics similar to the COVID-19 pandemic |
| 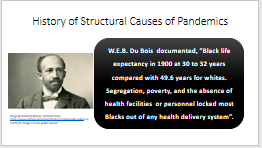 |  |
| *Suggested script/talking points:*  The same structural barriers to health highlighted by the current pandemic are merely reiterations of the same structural inequities that plagues communities of color. Read quote by W.E.B DuBois **Black life expectancy in 1900 at 30 to 32 years compared with 49.6 years for whites. Segregation, poverty, and the absence of health facilities or personnel locked most Blacks out of any health delivery system**  To understand the impact of COVID-19 fully, it's essential to connect the pandemic to structural causes. Structural factors such as socioeconomic disparities, access to healthcare, and systemic inequalities have significantly influenced the spread and severity of the virus.  For instance, communities with higher rates of poverty and limited access to healthcare have faced greater challenges in managing and recovering from COVID-19. These structural issues have exacerbated the health outcomes of marginalized populations, highlighting the urgent need for equitable public health responses.  By addressing these structural causes, we can better grasp why COVID-19 has had such varied impacts and why it's crucial to address these underlying issues to improve resilience against future health crises. | |
| **Slide 17**  Structural causes of pandemics | **Big Picture**: In the 1900s black physicians and social scientists rejected theories of racial inferiority and emphasized social factors as contributors to poor health among African Americans. |
| 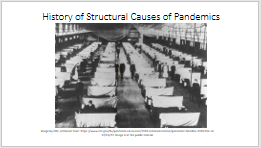 |  |
| *Suggested script/talking points:*  Segregated facilities were common for Richmond's African American community, impacting their health outcomes due to pervasive racism. Dr. Vanessa Gamble from George Washington University emphasized that the era's medicine cannot be discussed in isolation. Policies during that time had tangible health effects. In a paper on the 1918 pandemic's impact on African Americans, Gamble referred to sociologist W.E.B. Du Bois' analysis of the 1900 census, revealing higher death rates among African Americans for diseases like tuberculosis and pneumonia. Du Bois argued that these disparities were a result of social conditions, not racial susceptibilities. Between 1900 and 1920, black physicians and social scientists rejected theories of racial inferiority and emphasized social factors as contributors to poor health among African Americans. They sought to understand the underlying causes and refute claims of racial inferiority made by individuals like Frederick L. Hoffman.  We must not repeat the mistakes of the past and miss the call to attribute health outcomes to the structural and social determinants of health. | |
| **Slide 18**  Structural Differential: Anti-racist tool | **Big Picture**: Introducing the structural differential as an anti-racist teaching methodology |
| 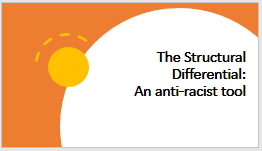 |  |
| *Suggested script/talking points:*  The COVID-19 pandemic exemplifies how health disparities arise from historical and structural inequities.  The structural differential is a standardized methodology that integrates structural competency into case-based presentations. It systematically incorporates contextual structural inequities, as seen in the COVID-19 pandemic, and disrupts clinical reasoning that reflexively uses race as surrogate for structural and social determinants of health.  The upcoming section will outline the structural differential's components and demonstrate its use in teaching anti-racist case conferences. | |
| **Slide 19**  How to implement structural differential | **Big Picture**: All types of case-based teaching are opportunities to guide learners on developing a structural differential. |
| 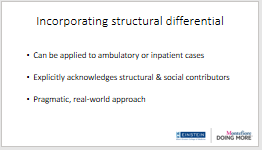 |  |
| *Suggested script/talking points:*  The structural differential can be applied to ambulatory or inpatient cases and is a pragmatic real-world approach to clinical care that explicitly acknowledges structural and social contributors. | |
| **Slide 20**  Structurally Competent Learning Goals | **Big Picture**:  The structural differential can easily be connected to a structurally competent learning goal for the teaching session |
| 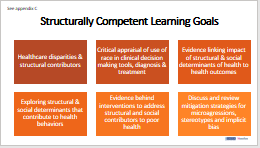 |  |
| *Suggested script/talking points:*  The following are examples of categories of structurally competent learning goals. We will go through each of them and give examples. You can pick one category to help guide the structurally competent learning goal you develop for your teaching session.  [If more examples needed:]   - *Why are Black, Latinx and Native American individuals more likely to be diagnosed with asthma, diabetes, etc.?* - *Critically appraise use of race in eGFR assessments* - *Highlight evidence linking environmental pollutant exposure to asthma* - *In instances of “non-compliance,” inquire about etiology, ex. medication affordability, stressors, etc.* - *Highlight evidence linking housing first programs to improved mental health* - *Acknowledge potential for implicit bias when addressing a patient who previously used injection drugs* | |
|  |  |
|  |  |
|  | |
|  |  |
|  |  |
|  | |
| **Slide 21**  How to implement structural differential | **Big Picture:** When constructing a teaching session using a case-based model, learners should be encouraged to create a structural differential. This serves as an entry point for discussions that relate to a specific structurally competent learning goal set for the session. |
| 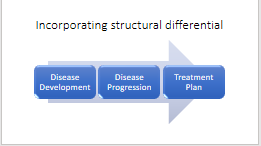 |  |
| *Suggested script/talking points:*  As learners practice using the structural differential, teaching points can be explored in multiple areas of case presentation, including highlighting:   - - Structural contributors to disease development, example: environmental exposure links to asthma or lead poisoning   - Structural contributors to delayed diagnosis or disease progression, example: lack of insurance and progression to end stage renal disease   - Structural barriers/facilitators to treatment plan, example: housing programs to reduce readmission rates among individuals experiencing homelessness | |
| **Slide 22**  Structural Competency Differential | **Big Picture**: Overview of the multi-step clinical reasoning process to create a structurally competent differential for medical case presentations. |
| 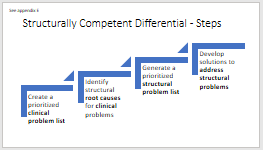 |  |
| *Suggested script/talking points:*  This is an overview of the steps of the structural differential. We’ll go through each of these steps in detail using a case example to help illustrate their use.  **First,** learners **create** a prioritized clinical problem list. Here it’s important to ensure congruity between patient and clinician problem lists.  **Second,** learners **identify** structural root causes for clinical problems. Here, it’s important to elicit upstream structural and social determinants of health that contribute to clinical problems, and integrate a historical context.  **Third,** learners **generate** a prioritized structural problem list. Here it’s important to incorporate patient priorities, preferences, and concerns; and prioritize urgent problems and problems for which clinical and community resources are available.  **Finally,** learners **develop** solutions to address structural problems. Here, they imagine individual-level, health system-level, community level and population-level solutions; incorporate patient priorities, preferences, and concerns (vital step); consider individual and community strengths/assets; and partner with an interdisciplinary team participating in community-led efforts. | |
| **Slide 23**  Case Presentation Example | **Big Picture**: Intro to walking through an example case with the large participant group |
| 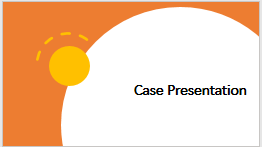 |  |
| *Suggested script/talking points:*  The following slides will walk the participants through how to implement a structural differential in a case presentation. | |
| **Slide 24**  Step 1: Clinical Problem list | **Big Picture**: Start by having learners create a prioritized clinical problem list |
| 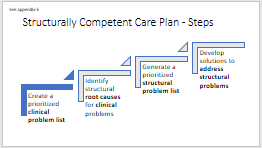 |  |
| *Suggested script/talking points:*  The first step is to **create** a **prioritized clinical problem list**. This is what we might traditionally do as a first step in our existing case conferences. What’s important here is to ensure congruity between patient and clinician problem lists. | |
| **Slide 25**  Example Case Presentation | **Big Picture**: Introduce case presentation example |
| 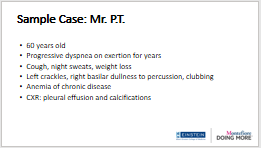 |  |
| *Let’s introduce Mr. P.T.:*  *Mr. P.T. (preferred pronouns he/his) is a 60-year-old who presents to the emergency room with progressive dyspnea on exertion for years, worse in the past three months. He has cough but no sputum production or wheezing. He has been experiencing night sweats and weight loss. His physical exam is notable for left basilar, fine end-inspiratory crackles, right sided basilar dullness to percussion and clubbing. Laboratory studies are notable for an anemia of chronic disease. CXR shows a right sided pleural effusion and pleural calcifications.* | |
| **Slide 26**  Start with clinical problem list | **Big Picture**: Start with clinical problem list |
| 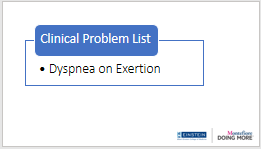 |  |
| *Suggested script/talking points:*  Here, a traditional clinical problem list might be dyspnea on exertion. Both the clinician and the patient are concerned about this symptom and want to diagnose the underlying etiology. | |
| **Slide 27**  Step 2 structural differential. Time Check 30:00 | **Big Picture**: Educators should have learners identify potential structural and social determinants of the clinical problem |
| 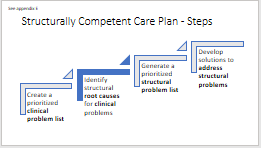 |  |
| *Suggested script/talking points:*  The next step of the structural differential is to:  **Identify** structural root causes for clinical problems. This involves:  Eliciting upstream structural and social determinants of health that contribute to clinical problems, and integrating a historical context. | |
| **Slide 28**  Root Cause Diagram | **Big Picture**: Use a root cause diagram to brainstorm and visualize structural and social root causes that contribute to a patient’s clinical presentation |
| 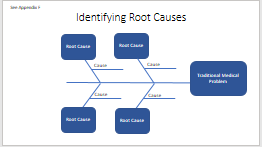 |  |
| *Suggested script/talking points:*  For this step, it’s helpful to use a root cause diagram to brainstorm and visualize structural and social root causes that contribute to a patient’s clinical presentation. A root cause diagram can help learners conceptualize both distal and proximal root causes. | |
| **Slide 29**  Social History | **Big Picture**: Reference a robust social history to identify structural and social determinants of the clinical problem. |
| 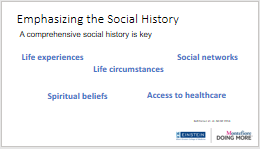 |  |
| *Suggested script/talking points:*  Root causes should be derived from a patient’s social history and lived experience. This necessitates generating a comprehensive social history, which could include:  **Life experiences**   - Education - Job history - Military service - Incarceration - Immigration - Key experiences   **Life circumstances**   - Family structures - Obligations - Housing - Neighborhood - Food security   **Spiritual beliefs**  **Social networks**  **Access to healthcare**   - Health literacy - Health insurance - Barriers to healthcare access - Medication affordability | |
| **Slide 30**  Documenting social needs | **Big Picture**: Instruct educators to expand awareness about social history documentation to learners. |
| 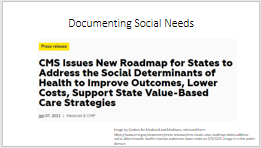 |  |
| *Suggested script/talking points:*  Many healthcare systems are now adopting universal screening for social needs that can also inform a root cause analysis. It assesses needs like safe/stable housing, housing conditions, utility needs, food insecurity, transportation needs, medication affordability, child/elder care, legal needs and personal safety. The screener is available in multiple languages and results can be inputted into our electronic health record, EMR (electronic medical records). As of 2023, screening for social needs will soon be an adopted standard by the Centers for Medicare and Medicaid Services and The Joint Commission.  Explicitly documenting social and structural problems in problem lists is crucial as it shifts focus away from personal blame and patient-level factors. Instead, it redirects attention to the broader factors influencing a patient's decisions, behaviors, and circumstances. There are several ICD codes available to document the structural and social factors affecting patients, with a notable one being "Problems related to the social environment. | |
| **Slide 31**  Structural Vulnerability Assessment | **Big Picture**: A structural vulnerability assessment is also another tool that can be used to obtain social history helpful to developing a root cause analysis. |
| 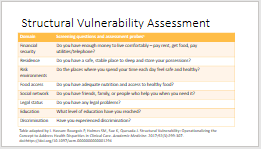 |  |
| *Suggested script/talking points:*  A structural vulnerability assessment is also another tool that can be used to obtain social history helpful to developing a root cause analysis. Note, this tool explicitly asks about experiences of discrimination. | |
| **Slide 32**  All session rubric component | **Big Picture**: Review a social history for the example clinical scenario |
| 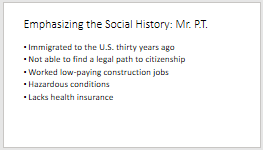 |  |
| *Suggested script/talking points:*  Now that we have some tools for obtaining a social history, let’s go back to Mr. P.T.  After obtaining a social history and screening for social needs/vulnerabilities, we learn the following:  *Mr. P.T. is immigrated to the U.S. thirty years ago in search of better economic opportunity for himself and his family. He was not able to find a legal path to citizenship, and spent years working low-paying, temporary construction jobs. Because of his immigration status, he often worked in hazardous conditions without appropriate protective equipment. He has delayed seeking care for his breathing problems because he lacks health insurance.* | |
| **Slide 33**  Refining Differential | **Big Picture**: Connect the sequence of clinical reasoning of step 1 and step 2 of creating a structural differential. |
| 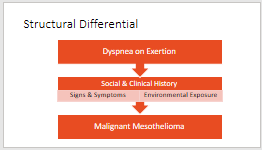 |  |
| *Suggested script/talking points:*  Now that we have our social history, we can begin to refine our differential for this patient. Taking into account Mr. P.T.’s social history of environmental exposure that increases his risk of asbestosis, and his clinical signs/symptoms, we are highly suspicious for malignant mesothelioma.  Let’s now help our learners identify some of the structural root causes and see how a root cause diagram for this patient might look like.  *After incorporating structural root causes and upon further medical workup, Mr. P.T. is diagnosed with asbestosis with malignant pleural mesothelioma. Mr. P.T. may be a candidate for surgical resection, which is known to prolong survival, but requires extensive testing and staging prior to surgery.* | |
| **Slide 34**  Case specific structural contributors’ context | **Big Picture**: Research the historic context of the structural determinants of health that will be highlighted in clinic presentations. |
| 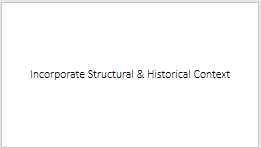 |  |
| *Suggested script/talking points:*  For our root cause analysis, not only must we obtain a social history from our patients, but it’s also important to include both the structural and historical context that has impacted our patients’ lives.  To do this, it’s important to:   - Gather information and perspectives from outside the clinical context - Identify policies, procedures, systems, institutions, forces through: Reading through literature, conversations with patients, community leaders, advocacy groups. | |
| **Slide 35**  Example of policy structural contributors | **Big Picture**: Policy contributors to structural barriers to care. |
| 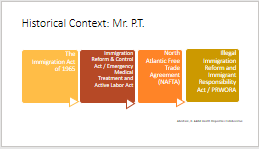 |  |
| *Suggested script/talking points:*  In the clinical scenario of Mr. P.T., several immigration policies directly led to his current presentation:   - Immigration Act of 1965 eliminated national origin admission quotas which previously favored northern and western white European immigrants. - 1986 Immigration Reform and Control Act gave legal status to some undocumented immigrants and encouraged guest farmworkers - Emergency Medical Treatment and Active Labor Act provided federal funding for emergency care regardless of immigration status but offered no coverage for preventative health and left it to states to define “emergent” care. Allows coverage for Mr. PT’s current inpatient care under Emergency Medicaid. - Global policies like NAFTA in 1994 moved multinational corporations into Mexico, forcing unemployed farm workers to migrate. Global economic inequity led to increased pressures for immigration and likely contributed to Mr. PT’s need to immigrate to the U.S. - 1996 Illegal Immigration Reform and Immigrant Responsibility Act and the Personal Responsibility and Work Opportunity Reconciliation Act made it easier to deport and deny welfare benefits to undocumented immigrants. - Recent political narrative of fear and oppression made it harder for undocumented immigrants to seek care. - Affordable Care Act provided no subsidies for undocumented immigrants to afford health insurance.   These policies likely contributed to Mr. PT delaying seeking treatment and not being able to afford health insurance or receive preventative care. It also made it easier to exploit workers like Mr. PT in unsafe/hazardous work conditions. | |
| **Slide 36**  Literature to support structural | **Big Picture**: Highlight evidence of structural barriers to health. |
| 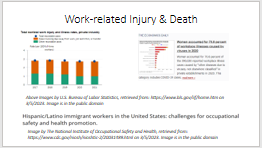 |  |
| *Suggested script/talking points:*  A review in the American Journal of Public Health noted that immigrant workers are likely to be particularly vulnerable to work related death and injury/illness due to:   - Stratification in hiring - Fear of reporting health and safety problems on the part of immigrants - Language barriers - Poor access to healthcare   A paper looking at racial discrimination and work-related illness noted that minoritized populations, particularly Hispanics, are more likely to experience work related illness and these associations were mediated by experiences of racial harassment and discrimination. | |
| **Slide 37**  Example Fishbone Diagram | **Big Picture**: An example fishbone diagram that incorporates Mr. PT’s unique social circumstances and broader structural and historical forces affecting his clinical presentation |
| 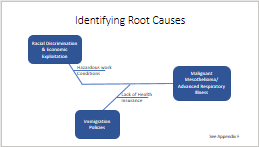 |  |
| *Suggested script/talking points:*  An example fishbone diagram that incorporates Mr. PT’s unique social circumstances and broader structural and historical forces affecting his clinical presentation, might look like this. For Mr. P.T., racial discrimination and economic exploitation led to hazardous work conditions. Immigration policies led to lack of health insurance. | |
| **Slide 38**  Step 3 of structural differential | **Big Picture**: Introduction to transforming a clinical problem list into a structural problem list that includes the result of the root cause analysis using the social history. |
| 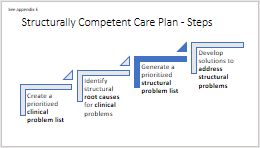 |  |
| *Suggested script/talking points:*  Once you place a disease within structural context, the next step is to develop a structural problem list. This involves:   - Add to problem list the explicit root causes identified through the root cause analysis - Prioritize problem list based on:   - Patient priorities, preferences, concerns   - Urgency of problems   - Availability of clinical and community resources | |
| **Slide 39**  Example Structural Problem List | **Big Picture**: Overview of example structural problem list |
| 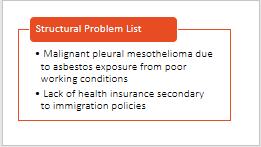 |  |
| *Suggested script/talking points:*  A structural problem list for Mr. PT might look like the following. | |
| **Slide 40**  Step 4 of structural differential | **Big Picture**: Overview of the fourth step of the structural differential: hypothesize solutions to structural problems |
| 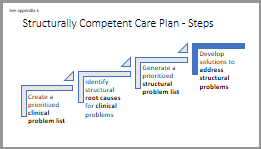 |  |
| *Suggested script/talking points:*  The final step is to develop solutions to address structural problems:  **Develop** solutions to address structural problems. This entails imagining individual-level, health system-level, community level and population-level solutions; considering patient priorities, experiences and concerns; considering individual and community strengths/assets; and partnering with an interdisciplinary team participating in community-led efforts. | |
| **Slide 41**  Structural Competency Care Plan | **Big Picture**: Tips for developing a patient-centered structurally competent care plan. |
| 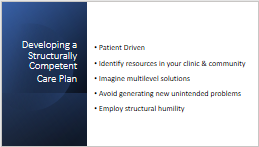 |  |
| *Suggested script/talking points:*  Solutions should reflect the following:  It is important that the care by patient driven. Our patients often have more experience navigating our social services system and are best able to take their prior experiences and priorities into account. It is vital to listen to our patient’s preferences and concerns. It is also important to be familiar with the resources available in your clinical setting and community, to push learners to imagine not only individual-level solutions but also community and policy level solutions. One should be careful to avoid generating new unintended problems, for example solutions that do not take into account resources needed to maintain viability/sustainability or cause additional hardship for the patient. Finally, it is important to approach structurally competent care plans with structural humility.  To employ structural humility:   - - Utilize an interdisciplinary team   - Participate in community-led (not physician led) advocacy initiatives | |
| **Slide 42**  Structural Plan | **Big Picture**: Example of creating a structural plan |
| 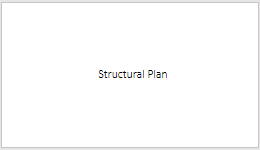 |  |
| *Suggested script/talking points:*  Returning to Mr. P.T’s clinical scenario:  *Mr. P.T. may be a candidate for surgical resection, which is known to prolong survival, but requires extensive testing and staging prior to surgery. In discussing options with Mr. P.T., he would like to pursue all options for therapy and treatment.* | |
| **Slide 43**  Patient Resources | **Big Picture**: Learn about and highlight available institutional and community resources to assist patients in overcoming structural barriers to health. |
| 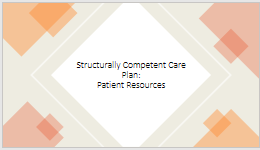 |  |
| *Suggested script/talking points:*  Know the resources that are available, with recognition access to variable resource will not be available in every healthcare context.  *Considering varying resources, investigate if your hospital has social workers and a medical legal partnership, as well as specialty medical and surgical services. In addition, if your institution has an active immigration working group comprised of physicians, psychologists, social workers, pharmacists, administrators, medical students, and researchers and other providers that advocate for immigrants within the healthcare system.* | |
| **Slide 44**  Example of Structurally Competent Care Plan | **Big Picture**: Walk through example structurally competent care plan with participants |
| 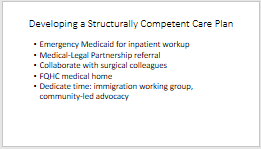 |  |
| *Suggested script/talking points:*  A structurally competent care plan for Mr. PT might include the following:  Enroll Mr. P.T. in Emergency Medicaid, conduct the majority of the workup while inpatient to maximize emergency Medicaid coverage.  Refer Mr. P.T. to a medical legal partnership for assistance with obtaining immigration status sufficient to apply for Medicaid (ex. PRUCOL).  In the meantime, reach out to colleagues in surgical specialties for charity services.  Join with community-based immigrants rights groups to advocate for universal health insurance, worker protections for undocumented immigrants, and legal pathways to citizenship. | |
| **Slide 45**  Structural Humility | **Big Picture**: Explain to participants the intention behind creating a structurally competent care plan is to connect patients to people and organizations that are better equipped to take on structural barriers to health |
| 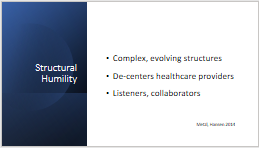 |  |
| *Suggested script/talking points:*  Structures are complex and evolving however are subject to change. Structural humility de-centers healthcare providers as principal agents of change, and re-centers patients and community with providers serving a supportive role. Employ structural humility  Recognize the complexity of structures  Decenter healthcare providers  Utilize an interdisciplinary team  Participate in community-led advocacy initiatives | |
| **Slide 46**  Quiz | **Big Picture**: Quiz to solidify understanding. Spend 3 minutes. |
| 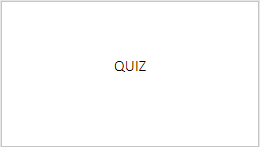 |  |
| *Suggested script/talking points:*  The following quiz was administered via a Zoom poll. Correct answers are bolded:  Which of the following should you do when building a structural differential (select all that apply):  **(a) Integrate historical context of disparities**  **(b) Incorporate patient priorities, preferences, concerns**  (d) Solve most structural problems during the clinic encounter  **(e) Name structural root causes of clinical problems** | |
| **Slide 47**  Small Group Exercise | **Big Picture**: Participants have to opportunity to apply the teaching method of developing a structural differential. |
| 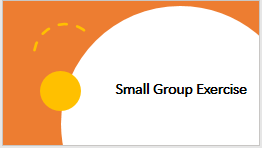 |  |
| *Suggested script/talking points:*  Let’s practice using the structural differential and creating a root cause fishbone diagram.  Small group of approximately 4-5 individuals. Each team works through the exercise on the next slide. | |
| **Slide 48**  Small Group Exercise. Time Check 58:00 | **Big Picture**: Link to small group exercise.  Spend 20 minutes |
| 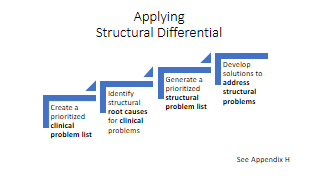 |  |
| *Suggested script/talking points:*  Google Doc Case Presentation:  Google Powerpoint Fishbone:  *P.T. (he/his) is a 48 year old with a past medical history of obesity and prior knee injury who is admitted to the hospital with recurrent swelling and severe pain in his left leg. Imaging studies show severe osteoarthritis of his knee with large effusion and extensive baker’s cyst. He requires opioid medications to alleviate the pain. P.T. is currently experiencing homelessness and living in a temporary basement dwelling after prior traumatic experiences within the shelter system. P.T. has been told he needs a knee replacement. He had a toe amputation for a prior infection in his right foot that did not heal properly leading to a deformed foot and consequently does not want surgery on his knee. He does not currently have a primary care physician.*   - 1. **Create a prioritized clinical problem list**   2. **Identify structural root causes for clinical problems**   3. **Generate a prioritized structural problem list**   4. **Develop solutions to address structural problems** | |
| **Slide 49**  Large Group Discussion. Time Check 78:00 | **Big Picture**: Open up discussion with participants using these three guiding questions. Spend 10 minutes. Allow waterfall chat responses in zoom. |
| 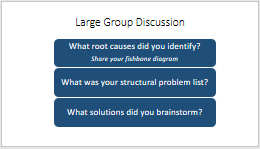 |  |
| *Suggested script/talking points:*  Follow the bullet points to guide large group discussion   - - Answers that can be provided are structural causes like issues with housing, food security, transportation, access to consistent healthcare. Solutions include the following:     - Connect patients to social services (e.g., financial aid, housing assistance).     - Provide low-cost or generic medication options.     - Partner with local food banks or nutrition programs.     - Use culturally competent care practices and advocate for anti-racist policies.     - Train healthcare staff on implicit bias and its effects on care delivery. | |
| **Slide 50**  Resources Time Check 88:00 | **Big Picture**: Resources for participants |
| 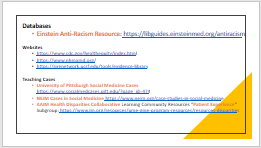 |  |
| *Suggested script/talking points:*  Here are some health disparities and social determinants of health resources you may find helpful to reference during your teaching or structurally competent case conference presentations. | |
| **Slide 51**  Resources | **Big Picture**: Resources for participants |
| 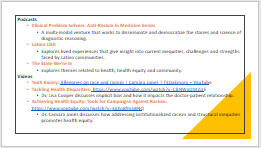 |  |
| *Suggested script/talking points:*  Here are some health disparities and social determinants of health resources you may find helpful to reference during your teaching or structurally competent case conference presentations. | |
