# Supplementary material for: Structural Competency: A Faculty Development Workshop Series for Anti-racism in Medical Education
Source: MedEdPORTAL. 2025 Feb 7;21:11492. doi: 10.15766/mep_2374-8265.11492 (PMC11802914; doi:10.15766/mep_2374-8265.11492)
Supplement: Supplementary file 1 — 1 - Introduction to SC.pptx1 - Facilitator Guide.docx1 - SC Rubric Handout.docx1 - Sample SC Learning Goals.docx2 - Resident Reports & Case-Based Presentations.pptx2 - Facilitator Guide.docx2 - Structural Differential Handout.docx2 - Small-Group Handout.docx3 - Demystifying SC.pptx3 - Facilitator Guide.docx3 - SC One-Minute Preceptor Handout.docx3 - SC SNAPPS Handout.docx3 - Role-Play Scenarios.docx4 - SC Hospital-Based Teaching.pptx4 - Facilitator Guide.docx4 - Daily Inpatient Checklist.docx4 - SC Discharge Checklist.docx4 - Small-Group Scenarios.docxPre- and Postsurveys.docx [file mep_2374-8265.11492-s001.zip › H. 2 - Small-Group Handout.docx]

**In small groups, faculty will read through the following clinical scenario and answer the four questions below, in the process completing a root cause analysis fishbone diagram of structural and social contributors.**

P.T. (he/his) is a 48 year old with a past medical history of obesity and prior knee injury who is admitted to the hospital with recurrent swelling and severe pain in his left leg. Imaging studies show severe osteoarthritis of his knee with large effusion and extensive baker’s cyst. He requires opioid medications to alleviate the pain. P.T. is currently experiencing homelessness and living in a temporary basement dwelling after prior traumatic experiences within the shelter system. P.T. has been told he needs a knee replacement. He had a toe amputation for a prior infection in his right foot that did not heal properly leading to a deformed foot and consequently does not want surgery on his knee. He does not currently have a primary care physician.

1. **Create a prioritized clinical problem list**

1. **Identify structural root causes for clinical problems using the fishbone diagram below**

1. **Generate a prioritized structural problem list**

1. **Develop solutions to address structural problems**

Traditional Medical Problem

Root Cause

Root Cause

Cause

Cause

**Root Cause**

Cause

Root Cause

Cause
