# Supplementary material for: Structural Competency: A Faculty Development Workshop Series for Anti-racism in Medical Education
Source: MedEdPORTAL. 2025 Feb 7;21:11492. doi: 10.15766/mep_2374-8265.11492 (PMC11802914; doi:10.15766/mep_2374-8265.11492)
Supplement: Supplementary file 1 — 1 - Introduction to SC.pptx1 - Facilitator Guide.docx1 - SC Rubric Handout.docx1 - Sample SC Learning Goals.docx2 - Resident Reports & Case-Based Presentations.pptx2 - Facilitator Guide.docx2 - Structural Differential Handout.docx2 - Small-Group Handout.docx3 - Demystifying SC.pptx3 - Facilitator Guide.docx3 - SC One-Minute Preceptor Handout.docx3 - SC SNAPPS Handout.docx3 - Role-Play Scenarios.docx4 - SC Hospital-Based Teaching.pptx4 - Facilitator Guide.docx4 - Daily Inpatient Checklist.docx4 - SC Discharge Checklist.docx4 - Small-Group Scenarios.docxPre- and Postsurveys.docx [file mep_2374-8265.11492-s001.zip › I. 3 - Demystifying SC.pptx]

## Slide 1
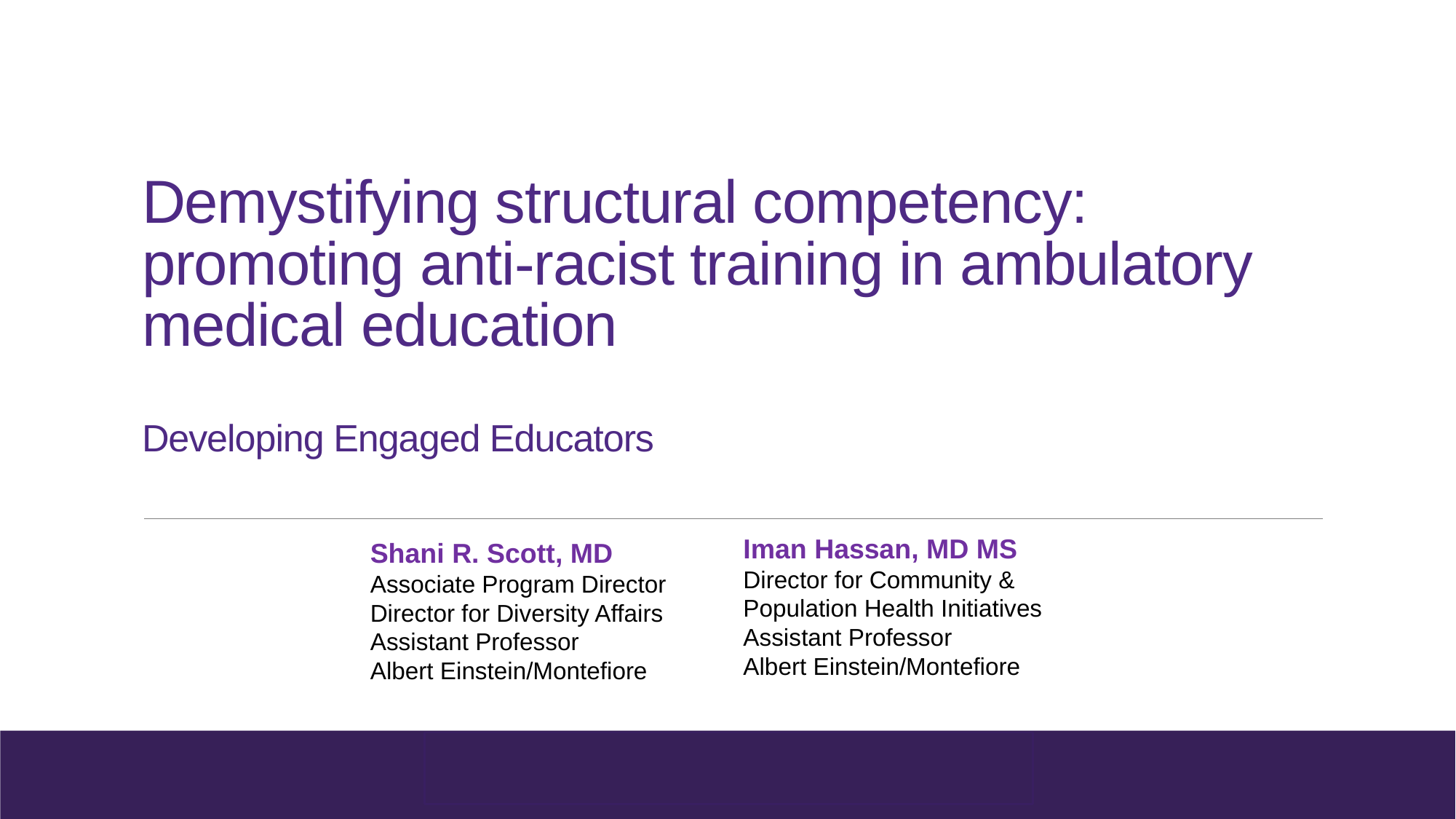

# Demystifying structural competency: promoting anti-racist training in ambulatory medical education Developing Engaged Educators
Iman Hassan, MD MS
Director for Community &
Population Health Initiatives
Assistant Professor
Albert Einstein/Montefiore
Shani R. Scott, MD
Associate Program Director
Director for Diversity Affairs
Assistant Professor
Albert Einstein/Montefiore

## Slide 2
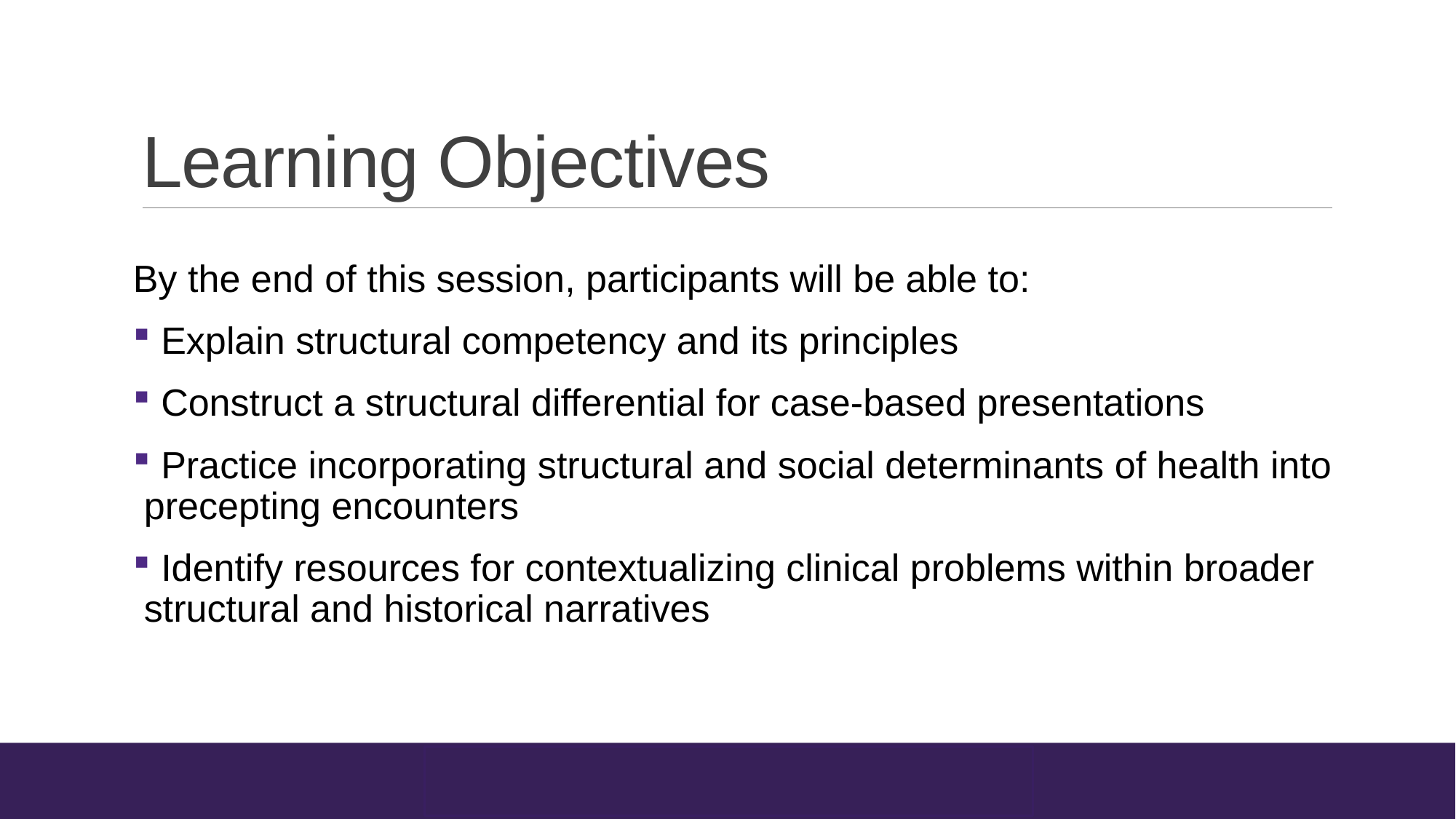

# Learning Objectives
By the end of this session, participants will be able to:
 Explain structural competency and its principles
 Construct a structural differential for case-based presentations
 Practice incorporating structural and social determinants of health into precepting encounters
 Identify resources for contextualizing clinical problems within broader structural and historical narratives

## Slide 3
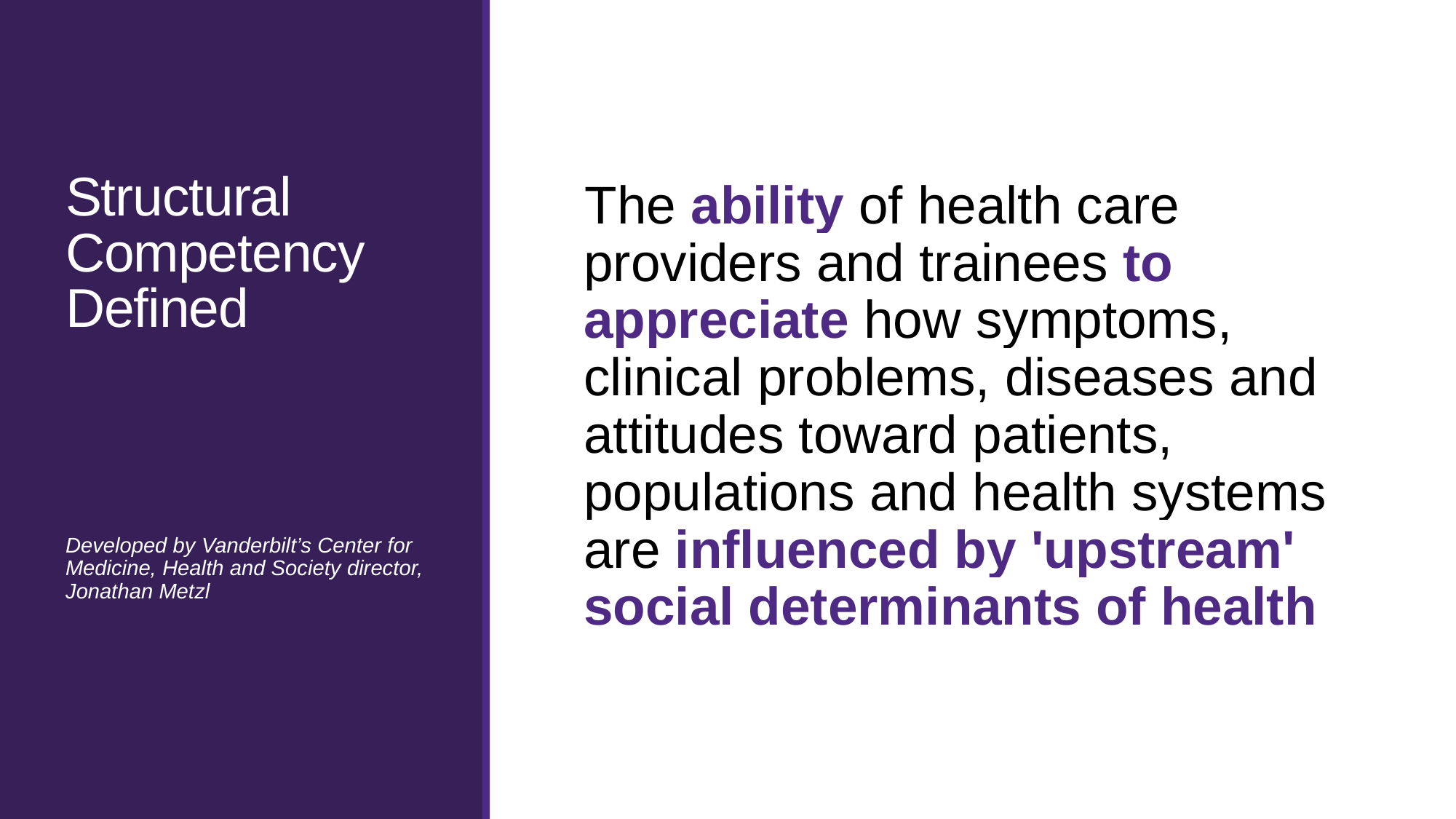

# Structural Competency Defined
The ability of health care providers and trainees to appreciate how symptoms, clinical problems, diseases and attitudes toward patients, populations and health systems are influenced by 'upstream' social determinants of health
Developed by Vanderbilt’s Center for Medicine, Health and Society director, Jonathan Metzl

## Slide 4
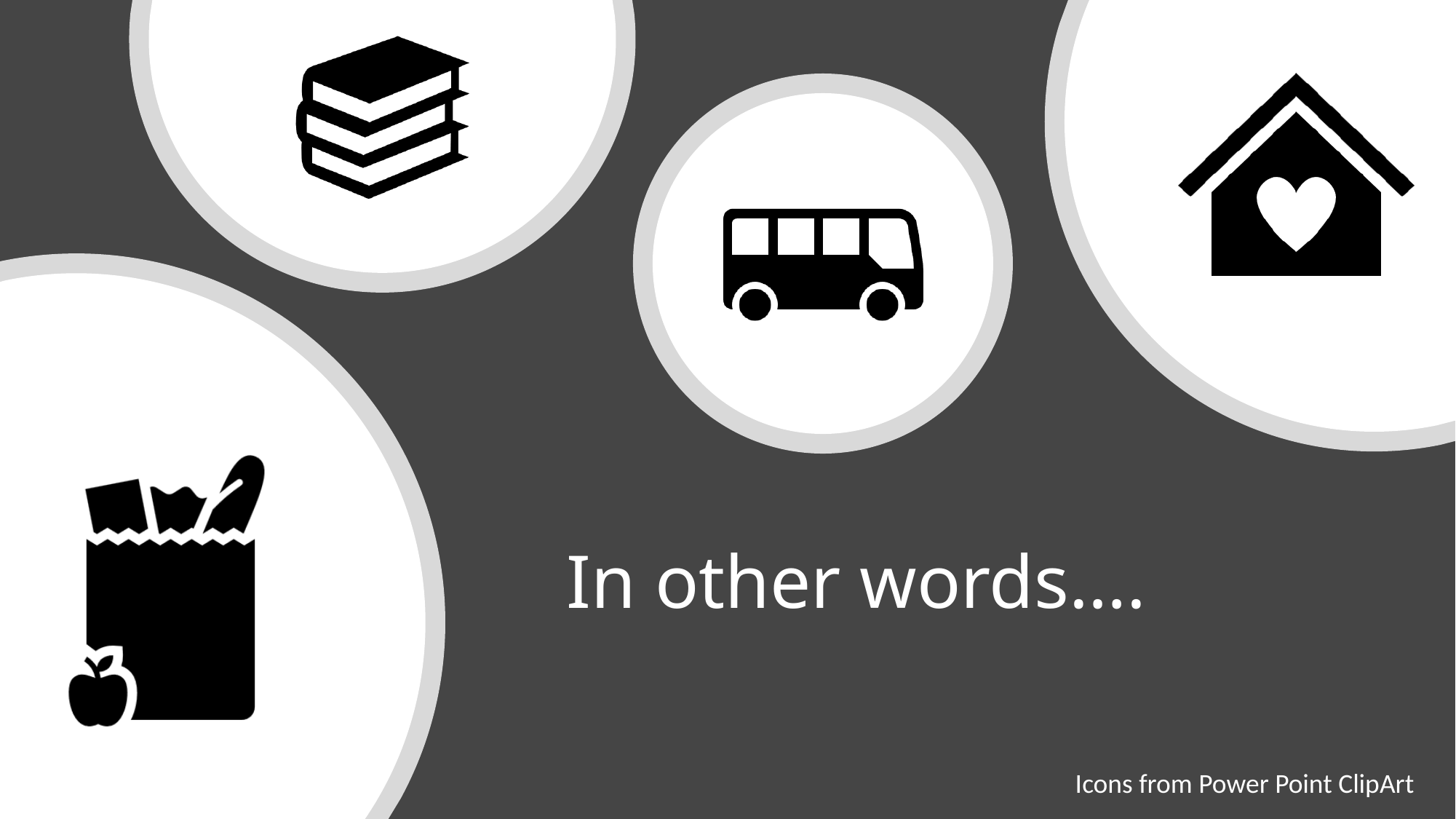

In other words….
Icons from Power Point ClipArt

## Slide 5
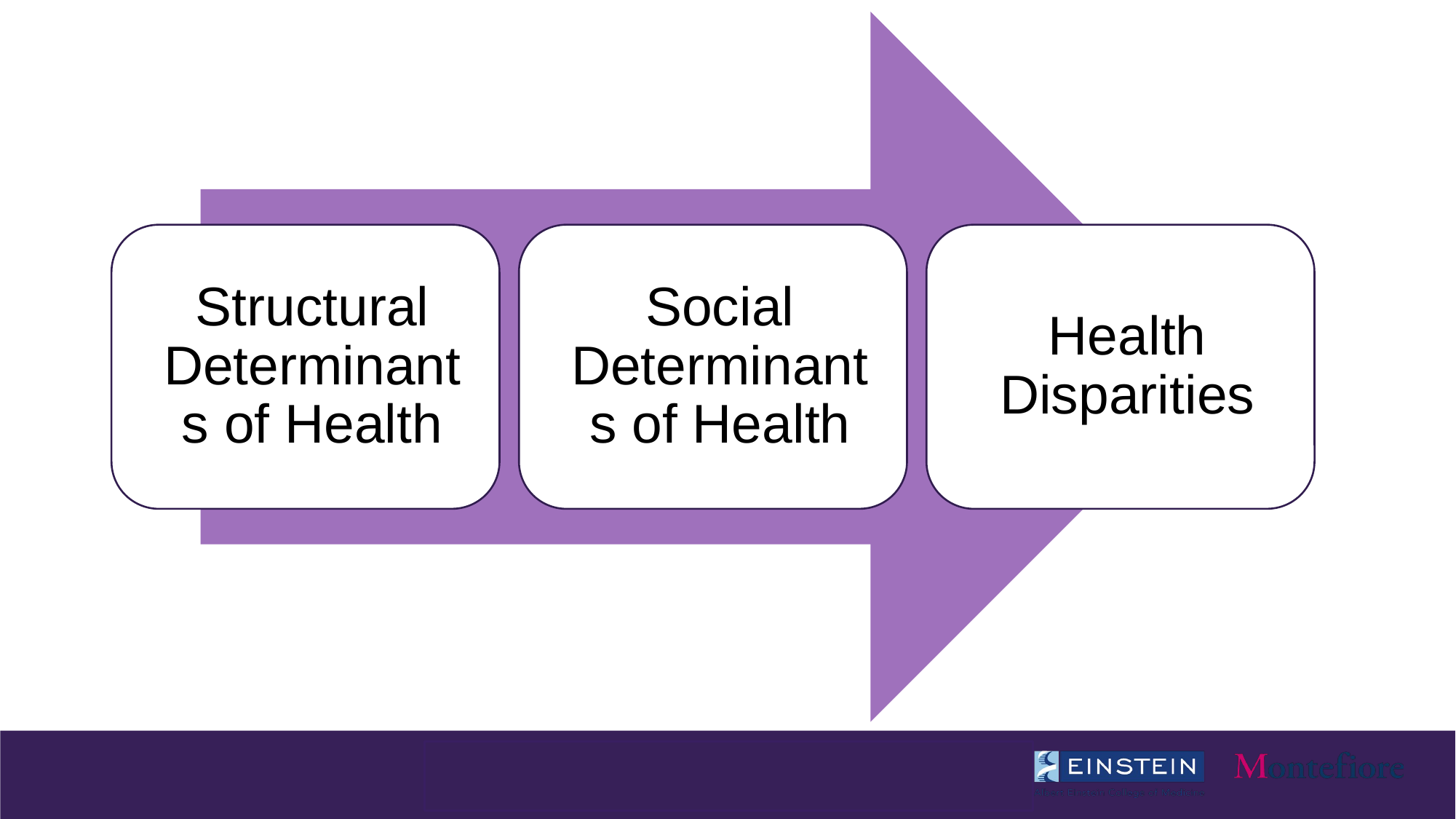

## Slide 6
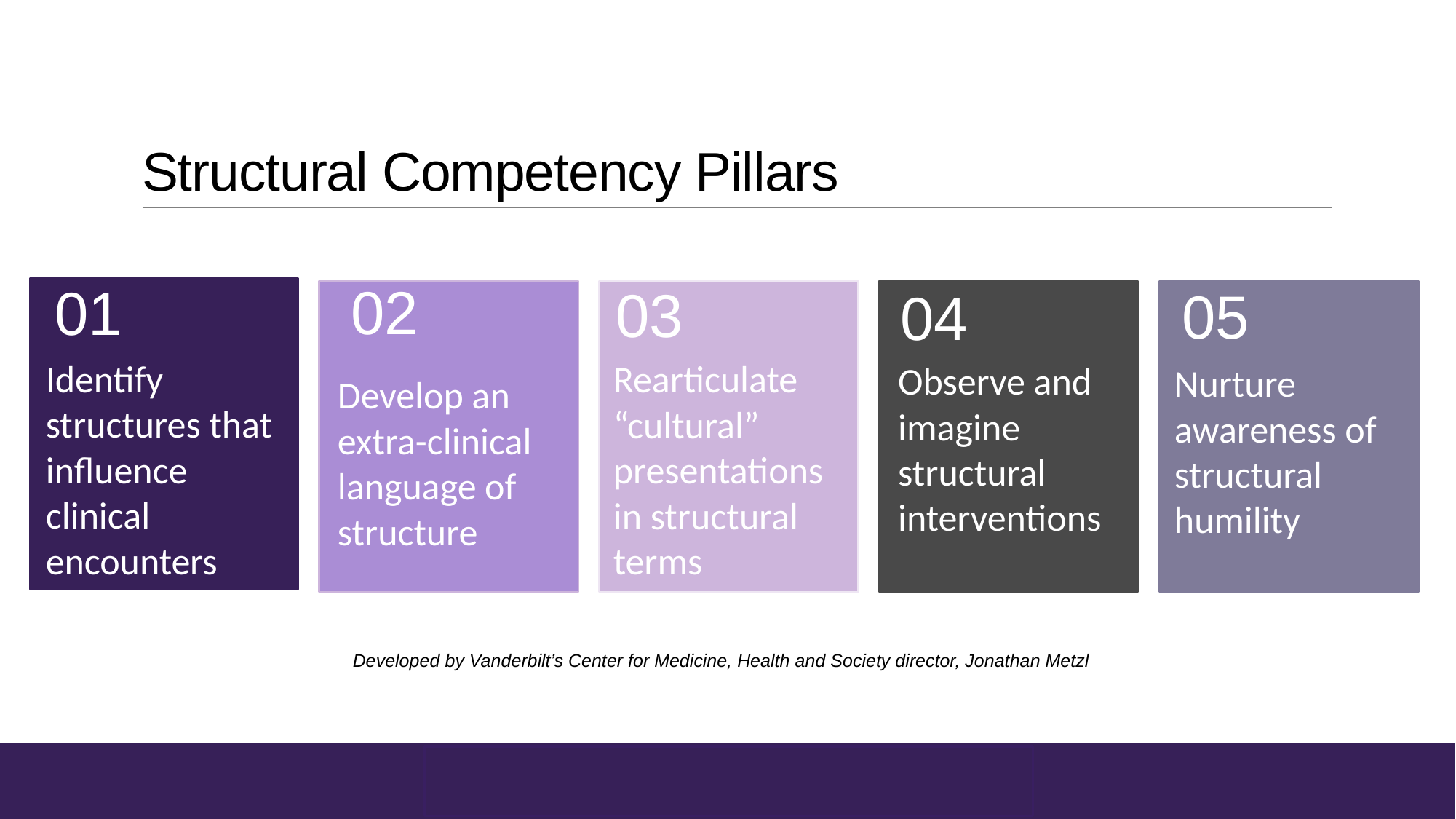

# Structural Competency Pillars
Identify structures that influence clinical encounters
Rearticulate “cultural” presentations in structural terms
Observe and imagine structural interventions
Nurture awareness of structural humility
Develop an extra-clinical language of structure
Developed by Vanderbilt’s Center for Medicine, Health and Society director, Jonathan Metzl

## Slide 7
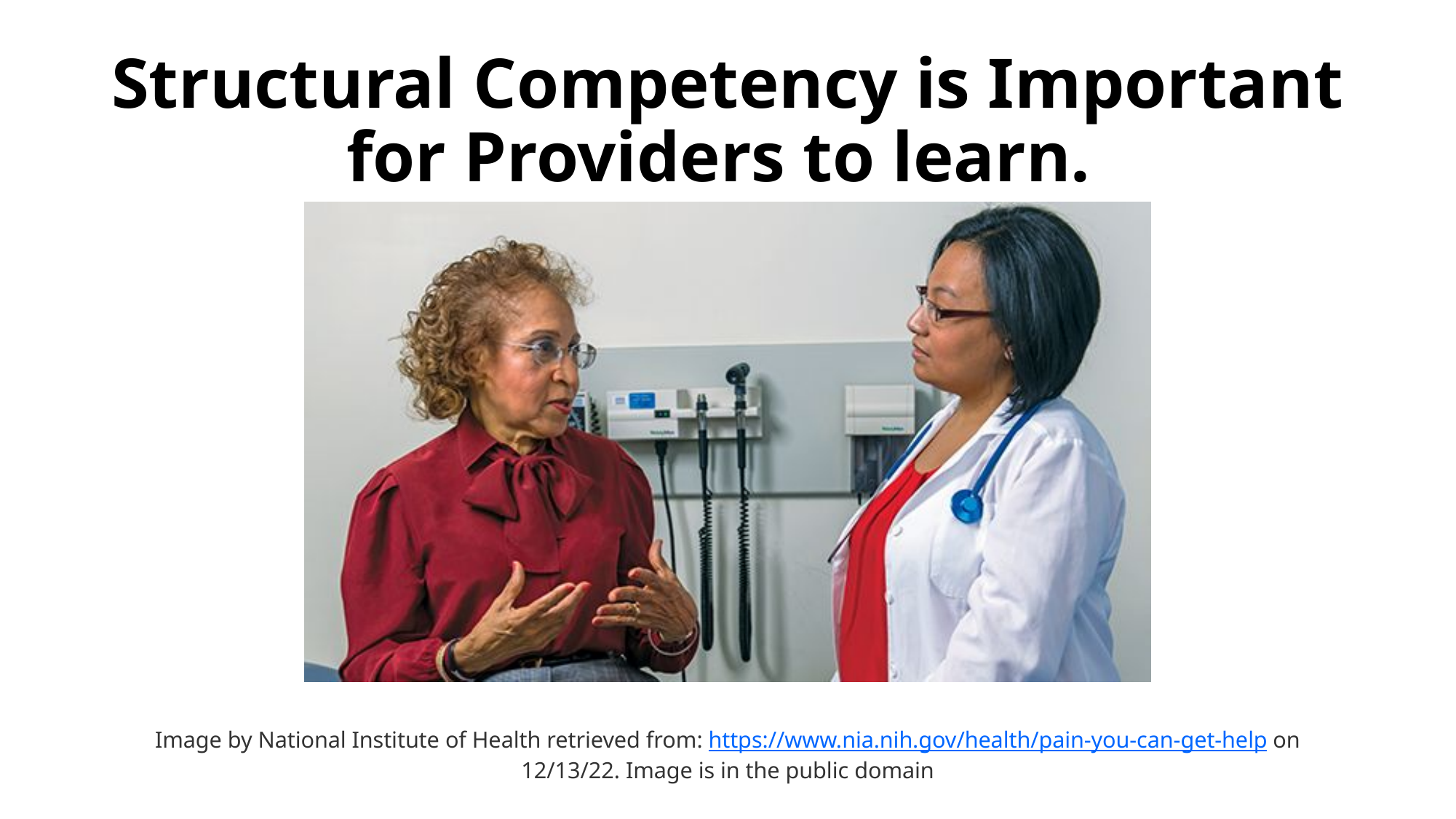

# Structural Competency is Important for Providers to learn.
Image by National Institute of Health retrieved from: https://www.nia.nih.gov/health/pain-you-can-get-help on 12/13/22. Image is in the public domain

## Slide 8
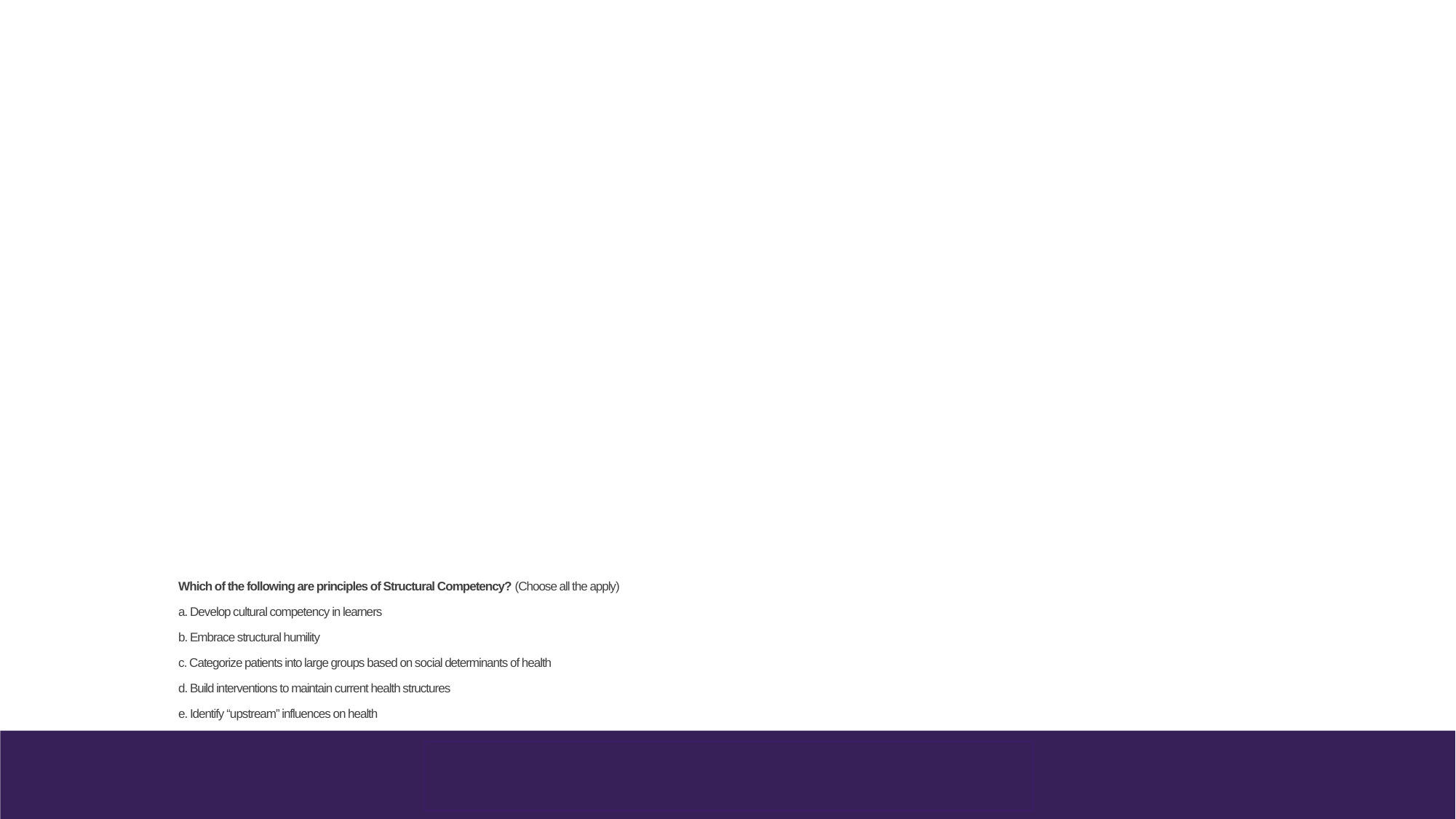

Which of the following are principles of Structural Competency? (Choose all the apply)
a. Develop cultural competency in learners
b. Embrace structural humility
c. Categorize patients into large groups based on social determinants of health
d. Build interventions to maintain current health structures
e. Identify “upstream” influences on health

## Slide 9
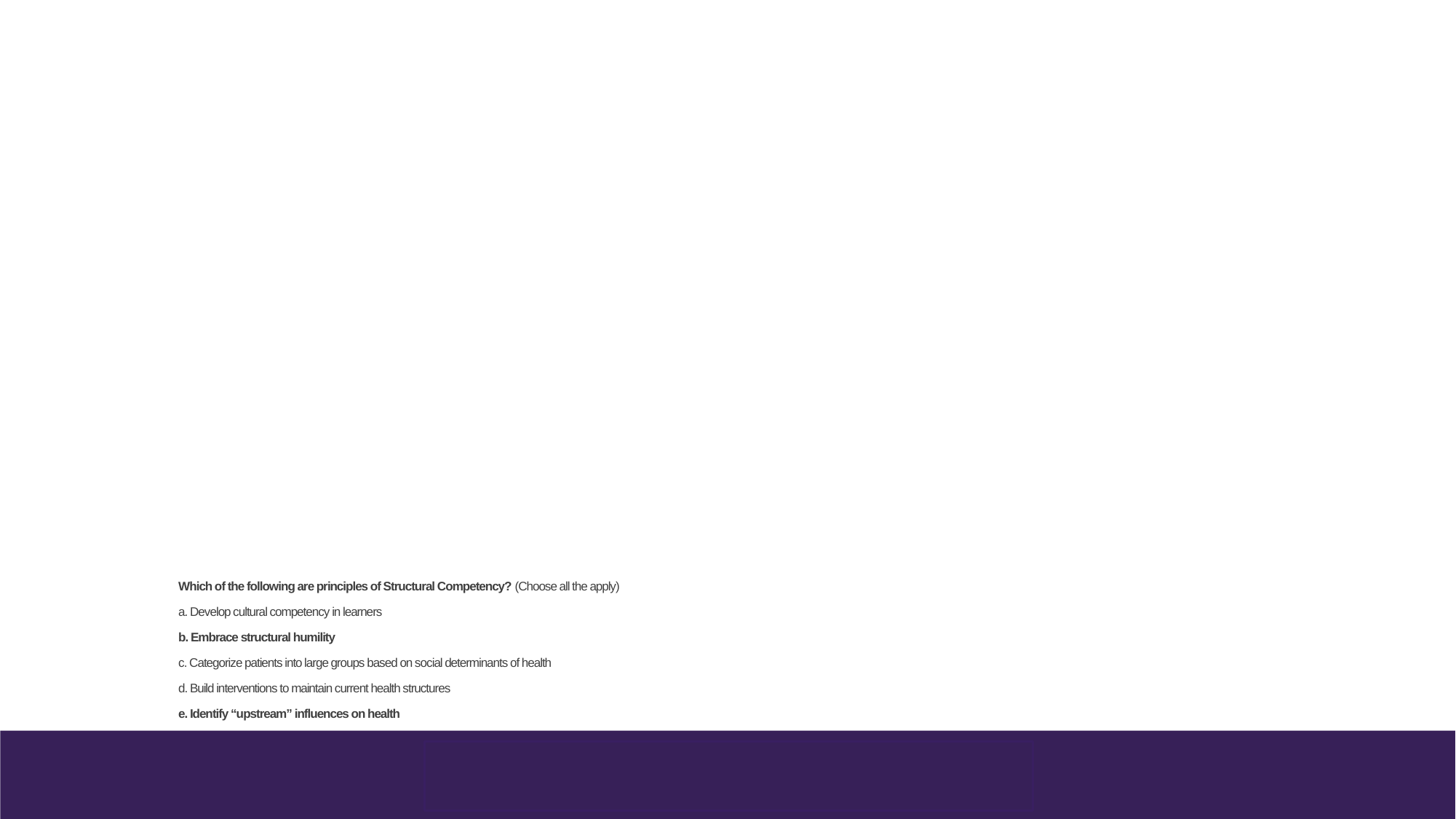

Which of the following are principles of Structural Competency? (Choose all the apply)
a. Develop cultural competency in learners
b. Embrace structural humility
c. Categorize patients into large groups based on social determinants of health
d. Build interventions to maintain current health structures
e. Identify “upstream” influences on health

## Slide 10
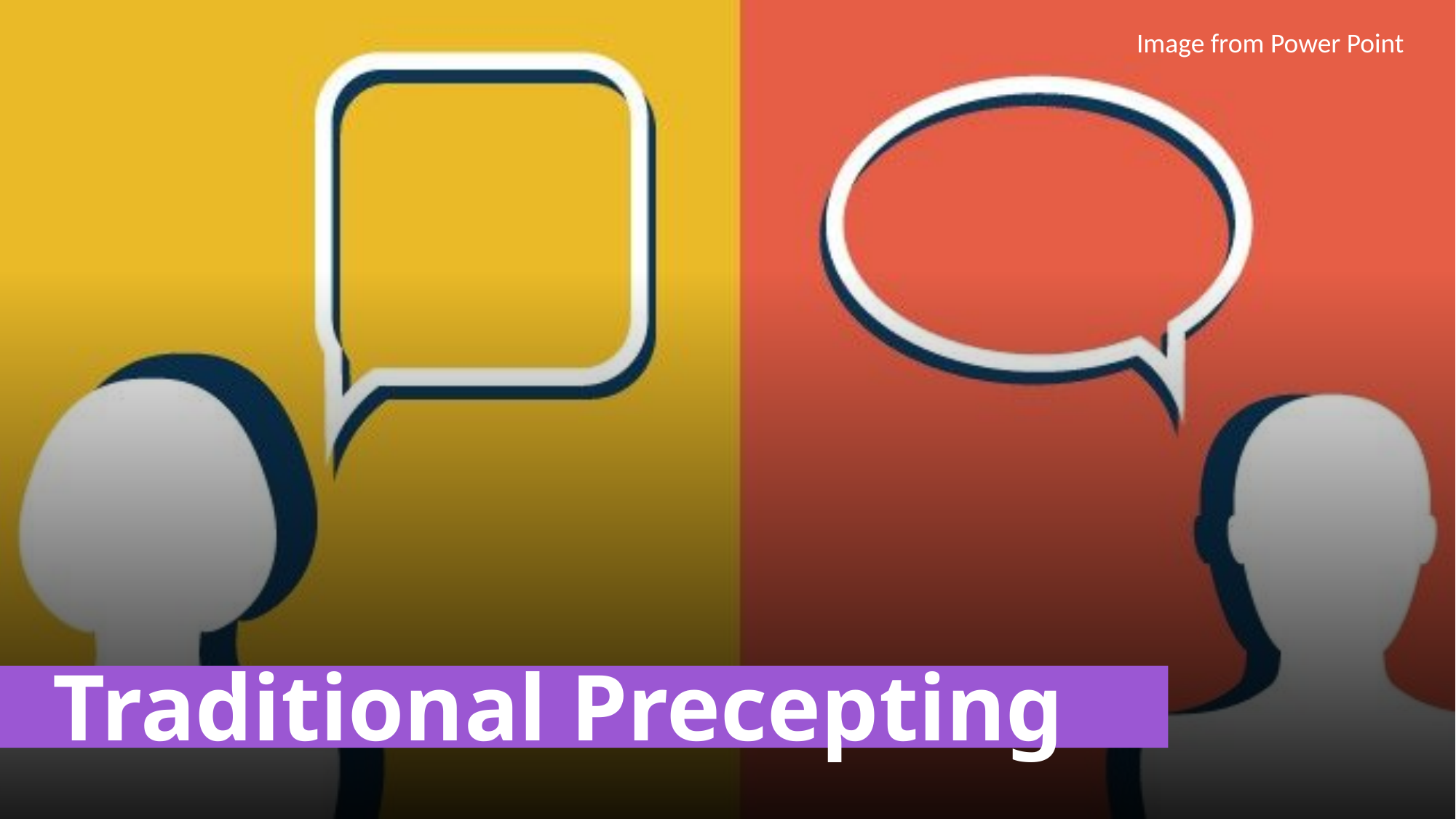

Image from Power Point
# Traditional Precepting

## Slide 11
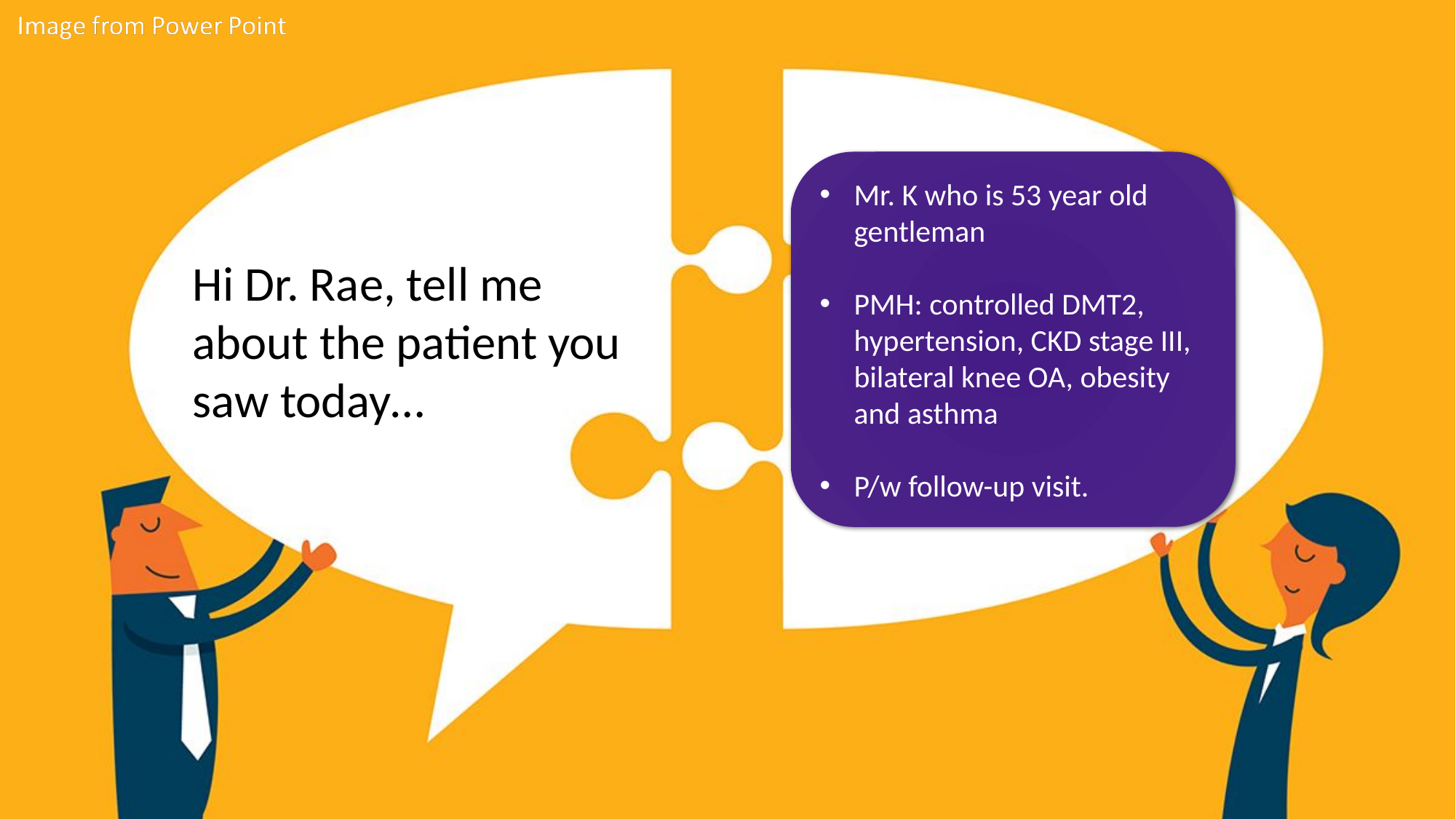

Mr. K who is 53 year old gentleman
PMH: controlled DMT2, hypertension, CKD stage III, bilateral knee OA, obesity and asthma
P/w follow-up visit.
Hi Dr. Rae, tell me about the patient you saw today…

## Slide 12
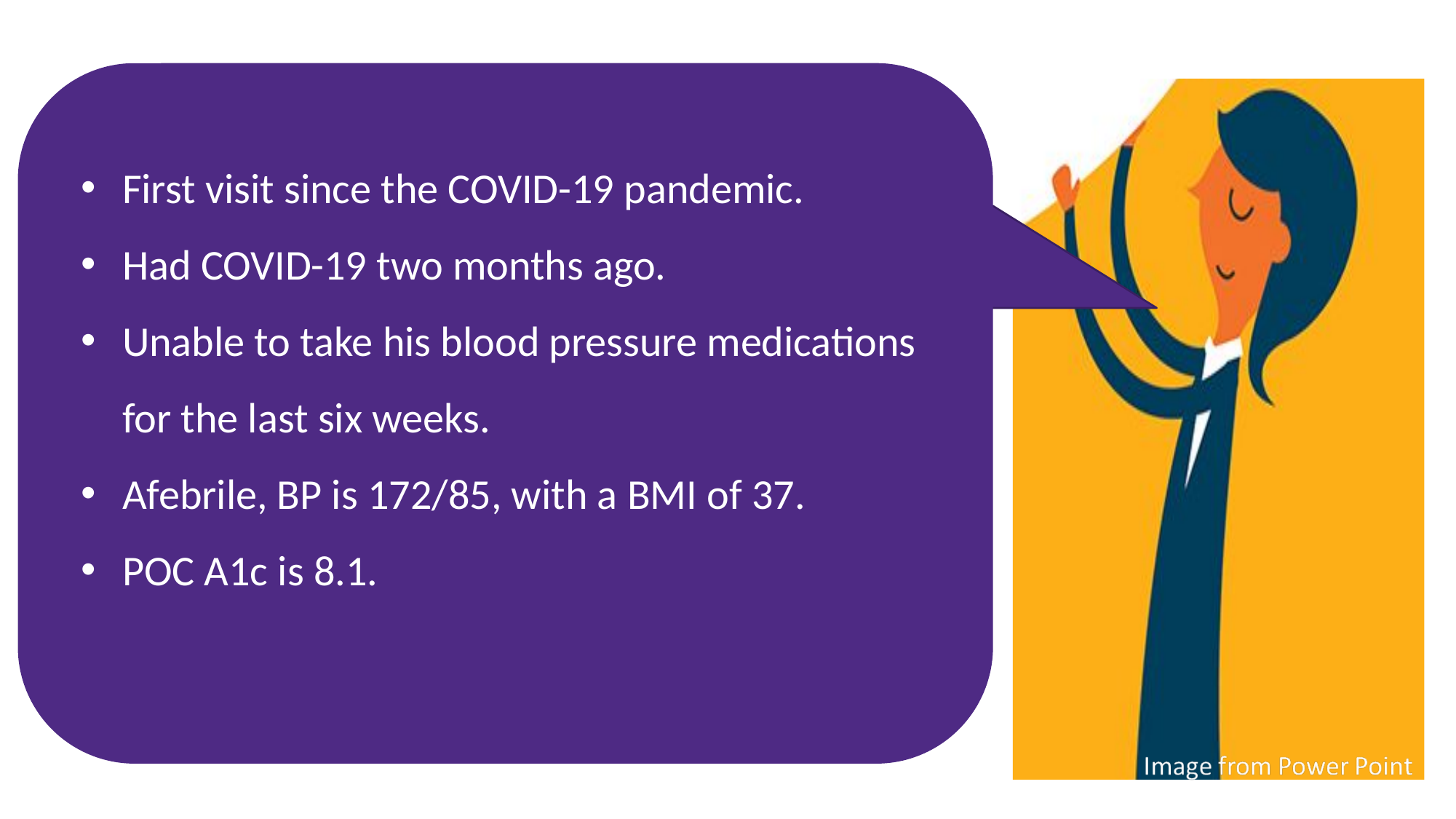

First visit since the COVID-19 pandemic.
Had COVID-19 two months ago.
Unable to take his blood pressure medications for the last six weeks.
Afebrile, BP is 172/85, with a BMI of 37.
POC A1c is 8.1.

## Slide 13
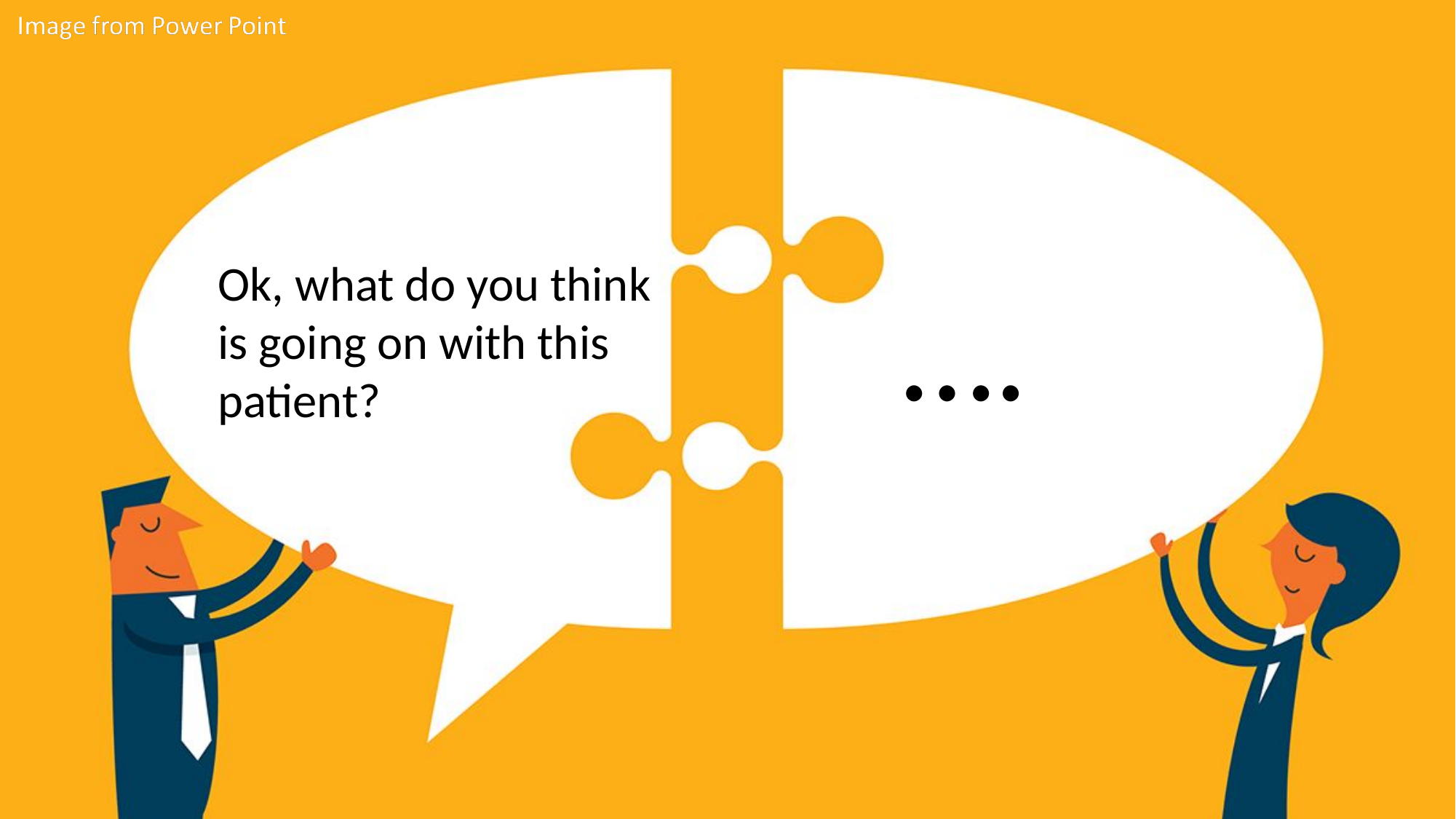

Ok, what do you think is going on with this patient?
….

## Slide 14
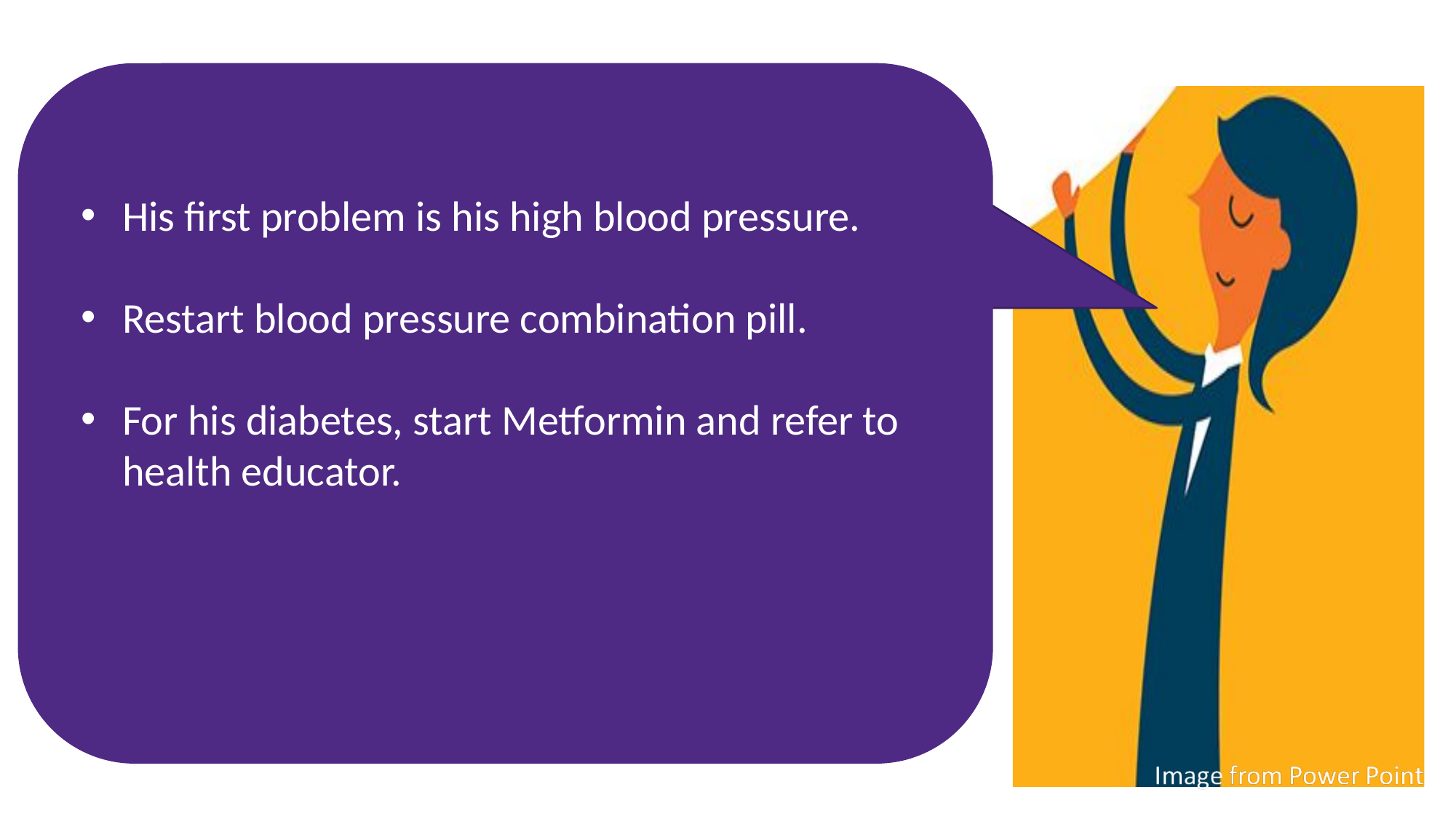

His first problem is his high blood pressure.
Restart blood pressure combination pill.
For his diabetes, start Metformin and refer to health educator.

## Slide 15
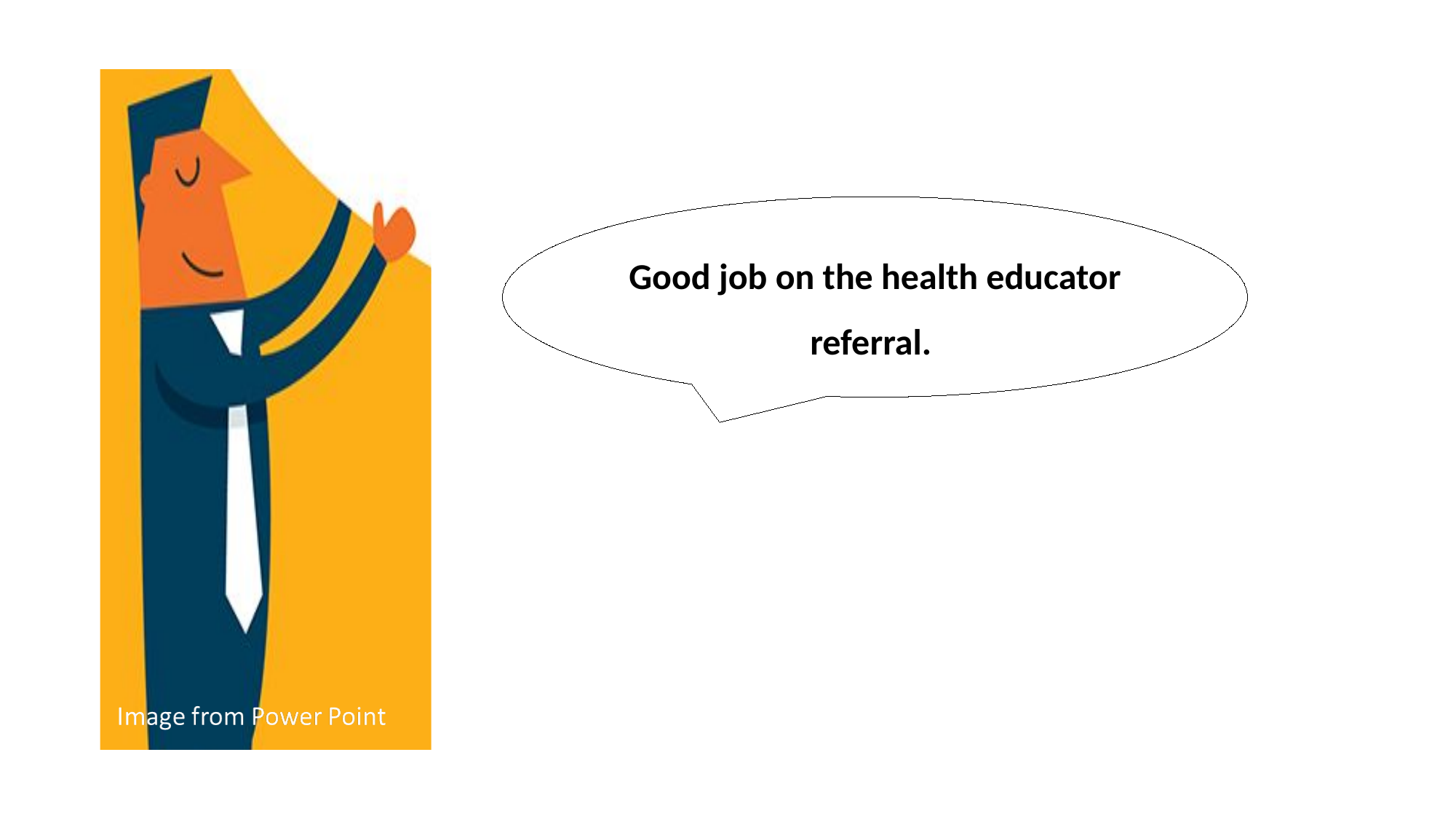

Good job on the health educator referral.

## Slide 16
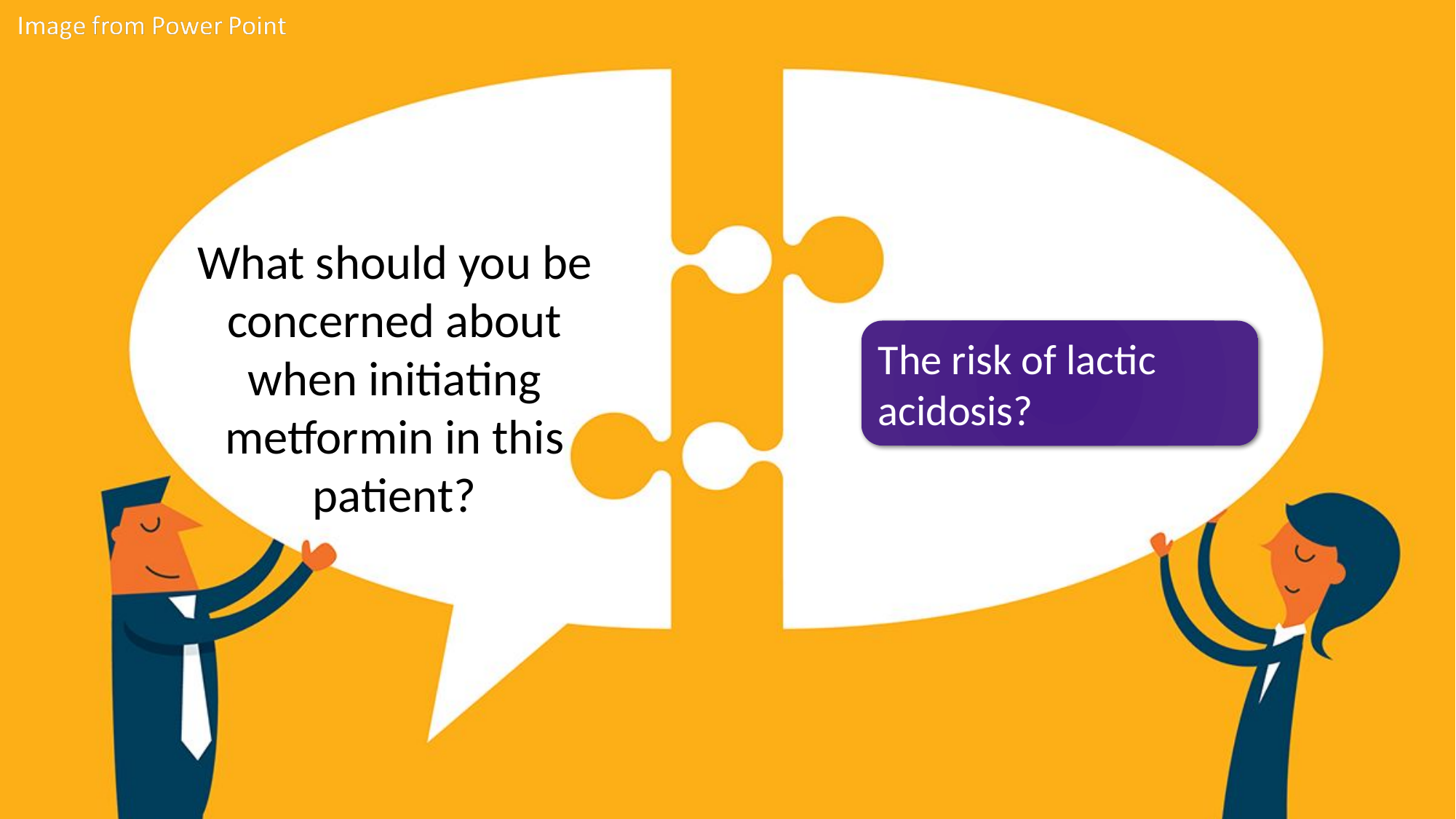

What should you be concerned about when initiating metformin in this patient?
The risk of lactic acidosis?

## Slide 17
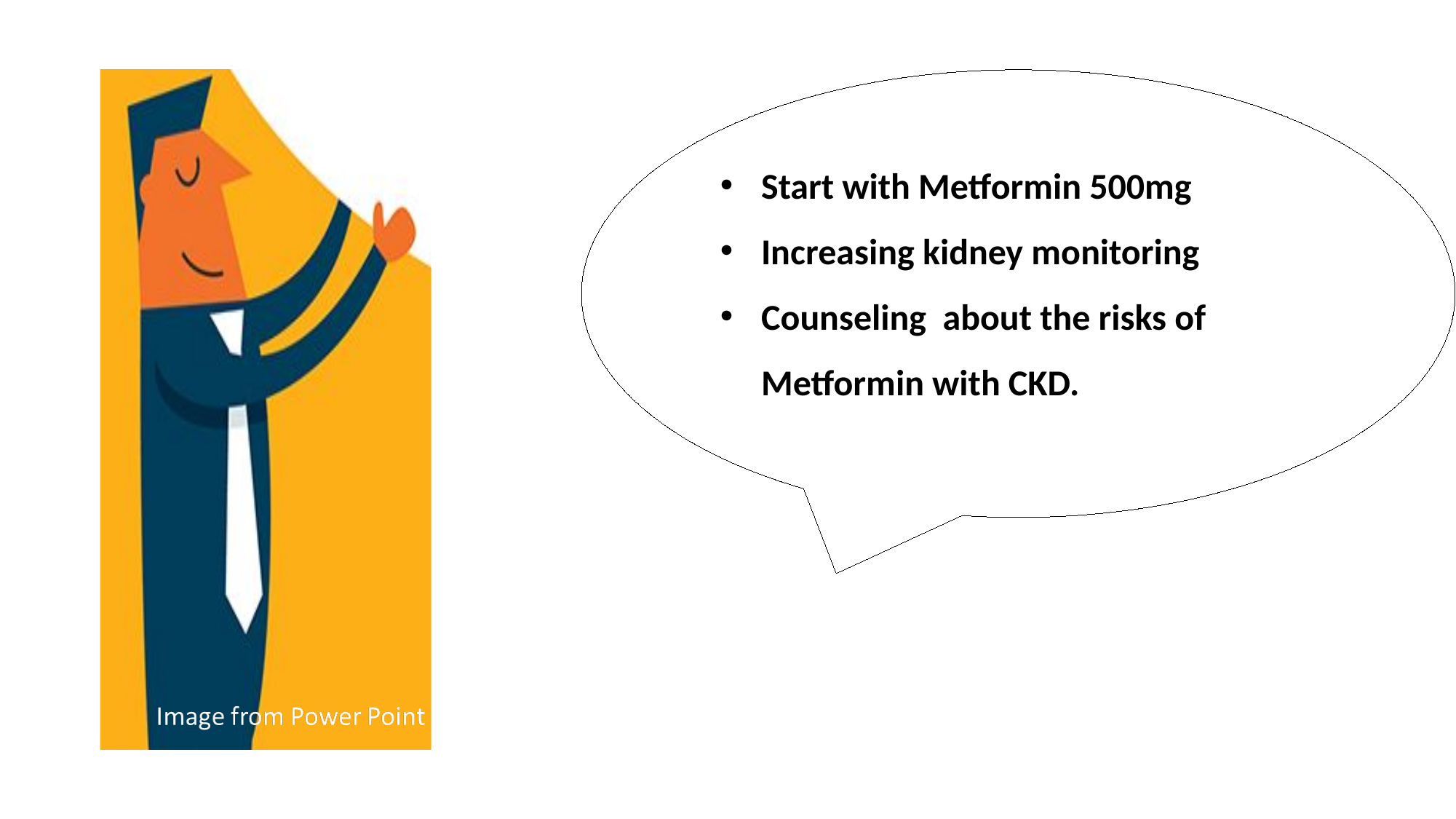

Start with Metformin 500mg
Increasing kidney monitoring
Counseling about the risks of Metformin with CKD.

## Slide 18
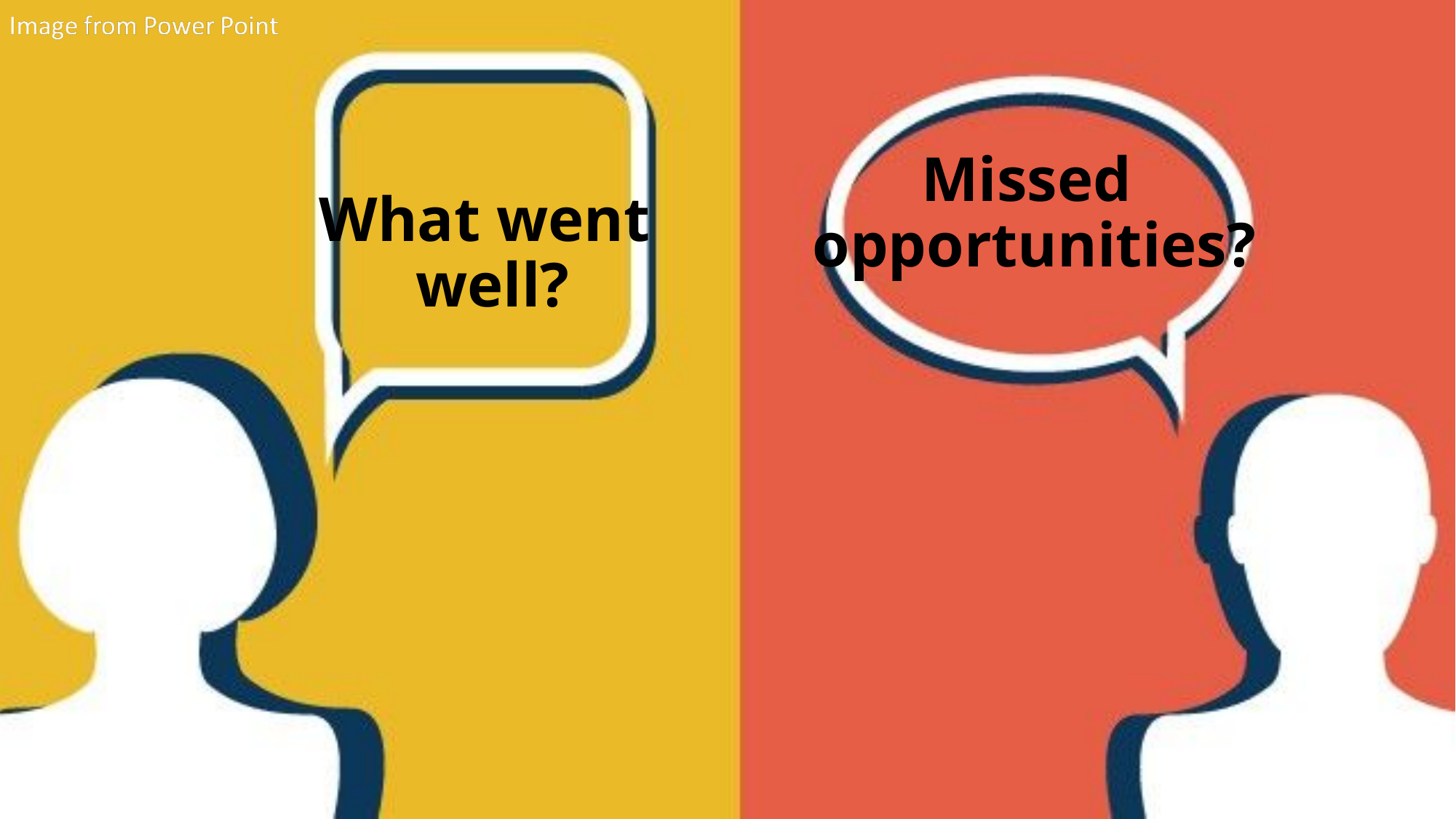

Missed
opportunities?
What went
well?
# Traditional Precepting Reflection

## Slide 19
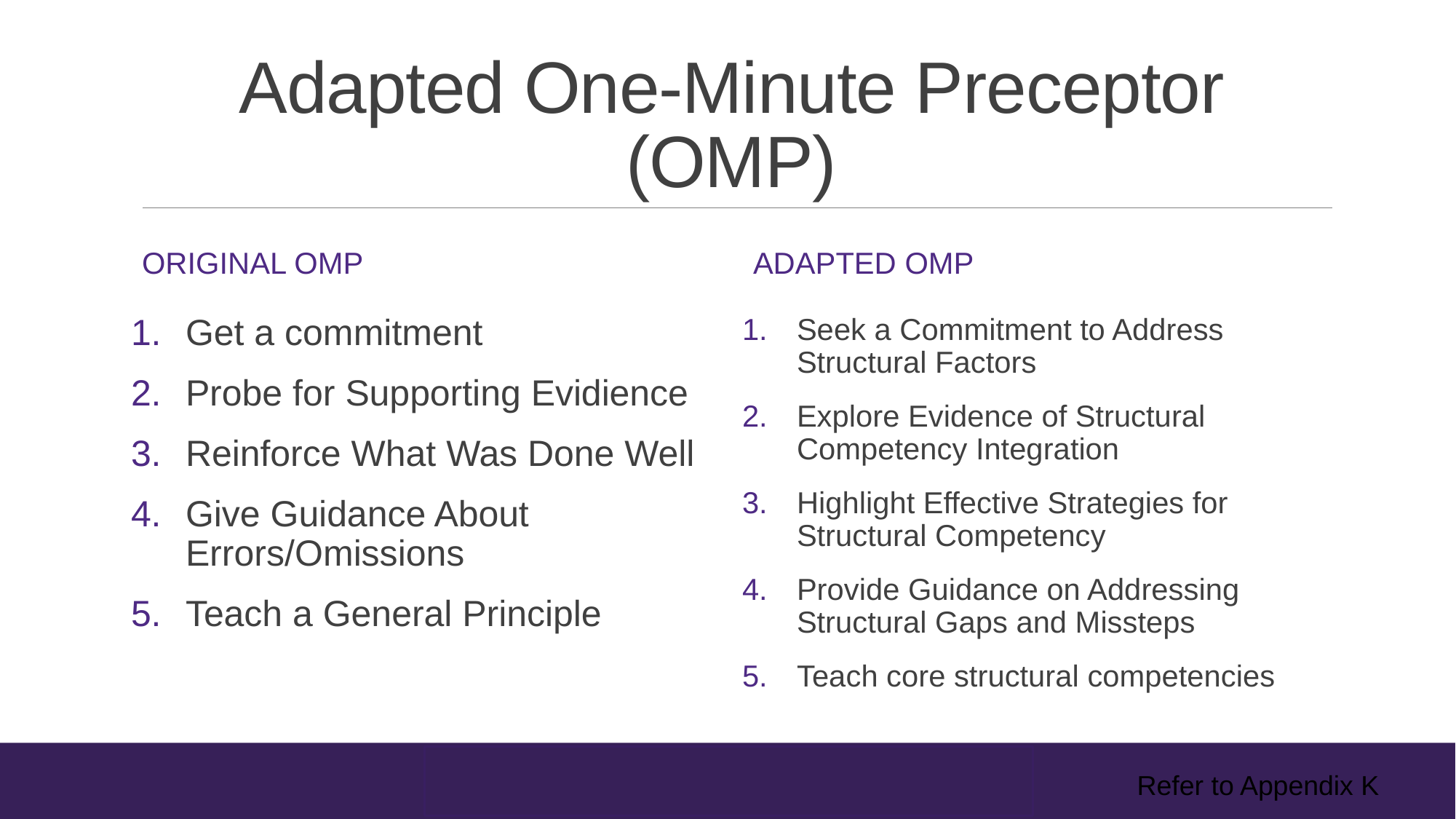

# Adapted One-Minute Preceptor (OMP)
Original omp
Adapted omp
Get a commitment
Probe for Supporting Evidience
Reinforce What Was Done Well
Give Guidance About Errors/Omissions
Teach a General Principle
Seek a Commitment to Address Structural Factors
Explore Evidence of Structural Competency Integration
Highlight Effective Strategies for Structural Competency
Provide Guidance on Addressing Structural Gaps and Missteps
Teach core structural competencies
Refer to Appendix K

## Slide 20
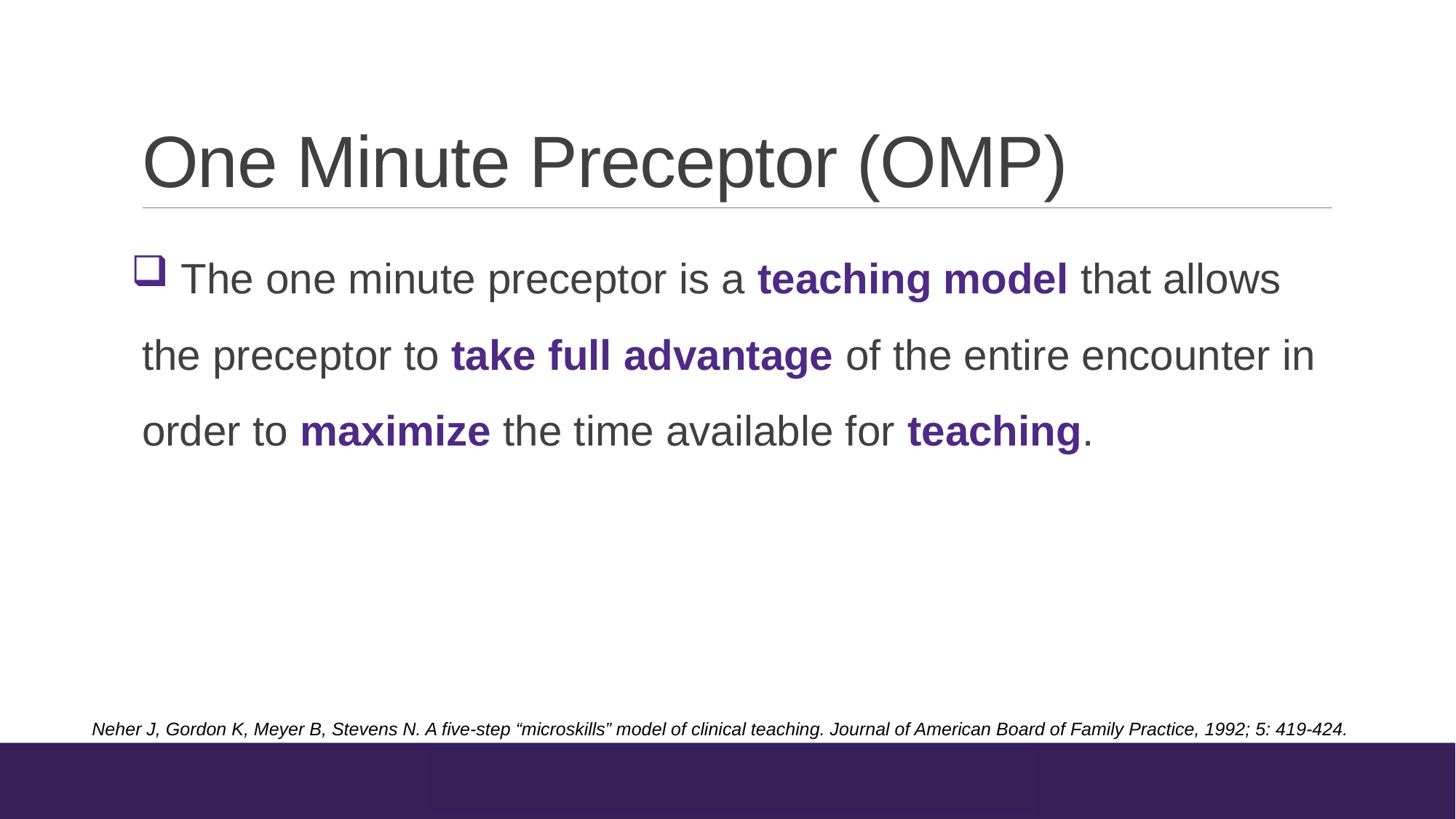

# One Minute Preceptor (OMP)
 The one minute preceptor is a teaching model that allows the preceptor to take full advantage of the entire encounter in order to maximize the time available for teaching.
Neher J, Gordon K, Meyer B, Stevens N. A five-step “microskills” model of clinical teaching. Journal of American Board of Family Practice, 1992; 5: 419-424.

## Slide 21
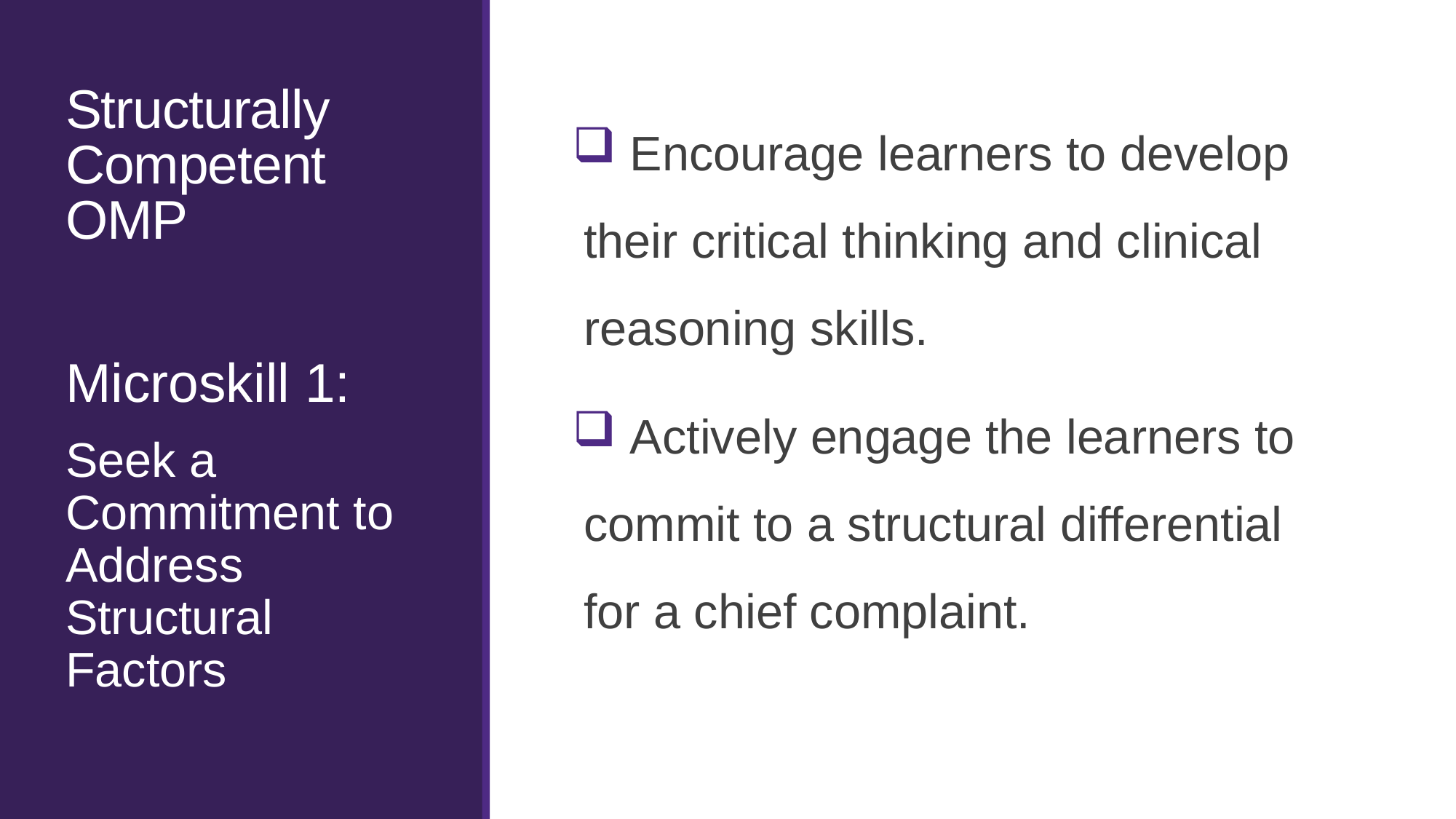

# Structurally Competent OMP
 Encourage learners to develop their critical thinking and clinical reasoning skills.
 Actively engage the learners to commit to a structural differential for a chief complaint.
Microskill 1:
Seek a Commitment to Address Structural Factors

## Slide 22
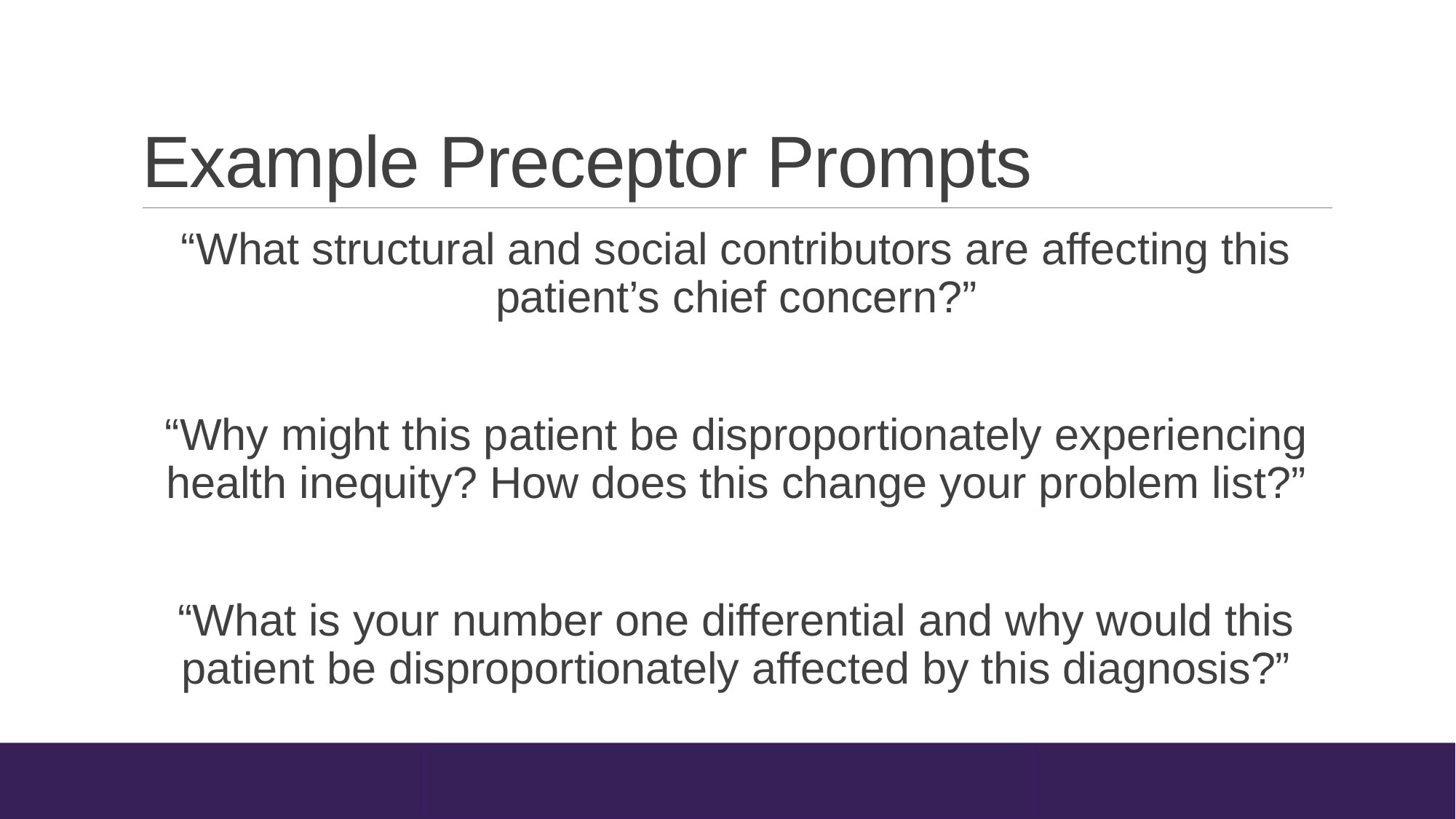

# Example Preceptor Prompts
“What structural and social contributors are affecting this patient’s chief concern?”
“Why might this patient be disproportionately experiencing health inequity? How does this change your problem list?”
“What is your number one differential and why would this patient be disproportionately affected by this diagnosis?”

## Slide 23
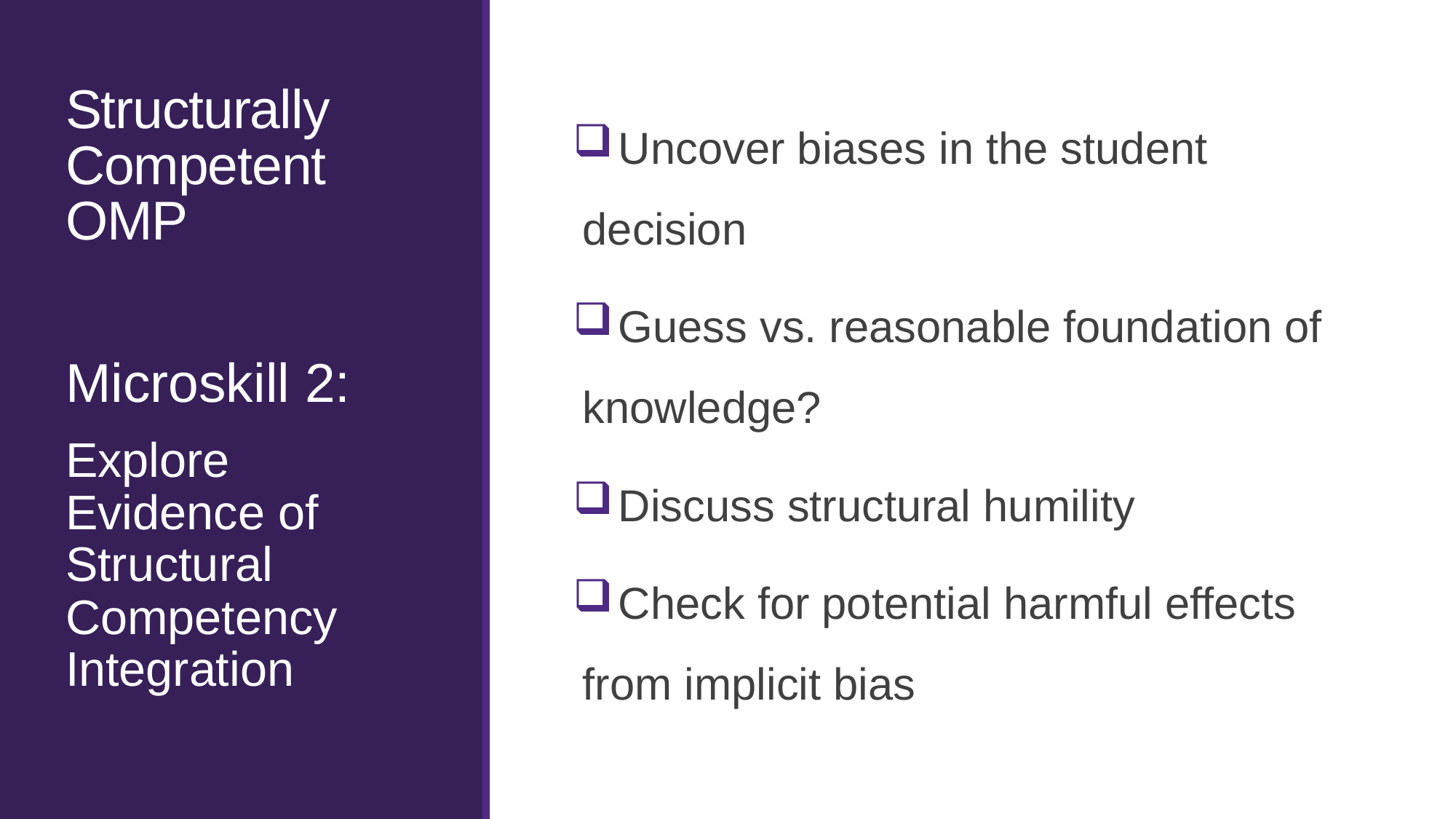

# Structurally Competent OMP
 Uncover biases in the student decision
 Guess vs. reasonable foundation of knowledge?
 Discuss structural humility
 Check for potential harmful effects from implicit bias
Microskill 2:
Explore Evidence of Structural Competency Integration

## Slide 24
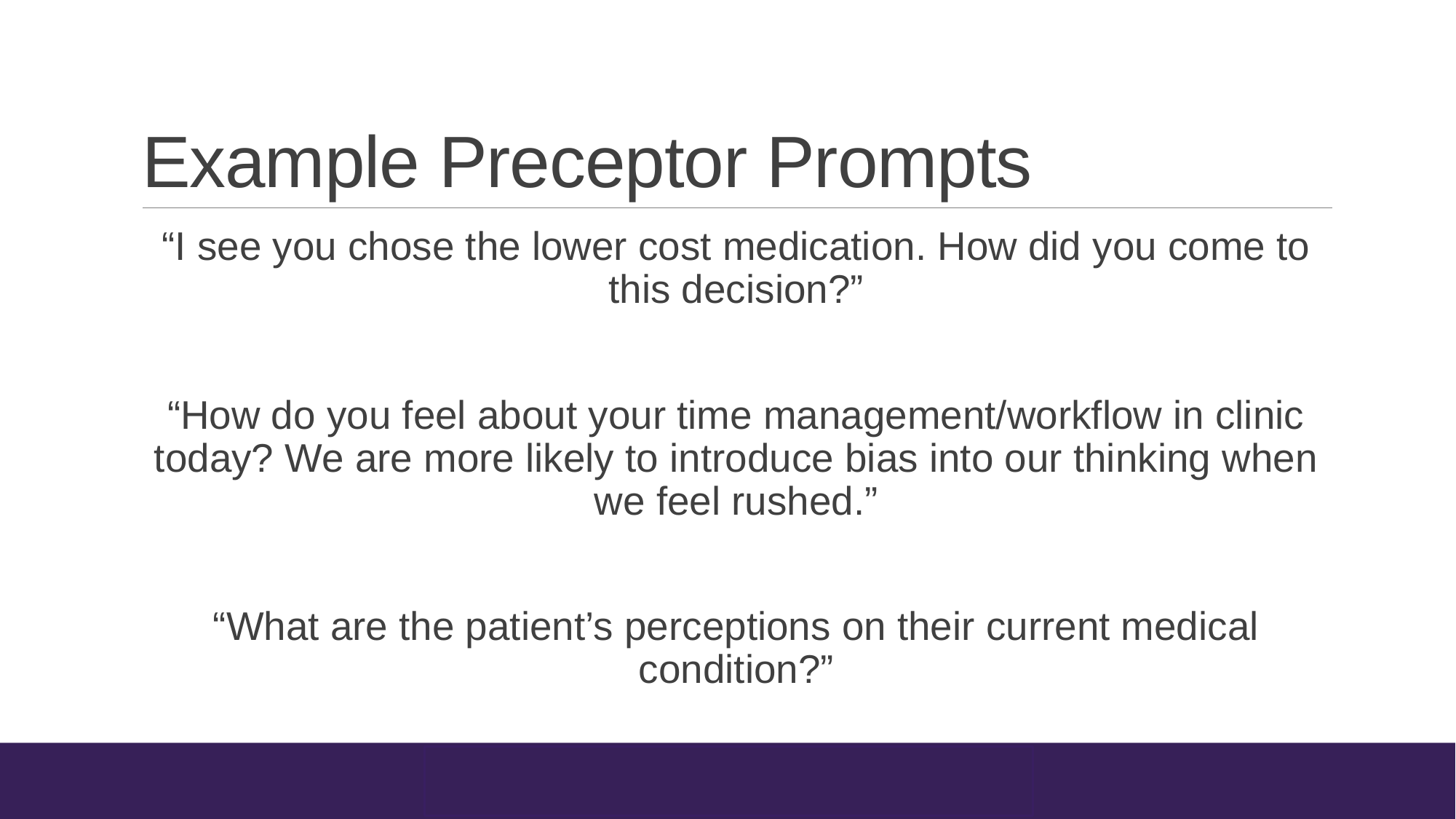

# Example Preceptor Prompts
“I see you chose the lower cost medication. How did you come to this decision?”
“How do you feel about your time management/workflow in clinic today? We are more likely to introduce bias into our thinking when we feel rushed.”
“What are the patient’s perceptions on their current medical condition?”

## Slide 25
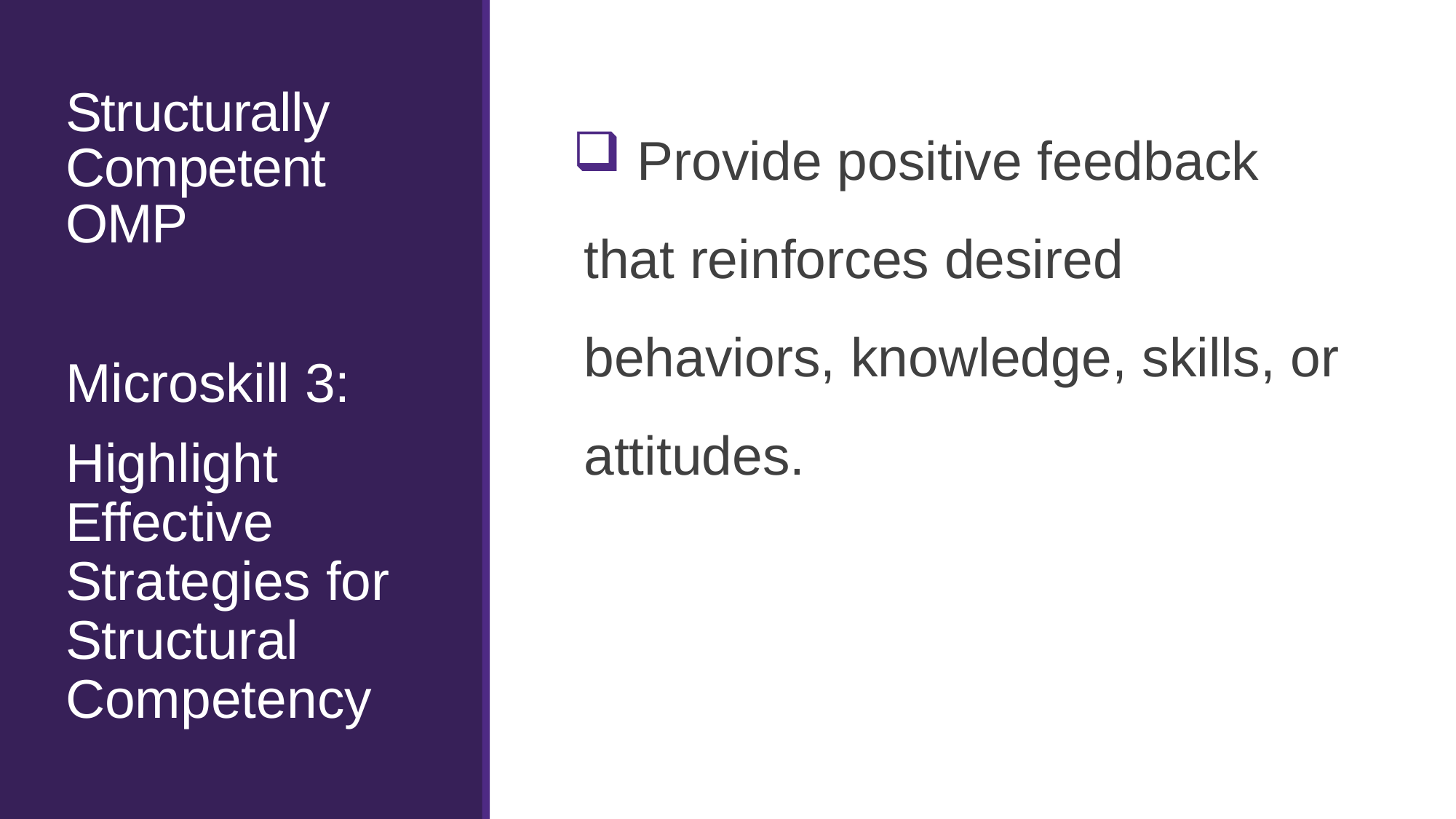

# Structurally Competent OMP
 Provide positive feedback that reinforces desired behaviors, knowledge, skills, or attitudes.
Microskill 3:
Highlight Effective Strategies for Structural Competency

## Slide 26
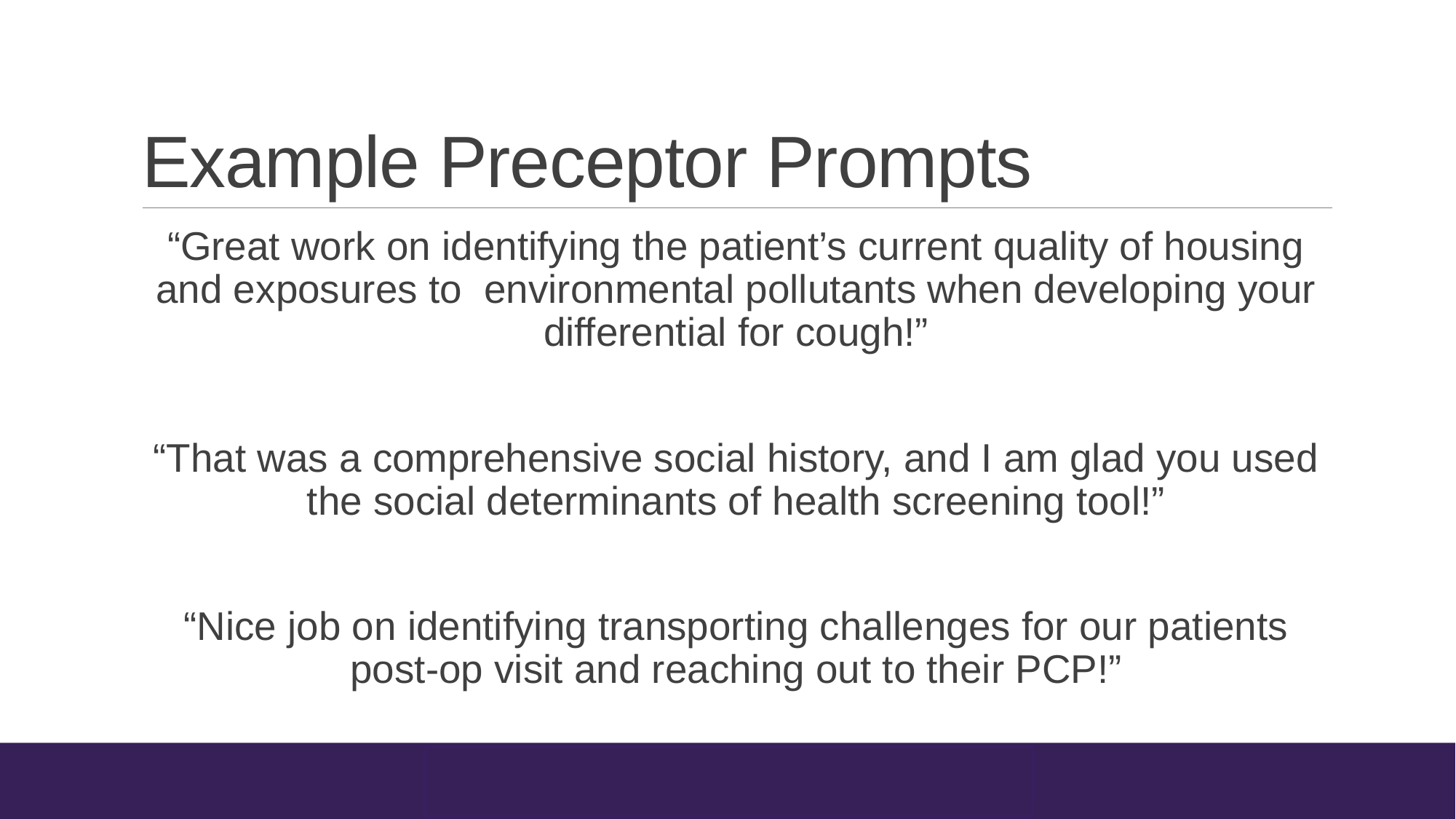

# Example Preceptor Prompts
“Great work on identifying the patient’s current quality of housing and exposures to environmental pollutants when developing your differential for cough!”
“That was a comprehensive social history, and I am glad you used the social determinants of health screening tool!”
“Nice job on identifying transporting challenges for our patients post-op visit and reaching out to their PCP!”

## Slide 27
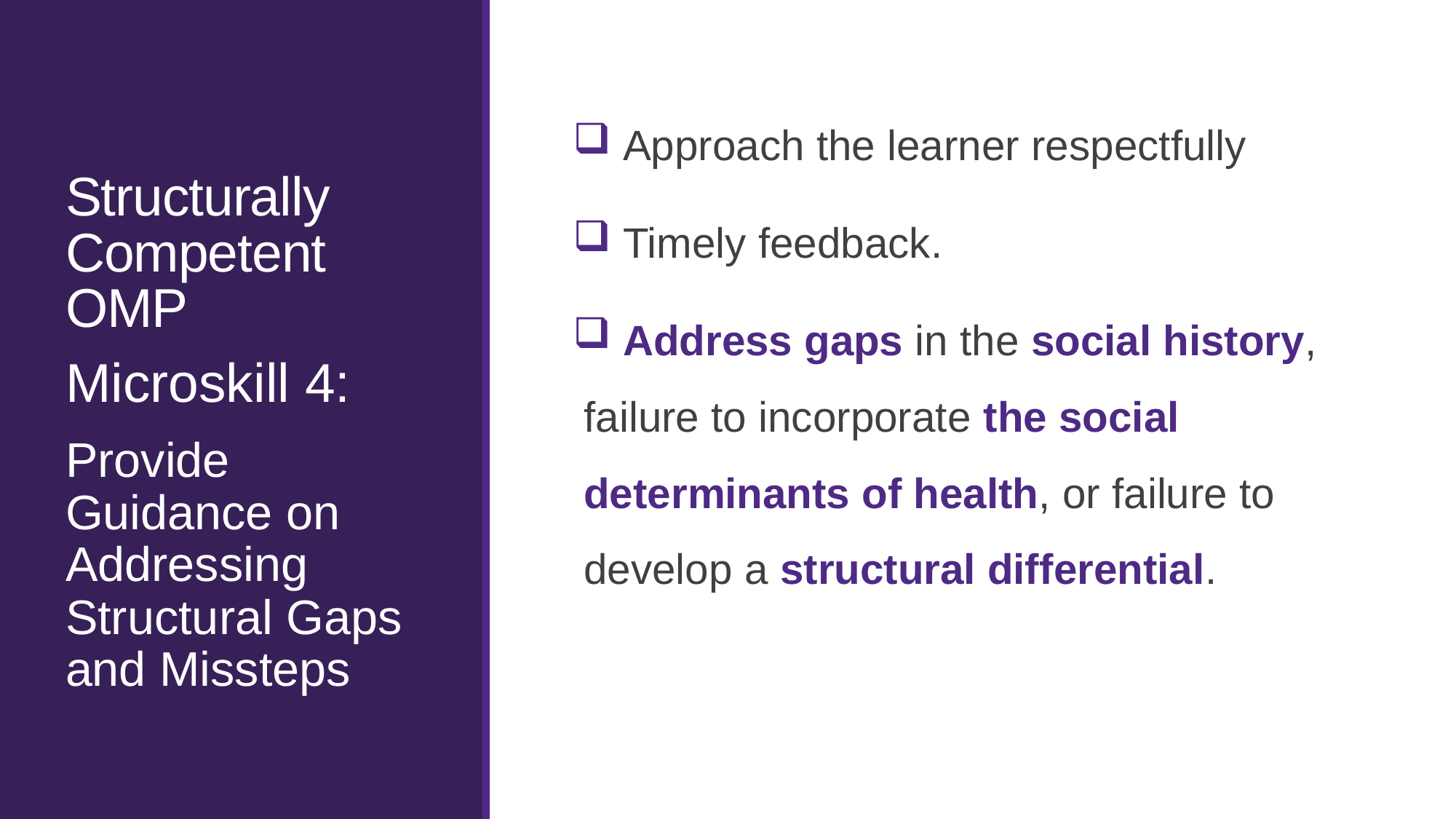

# Structurally Competent OMP
 Approach the learner respectfully
 Timely feedback.
 Address gaps in the social history, failure to incorporate the social determinants of health, or failure to develop a structural differential.
Microskill 4:
Provide Guidance on Addressing Structural Gaps and Missteps

## Slide 28
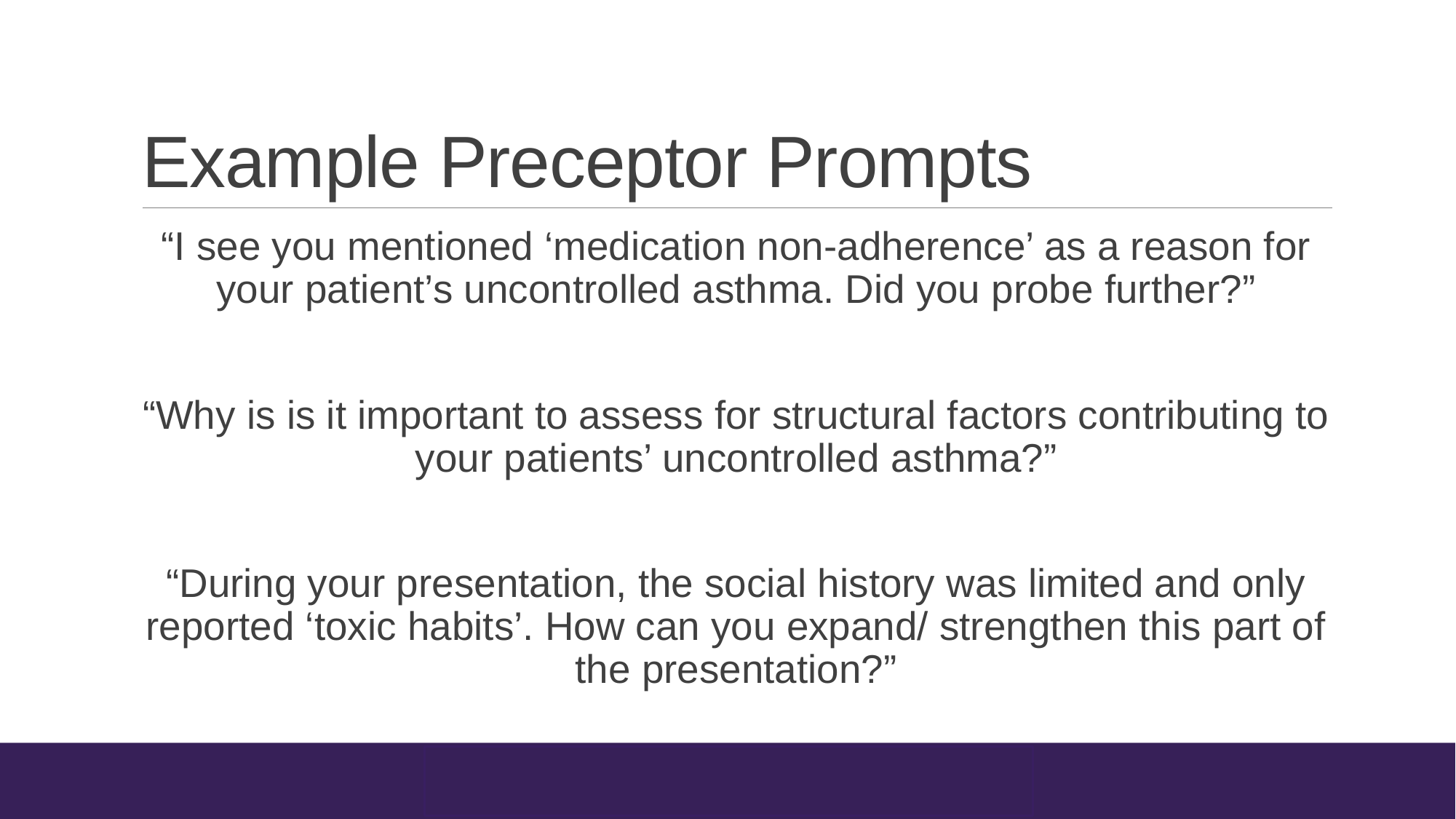

# Example Preceptor Prompts
“I see you mentioned ‘medication non-adherence’ as a reason for your patient’s uncontrolled asthma. Did you probe further?”
“Why is is it important to assess for structural factors contributing to your patients’ uncontrolled asthma?”
“During your presentation, the social history was limited and only reported ‘toxic habits’. How can you expand/ strengthen this part of the presentation?”

## Slide 29
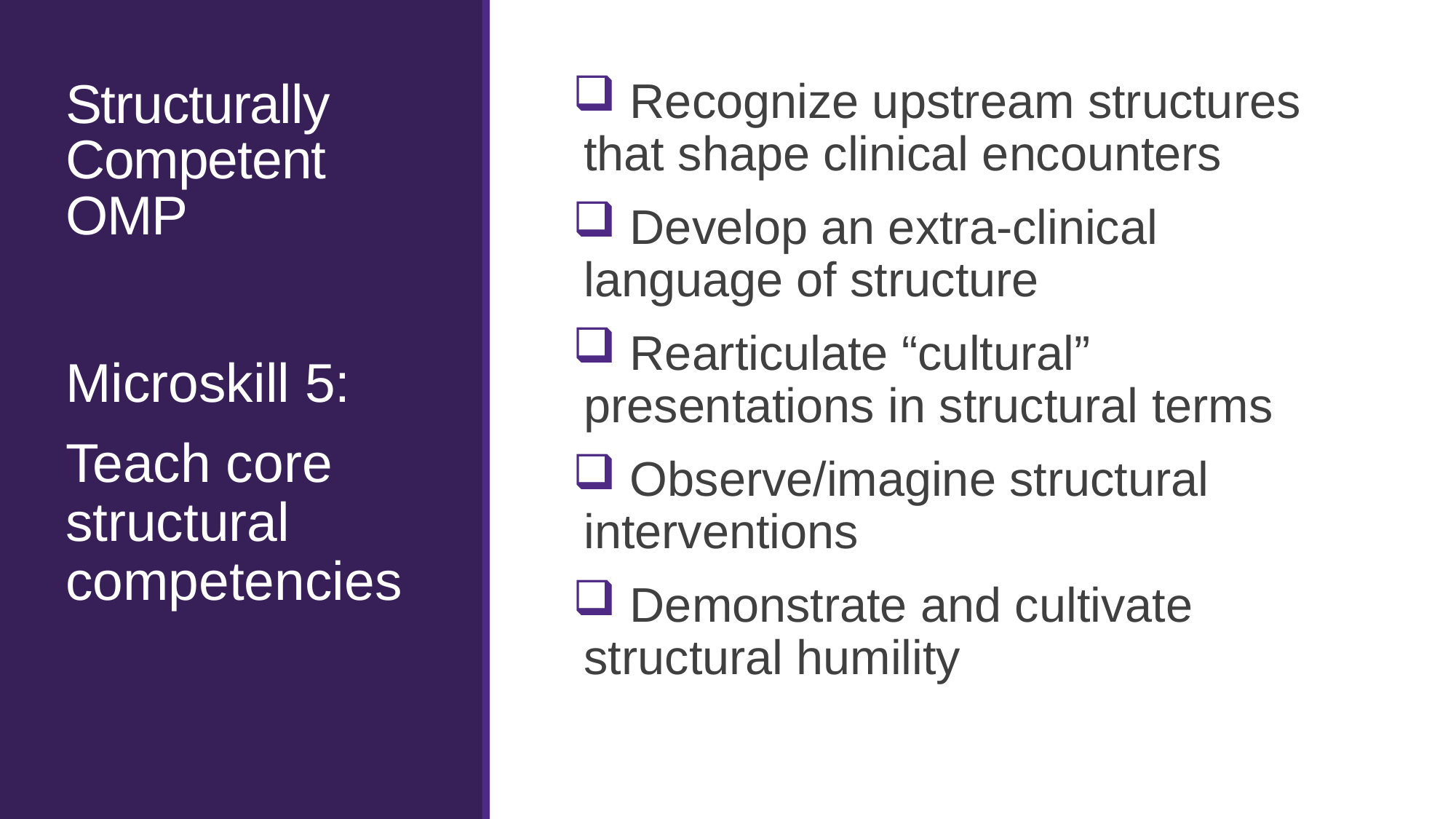

# Structurally Competent OMP
 Recognize upstream structures that shape clinical encounters
 Develop an extra-clinical language of structure
 Rearticulate “cultural” presentations in structural terms
 Observe/imagine structural interventions
 Demonstrate and cultivate structural humility
Microskill 5:
Teach core structural competencies

## Slide 30
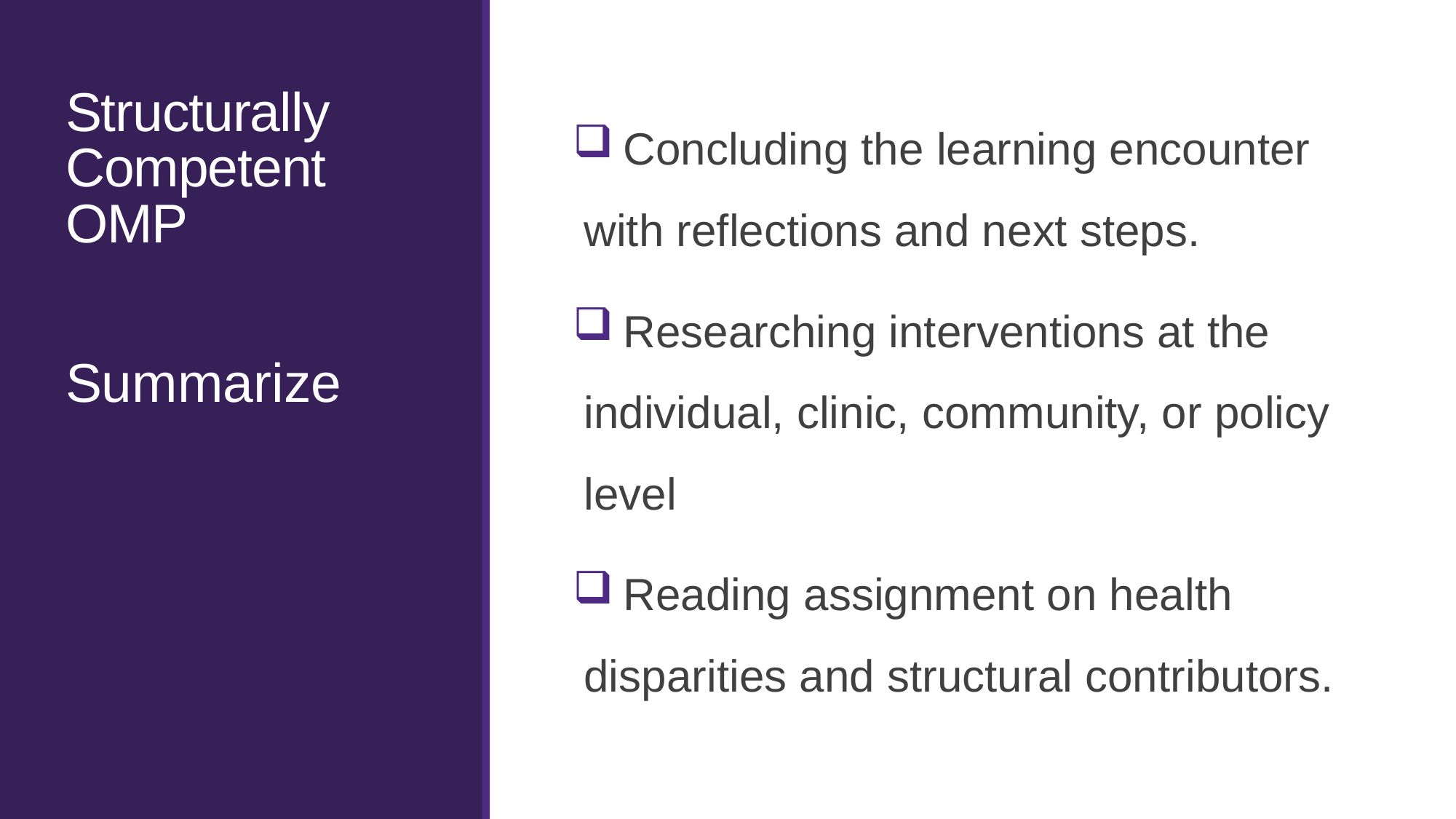

# Structurally Competent OMP
 Concluding the learning encounter with reflections and next steps.
 Researching interventions at the individual, clinic, community, or policy level
 Reading assignment on health disparities and structural contributors.
Summarize

## Slide 31
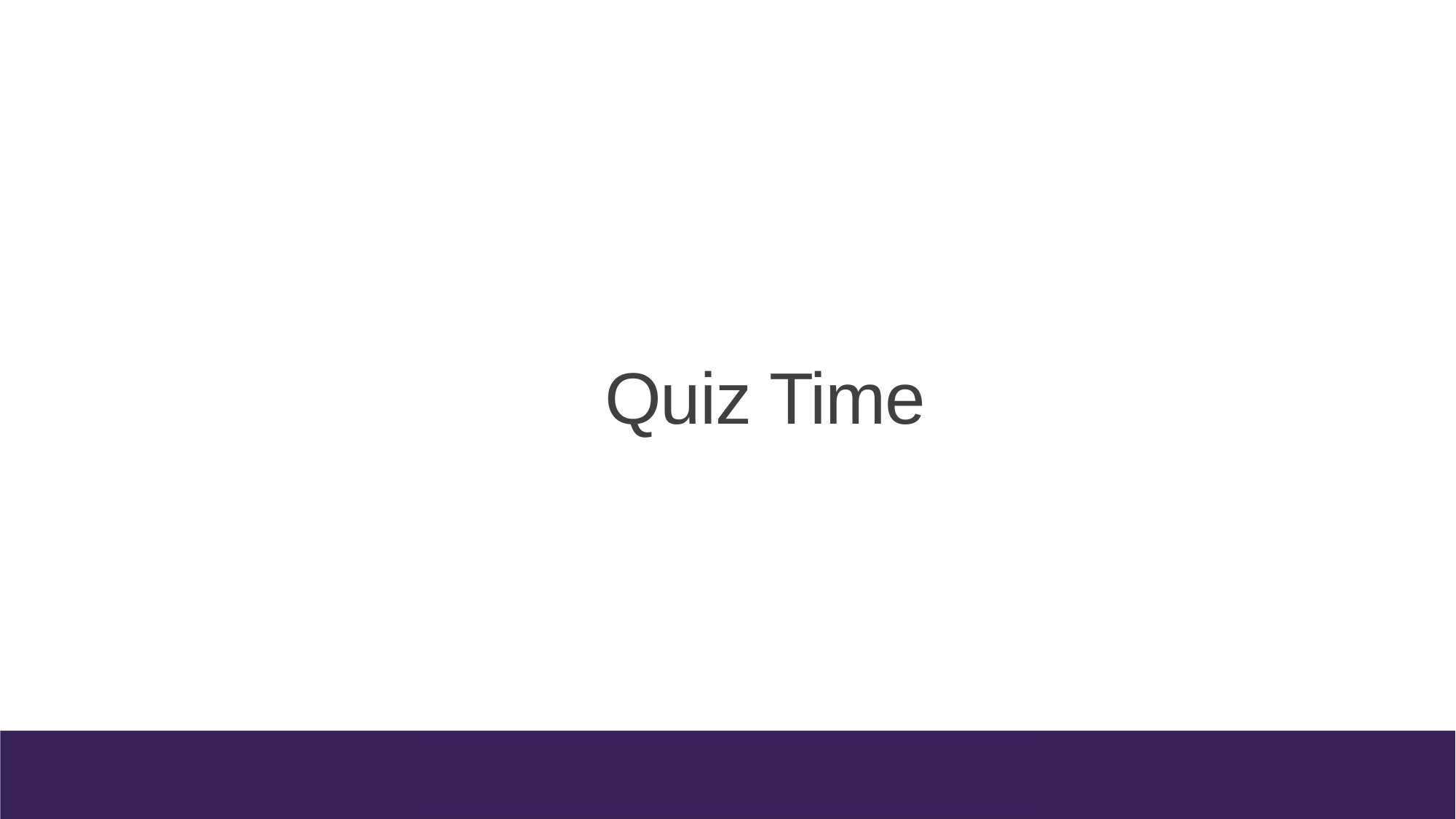

Quiz Time

## Slide 32
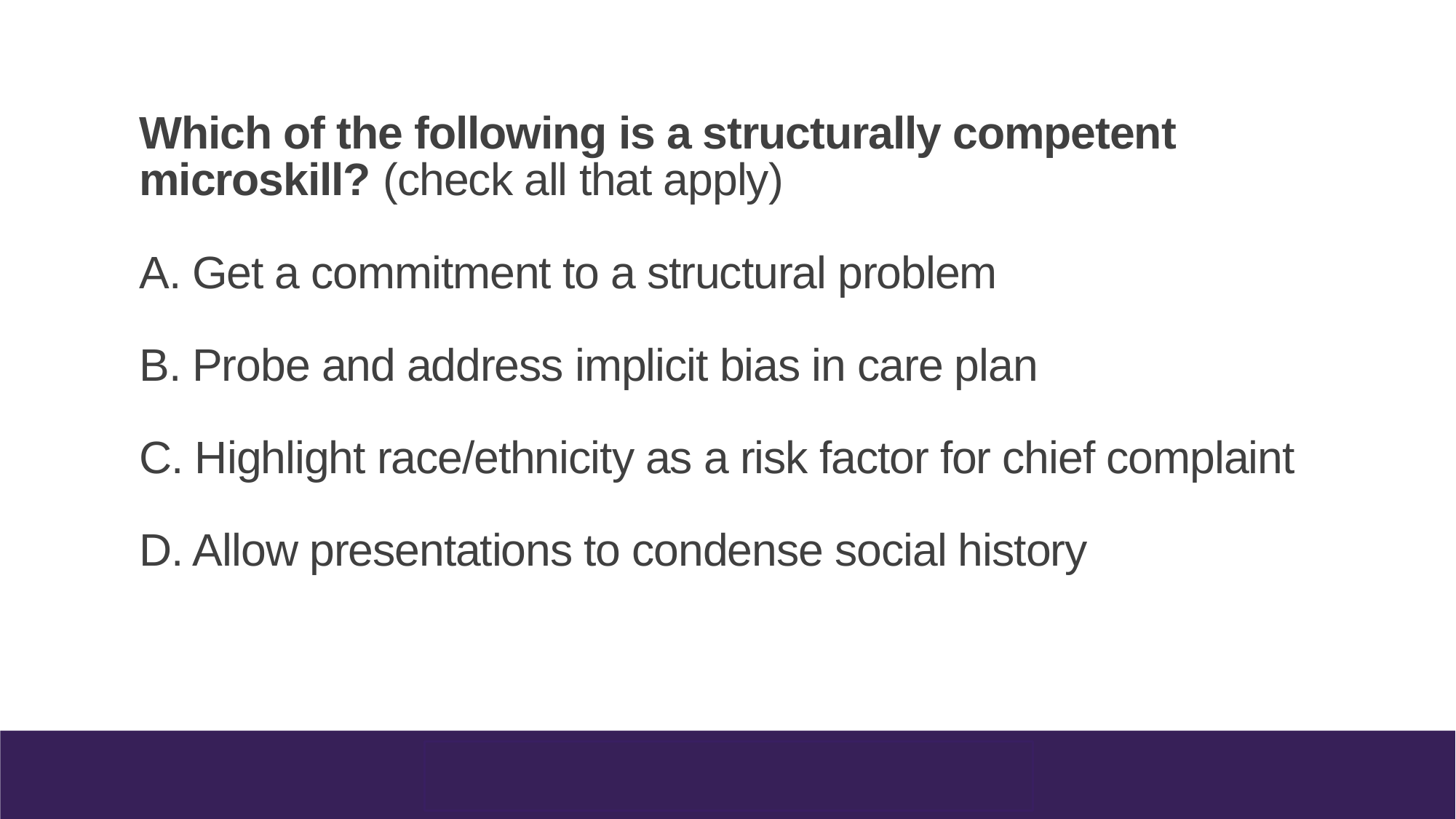

Which of the following is a structurally competent microskill? (check all that apply)
A. Get a commitment to a structural problem
B. Probe and address implicit bias in care plan
C. Highlight race/ethnicity as a risk factor for chief complaint
D. Allow presentations to condense social history

## Slide 33
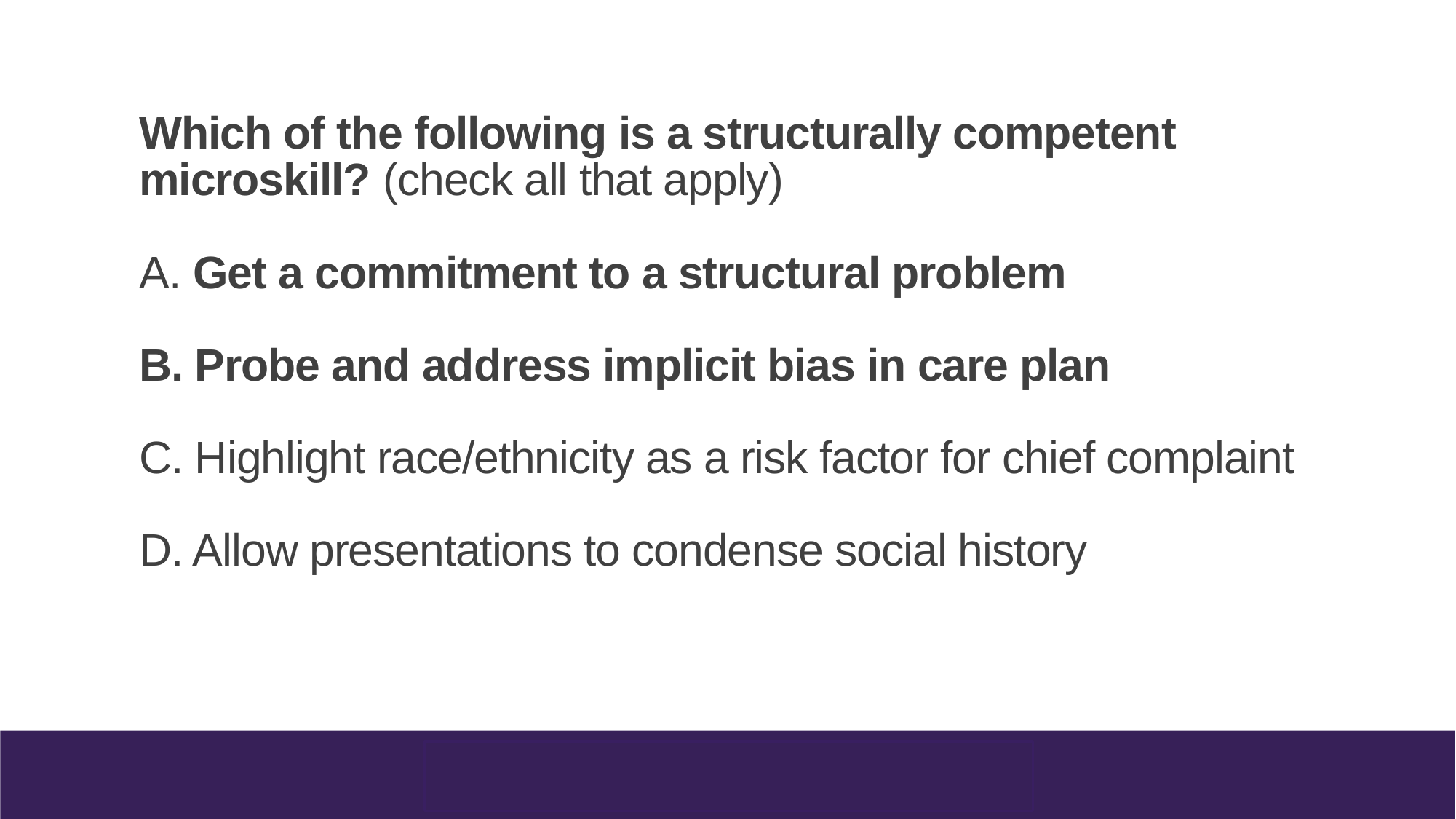

Which of the following is a structurally competent microskill? (check all that apply)
A. Get a commitment to a structural problem
B. Probe and address implicit bias in care plan
C. Highlight race/ethnicity as a risk factor for chief complaint
D. Allow presentations to condense social history

## Slide 34
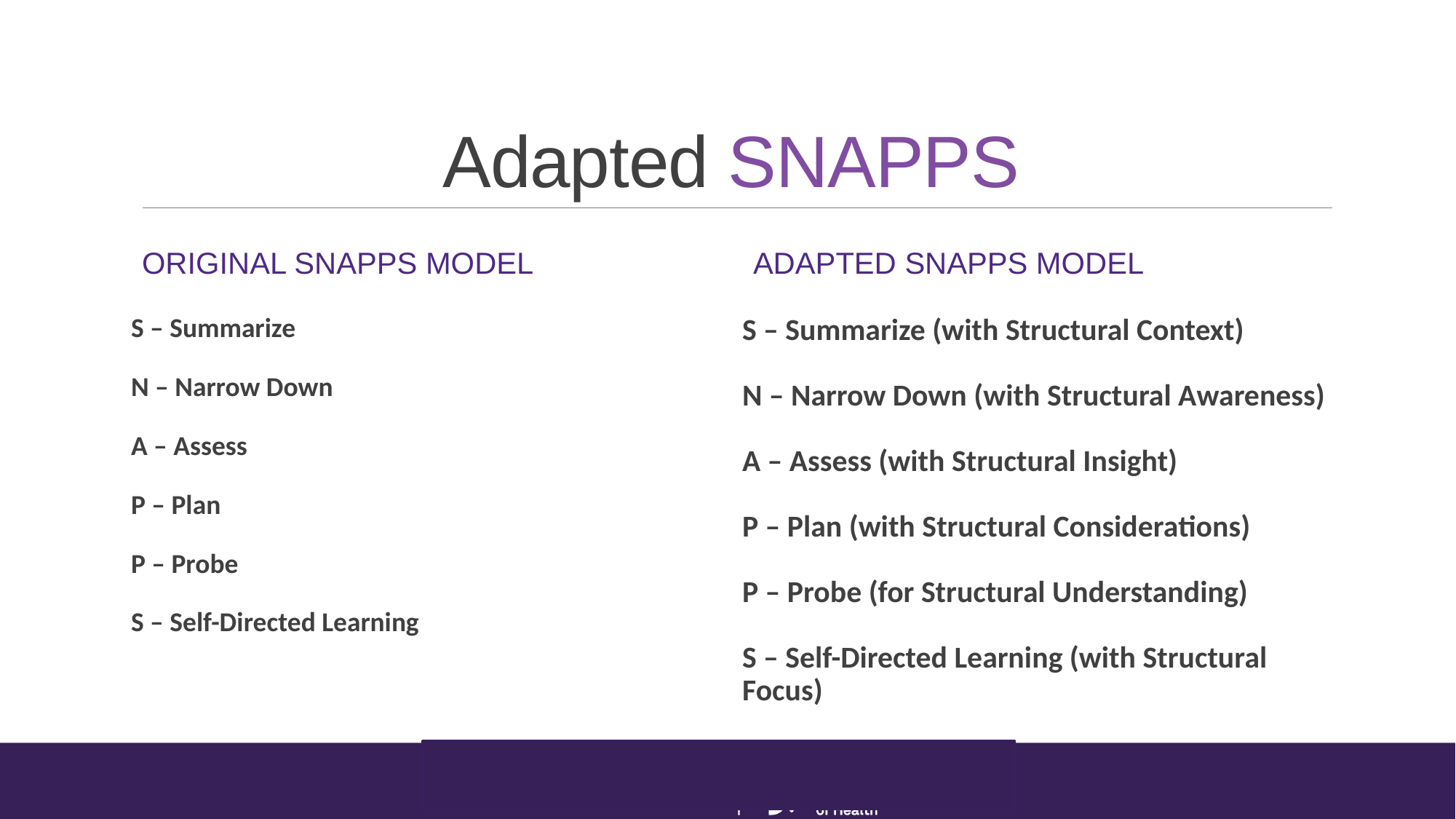

# Adapted SNAPPS
ORIGINAL SNAPPS MODEL
ADAPTED SNAPPS MODEL
S – Summarize
N – Narrow Down
A – Assess
P – Plan
P – Probe
S – Self-Directed Learning
S – Summarize (with Structural Context)
N – Narrow Down (with Structural Awareness)
A – Assess (with Structural Insight)
P – Plan (with Structural Considerations)
P – Probe (for Structural Understanding)
S – Self-Directed Learning (with Structural Focus)

## Slide 35
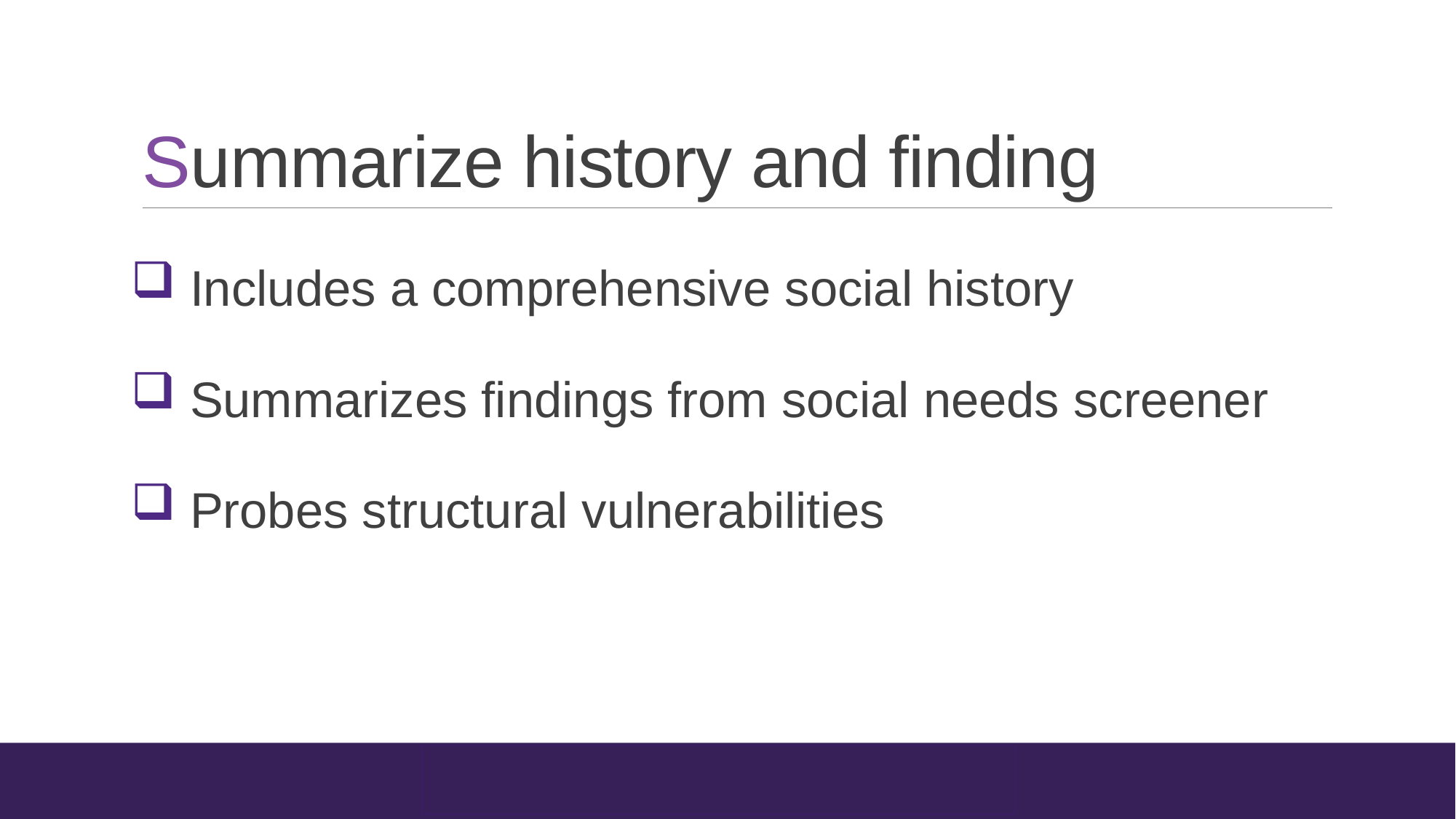

# Summarize history and finding
 Includes a comprehensive social history
 Summarizes findings from social needs screener
 Probes structural vulnerabilities

## Slide 36
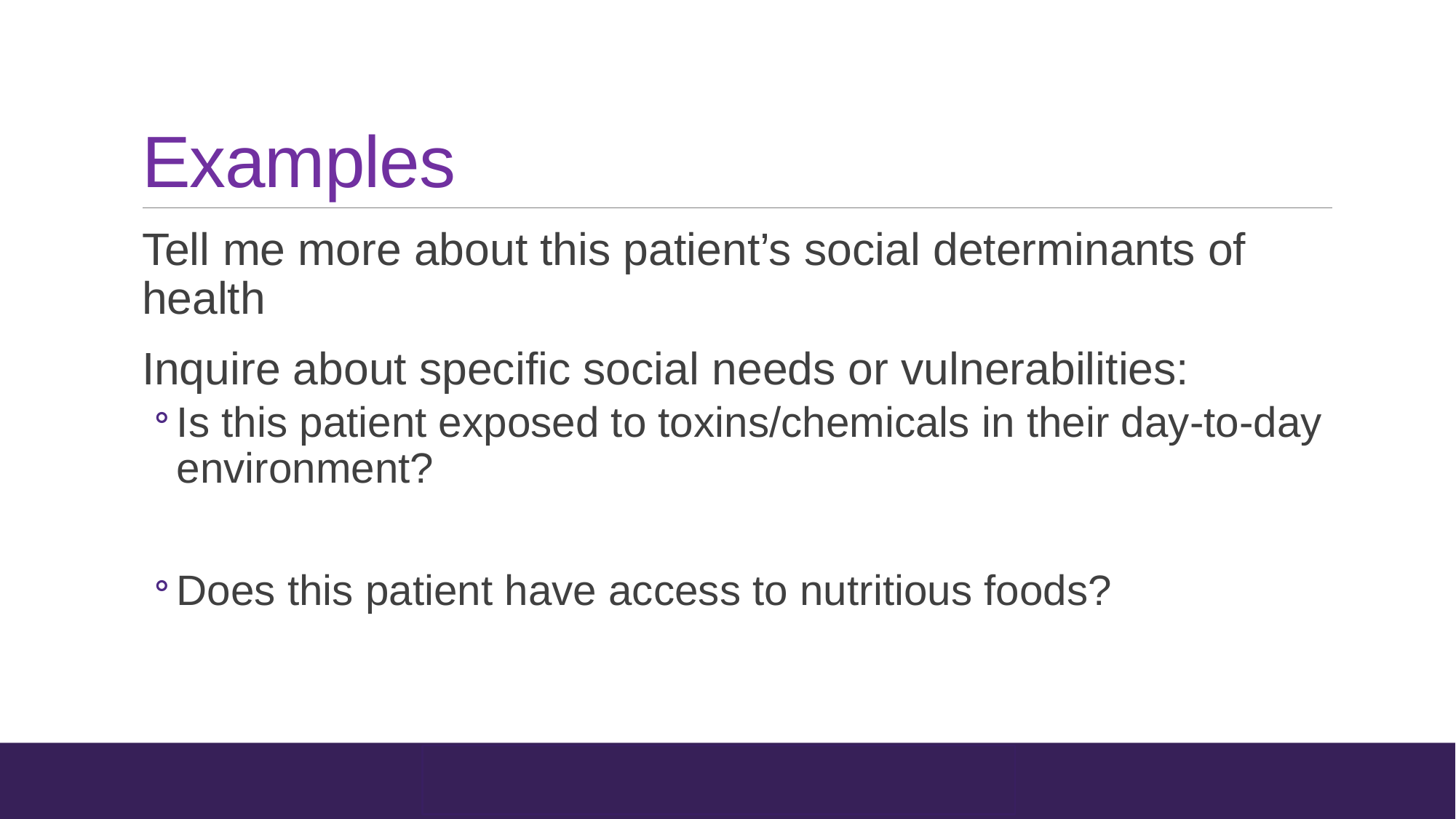

# Examples
Tell me more about this patient’s social determinants of health
Inquire about specific social needs or vulnerabilities:
Is this patient exposed to toxins/chemicals in their day-to-day environment?
Does this patient have access to nutritious foods?

## Slide 37
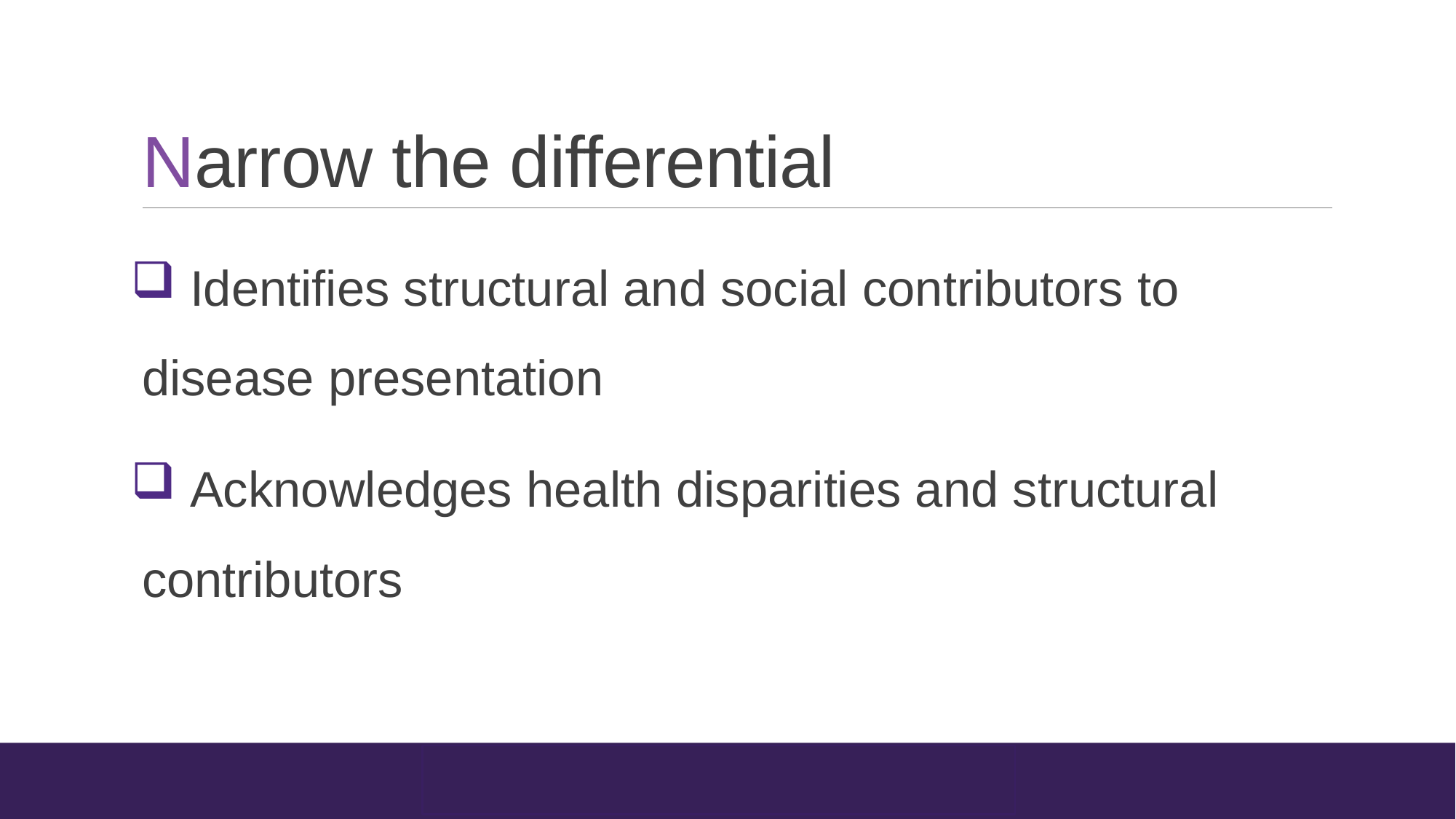

# Narrow the differential
 Identifies structural and social contributors to disease presentation
 Acknowledges health disparities and structural contributors

## Slide 38
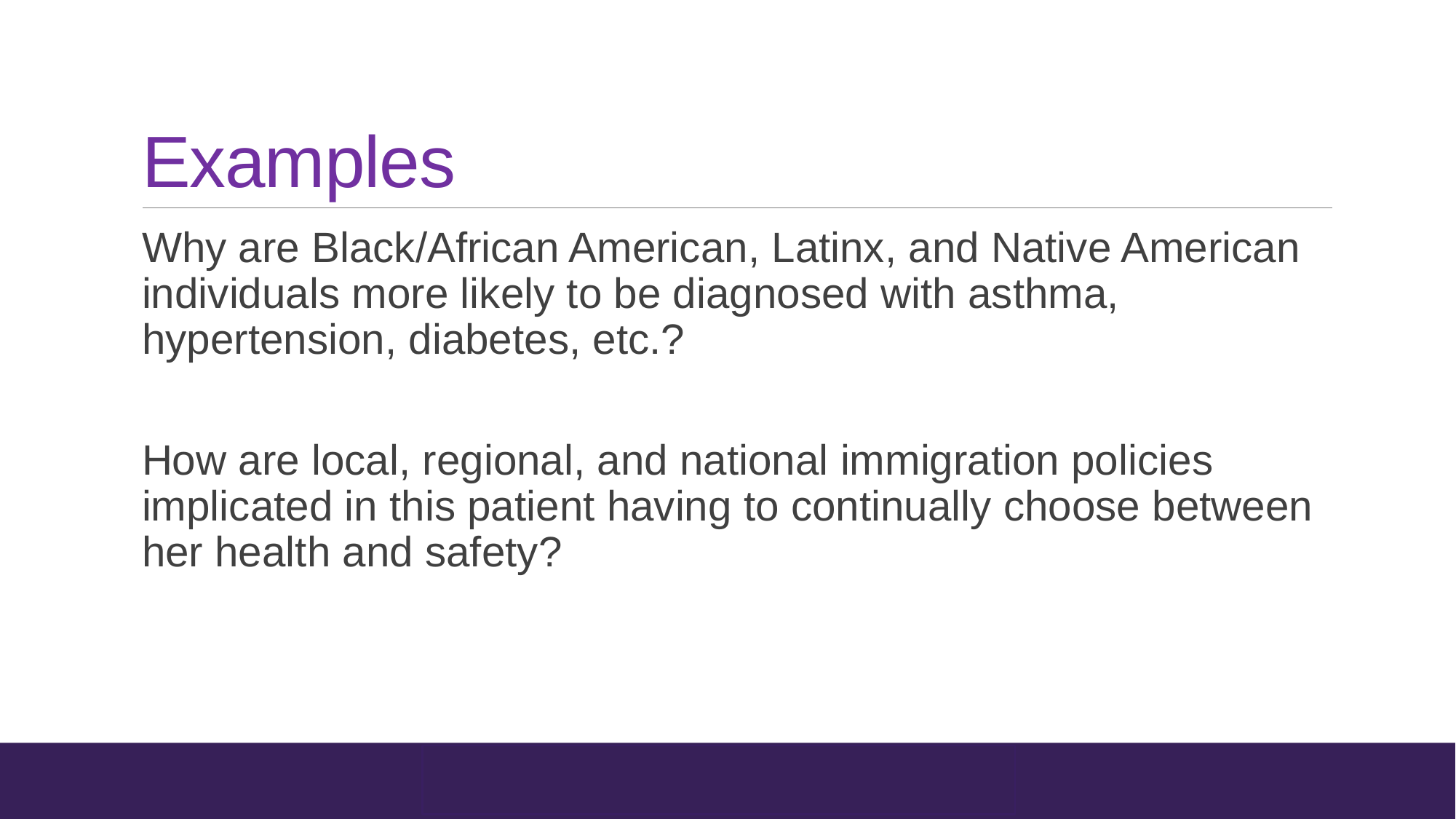

# Examples
Why are Black/African American, Latinx, and Native American individuals more likely to be diagnosed with asthma, hypertension, diabetes, etc.?
How are local, regional, and national immigration policies implicated in this patient having to continually choose between her health and safety?

## Slide 39
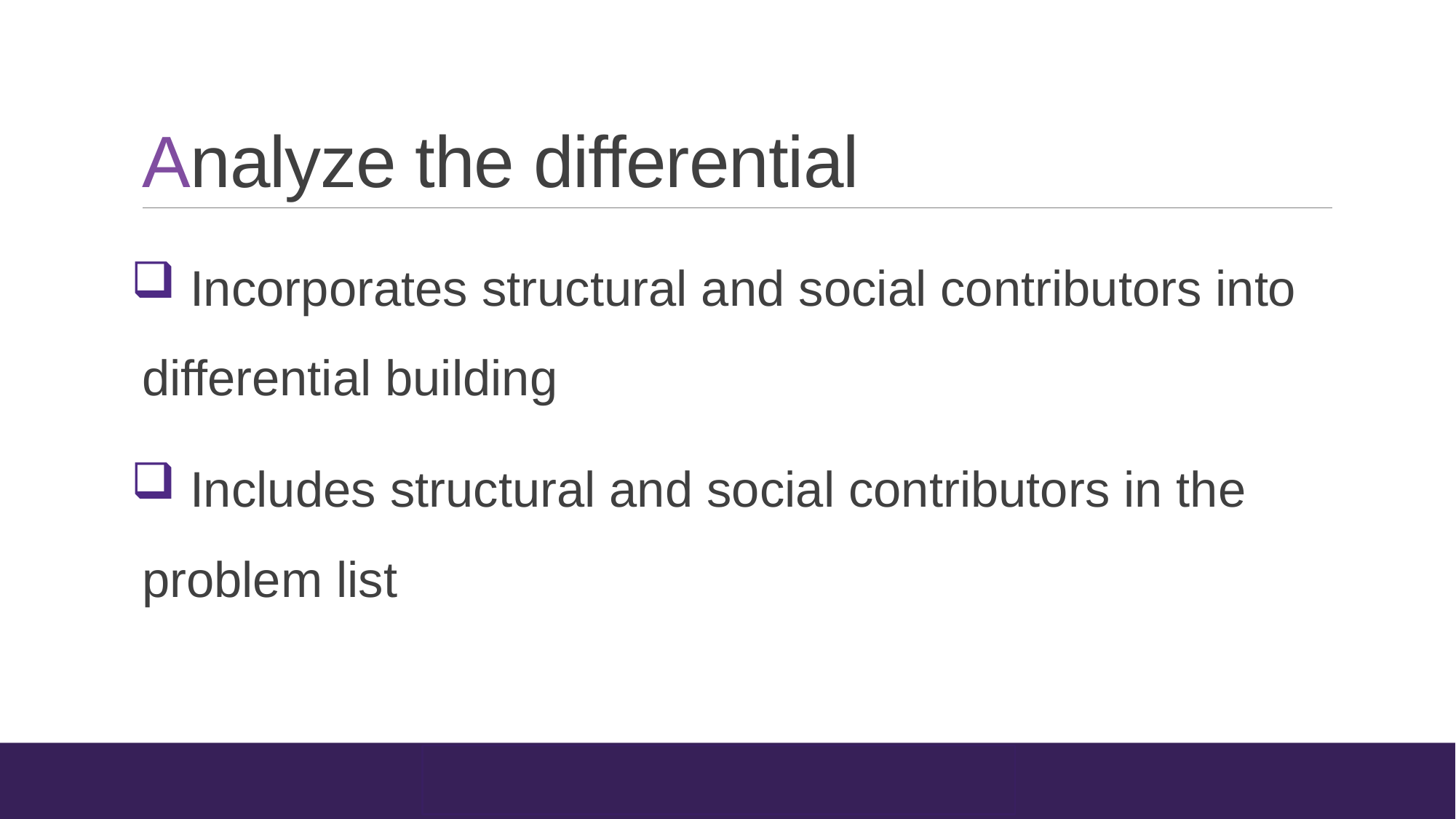

# Analyze the differential
 Incorporates structural and social contributors into differential building
 Includes structural and social contributors in the problem list

## Slide 40
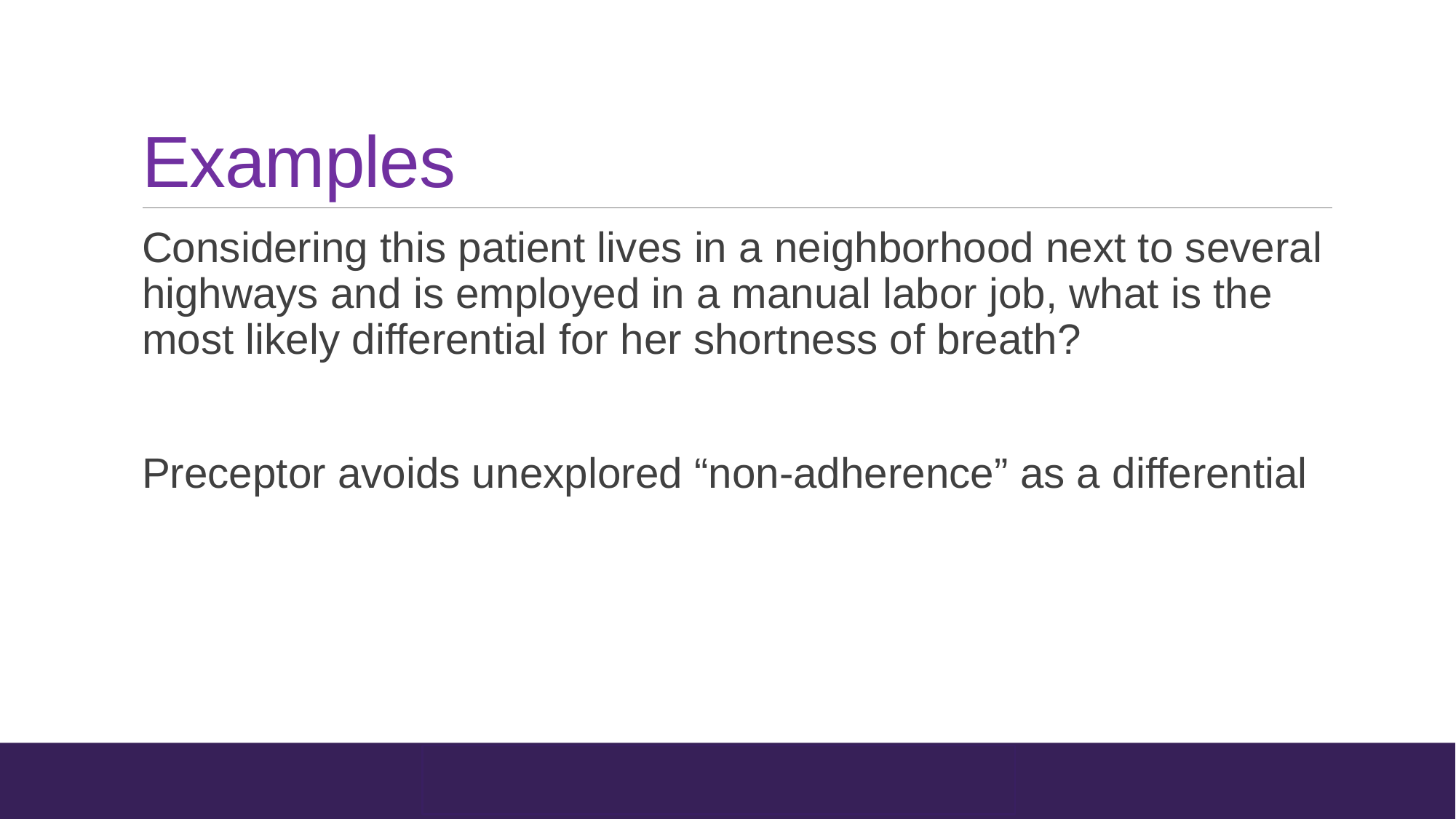

# Examples
Considering this patient lives in a neighborhood next to several highways and is employed in a manual labor job, what is the most likely differential for her shortness of breath?
Preceptor avoids unexplored “non-adherence” as a differential

## Slide 41
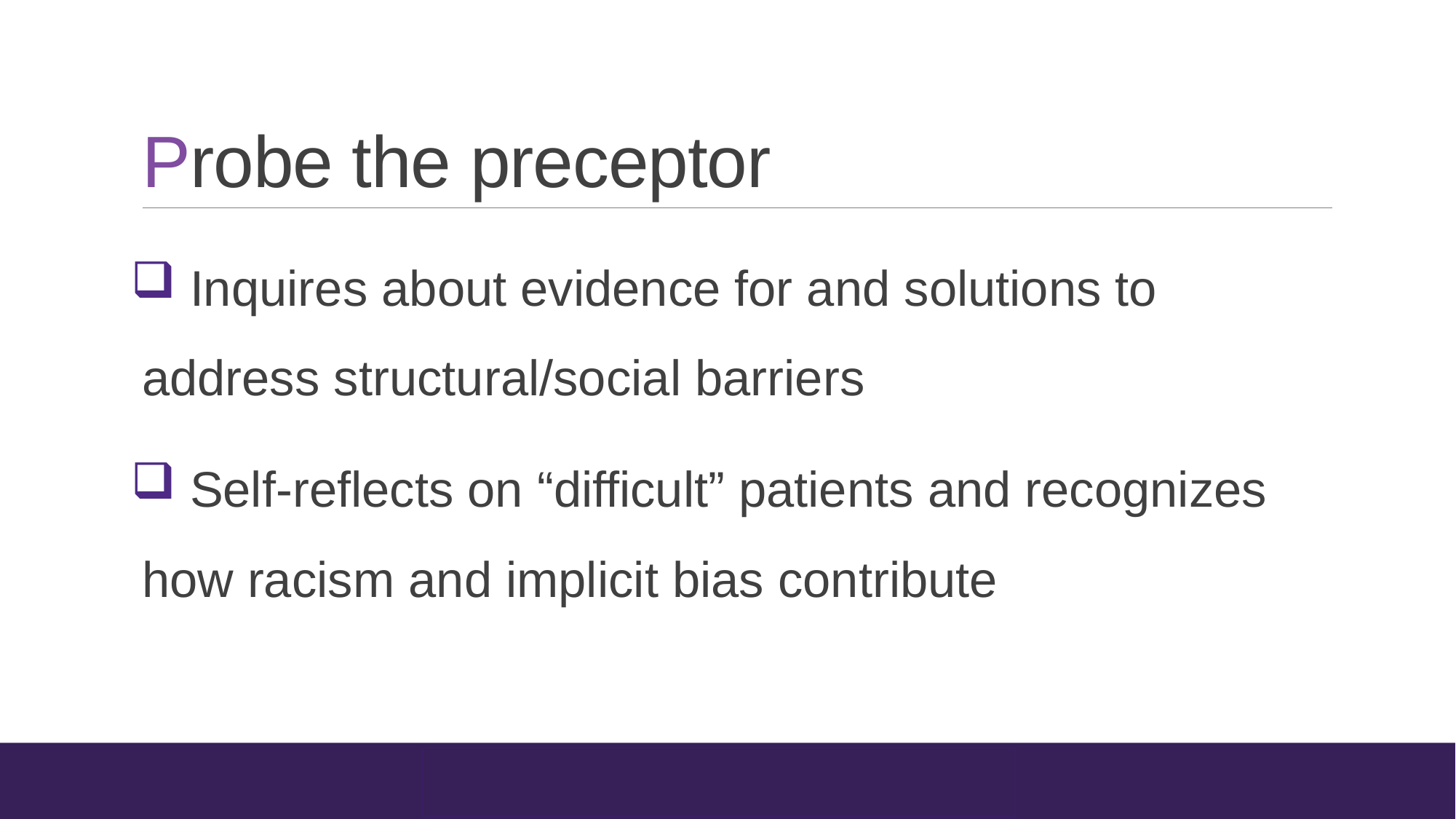

# Probe the preceptor
 Inquires about evidence for and solutions to address structural/social barriers
 Self-reflects on “difficult” patients and recognizes how racism and implicit bias contribute

## Slide 42
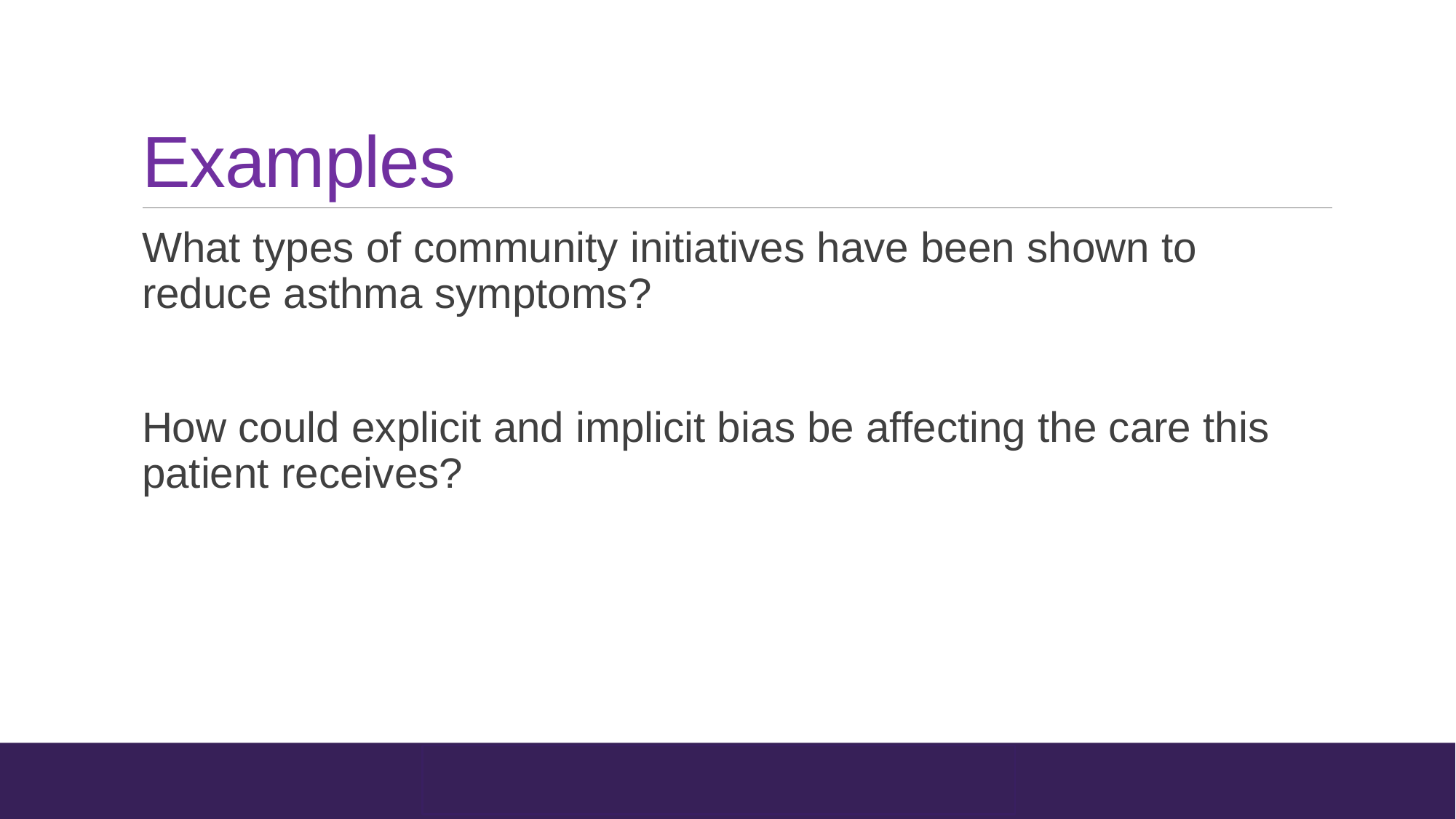

# Examples
What types of community initiatives have been shown to reduce asthma symptoms?
How could explicit and implicit bias be affecting the care this patient receives?

## Slide 43
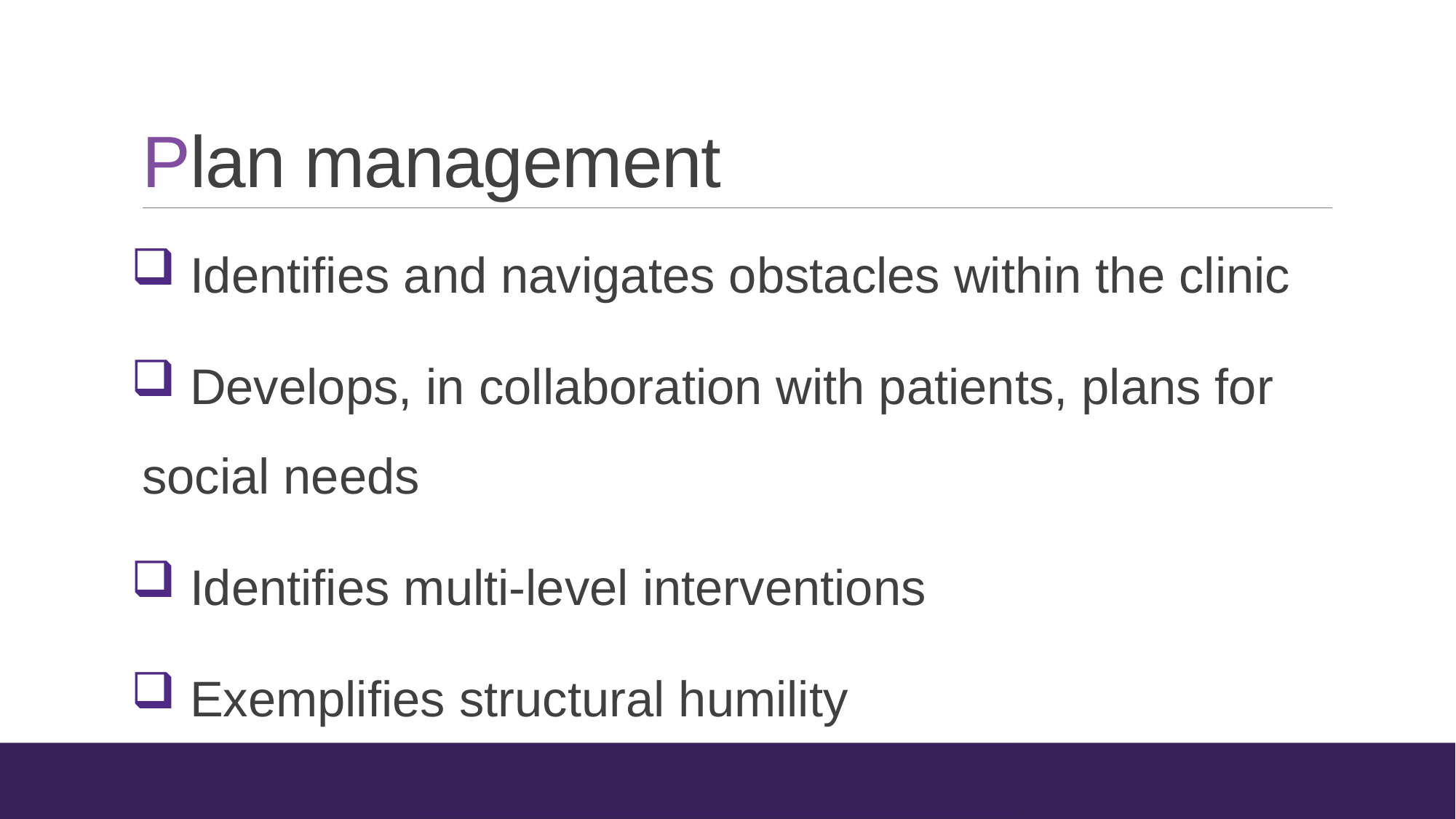

# Plan management
 Identifies and navigates obstacles within the clinic
 Develops, in collaboration with patients, plans for social needs
 Identifies multi-level interventions
 Exemplifies structural humility

## Slide 44
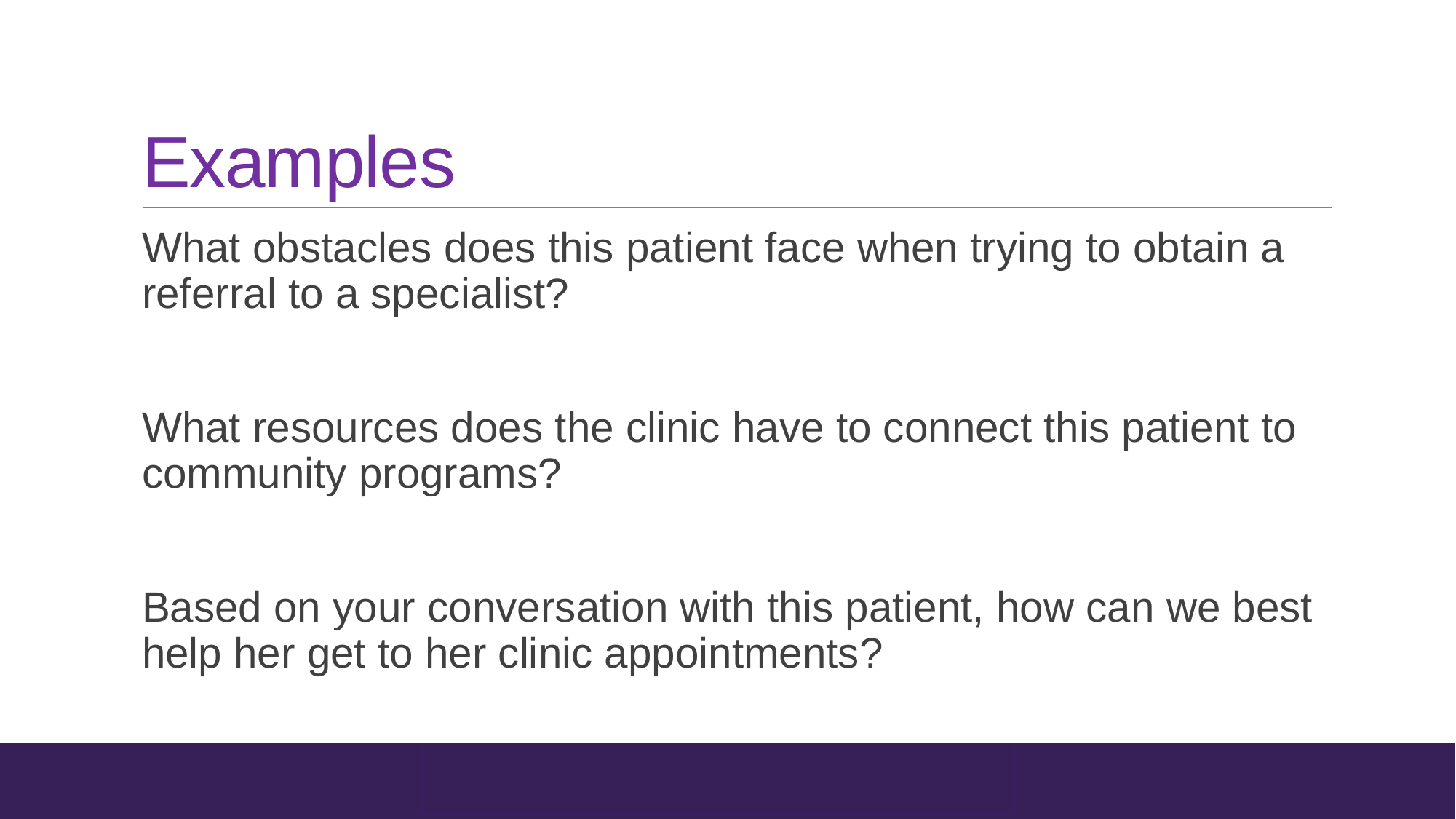

# Examples
What obstacles does this patient face when trying to obtain a referral to a specialist?
What resources does the clinic have to connect this patient to community programs?
Based on your conversation with this patient, how can we best help her get to her clinic appointments?

## Slide 45
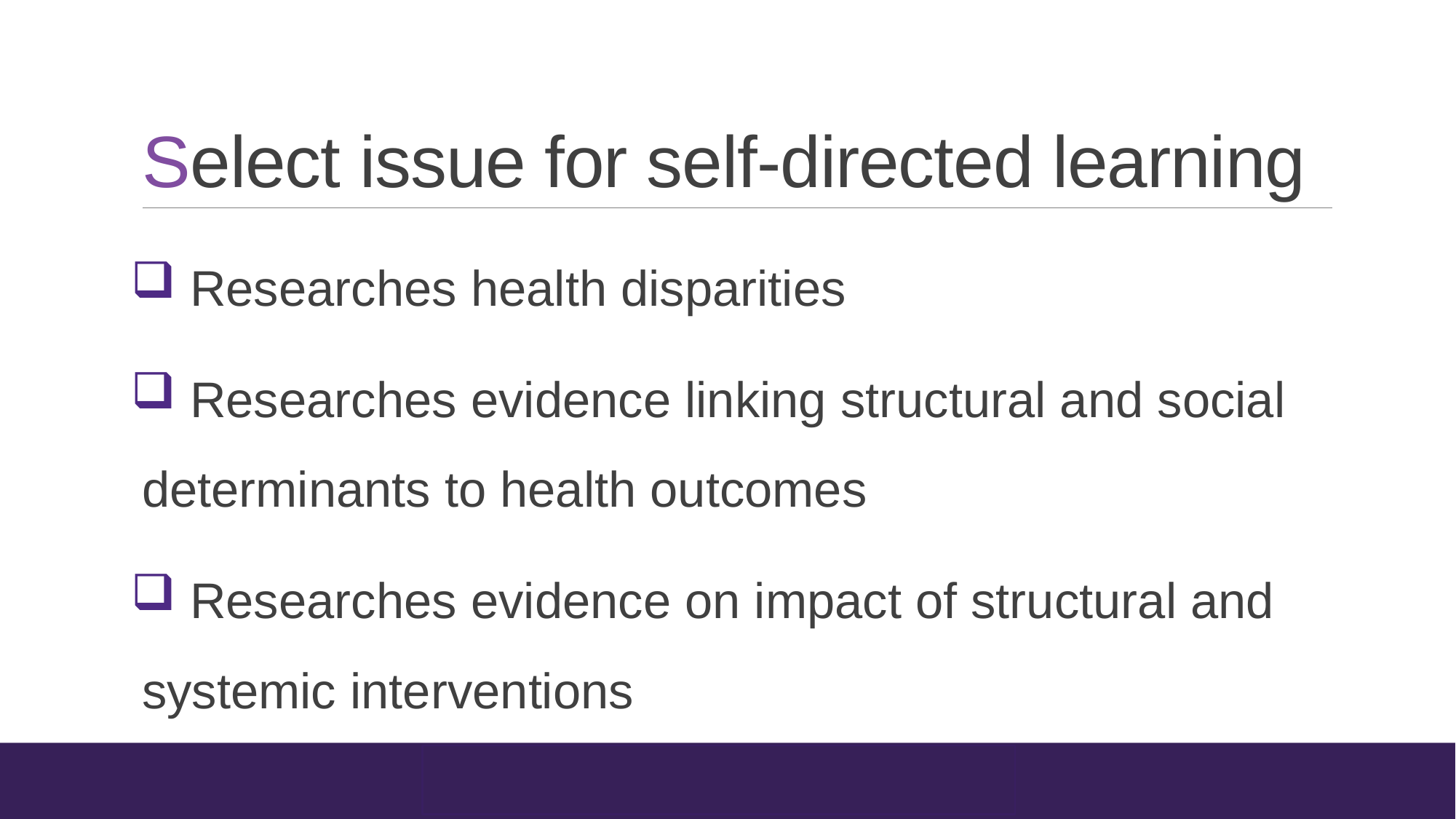

# Select issue for self-directed learning
 Researches health disparities
 Researches evidence linking structural and social determinants to health outcomes
 Researches evidence on impact of structural and systemic interventions

## Slide 46
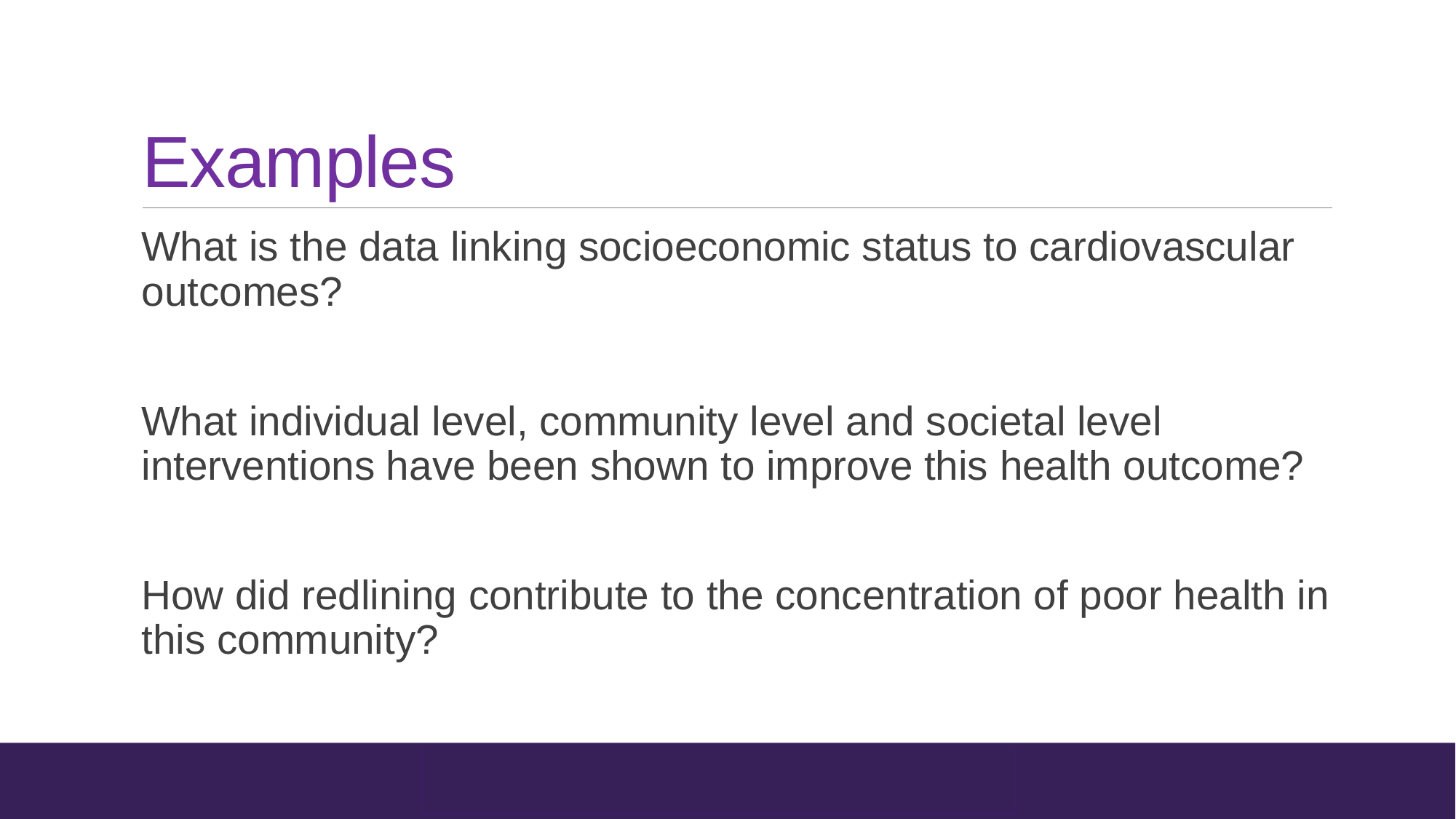

# Examples
What is the data linking socioeconomic status to cardiovascular outcomes?
What individual level, community level and societal level interventions have been shown to improve this health outcome?
How did redlining contribute to the concentration of poor health in this community?

## Slide 47
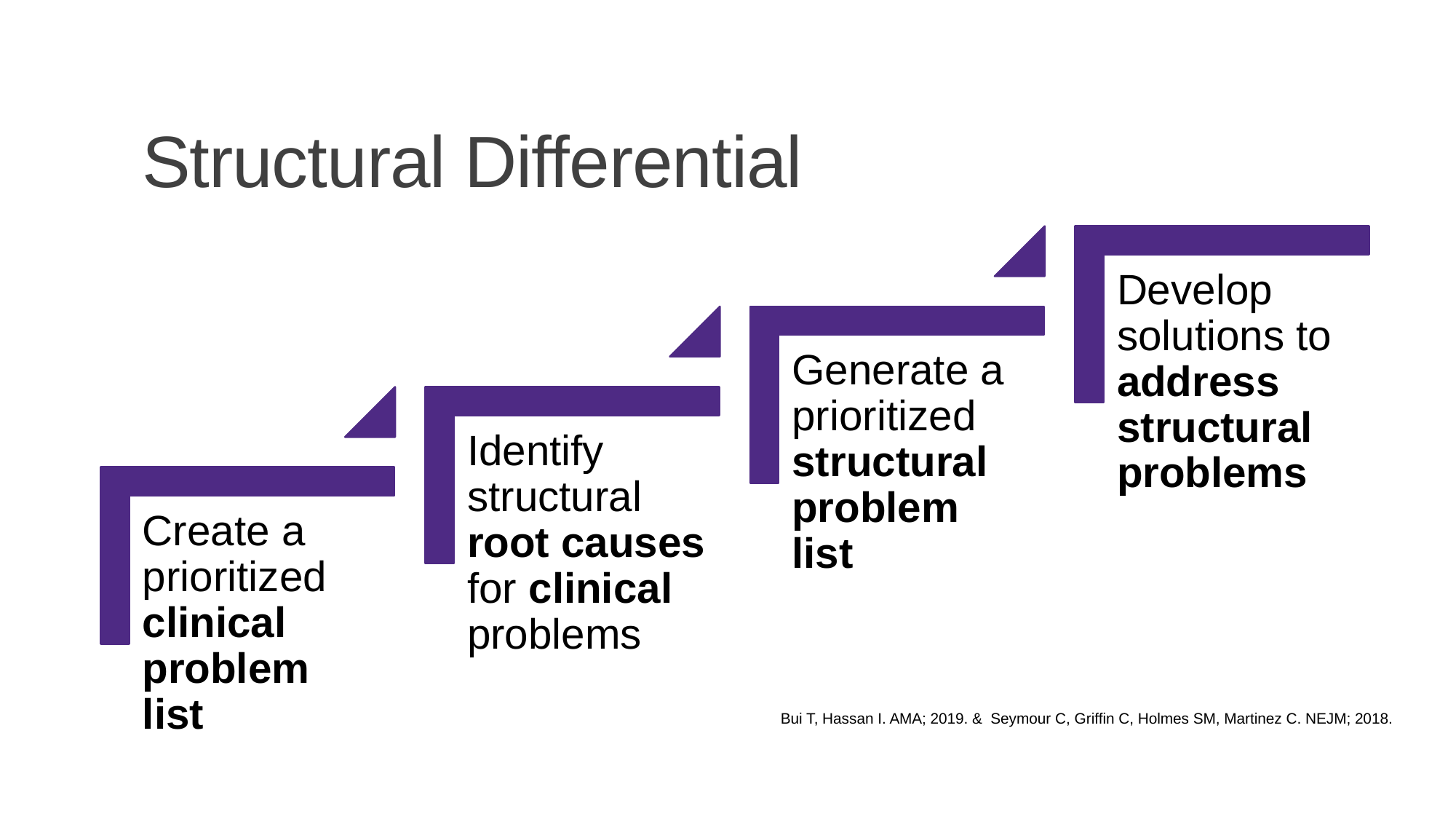

# Structural Differential
Bui T, Hassan I. AMA; 2019. & Seymour C, Griffin C, Holmes SM, Martinez C. NEJM; 2018.

## Slide 48
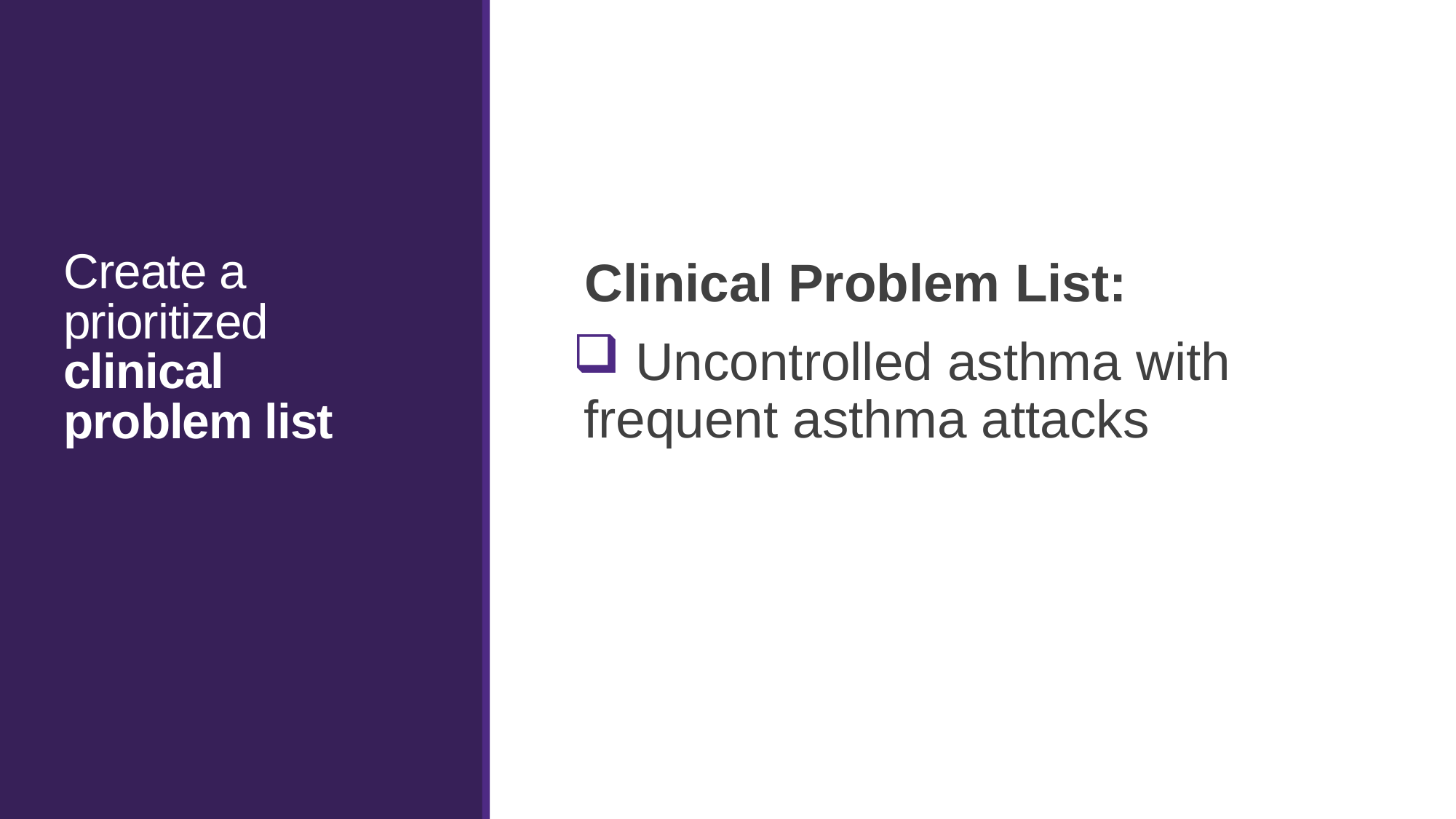

Clinical Problem List:
 Uncontrolled asthma with frequent asthma attacks
# Create a prioritized clinical problem list

## Slide 49
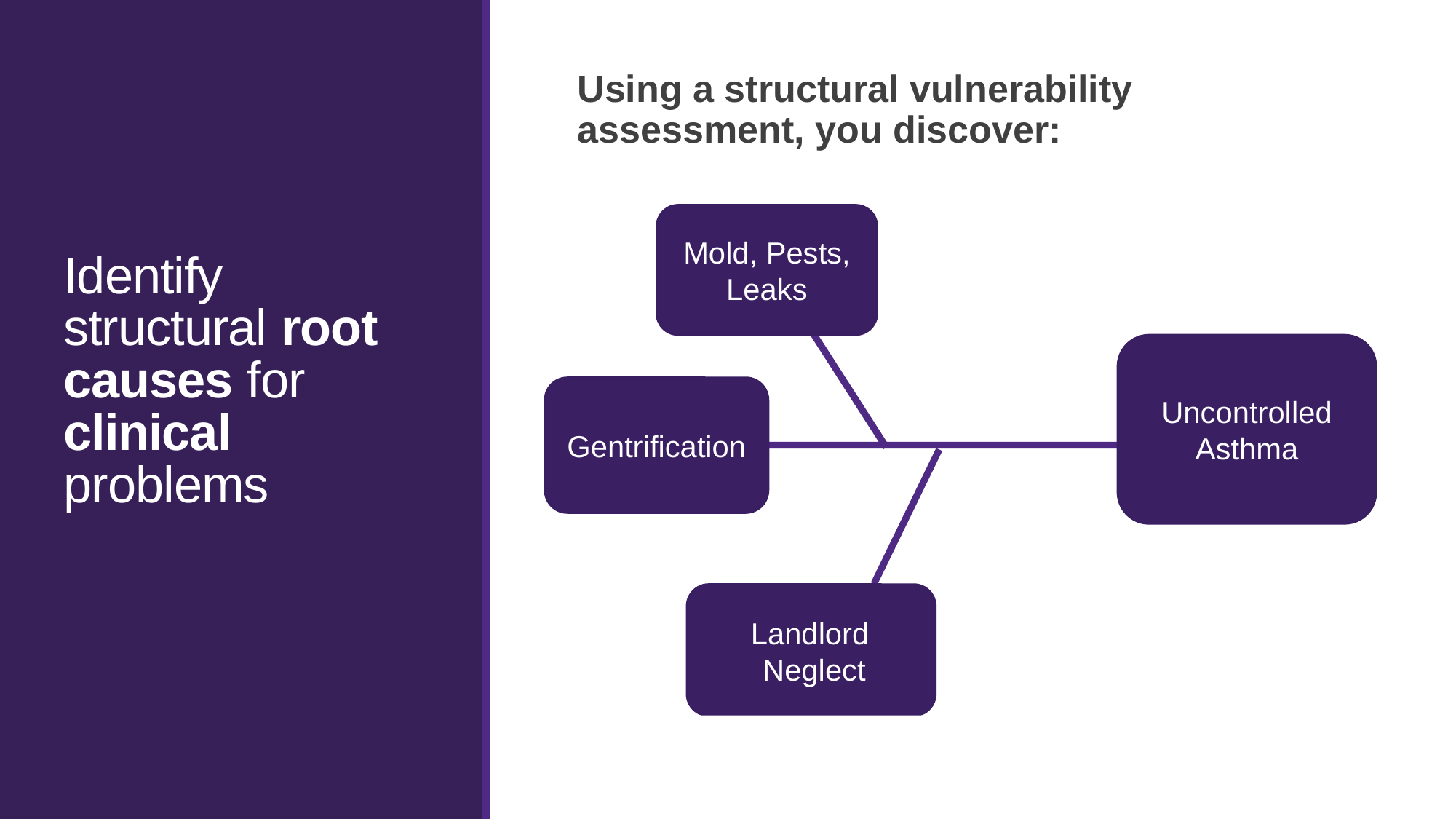

Using a structural vulnerability assessment, you discover:
Mold, Pests, Leaks
# Identify structural root causes for clinical problems
Uncontrolled Asthma
Gentrification
Landlord
Neglect

## Slide 50
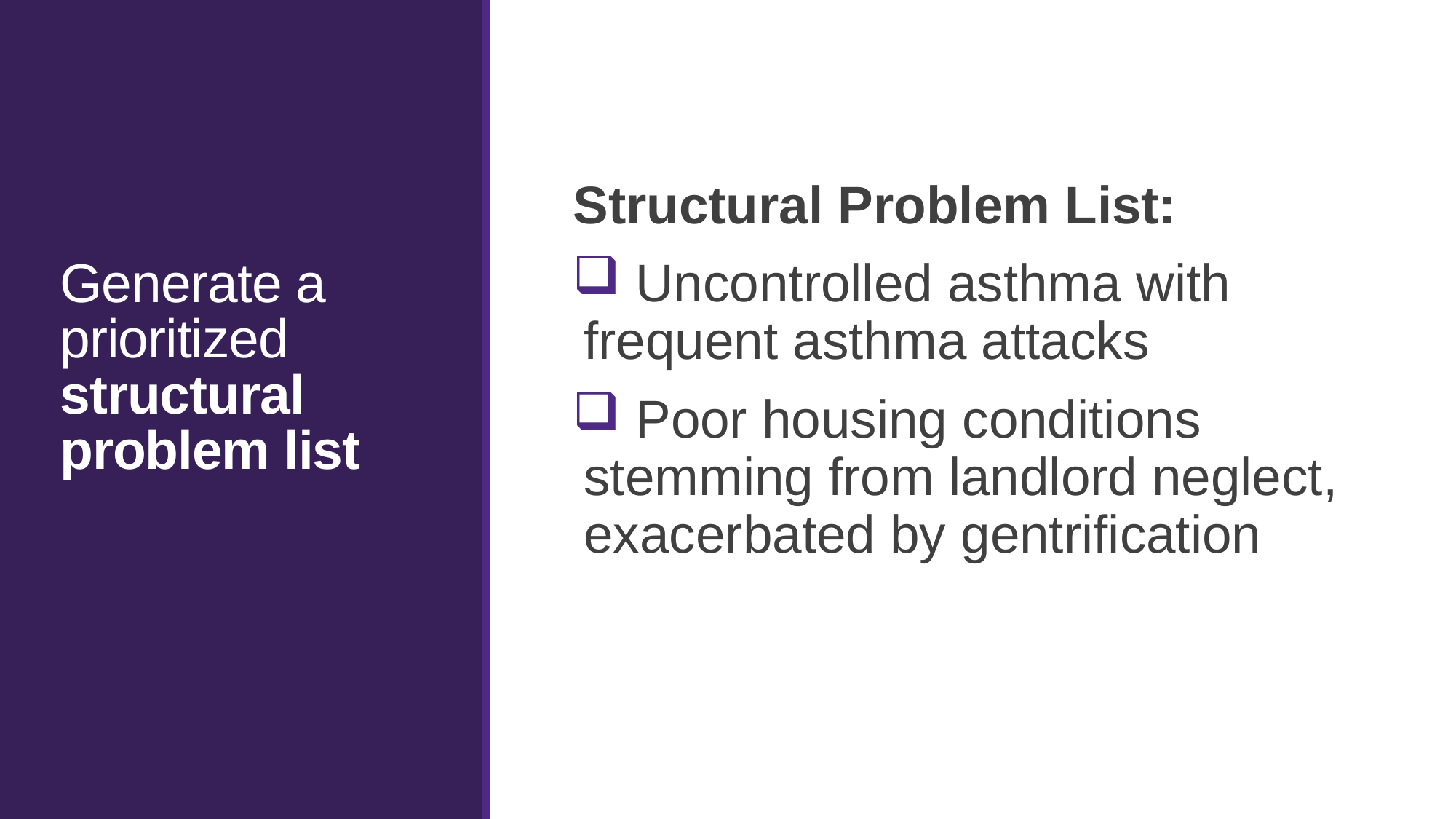

Structural Problem List:
 Uncontrolled asthma with frequent asthma attacks
 Poor housing conditions stemming from landlord neglect, exacerbated by gentrification
# Generate a prioritized structural problem list

## Slide 51
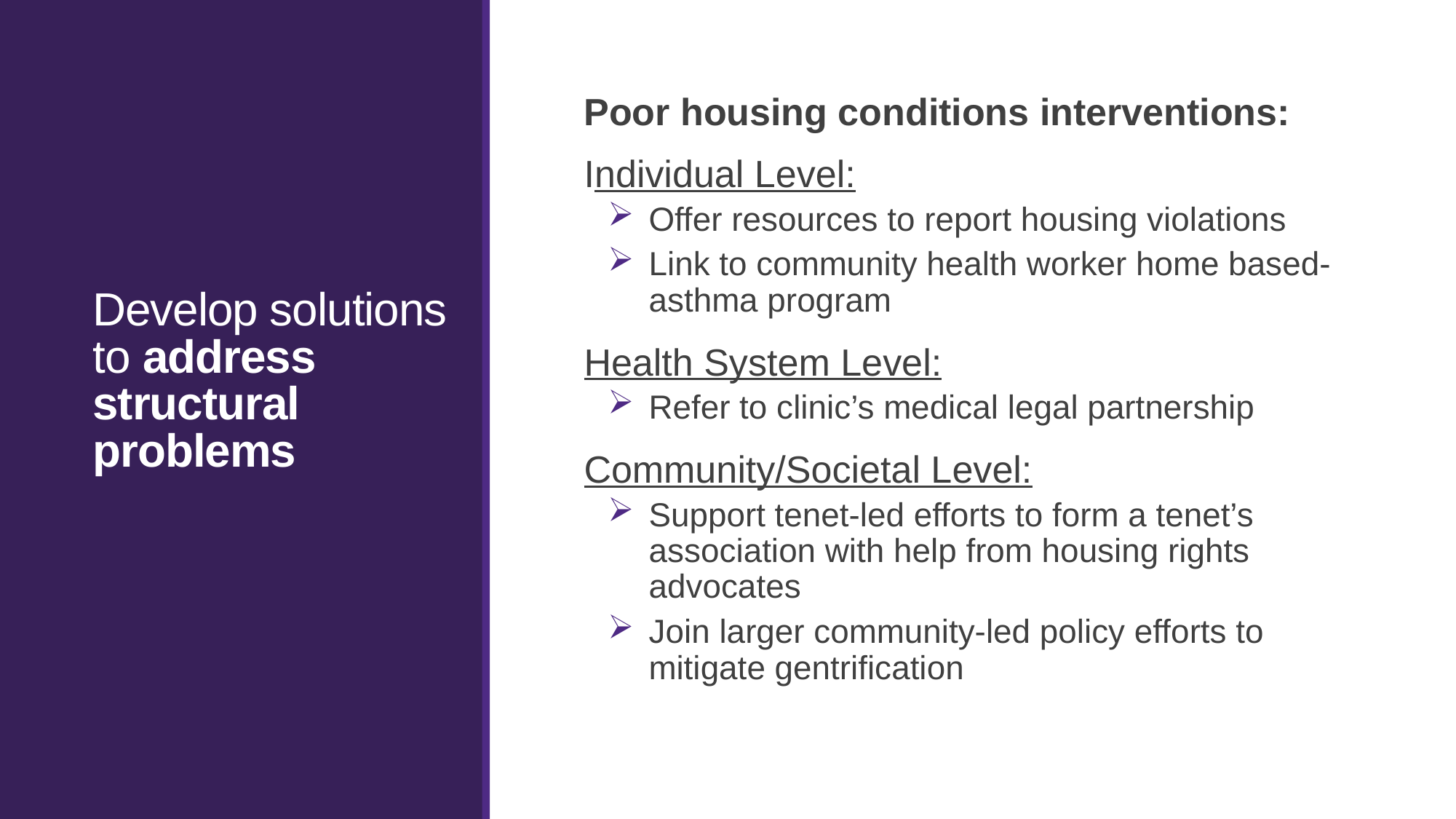

Poor housing conditions interventions:
Individual Level:
Offer resources to report housing violations
Link to community health worker home based-asthma program
Health System Level:
Refer to clinic’s medical legal partnership
Community/Societal Level:
Support tenet-led efforts to form a tenet’s association with help from housing rights advocates
Join larger community-led policy efforts to mitigate gentrification
# Develop solutions to address structural problems

## Slide 52
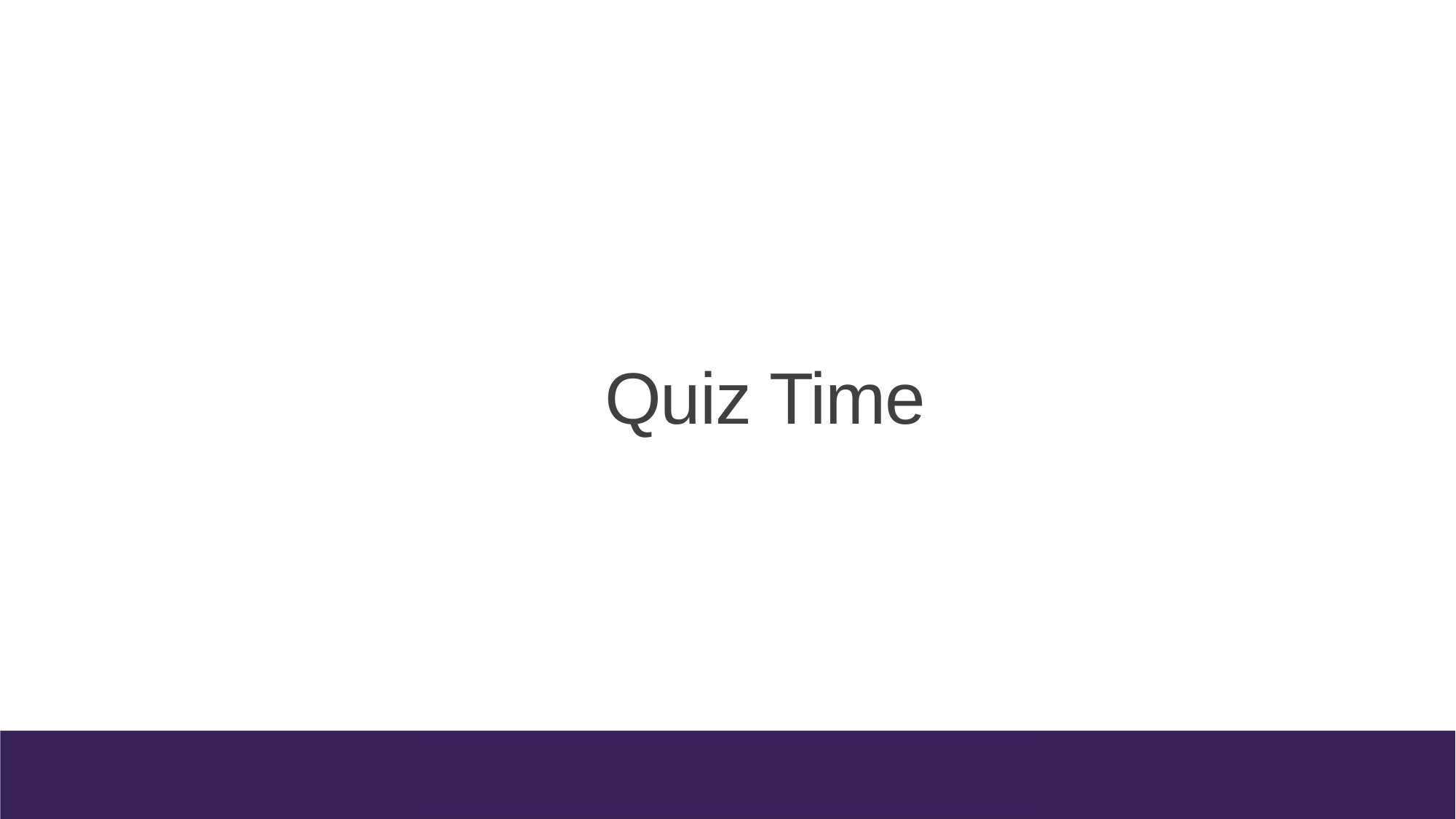

Quiz Time

## Slide 53
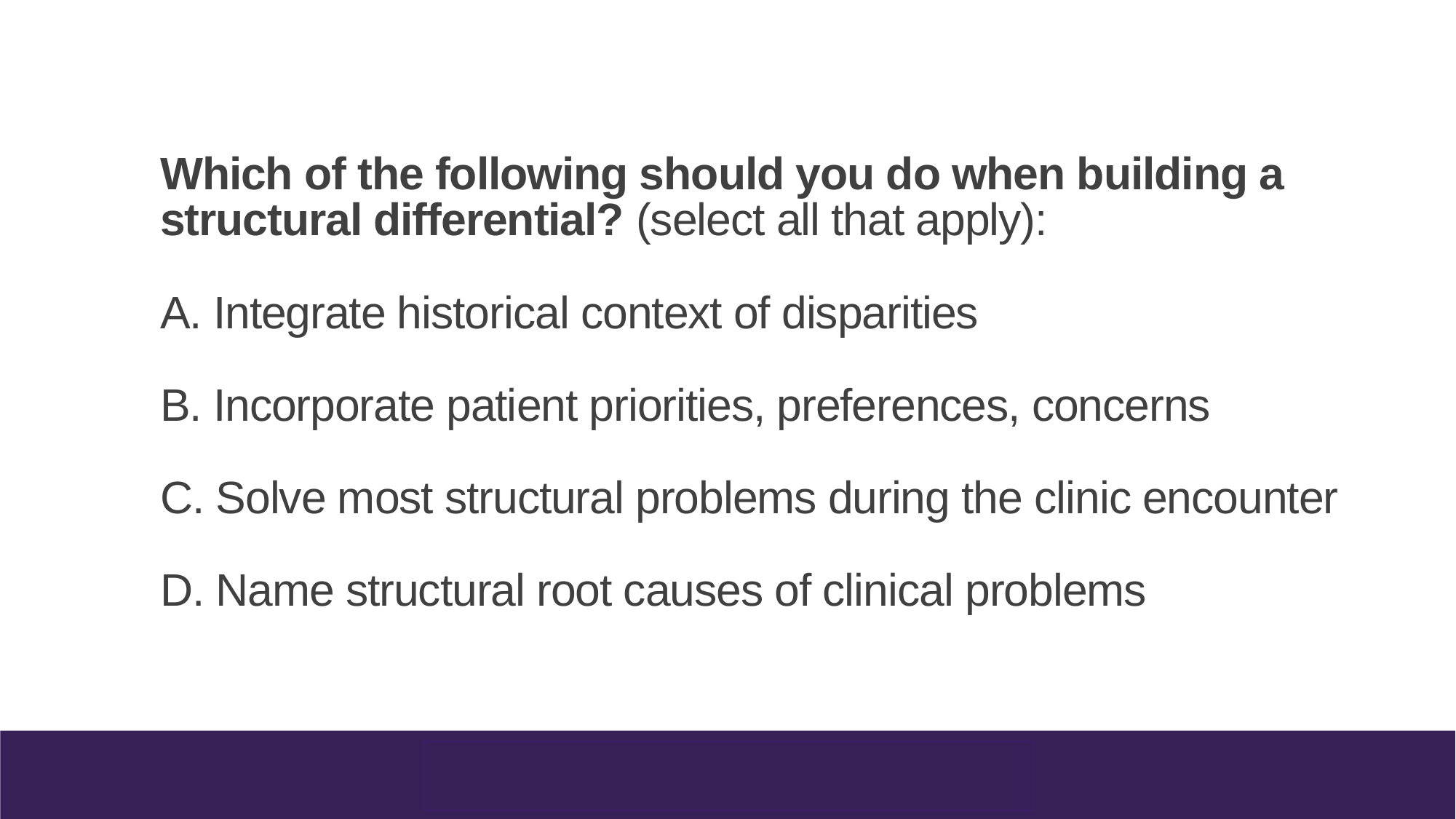

Which of the following should you do when building a structural differential? (select all that apply):
A. Integrate historical context of disparities
B. Incorporate patient priorities, preferences, concerns
C. Solve most structural problems during the clinic encounter
D. Name structural root causes of clinical problems

## Slide 54
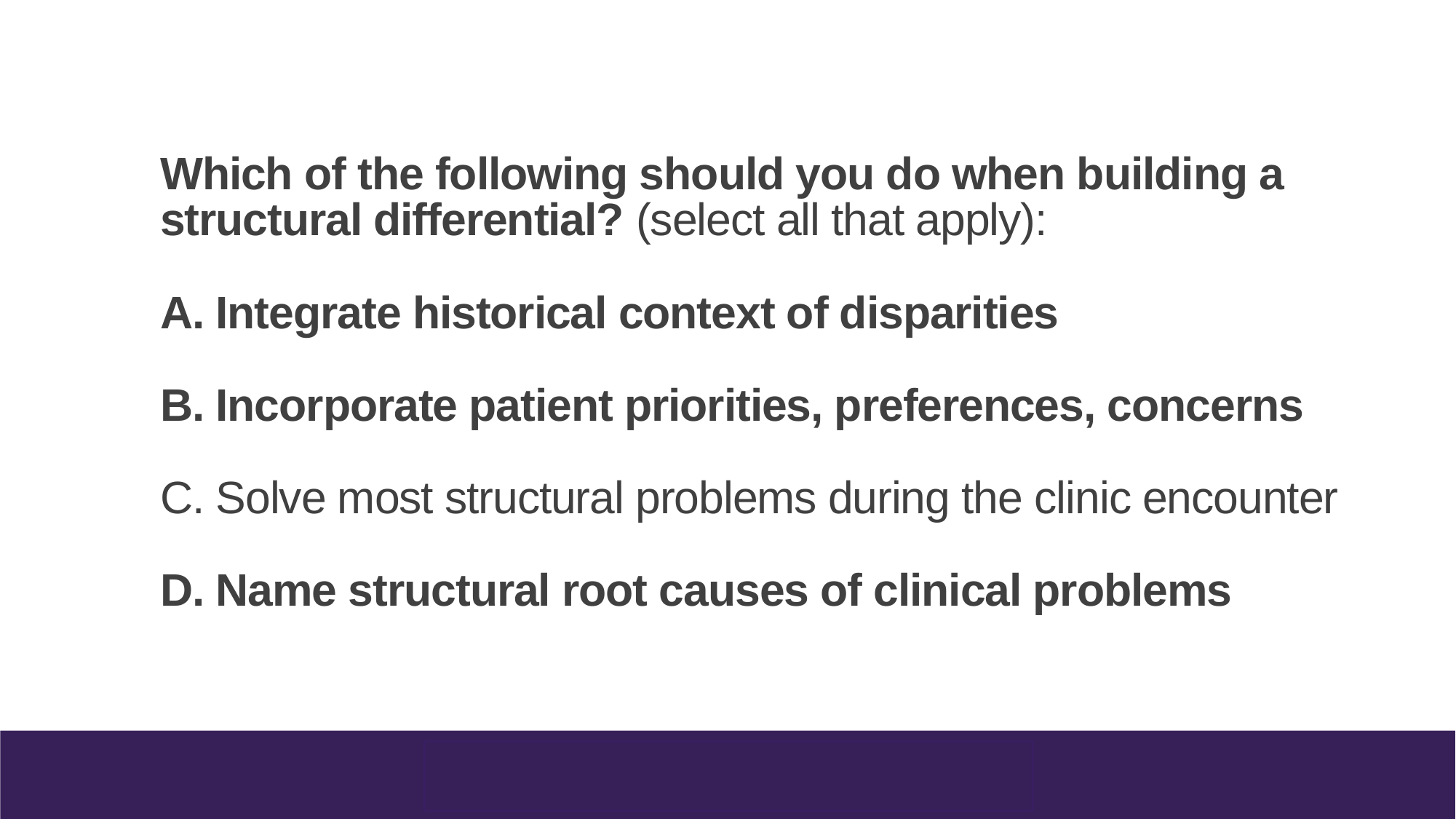

Which of the following should you do when building a structural differential? (select all that apply):
A. Integrate historical context of disparities
B. Incorporate patient priorities, preferences, concerns
C. Solve most structural problems during the clinic encounter
D. Name structural root causes of clinical problems

## Slide 55
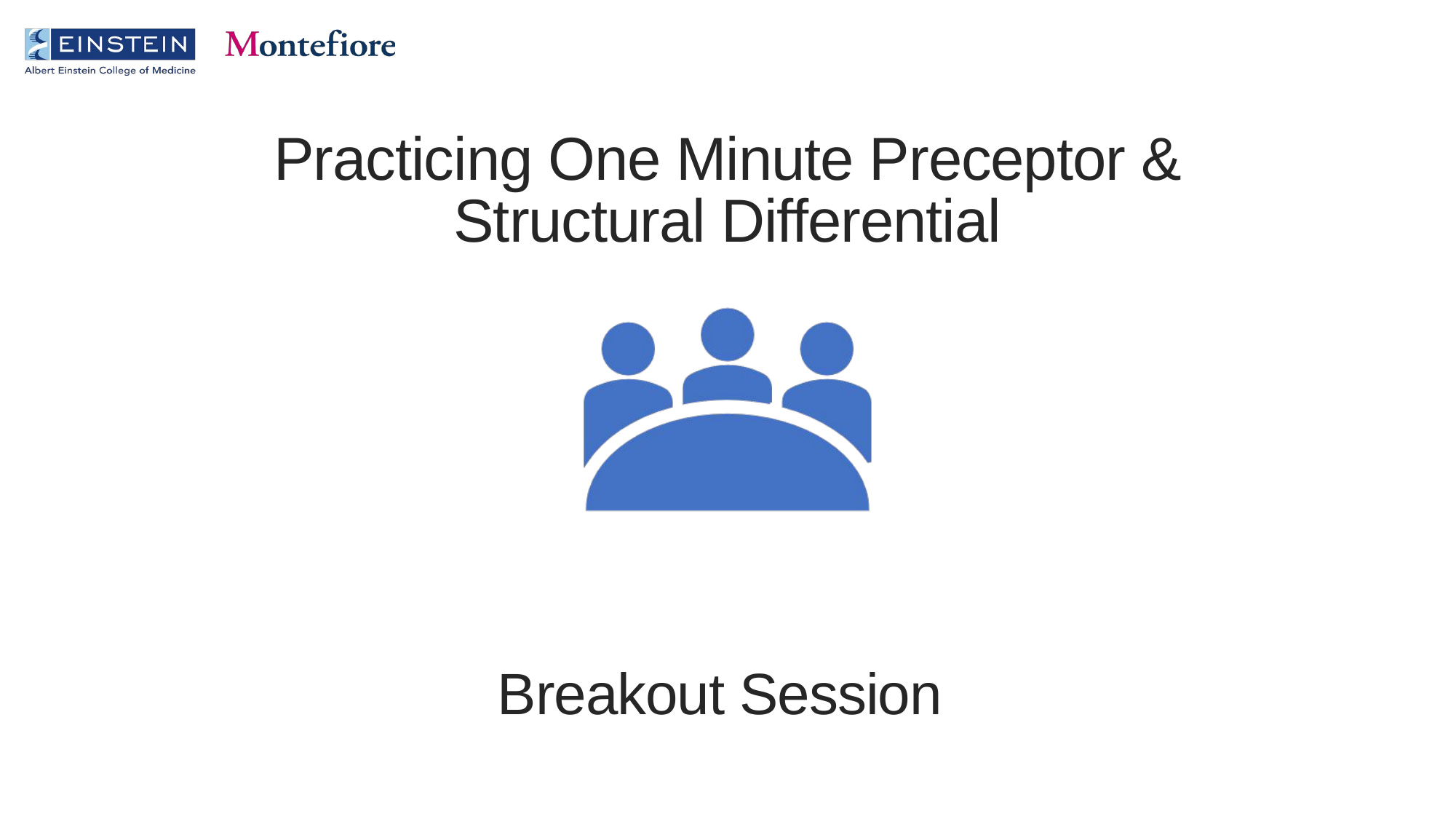

Practicing One Minute Preceptor & Structural Differential
Breakout Session

## Slide 56
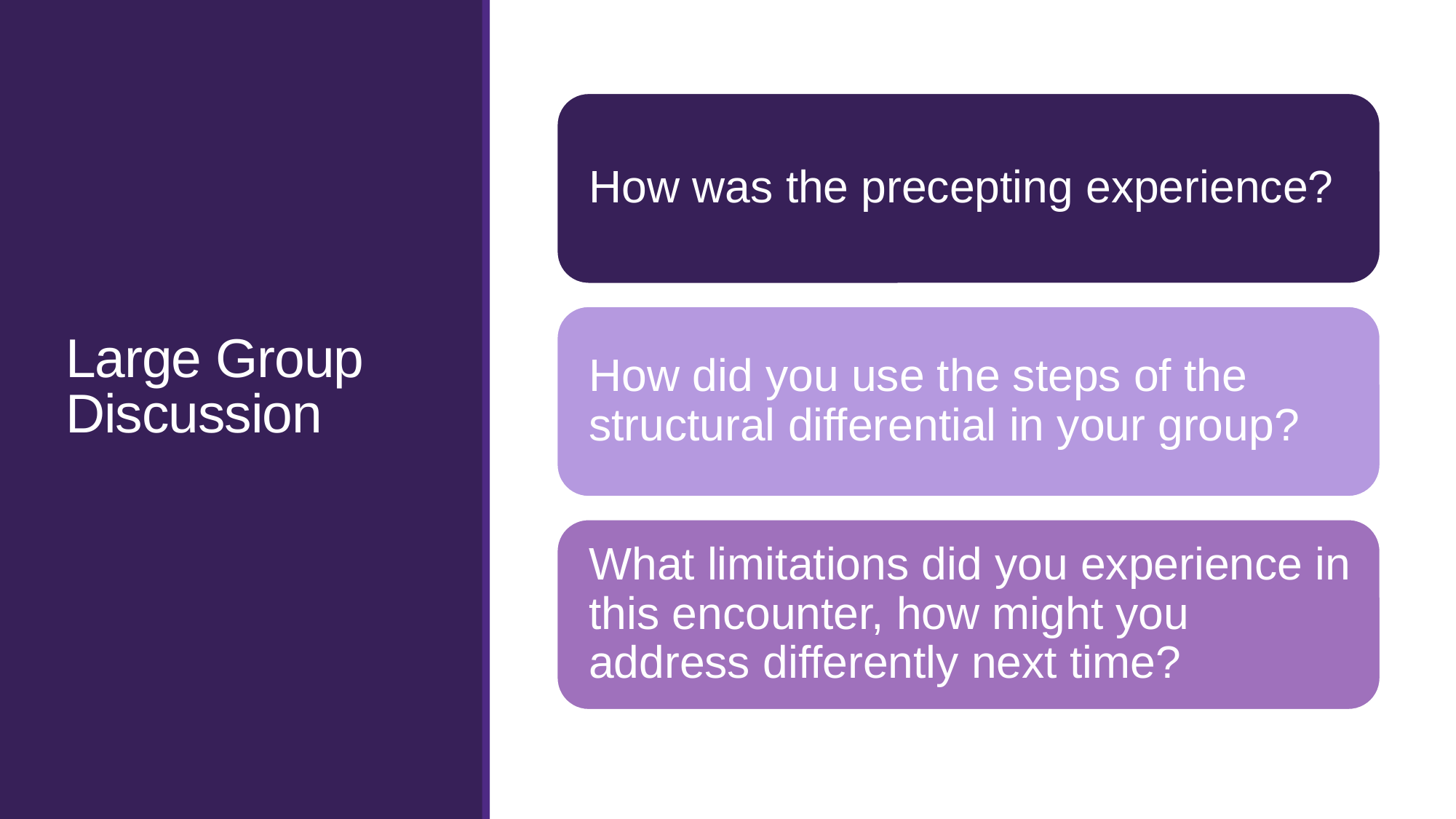

# Large Group Discussion

## Slide 57
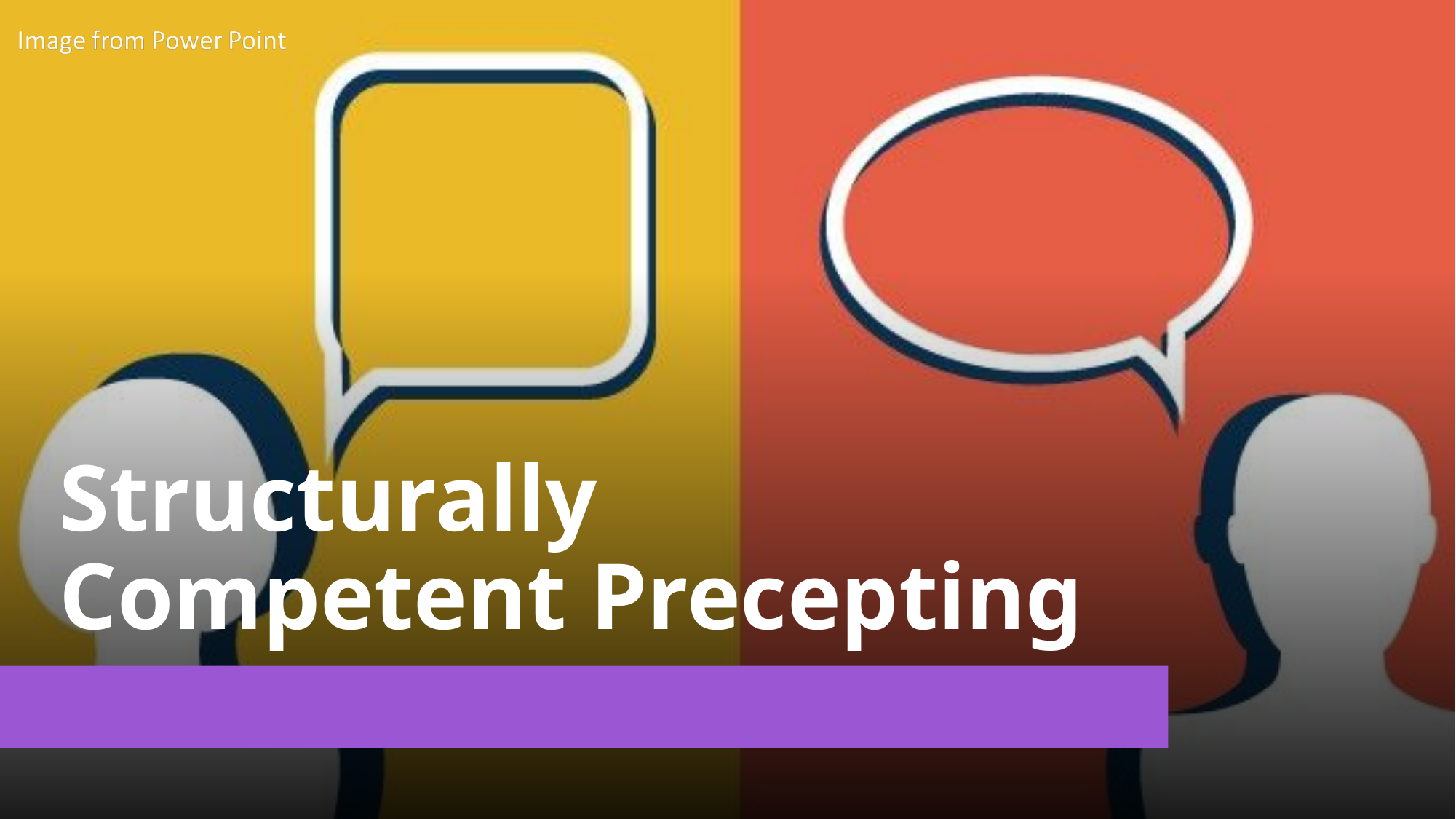

# Structurally Competent Precepting

## Slide 58
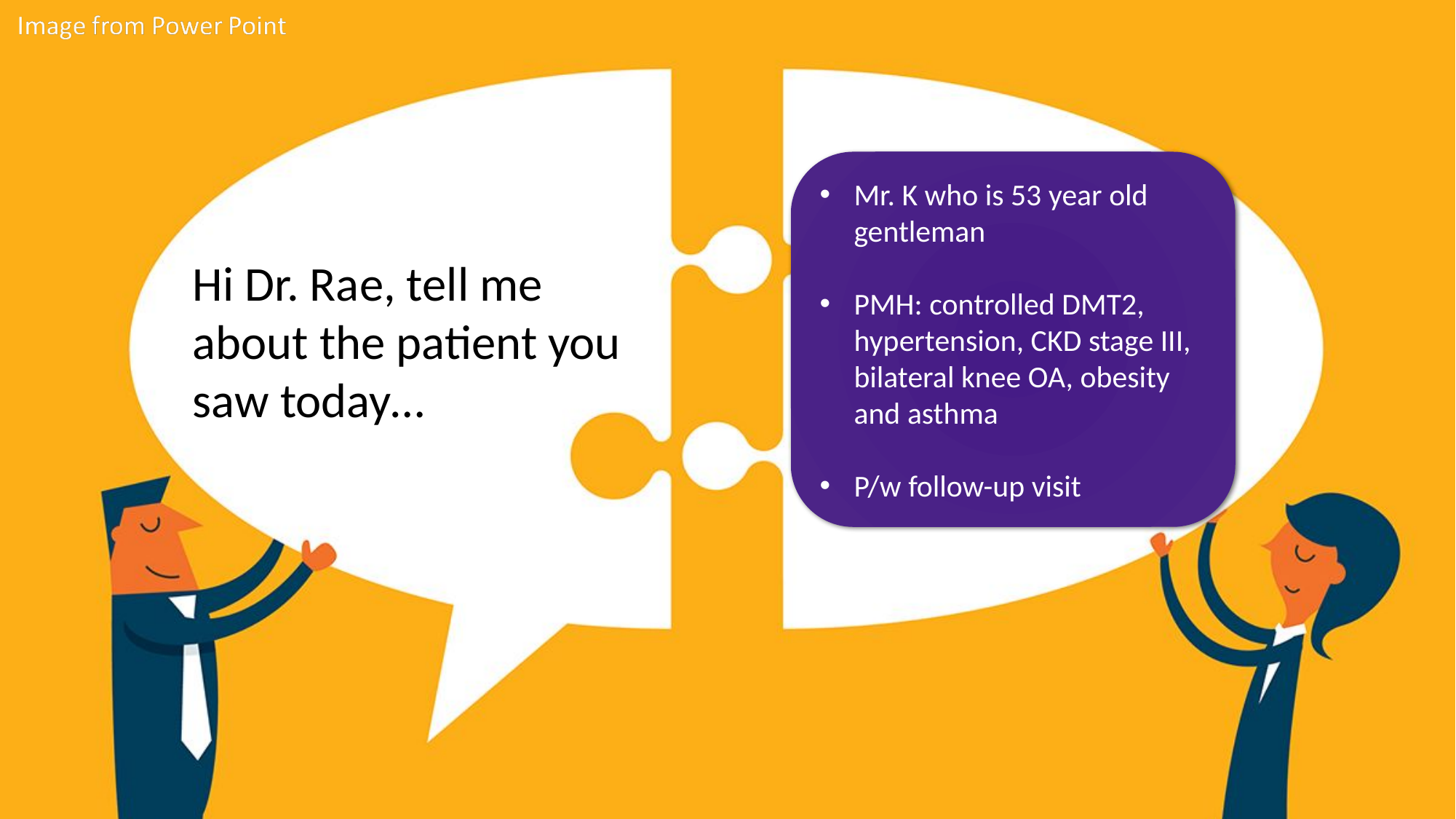

Mr. K who is 53 year old gentleman
PMH: controlled DMT2, hypertension, CKD stage III, bilateral knee OA, obesity and asthma
P/w follow-up visit
Hi Dr. Rae, tell me about the patient you saw today…

## Slide 59
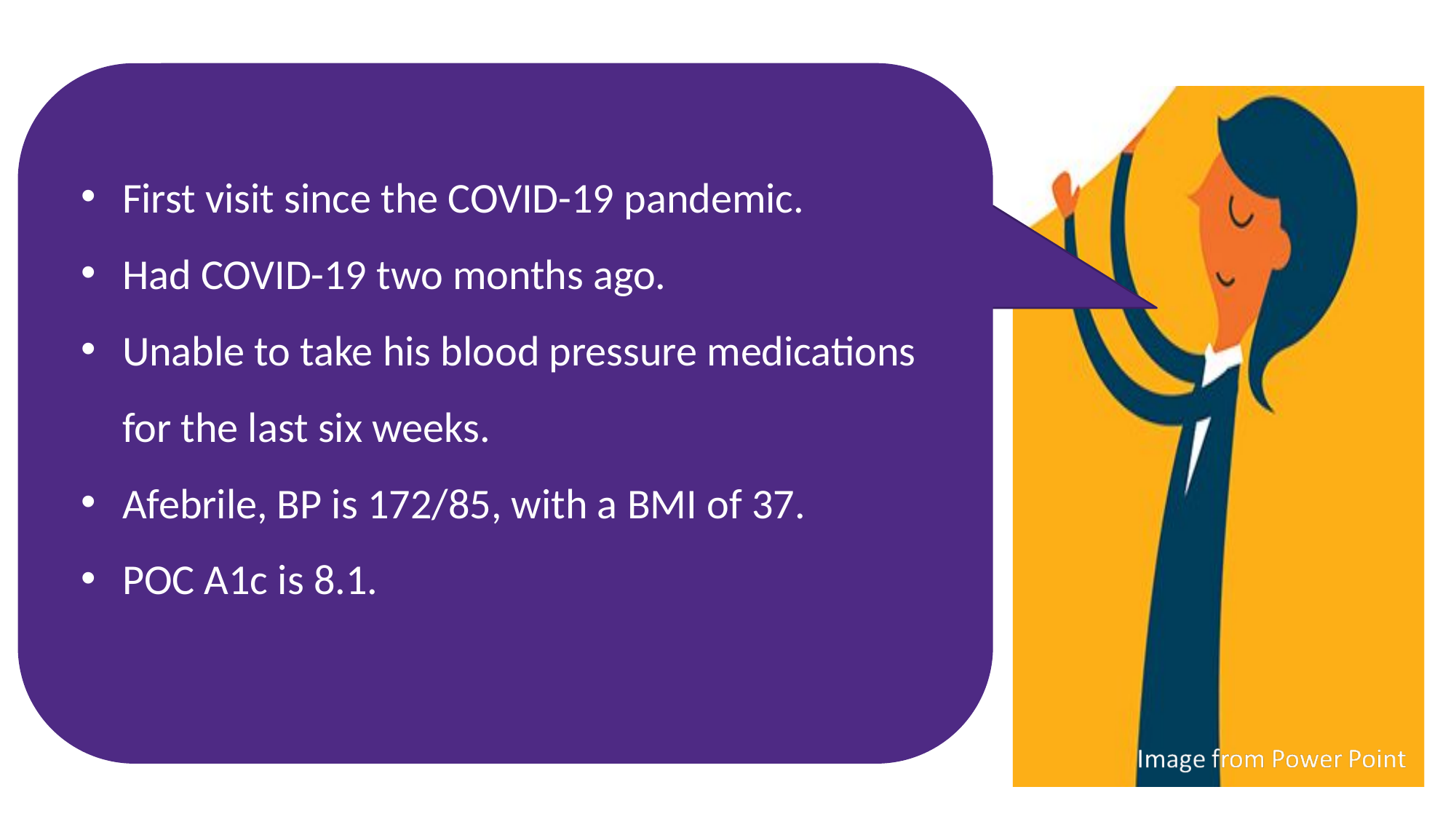

First visit since the COVID-19 pandemic.
Had COVID-19 two months ago.
Unable to take his blood pressure medications for the last six weeks.
Afebrile, BP is 172/85, with a BMI of 37.
POC A1c is 8.1.

## Slide 60
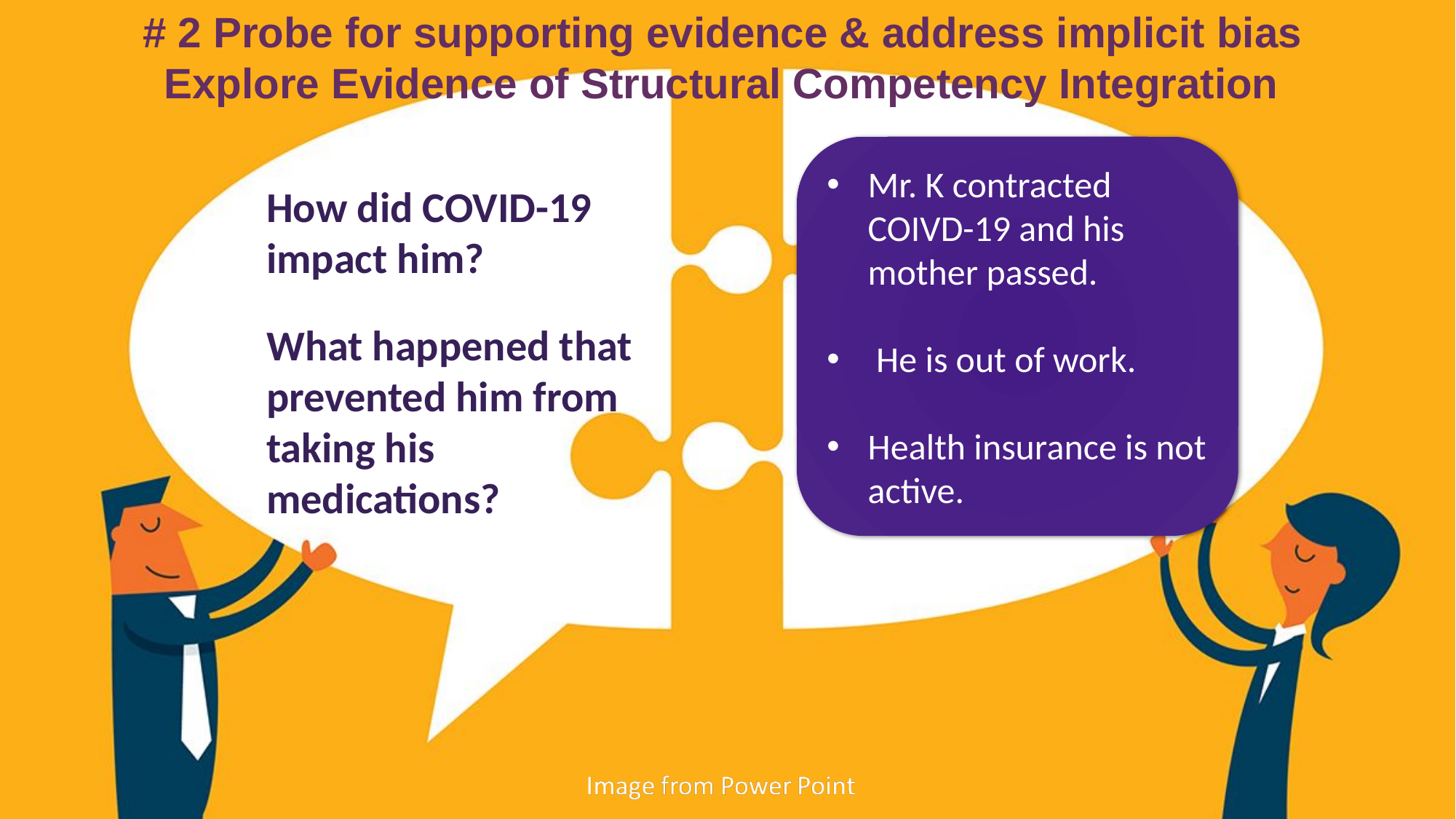

# 2 Probe for supporting evidence & address implicit bias
Explore Evidence of Structural Competency Integration
Mr. K contracted COIVD-19 and his mother passed.
 He is out of work.
Health insurance is not active.
How did COVID-19 impact him?
What happened that prevented him from taking his medications?

## Slide 61
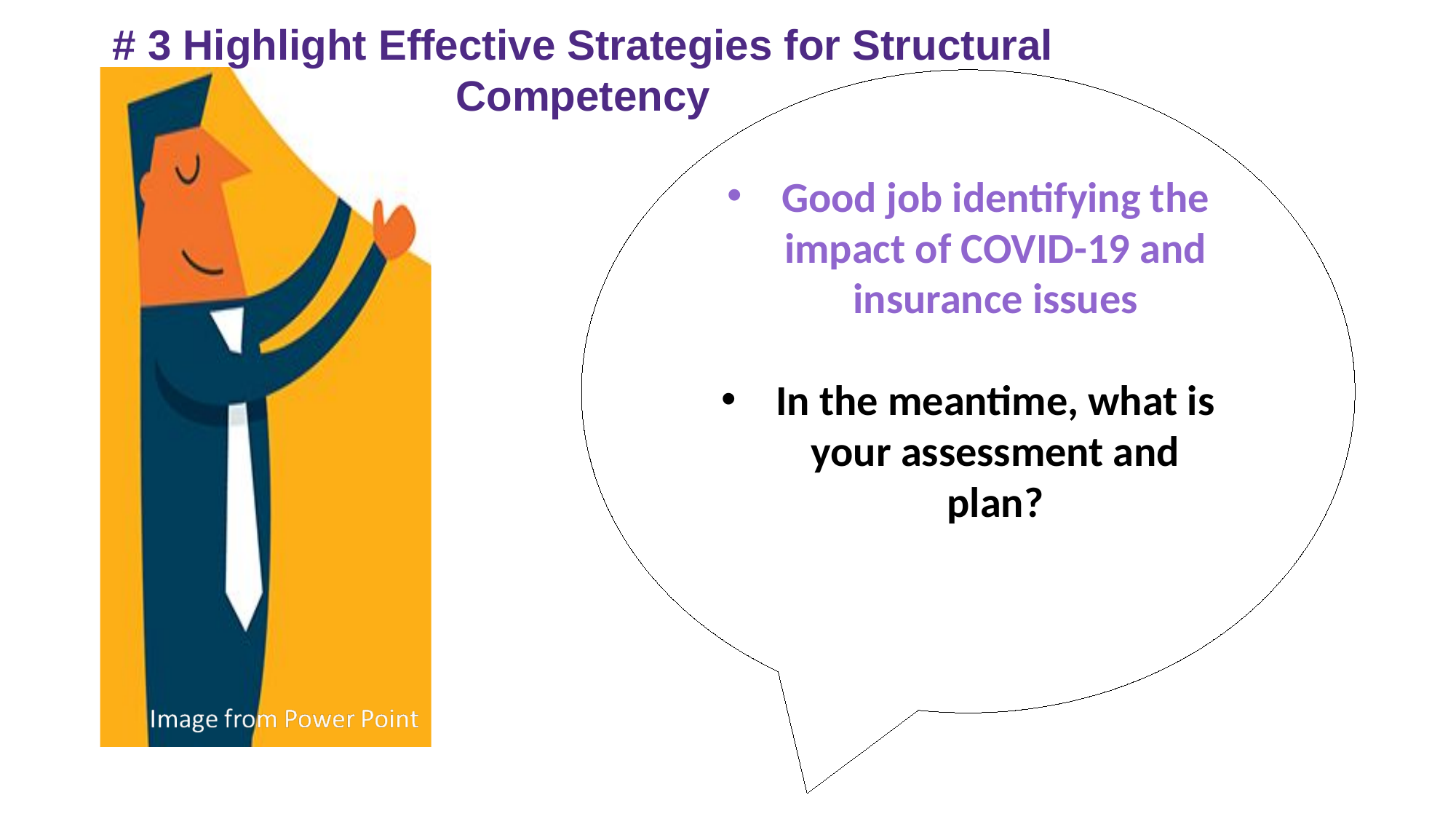

# 3 Highlight Effective Strategies for Structural Competency
Good job identifying the impact of COVID-19 and insurance issues
In the meantime, what is your assessment and plan?

## Slide 62
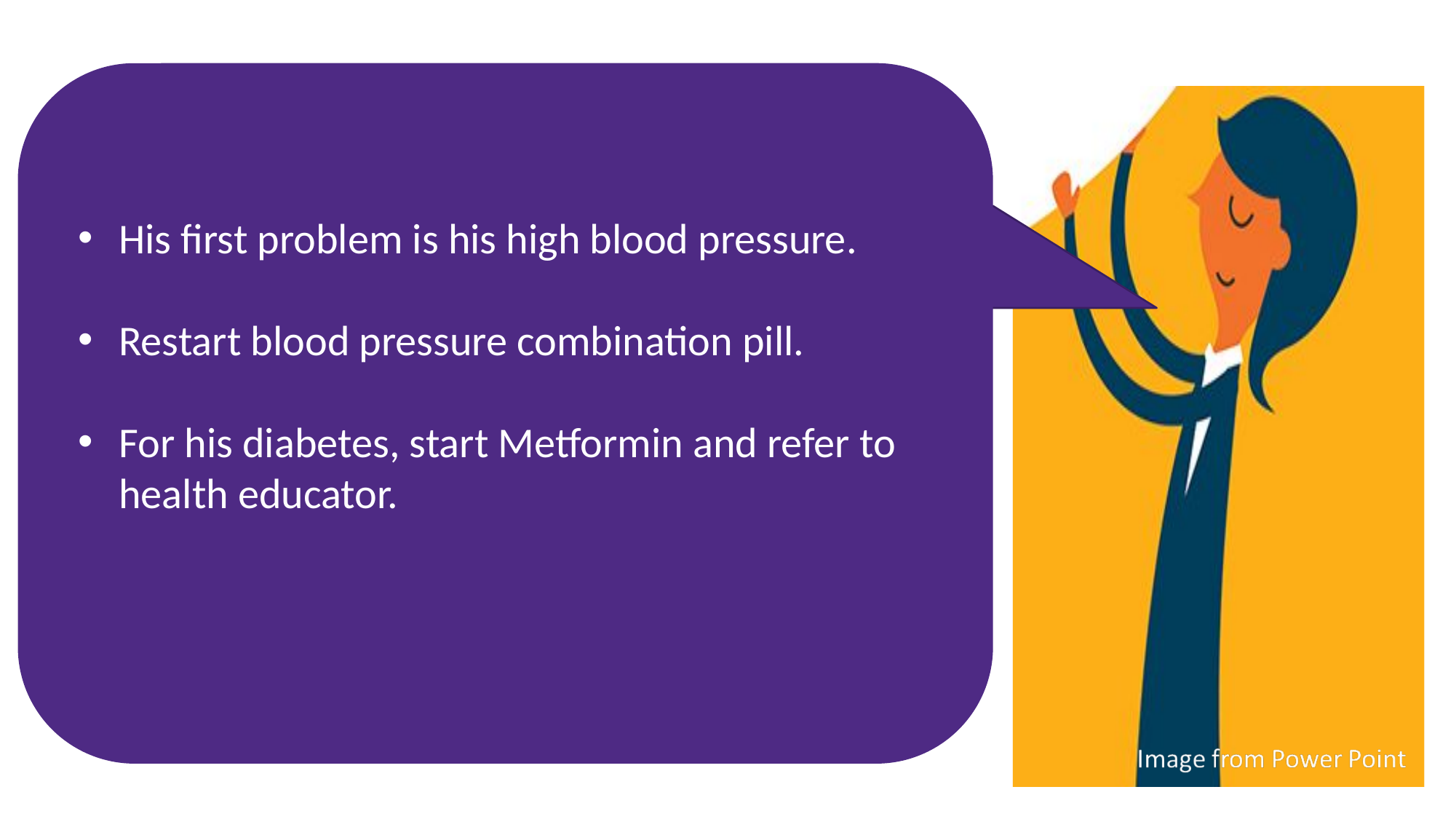

His first problem is his high blood pressure.
Restart blood pressure combination pill.
For his diabetes, start Metformin and refer to health educator.

## Slide 63
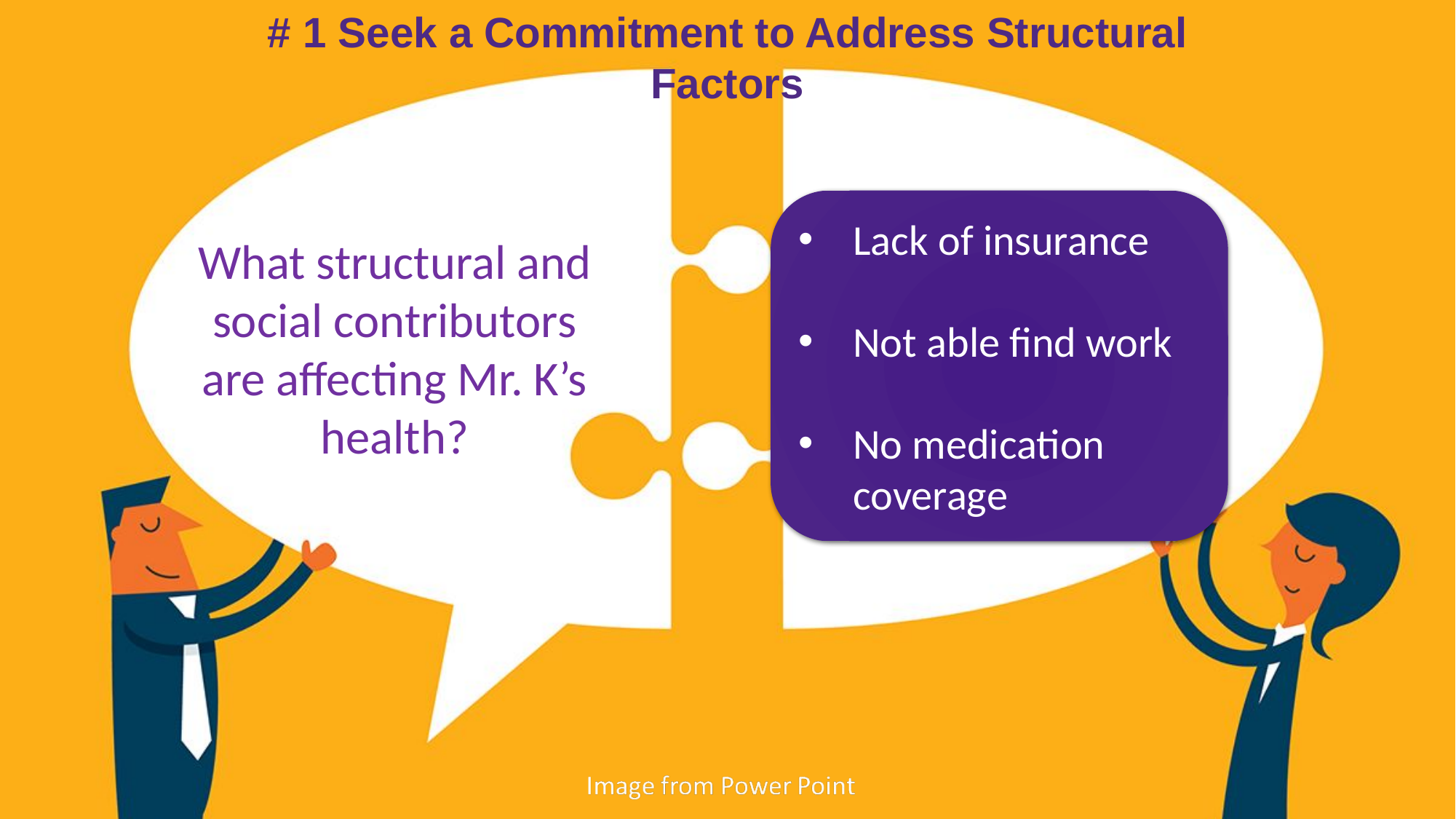

# 1 Seek a Commitment to Address Structural Factors
Lack of insurance
Not able find work
No medication coverage
What structural and social contributors are affecting Mr. K’s health?

## Slide 64
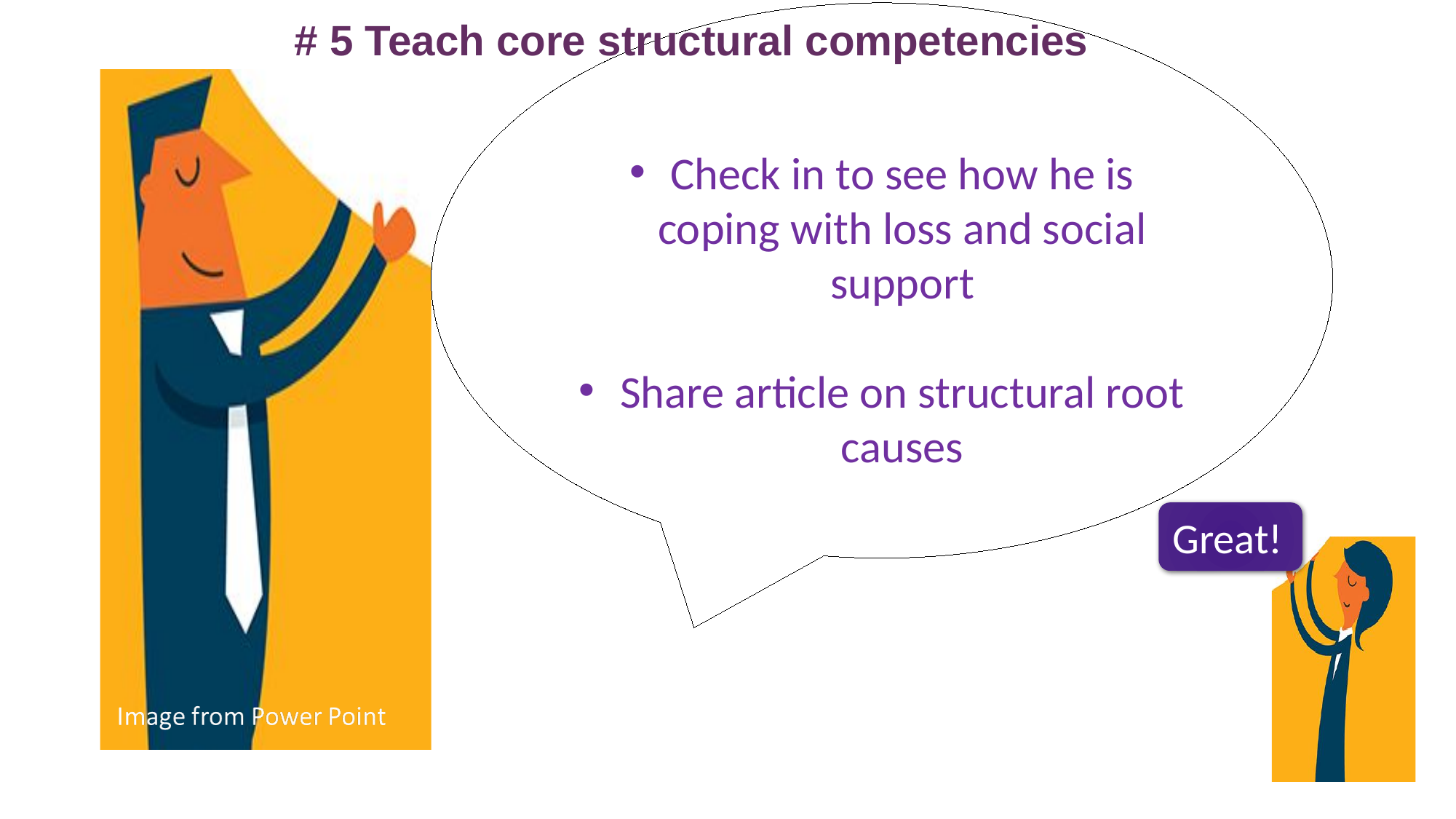

Check in to see how he is coping with loss and social support
Share article on structural root causes
# 5 Teach core structural competencies
Great!

## Slide 65
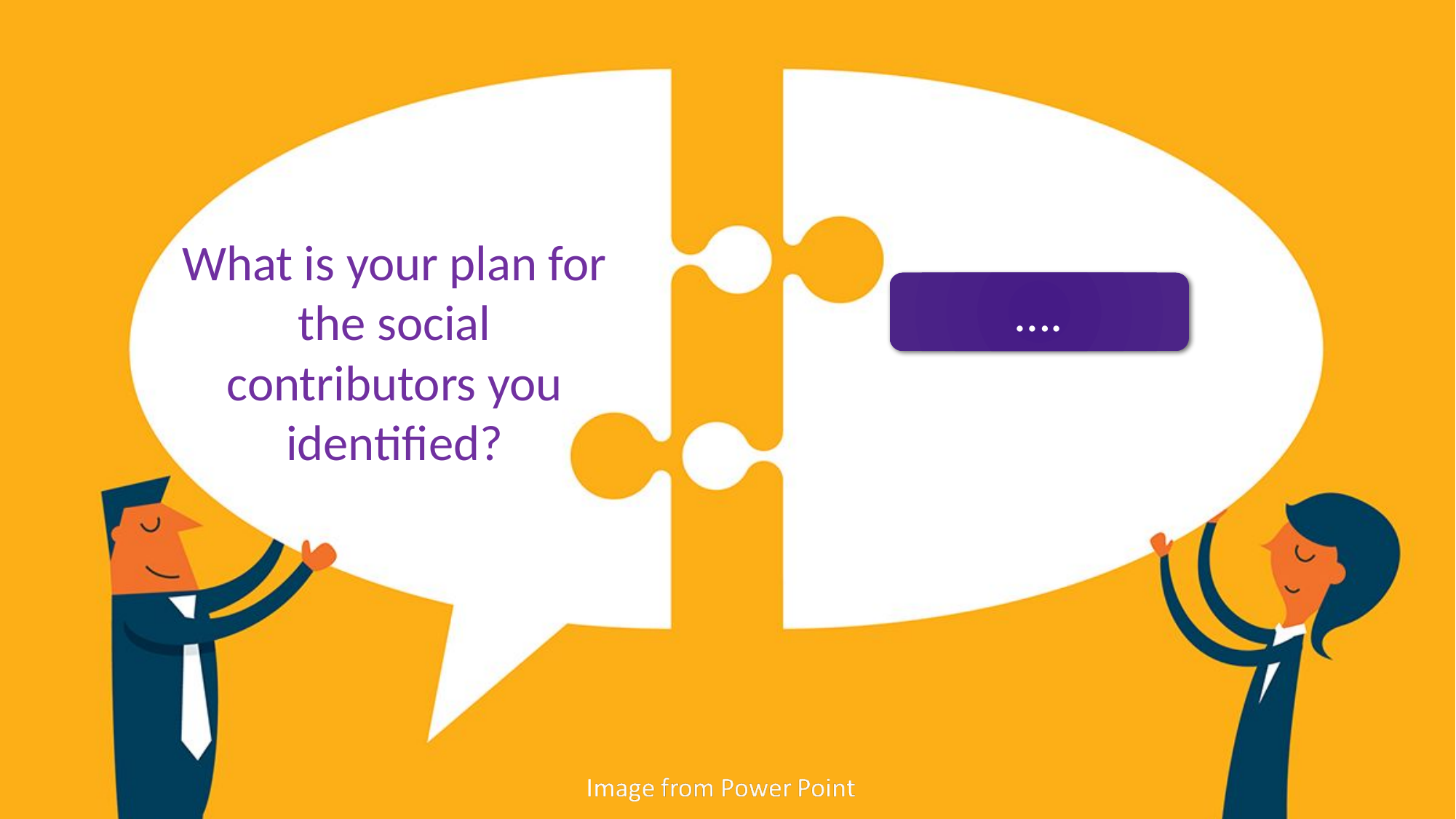

What is your plan for the social contributors you identified?
….

## Slide 66
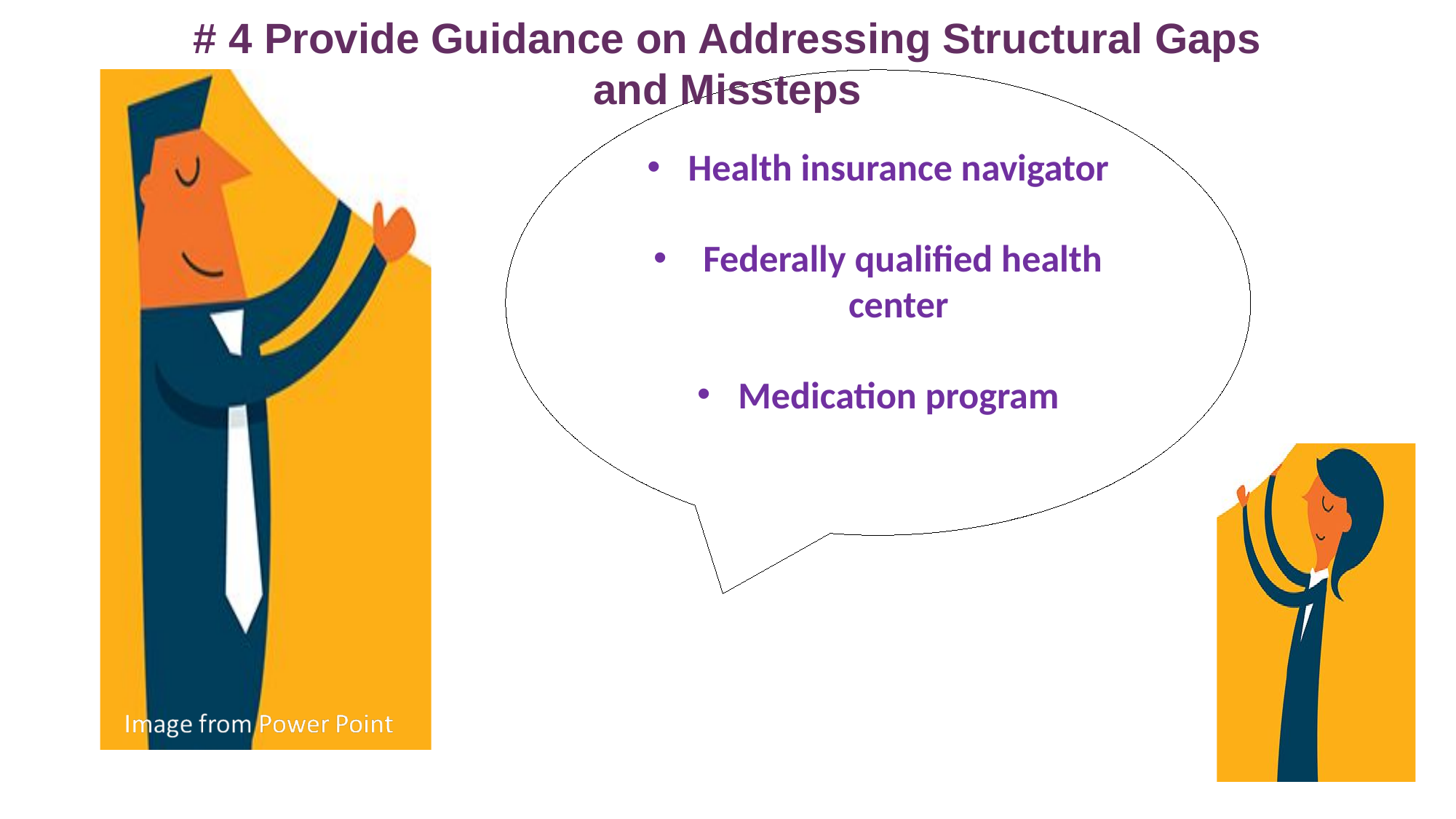

# 4 Provide Guidance on Addressing Structural Gaps and Missteps
Health insurance navigator
 Federally qualified health center
Medication program

## Slide 67
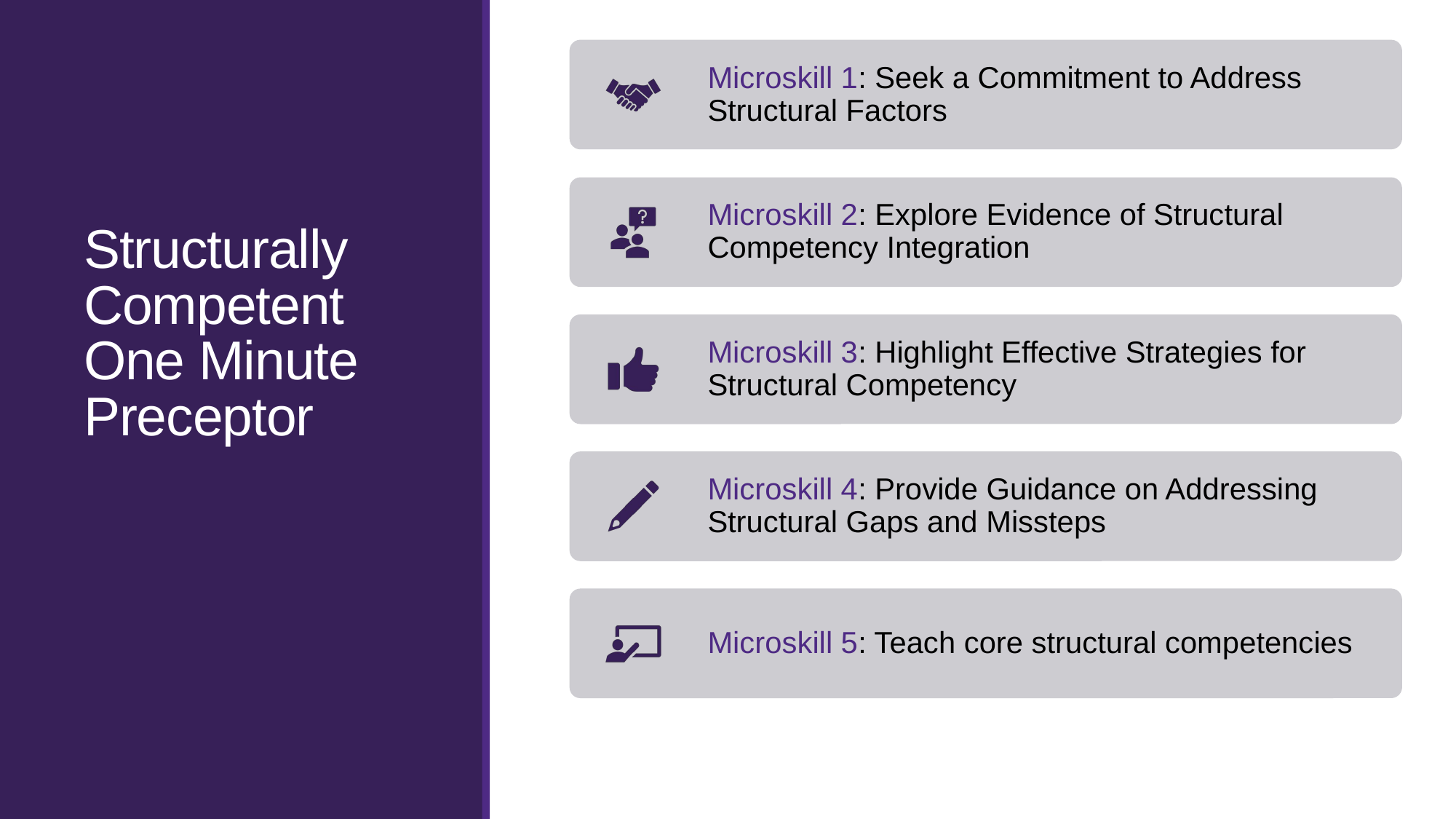

# Structurally Competent One Minute Preceptor

## Slide 68
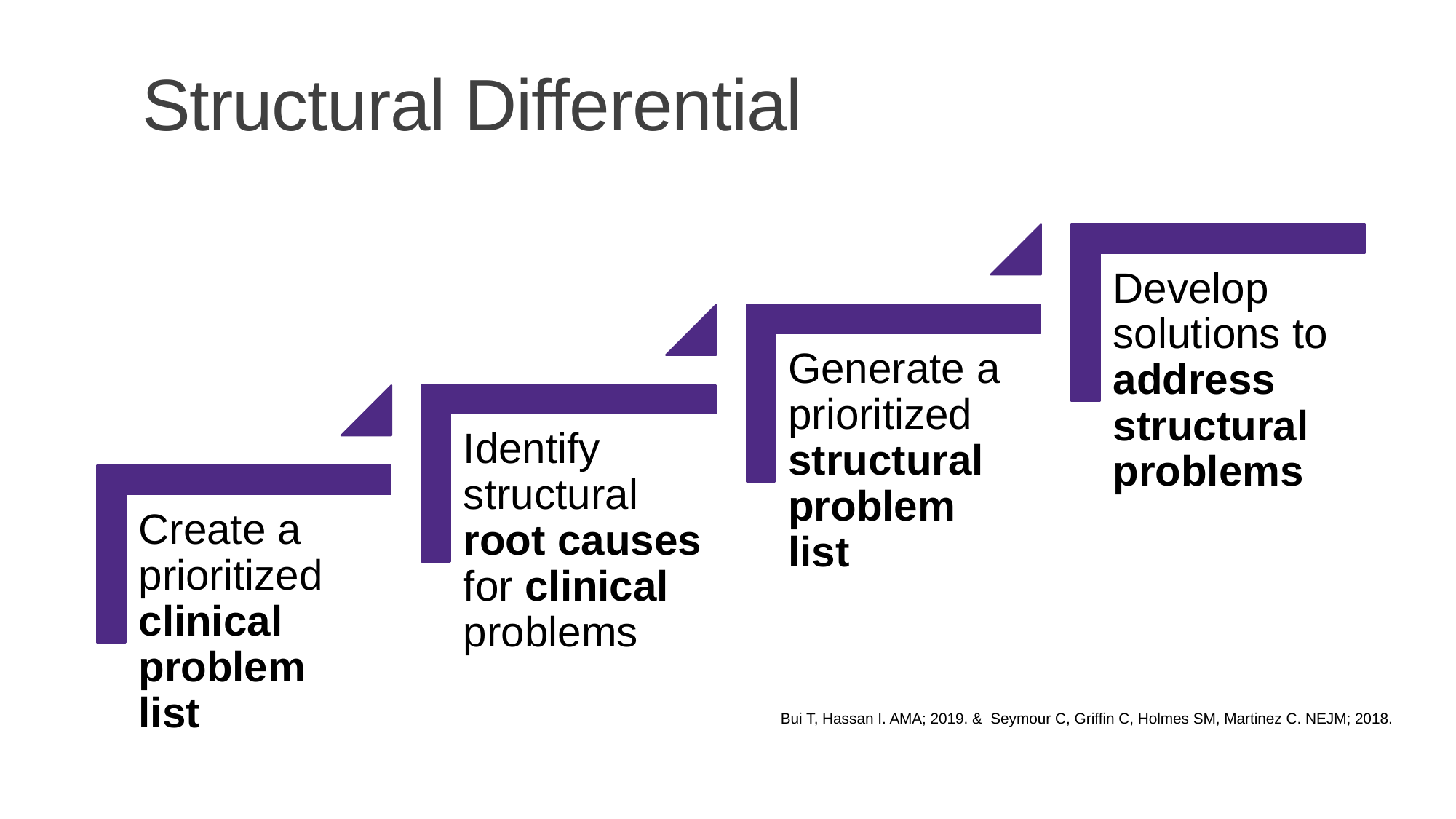

# Structural Differential
Bui T, Hassan I. AMA; 2019. & Seymour C, Griffin C, Holmes SM, Martinez C. NEJM; 2018.

## Slide 69
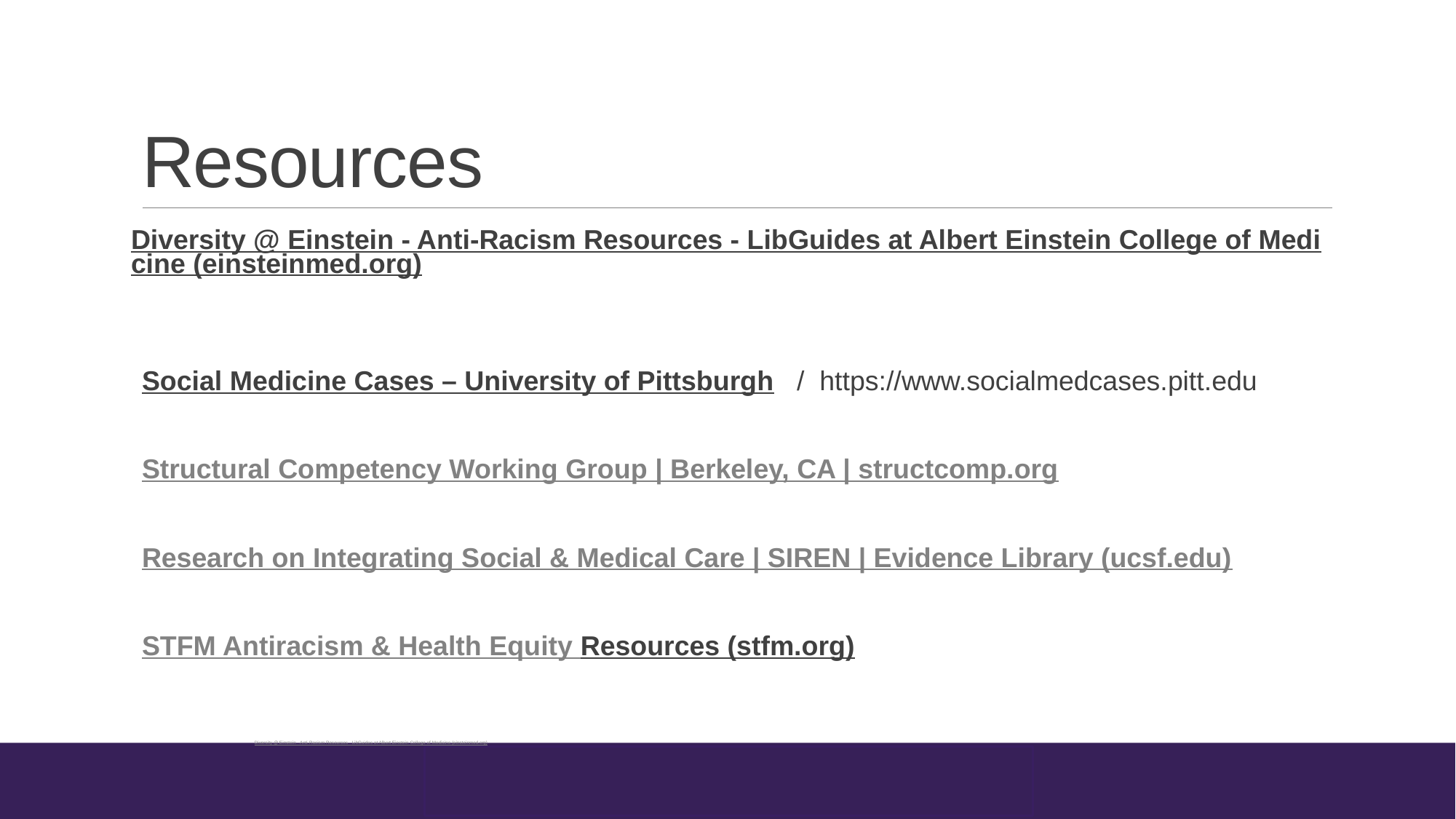

# Resources
Diversity @ Einstein - Anti-Racism Resources - LibGuides at Albert Einstein College of Medicine (einsteinmed.org)
Social Medicine Cases – University of Pittsburgh	/ https://www.socialmedcases.pitt.edu
Structural Competency Working Group | Berkeley, CA | structcomp.org
Research on Integrating Social & Medical Care | SIREN | Evidence Library (ucsf.edu)
STFM Antiracism & Health Equity Resources (stfm.org)
Diversity @ Einstein - Anti-Racism Resources - LibGuides at Albert Einstein College of Medicine (einsteinmed.org)
