# Supplementary material for: Structural Competency: A Faculty Development Workshop Series for Anti-racism in Medical Education
Source: MedEdPORTAL. 2025 Feb 7;21:11492. doi: 10.15766/mep_2374-8265.11492 (PMC11802914; doi:10.15766/mep_2374-8265.11492)
Supplement: Supplementary file 1 — 1 - Introduction to SC.pptx1 - Facilitator Guide.docx1 - SC Rubric Handout.docx1 - Sample SC Learning Goals.docx2 - Resident Reports & Case-Based Presentations.pptx2 - Facilitator Guide.docx2 - Structural Differential Handout.docx2 - Small-Group Handout.docx3 - Demystifying SC.pptx3 - Facilitator Guide.docx3 - SC One-Minute Preceptor Handout.docx3 - SC SNAPPS Handout.docx3 - Role-Play Scenarios.docx4 - SC Hospital-Based Teaching.pptx4 - Facilitator Guide.docx4 - Daily Inpatient Checklist.docx4 - SC Discharge Checklist.docx4 - Small-Group Scenarios.docxPre- and Postsurveys.docx [file mep_2374-8265.11492-s001.zip › J. 3 - Facilitator Guide.docx]

**Appx J- WS 3 SLIDES AND TALKING POINTS**

| **Slide 1**  Welcome Slide Time Check 00:00 | **Big Picture**: Set a welcoming learning environment. |
| --- | --- |
| 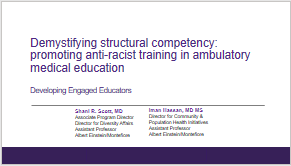 |  |
| *Suggested script/talking points:* Welcome the faculty participants and set a reassuring tone. | |

| **Slide 2**  Learning Objective Overview | **Big Picture:** Brief discussion on what will be discussed over the next ninety minutes |
| --- | --- |
| 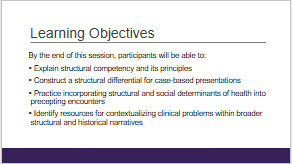 |  |
| *Suggested script/talking points:* Take them through each bullet point briefly. Emphasize that they are going to have specific opportunities to participate and that there will be time for questions at the end. | |

| **Slide 3**  Definition of Structural Competency | | | | | | | | | **Big Picture:** Refresher on the formal definition of structural competency. Repeat slide to reinforce critical concepts. |  |
| --- | --- | --- | --- | --- | --- | --- | --- | --- | --- | --- |
| 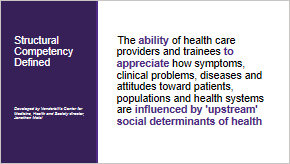 | | | | | | | | |  |  |
| *Suggested script/talking points:*  Structural competency refers to the trained ability to recognize and respond to health and illness as the downstream effects of broad social, economic, and political structures. Unlike cultural competency, which focuses on individual beliefs and behaviors, structural competency emphasizes understanding how systemic factors—such as policies, institutional practices, and social norms—shape health outcomes. This concept encourages healthcare professionals to consider these larger structural influences when diagnosing, treating, and preventing illness, ultimately aiming to address and reduce health disparities. | | | | | | | | | |  |
| **Slide 4**  Definition of Structural Competency | | | | | | | | | **Big Picture**: Rearticulation of structural competency. Repeat slide to reinforce critical concepts. |  |
| 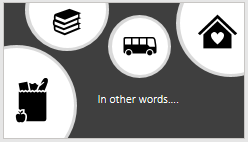 | | | | | | | | |  |  |
| *Suggested script/talking points:*  In other words structural competency is the ability of a healthcare professional to understand that a patient’s access to quality housing, education, food, jobs, and transportation are upstream influences on the clinical manifestation of disease within a person and the relationship between the patient, provider, and the health system they reside within. | | | | | | | | | |  |
|  | | | | | | | | | |  |
| **Slide 5**  Influence of SDoH on Health Outcomes | | | | | | | | | **Big Picture:** SDoH influence the environmental context of an individual. Repeat slide to reinforce critical concepts. |  |
| 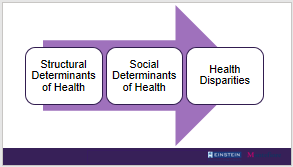 | | | | | | | | |  |  |
| *Suggested script/talking points:*  Structural Determinants of Health and the “social and political mechanism[s] that generate, configure and maintain social hierarchies”, for example, racism, labor markets and political institutions. These in turn impact the conditions in which people are *born, grow, work, live, and age,* also known as the Social Determinants of Health. Examples include economic stability, neighborhood and physical environment, education, food, community and social context, and health care system. Social determinants of health contribute to health outcomes and to the health disparities we see across social hierarchies, ex. across races/ethnicities. | | | | | | | | | |  |
| **Slide 6**  Structural Competency Pillars | | | | | | | | | **Big Picture:** Brief discussion on structural competency pillars. Repeat slide to reinforce critical concepts. |  |
| 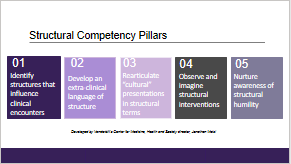 | | | | | | | | |  |  |
| *Suggested script/talking points:*  When teaching through the framework of structural competency physicians should evaluate their learners base on these four objectives. (Read Slide)  Understand structure via sociology, urban planning, and economics. Recasting case presentations to acknowledge these structural barriers to health  Develop interventions to address health infrastructures  Nurture a critical awareness of structural humility | | | | | | | | | |  |
|  | | | | | | | | |  |  |
| **Slide 7**  Importance of Structural Competency | | | | | | | | | **Big Picture**: Explains how structural competency is a vital skill for providers. Repeat slide to reinforce critical concepts. |  |
| 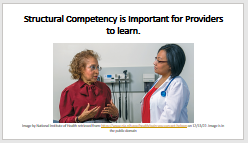 | | | | | | | | |  |  |
| *Suggested script/talking points:*  Structural competency is a critical skill to provide effective health services, cultivate a sense of fulfillment in health care professionals, and advocate to create structural change to support healthier patient communities. | | | | | | | | | |  |
|  | | | | | | | | | |  |
| **Slide 8**  Quiz. Time Check 07:00 minutes | | | | | | | | | **Big Picture**: Check understanding of structural competency. Spend 3 minutes |  |
| 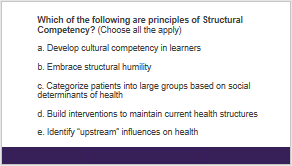 | | | | | | | | |  |  |
| *Suggested script/talking points:*  The following quiz was administered via Zoom Poll:  Name “upstream” contributors to health outcomes (check all that apply)   1. **Neighborhood Redlining** 2. Alcohol consumption 3. **Lack of green spaces** 4. Cigarette Smoking 5. Member of minority race/ethnicity | | | | | | | | | |  |
| **Slide 9**  Quiz | | | | | | | | | **Big Picture**: Check understanding of structural competency |  |
| 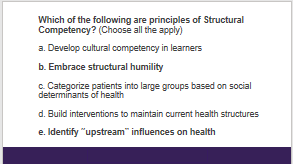 | | | | | | | | |  |  |
| *Suggested script/talking points:*  The following quiz was administered via Zoom Poll:  Name “upstream” contributors to health outcomes (check all that apply)   1. **Neighborhood Redlining** 2. Alcohol consumption 3. **Lack of green spaces** 4. Cigarette Smoking 5. Member of minority race/ethnicity | | | | | | | | | |  |
| **Slide 10**  Traditional Precepting Skit | | | | | | | | | **Big Picture**: |  |
| 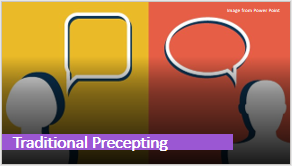 | | | | | | | | | The section following this is designed as a guided role play between two presenters, one presenter acting as the preceptor and the other acting as the learner. |  |
| *Suggested script/talking points:*  Including a historical context in medical education is crucial to addressing health inequities. The disparities we observe in chronic disease morbidity and mortality have deep roots dating back to the establishment of the medical profession. Healthcare professionals must become aware of this aspect of medical history that currently hinders their ability to meet the needs of marginalized communities. | | | | | | | | | |  |
| **Slide 11**  Example of traditional precepting scenario | | | | | | | | | **Big Picture**: Read skit that exemplifies normalized precepting encounter |  |
| 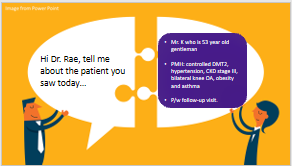 | | | | | | | | |  |  |
| *Suggested script/talking points:*  Preceptor: Hi Dr. Rae, tell me about the patient you saw today.  Learner: Thanks Dr. Sun. I saw Mr. K who is 53 years old gentleman and has a past medical history of controlled diabetes mellitus type II, hypertension, chronic kidney disease stage III, bilateral knee osteoarthritis, obesity and asthma who is presenting for a follow-up visit. | | | | | | | | | |  |
|  | | | | | | | | | |  |
| **Slide 12**  Example of traditional precepting scenario | | | | | | | | | **Big Picture**: Read skit |  |
| 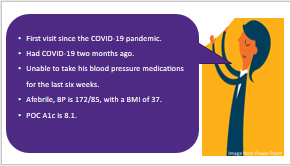 | | | | | | | | |  |  |
| *Suggested script/talking points:*  Learner: This is his first visit in nine months due to the COVID-19 pandemic. He had COVID-19 two months ago but has since recovered. He reports he has been unable to take his blood pressure medications for the past month and half. His blood pressure today is 172/85, his BMI is 37, and he is afebrile. His lung and heart exam are normal. His point-of-care A1c is 8.1. | | | | | | | | | |  |
| **Slide 13**  Example of traditional precepting scenario | | | | | | | | | **Big Picture**: Read Skit |  |
| 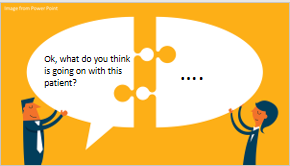 | | | | | | | | |  |  |
| *Suggested script/talking points:*  Preceptor: Ok, what do you think is going on with this patient? | | | | | | | | | |  |
| **Slide 14**  Example of traditional precepting scenario | | | | | | | | | **Big Picture**: Read Skit |  |
| 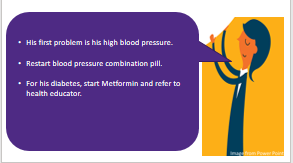 | | | | | | | | |  |  |
| *Suggested script/talking points:*  Learner: I think this patient has a few things going on. His first problem is his high blood pressure. I would like to restart him on his blood pressure combination pill. For his diabetes, his A1c is now above goal so I would like to start Metformin and refer him to our health educator for weight loss counseling. | | | | | | | | | |  |
| **Slide 15**  Example of traditional precepting scenario | | | | | | | | | **Big Picture**: Read Skit |  |
| 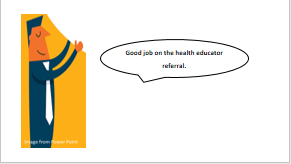 | | | | | | | | |  |  |
| *Suggested script/talking points:* Read slide | | | | | | | | | |  |
| **Slide 16**  Example of traditional precepting scenario | | | | | | | | | **Big Picture**: Read Skit |  |
| 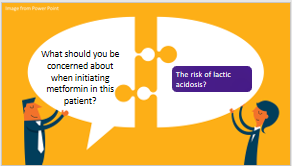 | | | | | | | | |  |  |
| *Suggested script/talking points:*  Preceptor: What should you be concerned about when initiating metformin in this patient?  Learner: the risk of lactic acidosis? | | | | | | | | | |  |
| **Slide 17**  Example of traditional precepting scenario | | | | | | | | | **Big Picture:** Read Skit |  |
| 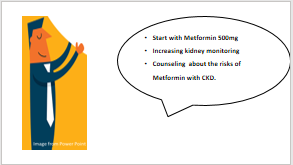 | | | | | | | | |  |  |
| *Suggested script/talking points:*  Preceptor:  Yes! Given this patient’s GFR, I would suggest starting with Metformin 500mg up-titrating to twice daily as tolerated, while increasing kidney monitoring and counseling your patient about the risks of metformin with CKD. | | | | | | | | | |  |
| **Slide 18**  Small Group Discussion Time Check 18:00 minutes | | | | | | | | | **Big Picture**: Through discussions, faculty members can enhance their communication, critical thinking, and problem-solving skills. Spend 8 minutes. |  |
| 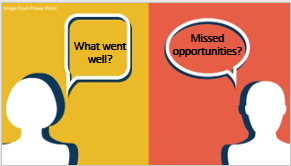 | | | | | | | | |  |  |
| *Suggested script/talking points:*  **Large group discussion:**  Allow raised hands and Zoom chat responses. We are going to be able to dive into this together. (use silence as a cue to motivate)  What went well?  Possible answers: preceptor reinforced what was done well, learner referred to a health educator, etc.  What were some missed opportunities?  Possible answers: probing further into why patient had not been seen in so long, why patient was not taking medications, how the COVID-19 pandemic affected this patient, etc. | | | | | | | | | |  |
| **Slide 19**  Transition to Adapted One Minute Preceptor | | | | | | | | | **Big Picture**: Intro to adapted one-minute preceptor. Spend three minutes |  |
| 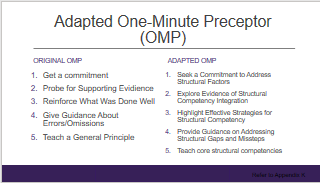 | | | | | | | | |  |  |
| *Suggested script/talking points:*  **Reference Appendix K**  On this slide, we’re comparing the original SNAPPS model with the adapted Structurally Competent One-Minute Preceptor model. This comparison highlights how each approach addresses clinical teaching and reasoning, with an emphasis on integrating structural competency into the One-Minute Preceptor framework.  The adapted model incorporates structural competency into each step, ensuring that learners not only understand clinical concepts but also appreciate the broader social and structural contexts affecting patient care. This integration enhances their ability to provide holistic and equitable care  . | | | | | | | | | |  |
| **Slide 20**  Adapted one minute preceptor Time Check 30:00 minutes | | | | | | | | | **Big Picture**: Explain the teaching framework |  |
| 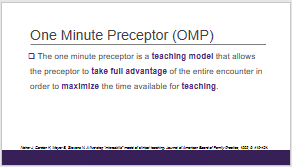 | | | | | | | | |  |  |
| *Suggested script/talking points:*  The teaching encounter will still take longer than a minute but the time spent is more efficiently used and the teaching effectiveness is optimized.  Benefits: The One-Minute Preceptor provides five microskills to organize a learning experience for students in the clinical environment.  Limitations: Lacks support for faculty to consistently address the social determinants of health and up-stream structural influences on the clinical encounter.  Therefore we have adapted the one-minute preceptor model to be rooted in structural competency in order to address its current limitations. Starting with its first organizing microskill. | | | | | | | | | |  |
| **Slide 21**  OMP: Microskill 1 | | | | | | | | | **Big Picture**: Explain the skills that can be cultivated in the learner to strengthen structurally competent clinical reasoning |  |
| 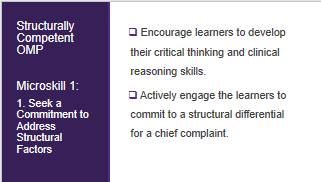 | | | | | | | | |  |  |
| *Suggested script/talking points:*  On this slide, I want to clarify that the microskills we’re discussing are integral components of the Overall Model of Practice (OMP). These microskills are specific techniques that contribute to implementing the OMP effectively.  **Big Picture:** These skills can be cultivated in learners to enhance structurally competent clinical reasoning. By developing these microskills, learners can better address and integrate structural factors into their clinical practice. This strengthening of clinical reasoning helps ensure that practice is both comprehensive and responsive to the broader social and structural context of patient care. | | | | | | | | | |  |
| **Slide 22**  OMP: Microskill 1 | | | | | | | | | **Big Picture**: Review verbal prompts that can be used in learning encounter |  |
| 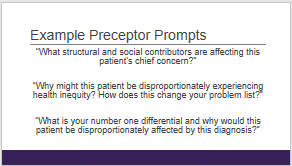 | | | | | | | | |  |  |
| *Suggested script/talking points:*  On this slide, we’ll review various verbal prompts that can be used during learning encounters. These prompts are designed to facilitate deeper engagement and reflection, helping learners better understand and apply the concepts discussed.  Verbal prompts can guide learners to think critically, clarify their understanding, and apply their knowledge in practical scenarios. By incorporating these prompts, we can create a more interactive and effective learning environment.  As we go through each prompt, consider how it can be adapted to fit different learning contexts and how it can support learners in developing their skills further. | | | | | | | | | |  |
| **Slide 23**  OMP: Microskill 2 | | | | | | | | | **Big Picture**: Explain the skills that can be cultivated in the learner to strengthen structurally competent clinical reasoning |  |
| 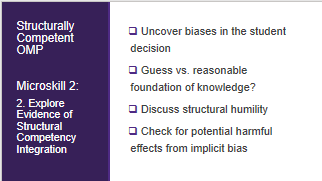 | | | | | | | | |  |  |
| *Suggested script/talking points:*  Uncover biases in the student decision. Was it a guess or was it based on a reasonable foundation of knowledge? Be sure to discuss structural humility in decision making and check for potential harmful effects from implicit bias. | | | | | | | | | |  |
| **Slide 24**  OMP: Microskill 2 | | | | | | | | | **Big Picture**: Review verbal prompts that can be used in learning encounter |  |
| 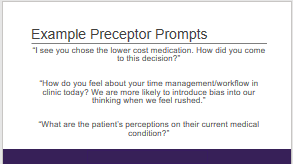 | | | | | | | | |  |  |
| *Suggested script/talking points:*  On this slide, we’ll focus on verbal prompts that can be used to help learners cultivate skills essential for strengthening structurally competent clinical reasoning. These prompts are key tools for guiding learners to think about and integrate structural factors into their clinical practice | | | | | | | | | |  |
| **Slide 25**  OMP: Microskill 3 | | | | | | | | | **Big Picture**: Explain positive reinforcement |  |
| 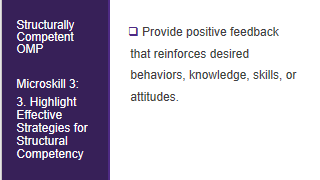 | | | | | | | | |  |  |
| *Suggested script/talking points:*  The learner might not realize they have done something well. Provide positive feedback that reinforces desired behaviors, knowledge, skills, or attitudes. | | | | | | | | | |  |
| **Slide 26**  OMP-Microskill 3 | | | | | | | | | **Big Picture**: Provide example phrases to be included in teaching encounter |  |
| 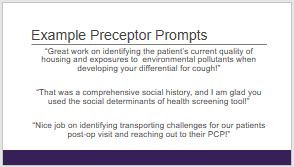 | | | | | | | | |  |  |
| *Suggested script/talking points:*  On this slide, we’ll focus on verbal prompts that can be used to help learners cultivate skills essential for strengthening structurally competent clinical reasoning. These prompts are key tools for guiding learners to think about and integrate structural factors into their clinical practice | | | | | | | | | |  |
| **Slide 27**  OMP: Microsckill 4 | | | | | | | | | **Big Picture**: Promptly address gaps during the encounter |  |
| 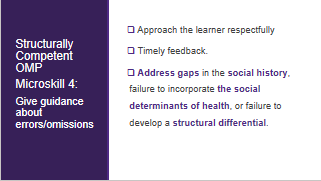 | | | | | | | | |  |  |
| *Suggested script/talking points:*  A structural vulnerability assessment is also another tool that can be used to obtain social history helpful to developing a root cause analysis. Note, this tool explicitly asks about experiences of discrimination. | | | | | | | | | |  |
| **Slide 28**  OMP: Microskill 4 prompts | | | | | | | | | **Big Picture**: Provide example phrases to be included in teaching encounter |  |
| 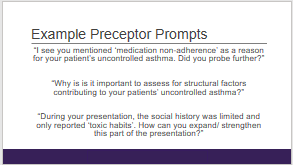 | | | | | | | | |  |  |
| *Suggested script/talking points:*  On this slide, we’ll review verbal prompts designed to help address gaps promptly during a learning encounter. These prompts are crucial for identifying and filling in any missing pieces of information or understanding in real-time | | | | | | | | | |  |
| **Slide 29**  OMP-Microskill 5 | | | | | | | | | **Big Picture**: Pick one of the five core pillars to focus on during the teaching encounter |  |
| 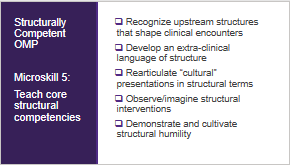 | | | | | | | | |  |  |
| *Suggested script/talking points:*  On this slide, we’ll discuss the importance of selecting one of the five core pillars to focus on during a teaching encounter. Each pillar represents a fundamental aspect of the content we are covering and contributes to a comprehensive understanding of the subject. | | | | | | | | | |  |
| **Slide 30**  OMP-Microskill 5 | | | | | | | | | **Big Picture**: Review five pillars |  |
| 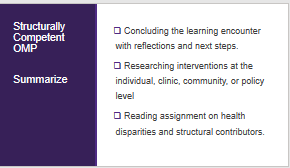 | | | | | | | | |  |  |
| *Suggested script/talking points:*  Consider concluding the learning encounter with reflections and steps. Next steps might include researching interventions at the individual level, clinic level, community level, research or policy level or a reading assignment on health disparities and structural contributors. | | | | | | | | | |  |
| **Slide 31**  OMP- Quiz Time Check 40:00 minutes | | | | | | | | | **Big Picture**: Test understanding of adapted OMP Spend three minutes |  |
| 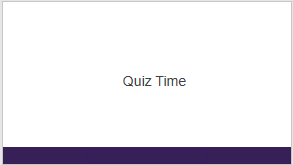 | | | | | | | | |  |  |
| *Suggested script/talking points:*  The following quiz was administered using a Zoom poll:  Which of the following is a structurally competent microskill (check all that apply)   1. **Get a commitment to a structural problem** 2. **Probe and address implicit bias in care plan** 3. Highlight race/ethnicity as a risk factor for chief complaint 4. Allow presentations to condense social history | | | | | | | | | |  |
| **Slide 32**  OMP-Quiz | | | | | | | | | **Big Picture**: Testing understanding of OMP |  |
| 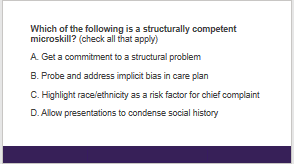 | | | | | | | | |  |  |
| *Suggested script/talking points:*  The following quiz was administered using a Zoom poll:  Which of the following is a structurally competent microskill (check all that apply)   1. **Get a commitment to a structural problem** 2. **Probe and address implicit bias in care plan** 3. Highlight race/ethnicity as a risk factor for chief complaint 4. Allow presentations to condense social history | | | | | | | | | |  |
| **Slide 33**  OMP- Quiz | | | | | | | | | **Big Picture**: Review Quiz answers |  |
| 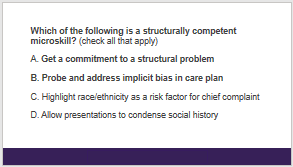 | | | | | | | | |  |  |
| *Suggested script/talking points:*  The following quiz was administered using a Zoom poll:  Which of the following is a structurally competent microskill (check all that apply)   1. **Get a commitment to a structural problem** 2. **Probe and address implicit bias in care plan** 3. Highlight race/ethnicity as a risk factor for chief complaint 4. Allow presentations to condense social history | | | | | | | | | |  |
| **Slide 34**  Introduction to SNAPPS | | | | | | | | | **Big Picture**: Intro to adapted SNAPPS. Spend 3 minutes |  |
| 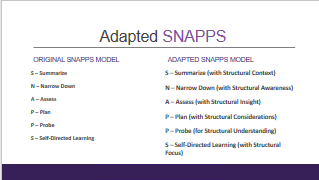 | | | | | | | | |  |  |
| *Suggested script/talking points:*  **Refer to Appendix L**  Welcome to this slide where we introduce the adapted SNAPPS model. As you can see, the slide demonstrates the original SNAPPS steps alongside its adapted counterparts, incorporating structural competency.  **Original SNAPPS Model:**   1. **S – Summarize:** Provide a concise summary of the patient's case. 2. **N – Narrow Down:** Focus on the most likely diagnoses. 3. **A – Assess:** Evaluate the most probable diagnosis. 4. **P – Plan:** Outline the next steps in diagnosis and treatment. 5. **P – Probe:** Explore further details or clarification. 6. **S – Self-Directed Learning:** Reflect on learning needs and seek further knowledge independently.   **Adapted SNAPPS Model:**   1. **S – Summarize (with Structural Context):** Provide a summary of the patient’s case, including relevant structural factors influencing the situation. 2. **N – Narrow Down (with Structural Awareness):** Focus on diagnoses while considering how structural factors might affect the differential. 3. **A – Assess (with Structural Insight):** Evaluate the most probable diagnosis, taking into account structural influences and systemic issues. 4. **P – Plan (with Structural Considerations):** Outline the next steps in diagnosis and treatment, integrating structural factors into the plan. 5. **P – Probe (for Structural Understanding):** Encourage deeper exploration of how structural factors impact the case and the learner’s reasoning. 6. **S – Self-Directed Learning (with Structural Focus):** Promote independent learning with an emphasis on understanding and addressing structural issues.   This adapted model retains the core components of the original SNAPPS but enhances each step by integrating structural competency. This approach ensures that learners develop a comprehensive understanding of both clinical and structural factors, leading to more holistic patient care. | | | | | | | | | |  |
| **Slide 35**  Review teaching points with SNAPPS acronym | | | | | | | | | **Big Picture**: Explain the meaning of “S” in the SNAPPS acronym |  |
| 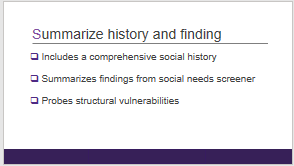 | | | | | | | | |  |  |
| *Suggested script/talking points:*  On this slide, we’ll focus on the meaning of 'S' in the SNAPPS acronym. 'S' stands for **Summarize**.  In this context, summarizing involves presenting a concise overview of the patient's case. This summary should capture the key details, including relevant history, presenting symptoms, and any pertinent findings.  The goal is to distill the information into a brief, focused narrative that highlights the most important aspects of the case. This approach ensures that everyone involved in the discussion has a clear and shared understanding of the patient’s situation before moving on to the next steps.  Effectively summarizing sets the stage for narrowing down the differential diagnosis and planning further actions, making it a crucial first step in the SNAPPS process. | | | | | | | | | |  |
| **Slide 36**  Example of SNAPPS Phrases | | | | | | | | | **Big Picture**: Review SNAPPS phrase |  |
| 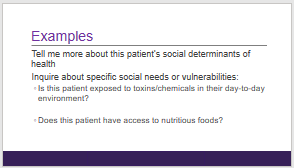 | | | | | | | | |  |  |
| *Suggested script/talking points:*  On this slide, we have examples illustrating the 'S' in SNAPPS, which stands for **Summarize**. Each example demonstrates how to concisely present the essential details of a patient's case | | | | | | | | | |  |
| **Slide 37**  Review teaching points with SNAPPS acronym | | | | | | | | | **Big Picture**: Explain the meaning of “N” in the SNAPPS acronym |  |
| 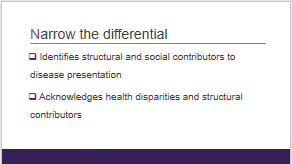 | | | | | | | | |  |  |
| *Suggested script/talking points:*  On this slide, we’re focusing on the meaning of 'N' in the SNAPPS acronym, which stands for **Narrow Down**.  The 'N' step involves narrowing down the differential diagnosis to focus on the most likely possibilities based on the summary provided. This means critically analyzing the patient’s symptoms, history, and initial findings to identify the most probable diagnoses  This step is crucial because it helps prioritize further diagnostic testing and treatment plans, directing your clinical approach towards the most likely causes of the patient’s symptoms. | | | | | | | | | |  |
| **Slide 38**  Example phrases | | | | | | | | | **Big Picture**: Review example phrases to narrow the differential |  |
| 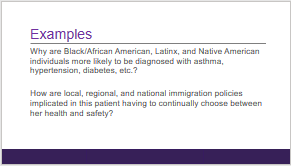 | | | | | | | | |  |  |
| *Suggested script/talking points:*  On this slide, we’ll review example phrases for the 'N' step in SNAPPS, which is to **Narrow Down** the differential diagnosis.  These example phrases are designed to help you focus on the most likely diagnoses based on the patient’s case summary | | | | | | | | | |  |
| **Slide 39**  Review teaching points with SNAPPS acronym | | | | | | | | | **Big Picture**: Explain the meaning of “A” in the SNAPPS acronym |  |
| 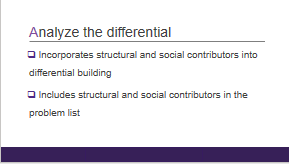 | | | | | | | | |  |  |
| *Suggested script/talking points:*  Now, let’s delve into the meaning of 'A' in the SNAPPS acronym, which stands for **Assess**.  In this context, 'Assess' refers to evaluating the most likely diagnosis or problem identified after narrowing down the differential. This step involves critically analyzing which diagnosis best fits the patient's presentation based on the summary and narrowed differential | | | | | | | | | |  |
| **Slide 40**  Review teaching points with SNAPPS acronym | | | | | | | | | **Big Picture**: Explain the meaning of “A” in the SNAPPS acronym |  |
| 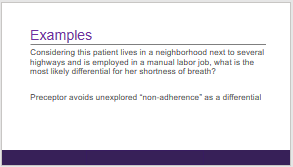 | | | | | | | | |  |  |
| *Suggested script/talking points:*  On this slide, we’ll explore examples that illustrate the 'A' in the SNAPPS acronym, which stands for **Assess**. This step involves evaluating the most likely diagnosis or problem from your narrowed differential.  These examples demonstrate how to apply clinical reasoning to prioritize the most likely diagnosis. By assessing which diagnosis fits best with the patient’s presentation and history, you can streamline your approach to further testing and treatment. | | | | | | | | | |  |
| **Slide 41**  Review teaching points with SNAPPS acronym | | | | | | | | | **Big Picture**: Explain the meaning of “P” in the SNAPPS acronym |  |
| 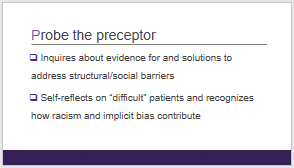 | | | | | | | | |  |  |
| *Suggested script/talking points:*  "Now, let’s focus on the first 'P' in SNAPPS, which stands for **Probe**.  The 'P' step involves probing for further details or clarification about the patient’s condition. This means asking targeted questions to gather additional information that helps refine your differential diagnosis and deepen your understanding of the case.  Effective probing helps in identifying key details that might not be immediately apparent, allowing for a more accurate diagnosis and tailored management plan. It’s a crucial step for ensuring that all relevant aspects of the patient’s condition are considered. | | | | | | | | | |  |
| **Slide 42**  Review teaching points with SNAPPS acronym | | | | | | | | | **Big Picture**: Prompts for the educator to probe for deeper understanding |  |
| 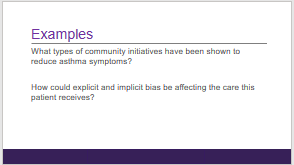 | | | | | | | | |  |  |
| *Suggested script/talking points:*  On this slide, we’ll review several prompts designed to help educators probe for a deeper understanding during learning encounters. These prompts are essential for encouraging learners to think critically and expand their comprehension of the material.  Here are some example prompts  By using these prompts, educators can guide learners towards a more comprehensive and nuanced understanding of clinical scenarios, facilitating deeper engagement and learning. | | | | | | | | | |  |
| **Slide 43**  Review teaching points with SNAPPS acronym | | | | | | | | | **Big Picture**: Review the second “P” in the SNAPPS acronym |  |
| 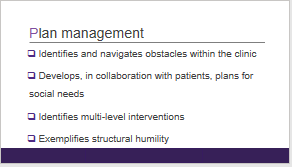 | | | | | | | | |  |  |
| *Suggested script/talking points:*  Now, let’s introduce the meaning of 'P' in the SNAPPS acronym, which stands for **Plan**.  The 'P' step involves outlining the next steps in the diagnostic process or management plan based on your assessment. This includes deciding on further diagnostic tests, treatments, or referrals that are needed to address the patient’s condition effectively.  In essence, the 'P' step is about translating your assessment into actionable steps to manage the patient’s condition. It guides the next phase of patient care and helps ensure that you address the most pressing needs identified during the assessment. | | | | | | | | | |  |
| **Slide 44**  Review teaching points with SNAPPS acronym | | | | | | | | | **Big Picture**: Review prompts from second “P” |  |
| 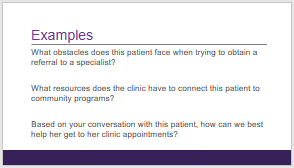 | | | | | | | | |  |  |
| *Suggested script/talking points:*  On this slide, we’ll look at examples for the 'P' step in the SNAPPS acronym, which stands for **Plan**. This step involves outlining the specific actions you will take based on your assessment of the patient’s condition.  Let’s review a couple of examples  These examples show how to translate your clinical assessment into actionable steps. The 'P' step ensures that you address the patient’s needs systematically and effectively, guiding the next stages of care." | | | | | | | | | |  |
| **Slide 45**  Review teaching points with SNAPPS acronym | | | | | | | | | **Big Picture**: Review the last “S” of the SNAPPS acronym |  |
| 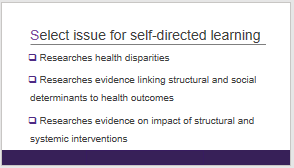 | | | | | | | | |  |  |
| *Suggested script/talking points:*  Let's now discuss the last 'S' in SNAPPS, which stands for **Self-Directed Learning**.  In this context, 'Self-Directed Learning' refers to the process of reflecting on the case and identifying areas where you need to further your knowledge or skills independently. This step encourages learners to take initiative in their own learning journey by exploring additional resources and addressing gaps in their understanding.  Self-Directed Learning is crucial because it fosters lifelong learning habits, encourages curiosity, and empowers learners to take responsibility for their professional development. By embracing this approach, you ensure that your learning continues beyond formal settings and adapts to evolving clinical knowledge and practices. | | | | | | | | | |  |
| **Slide 46**  Review teaching points with SNAPPS acronym | | | | | | | | | **Big Picture**: Review verbal prompts for last “S” acronym |  |
| 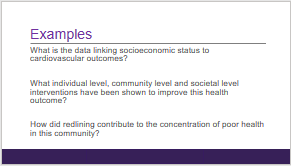 | | | | | | | | |  |  |
| *Suggested script/talking points:*  On this slide, we’re going to look at examples of how to apply **Self-Directed Learning**, the last 'S' in the SNAPPS acronym. This step involves reflecting on your learning needs and taking proactive steps to address them independently.  Here are a few examples to illustrate this concept: on slide  These examples highlight how self-directed learning encourages learners to actively seek out resources, reflect on their learning needs, and engage in continuous improvement. By adopting this approach, learners can take charge of their development and stay updated with the latest clinical knowledge and skills. | | | | | | | | | |  |
| **Slide 47**  Resources | | | | | | | | | **Big Picture**: Structural Differential Steps |  |
| 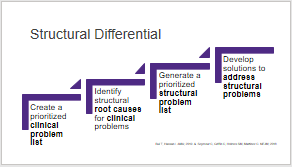 | | | | | | | | |  |  |
| *Suggested script/talking points:*  Some of you may have been wondering, what is a structural differential? A structural differential is a step-by-step guide to incorporating structural competency into clinical reasoning. A structurally competent precepting encounter will guide learners to develop a structural differential.  The steps of the structural differential are:  **Create** a prioritized clinical problem list  Ensure congruity between patient and clinician problem lists.  **Identify** structural root causes for clinical problems  Elicit upstream structural and social determinants of health that contribute to clinical problems.  Integrate historical context.  **Generate** a prioritized structural problem list  Incorporate patient priorities, preferences, and concerns.  Prioritize urgent problems and problems for which clinical and community resources are available.  Prioritize patient priorities, concerns, experiences  **Develop** solutions to address structural problems  Prioritize patient priorities, concerns, experiences  Imagine individual-level, health system-level, community level and population-level solutions.  Consider individual and community strengths/assets.  Partner with an interdisciplinary team participating in community-led efforts | | | | | | | | | |  |
| **Slide 48**  Resources | | | | | | | | | **Big Picture**: Review Clinical Problem List |  |
| 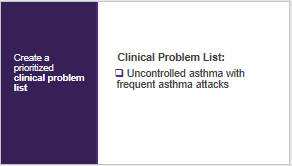 | | | | | | | | |  |  |
| *Suggested script/talking points:*  To give a brief example, one might start with a traditional clinical problem list of a patient with uncontrolled asthma and frequent asthma attacks. | | | | | | | | | |  |
| **Slide 49**  Resources | | | | | | | | | **Big Picture**: Review Root Cause |  |
| 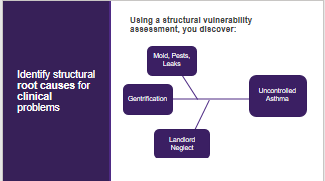 | | | | | | | | |  |  |
| *Suggested script/talking points:*  Using a structural vulnerability assessment, the learner uncovers that their patient has poor housing conditions and numerous triggers in their home such as leaks, mold and pests. The patient’s building has been cited in an increasing number of housing violations as the neighborhood is starting to gentrify. Using this information, the learner can identify the following structural/social root causes: mold/pests/leaks (poor housing conditions), gentrification and landlord neglect. A root cause analysis fishbone diagram can help in identifying root causes. | | | | | | | | | |  |
| **Slide 50** | | | | | | | | | **Big Picture**: Resources for participants |  |
| 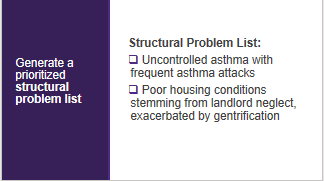 | | | | | | | | |  |  |
| *Suggested script/talking points:*  Here are some health disparities and social determinants of health resources you may find helpful to reference during your teaching or structurally competent case conference presentations. | | | | | | | | | |  |
| **Slide 51**  Resources | | | | | | | | | **Big Picture**: Reviewing structural differential example |  |
| 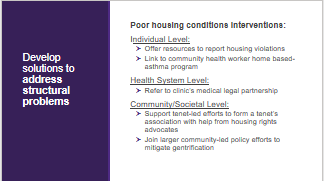 | | | | | | | | |  |  |
| *Suggested script/talking points:*  Solutions can then be developed for this structural problem at the individual, health system and community/societal level. Solutions should draw on the resources available in locally within the healthcare system and the community, be developed in partnership with patients, and exhibit structural humility. | | | | | | | | | |  |
| **Slide 52** | | | | | | | | | **Big Picture**: Quiz to test understanding |  |
| 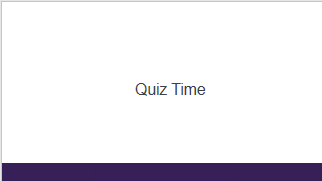 | | | | | | | | |  |  |
| *Suggested script/talking points:*  The following quiz was administered via a Zoom poll:  Which of the following should you do when building a structural differential (select all that apply):  **Integrate historical context of disparities**  **Incorporate patient priorities, preferences, concerns**  Solve most structural problems during the clinic encounter  **Name structural root causes of clinical problems** | | | | | | | | | |  |
| **Slide 53** | | | | | | | | | **Big Picture**: Resources for participants |  |
| 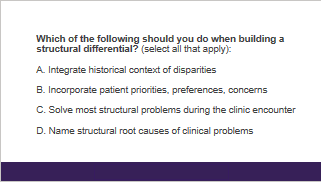 | | | | | | | | |  |  |
| *Suggested script/talking points:*  Which of the following should you do when building a structural differential (select all that apply):  **Integrate historical context of disparities**  **Incorporate patient priorities, preferences, concerns**  Solve most structural problems during the clinic encounter  **Name structural root causes of clinical problems** | | | | | | | | | |  |
| **Slide 54** | | | | | | | | | **Big Picture**: Quiz Spend 3 minutes |  |
| 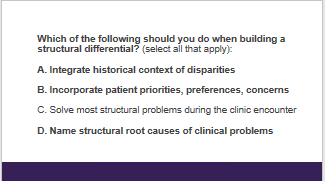 | | | | | | | | |  |  |
| *Suggested script/talking points:*  Which of the following should you do when building a structural differential (select all that apply):  **Integrate historical context of disparities**  **Incorporate patient priorities, preferences, concerns**  Solve most structural problems during the clinic encounter  **Name structural root causes of clinical problems** | | | | | | | | | |  |
| **Slide 55** Small Group Exercise Time Check: 50:00 minutes | | | | | | | | | **Big Picture**: Facilitate small group exercise. Allow 20 minutes for exercise |  |
| 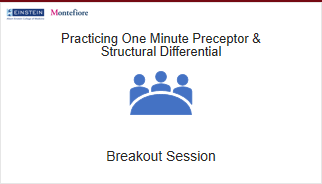 | | | | | | | | |  |  |
| *Suggested script/talking points:*  Although I have introduced two models of precepting, for the sake of time we will focus on the one minute preceptor. I encourage participants to review the SNAPPS model independently. We will now have a breakout session where we will have the opportunity to role play as preceptors, learners and observers and utilize the Structurally Competent One-Minute Preceptor.   - Breakout groups of about 5 people - 1 preceptor, 1 learner, remaining observers - Each member should follow their respective worksheet (below)   **Refer to Appendix G, K, M**  Encourage preceptors to feel comfortable following different leads based on each learner’s needs. Remind them that it’s not always possible to address all structural issues in one session. They should help learners prioritize effectively, even if the learner’s priorities differ from the preceptor’s own. It’s important to be flexible and support learners in focusing on the most relevant issues for their development. | | | | | | | | | |  |
| **Slide 56** | | | | | | | | | **Big Picture**: Facilitate large group discussion |  |
| 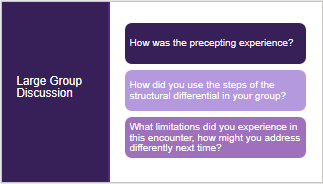 | | | | | | | | |  |  |
| *Suggested script/talking points:*  Following the breakout sessions there is a large group discussion reflecting on the questions listed. Depending on the size of the group, participants can unmute themselves, use the chat or use the hand raise feature and wait to be called. | | | | | | | | | |  |
| **Slide 57**  Structurally competency skit Time Check 75:00 | | | | | | | | | **Big Picture**: Review skit rooted in structurally competent microskills |  |
| 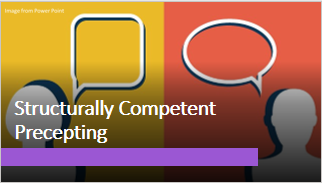 | | | | | | | | |  |  |
| *Suggested script/talking points:*  We will now illustrate a structurally competent version of the case you all worked on in your breakout sessions. You’ll notice that the one minute preceptor microskill appears on the screen as it is utilized in the precepting encounter.  The following slides are meant to be presented as a role play between two presenters (the preceptor and the learner). | | | | | | | | | |  |
| **Slide 58**  Structurally competency skit | | | | | | | | | | **Big Picture**: Skit |
| 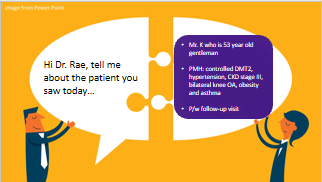 | | | | | | | | | |  |
| *Suggested script/talking points:*  Preceptor:  Dr. Rae, tell me about the patient you saw today  Learner:  Thanks Dr. Sun. I saw Mr. K who is a 53-year-old gentleman and has a past medical history of uncontrolled diabetes mellitus type II, hypertension, chronic kidney disease stage III, bilateral knee osteoarthritis, obesity and asthma who is presenting for a follow-up visit. | | | | | | | | | | |
| **Slide 59**  Structurally competency skit | | **Big Picture**: Review skit rooted in structurally competent microskills | | | | | | | | |
| 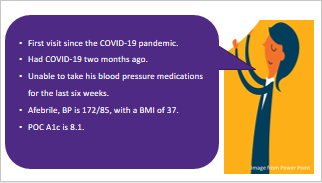 | |  | | | | | | | | |
| *Suggested script/talking points:*  Learner:  This is his first visit in nine months due to the COVID-19 pandemic. He had COVID-19 two months ago but has since recovered. He reports he has been unable to take his blood pressure medications for the past month and half. His blood pressure today is 172/85, his BMI is 37, and he is afebrile. His lung and heart exam are normal. His point-of-care A1c is 8.1. | | | | | | | | | | |
| **Slide 60**  Structurally competency skit | | | | **Big Picture**: Review skit rooted in structurally competent microskills | | | | | | |
| 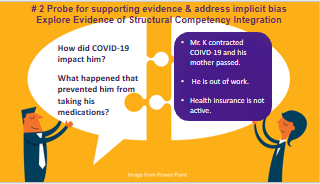 | | | |  | | | | | | |
| *Suggested script/talking points:*  Preceptor: Ok, tell me more about Mr. K’s social situation. How did COVID-19 impact him? What happened that prevented him from taking his medications?  Learner: Mr. K’s household contracted COIVD-19 and his mother passed away from COVID-19 shortly after. He has not been able to find work since he got sick. Mr. K is grieving over the loss of his mom, who was the main source of support for him. It seems almost everyone in his building got sick from COVID-19. He expressed really wanting to take care of himself and his health now that his mom is gone. However, he’s been told his health insurance is not active, and now he is worried about paying for his medications. | | | | | | | | | | |
| **Slide 61**  Structurally competency skit | | | | | | | **Big Picture**: Review skit rooted in structurally competent microskills | | | |
| 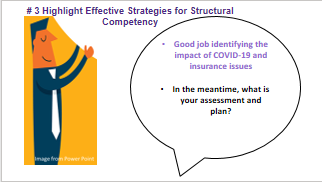 | | | | | | |  | | | |
| *Suggested script/talking points:*  Preceptor: Ok, that’s helpful to know and include in your presentation. Good job identifying the impact COVID-19 has had on Mr. K and the insurance issues he is facing.  What is your assessment and plan? | | | | | | | | | | |
| **Slide 62**  Structurally competency skit | | | **Big Picture**: Review skit rooted in structurally competent microskills | | | | | | | |
| 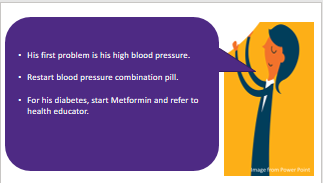 | | |  | | | | | | | |
| *Suggested script/talking points:*  Learner: I think this patient has a few things going on. His first problem is his high blood pressure. I would like to restart him on his blood pressure combination pill. For his diabetes, his A1c is now above goal so I would like to start Metformin and refer him to our health educator for weight loss counseling. | | | | | | | | | | |
| **Slide 63**  Structurally competency skit | | | | | **Big Picture**: Review skit rooted in structurally competent microskills | | | | | |
| 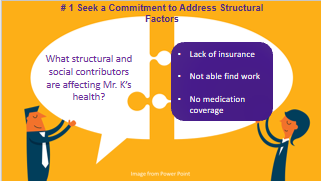 | | | | |  | | | | | |
| *Suggested script/talking points:*  Preceptor: Great, you and Mr. K identified high blood pressure and diabetes as being clinical conditions you would like to address with Mr. K today.  What structural and social contributors are affecting Mr. K’s health?  Learner: Well, his lack of insurance and not being able to find work. I guess his lack of insurance is making him unable to get his medications. | | | | | | | | | | |
| **Slide 64**  Structurally competency skit | | | | | | **Big Picture**: Review skit rooted in structurally competent microskills | | | | |
| 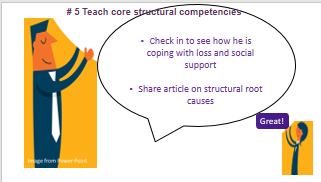 | | | | | |  | | | | |
| *Suggested script/talking points:*  We will now illustrate a structurally competent version of the case you all worked on in your breakout sessions. You’ll notice that the one minute preceptor microskill appears on the screen as it is utilized in the precepting encounter.  The following slides are meant to be presented as a role play between two presenters (the preceptor and the learner). | | | | | | | | | | |
| **Slide 65**  Structurally competency skit | | | | | | | **Big Picture**: Review skit rooted in structurally competent microskills | | | |
| 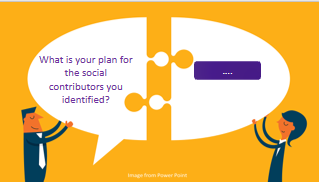 | | | | | | |  | | | |
| *Suggested script/talking points:*  We will now illustrate a structurally competent version of the case you all worked on in your breakout sessions. You’ll notice that the one minute preceptor microskill appears on the screen as it is utilized in the precepting encounter.  The following slides are meant to be presented as a role play between two presenters (the preceptor and the learner). | | | | | | | | | | |
| **Slide 66**  Structurally competency skit | | | | | | | | **Big Picture**: Review skit rooted in structurally competent microskills | | |
| 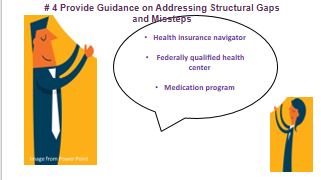 | | | | | | | |  | | |
| *Suggested script/talking points:*  We will now illustrate a structurally competent version of the case you all worked on in your breakout sessions. You’ll notice that the one minute preceptor microskill appears on the screen as it is utilized in the precepting encounter.  The following slides are meant to be presented as a role play between two presenters (the preceptor and the learner). | | | | | | | | | | |
| **Slide 67** | | | | | | | | | **Big Picture**: Overview microskills | |
| 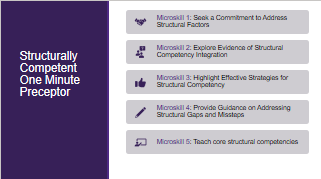 | | | | | | | | |  | |
| *Suggested script/talking points:*  On this slide, we’re summarizing the microskills used in the Structurally Competent One-Minute Preceptor approach. These microskills are key components that facilitate effective teaching and clinical reasoning within this framework.  By integrating these microskills, the Structurally Competent One-Minute Preceptor approach effectively supports learners in developing their clinical reasoning and practice within a structured and reflective framework | | | | | | | | | | |
| **Slide 68** | | | | | | | | | **Big Picture**: Structural Differntial | |
| 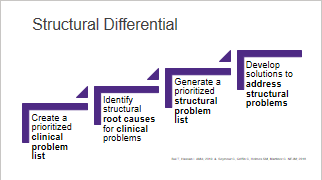 | | | | | | | | |  | |
| *Suggested script/talking points:*  To remind us of the structural differential:  **Create** a prioritized clinical problem list  Ensure congruity between patient and clinician problem lists.  **Identify** structural root causes for clinical problems  Elicit upstream structural and social determinants of health that contribute to clinical problems.  Integrate historical context.  **Generate** a prioritized structural problem list  Incorporate patient priorities, preferences, and concerns.  Prioritize urgent problems and problems for which clinical and community resources are available.  Prioritize patient priorities, concerns, experiences  **Develop** solutions to address structural problems  Prioritize patient priorities, concerns, experiences  Imagine individual-level, health system-level, community level and population-level solutions.  Consider individual and community strengths/assets.  Partner with an interdisciplinary team participating in community-led efforts | | | | | | | | | | |
| **Slide 69**  Resources Time Check 88:00 | | | | | | | | | **Big Picture**: Review resources for structural competency | |
| 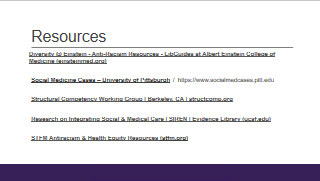 | | | | | | | | |  | |
| *Suggested script/talking points:*  On this slide, we have a curated list of resources that can further support your understanding and application of the concepts we've discussed today  **Diversity at Einstein:** https://libguides.einsteinmed.org/antiracism  **Social Medicine Cases U Pitt:** https://www.socialmedcases.pitt.edu/?page_id=474  **Structural Competency Working Group:** https://www.structcomp.org/  **SIREN:** https://www.structcomp.org/  **STFM Resources:** https://www.stfm.org/about/keyinitiatives/antiracism-and-health-equity/anti-racism/  Feel free to explore these resources at your own pace. They are intended to enhance your learning and provide additional support as you apply these concepts in your practice. If you have any questions or need further recommendations, don’t hesitate to reach out | | | | | | | | | | |
