# Supplementary material for: Structural Competency: A Faculty Development Workshop Series for Anti-racism in Medical Education
Source: MedEdPORTAL. 2025 Feb 7;21:11492. doi: 10.15766/mep_2374-8265.11492 (PMC11802914; doi:10.15766/mep_2374-8265.11492)
Supplement: Supplementary file 1 — 1 - Introduction to SC.pptx1 - Facilitator Guide.docx1 - SC Rubric Handout.docx1 - Sample SC Learning Goals.docx2 - Resident Reports & Case-Based Presentations.pptx2 - Facilitator Guide.docx2 - Structural Differential Handout.docx2 - Small-Group Handout.docx3 - Demystifying SC.pptx3 - Facilitator Guide.docx3 - SC One-Minute Preceptor Handout.docx3 - SC SNAPPS Handout.docx3 - Role-Play Scenarios.docx4 - SC Hospital-Based Teaching.pptx4 - Facilitator Guide.docx4 - Daily Inpatient Checklist.docx4 - SC Discharge Checklist.docx4 - Small-Group Scenarios.docxPre- and Postsurveys.docx [file mep_2374-8265.11492-s001.zip › K. 3 - SC One-Minute Preceptor Handout.docx]

One-Minute Preceptor with Structural Competency

The One-Minute Preceptor teaching method guides the preceptor-learner encounter via five microskills. This method is a brief teaching tool that fosters assessment of trainee knowledge as well as provision of timely feedback. The strengths of this teaching method include: increased involvement with patients, increased clinical reasoning by the learner, and the learner receiving concise, high-quality feedback from the preceptor.

| **Microskills** | |
| --- | --- |
| 1. **Seek a Commitment to Address Structural Factors**   Focus on one learning point. Encourage learners to develop their critical thinking and clinical reasoning skills. Actively engage the learners and push them to make a decision about something, regarding a structural differential for a chief complaint or a plan.  Ex: “What structural contributors to health disparities are potentially affecting this patient, and how does that change the  problem list?” | 1. **Explore Evidence of Structural Competency Integration**   Uncover the basis for the student’s decision - was it a guess or was it based on a reasonable foundation of knowledge? Be sure to discuss structural humility in decision making and check for potential harmful effects from implicit bias.  Ex: “I see you chose the lower cost medication to prescribe. How did you come to this decision? What might you want to discuss with your patient when counseling on  what medication to start?” |
| 1. **Highlight Effective Strategies for Structural Competency**   The learner might not realize they have done something well. Positive feedback reinforces desired behaviors, knowledge, skills, or attitudes.  Ex: “Good job on incorporating the patient’s housing quality and exposure to environmental pollutants in your differential for cough. This will center potential interventions to mitigate recurrence or  progression of the underlying disease.” | 1. **Provide Guidance on Addressing Structural Gaps and Missteps**   Approach the learner respectfully while concurrently addressing areas of need/improvement. Without timely feedback, it is difficult to improve. If mistakes are not pointed out, learners may never discover that they are making these errors and hence repeat them.  Ex: “I see you mentioned ‘medication non- adherence’ as a reason for your patient’s uncontrolled asthma. Did you probe further? Why is it important to assess for structural factors contributing to your patients’  uncontrolled asthma?” |
| **5. Teach Core Structural Competencies**   - Recognizing the structures that shape clinical interactions   - Developing an extra-clinical language of structure - Rearticulating “cultural” presentations in structural terms   - Observing and imagining structural interventions     - Developing structural humility | |
| Summarize:  **Conclude the learning encounter with reflections and steps. Next steps might include researching interventions at the individual level, clinic level, community level, research or policy level or a reading**  **assignment on health disparities and structural contributors.** | |

Adapted by: Scott S. and Hassan I.

Sources: (1) Neher J, Gordon K, Meyer B, Stevens N. A five-step “microskills” model of clinical teaching. Journal of American Board of Family Practice, 1992; 5: 419-424. (2) Metzl JM, Hansen H. Structural competency: theorizing a new medical engagement with stigma and inequality. *Soc Sci Med*. 2014;103:126-133. doi:10.1016/j.socscimed.2013.06.032
