# Supplementary material for: Structural Competency: A Faculty Development Workshop Series for Anti-racism in Medical Education
Source: MedEdPORTAL. 2025 Feb 7;21:11492. doi: 10.15766/mep_2374-8265.11492 (PMC11802914; doi:10.15766/mep_2374-8265.11492)
Supplement: Supplementary file 1 — 1 - Introduction to SC.pptx1 - Facilitator Guide.docx1 - SC Rubric Handout.docx1 - Sample SC Learning Goals.docx2 - Resident Reports & Case-Based Presentations.pptx2 - Facilitator Guide.docx2 - Structural Differential Handout.docx2 - Small-Group Handout.docx3 - Demystifying SC.pptx3 - Facilitator Guide.docx3 - SC One-Minute Preceptor Handout.docx3 - SC SNAPPS Handout.docx3 - Role-Play Scenarios.docx4 - SC Hospital-Based Teaching.pptx4 - Facilitator Guide.docx4 - Daily Inpatient Checklist.docx4 - SC Discharge Checklist.docx4 - Small-Group Scenarios.docxPre- and Postsurveys.docx [file mep_2374-8265.11492-s001.zip › L. 3 - SC SNAPPS Handout.docx]

| **Structural Competency SNAPPS Adaptation for Preceptors** | | | |
| --- | --- | --- | --- |
| **Steps^3^** | Learner Objective Topic | Structure Competency^1^ Adaptation | Example Question Prompts for Preceptor |
| **S**  **Summarize briefly the history and the findings** | Obtains a history, performs a physical examination, and presents a summary of their findings to the preceptor. The summary should be brief and concise and should not utilize more than 50% of the learning encounter (~3 minutes maximum to present) | **Add:** Includes patient’s responses to pertinent questions assessing structural vulnerability from one of the eight domains of the structural vulnerability questionnaire2 in the HPI and/or discusses a social need identified using a validated social needs screener4 | Tell me more about this patient’s social determinants of health.  Does this patient have enough money to live comfortably – pay rent, get food, pay utilities, etc.?2  Is this patient exposed to toxins/chemicals in their day-to-day environment?2  Has this patient been discriminated against due to their race/ethnicity/gender/sexual orientation/religious identity, etc? |
|  |  |  | Does this patient have access to nutritious food? |
| **N**  **Narrow the differential to two or three relevant possibilities** | Provides two to three possibilities of what the diagnosis could be  Presents their list prior to the preceptor revising the list | **Add:** Identifies health disparities and structural contributors to health disparities affecting patients and includes these as part of the differential  Discusses how social, political, and economic structures might be contributing to this patient’s current presentation of illness and how these structures influence pathophysiology | Why are Black/African American, Latinx, and Native American individuals more likely to be diagnosed with asthma, hypertension, diabetes, etc.?  How are local, regional, and national immigration policies implicated in this patient having to continually choose between her health and safety? |
| **A**  **Analyze the differential comparing and contrasting the possibilities** | Discusses the possibilities and analyzes why the patient presentation supports or refutes the differential diagnoses  Thinks out loud in front of the preceptor | **Add:** Identifies health disparities and structural contributors to health disparities the patient is at risk for and how that changes the prioritization of the differential diagnosis | Considering this patient lives in a neighborhood next to several highways and is employed in a manual labor job, where she also experiences additional stress from gender discrimination, what is the most likely differential for her shortness of breath? |
|  |  |  | Preceptor may suggest prioritizing housing-related asthma triggers as a differential for patient’s asthma exacerbation and avoid unexplored “non-adherence” as a differential. |
| **P**  **Probe the preceptor by asking questions about uncertainties, difficulties, or alternative approaches** | Discusses areas of confusion and asks questions of the preceptor  Allows the preceptor to learn about their thinking and knowledge base  Prompts discussion from the preceptor on clinical pearls or areas of importance | **Add:** Inquires about evidence for and solutions to social and structural barriers  Self reflects on experiences treating patients they found “difficult to work with” and recognizes how structural/social factors, including racism, and implicit bias contribute | What types of community initiatives have been shown to reduce asthma symptoms?  How could explicit and implicit bias be affecting the care this patient receives? |

| **P**  **Plans management for the patient’s medical issues** | Discusses a management plan for the patient or outlines next steps  Commits to their plan and utilizes the preceptor as a source of knowledge | **Add:** Appropriately identifies obstacles within the clinic that can influence the management plan  Develops plans for unmet social needs after inquiring about priorities, barriers and past experiences, and in collaboration with patients | What obstacles does this patient face when trying to obtain a referral to a specialist?  What resources does the clinic have to connect this patient to community programs?  Based on your conversation with this patient, how can we best help her get to her clinic appointments? |
| --- | --- | --- | --- |
|  |  | Identifies potential interventions at the individual, interpersonal, clinic/institutional, community, policy and research level | What medications on the prescription program is this patient eligible for? |
|  |  | Learning exemplifies structural humility |  |
| **S**  **Select a case-related issue for self-directed learning** | Identifies a learning issue related to the patient encounter  Discusses the findings from the learning issue with the preceptor | **Add:** Researches data on health disparities  Researches evidence linking structural and social factors, including policies, to health disparities  Researches evidence linking structural and systemic interventions to improvements in health outcomes | What are the data linking socioeconomic status to cardiovascular outcomes?  What individual level, community level and societal level interventions have been shown to improve this health outcome?  How did redlining contribute to the concentration of poor health in this community? |

Developed by: Scott S. and Hassan I. | Montefiore-Einstein **Accompanies WS3 Appendix I Slide 34**

References:

1 Metzl JM, Hansen H. Structural competency: theorizing a new medical engagement with stigma and inequality. *Soc Sci Med*. 2014;103:126-133. doi:10.1016/j.socscimed.2013.06.032

2Bourgois P, Holmes SM, Sue K, Quesada J. Structural Vulnerability: Operationalizing the Concept to Address Health Disparities in Clinical Care. *Acad Med*. 2017;92(3):299-307. doi:10.1097/ACM.0000000000001294

3Wolpaw T, Wolpaw D, Papp K. SNAPPS: A learner-centered model for outpatient education. Academic Medicine. 2003; 78(9): 893- 898. "Teaching Skills for the Preceptor: Learner-Centered Model." The Association of Gynecology and Obstetrics. [www.pnwu.edu/files/4414/2551/7541/Teaching_Skills_for_the_Preceptor_Learner-Centered_Model.pdf.](http://www.pnwu.edu/files/4414/2551/7541/Teaching_Skills_for_the_Preceptor_Learner-Centered_Model.pdf) Accessed August 2016.

4Screening Tools. American Academy of Pediatrics & Screening Time. Accessed 2020 July 22. https://screeningtime.org/star- center/#/screening-tools
