# Supplementary material for: Structural Competency: A Faculty Development Workshop Series for Anti-racism in Medical Education
Source: MedEdPORTAL. 2025 Feb 7;21:11492. doi: 10.15766/mep_2374-8265.11492 (PMC11802914; doi:10.15766/mep_2374-8265.11492)
Supplement: Supplementary file 1 — 1 - Introduction to SC.pptx1 - Facilitator Guide.docx1 - SC Rubric Handout.docx1 - Sample SC Learning Goals.docx2 - Resident Reports & Case-Based Presentations.pptx2 - Facilitator Guide.docx2 - Structural Differential Handout.docx2 - Small-Group Handout.docx3 - Demystifying SC.pptx3 - Facilitator Guide.docx3 - SC One-Minute Preceptor Handout.docx3 - SC SNAPPS Handout.docx3 - Role-Play Scenarios.docx4 - SC Hospital-Based Teaching.pptx4 - Facilitator Guide.docx4 - Daily Inpatient Checklist.docx4 - SC Discharge Checklist.docx4 - Small-Group Scenarios.docxPre- and Postsurveys.docx [file mep_2374-8265.11492-s001.zip › A. 1 - Introduction to SC.pptx]

## Slide 1
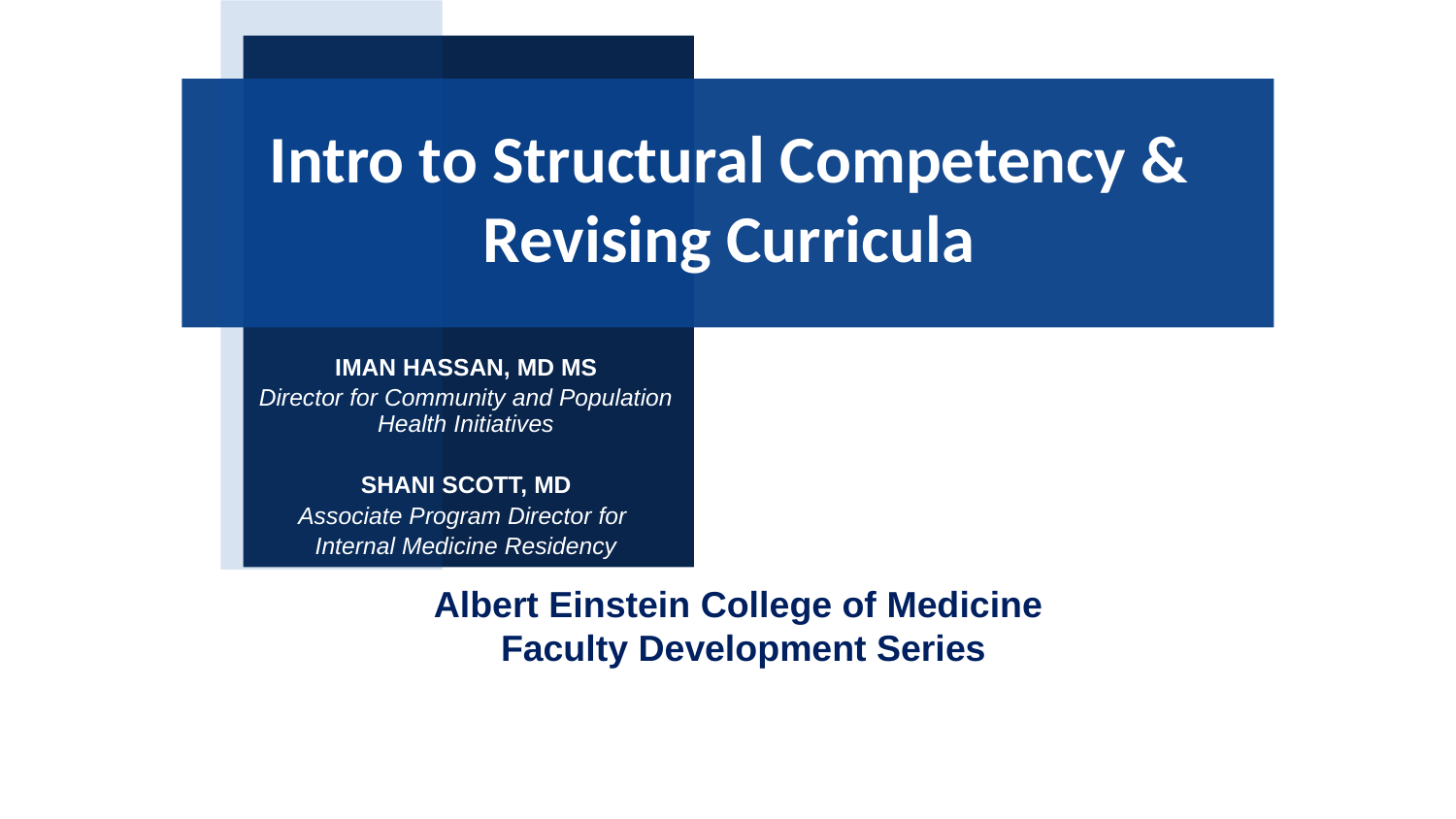

Intro to Structural Competency & Revising Curricula
IMAN HASSAN, MD MS
Director for Community and Population Health Initiatives
SHANI SCOTT, MD
Associate Program Director for
Internal Medicine Residency
Albert Einstein College of Medicine
Faculty Development Series

## Slide 2
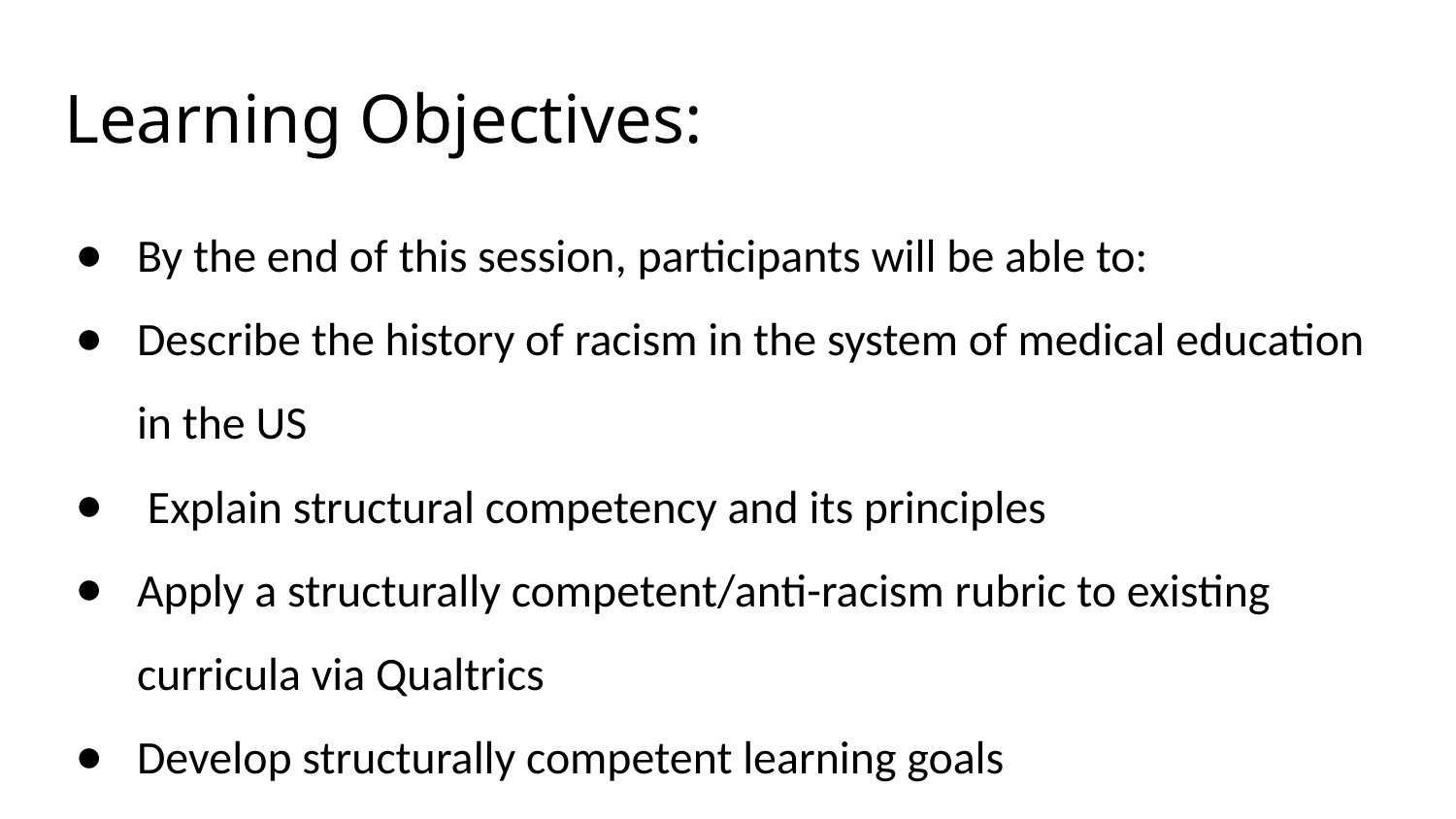

# Learning Objectives:
By the end of this session, participants will be able to:
Describe the history of racism in the system of medical education in the US
 Explain structural competency and its principles
Apply a structurally competent/anti-racism rubric to existing curricula via Qualtrics
Develop structurally competent learning goals

## Slide 3
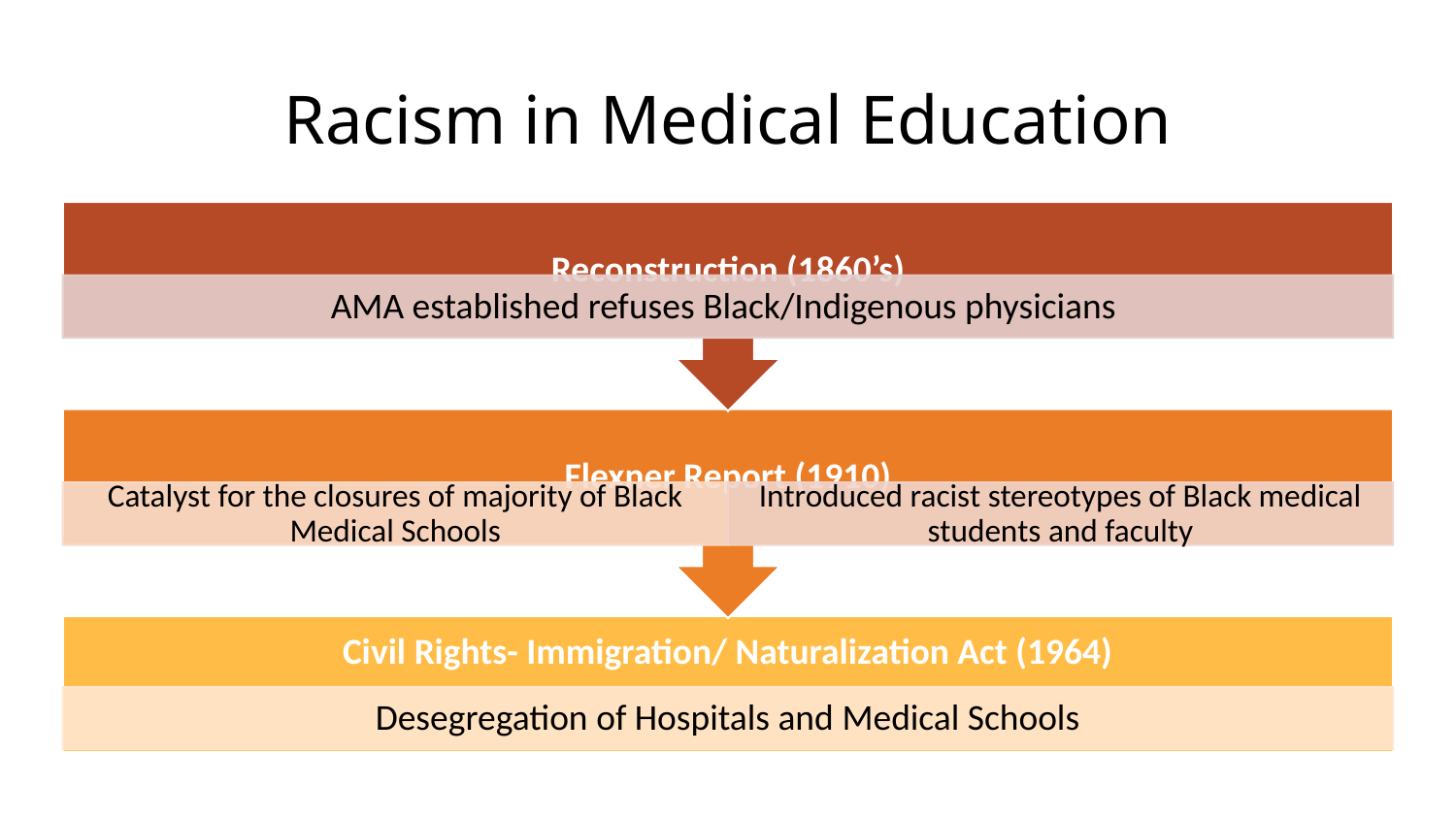

# Racism in Medical Education

## Slide 4
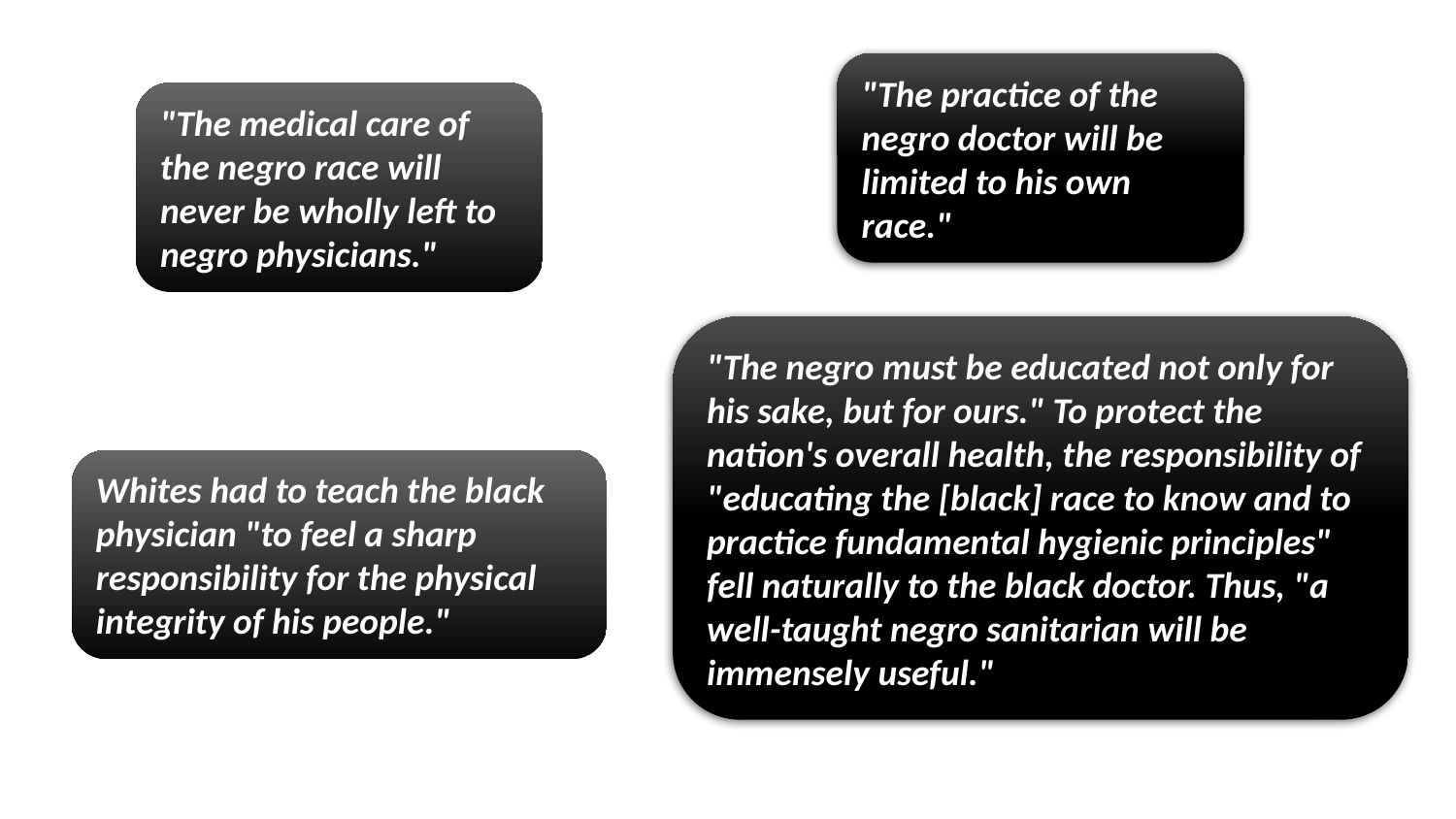

"The practice of the negro doctor will be limited to his own race."
"The medical care of the negro race will never be wholly left to negro physicians."
"The negro must be educated not only for his sake, but for ours." To protect the nation's overall health, the responsibility of "educating the [black] race to know and to practice fundamental hygienic principles" fell naturally to the black doctor. Thus, "a well-taught negro sanitarian will be immensely useful."
Whites had to teach the black physician "to feel a sharp responsibility for the physical integrity of his people."

## Slide 5
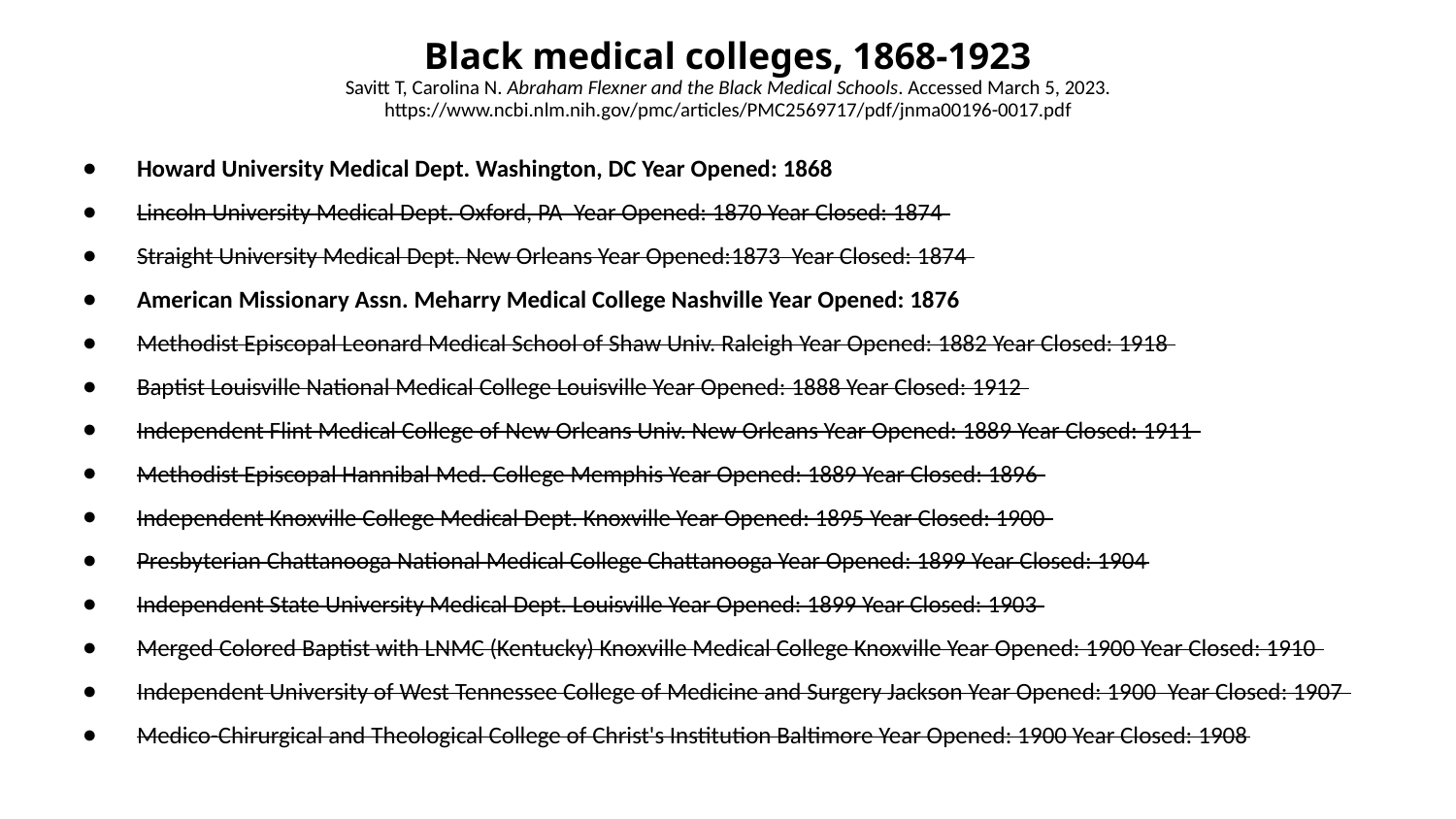

# Black medical colleges, 1868-1923 Savitt T, Carolina N. Abraham Flexner and the Black Medical Schools. Accessed March 5, 2023. https://www.ncbi.nlm.nih.gov/pmc/articles/PMC2569717/pdf/jnma00196-0017.pdf ‌
Howard University Medical Dept. Washington, DC Year Opened: 1868
Lincoln University Medical Dept. Oxford, PA Year Opened: 1870 Year Closed: 1874
Straight University Medical Dept. New Orleans Year Opened:1873 Year Closed: 1874
American Missionary Assn. Meharry Medical College Nashville Year Opened: 1876
Methodist Episcopal Leonard Medical School of Shaw Univ. Raleigh Year Opened: 1882 Year Closed: 1918
Baptist Louisville National Medical College Louisville Year Opened: 1888 Year Closed: 1912
Independent Flint Medical College of New Orleans Univ. New Orleans Year Opened: 1889 Year Closed: 1911
Methodist Episcopal Hannibal Med. College Memphis Year Opened: 1889 Year Closed: 1896
Independent Knoxville College Medical Dept. Knoxville Year Opened: 1895 Year Closed: 1900
Presbyterian Chattanooga National Medical College Chattanooga Year Opened: 1899 Year Closed: 1904
Independent State University Medical Dept. Louisville Year Opened: 1899 Year Closed: 1903
Merged Colored Baptist with LNMC (Kentucky) Knoxville Medical College Knoxville Year Opened: 1900 Year Closed: 1910
Independent University of West Tennessee College of Medicine and Surgery Jackson Year Opened: 1900 Year Closed: 1907
Medico-Chirurgical and Theological College of Christ's Institution Baltimore Year Opened: 1900 Year Closed: 1908

## Slide 6
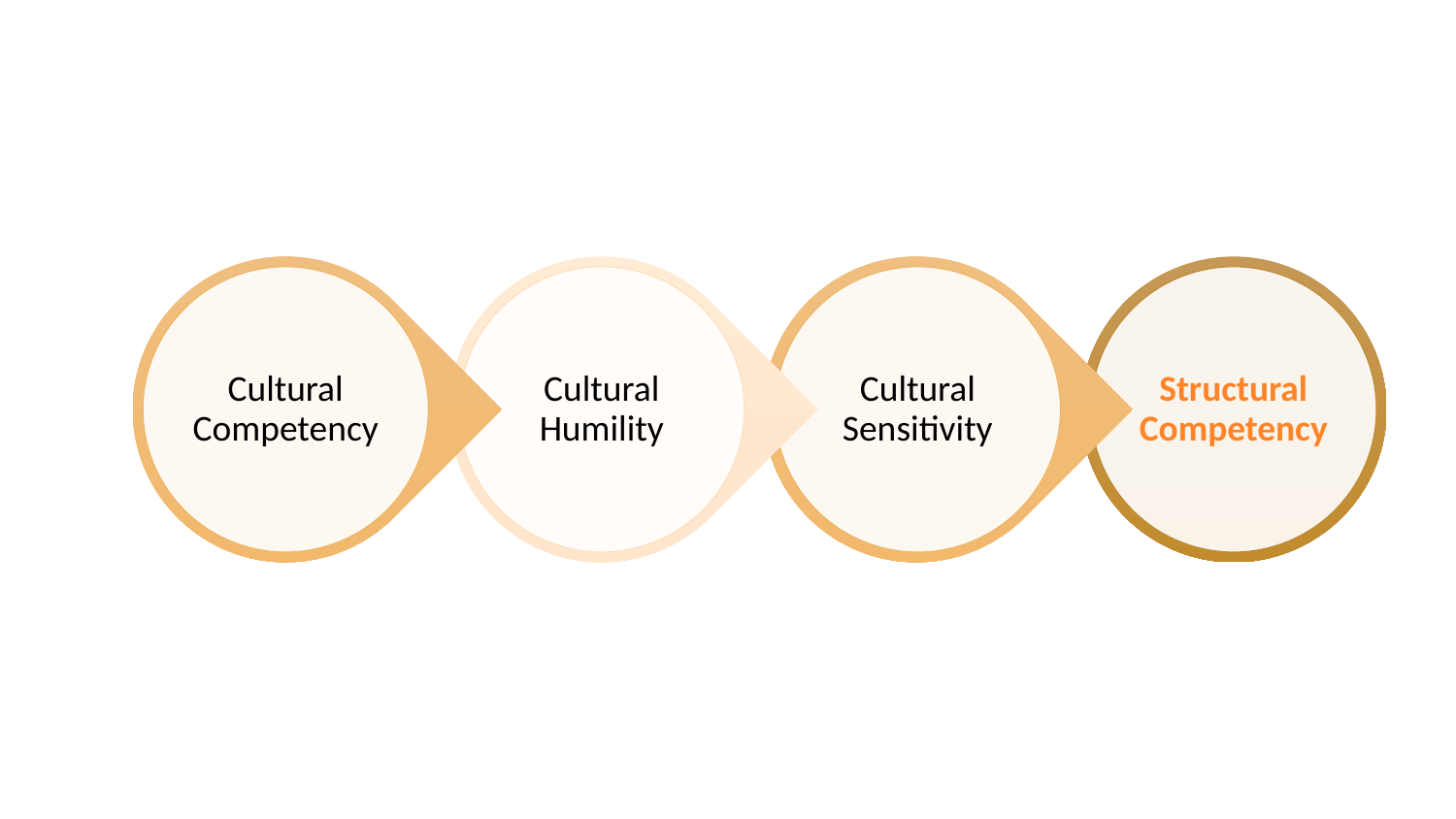

## Slide 7
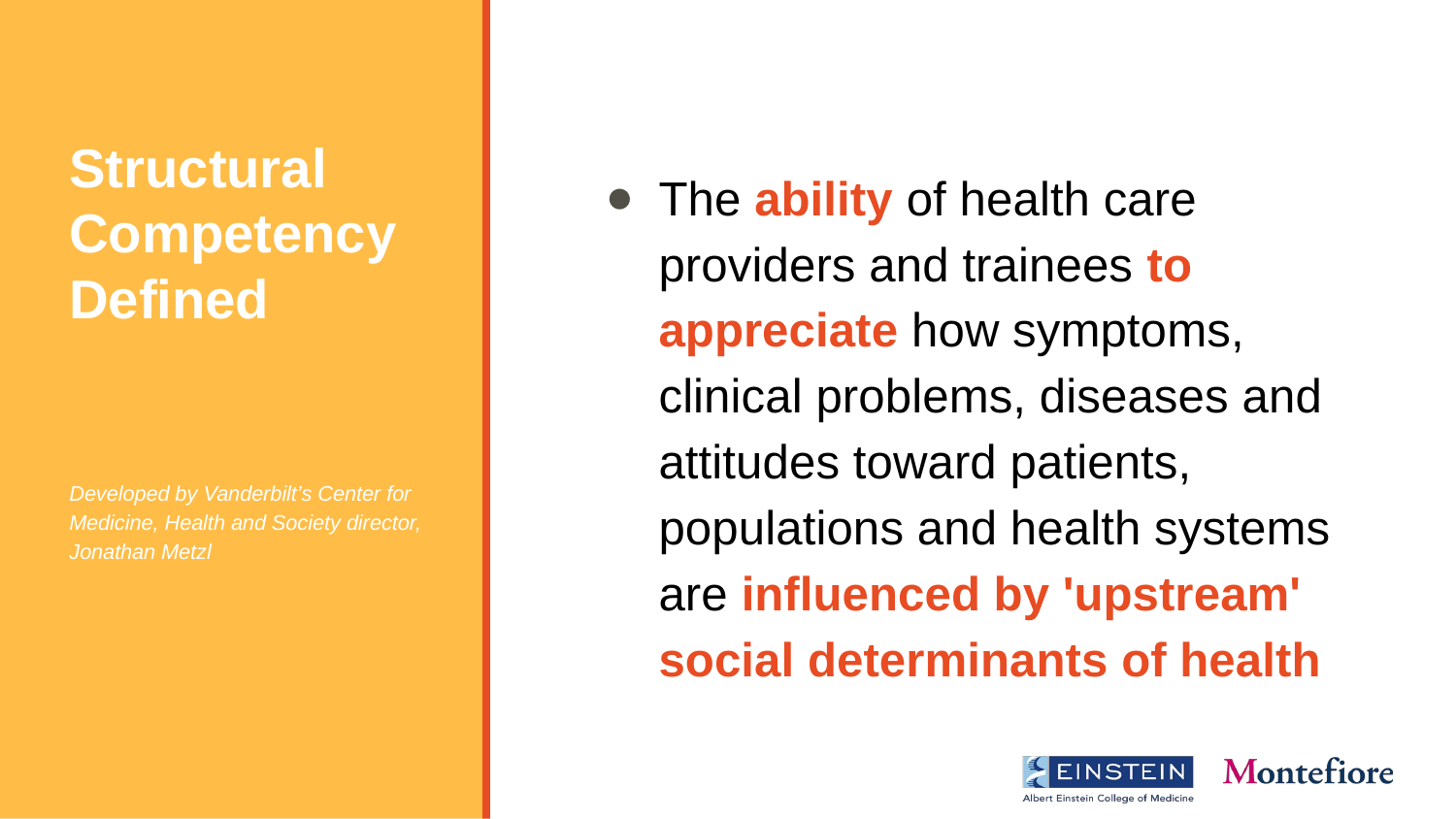

# Structural Competency Defined
The ability of health care providers and trainees to appreciate how symptoms, clinical problems, diseases and attitudes toward patients, populations and health systems are influenced by 'upstream' social determinants of health
Developed by Vanderbilt’s Center for Medicine, Health and Society director, Jonathan Metzl

## Slide 8
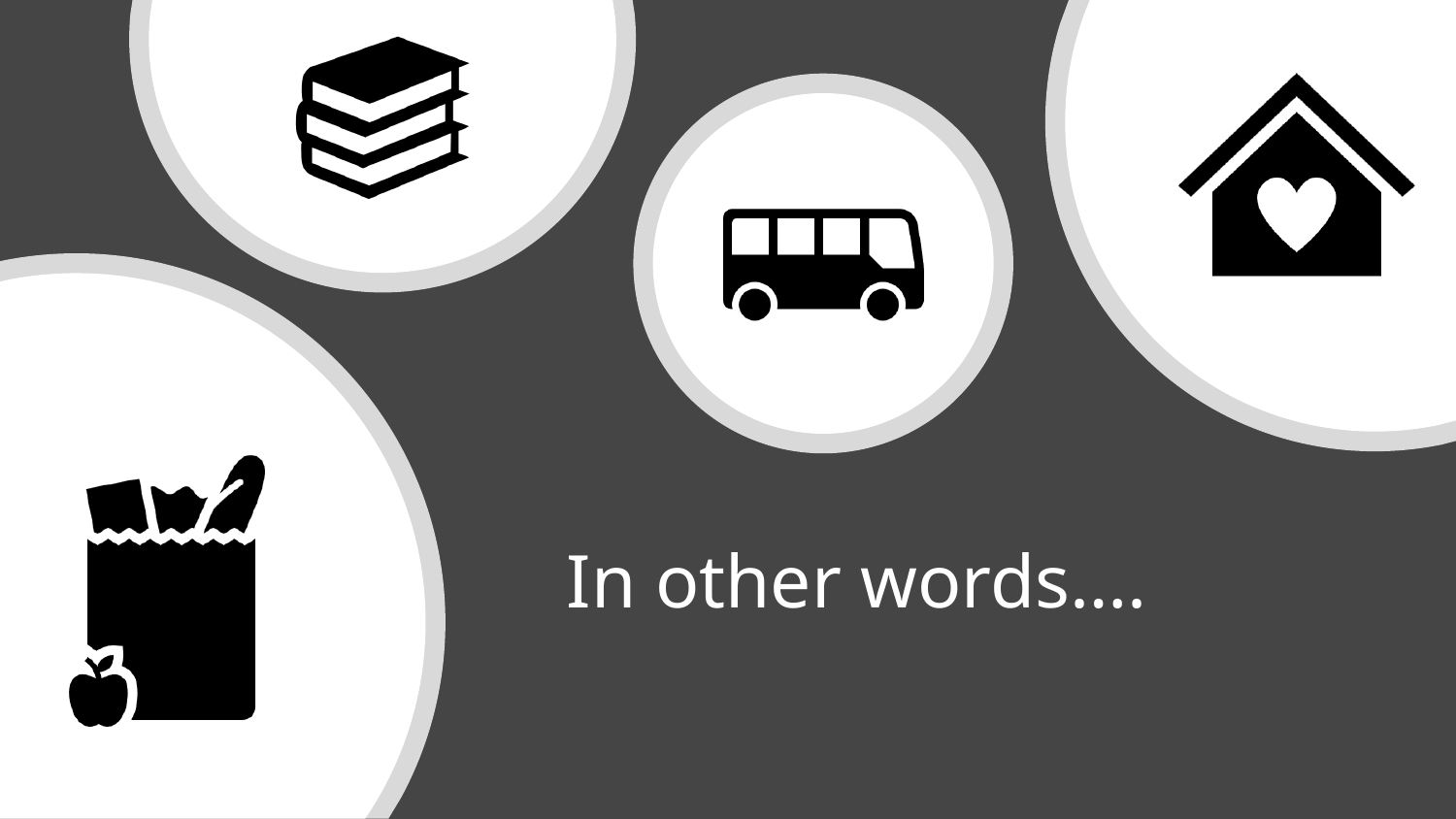

In other words….

## Slide 9
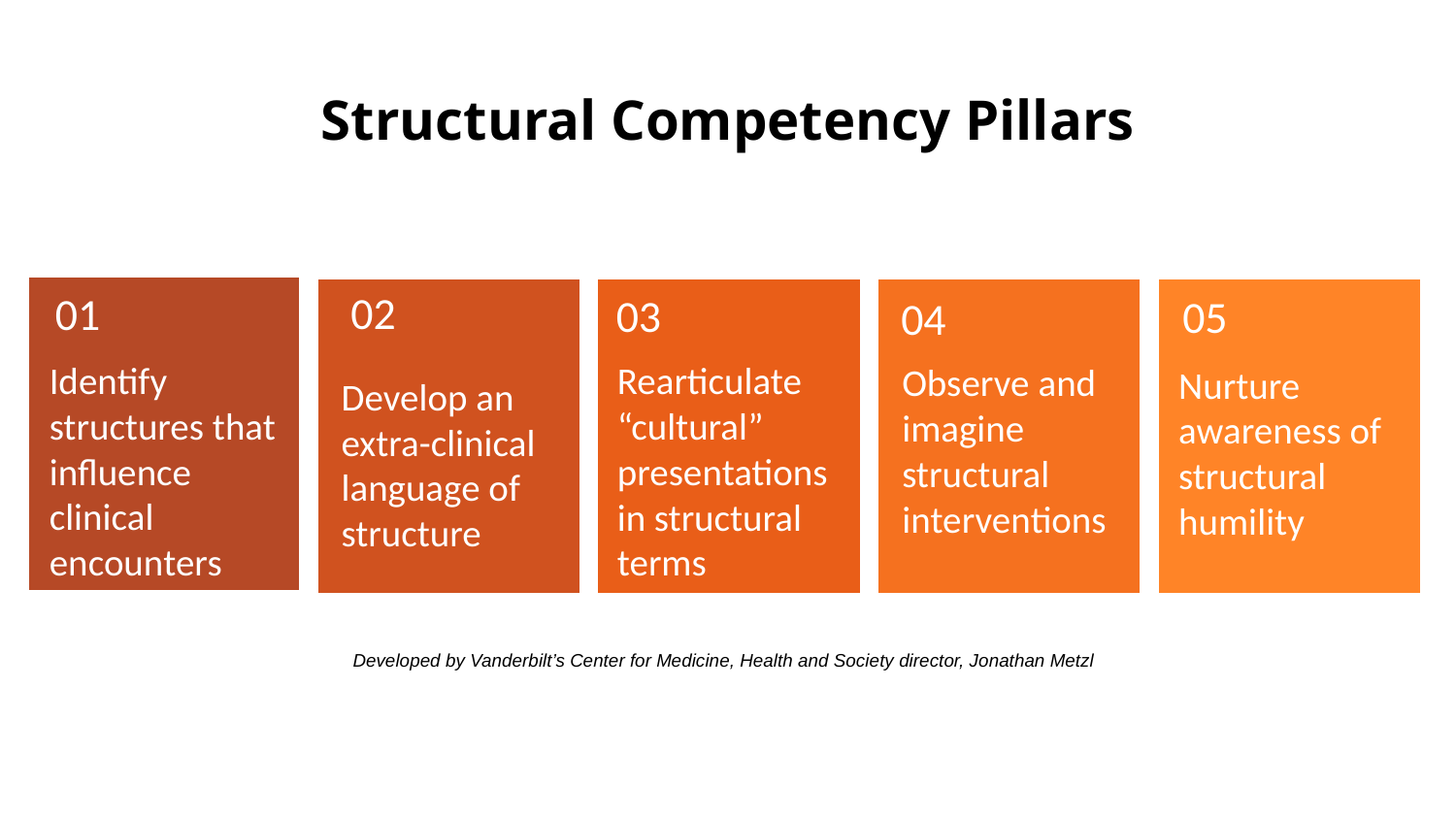

# Structural Competency Pillars
Identify structures that influence clinical encounters
Rearticulate “cultural” presentations in structural terms
Observe and imagine structural interventions
Nurture awareness of structural humility
Develop an extra-clinical language of structure
Developed by Vanderbilt’s Center for Medicine, Health and Society director, Jonathan Metzl

## Slide 10
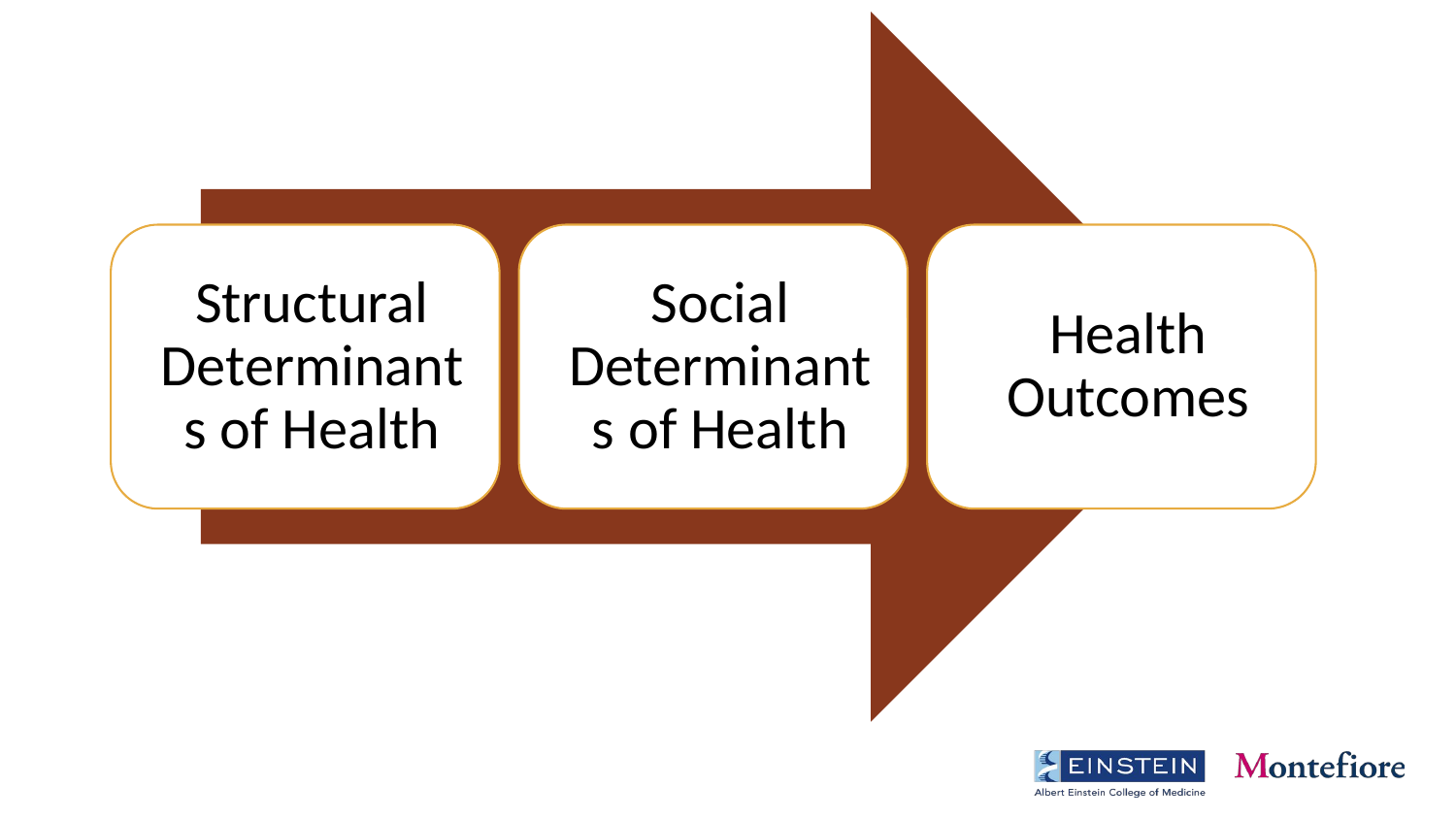

## Slide 11
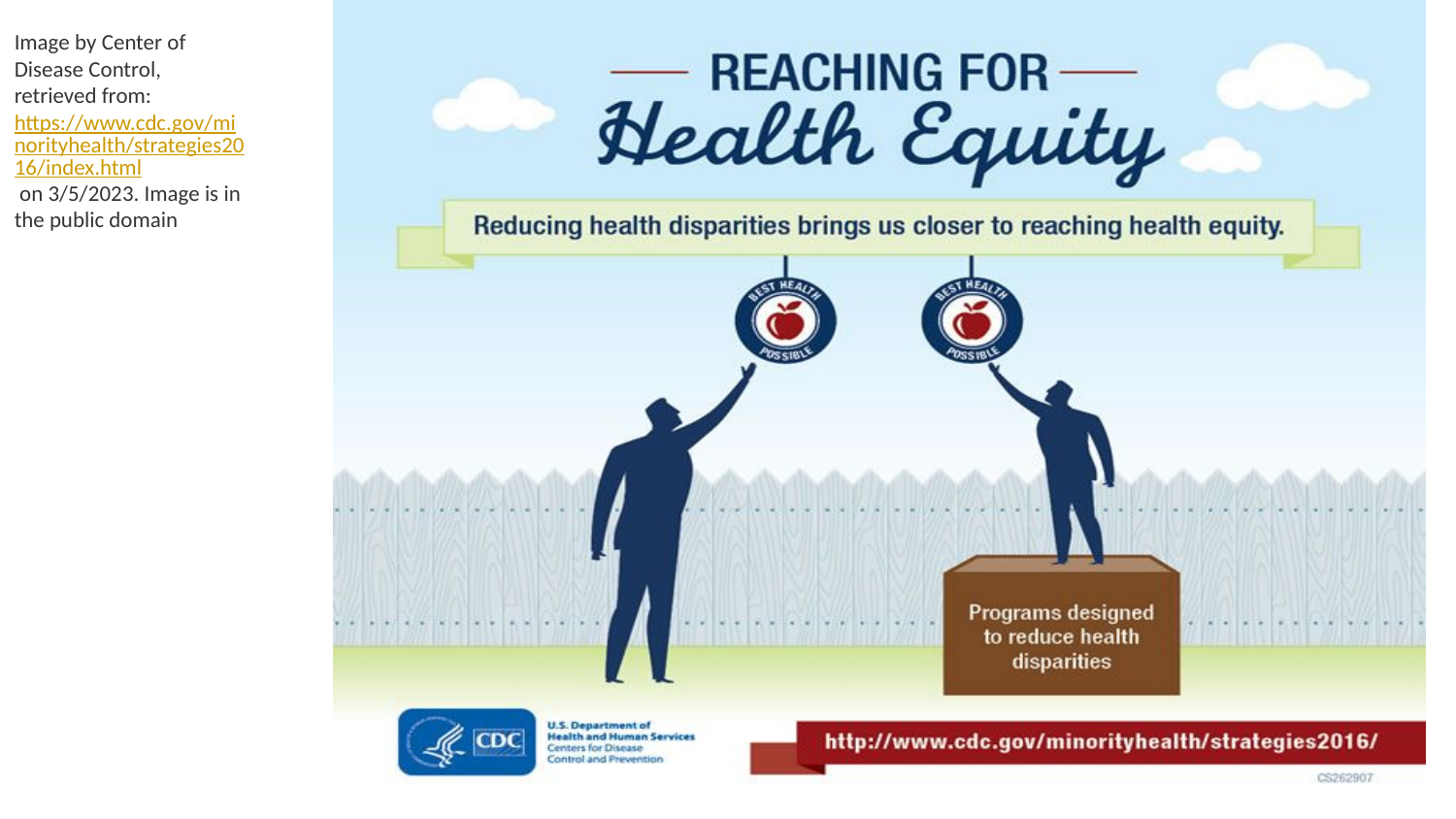

Image by Center of Disease Control, retrieved from: https://www.cdc.gov/minorityhealth/strategies2016/index.html on 3/5/2023. Image is in the public domain
‌

## Slide 12
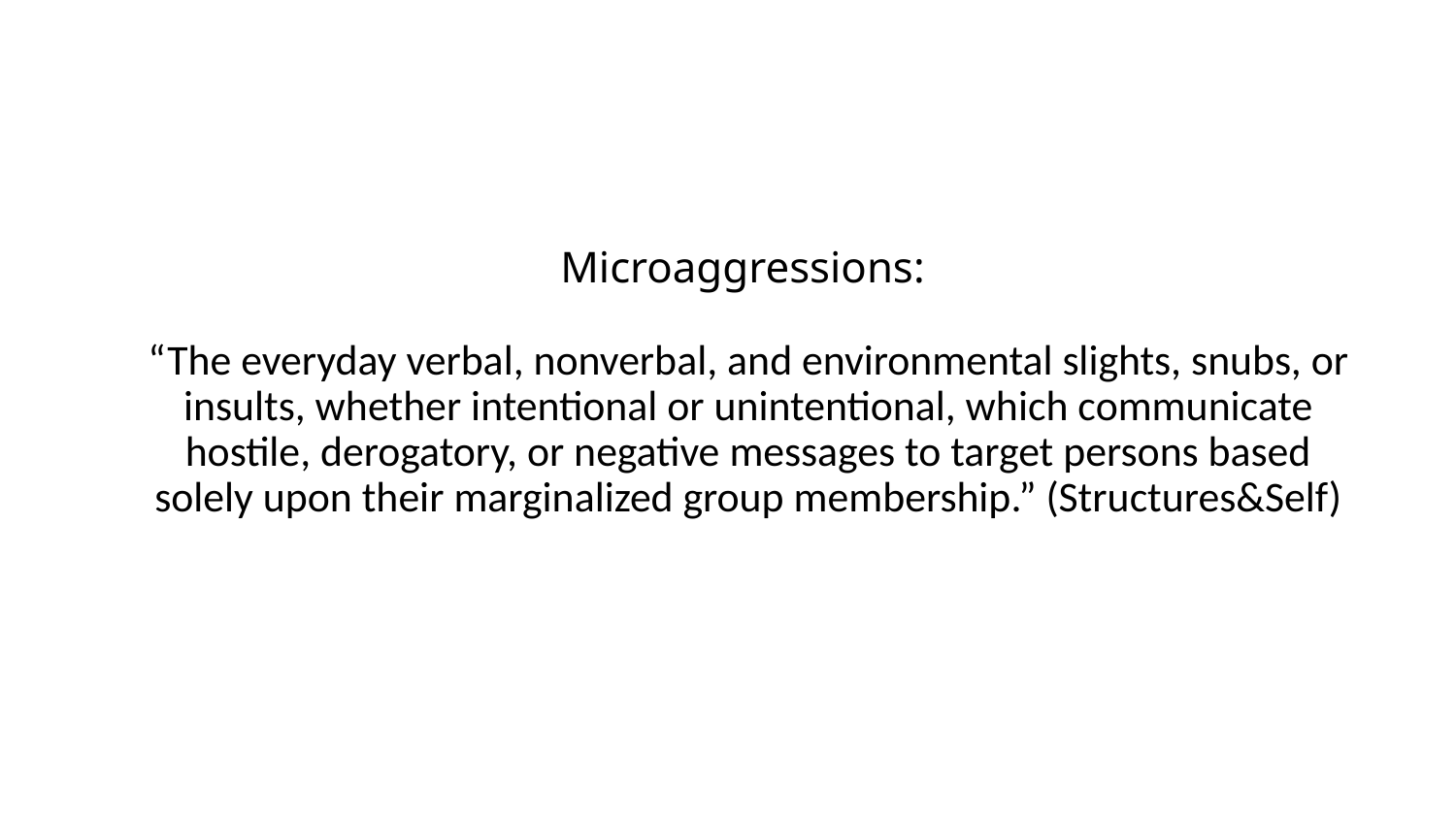

# Microaggressions: “The everyday verbal, nonverbal, and environmental slights, snubs, or insults, whether intentional or unintentional, which communicate hostile, derogatory, or negative messages to target persons based solely upon their marginalized group membership.” (Structures&Self)

## Slide 13
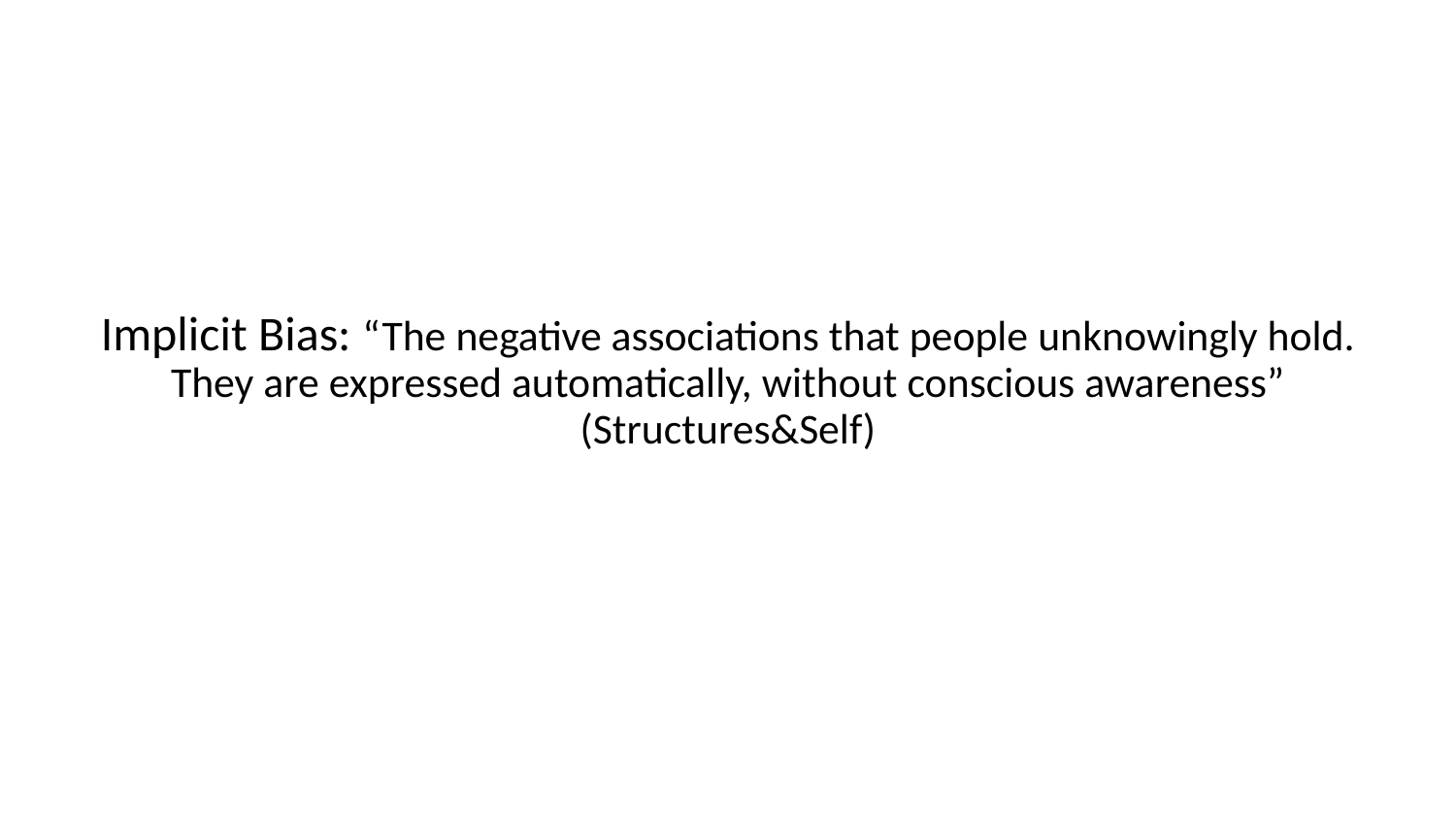

#
Implicit Bias: “The negative associations that people unknowingly hold. They are expressed automatically, without conscious awareness” (Structures&Self)

## Slide 14
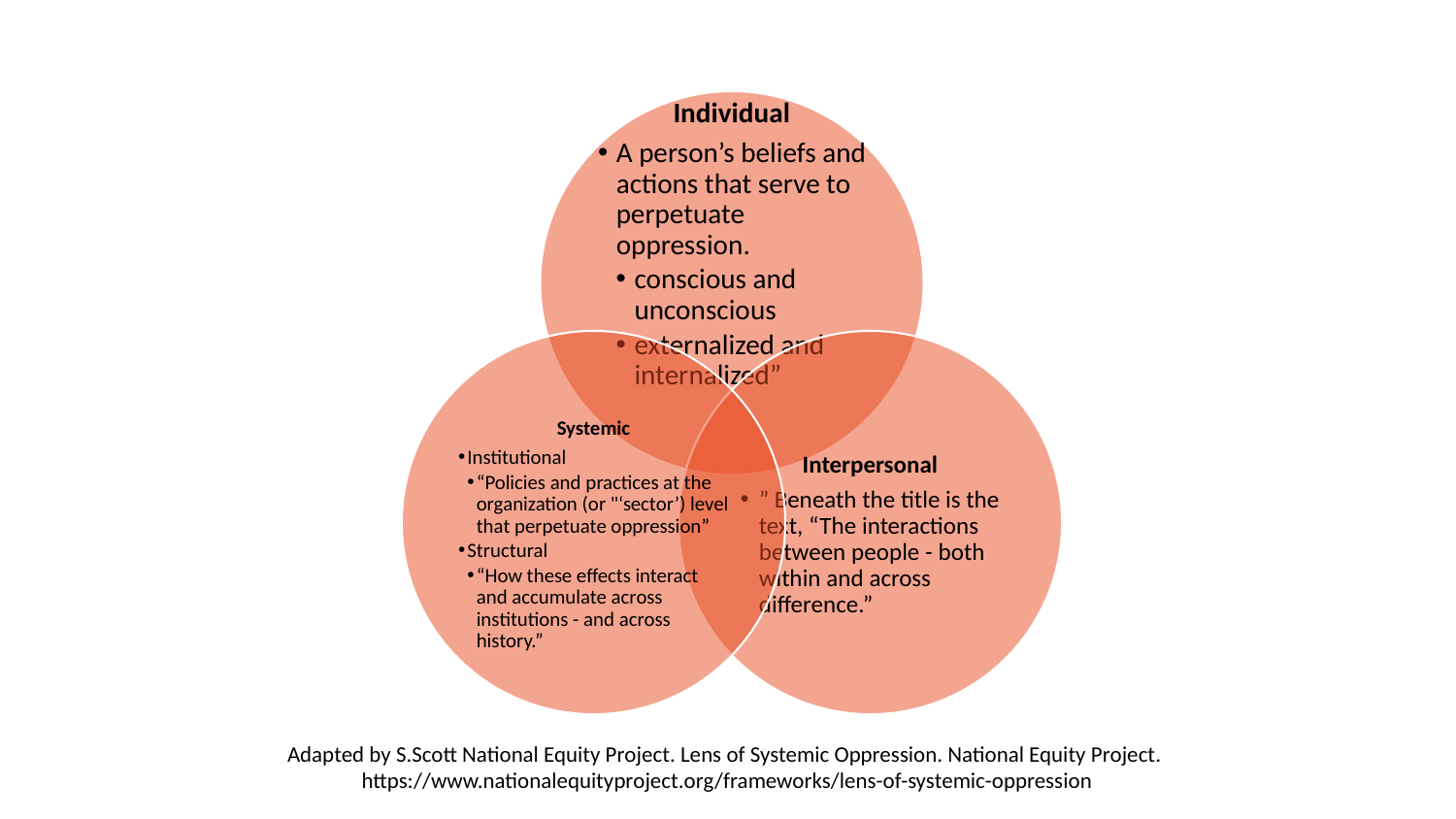

Adapted by S.Scott National Equity Project. Lens of Systemic Oppression. National Equity Project. https://www.nationalequityproject.org/frameworks/lens-of-systemic-oppression
‌

## Slide 15
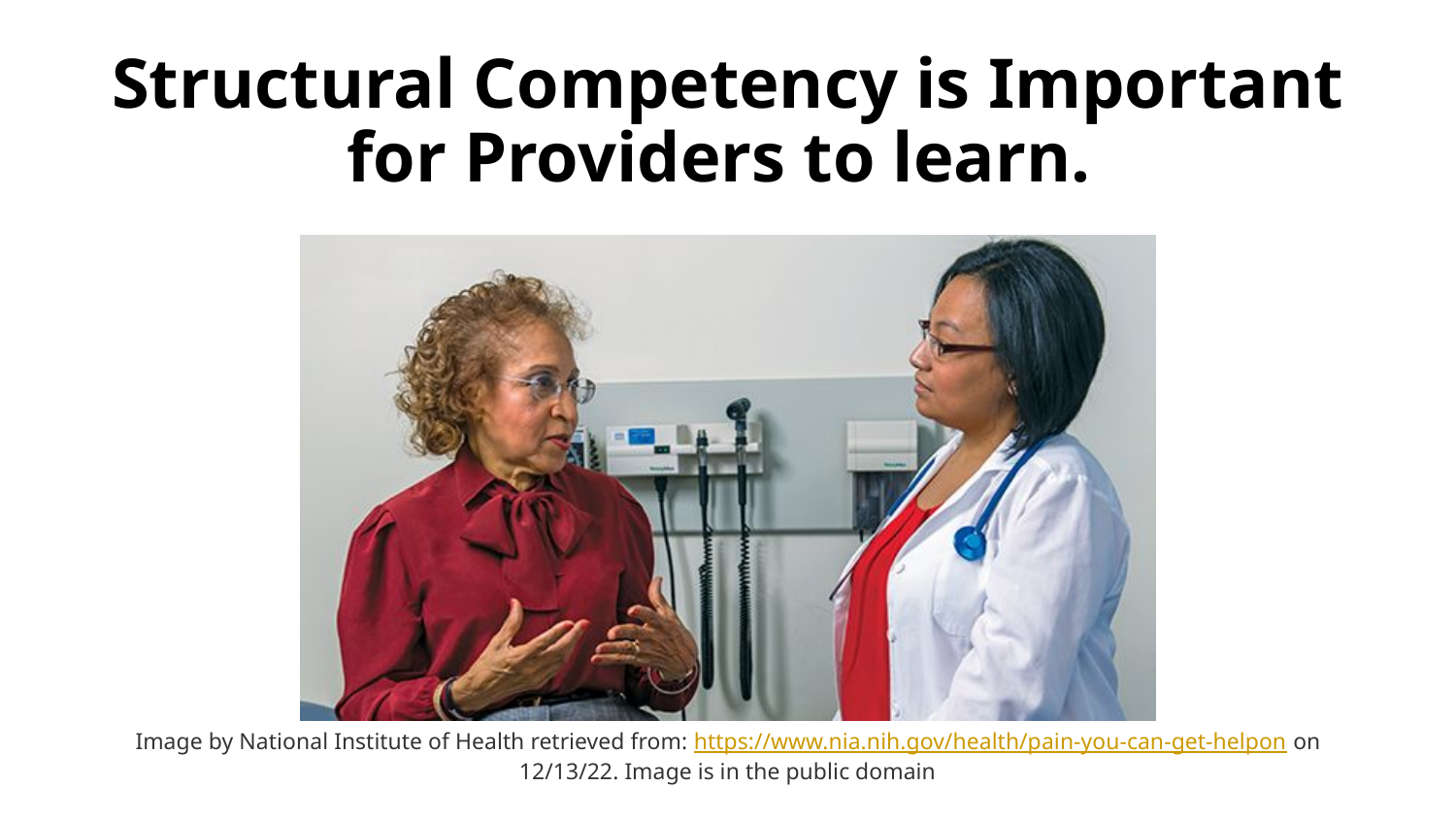

# Structural Competency is Important for Providers to learn.
Image by National Institute of Health retrieved from: https://www.nia.nih.gov/health/pain-you-can-get-helpon on 12/13/22. Image is in the public domain

## Slide 16
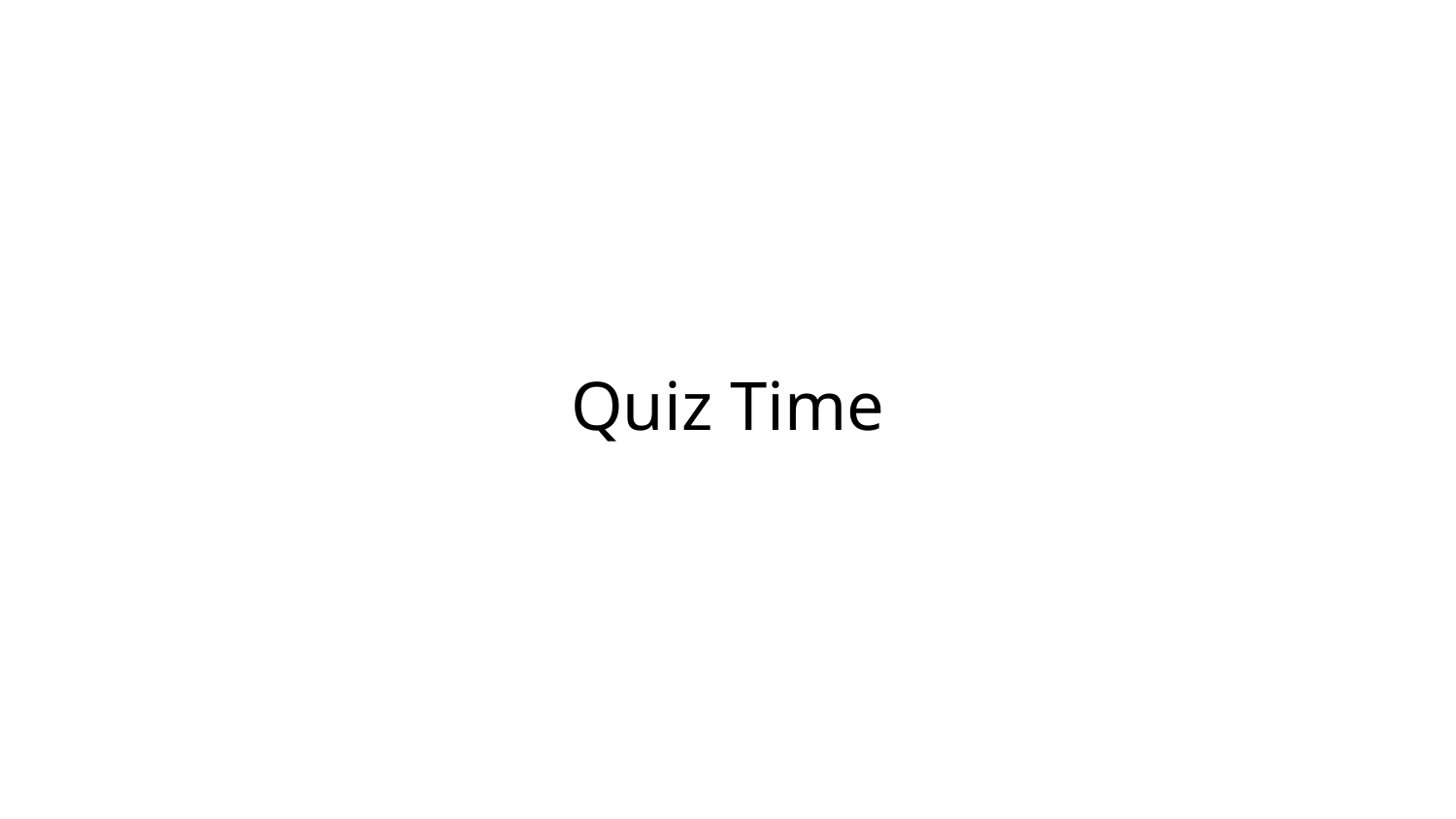

Quiz Time

## Slide 17
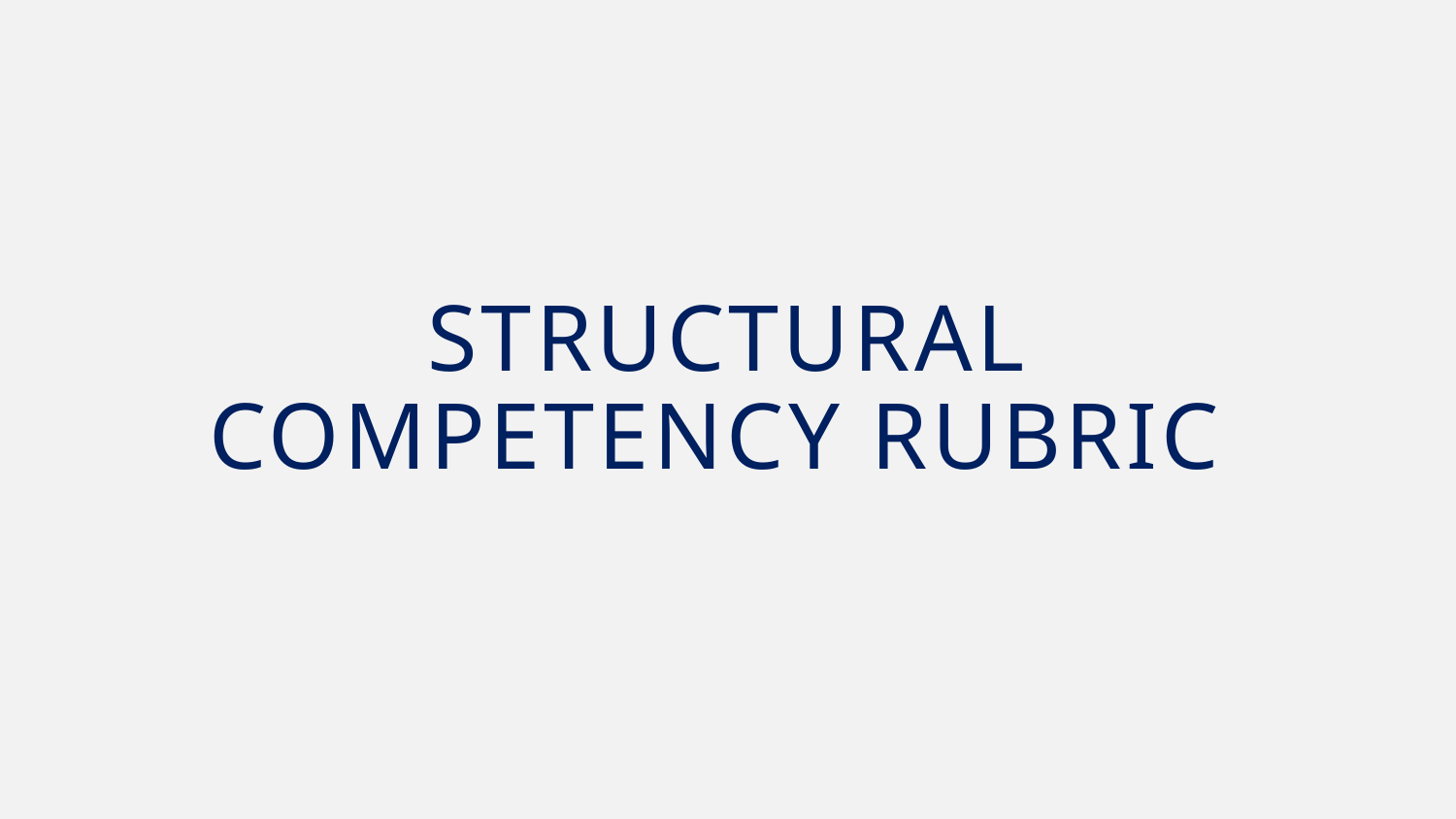

# Structural Competency Rubric

## Slide 18
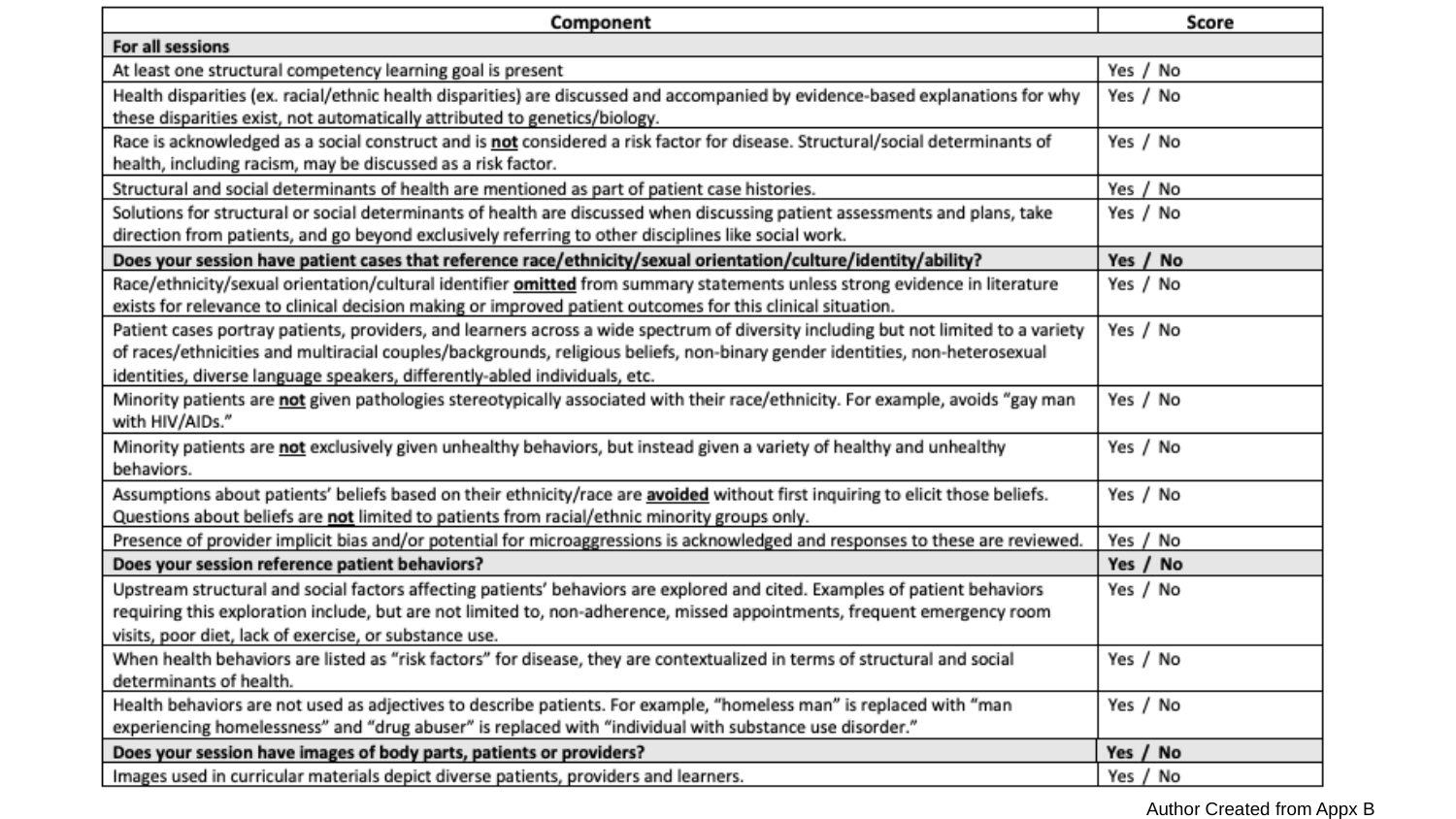

Author Created from Appx B

## Slide 19
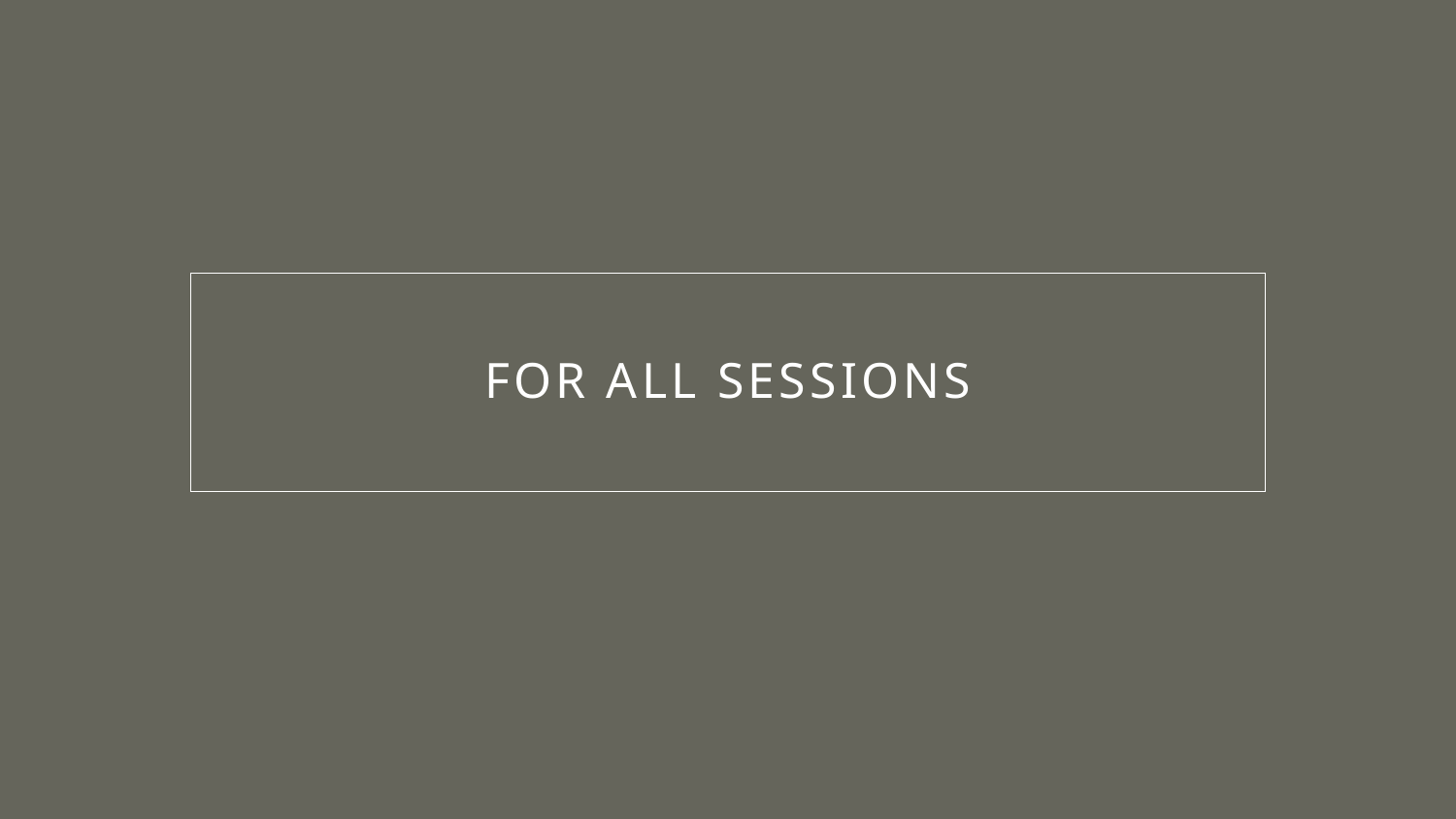

# FOR all sessions

## Slide 20
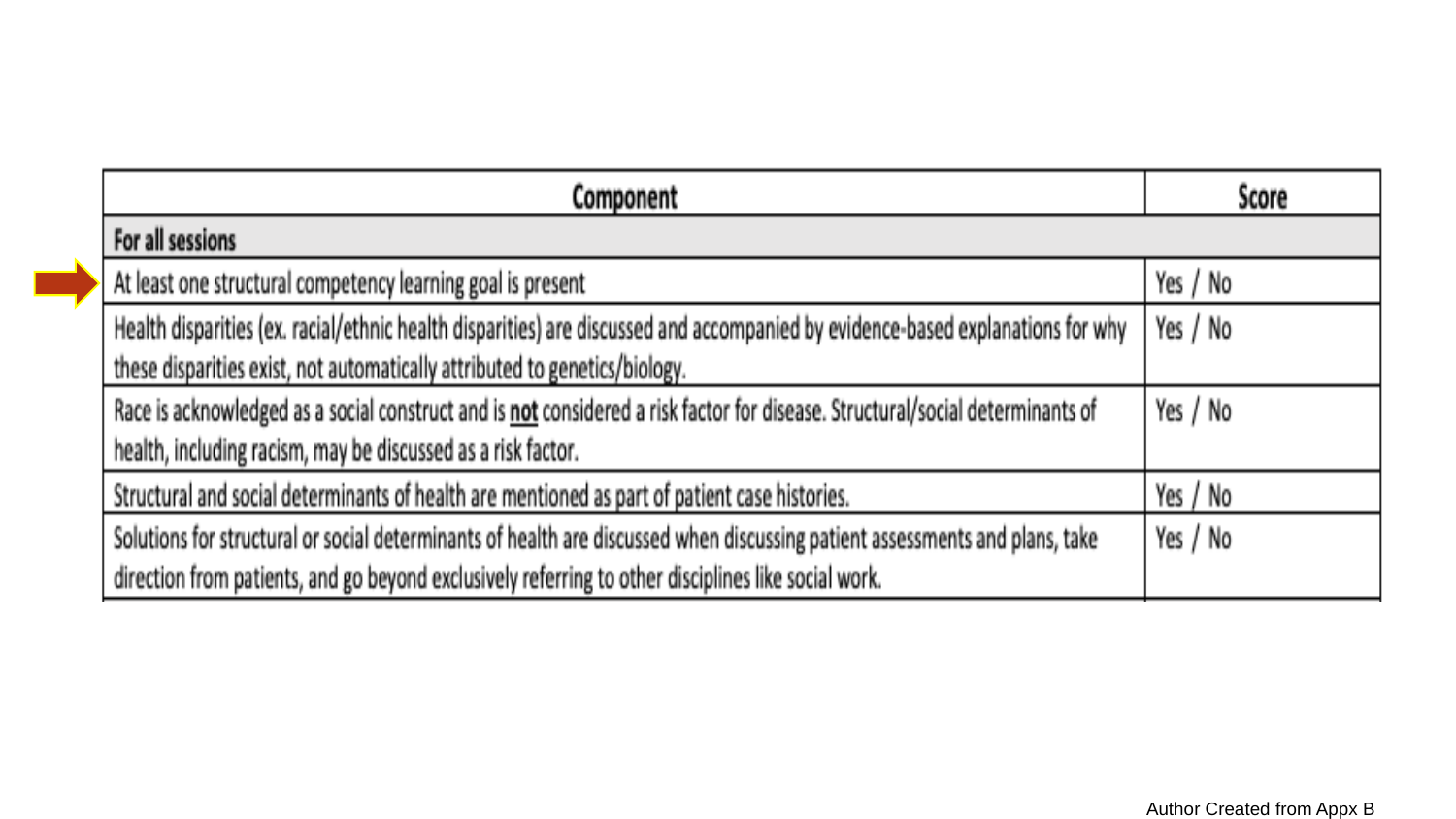

Author Created from Appx B

## Slide 21
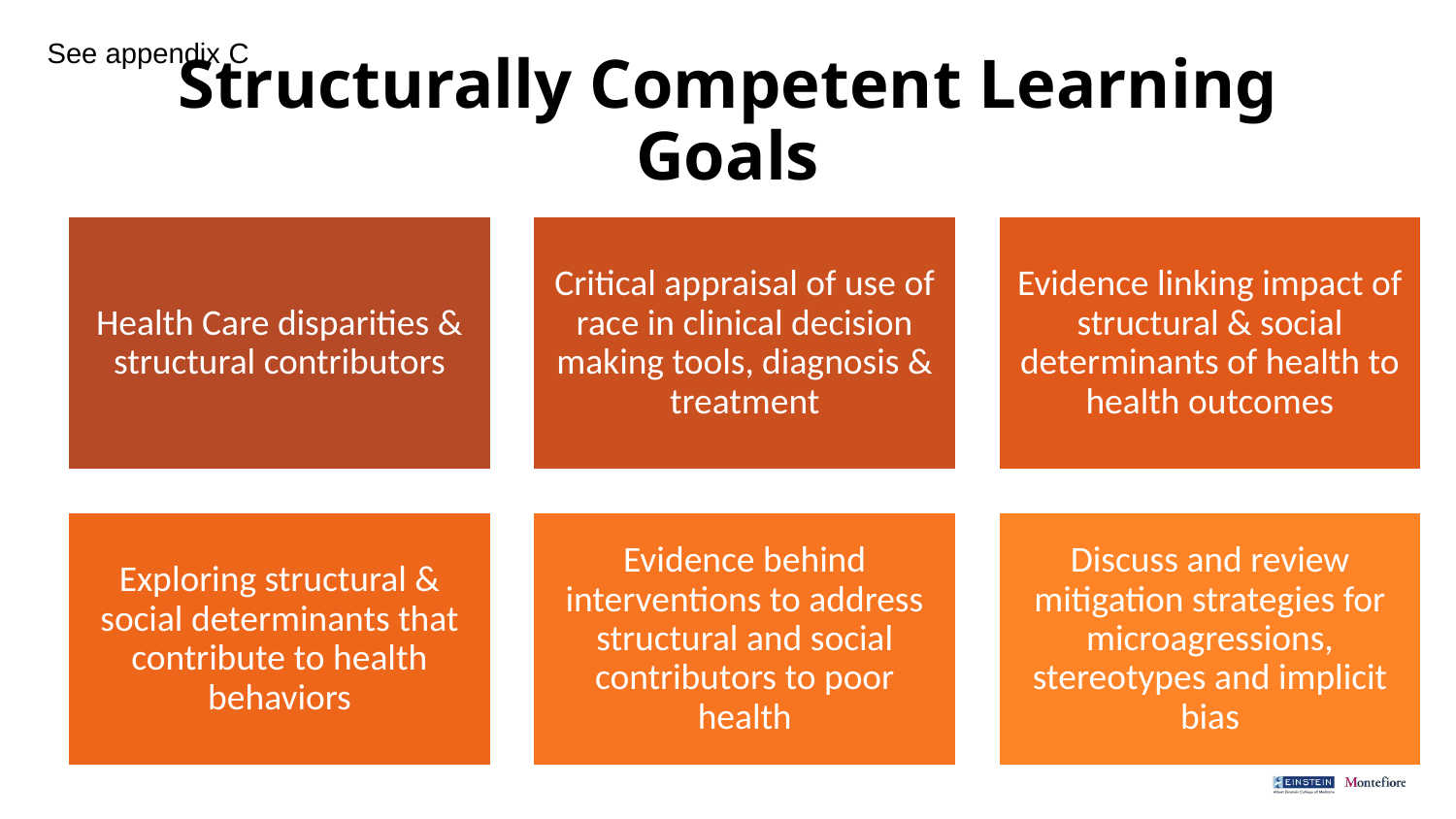

See appendix C
# Structurally Competent Learning Goals

## Slide 22
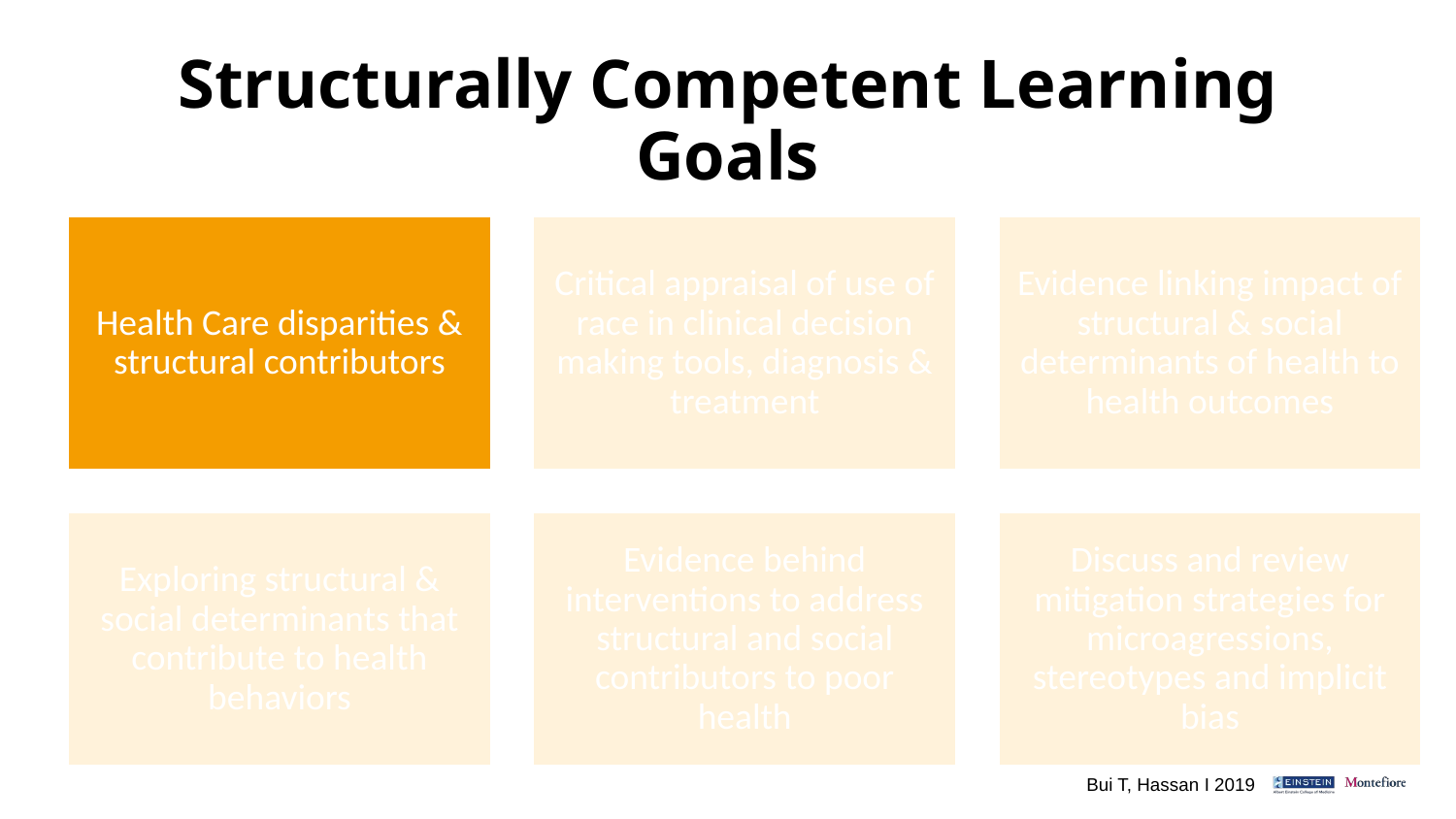

# Structurally Competent Learning Goals
Bui T, Hassan I 2019

## Slide 23
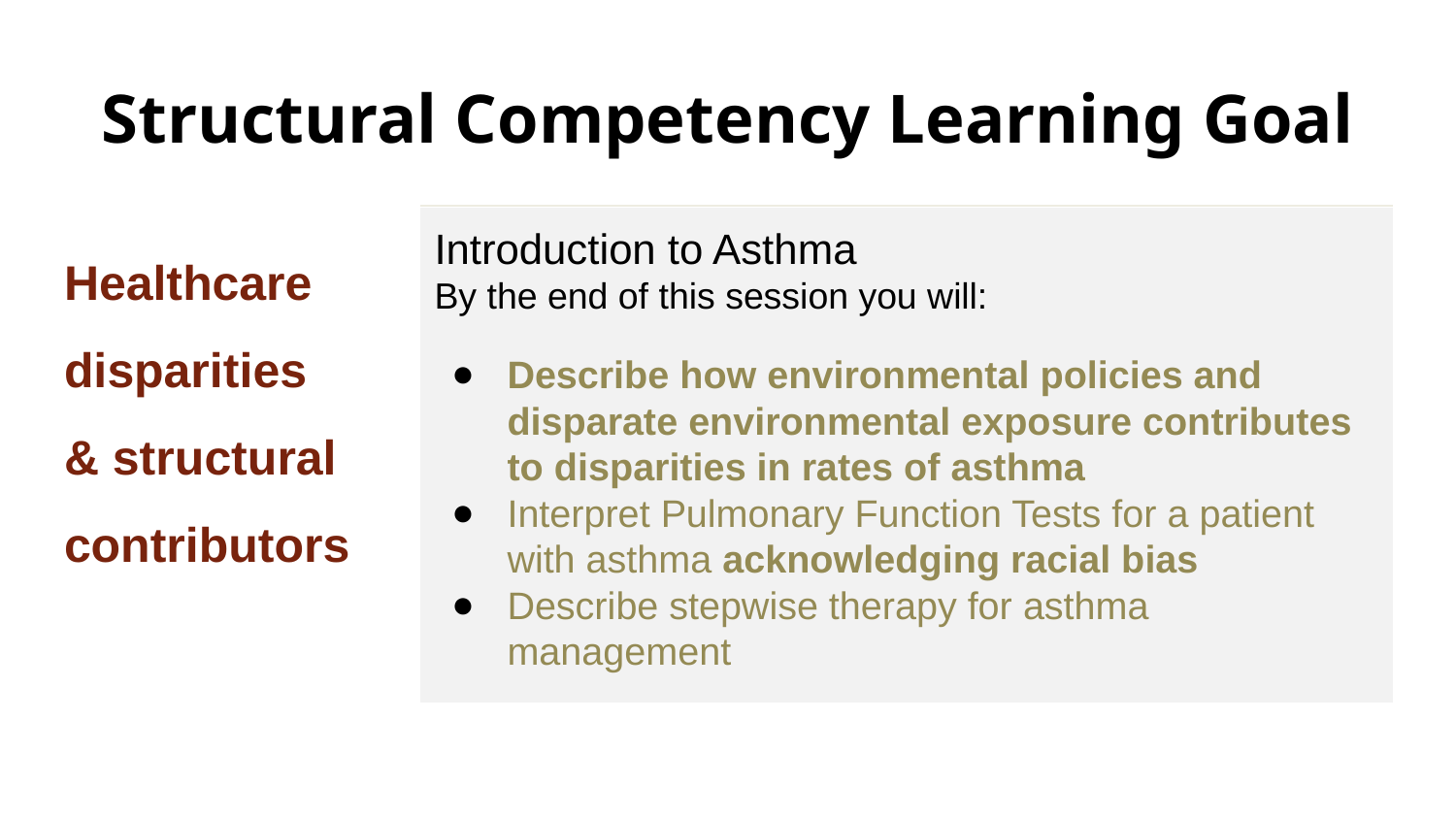

# Structural Competency Learning Goal
Introduction to Asthma
By the end of this session you will:
Describe how environmental policies and disparate environmental exposure contributes to disparities in rates of asthma
Interpret Pulmonary Function Tests for a patient with asthma acknowledging racial bias
Describe stepwise therapy for asthma management
Healthcare disparities
& structural contributors

## Slide 24
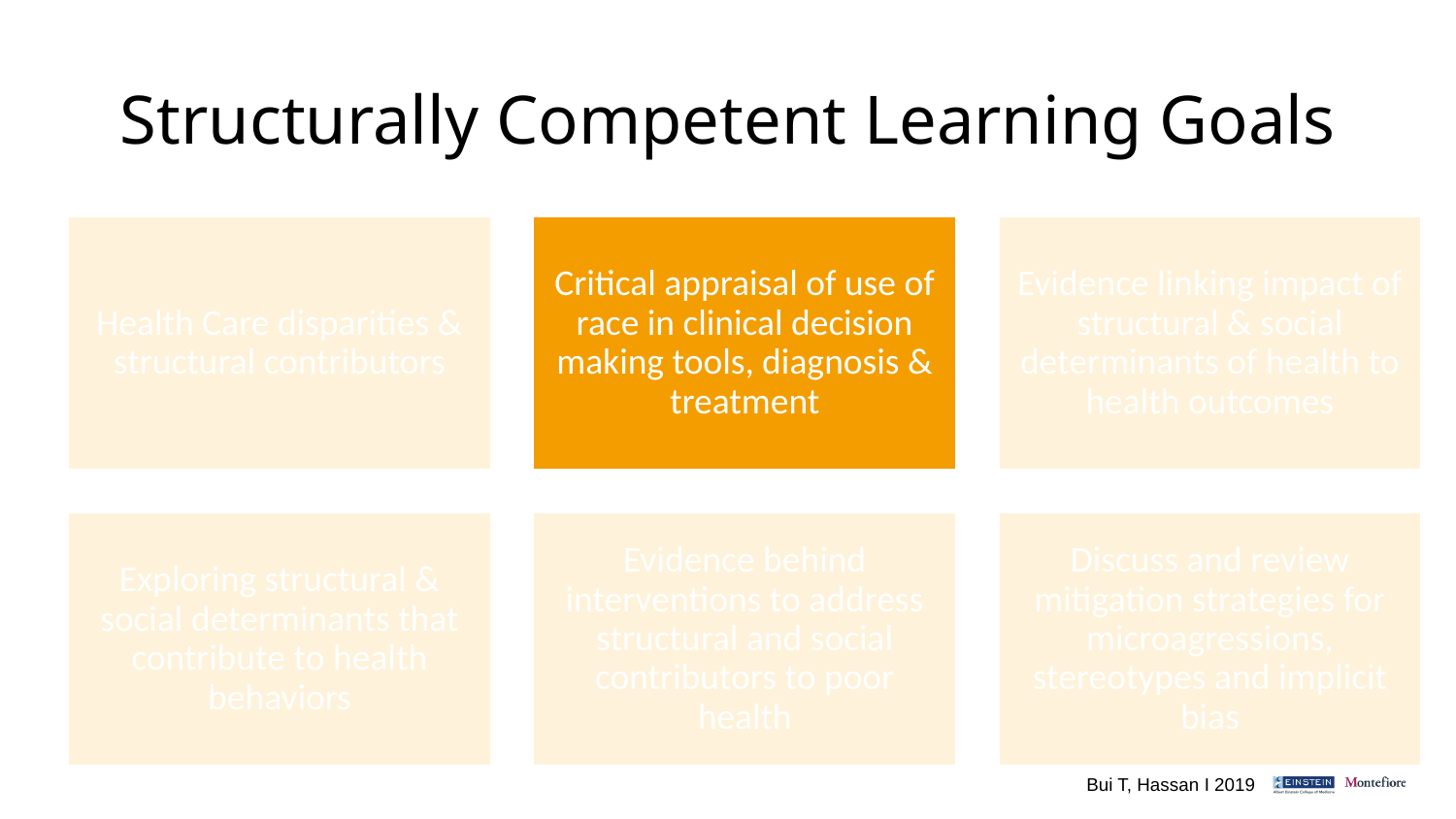

# Structurally Competent Learning Goals
Bui T, Hassan I 2019

## Slide 25
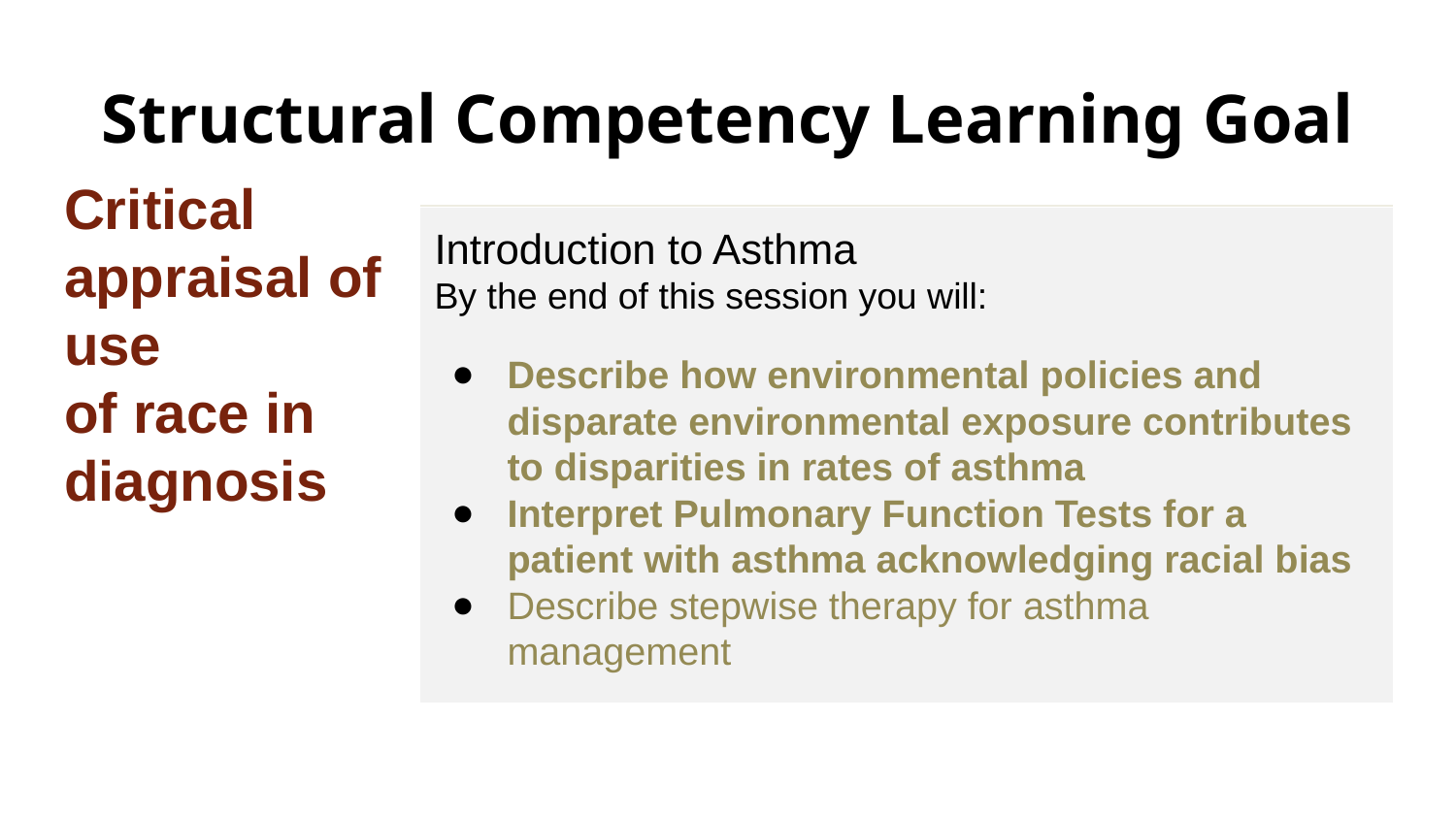

# Structural Competency Learning Goal
Critical appraisal of use
of race in diagnosis
Introduction to Asthma
By the end of this session you will:
Describe how environmental policies and disparate environmental exposure contributes to disparities in rates of asthma
Interpret Pulmonary Function Tests for a patient with asthma acknowledging racial bias
Describe stepwise therapy for asthma management

## Slide 26
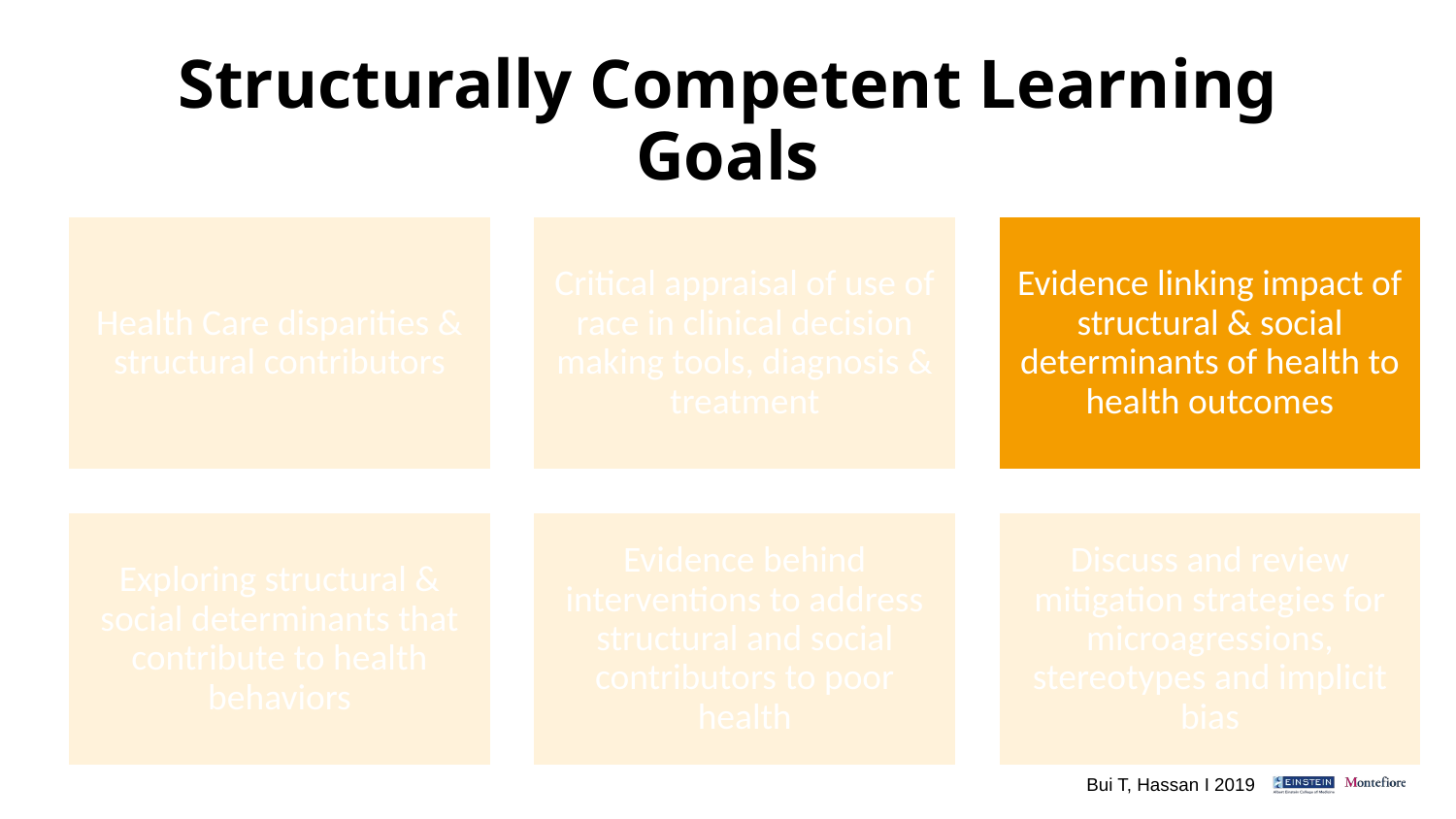

# Structurally Competent Learning Goals
Bui T, Hassan I 2019

## Slide 27
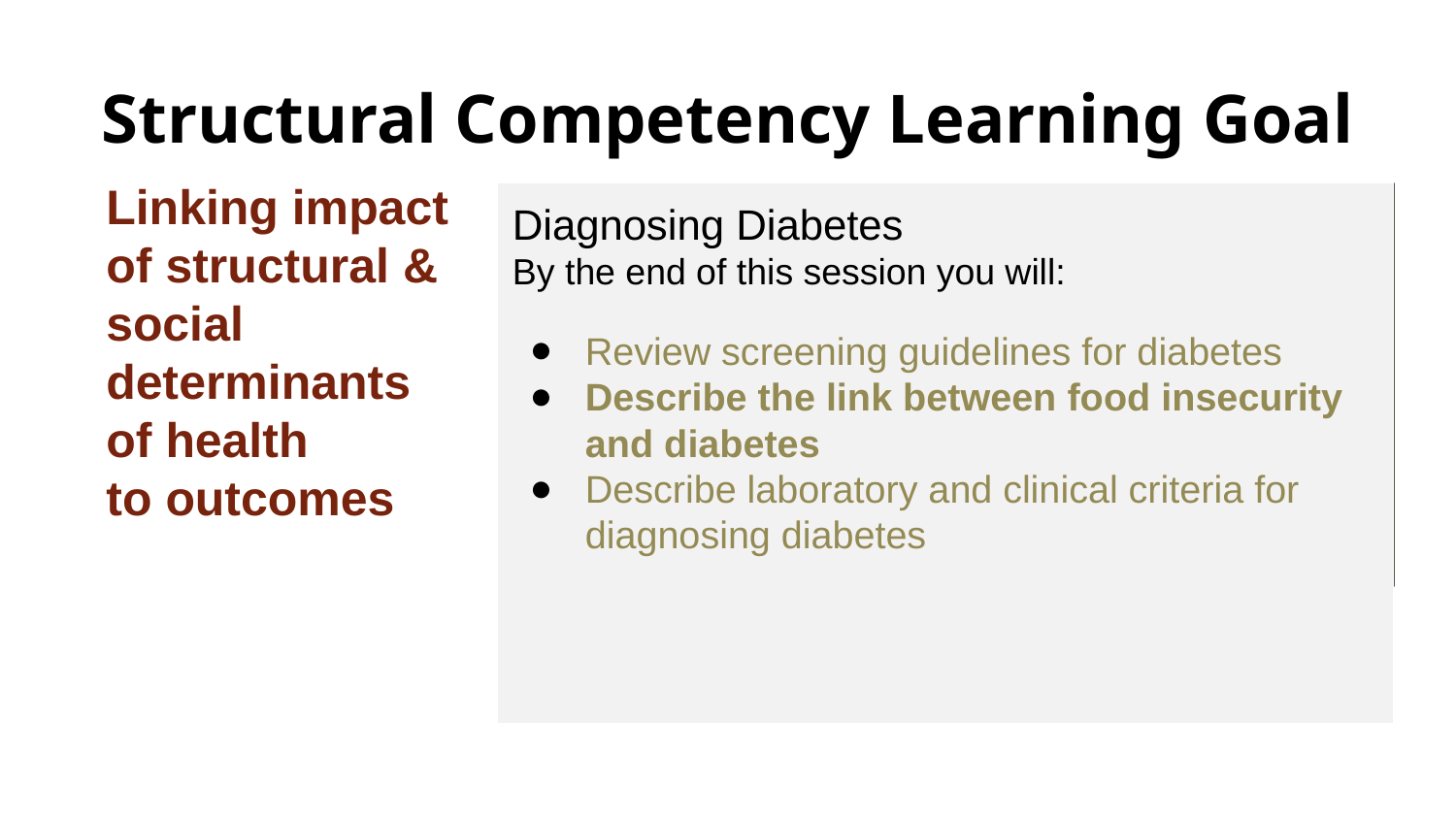

# Structural Competency Learning Goal
Linking impact
of structural & social
determinants of health
to outcomes
Diagnosing Diabetes
By the end of this session you will:
Review screening guidelines for diabetes
Describe the link between food insecurity and diabetes
Describe laboratory and clinical criteria for diagnosing diabetes

## Slide 28
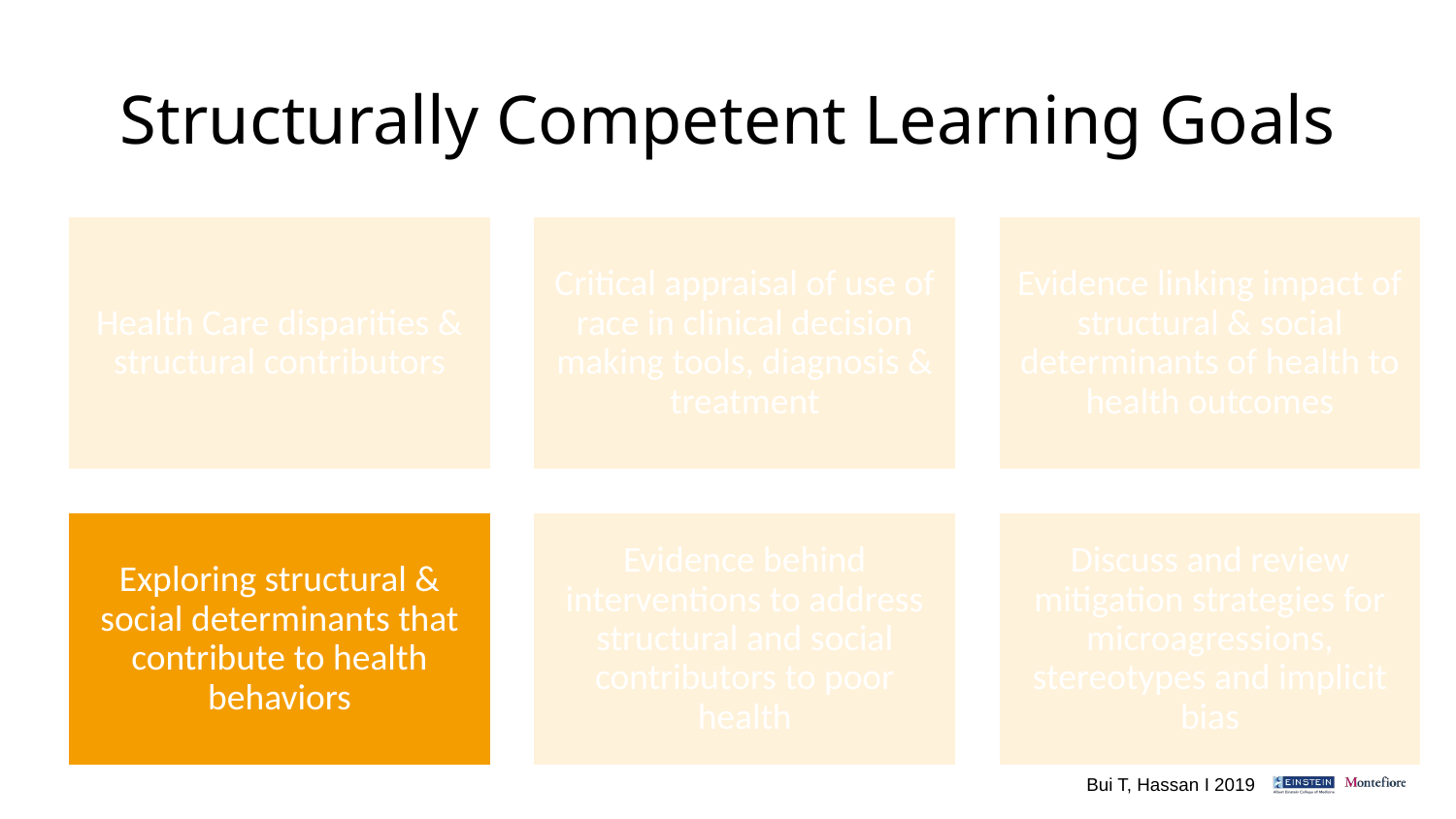

# Structurally Competent Learning Goals
Bui T, Hassan I 2019

## Slide 29
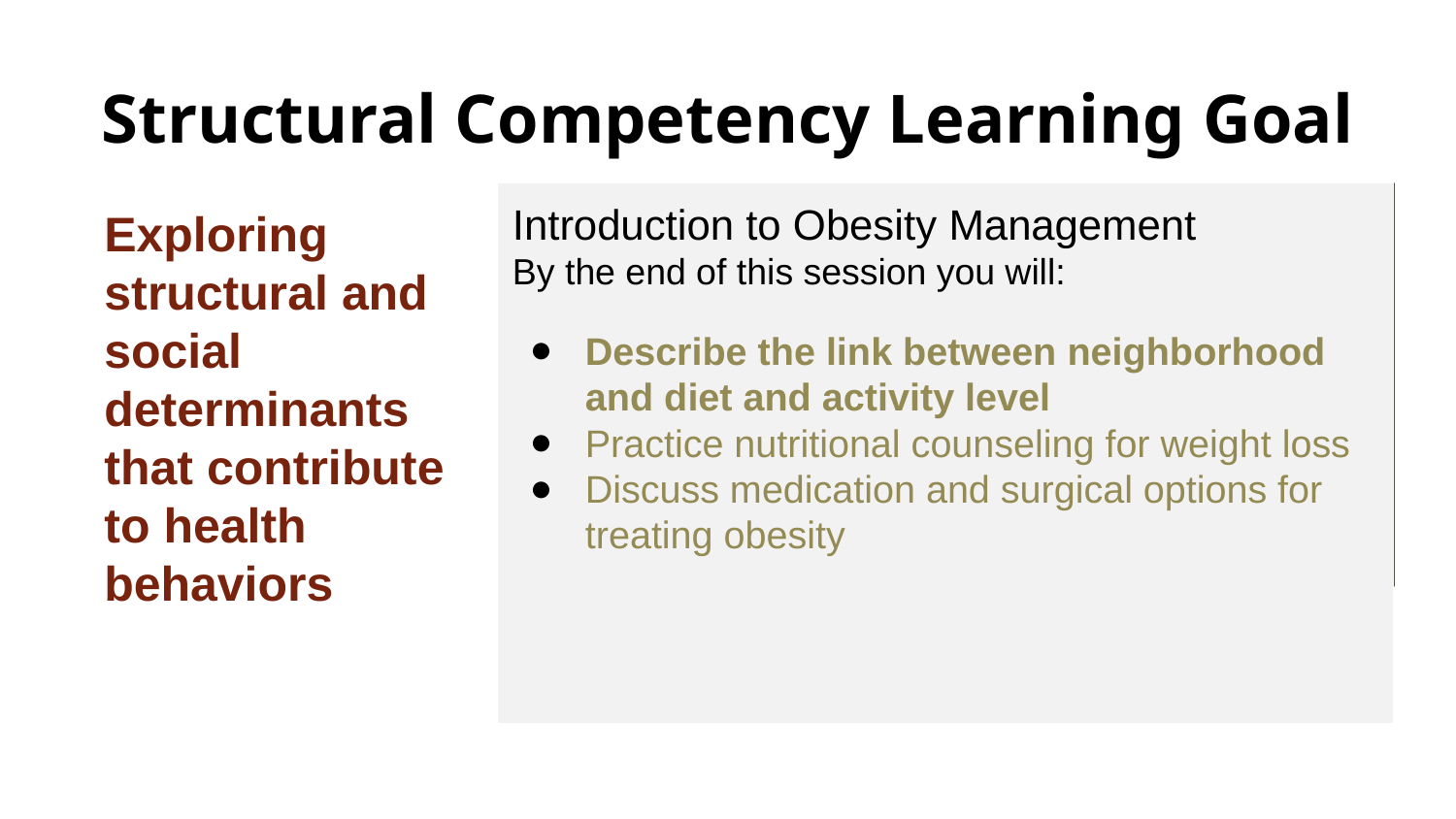

# Structural Competency Learning Goal
Introduction to Obesity Management
By the end of this session you will:
Describe the link between neighborhood and diet and activity level
Practice nutritional counseling for weight loss
Discuss medication and surgical options for treating obesity
Exploring structural and social determinants that contribute to health behaviors

## Slide 30
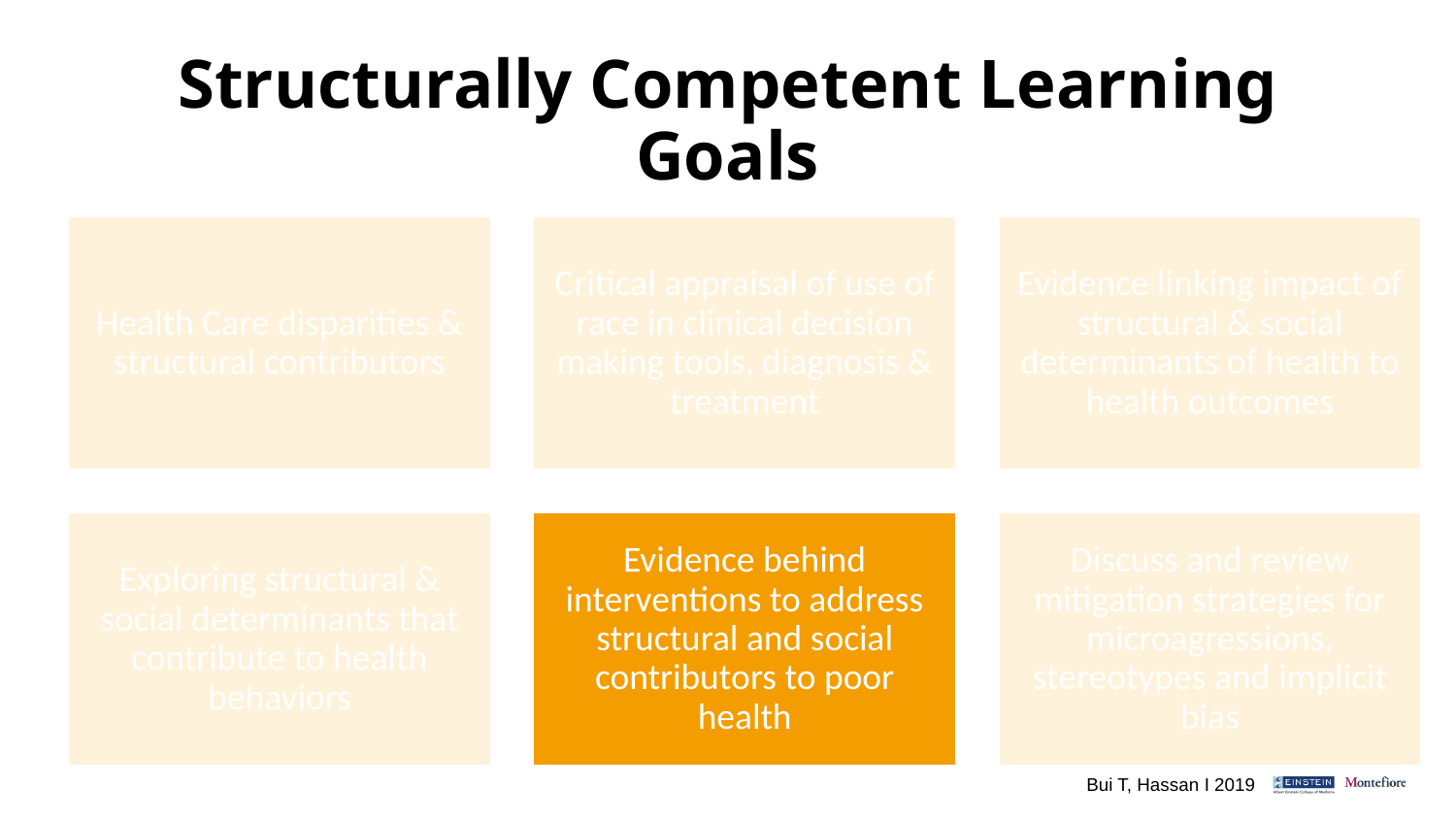

# Structurally Competent Learning Goals
Bui T, Hassan I 2019

## Slide 31
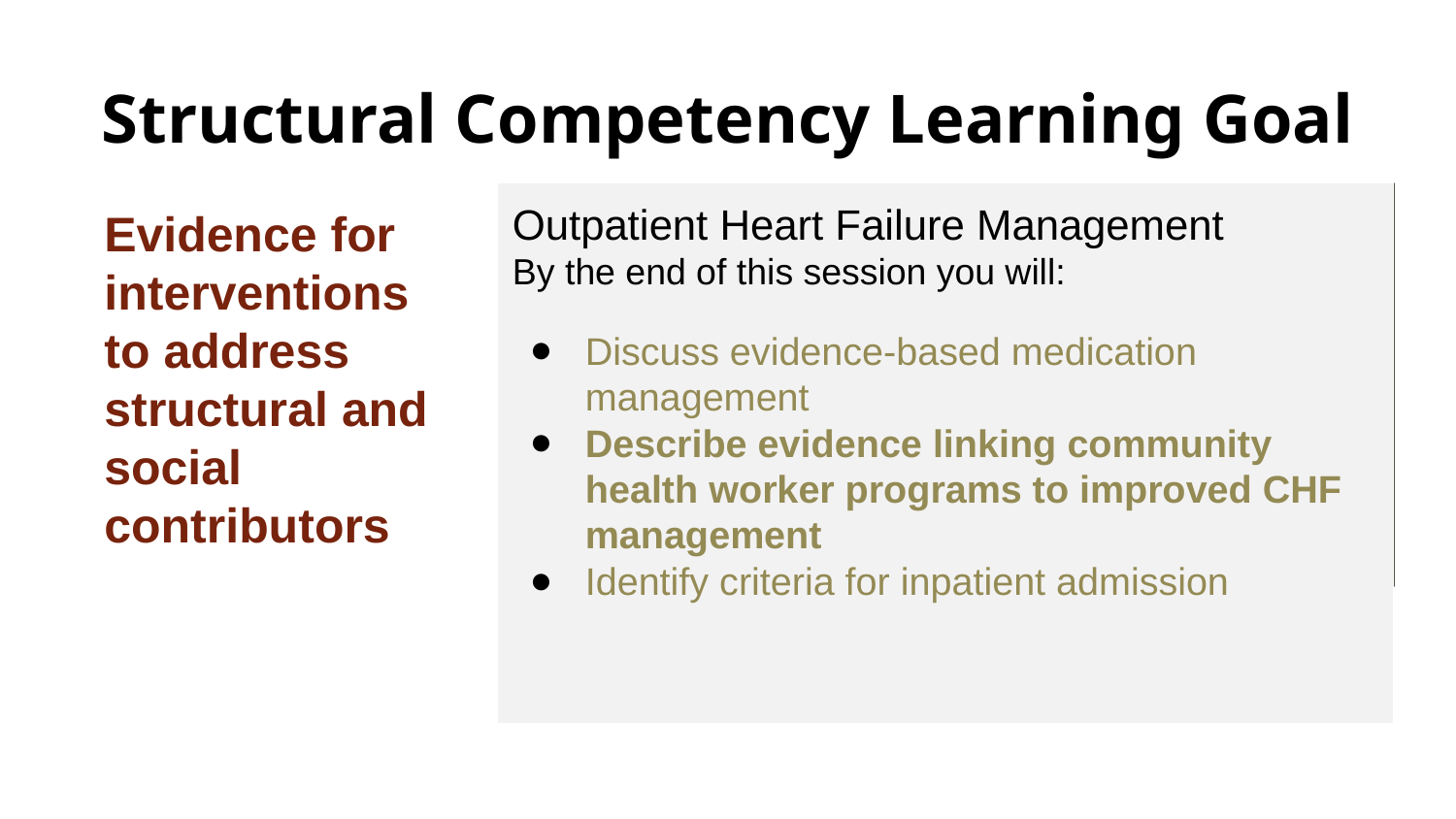

# Structural Competency Learning Goal
Outpatient Heart Failure Management
By the end of this session you will:
Discuss evidence-based medication management
Describe evidence linking community health worker programs to improved CHF management
Identify criteria for inpatient admission
Evidence for interventions to address structural and social contributors

## Slide 32
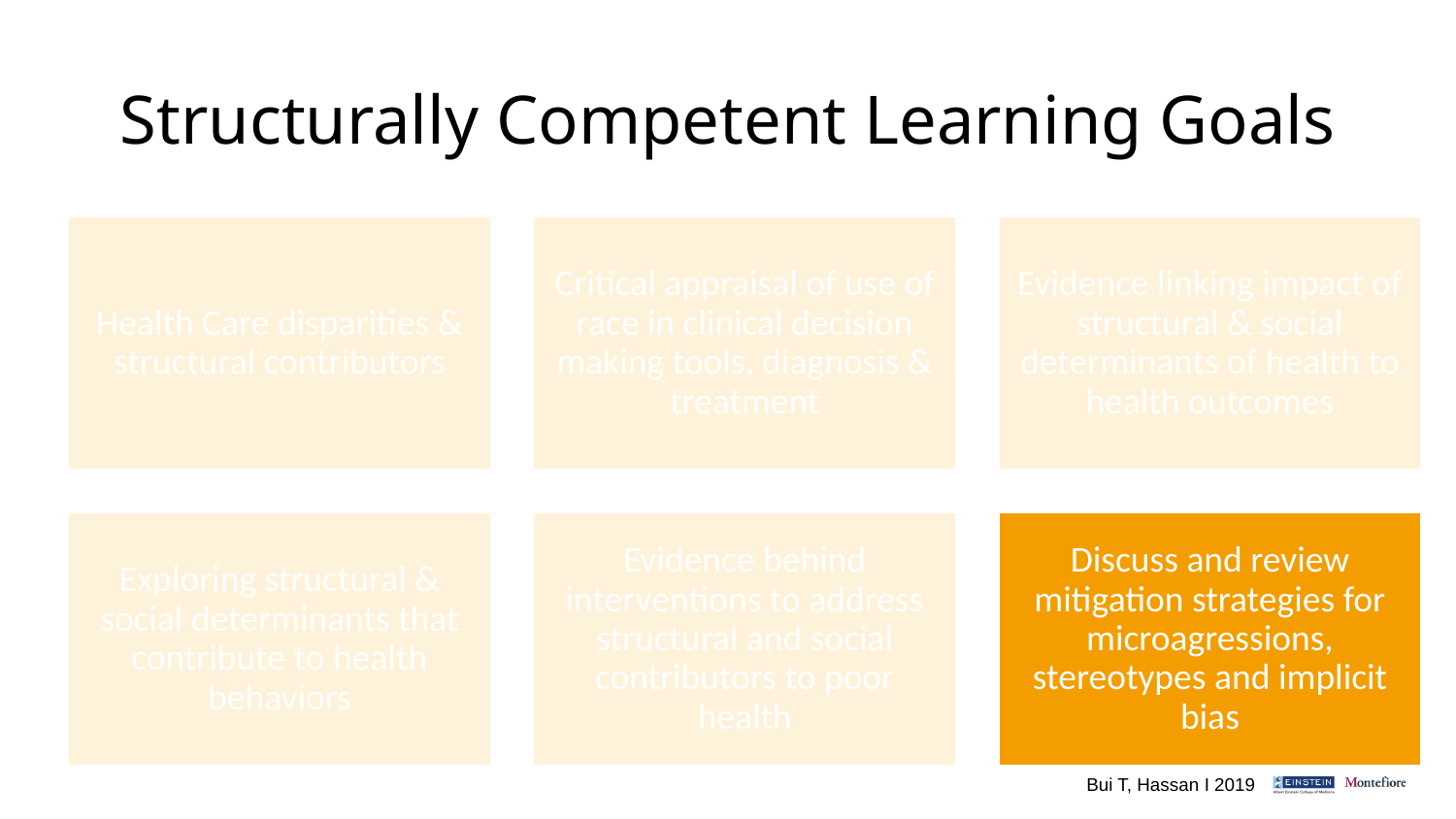

# Structurally Competent Learning Goals
Bui T, Hassan I 2019

## Slide 33
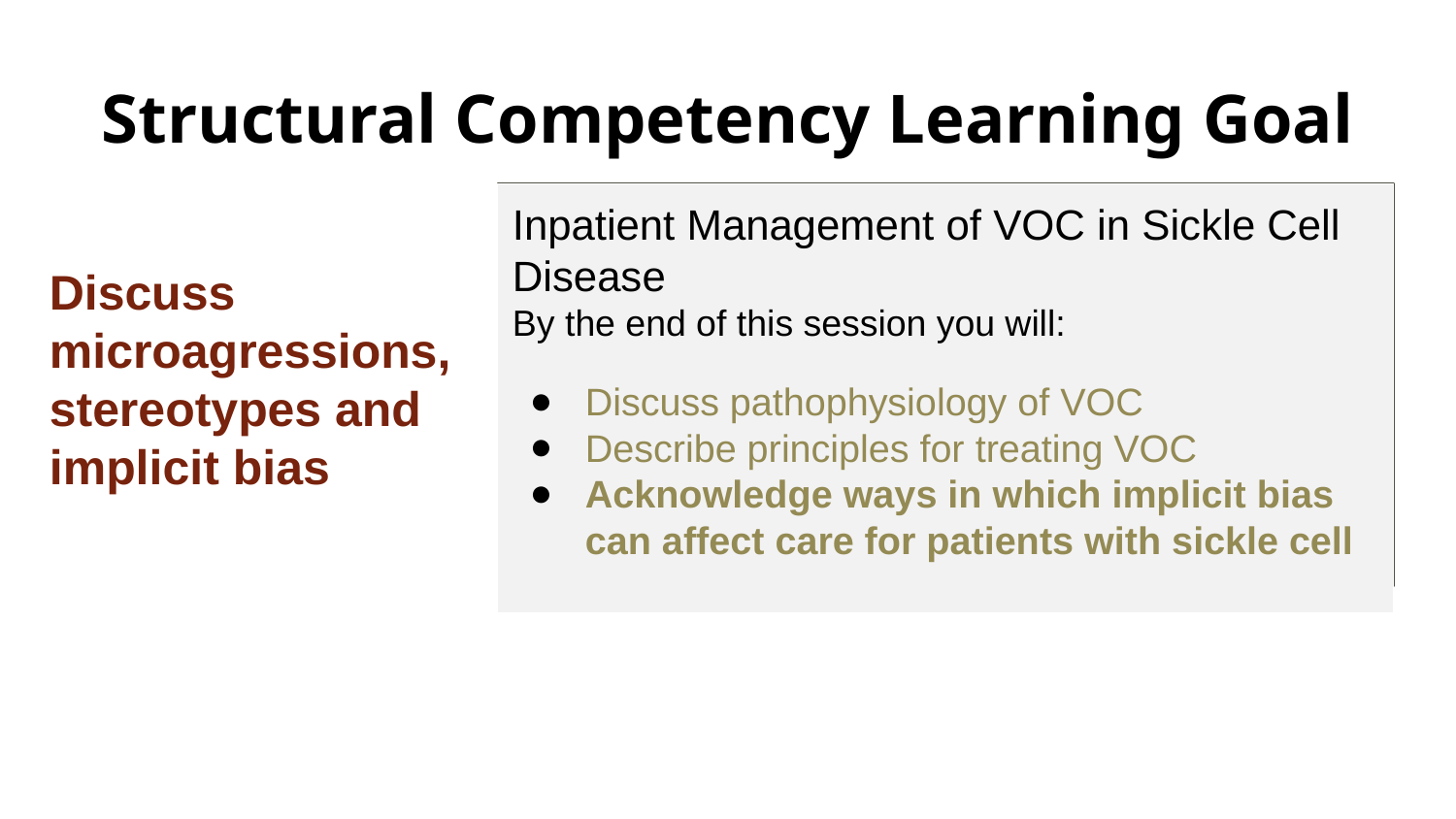

# Structural Competency Learning Goal
Inpatient Management of VOC in Sickle Cell Disease
By the end of this session you will:
Discuss pathophysiology of VOC
Describe principles for treating VOC
Acknowledge ways in which implicit bias can affect care for patients with sickle cell
Discuss microagressions, stereotypes and implicit bias

## Slide 34
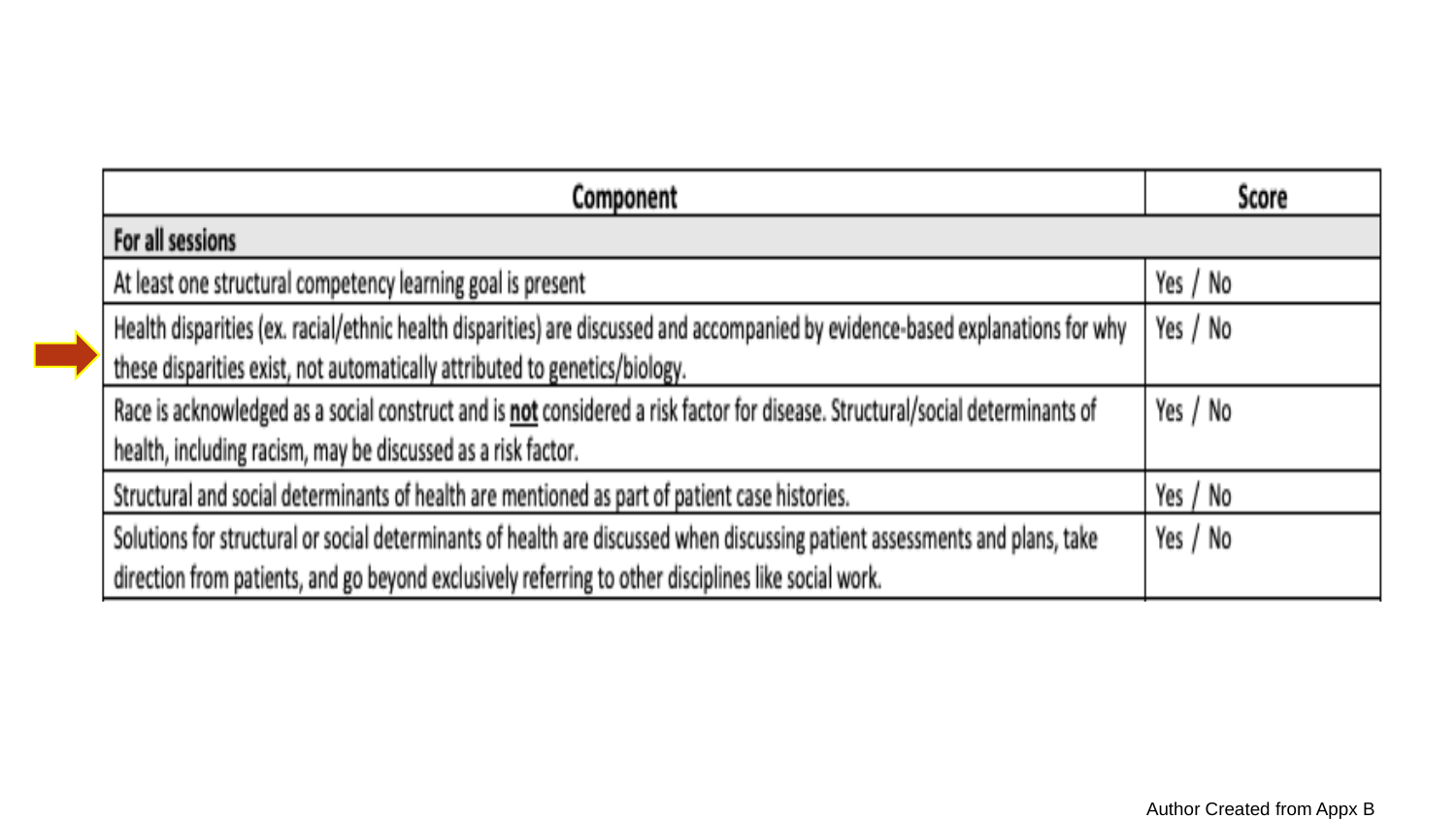

Author Created from Appx B

## Slide 35
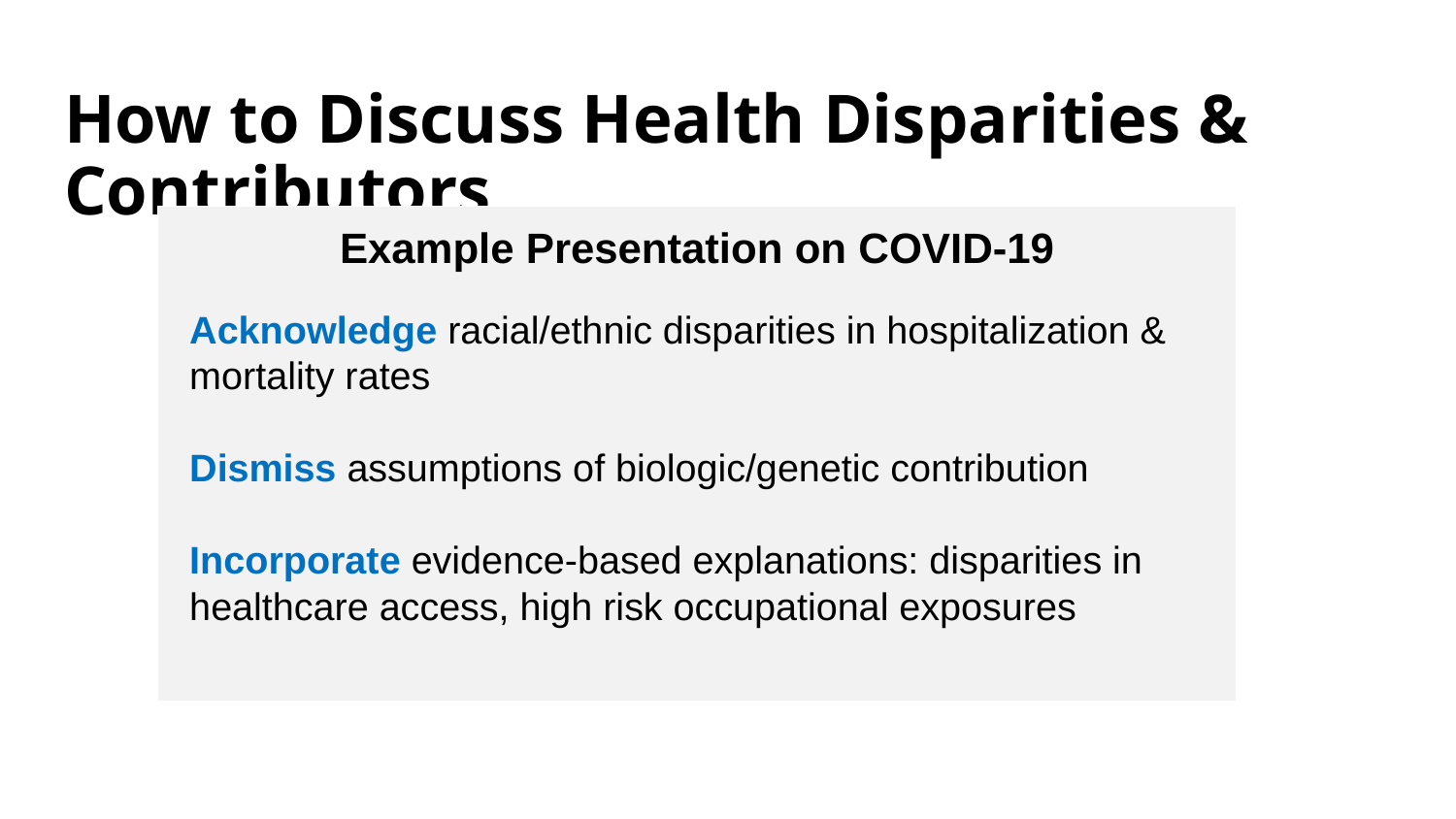

# How to Discuss Health Disparities & Contributors
Example Presentation on COVID-19
Acknowledge racial/ethnic disparities in hospitalization & mortality rates
Dismiss assumptions of biologic/genetic contribution
Incorporate evidence-based explanations: disparities in healthcare access, high risk occupational exposures

## Slide 36
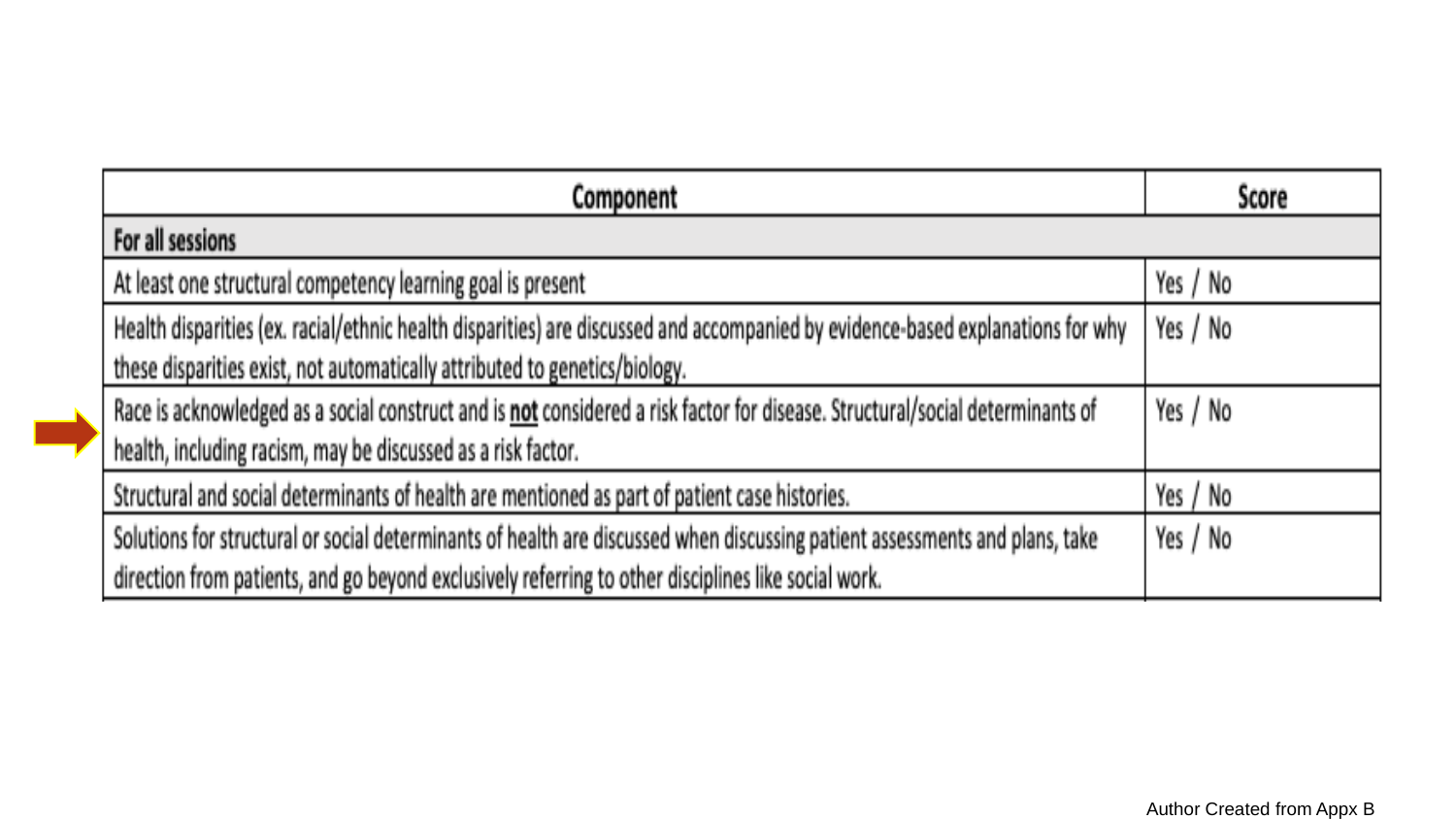

Author Created from Appx B

## Slide 37
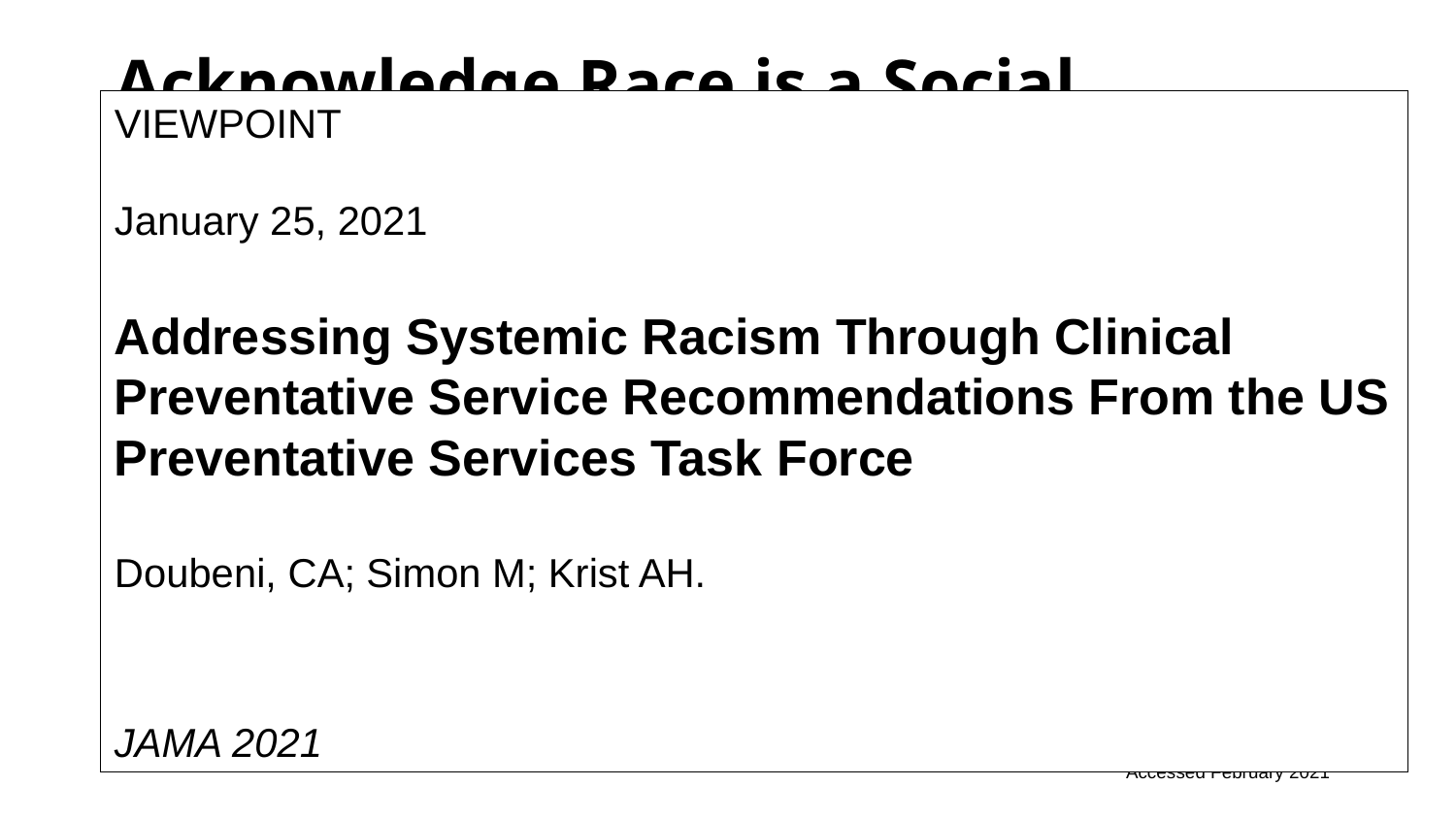

# Acknowledge Race is a Social Construct
VIEWPOINT
January 25, 2021
Addressing Systemic Racism Through Clinical Preventative Service Recommendations From the US Preventative Services Task Force
Doubeni, CA; Simon M; Krist AH.
JAMA 2021
“Factors associated with increased prevalence that clinicians should consider include history of incarceration, history of commercial sex work, certain racial/ethnic groups…”
USPSTF Recommendation 2016
Accessed February 2021

## Slide 38
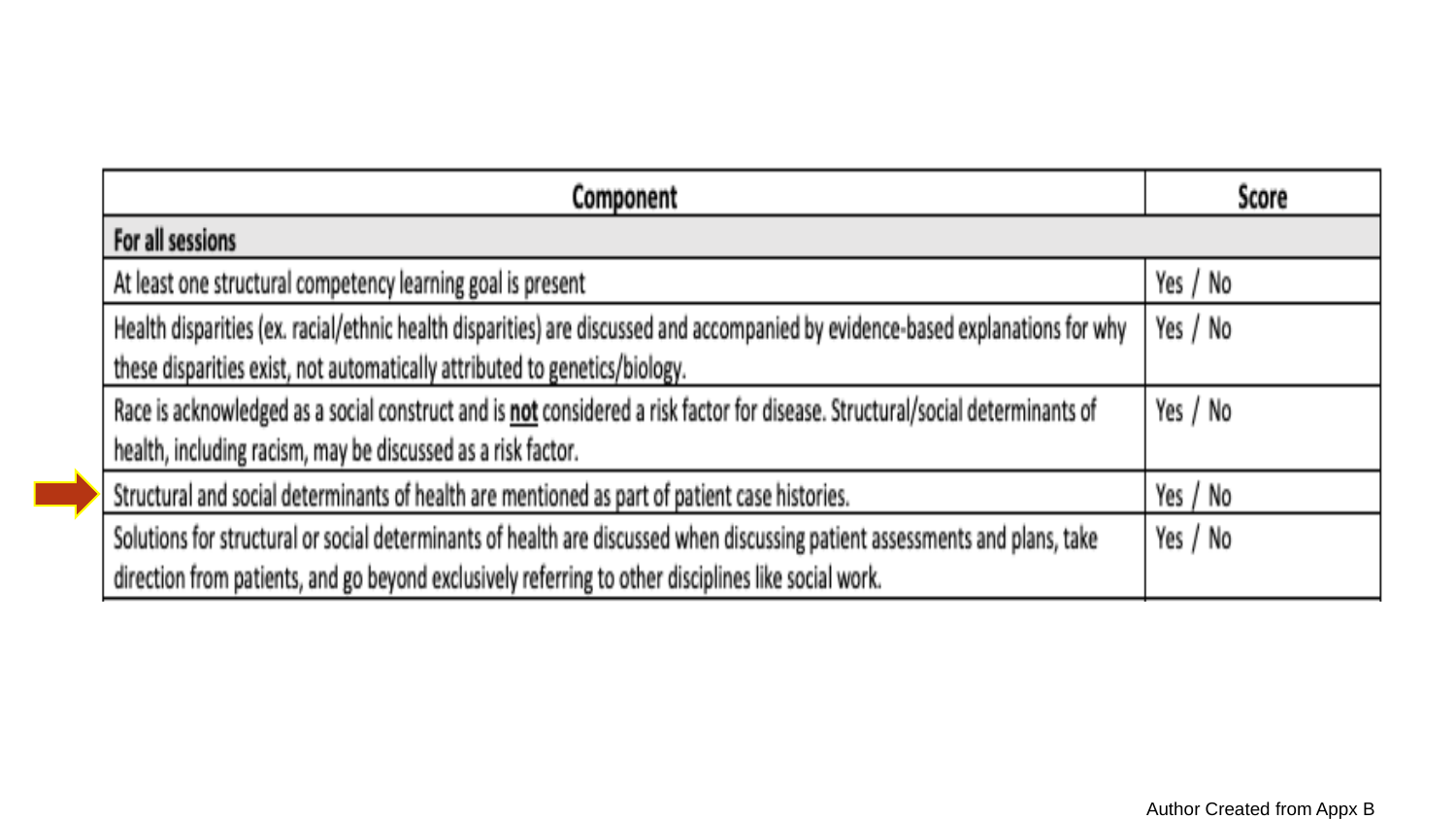

Author Created from Appx B

## Slide 39
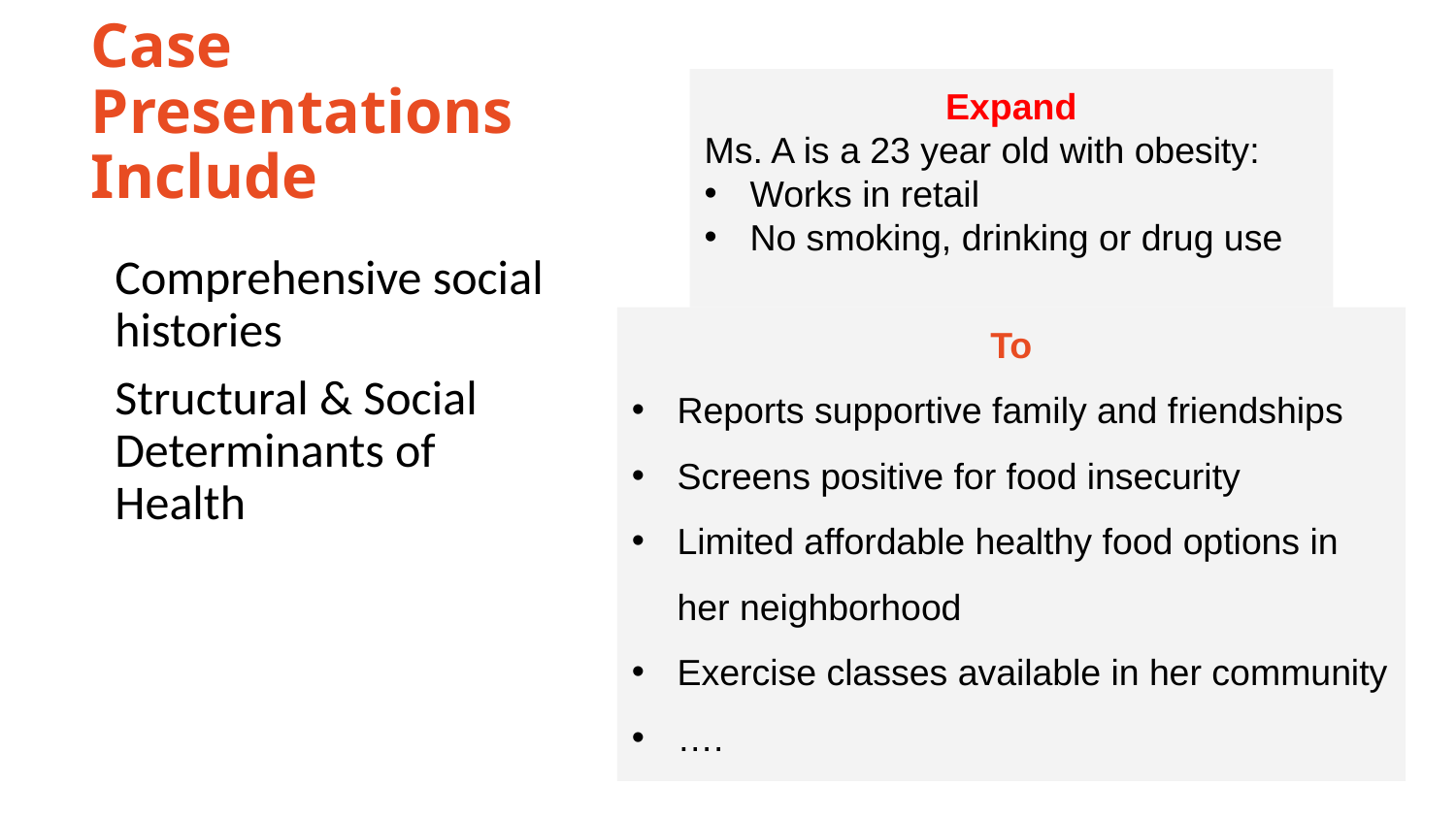

# Case Presentations Include
Expand
Ms. A is a 23 year old with obesity:
Works in retail
No smoking, drinking or drug use
Comprehensive social histories
Structural & Social Determinants of Health
To
Reports supportive family and friendships
Screens positive for food insecurity
Limited affordable healthy food options in her neighborhood
Exercise classes available in her community
….

## Slide 40
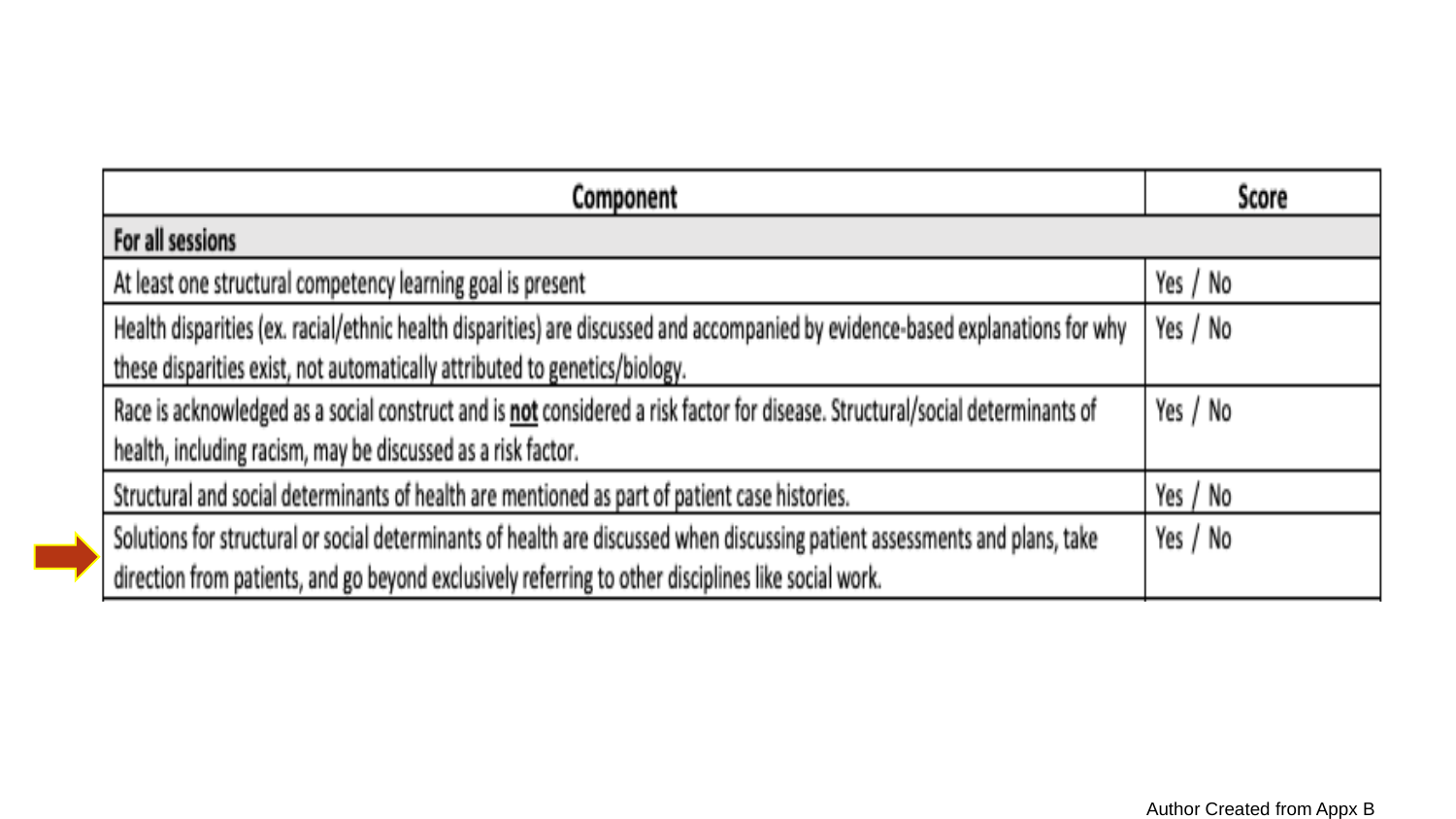

Author Created from Appx B

## Slide 41
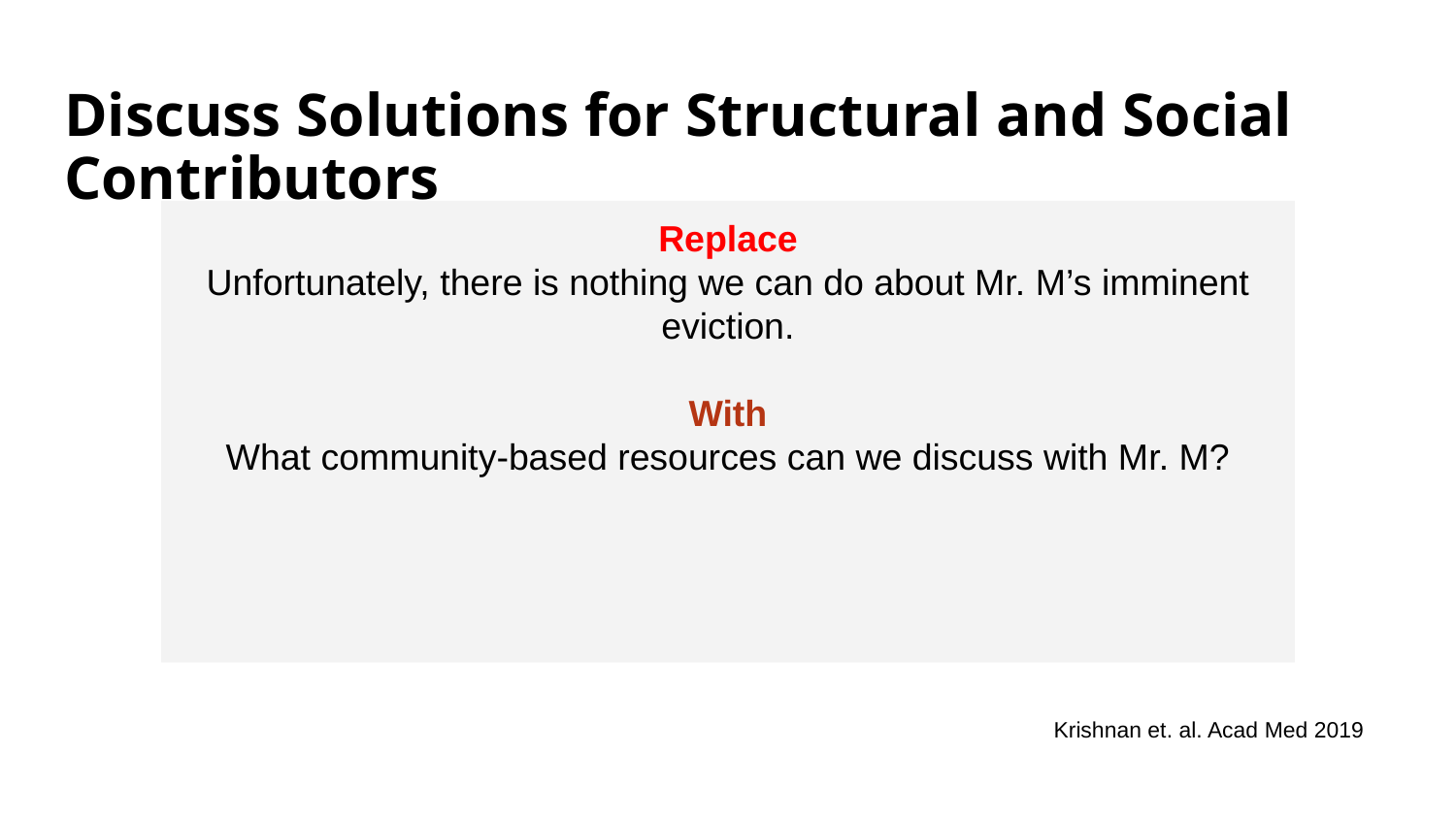

# Discuss Solutions for Structural and Social Contributors
Replace
Unfortunately, there is nothing we can do about Mr. M’s imminent eviction.
With
What community-based resources can we discuss with Mr. M?
Krishnan et. al. Acad Med 2019

## Slide 42
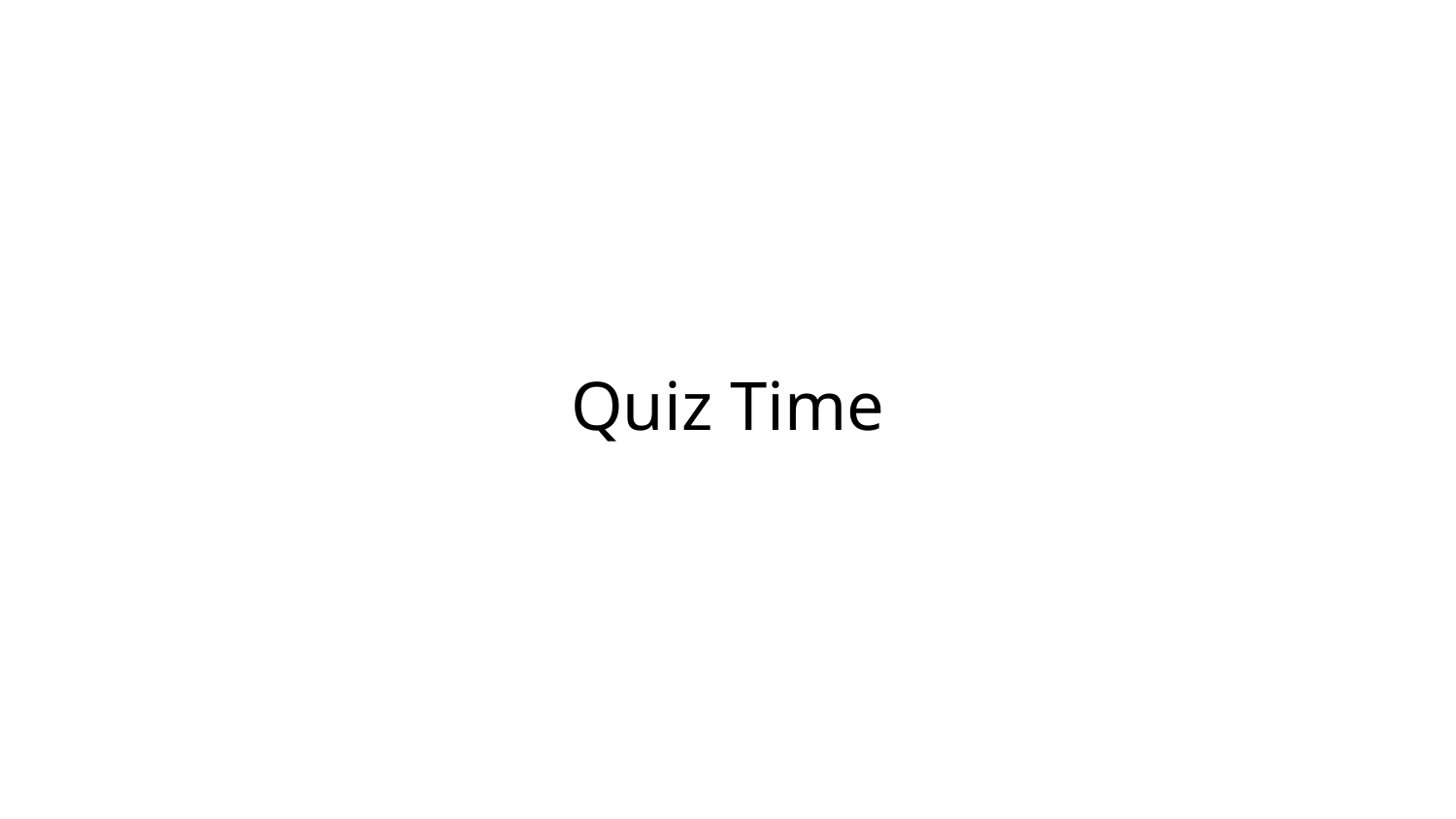

Quiz Time

## Slide 43
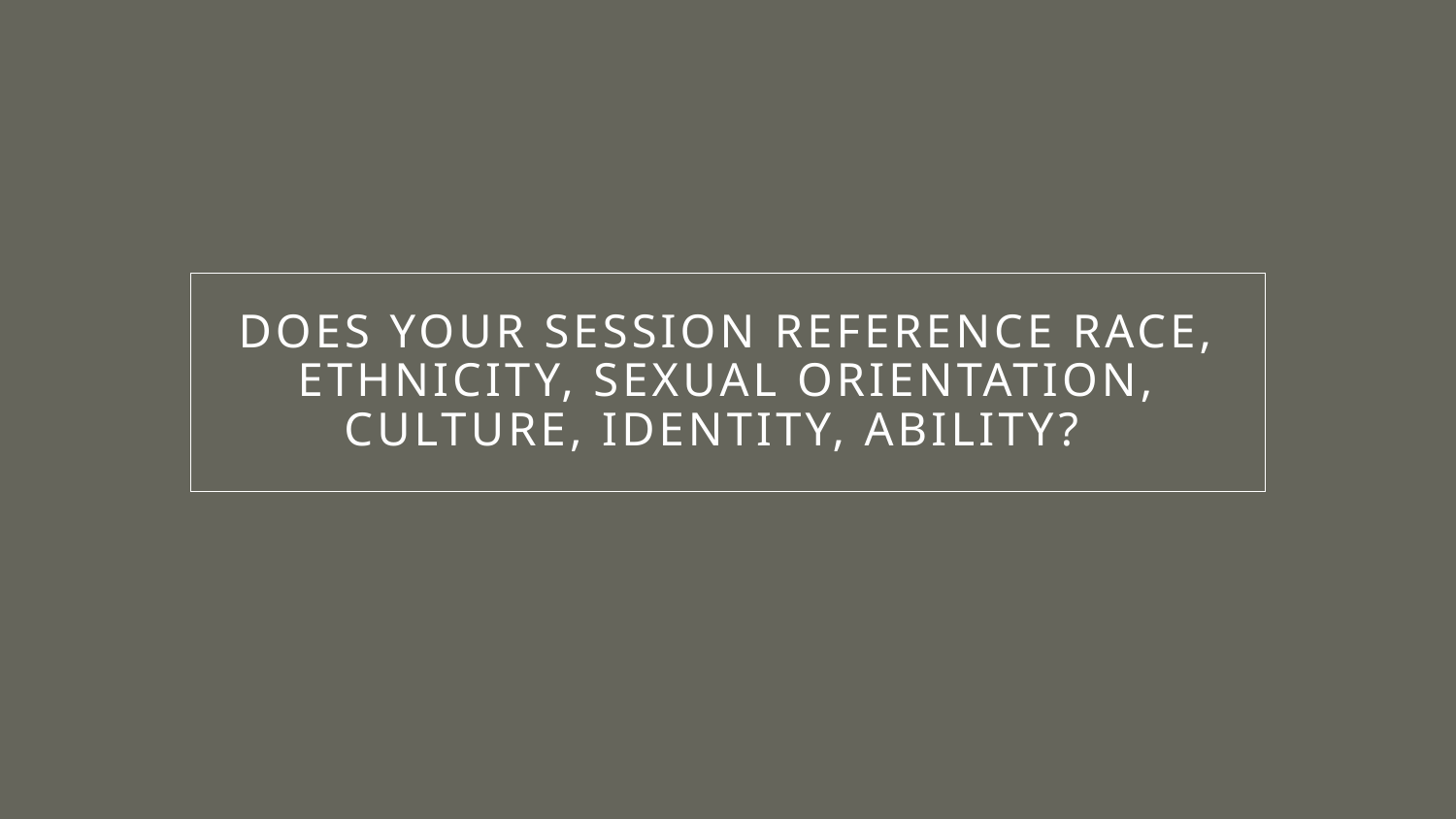

# Does your session reference race, ethnicity, sexual orientation, culture, identity, ability?

## Slide 44
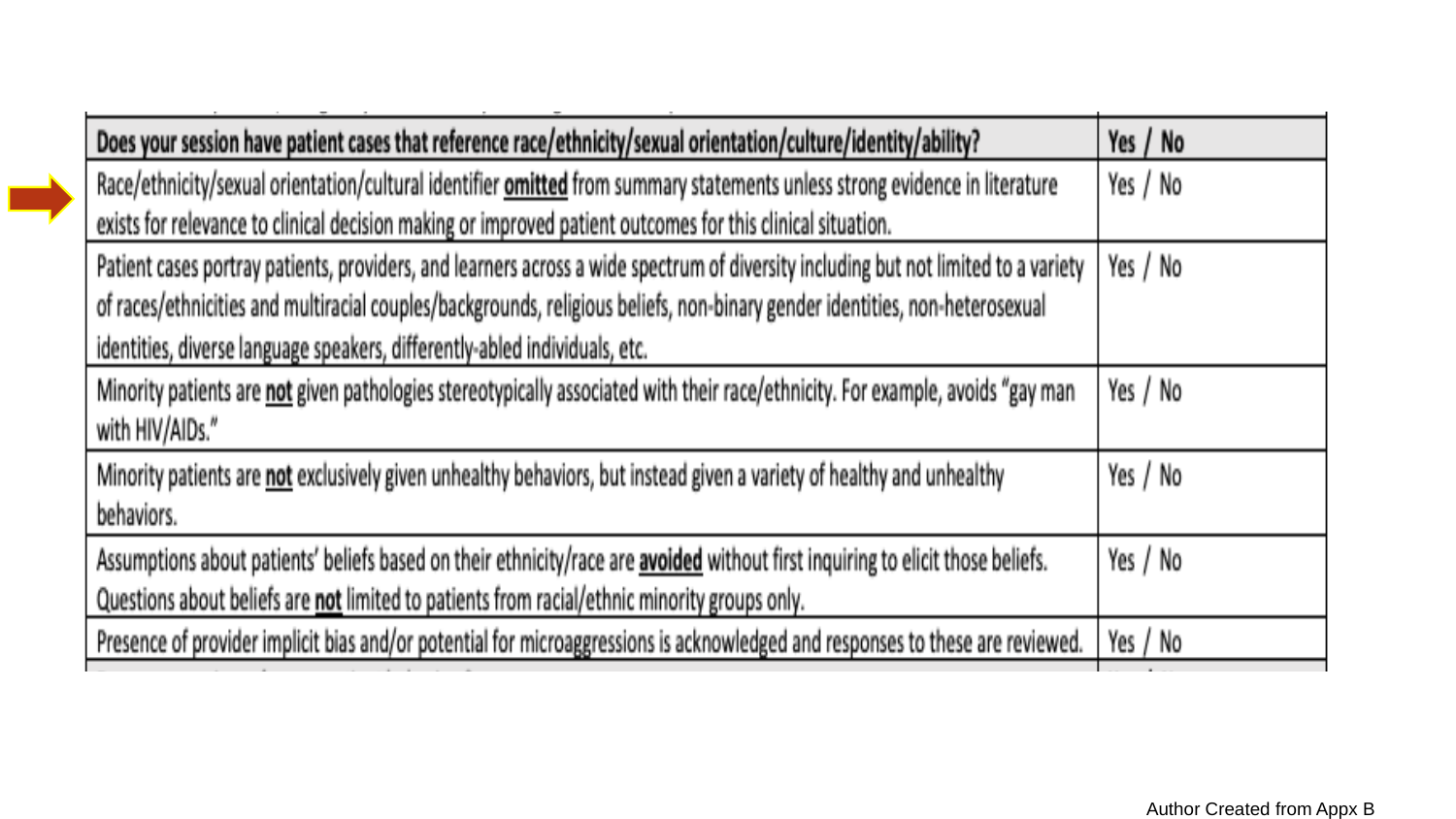

Author Created from Appx B

## Slide 45
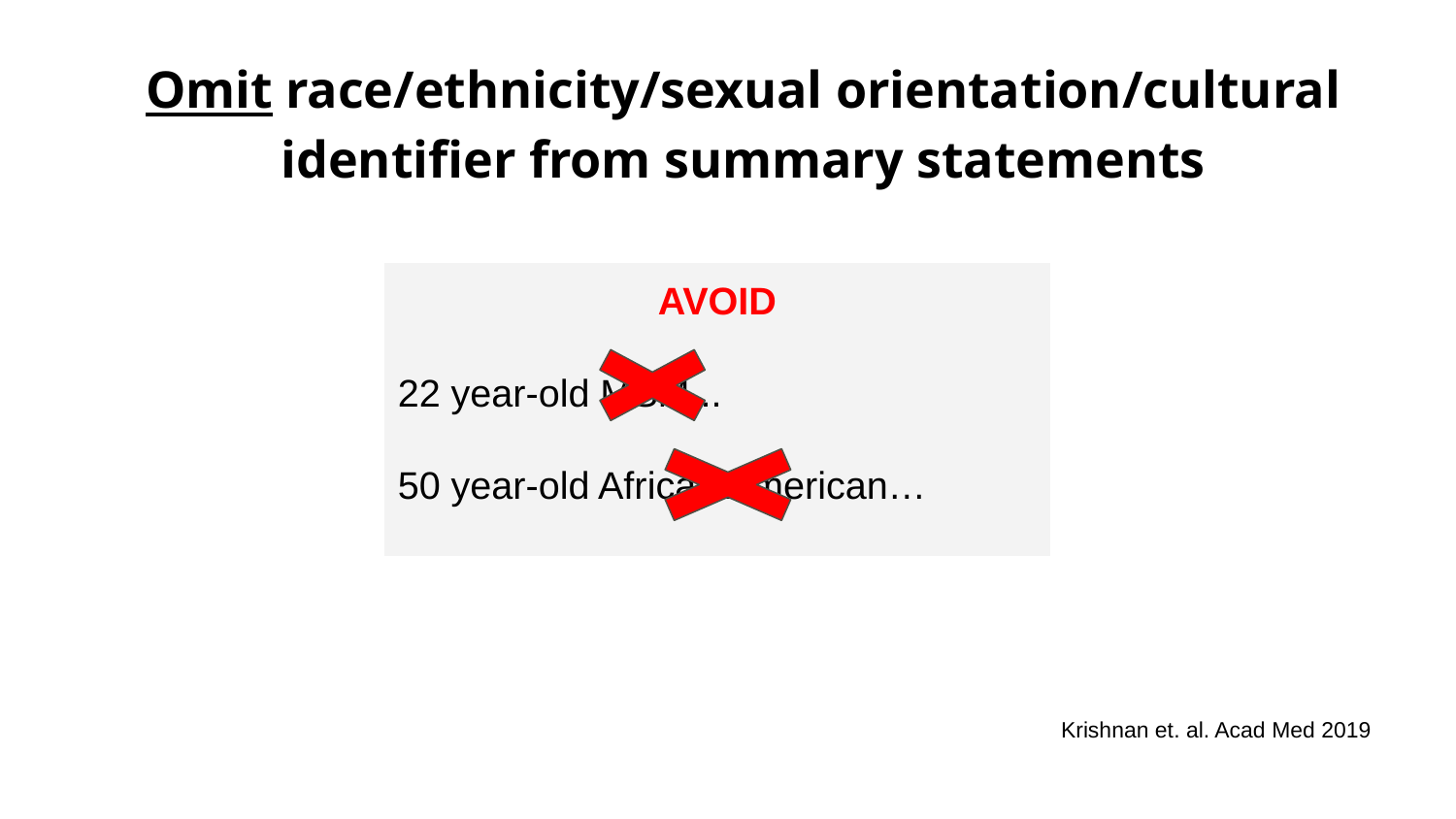

# Omit race/ethnicity/sexual orientation/cultural identifier from summary statements
AVOID
22 year-old MSM...
50 year-old African American…
Krishnan et. al. Acad Med 2019

## Slide 46
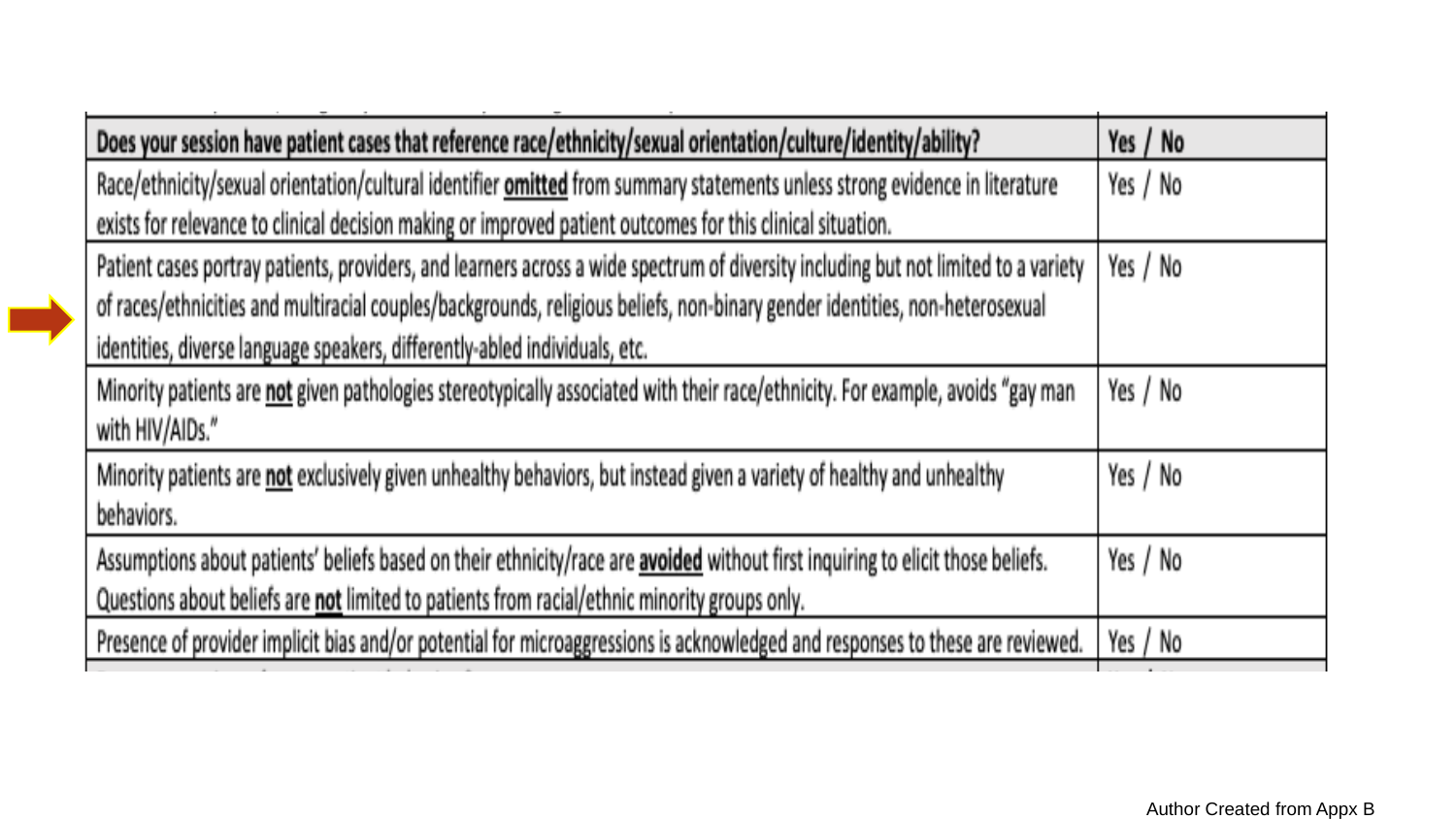

Author Created from Appx B

## Slide 47
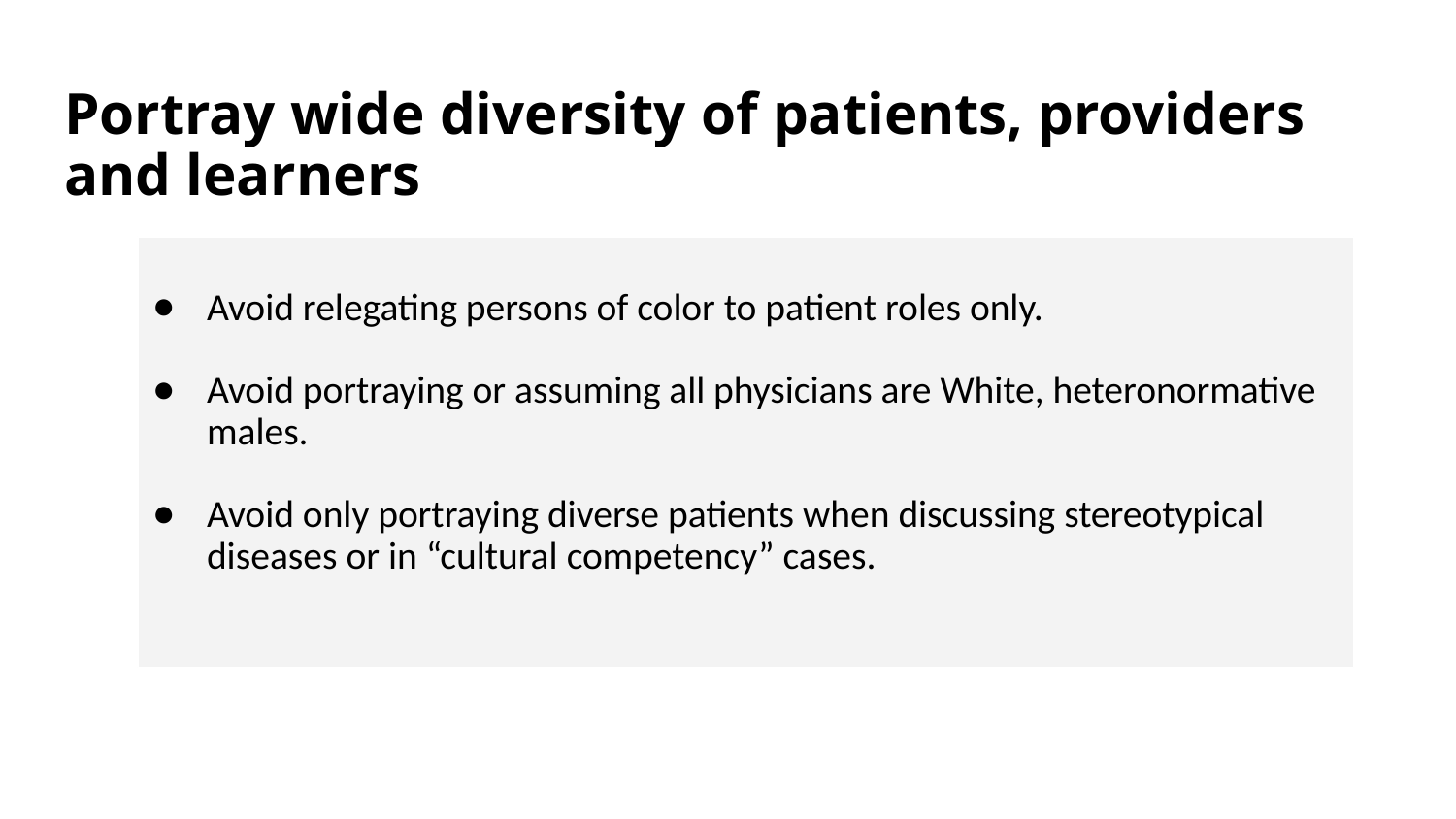

# Portray wide diversity of patients, providers and learners
Avoid relegating persons of color to patient roles only.
Avoid portraying or assuming all physicians are White, heteronormative males.
Avoid only portraying diverse patients when discussing stereotypical diseases or in “cultural competency” cases.

## Slide 48
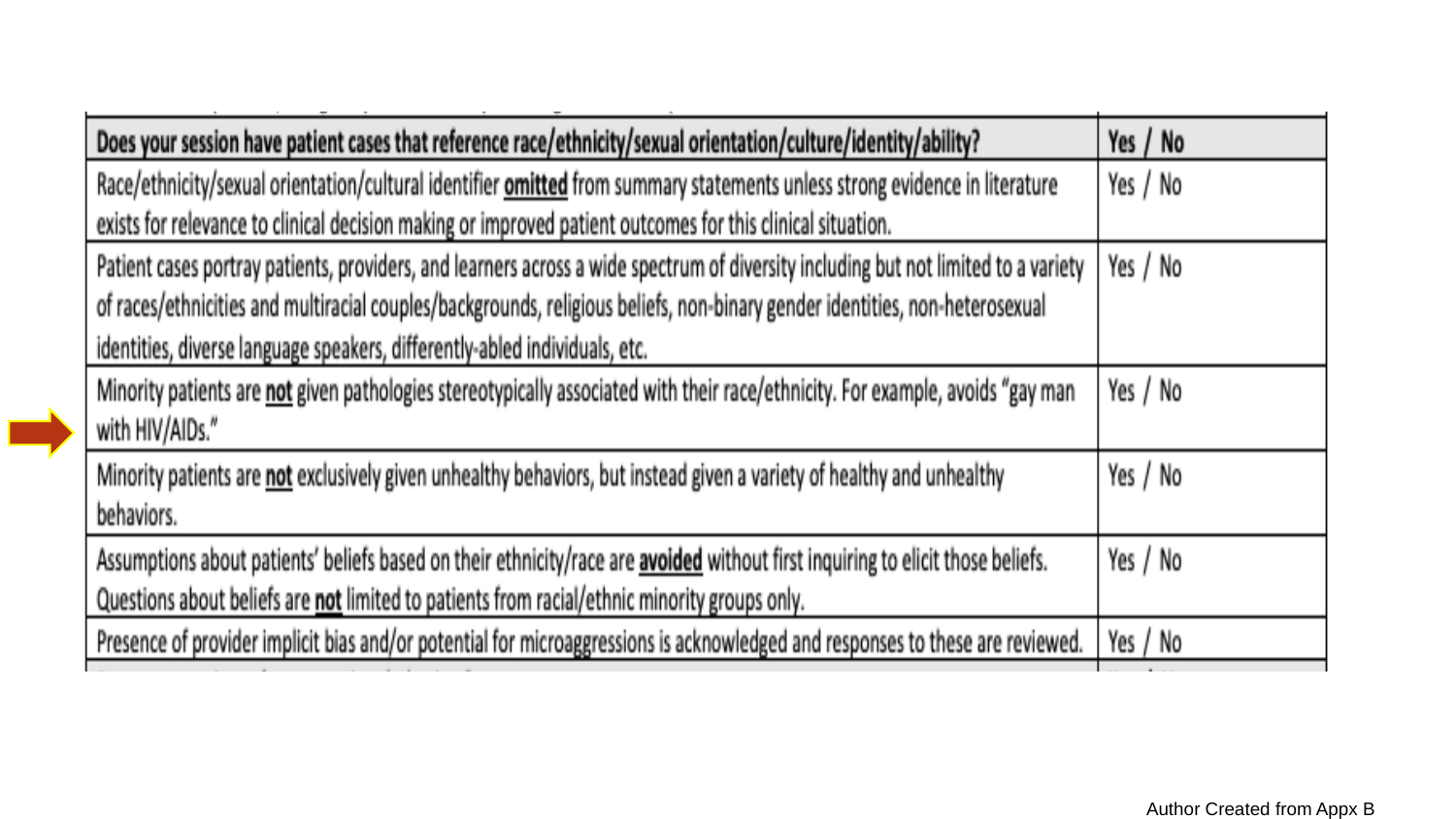

Author Created from Appx B

## Slide 49
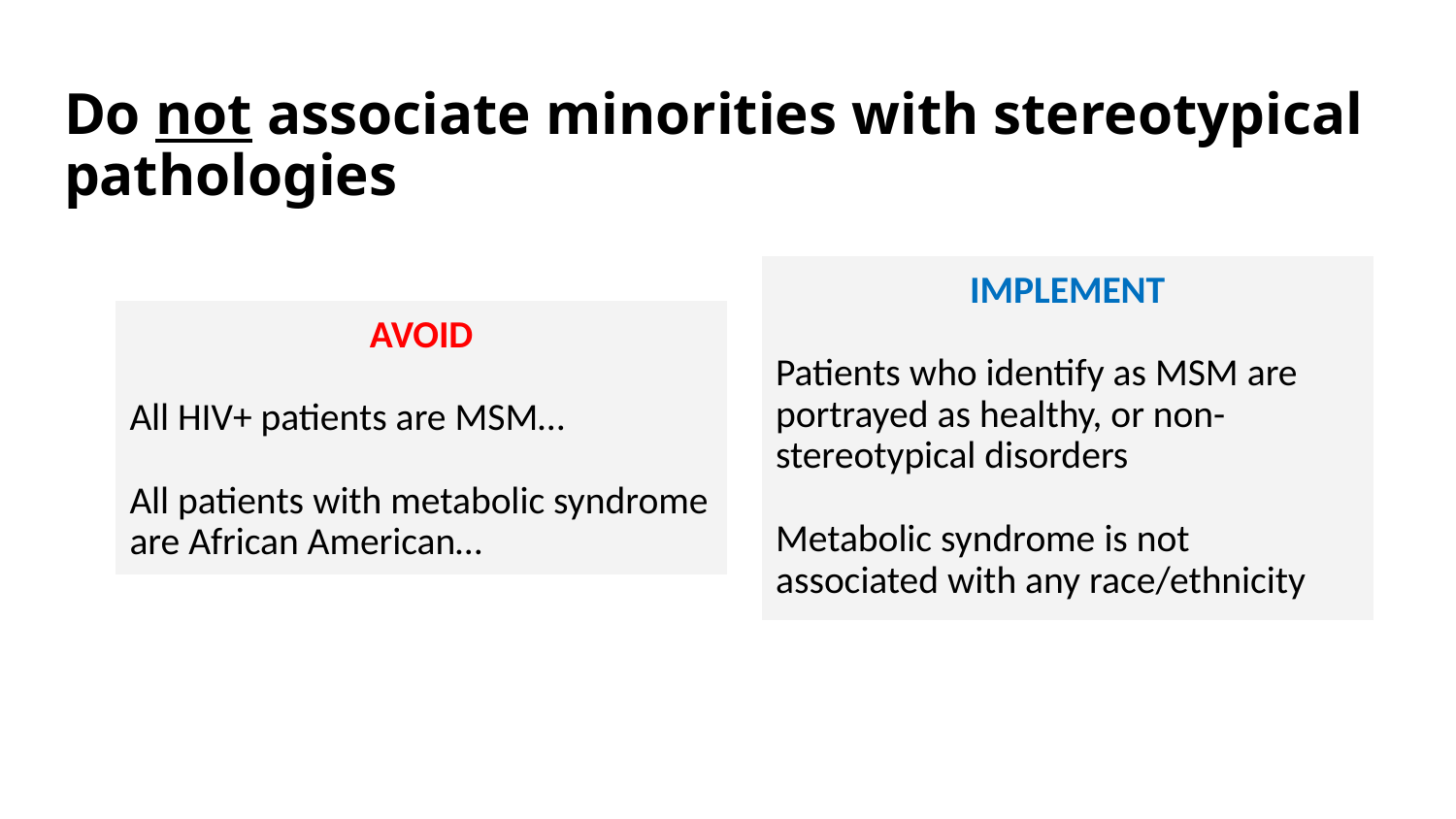

# Do not associate minorities with stereotypical pathologies
IMPLEMENT
Patients who identify as MSM are portrayed as healthy, or non-stereotypical disorders
Metabolic syndrome is not associated with any race/ethnicity
AVOID
All HIV+ patients are MSM…
All patients with metabolic syndrome are African American…

## Slide 50
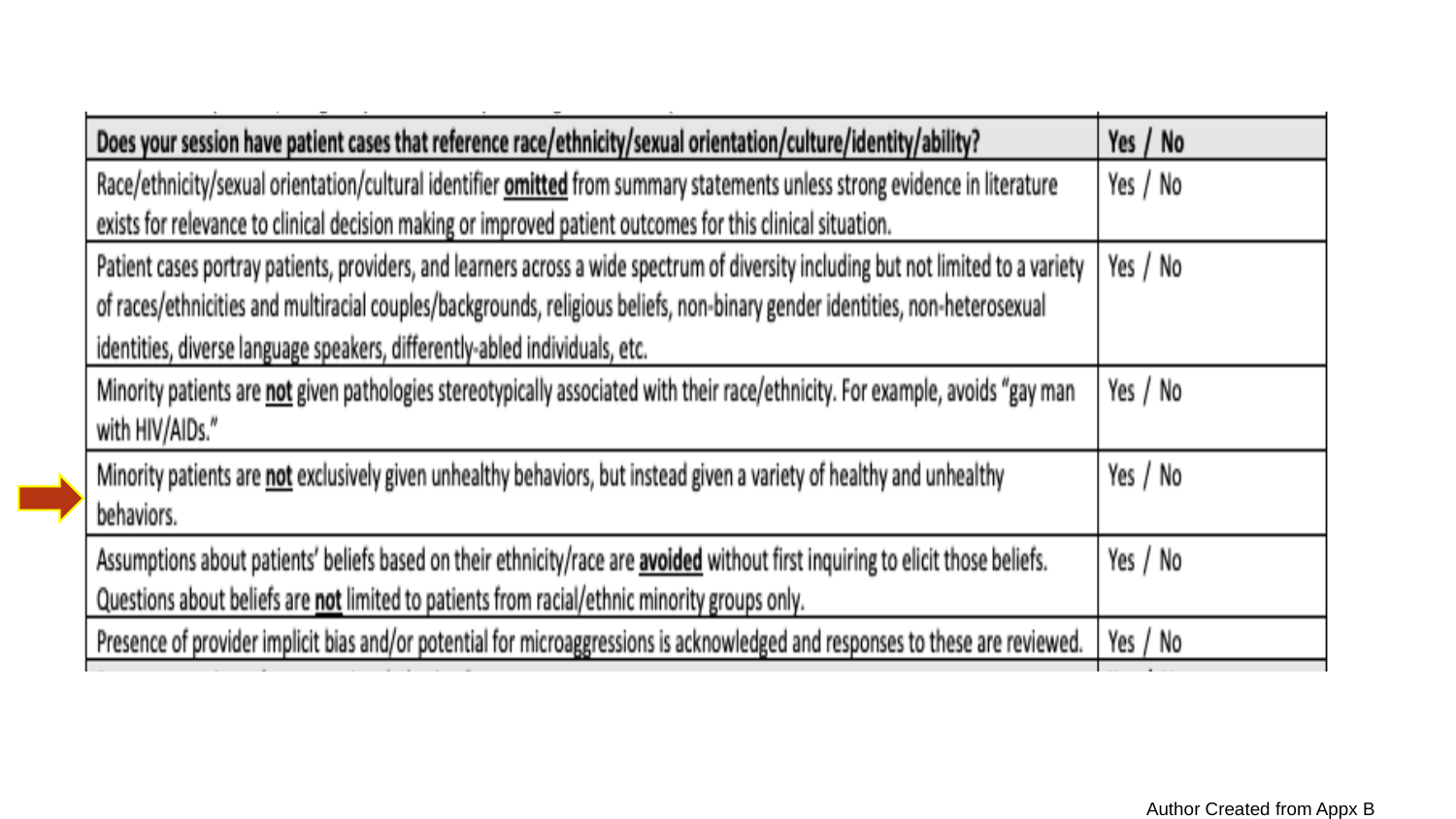

Author Created from Appx B

## Slide 51
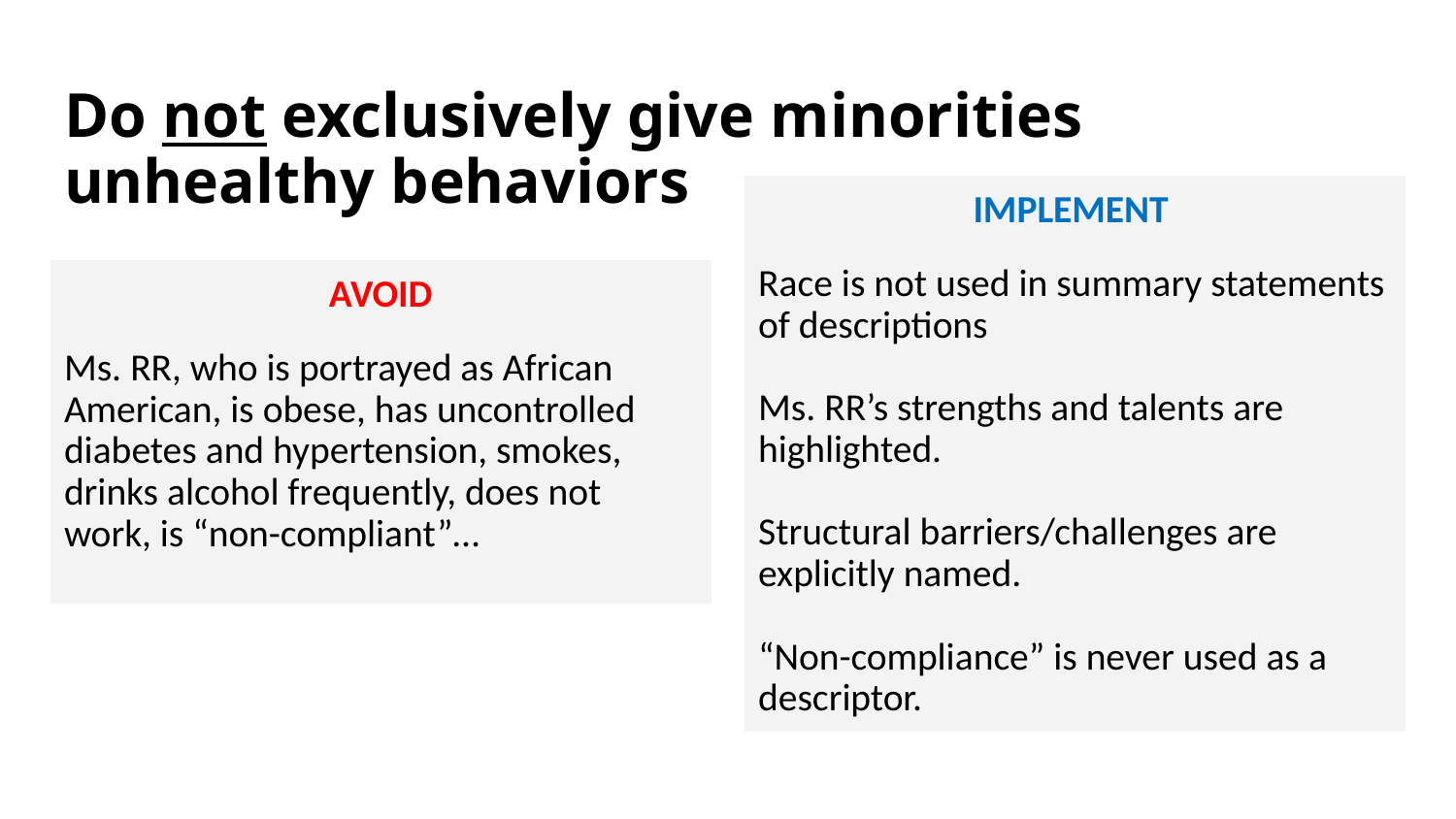

# Do not exclusively give minorities unhealthy behaviors
IMPLEMENT
Race is not used in summary statements of descriptions
Ms. RR’s strengths and talents are highlighted.
Structural barriers/challenges are explicitly named.
“Non-compliance” is never used as a descriptor.
AVOID
Ms. RR, who is portrayed as African American, is obese, has uncontrolled diabetes and hypertension, smokes, drinks alcohol frequently, does not work, is “non-compliant”…

## Slide 52
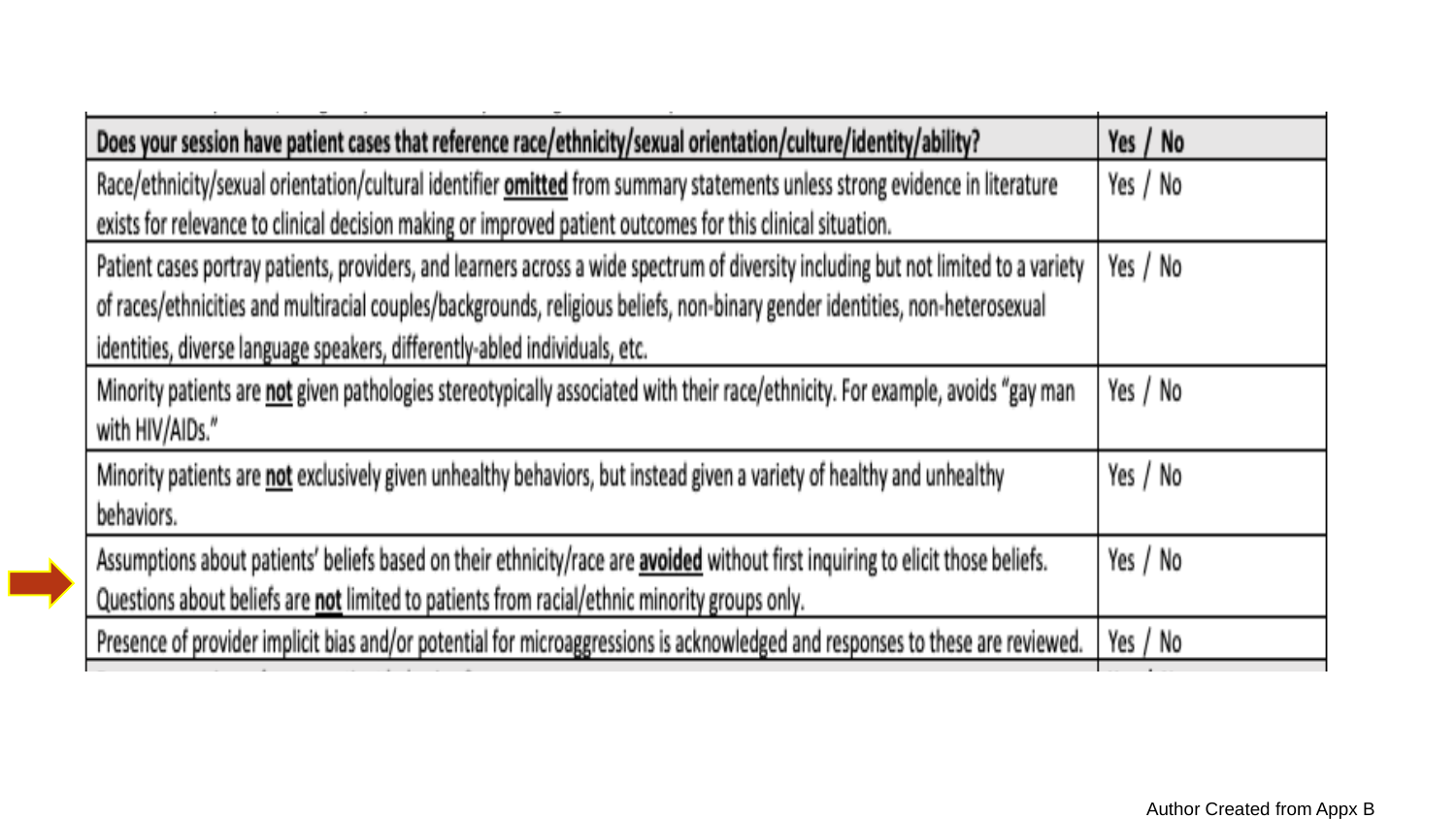

Author Created from Appx B

## Slide 53
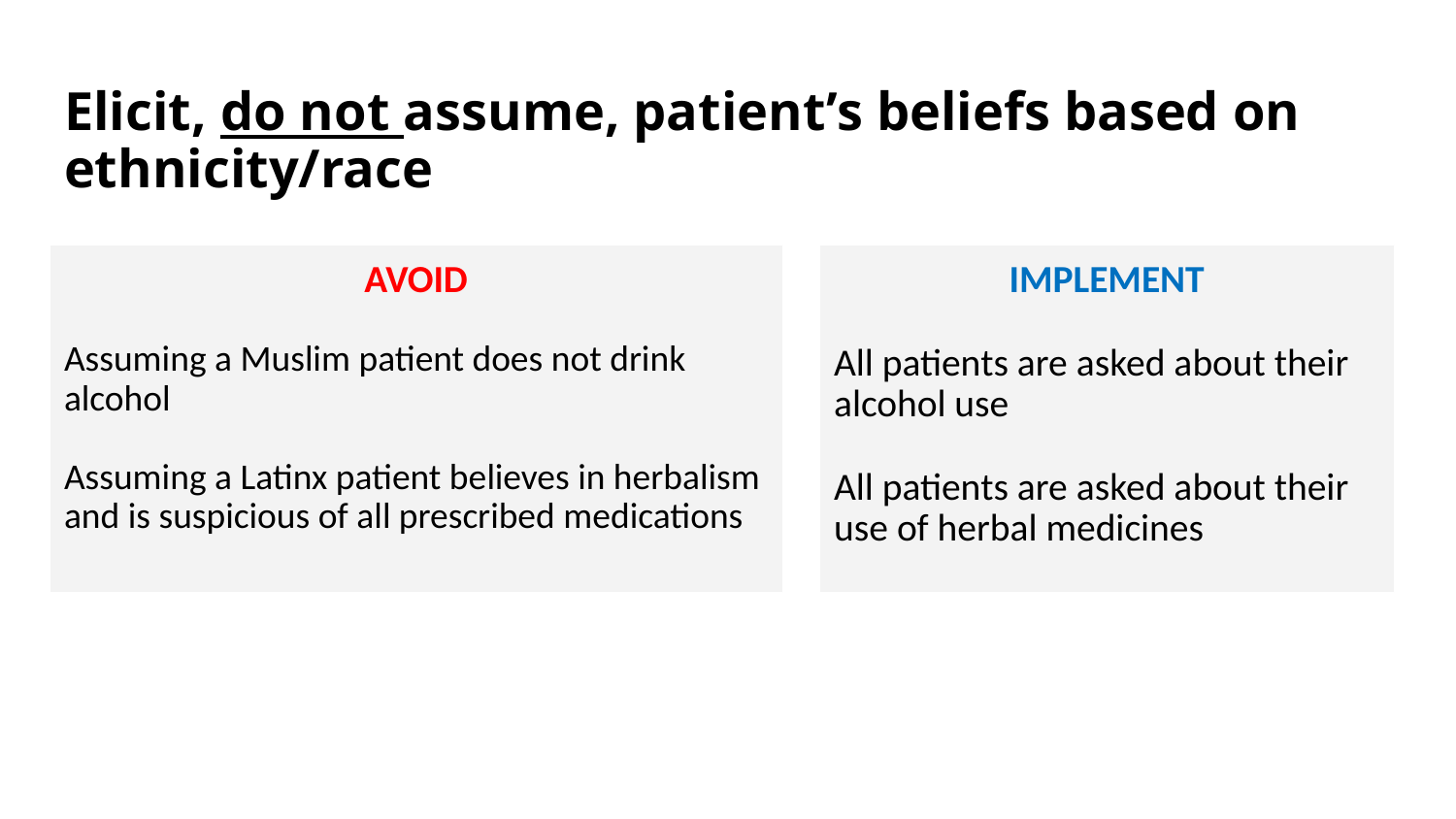

# Elicit, do not assume, patient’s beliefs based on ethnicity/race
AVOID
Assuming a Muslim patient does not drink alcohol
Assuming a Latinx patient believes in herbalism and is suspicious of all prescribed medications
IMPLEMENT
All patients are asked about their alcohol use
All patients are asked about their use of herbal medicines

## Slide 54
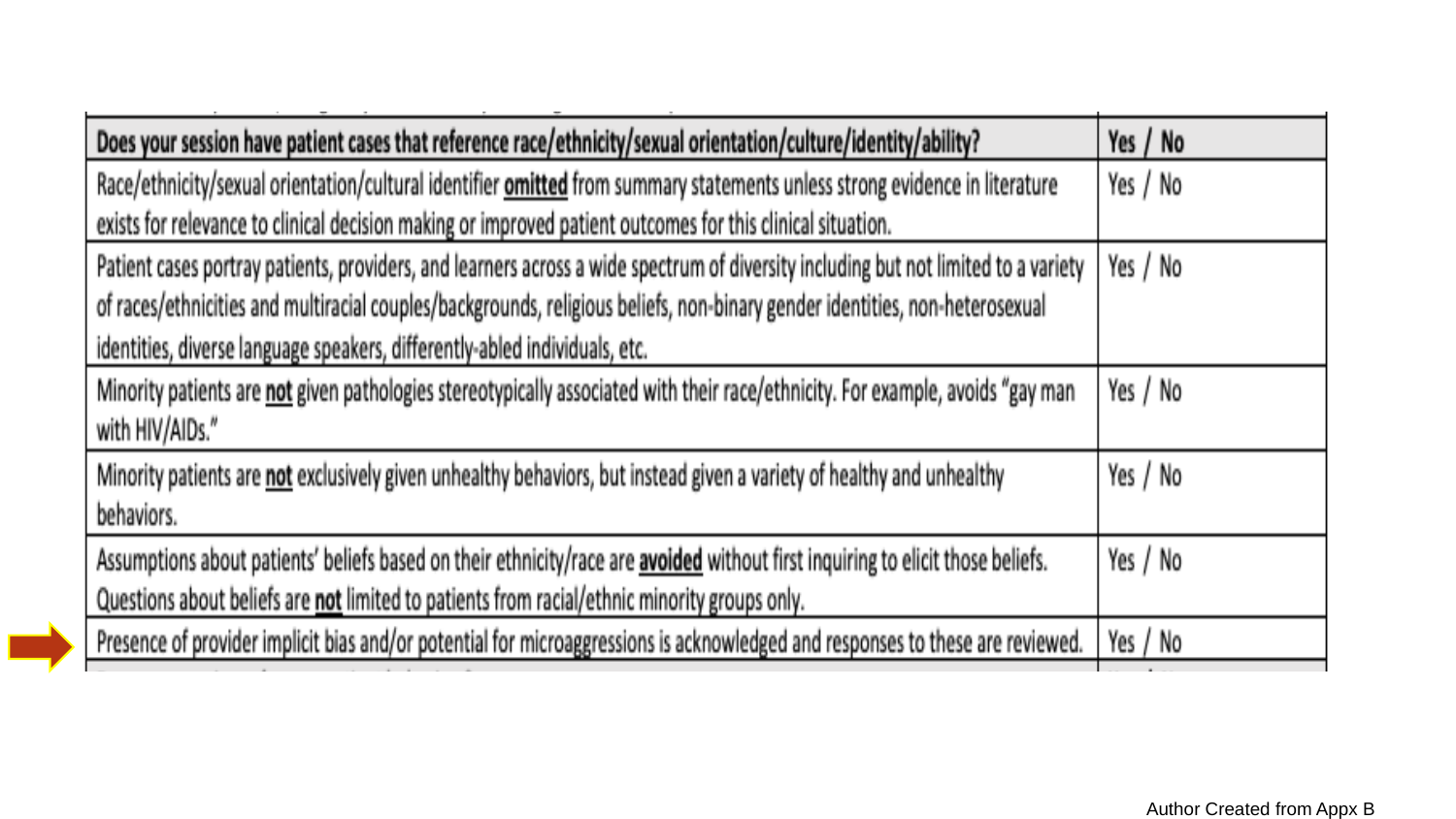

Author Created from Appx B

## Slide 55
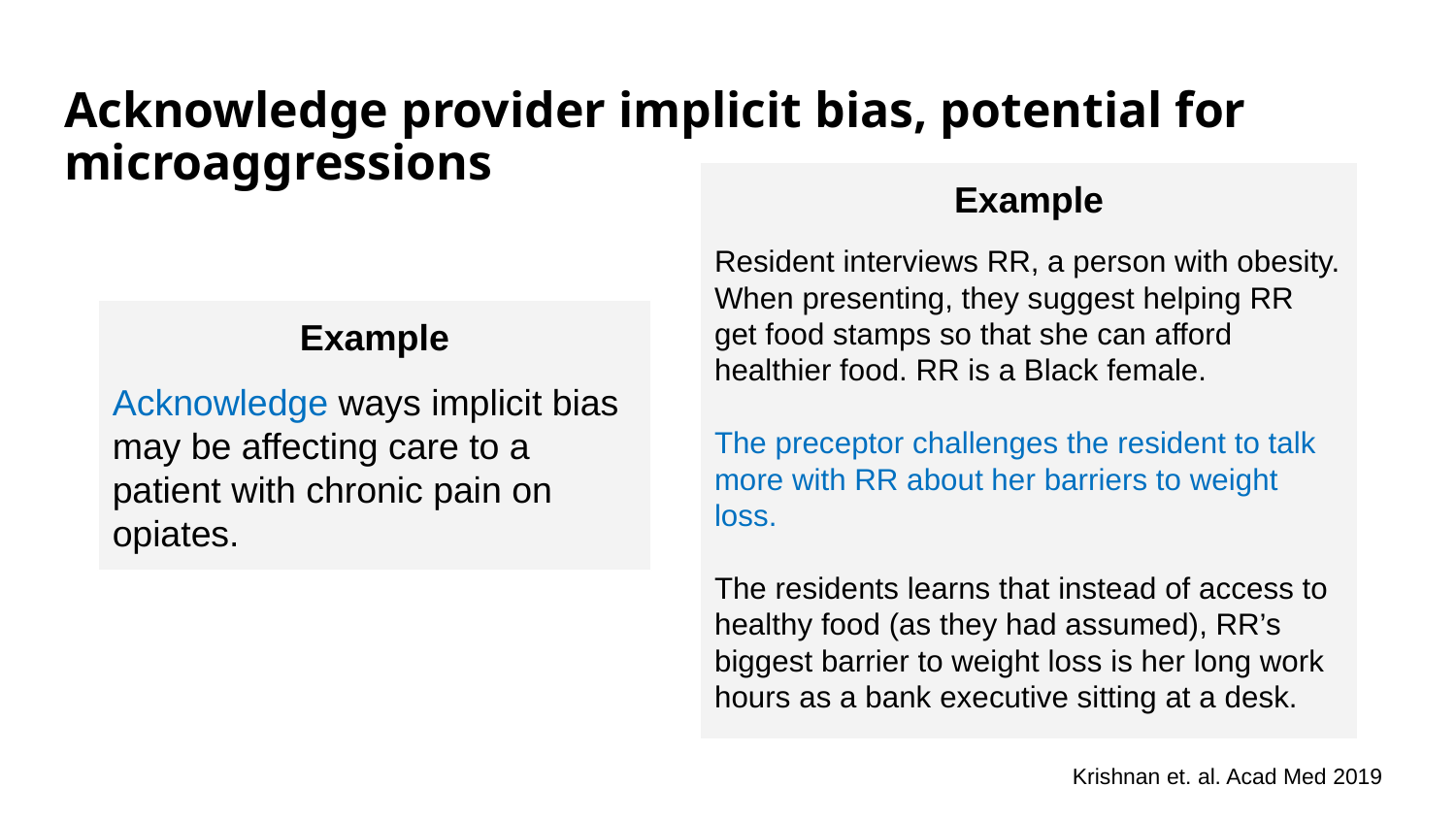

# Acknowledge provider implicit bias, potential for microaggressions
Example
Resident interviews RR, a person with obesity. When presenting, they suggest helping RR get food stamps so that she can afford healthier food. RR is a Black female.
The preceptor challenges the resident to talk more with RR about her barriers to weight loss.
The residents learns that instead of access to healthy food (as they had assumed), RR’s biggest barrier to weight loss is her long work hours as a bank executive sitting at a desk.
Example
Acknowledge ways implicit bias may be affecting care to a patient with chronic pain on opiates.
Krishnan et. al. Acad Med 2019

## Slide 56
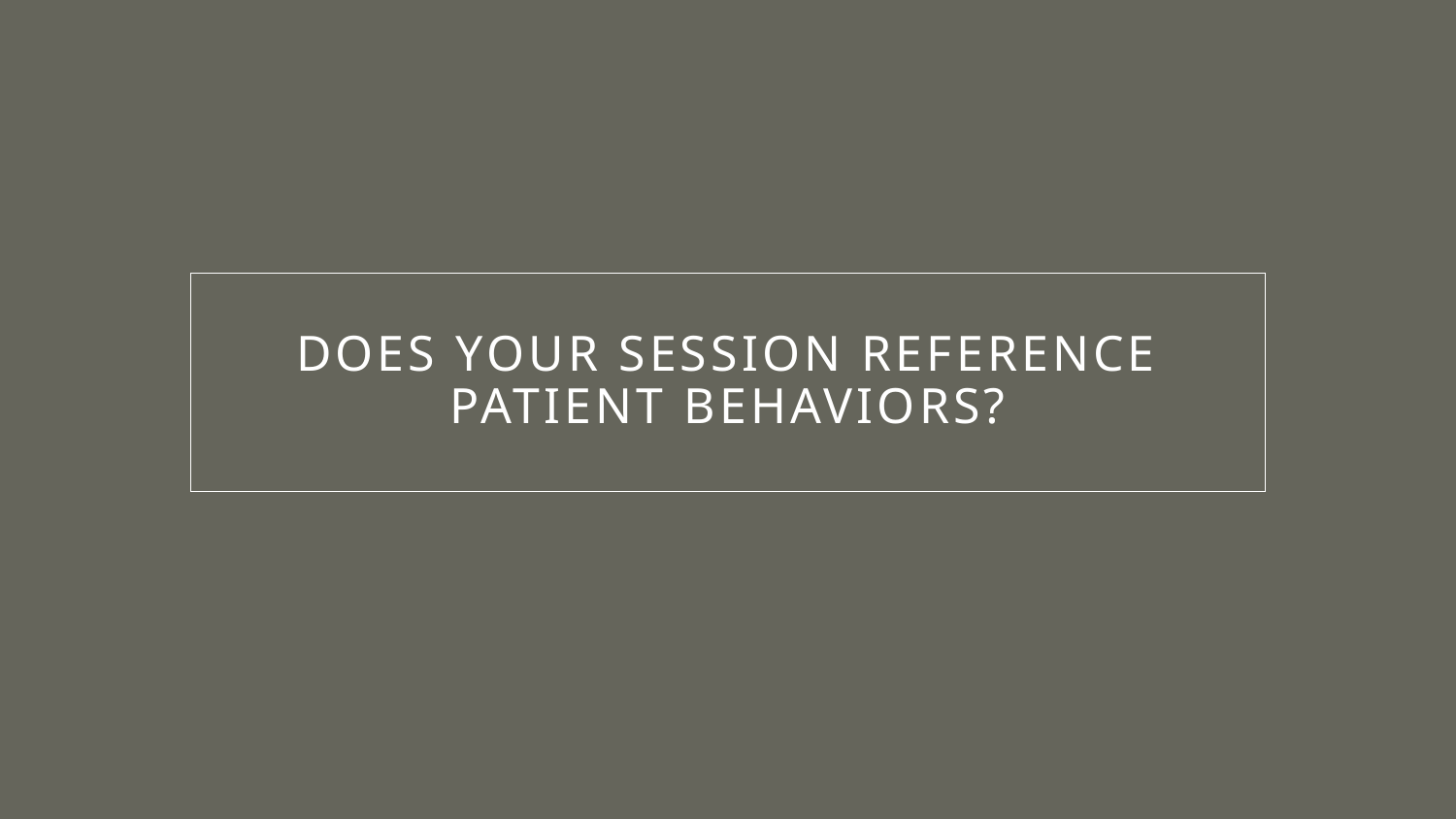

# Does your session reference patient behaviors?

## Slide 57
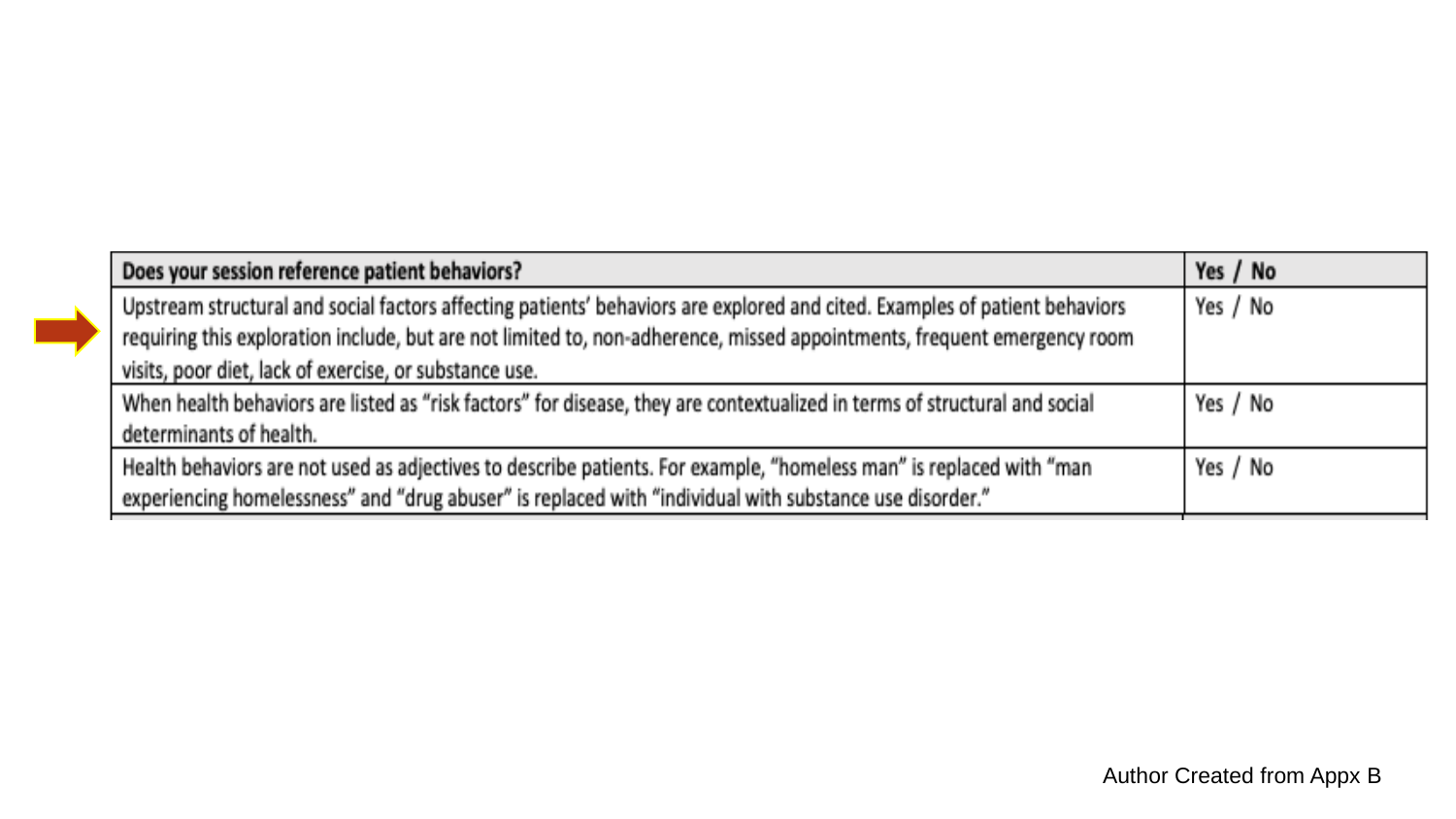

Author Created from Appx B

## Slide 58
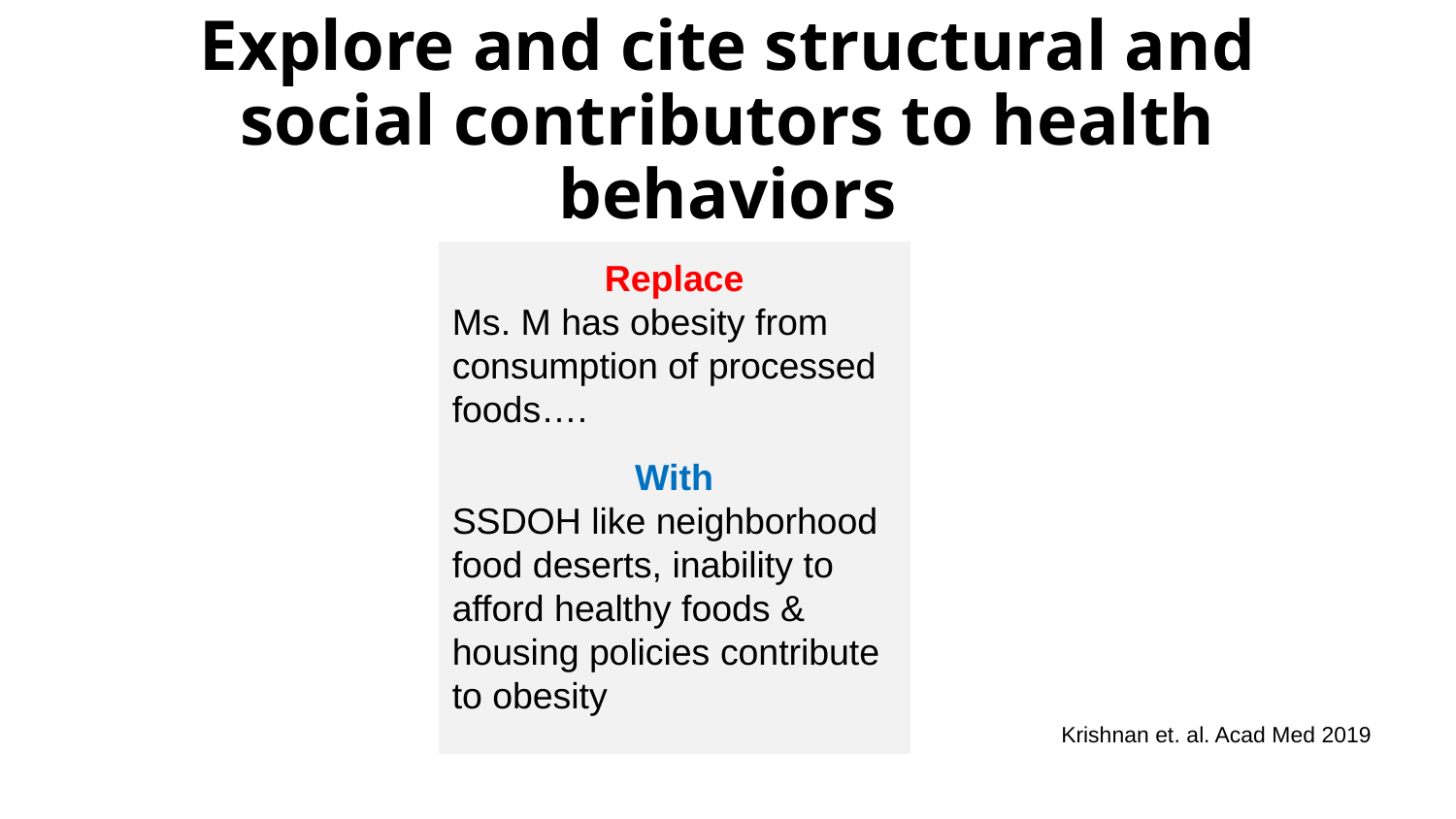

# Explore and cite structural and social contributors to health behaviors
Replace
Ms. M has obesity from consumption of processed foods….
With
SSDOH like neighborhood food deserts, inability to afford healthy foods & housing policies contribute to obesity
Krishnan et. al. Acad Med 2019

## Slide 59
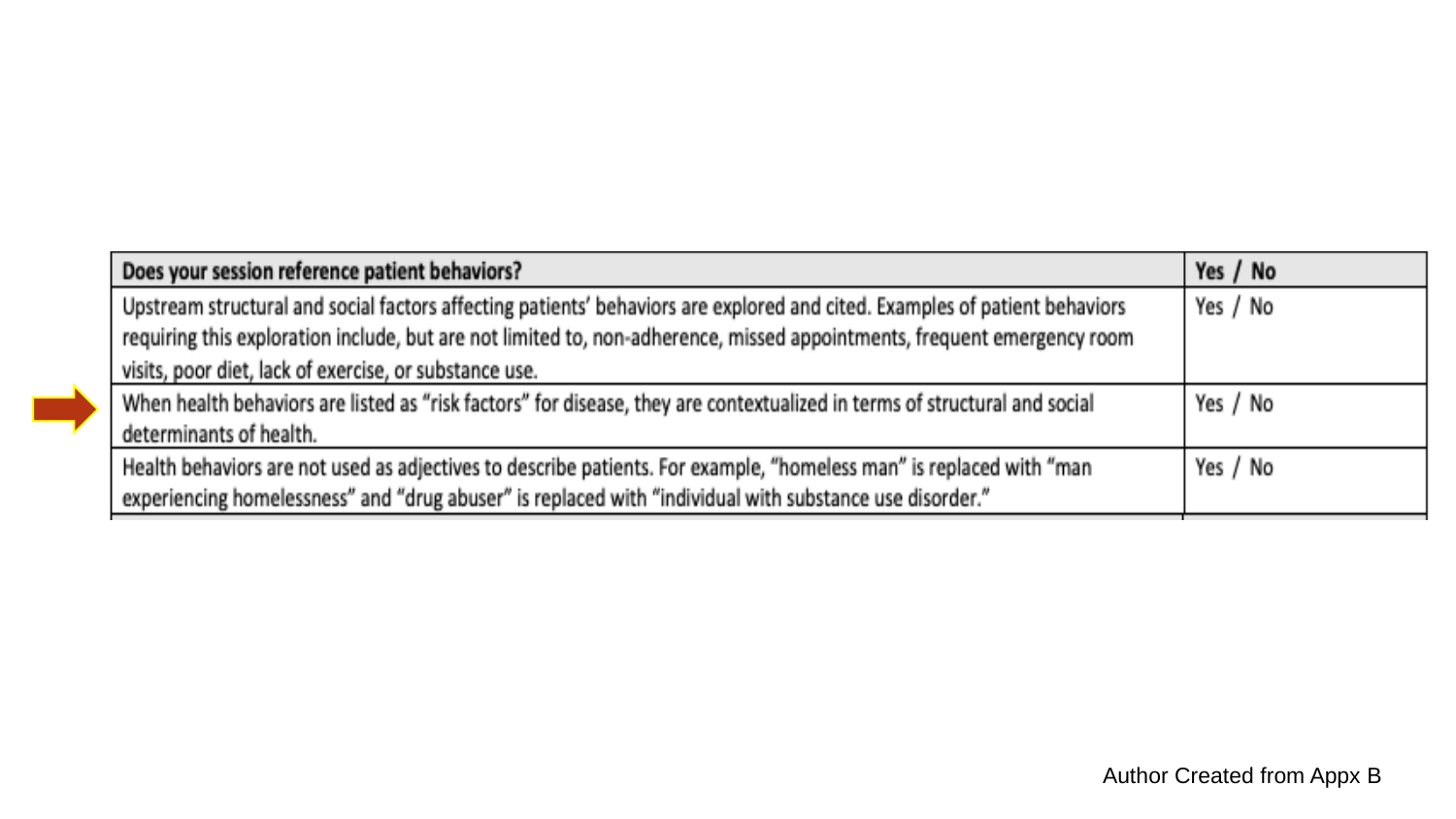

Author Created from Appx B

## Slide 60
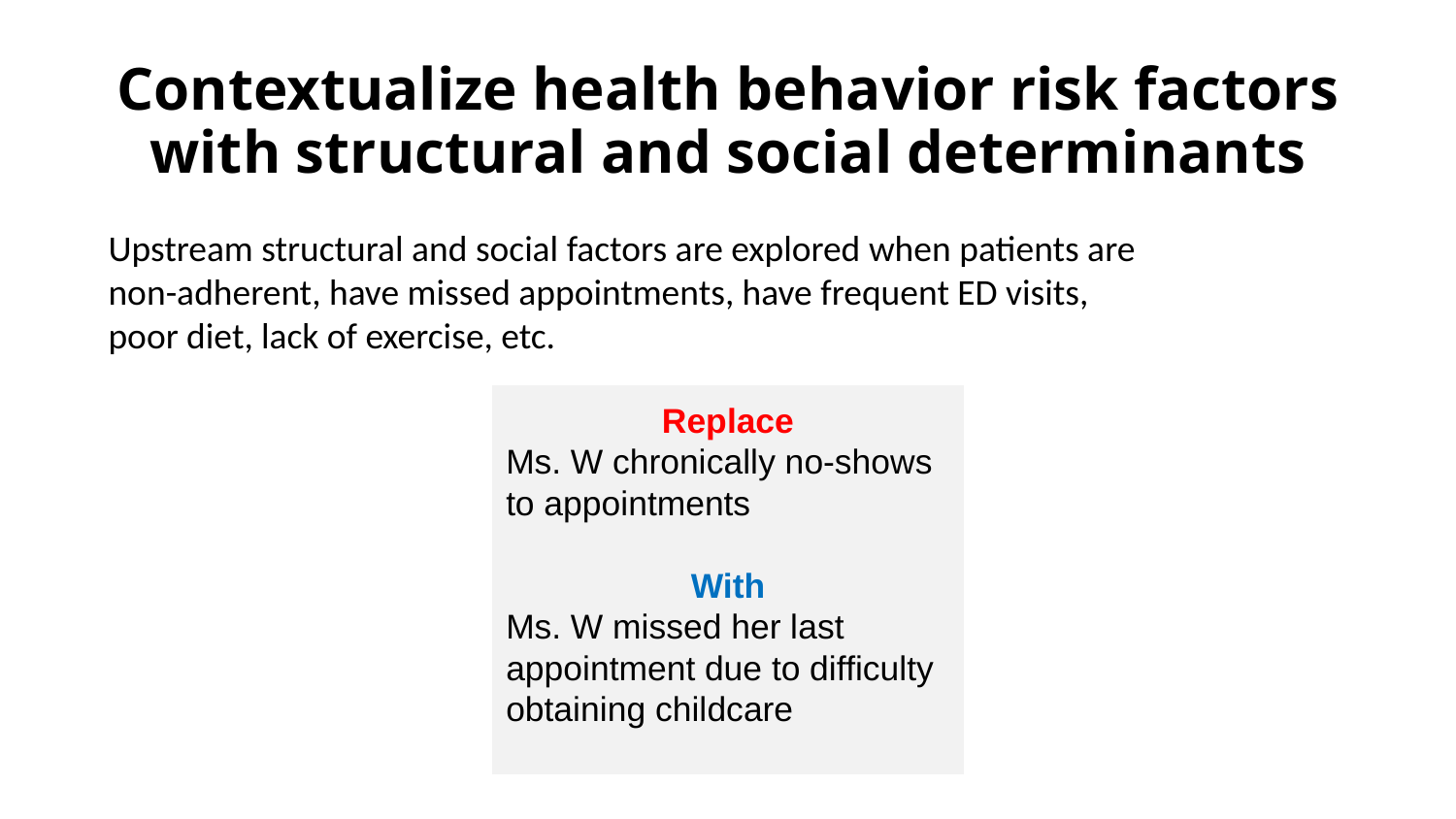

# Contextualize health behavior risk factors with structural and social determinants
Upstream structural and social factors are explored when patients are
non-adherent, have missed appointments, have frequent ED visits,
poor diet, lack of exercise, etc.
Replace
Ms. W chronically no-shows to appointments
With
Ms. W missed her last appointment due to difficulty obtaining childcare

## Slide 61
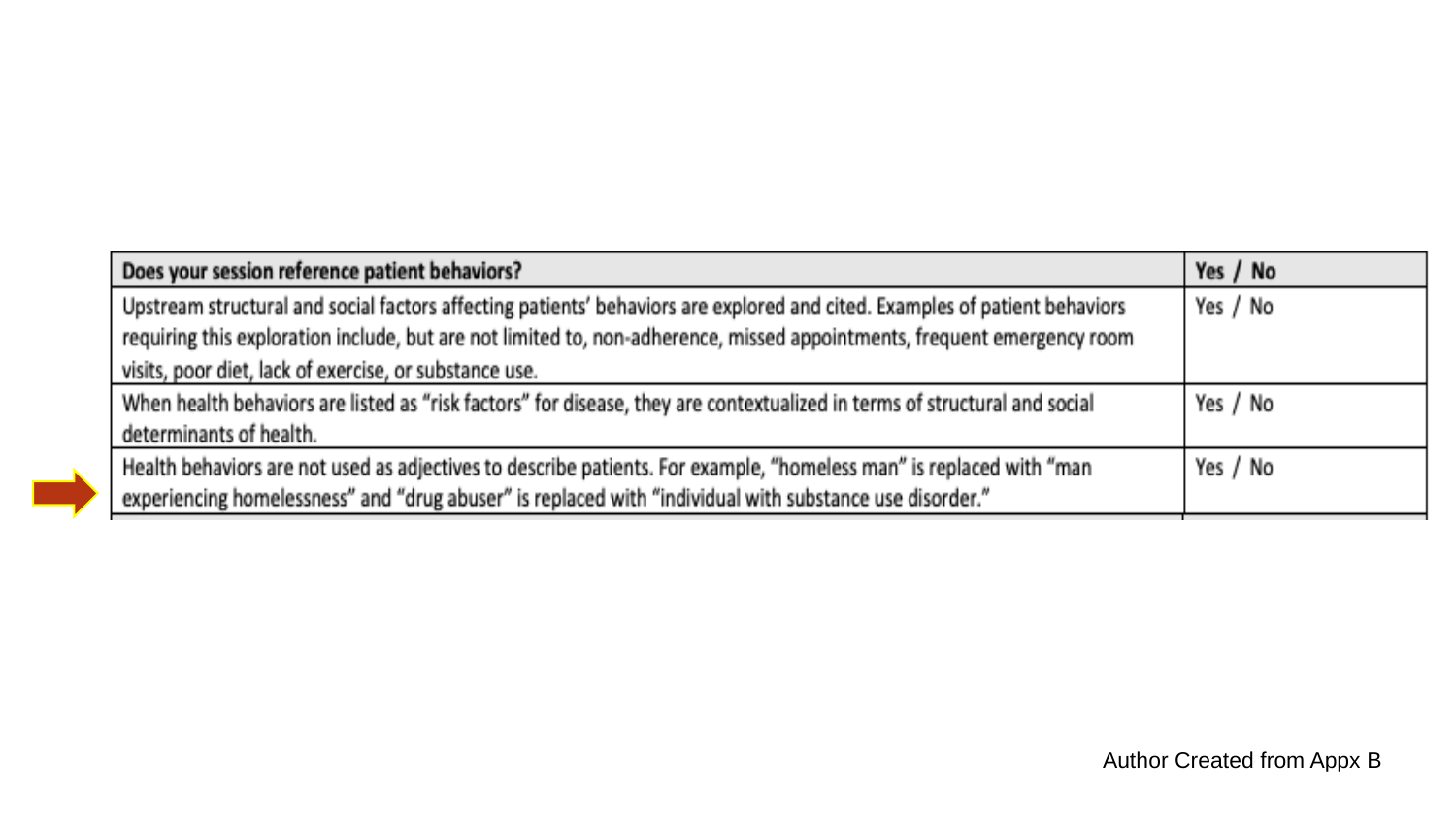

Author Created from Appx B

## Slide 62
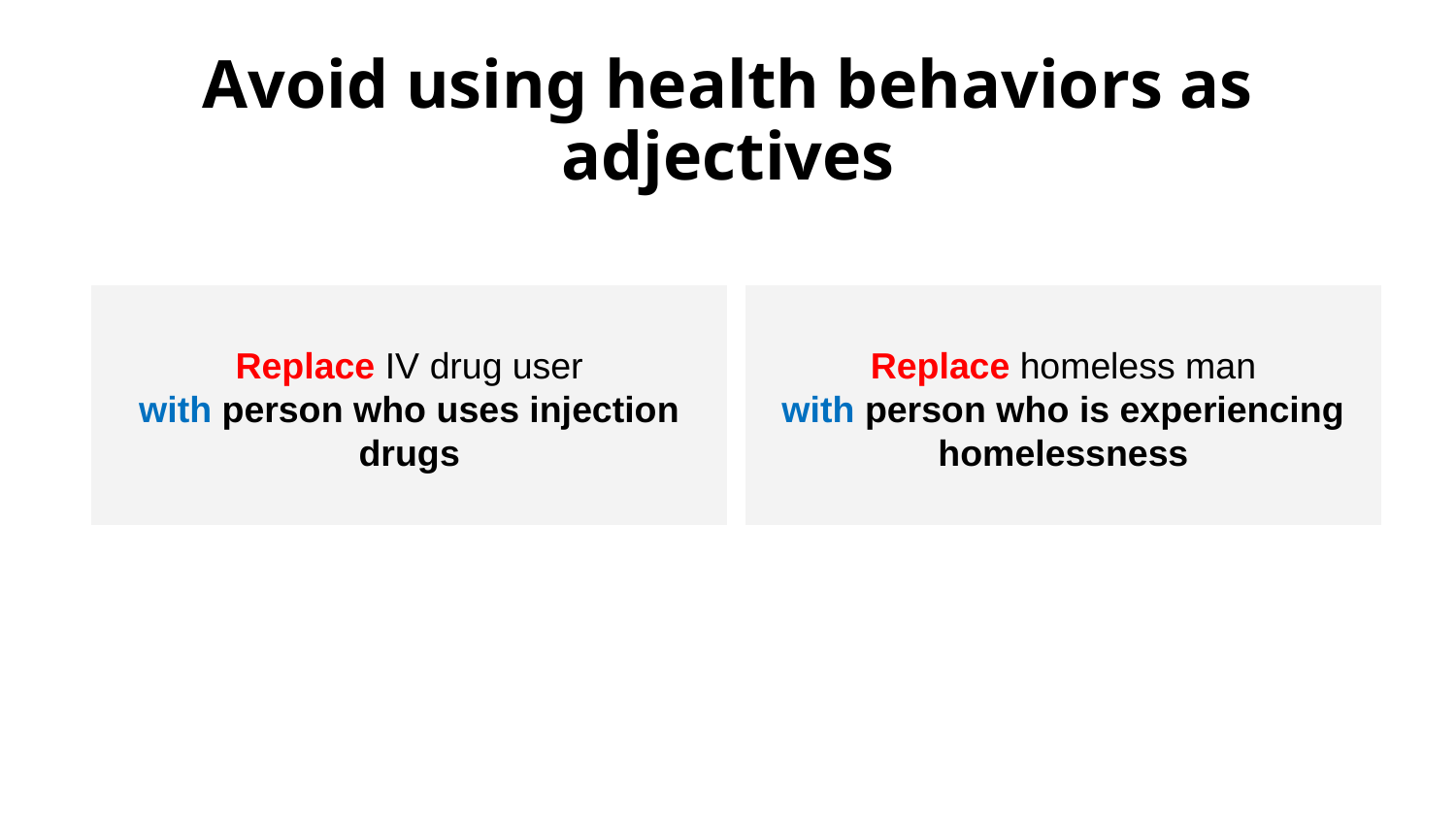

# Avoid using health behaviors as adjectives
Replace IV drug user
with person who uses injection drugs
Replace homeless man
with person who is experiencing homelessness

## Slide 63
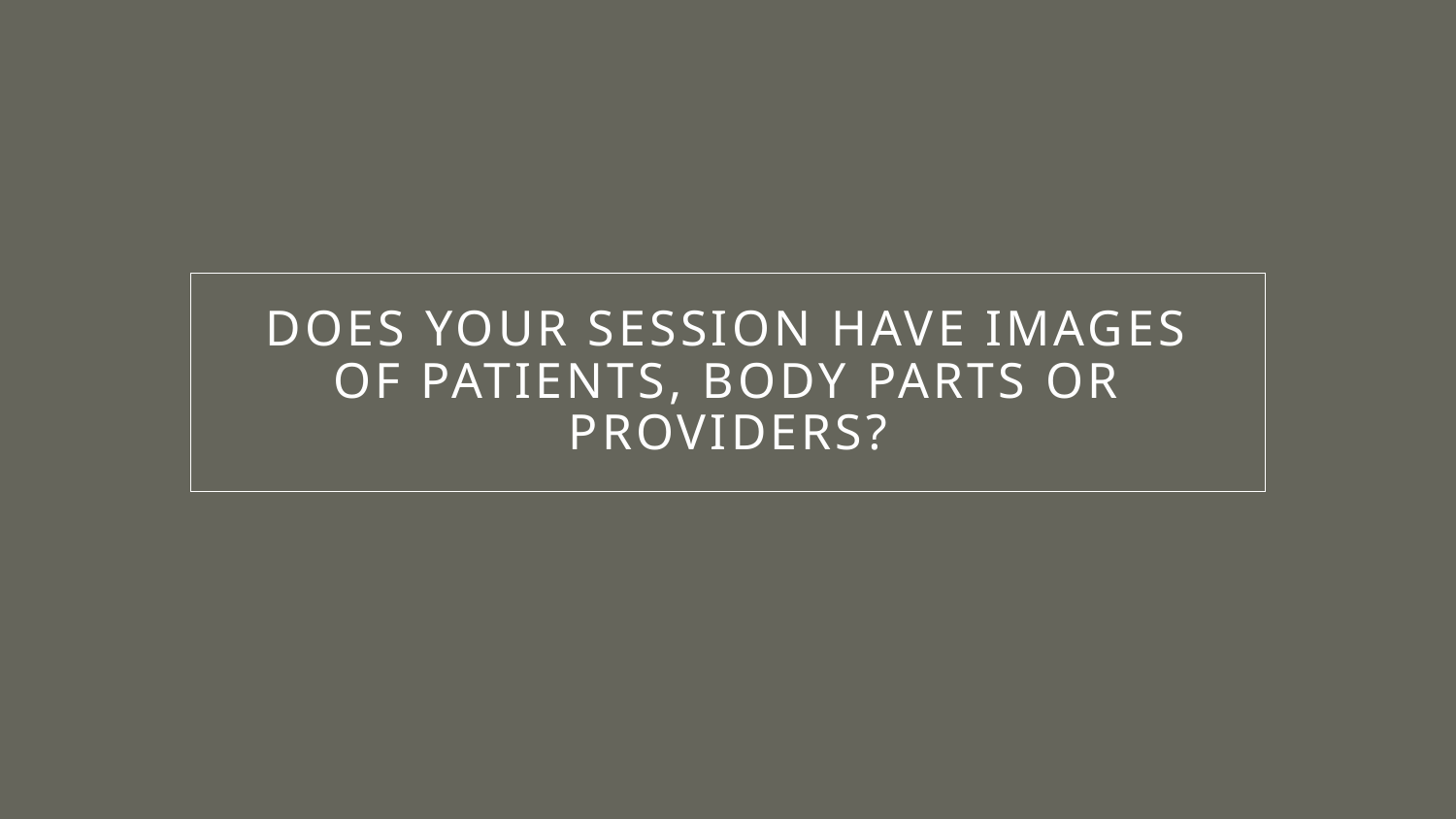

# Does your session have images of patients, body parts or providers?

## Slide 64
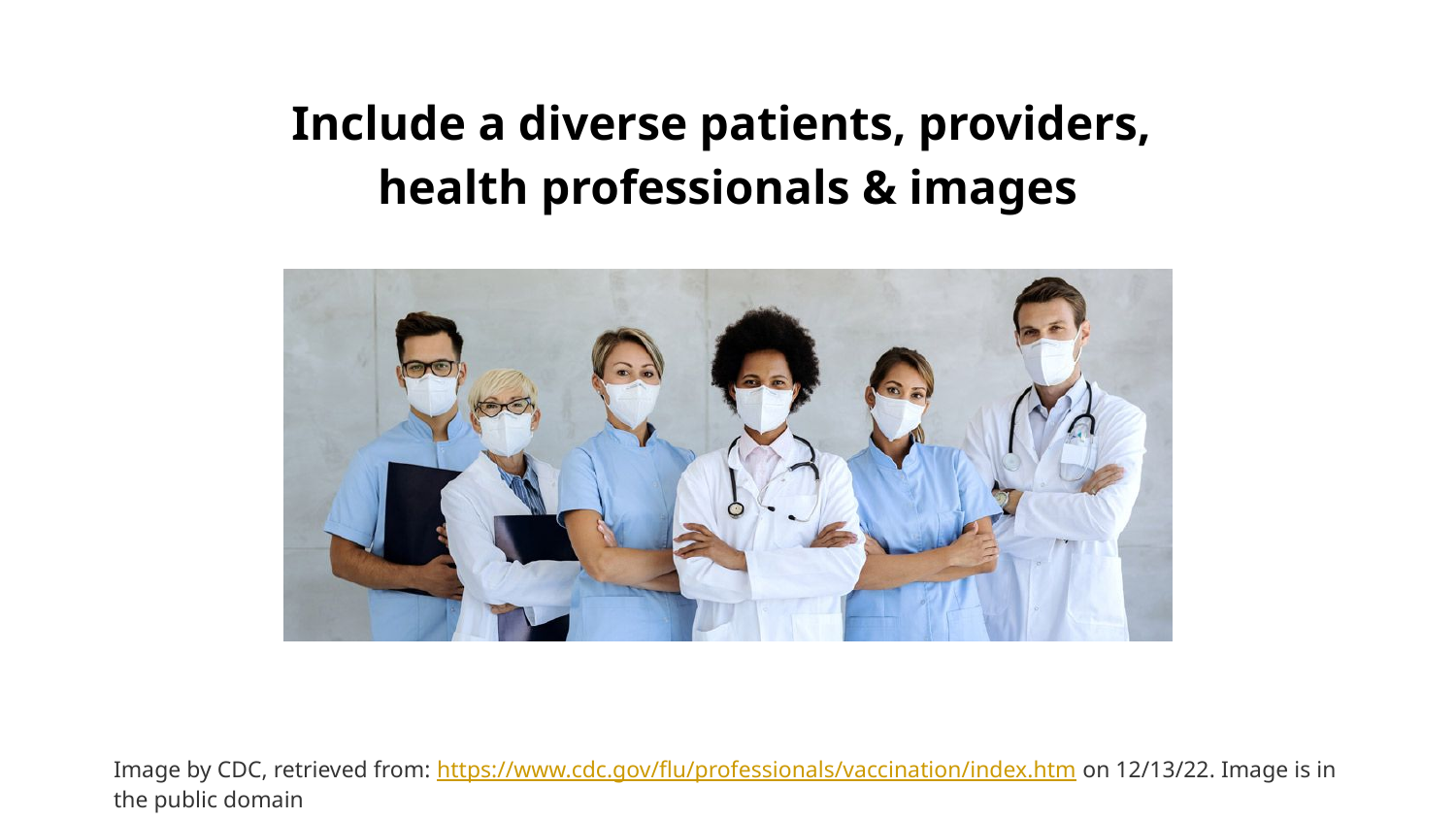

# Include a diverse patients, providers, health professionals & images
Image by CDC, retrieved from: https://www.cdc.gov/flu/professionals/vaccination/index.htm on 12/13/22. Image is in the public domain

## Slide 65
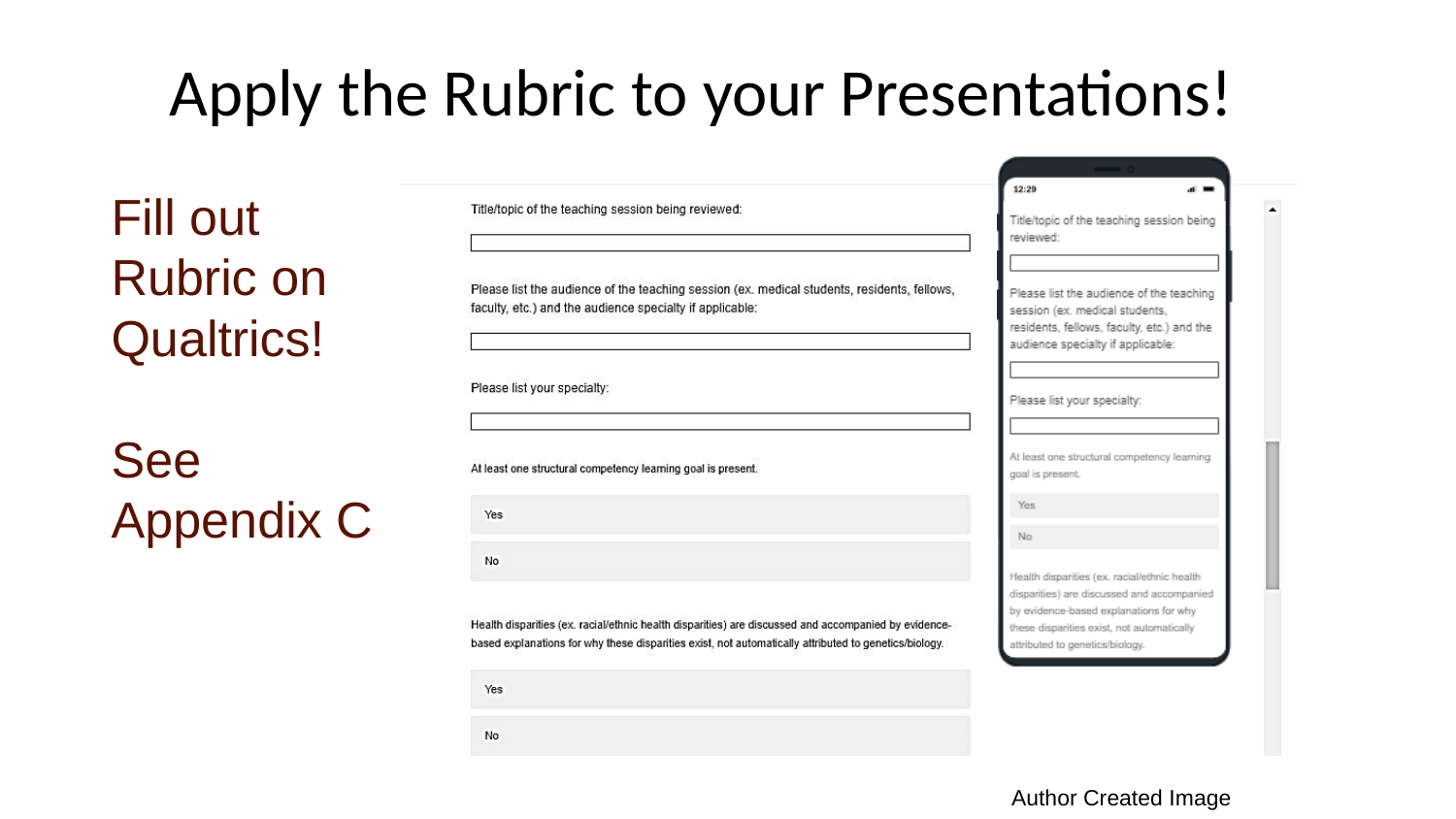

# Apply the Rubric to your Presentations!
Fill out Rubric on Qualtrics!
See Appendix C
Author Created Image

## Slide 66
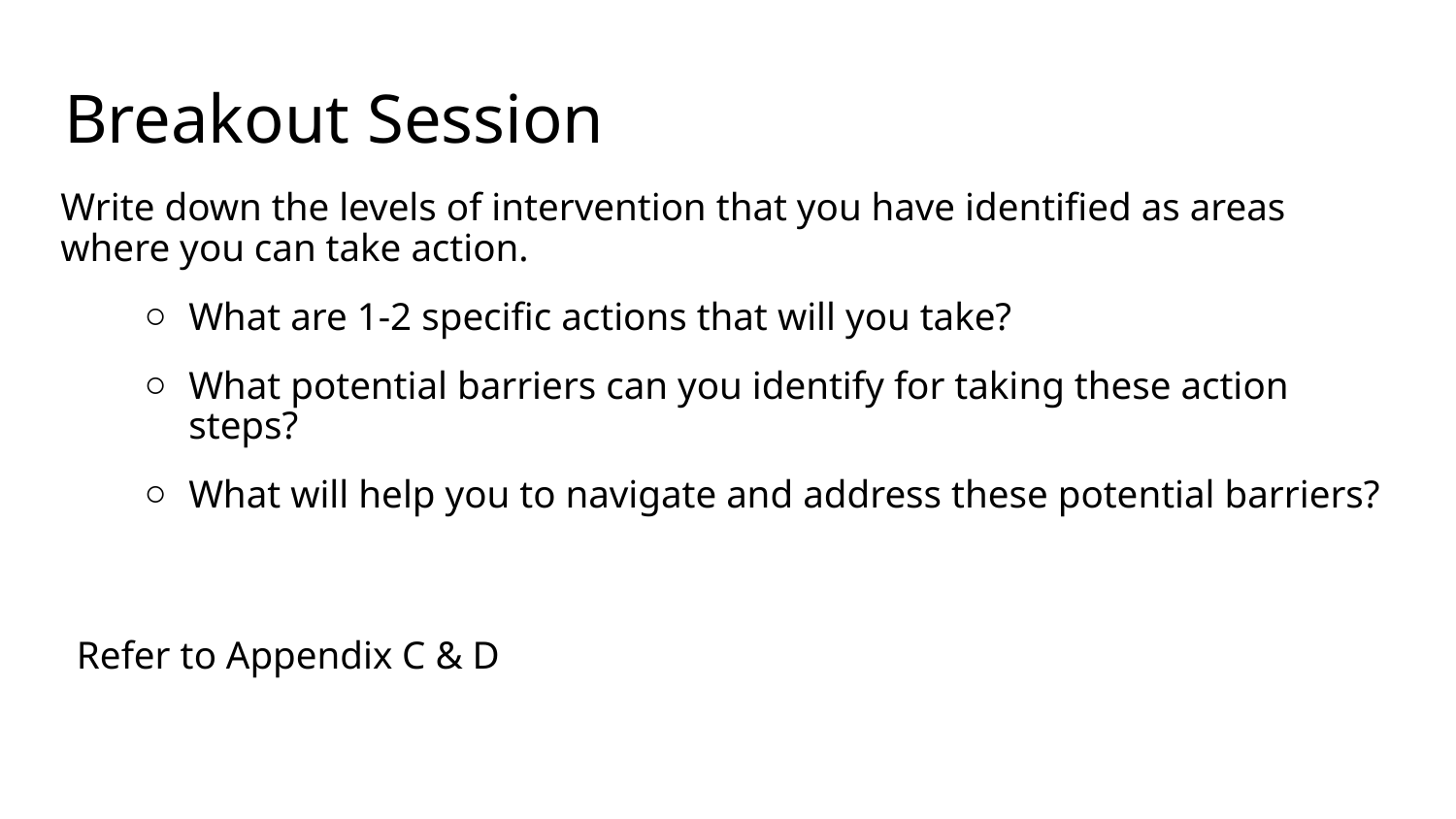

# Breakout Session
Write down the levels of intervention that you have identified as areas where you can take action.
What are 1-2 specific actions that will you take?
What potential barriers can you identify for taking these action steps?
What will help you to navigate and address these potential barriers?
Refer to Appendix C & D

## Slide 67
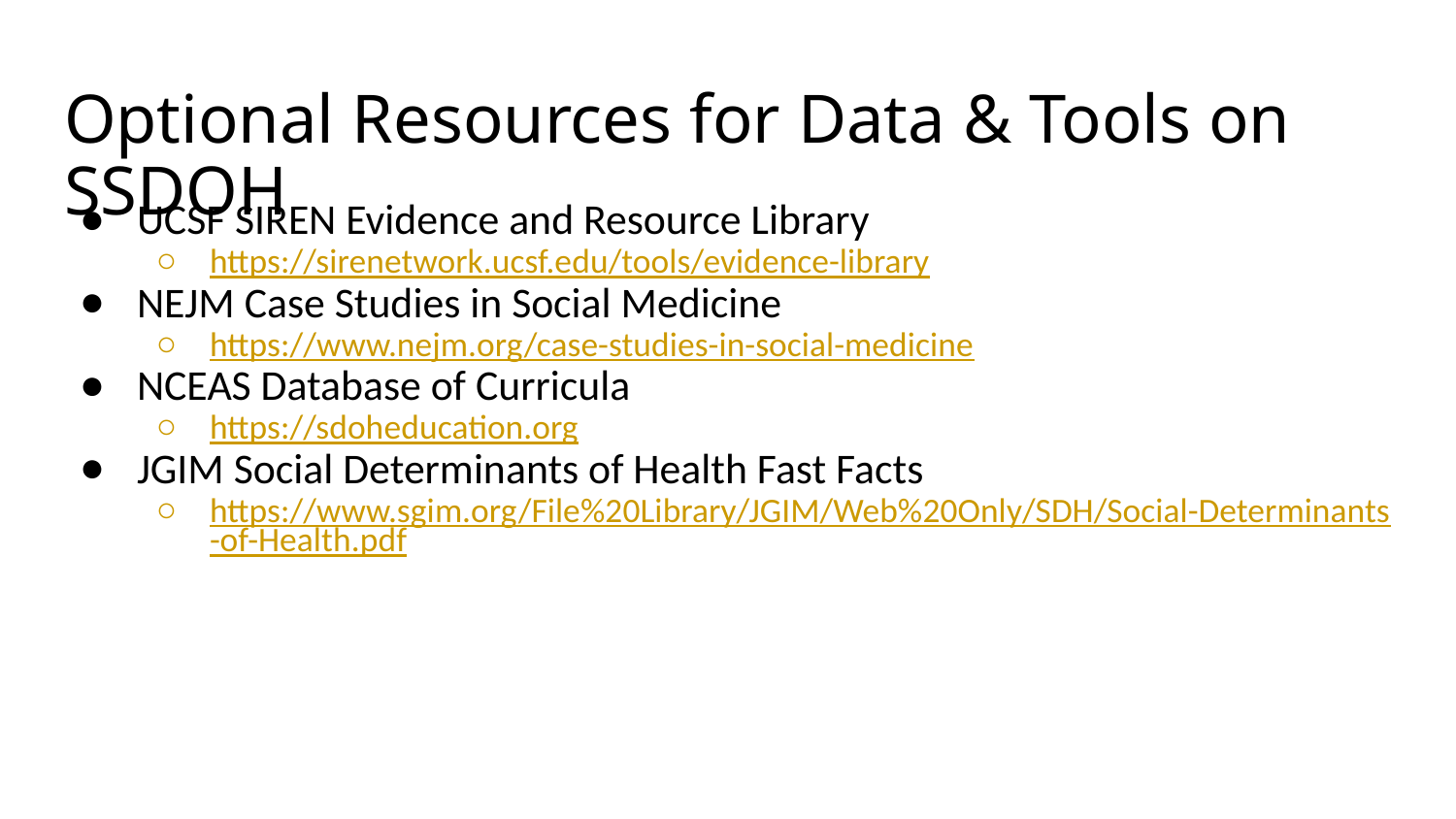

# Optional Resources for Data & Tools on SSDOH
UCSF SIREN Evidence and Resource Library
https://sirenetwork.ucsf.edu/tools/evidence-library
NEJM Case Studies in Social Medicine
https://www.nejm.org/case-studies-in-social-medicine
NCEAS Database of Curricula
https://sdoheducation.org
JGIM Social Determinants of Health Fast Facts
https://www.sgim.org/File%20Library/JGIM/Web%20Only/SDH/Social-Determinants-of-Health.pdf

## Slide 68
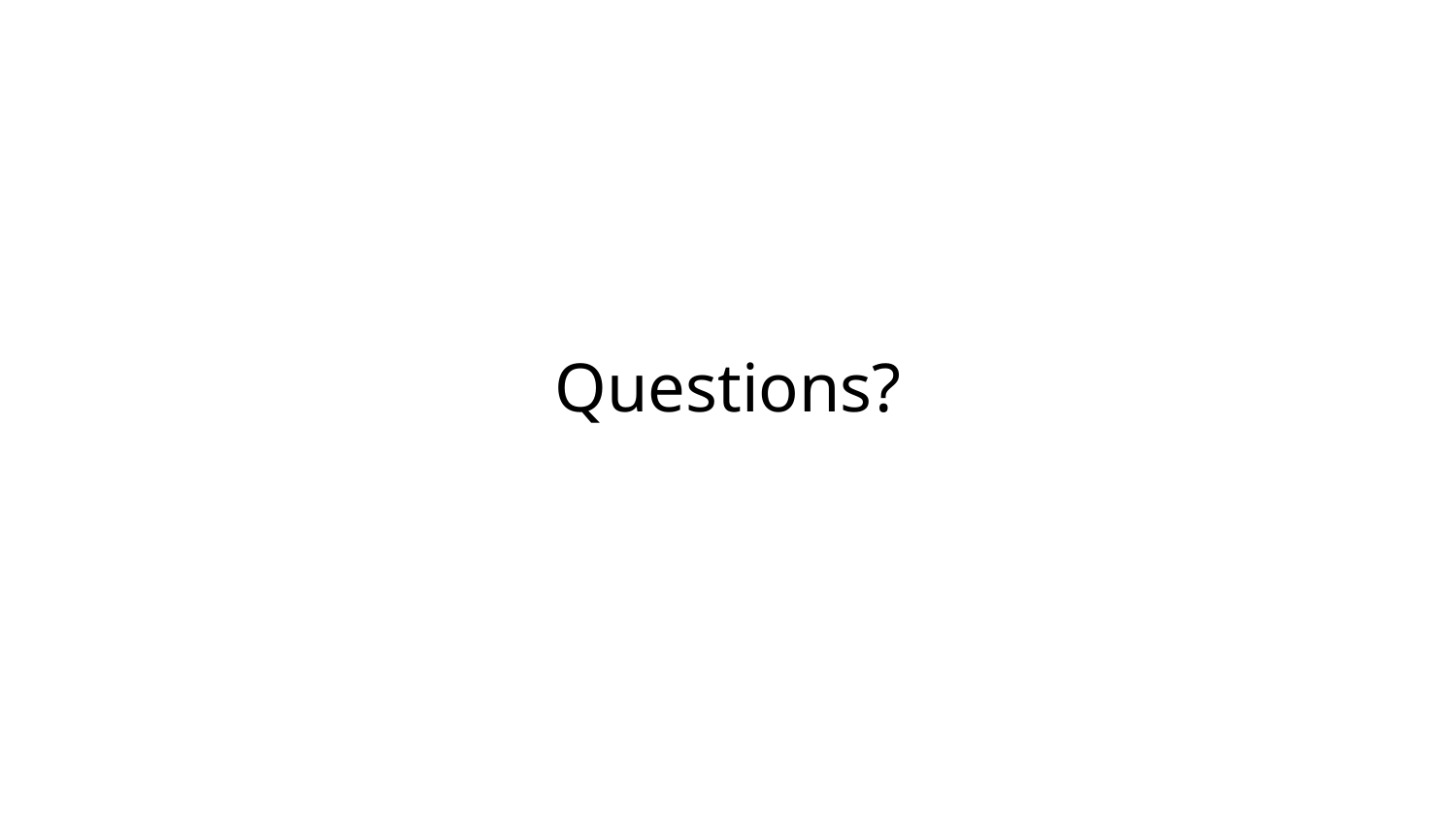

# Questions?
