# Supplementary material for: Structural Competency: A Faculty Development Workshop Series for Anti-racism in Medical Education
Source: MedEdPORTAL. 2025 Feb 7;21:11492. doi: 10.15766/mep_2374-8265.11492 (PMC11802914; doi:10.15766/mep_2374-8265.11492)
Supplement: Supplementary file 1 — 1 - Introduction to SC.pptx1 - Facilitator Guide.docx1 - SC Rubric Handout.docx1 - Sample SC Learning Goals.docx2 - Resident Reports & Case-Based Presentations.pptx2 - Facilitator Guide.docx2 - Structural Differential Handout.docx2 - Small-Group Handout.docx3 - Demystifying SC.pptx3 - Facilitator Guide.docx3 - SC One-Minute Preceptor Handout.docx3 - SC SNAPPS Handout.docx3 - Role-Play Scenarios.docx4 - SC Hospital-Based Teaching.pptx4 - Facilitator Guide.docx4 - Daily Inpatient Checklist.docx4 - SC Discharge Checklist.docx4 - Small-Group Scenarios.docxPre- and Postsurveys.docx [file mep_2374-8265.11492-s001.zip › B. 1 - Facilitator Guide.docx]

**Appendix B – WS 1 SLIDES AND TALKING POINTS**

| **Slide 1**  Welcome Slide: Time Check 00:00 | **Big Picture**: Set a welcoming learning environment. |
| --- | --- |
| 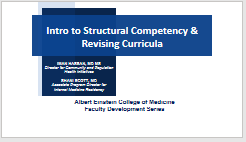 |  |
| *Suggested script/talking points:* Welcome the faculty participants and set a reassuring tone. | |

| **Slide 2**  Learning Objective Overview | **Big Picture:** Brief outline of what will be discussed over the next ninety minutes |
| --- | --- |
| 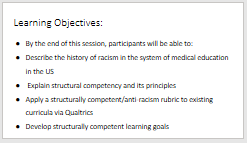 |  |
| *Suggested script/talking points:* Take them through each bullet point briefly. Emphasize that they are going to have specific opportunities to participate and that there will be time for questions at the end. | |

| **Slide 3**  Structural Racism Timeline in Medical Education | **Big Picture:** Brief discussion on what will be discussed over the next ninety minutes |
| --- | --- |
| 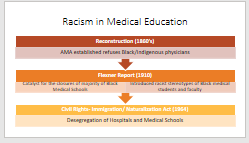 |  |
| *Suggested script/talking points:* Take them through each bullet point briefly. Emphasize that they are going to have specific opportunities to participate and that there will be time for questions at the end. | |

| **Slide 4**  Flexner Report | **Big Picture**: Provide historical context on racist attitudes that shaped policy and regulations for medical education |
| --- | --- |
| 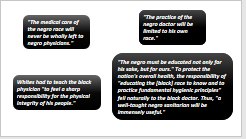 |  |
| *Suggested script/talking points:* Abraham Flexner’s Report created the foundation for our system of medical education  The report did more than describe under-resourced conditions at black medical schools; it explicitly advocated to limited the role for black physicians in their practices and hinted that black physicians possessed less potential and ability than their white counterparts. Read the quotes off the slide. Abraham Flexner continued to introduce language and ideology that devalued Black intellect and capacity. | |
| **Slide 5**  Closure of Black Medical Schools | **Big Picture:** Brief discussion on downstream effects of Flexner Report |
| 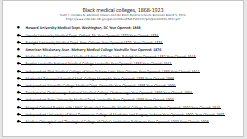 |  |
| *Suggested script/talking points:*  Downstream effects led to the closure of several health profession schools. Meharry and Howard are the only two remained from that era. Now there is the addition of Morehouse School of Medicine and Charles Drew Medical College | |
| **Slide 6**  Evolution to Structural Competency | **Big Picture**: Brief discussion on the conceptual development of structural competency |
| 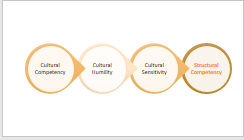 |  |
| *Suggested script/talking points:* Understanding the patient's life context is crucial for building a robust therapeutic relationship and devising effective health management plans. Historically, terms like "cultural competence" have been used to emphasize the importance of integrating a patient's culture into clinical reasoning. However, these terms have evolved over time because they can inadvertently perpetuate bias and harmful stereotypes, potentially pathologizing cultural norms. This evolution reflects a growing awareness of the limitations and potential pitfalls of focusing solely on cultural factors.  Progressing toward structural competency represents an important shift. It empowers health professionals to consider the broader social, economic, and political structures that impact patient health. By understanding these factors, healthcare providers can create more accurate health recommendations and set achievable health outcome goals that align with the patient's unique life context. This approach moves beyond individual cultural considerations, aiming to address systemic issues and promote equity in healthcare. | |
| **Slide 7**  Definition of Structural Competency | **Big Picture:** Introduce the definition of Structural Competency |
| 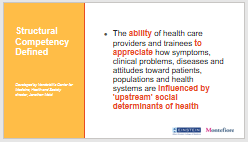 |  |
| *Suggested script/talking points:* Structural competency refers to the trained ability to recognize and respond to health and illness as the downstream effects of broad social, economic, and political structures. Unlike cultural competency, which focuses on individual beliefs and behaviors, structural competency emphasizes understanding how systemic factors—such as policies, institutional practices, and social norms—shape health outcomes. This concept encourages healthcare professionals to consider these larger structural influences when diagnosing, treating, and preventing illness, ultimately aiming to address and reduce health disparities. | |
| **Slide 8**  Definition of Structural Competency | **Big Picture**: Rearticulation of structural competency |
| 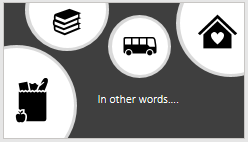 |  |
| *Suggested script/talking points:*  In other words structural competency is the ability of a healthcare professional to understand that a patient’s access to quality housing, education, food, jobs, and transportation are upstream influences on the clinical manifestation of disease within a person and the relationship between the patient, provider, and the health system they reside within. | |
| **Slide 9**  Structural Competency Pillars | **Big Picture:** Brief discussion on structural competency pillars |
| 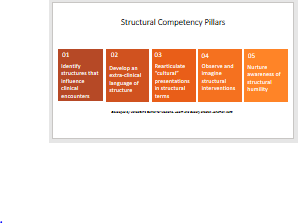 |  |
| *Suggested script/talking points:*  When teaching through the framework of structural competency physicians should evaluate their learners base on these four objectives. | |
| **Slide 10**  Influence of SSDoH on Health Outcomes | **Big Picture:** Structural and social determinants of health (SSDoH) impact health outcomes |
| 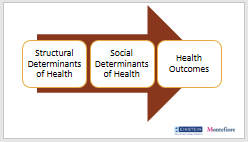 |  |
| *Suggested script/talking points:*  Structural Determinants of Health and the “social and political mechanism[s] that generate, configure and maintain social hierarchies”, for example, racism, labor markets and political institutions. These in turn impact the conditions in which people are *born, grow, work, live, and age,* also known as the Social Determinants of Health. Examples include economic stability, neighborhood and physical environment, education, food, community and social context, and health care system. Social determinants of health contribute to health outcomes and to the health disparities we see across social hierarchies, ex. across races/ethnicities. | |
| **Slide 11**  Define Health Disparity and Health Equity | **Big Picture**: Defining key terminology to enable participants to expand language and meaningful participation in discussions regarding structural competency |
| 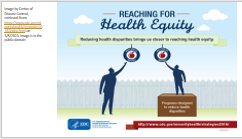 |  |
| *Suggested script/talking points:*  Decoding buzz words is critical to helping learners to understand structural competency and imbed in their clinical reasoning.  Health Disparities: “preventable differences in the burden of disease, injury, violence, or opportunities to achieve optimal health that are experienced by disenfranchised populations” (CDC)  Health Equity: Health equity implies that “everyone should have a fair opportunity to attain their full health potential and that no one should be disadvantaged from achieving this potential” (WHO) | |
| **Slide 12**  Microaggression Definition | **Big Picture**: Defining key terminology to enable participants to expand language and meaningful participation in discussions regarding structural competency |
| 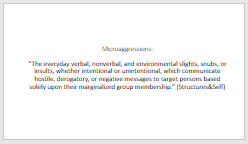 |  |
| *Suggested script/talking points:* Read slide | |
| **Slide 13**  Implicit Bias Definition | **Big Picture**: Defining key terminology to enable participants to expand language and meaningful participation in discussions regarding structural competency |
| 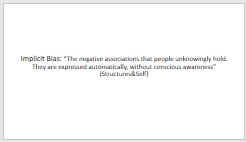 |  |
| *Suggested script/talking points:*  Read slide | |
| **Slide 14**  Systems of Oppression Definition | **Big Picture**: Depicting the key areas in where oppression can be facilitated to enable participants to conceptualize behaviors and institutional rules which lead to the repeated marginalization of patients, communities, and providers. |
| 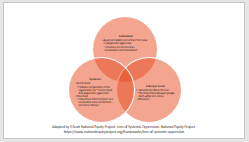 |  |
| *Suggested script/talking points:*  On this slide, we have a Venn diagram illustrating the concept of the 'Lens of Systemic Oppression.' It visually represents how various forms of oppression—such as race, gender, class, sexual orientation, and ability—overlap and intersect within societal structures.  Each circle in the diagram signifies a distinct system of power and inequality. The intersections of these circles highlight how multiple forms of oppression can co-occur, creating unique experiences of discrimination and privilege. For instance, an individual may experience both racial and gender-based discrimination, compounding their daily challenges.  It's important to recognize that these systems are interconnected and can intensify each other's impacts. Oppression, defined as the state of being under unjust treatment or excessive control, is prevalent in medical education and health systems. This presentation introduces the structurally competent rubric, a tool for medical educators to highlight systems of oppression in their teaching. The rubric shifts the focus towards social and structural determinants of health, raising awareness of the potential for pathologizing marginalized identities in patient case presentations. | |
| **Slide 15**  Importance of Structural Competency | **Big Picture**: Explains how structural competency is a vital skill for providers |
| 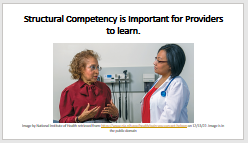 |  |
| *Suggested script/talking points:*  Structural competency is a critical skill to provide effective health services, cultivate a sense of fulfillment in health care professionals, and advocate to create structural change to support healthier patient communities. | |
| **Slide 16**  Quiz | **Big Picture**: Check understanding of structural competency. Spend 3 minutes |
| 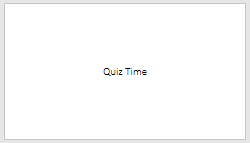 |  |
| *Suggested script/talking points:*  The below quiz was delivered using a Zoom poll:  Which of the following are principles of Structural Competency? (Choose all the apply)   - a. Develop cultural competency in learners - **b. Embrace structural humility** - c. Categorize patients into large groups based on social determinants of health - d. Build interventions to maintain current health structures - e. **Identify “upstream” influences on health** | |
| **Slide 17**  Into for the Structural Competency Rubric  Time Check 20:00 minutes | **Big Picture:** Introduce how to implement the rubric |
| 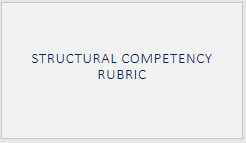 |  |
| *Suggested script/talking points:*  The structural competency rubric as a tool to help guide teaching sessions taught by medical educators. This standardized rubric consists of a structured checklist that can be applied to all didactic sessions, case-based presentations, and other types of teaching sessions on any topic. We will explain in detail the components of the rubric and provide examples of how to apply it. | |
| **Slide 18**  Rubric Overview | **Big Picture**: Provide Reassurance regarding the feasibility of using the rubric |
| 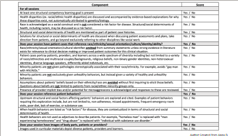 |  |
| *Suggested script/talking points:*  I know this slide looks overwhelming but we will soon show you how straightforward it is  This rubric is a tool to use when creating educational content for your team learners  It can be used in brainstorming sessions to guide the content development process, or after content has been created to aid in making modifications and improvements  This rubric is designed to make teaching actively anti-racist  **This rubric was developed by the authors. Appendix C**  References: Krishnan A, Rabinowitz M, Ziminsky A, Scott SM, Chretien KC. Addressing Race, Culture, and Structural Inequality in Medical Education: A Guide for Revising Teaching Cases. *Acad Med*. 2019;94(4):550-555. doi:10.1097/ACM.0000000000002589 | |
| **Slide 19**  First section of the rubric | **Big Picture**: Introduce the first section of the rubric |
| 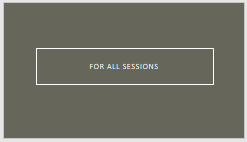 |  |
| *Suggested script/talking points:*  The following items of the rubric apply to all teaching sessions, regardless of content. | |
| **Slide 20**  All session rubric component | **Big Picture**: Structural competency learning goal can be applied to all teaching sessions |
| 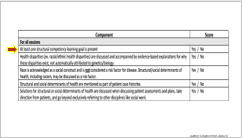 |  |
| *Suggested script/talking points:*  Encourage participants to follow along on pdf  The first item ensures that at least one structural competency learning goal is present in your session | |
| **Slide 21**  Structurally Competent Learning Goals | **Big Picture**: Overview of examples of structural competent learning goals |
| 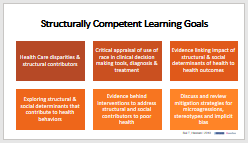 |  |
| *Suggested script/talking points:*  The following are examples of categories of structurally competent learning goals. We will go through each of them and give examples. You can pick one category to help guide the structurally competent learning goal you develop for your teaching session.  [If more examples needed:]   - *Why are Black, Latinx and Native American individuals more likely to be diagnosed with asthma, diabetes, etc.?* - *Critically appraise use of race in eGFR assessments* - *Highlight evidence linking environmental pollutant exposure to asthma* - *In instances of “non-compliance,” inquire about etiology, ex. medication affordability, stressors, etc.* - *Highlight evidence linking housing first programs to improved mental health* - *Acknowledge potential for implicit bias when addressing a patient who previously used injection drugs* | |
| **Slide 22**  Health Disparities learning goal | **Big Picture**: Naming/discussing health disparities as a structurally competent learning goal |
| 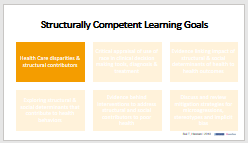 |  |
| *Suggested script/talking points:*  The first category of structurally competent learning goal focuses on naming health disparities and explicitly stating the structural and social contributors to those disparities. | |
| **Slide 23**  Example of Health Disparities as a Learning goal | **Big Picture:** Naming/discussing health disparities as a structurally competent learning goal |
| 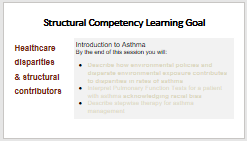 |  |
| *Suggested script/talking points:*  So as an example, if you are teaching a session on asthma, you may choose to include the following structurally competent learning goal that links racial/ethnic health disparities in asthma to the structural contributors of racism in environmental policies and neighborhood pollutant concentration:   - Describe how environmental policies and disparate environmental exposure contributes to disparities in rates of asthma | |
| **Slide 24**  Critical Appraisal of Race in medical literature | **Big Picture:** Critical appraisal of race in medical literature as a structurally competent learning goal |
| 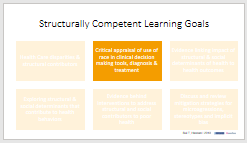 |  |
| *Suggested script/talking points:*  The second type of structurally competent learning goal focuses on the critical appraisal of the use of race in clinical decision-making tools, diagnoses, and treatment.  Race should be accurately defined as a socio-political construct and not rooted in genetics.  Examine if race is being used as an erroneous surrogate for social and structural determinants of health. | |
| **Slide 25**  Example of critically appraising race in the literature | **Big Picture:** Critical appraisal of race in medical literature as a structurally competent learning goal example |
| 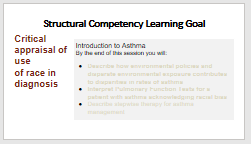 |  |
| *Suggested script/talking points:*  So, for example, in the same session on asthma, a structural competency learning goal that falls in this category might be:   - Interpret pulmonary function tests for a patient with asthma acknowledging racial bias | |
| **Slide 26**  SSDoH impact on health outcomes | **Big Picture**: SSDoH impact on health outcomes as a structurally competent learning goal example |
| 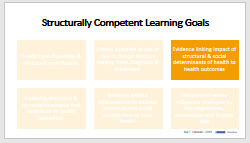 |  |
| *Suggested script/talking points:*  Another category of structurally competent learning goals links evidence of the impact of structural and social determinants of health on health outcomes. | |
| **Slide 27**  Example SSDoH impact on health outcomes | **Big Picture**: SSDoH impact on health outcomes as a structurally competent learning goal example |
| 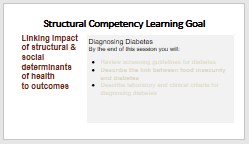 |  |
| *Suggested script/talking points:*  An example of a learning goal that falls under this category, could be:   - Describe the link between food insecurity and diabetes   In a session on diagnosing diabetes.  Here, the social determinant of health is food insecurity and the health outcome is diabetes. | |
| **Slide 28**  Example SSDoH influence on health behaviors | **Big Picture:** SSDoH impact on health behaviors as a structurally competent learning goal example |
| 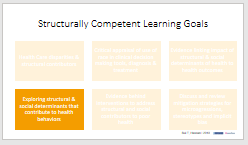 |  |
| *Suggested script/talking points:*  Alternatively, you can choose to explore the link between structural and social determinants of health and health behaviors. | |
| **Slide 29**  Example SSDoH influence on health behaviors | **Big Picture:** SSDoH impact on health behaviors as a structurally competent learning goal example |
| 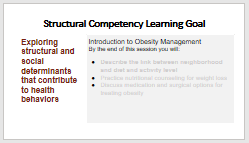 |  |
| *Suggested script/talking points:*  As an example, in this session on Introduction to Obesity Management, an example structurally competent learning goal could be:   - Describe the link between neighborhood and diet and activity level   Where neighborhood is the social determinant of health and diet/activity level is the impacted health behavior. | |
| **Slide 30**  Interventions on SSDoH | **Big Picture:** Interventions on SSDoH as a structurally competent learning goal example |
| 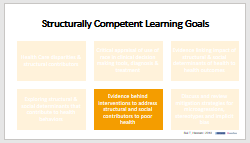 |  |
| *Suggested script/talking points:*  You may choose to focus on evidence behind interventions to address structural and social contributors to poor health as your structurally competent learning goal. | |
| **Slide 31**  Interventions on SSDoH | **Big Picture**: Interventions on SSDoH as a structurally competent learning goal example |
| 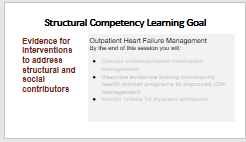 |  |
| *Suggested script/talking points:*  In this session on Outpatient Heart Failure Management, the structurally competent learning goal is:   - Describe evidence linking community health worker programs to improved congestive health failure management | |
| **Slide 32**  Mitigation strategies for microaggressions, stereotypes and implicit bias as a learning goal | **Big Picture**: Discussing/reviewing mitigation strategies for microaggressions, stereotypes and implicit bias as a learning goal |
| 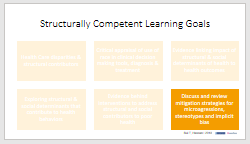 |  |
| *Suggested script/talking points:*  Finally, your structurally competent learning goal could focus on discussing/reviewing mitigation strategies for microaggressions, stereotypes and implicit bias. | |
| **Slide 33**  Example of Discussing/reviewing mitigation strategies for microaggressions, stereotypes and implicit bias | **Big Picture:** Discussing/reviewing mitigation strategies for microaggressions, stereotypes and implicit bias as a learning goal |
| 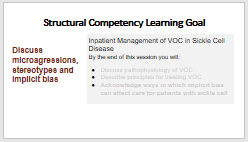 |  |
| *Suggested script/talking points:*  In this session on Inpatient Management of Vaso-Occlusive Crisis in Sickle Cell Disease, a structurally competent learning goal under this category could be:   - Acknowledge the ways in which implicit bias can affect care for patients with sickle cell   Explicitly naming and discussing the ways microagressions, stereotypes and implicit bias manifest is the first step in raising awareness and fostering mitigation | |
| **Slide 34**  The second item of the rubric | **Big Picture**: Highlight health disparities and accompany with evidence-based explanations that are **not** rooted in assumptions about genetics or biology |
| 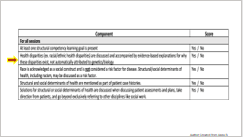 |  |
| *Suggested script/talking points:*  The second item of the rubric asks educators to ensure that health disparities are mentioned in your presentation and accompanied by evidence-based explanations that are not rooted in assumptions about genetics or biology | |
| **Slide 35**  How to Discuss Health Disparities and Contributors | **Big Picture**: Example of highlighting health inequity in a teaching session |
| 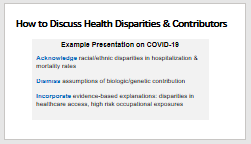 |  |
| *Suggested script/talking points:*  For example, in a presentation on COVID-19, it would be important to acknowledge racial/ethnic disparities, discuss assumptions about biologic/genetic contribution, and incorporate evidence-based explanations like reduced access to healthcare, high occupational exposure, higher incidence of underlying medical conditions from decades of structural racism (in policies/laws/societal practices) | |
| **Slide 36**  Race is a social construct | **Big Picture**: Highlights race as a social construct and not a risk factor for disease. Racism should be identified as a risk factor for disease |
| 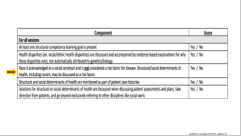 |  |
| *Suggested script/talking points:*  The next item of the rubric that applies to all sessions acknowledges race as a social construct and not a risk factor for disease. Instead racism can be discussed as a risk factor. | |
| **Slide 37**  Acknowledge Race is a social construct | **Big Picture:** Provide example of updated guidelines to reflect race as a social not a biological concept |
| 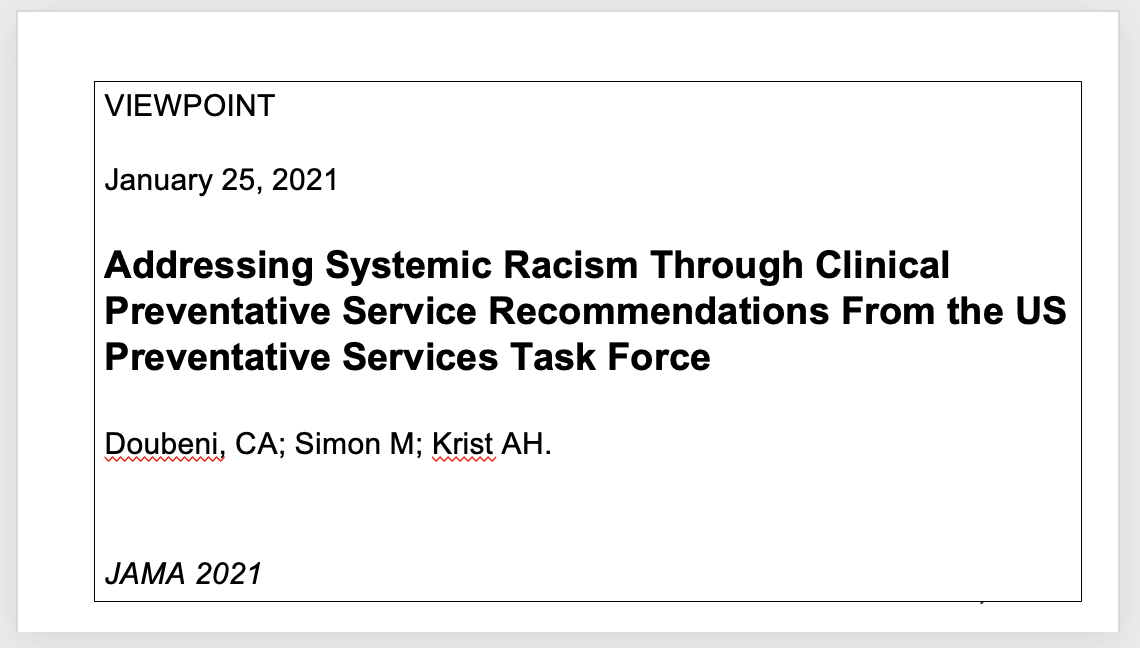 |  |
| *Suggested script/talking points:*  As an example, the USPSTF’s current guidelines for screening for syphilis recommended providers screen high risk populations and includes race/ethnicity as a risk factor. The USPSTF falsely treated race/ethnicity as a biologic/genetic construct and not as the social construct by not acknowledging the structural/social contributors that lead to disparities in rates of infection. In a recent paper in JAMA, the USPSTF committed to treating race/ethnicity as a social construct and not a risk factor for disease. | |
| **Slide 38**  All sessions should highlight structural and social determinants of health | **Big Picture**: Structural and social determinants of health as part of patient case histories. |
| 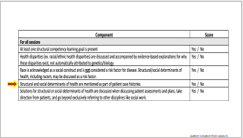 |  |
| *Suggested script/talking points:*  Next, all sessions should mention structural and social determinants of health as part of patient case histories. | |
| **Slide 39**  All sessions should highlight structural and social determinants of health | **Big Picture:** Structural and social determinants of health as part of patient case histories. |
| 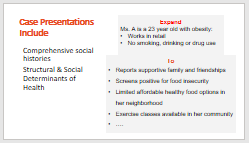 |  |
| *Suggested script/talking points:*   - Structural and social determinants of health should be included in the social history - This can be obtained via a comprehensive social history, screening for social needs or assessing structural vulnerabilities using a structured questionnaire - It is important to include patient’s strengths as well   As an examples, one should expand a “traditional” history of a 23 year old with obesity who works in retail and does not smoke, drink alcohol or use drugs to include:  Strengths like: reports supportive family and friendships, has exercise classes available in her community  Social needs like: screens positive for food insecurity and has limited affordable healthy food options in her neighborhood | |
| **Slide 40**  Solutions for structural or social determinants of health when discussing assessments/plans | **Big Picture**: All sessions should present solutions for structural or social determinants of health when discussing assessments/plans |
| 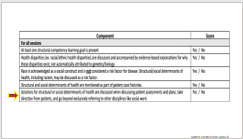 |  |
| *Suggested script/talking points:*   - Finally, all sessions should present solutions for structural or social determinants of health when discussing assessments/plans - Solutions should go beyond exclusively referring to disciplines like social work | |
| **Slide 41**  Discuss solutions for structural and social contributors | **Big Picture:** All sessions should present solutions for structural or social determinants of health when discussing assessments/plans |
| 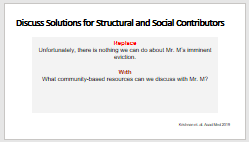 |  |
| *Suggested script/talking points:*   - Solutions take direction from patients - Solutions embed structural humility - Prompt learners to imagine short term and longer term solutions for structural and social determinants of health - Avoid hopelessness/futility   As an examples, in your teaching session, you should replace “unfortunately there is nothing we can do about Mr. M’s imminent eviction” with “what community-based resources can we discuss with Mr. M.” This could prompt discussion about resources such as rent assistance programs, legal services, etc. | |
| **Slide 42**  Quiz | **Big Picture:** Quiz knowledge of what is a structurally competent learning goal. Spend 3 minutes |
| 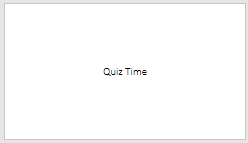 |  |
| *Suggested script/talking points:*  Just to make sure you are still with us….  The following quiz was administered using a Zoom poll:  **Which is the following is a structurally competent learning goal or strategy?**  (a) State the link between obesity and heart disease  **(b) Describe the link between food insecurity and childhood obesity**  (c) Identify race as a risk factor for breast cancer  (d) Not discussing ethnic disparities in preeclampsia | |
| **Slide 43**  Teaching sessions which reference race, ethnicity, sexual orientation, culture, identity, ability or other identifier. Time Check 45:00 | **Big Picture:** Intro to the next section of the rubric |
| 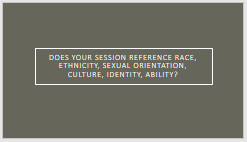 |  |
| *Suggested script/talking points:*  The next section of the structurally competent rubric focuses on those teaching sessions which reference race, ethnicity, sexual orientation, culture, identity, ability or other identifier. If your session does not reference these, you can skip this section of the rubric. If your session does reference an identifier, the following items under this section of the rubric will help ensure your session is anti-racist. | |
| **Slide 44**  How to address race/ethnicity/sexual orientation or other identifier in teaching sessions | **Big Picture:** Omit race/ethnicity/sexual orientation or other identifier from the summary statement. |
| 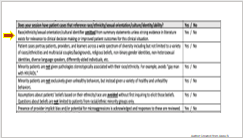 |  |
| *Suggested script/talking points:*  The first item is to omit race/ethnicity/sexual orientation or other identifier from the summary statement. Including identifiers in the summary statement can bias the reader/listener and implies biologic contribution. Identifiers should only be included in the summary statement is very strong evidence exists in the literature regarding relevance to clinical decision making and improved patient outcomes, which is rarely the case. | |
| **Slide 45**  How to address race/ethnicity/sexual orientation or other identifier in teaching sessions | **Big Picture:** Omit race/ethnicity/sexual orientation or other identifier from the summary statement. |
| 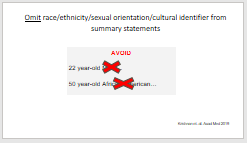 |  |
| *Suggested script/talking points:*  So for example, in your teaching session, you should avoid 22-year-old male who has sex with men or 50-year-old African American. | |
| **Slide 46**  How to address race/ethnicity/sexual orientation or other identifier in teaching sessions | **Big Picture**: Portray a wide diversity of representation of patients and providers |
| 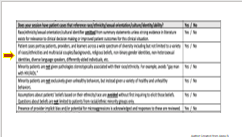 |  |
| *Suggested script/talking points:*  The second item ensures that patient cases portray patients, providers and learners across a wide spectrum of diversity (including race/ethnicity, religion, gender identity, sexuality, language, ability). This ensures representation in cases and scenarios and normalizes differences. | |
| **Slide 47**  Portray a wide diversity of patients and providers | **Big Picture**: Example on how to portray a wide diversity of representation of patients and providers |
| 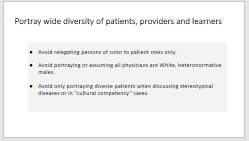 |  |
| *Suggested script/talking points:*   - For example, avoiding relegating persons of color to patient roles only, have providers also be of color - Avoid assuming or displaying provides as White, heteronormative or male - Avoid only portraying diverse patients when discussing stereotypical diseases, for ex. HIV positivity in male who has sex with men | |
| **Slide 48**  Addressing stereotypes of minoritized patients | **Big Picture**: Correct didactics that stereotypically depict minoritized patients with pathologies associated with their race/ethnicity or other identifier |
| 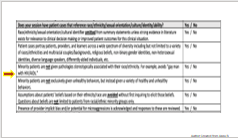 |  |
| *Suggested script/talking points:*  The next item explicitly states that minoritized patients should not be given pathologies associated with their race/ethnicity or other identifier. This helps prevent the perpetuation of stereotypes. | |
| **Slide 49**  Removing stereotypes related to pathology in minoritized patients | **Big Picture**: Correct didactics that stereotypically depict minoritized patients with pathologies associated with their race/ethnicity or other identifier |
| 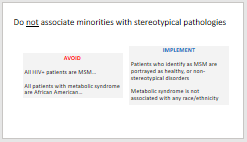 |  |
| *Suggested script/talking points:*  Instead of portraying all HIV+ patients as men who have sex with men (MSM), instead patients who identify as MSM should be portrayed as healthy or having non-stereotypical disorders.  Instead of portraying all patients with metabolic syndrome as African American, metabolic syndrome should not be associated with a race/ethnicity. | |
| **Slide 50**  Removing stereotypes relating to health behaviors | **Big Picture**: Correct didactics that stereotypically depict minoritized patients with pathologies associated with their race/ethnicity or other identifier |
| 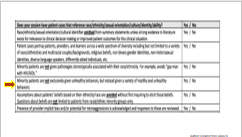 |  |
| *Suggested script/talking points:*  Next, minoritized patients should not be given only unhealthy behaviors. Instead, they should be given a mix of healthy and unhealthy behaviors. | |
| **Slide 51**  Removing stereotypes relating to health behaviors | **Big Picture**: Example of avoiding associating high risk health behaviors with minoritized patients |
| 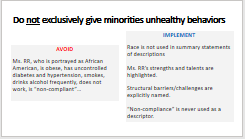 |  |
| *Suggested script/talking points:*  For example, instead of portraying Ms. RR as having exclusively unhealthy behaviors, she should also have strengths and talents. Moreover, race should not be included in the summary statement and structural/social barriers should be explicitly named. Non-compliance should not be used, and instead underlying reasons for medication non-adherence (the structural/social barriers) should be discussed. | |
| **Slide 52**  Assumptions about patient’s beliefs based on their race/ethnicity should be avoided without first inquiring about and eliciting those beliefs | **Big Picture**: Audit teaching material to remove assumptions about patient’s beliefs based on their race/ethnicity |
| 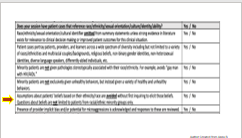 |  |
| *Suggested script/talking points:*  When using an identifier, assumptions about patient’s beliefs based on their race/ethnicity should be avoided without first inquiring about and eliciting those beliefs. Certain beliefs should not be limited to patients from a specific racial/ethnic group. | |
| **Slide 53**  Remove assumptions regarding patient beliefs | **Big Picture**: Example of how to remove assumptions about patient’s beliefs based on their race/ethnicity |
| 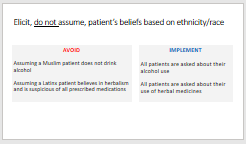 |  |
| *Suggested script/talking points:*  As an example, assumptions should not be made that all Muslim patients do not drink alcohol or that all Latinx patients believe in herbalism. Instead, all patients should be asked about their alcohol use and use of herbal medications. This item of the rubric emphasizes the need to avoid making assumptions and generalities about persons based on their racial/ethnic or other identifier and avoids perpetuating stereotypes. | |
| **Slide 54**  Acknowledge provider potential for implicit bias and microaggressions and mitigation strategies | **Big Picture:** Audit teaching material to acknowledge provider potential for implicit bias and microaggressions and mitigation strategies |
| 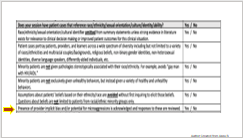 |  |
| *Suggested script/talking points:*  The final item of this section asks educators to acknowledge provider potential for implicit bias and microaggressions and mitigation strategies. | |
| **Slide 55**  Acknowledge provider potential for implicit bias and microaggressions and mitigation strategies | **Big Picture**: Example of how to acknowledge provider potential for implicit bias and microaggressions and mitigation strategies in teaching material. |
| 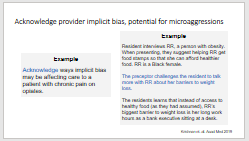 |  |
| *Suggested script/talking points:*  As an examples, a teaching session can acknowledge the ways implicit bias may affect care of a patient with chronic pain on opiates, or highlight the need to explore patient’s experiences, values and beliefs before making assumptions that can lead to microaggressions. | |
| **Slide 56**  Audit session for patient behaviors | **Big Picture**: This section of the structurally competent rubric applies to teaching session that explicitly reference patient behaviors. |
| 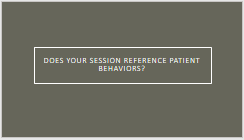 |  |
| *Suggested script/talking points:*  The next section of the structurally competent rubric applies to teaching session that explicitly reference patient behaviors. If your session does not reference patient behaviors, you may skip this section of the rubric. | |
| **Slide 57**  Structural and social determinants of health affecting patient behaviors | **Big Picture:** Audit material to ensure structural and social determinants of health affecting patient behaviors. |
| 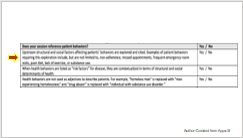 |  |
| *Suggested script/talking points:*  The first item ensures that upstream structural and social determinants of health affecting patient behaviors are explicitly discussed. | |
| **Slide 58**  Structural and social determinants of health affecting patient behaviors | **Big Picture**: Example of structural and social determinants of health affecting patient behaviors |
| 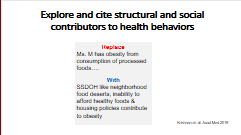 |  |
| *Suggested script/talking points:*  As an example, avoiding Ms. M has obesity from consumption of processed food (a behavior) without mentioning structural and social determinants of health (SSDoH). Instead, SSDoH like neighborhood food deserts, inability to afford healthy foods and housing policies should be explicitly mentioned as contributors to diet. | |
| **Slide 59**  Health behaviors in the context of structural and social determinants of health | **Big Picture:** Contextualize health behaviors in terms of structural and social determinants of health. |
| 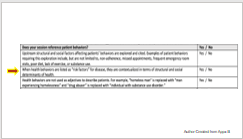 |  |
| *Suggested script/talking points:*  Next, when health behaviors are listed as risk factors for disease, they should be contextualized in terms of structural and social determinants of health. | |
| **Slide 60**  Health behaviors in the context of structural and social determinants of health | **Big Picture**: Example of how to contextualize health behaviors in the setting of structural and social determinants of health |
| 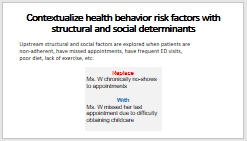 |  |
| *Suggested script/talking points:*  Risk factors like “non-adherence,” missed appointments, frequent emergency department visits, unhealthy diet, lack of exercise should not be mentioned without mentioning underlying structural and social contributors. As an example, Ms. W chronically no-shows to appointments should be replaced with Ms. W missed her last appointment due to difficulty obtaining childcare. | |
| **Slide 61**  Addressing health behaviors being used as adjectives for patients | **Big Picture:** Audits teaching sessions for health behaviors being used as adjectives for patients. |
| 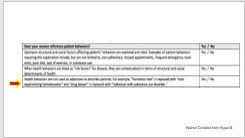 |  |
| *Suggested script/talking points:*  Finally, health behaviors should not be used as adjectives to describe patients. | |
| **Slide 62**  Addressing health behaviors being used as adjectives for patients | **Big Picture**: Example of how to address health behaviors being used as adjectives for patients. |
| 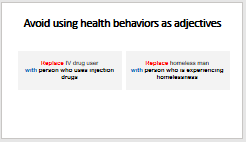 |  |
| *Suggested script/talking points:*  As an example, instead of saying 'IV drug user,' I would say 'person who uses injection drugs.' Instead of 'homeless man,' I would say 'person who is experiencing homelessness. | |
| **Slide 63**  Imaging of patients, body parts or providers | **Big Picture**: Auditing teaching session images of patients, body parts, and providers. |
| 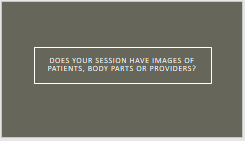 |  |
| ***Suggested script/talking points:***  **The last section of the rubric applies to sessions that have images of patients, providers or body parts.** | |
| **Slide 64**  Imaging of patients, body parts or providers | **Big Picture:** Example of diverse images of patients, body parts, and providers. |
| 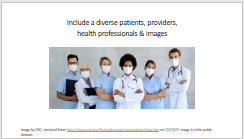 |  |
| *Suggested script/talking points:*  This section of the rubric has only one item, and that is that images should represent a diverse range of patients, providers and skin tones, body habitus, and ability. | |
| **Slide 65**  Qualtrics link to rubric | **Big Picture**: Link to the rubric on Qualtrics for easy use. Or use Appx C |
| 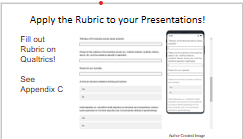 |  |
| *Suggested script/talking points:*   - Link for rubric: <https://einsteinmed.co1.qualtrics.com/jfe/form/SV_1U3maeK8CwM3kqi> | |
| **Slide 66**  Break Out Session- Time Check 65:00 min | **Big Picture**: Opportunity for participants to apply rubric to a teaching case presentation. Spend 20 minutes |
| 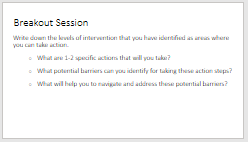 |  |
| *Suggested script/talking points:*   - Clear directions on how to pull up structurally competent rubric on Qualtrics - Small groups of about five individuals in each group - Each group looks at a didactic presentation using the rubric and discusses where improvements can be made   Take Home Points:   - Add a structurally competent learning goal - Avoid attributing racial/ethnic disparities to genetics/biology - Look to structural & social determinants of health to explain health disparities and patient behavior - Treatment plans should discuss structural and social contributors - Avoid racial/cultural stereotypes - Discuss implicit bias & microagressions - Diversify patients, providers and health professional roles   Reference Appendix C and D | |
| **Slide 67**  Resources to support structural competence | **Big Picture**: Provide online resources to additional teaching material that support educators in building structural competency in learners. |
| 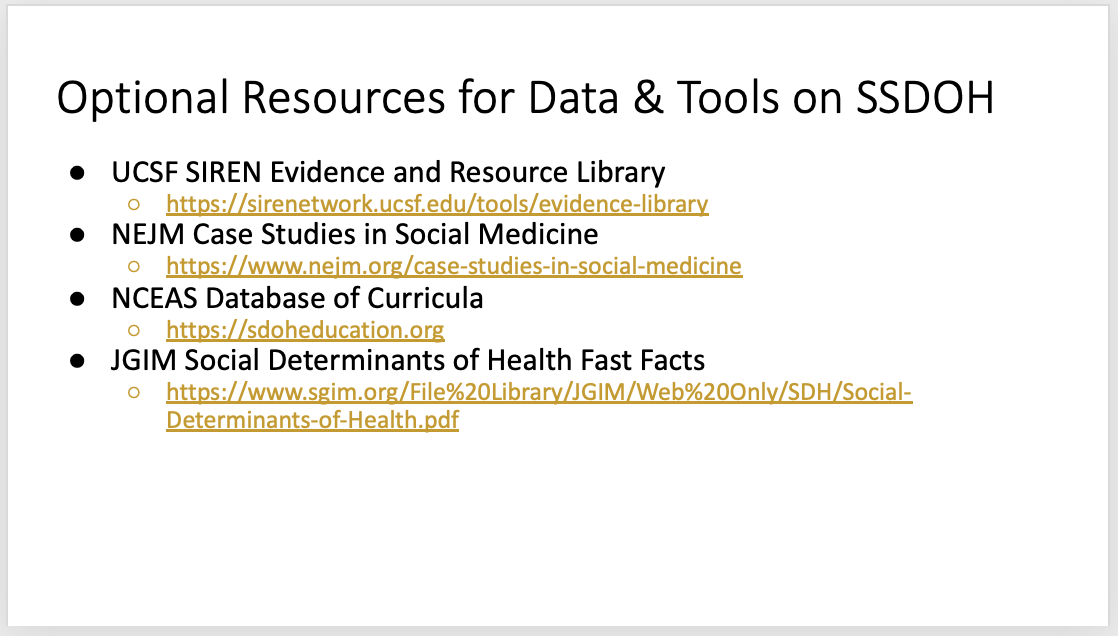 |  |
| *Suggested script/talking points:* These are optional resource links. Take them through each bullet point briefly. | |
| **Slide 68**  Questions: Time Check 85:00 | **Big Picture**: Large group discussion for questions regarding the rubric or overall presentation . Spend Five minutes or more as time allows |
| 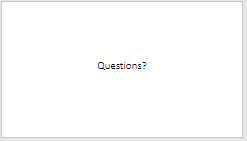 |  |
| *Suggested script/talking points:*  Take questions from participants regarding the presentation or structural competency rubric | |
