# Supplementary material for: Structural Competency: A Faculty Development Workshop Series for Anti-racism in Medical Education
Source: MedEdPORTAL. 2025 Feb 7;21:11492. doi: 10.15766/mep_2374-8265.11492 (PMC11802914; doi:10.15766/mep_2374-8265.11492)
Supplement: Supplementary file 1 — 1 - Introduction to SC.pptx1 - Facilitator Guide.docx1 - SC Rubric Handout.docx1 - Sample SC Learning Goals.docx2 - Resident Reports & Case-Based Presentations.pptx2 - Facilitator Guide.docx2 - Structural Differential Handout.docx2 - Small-Group Handout.docx3 - Demystifying SC.pptx3 - Facilitator Guide.docx3 - SC One-Minute Preceptor Handout.docx3 - SC SNAPPS Handout.docx3 - Role-Play Scenarios.docx4 - SC Hospital-Based Teaching.pptx4 - Facilitator Guide.docx4 - Daily Inpatient Checklist.docx4 - SC Discharge Checklist.docx4 - Small-Group Scenarios.docxPre- and Postsurveys.docx [file mep_2374-8265.11492-s001.zip › C. 1 - SC Rubric Handout.docx]

| **Component** | **Score** |
| --- | --- |
| **For all sessions** | |
| At least one structural competency learning goal is present | Yes / No |
| Health disparities (ex. racial/ethnic health disparities) are discussed and accompanied by evidence-based explanations for why these disparities exist, not automatically attributed to genetics/biology. | Yes / No |
| Race is acknowledged as a social construct and is **not** considered a risk factor for disease. Structural/social determinants of  health, including racism, may be discussed as a risk factor. | Yes / No |
| Structural and social determinants of health are mentioned as part of patient case histories. | Yes / No |
| Solutions for structural or social contributors are discussed when discussing patient assessments and plans, take direction from patients, and go beyond exclusively referring to other disciplines like social work. | Yes / No |
| **Does your session have patient cases that reference race/ethnicity/sexual orientation/culture/identity/ability?** | **Yes / No** |
| Race/ethnicity/sexual orientation/cultural identifier is **omitted** from summary statements unless strong evidence in literature exists for relevance to clinical decision making or improved patient outcomes for this clinical situation. | Yes / No |
| Patient cases portray patients, providers, and learners across a wide spectrum of diversity including but not limited to a variety of races/ethnicities and multiracial couples/backgrounds, religious beliefs, non-binary gender identities, non-heterosexual  identities, diverse language speakers, differently-abled individuals, etc. | Yes / No |
| Minority patients are **not** given pathologies stereotypically associated with their race/ethnicity. For example, avoids “gay man with HIV/AIDs.” | Yes / No |
| Minority patients are **not** exclusively given unhealthy behaviors, but instead given a variety of healthy and unhealthy  behaviors. | Yes / No |
| Assumptions about patients’ beliefs based on their ethnicity/race are **avoided** without first inquiring to elicit those beliefs. Questions about beliefs are **not** limited to patients from racial/ethnic minority groups only. | Yes / No |
| Presence of provider implicit bias and/or potential for microaggressions is acknowledged and responses to these are reviewed. | Yes / No |
| **Does your session reference patient behaviors?** | **Yes / No** |
| Upstream structural and social factors affecting patients’ behaviors are explored and cited. Examples of patient behaviors requiring this exploration include, but are not limited to, non-adherence, missed appointments, frequent emergency room visits, poor diet, lack of exercise, or substance use. | Yes / No |
| When health behaviors are listed as “risk factors” for disease, they are contextualized in terms of structural and social  determinants of health. | Yes / No |
| Health behaviors are not used as adjectives to describe patients. For example, “homeless man” is replaced with “man experiencing homelessness” and “drug abuser” is replaced with “individual with substance use disorder.” | Yes / No |
| **Does your session have images of body parts, patients or providers?** | **Yes / No** |
| Images used in curricular materials depict diverse patients, providers and learners. | Yes / No |

DEFINITIONS:

**Structural competency:** “A shift in medical education toward attention to forces that influence health outcomes at levels above individual interactions” and the capacity for health professionals to recognize and respond to health and illness as the downstream effects of broad social, political, and economic structures (Metzl and Hansen).

**Structural determinants of health:** The structural mechanisms that “generate stratification and social class divisions in the society and that define individual socioeconomic position within hierarchies of power, prestige an access to resources” the most important being race/ethnicity, income, education, occupation, social class and gender; and the resultant socioeconomic position of individuals (WHO).

**Social determinants of health:** The “conditions in which people are born, grow, live, work and age” that “are shaped by the distribution of money, power and resources at global, national and local levels” (WHO).

**Implicit bias:** “The attitudes or stereotypes that affect our understanding, actions, and decisions in an unconscious manner” (Kirwan Institute).

**Microaggressions:** “The everyday verbal, nonverbal, and environmental slights, snubs, or insults, whether intentional or unintentional, which communicate hostile, derogatory, or negative messages to target persons based solely upon their marginalized group membership” (Structures&Self).

**Upstream:** The macro factors that comprise social and structural influences on health and health systems, government policies, and the social, physical, economic and environmental factors that determine health (RAND).
