# Supplementary material for: Structural Competency: A Faculty Development Workshop Series for Anti-racism in Medical Education
Source: MedEdPORTAL. 2025 Feb 7;21:11492. doi: 10.15766/mep_2374-8265.11492 (PMC11802914; doi:10.15766/mep_2374-8265.11492)
Supplement: Supplementary file 1 — 1 - Introduction to SC.pptx1 - Facilitator Guide.docx1 - SC Rubric Handout.docx1 - Sample SC Learning Goals.docx2 - Resident Reports & Case-Based Presentations.pptx2 - Facilitator Guide.docx2 - Structural Differential Handout.docx2 - Small-Group Handout.docx3 - Demystifying SC.pptx3 - Facilitator Guide.docx3 - SC One-Minute Preceptor Handout.docx3 - SC SNAPPS Handout.docx3 - Role-Play Scenarios.docx4 - SC Hospital-Based Teaching.pptx4 - Facilitator Guide.docx4 - Daily Inpatient Checklist.docx4 - SC Discharge Checklist.docx4 - Small-Group Scenarios.docxPre- and Postsurveys.docx [file mep_2374-8265.11492-s001.zip › D. 1 - Sample SC Learning Goals.docx]

| **Learning Goal/Objective Topic** | **Examples** |
| --- | --- |
| Healthcare disparities & structural contributors | *Why are Black, Latinx and Native American individuals more likely to be diagnosed with asthma, diabetes, etc.?* |
| Critical appraisal of use of race in clinical decision making tools, diagnosis and treatment | *Critically appraise use of race in eGFR assessments* |
| Evidence linking impact of structural & social determinants of health to health outcomes | *Highlight evidence linking environmental pollutant exposure to asthma* |
| Exploring structural & social determinants of health that contribute to health behaviors | *In instances of “non-compliance,” inquire about etiology, ex. medication affordability, stressors, etc.* |
| Evidence behind interventions to address structural and social contributors to poor health | *Highlight evidence linking Housing First programs to improved mental health* |
| Discuss and review mitigation strategies for microagressions, stereotypes and implicit bias | *Acknowledge potential for implicit bias when addressing a patient who previously used injection drugs* |

Compiled by: Hassan, I & Scott, S ⏐ Montefiore-Einstein **Appendix D: Accompanies Appendix A WS1 slide 66**

Ref: Krishnan et. al. Acad Med. 2019; Bui T et. al. 2019
