# Supplementary material for: Structural Competency: A Faculty Development Workshop Series for Anti-racism in Medical Education
Source: MedEdPORTAL. 2025 Feb 7;21:11492. doi: 10.15766/mep_2374-8265.11492 (PMC11802914; doi:10.15766/mep_2374-8265.11492)
Supplement: Supplementary file 1 — 1 - Introduction to SC.pptx1 - Facilitator Guide.docx1 - SC Rubric Handout.docx1 - Sample SC Learning Goals.docx2 - Resident Reports & Case-Based Presentations.pptx2 - Facilitator Guide.docx2 - Structural Differential Handout.docx2 - Small-Group Handout.docx3 - Demystifying SC.pptx3 - Facilitator Guide.docx3 - SC One-Minute Preceptor Handout.docx3 - SC SNAPPS Handout.docx3 - Role-Play Scenarios.docx4 - SC Hospital-Based Teaching.pptx4 - Facilitator Guide.docx4 - Daily Inpatient Checklist.docx4 - SC Discharge Checklist.docx4 - Small-Group Scenarios.docxPre- and Postsurveys.docx [file mep_2374-8265.11492-s001.zip › P. 4 - Daily Inpatient Checklist.docx]

|  | DAILY INPATIENT CHECKLIST |
| --- | --- |
| 🞎 | **Prompt the team to elicit social histories & assess social needs** |
| 🞎 | **Utilize a structural differential** |
| 🞎 | **Ensure communication with interprofessional team including** |
| 🞎 | **outpatient providers** |
| 🞎 | **Review documentation for bias and inclusion of structural/social** |
| 🞎 | **contributors** |
| 🞎 | **Incorporate structurally competent teaching topics** |
| 🞎 | **Stimulate structurally competent self-directed learning** |

Hassan I & Scott S | Albert Einstein College of Medicine 2021

**Adapted by**: Hassan I. & Scott S. **References**: Soong C, Daub S, Lee J, Majewski C, Musing E, Nord P, Wyman R et. al. Developing of a checklist of safe discharge practices for hospital patients. *Journal of Hospital Medicine*. 2013;8:444-419**. DISTRIBUTE WITH APPENDIX N**
