# Supplementary material for: Structural Competency: A Faculty Development Workshop Series for Anti-racism in Medical Education
Source: MedEdPORTAL. 2025 Feb 7;21:11492. doi: 10.15766/mep_2374-8265.11492 (PMC11802914; doi:10.15766/mep_2374-8265.11492)
Supplement: Supplementary file 1 — 1 - Introduction to SC.pptx1 - Facilitator Guide.docx1 - SC Rubric Handout.docx1 - Sample SC Learning Goals.docx2 - Resident Reports & Case-Based Presentations.pptx2 - Facilitator Guide.docx2 - Structural Differential Handout.docx2 - Small-Group Handout.docx3 - Demystifying SC.pptx3 - Facilitator Guide.docx3 - SC One-Minute Preceptor Handout.docx3 - SC SNAPPS Handout.docx3 - Role-Play Scenarios.docx4 - SC Hospital-Based Teaching.pptx4 - Facilitator Guide.docx4 - Daily Inpatient Checklist.docx4 - SC Discharge Checklist.docx4 - Small-Group Scenarios.docxPre- and Postsurveys.docx [file mep_2374-8265.11492-s001.zip › Q. 4 - SC Discharge Checklist.docx]

| **Checklist of Structurally Competent Discharge Practices for Hospital Patients** | **Day of Admission** | **Subsequent Hospital Days** | **Discharge Day** |
| --- | --- | --- | --- |
| 1. **Identify & address social needs that impact safe discharge**    1. **Assess for social needs.**    2. **Document social needs in assessments and plans for admissions, progress notes & discharges.**    3. **Work with patient and interdisciplinary team to develop sustainable solutions.***    4. **Connect patients to clinic & community resources for addressing social needs.** | **√**  **√**  **√** | **√**  **√**  **√**  **√** | **√**  **√**  **√** |
| 1. **Ensure primary care continuity and follow-up**    1. **Identify patient’s PCP; alert care team if no PCP and/or begin PCP search. If patient uninsured, seek PCP at local FQHC.**    2. **Contact PCP and notify of patient’s admission, diagnoses including diagnoses of unmet social needs, and predicted discharge date.**    3. **Schedule PCP follow-up appointment within 7–14 days of discharge according to patient/caregiver availability and after assessing transportation needs.**    4. **Update PCP on discharge including medication changes, interventions, social needs.** | **√**  **√** | **√** | **√**  **√** |
| 1. **Ensure medication access and safety**    1. **Reconcile medications on admission**    2. **Ensure patient is able to obtain medications (medication covered by insurance, patient can obtain co-pay, pharmacy availability)**    3. **Train patient, with help of interdisciplinary team, how to properly use discharge medications and how these relate to the medications patient was taking prior to admission.**    4. **Reconcile medications on discharge and communicate with patient and all parties** | **√** | **√**  **√**  **√** | **√**  **√**  **√**  **√** |
| 1. **Home care and follow-up**    1. **Assess need for home services and set up.**    2. **Communicate with home services (including home health aides) or long-term facility to ensure appropriate follow- up**    3. **Arrange post-discharge follow-up.** |  | **√**  **√** | **√**  **√**  **√** |
| 1. **Patient education in their preferred language**    1. **Review hospitalization course and interventions.**    2. **Explain medications, treatments, goals and next steps.**    3. **Perform teach back† with patients.**    4. **Provide anticipatory guidance on when to return to the emergency room / seek care.** | **√**  **√**  **√** | **√**  **√**  **√** | **√**  **√**  **√**  **√** |
| 1. **Communication with all parties**    1. **Incorporate patients’ families, caregivers, aides and supporters in patient education.**    2. **Provide discharge summary to care team (patient, PCP, homecare or long-term facility, etc.).**    3. **Reconcile medications with patients’ pharmacy.**    4. **Communicate with referring outpatient services and clinic and community organizations.** | **√** | **√**  **√** | **√**  **√**  **√** |

NOTES: Abbreviations: PCP, primary care physician, FQHC, federally-qualified health center. *Sustainable solutions are patient-centered, multidisciplinary and avoid unintended consequences. †Teach-back is the process of explaining information to patients and asking them to restate the information to assess accuracy. The instructor then repeats the process until the patient demonstrates correct recall and comprehension. **REFERR TO APPENDIX N SLIDE 19**

**Adapted by**: Hassan I. & Scott S. **References**: Soong C, Daub S, Lee J, Majewski C, Musing E, Nord P, Wyman R et. al. Developing of a checklist of safe discharge practices for hospital patients. *Journal of Hospital Medicine*. 2013;8:444-419.
