# Supplementary material for: Structural Competency: A Faculty Development Workshop Series for Anti-racism in Medical Education
Source: MedEdPORTAL. 2025 Feb 7;21:11492. doi: 10.15766/mep_2374-8265.11492 (PMC11802914; doi:10.15766/mep_2374-8265.11492)
Supplement: Supplementary file 1 — 1 - Introduction to SC.pptx1 - Facilitator Guide.docx1 - SC Rubric Handout.docx1 - Sample SC Learning Goals.docx2 - Resident Reports & Case-Based Presentations.pptx2 - Facilitator Guide.docx2 - Structural Differential Handout.docx2 - Small-Group Handout.docx3 - Demystifying SC.pptx3 - Facilitator Guide.docx3 - SC One-Minute Preceptor Handout.docx3 - SC SNAPPS Handout.docx3 - Role-Play Scenarios.docx4 - SC Hospital-Based Teaching.pptx4 - Facilitator Guide.docx4 - Daily Inpatient Checklist.docx4 - SC Discharge Checklist.docx4 - Small-Group Scenarios.docxPre- and Postsurveys.docx [file mep_2374-8265.11492-s001.zip › R. 4 - Small-Group Scenarios.docx]

***Instructions:*** *Take some time to read the following presentation. Assign the following roles: ward attending to provide feedback, learner, observer/notetaker*.

***Task 1:*** *Please provide feedback to the learner on bias in documentation. When providing feedback on bias in documentation, facilitators should create a safe, supportive environment that emphasizes learning and self-reflection. Focus on specific examples of biased language, explain its potential impact on patient care and team dynamics, and encourage learners to consider more neutral, patient-centered alternatives. Tie the discussion to broader goals of professionalism and equity.*

***Task 2:*** *Please provide feedback to the learner on developing a structurally competent assessment and plan and help them formulate a new assessment and plan. Encourage learners to consider social determinants of health, structural barriers, and systemic inequities that impact patient care. Support learners in formulating a revised assessment and plan that integrates patient-centered, equity-focused strategies and aligns with the broader context of the patient's life and needs.*

Mr. R is a 28-year-old sickle cell patient with chronic left hip osteomyelitis who comes to the ED stating he has 10/10 pain “all up in my arms and legs.” He is narcotic dependent and, in our ED, frequently. At home he reportedly takes 100 mg OxyContin BID and oxycodone 5 mg for breakthrough pain. Over the past few days, he says that he has taken 2 tabs every 4–6 hours. About 3 months ago, patient states that the housing authority moved him to a new neighborhood and he now has to wheel himself in a manual wheelchair up 3 blocks from the bus stop. Yesterday afternoon, he was hanging out with friends outside McDonald’s where he wheeled himself around more than usual and got dehydrated due to the heat. He believes that this, along with some “stressful situations,” has precipitated his current crisis. Pain is aching in quality, severe (10/10), and has not been helped by any of the narcotic medications he says he has already taken.

On physical exam, he appears to be in distress. He has no fever and his pulse ox is 96% on RA. The rest of the physical exam is normal although he reports tenderness to palpation on the left hip.

**Plan:**

Admission for sickle cell crisis:

-   Pain regimen

-   Fluids

-   Labs

-   Incentive spirometer
