# Supplementary material for: Structural Competency: A Faculty Development Workshop Series for Anti-racism in Medical Education
Source: MedEdPORTAL. 2025 Feb 7;21:11492. doi: 10.15766/mep_2374-8265.11492 (PMC11802914; doi:10.15766/mep_2374-8265.11492)
Supplement: Supplementary file 1 — 1 - Introduction to SC.pptx1 - Facilitator Guide.docx1 - SC Rubric Handout.docx1 - Sample SC Learning Goals.docx2 - Resident Reports & Case-Based Presentations.pptx2 - Facilitator Guide.docx2 - Structural Differential Handout.docx2 - Small-Group Handout.docx3 - Demystifying SC.pptx3 - Facilitator Guide.docx3 - SC One-Minute Preceptor Handout.docx3 - SC SNAPPS Handout.docx3 - Role-Play Scenarios.docx4 - SC Hospital-Based Teaching.pptx4 - Facilitator Guide.docx4 - Daily Inpatient Checklist.docx4 - SC Discharge Checklist.docx4 - Small-Group Scenarios.docxPre- and Postsurveys.docx [file mep_2374-8265.11492-s001.zip › N. 4 - SC Hospital-Based Teaching.pptx]

## Slide 1
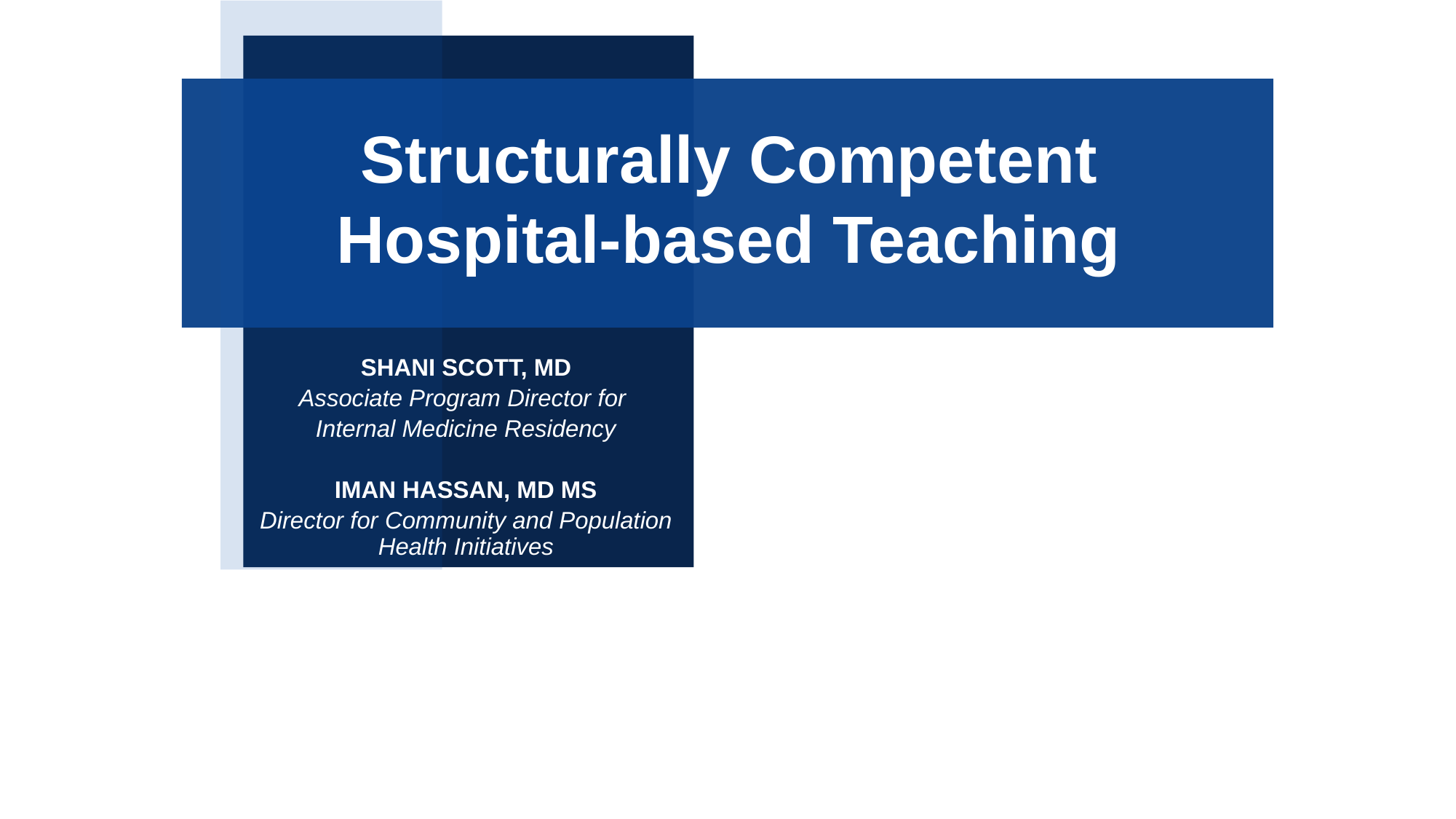

Structurally Competent Hospital-based Teaching
SHANI SCOTT, MD
Associate Program Director for
Internal Medicine Residency
IMAN HASSAN, MD MS
Director for Community and Population Health Initiatives

## Slide 2
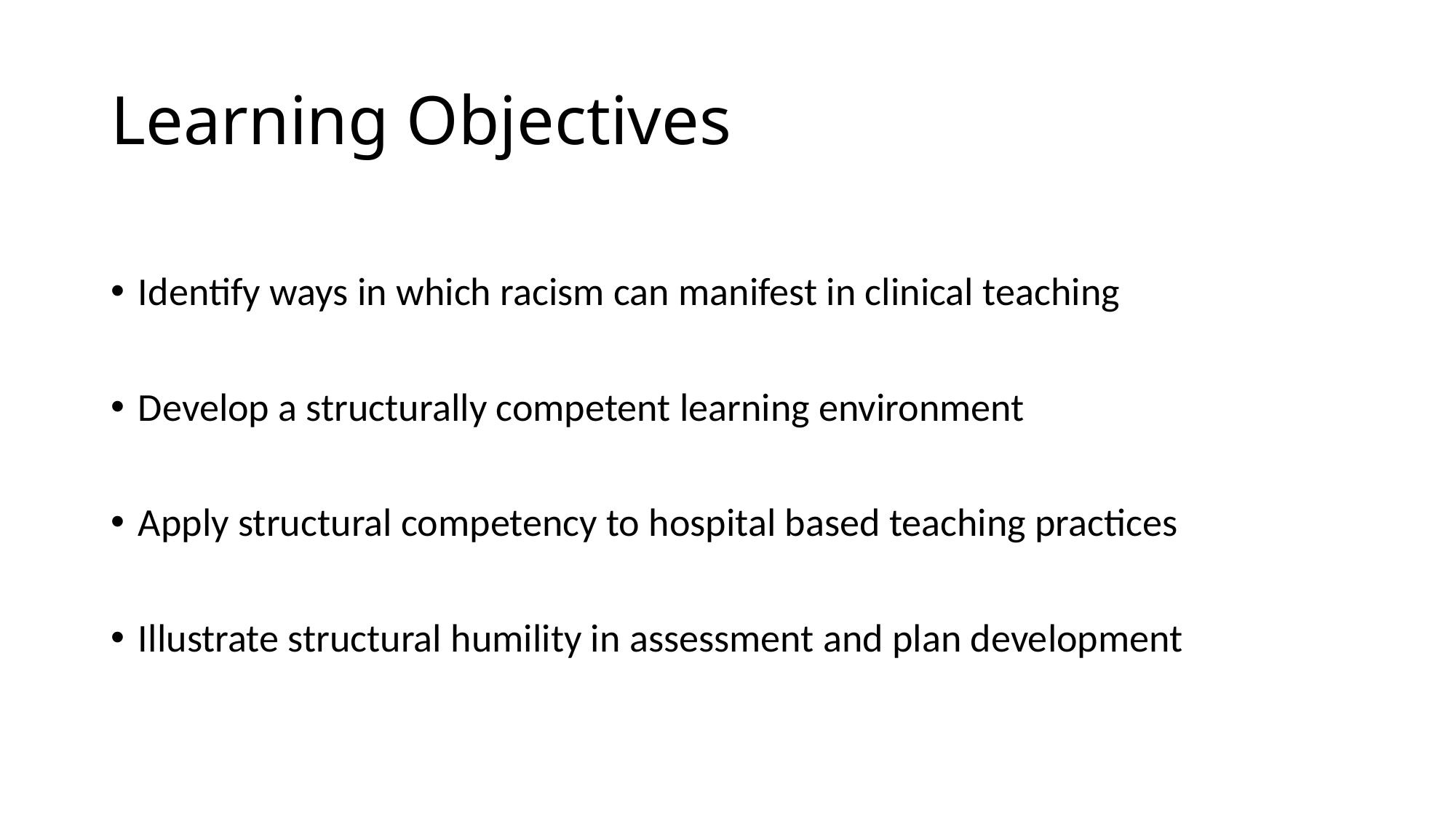

# Learning Objectives
Identify ways in which racism can manifest in clinical teaching
Develop a structurally competent learning environment
Apply structural competency to hospital based teaching practices
Illustrate structural humility in assessment and plan development

## Slide 3
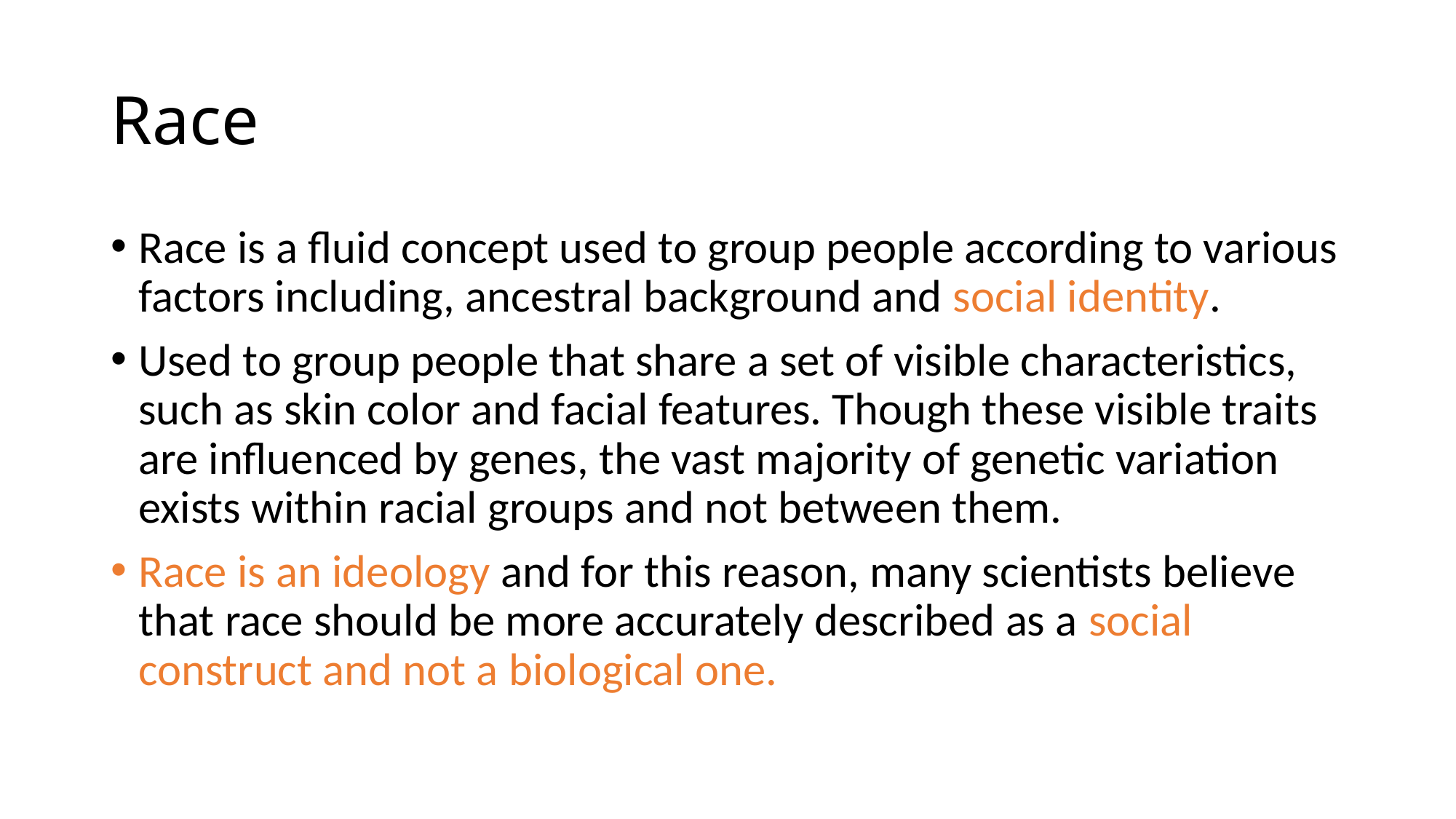

# Race
Race is a fluid concept used to group people according to various factors including, ancestral background and social identity.
Used to group people that share a set of visible characteristics, such as skin color and facial features. Though these visible traits are influenced by genes, the vast majority of genetic variation exists within racial groups and not between them.
Race is an ideology and for this reason, many scientists believe that race should be more accurately described as a social construct and not a biological one.

## Slide 4
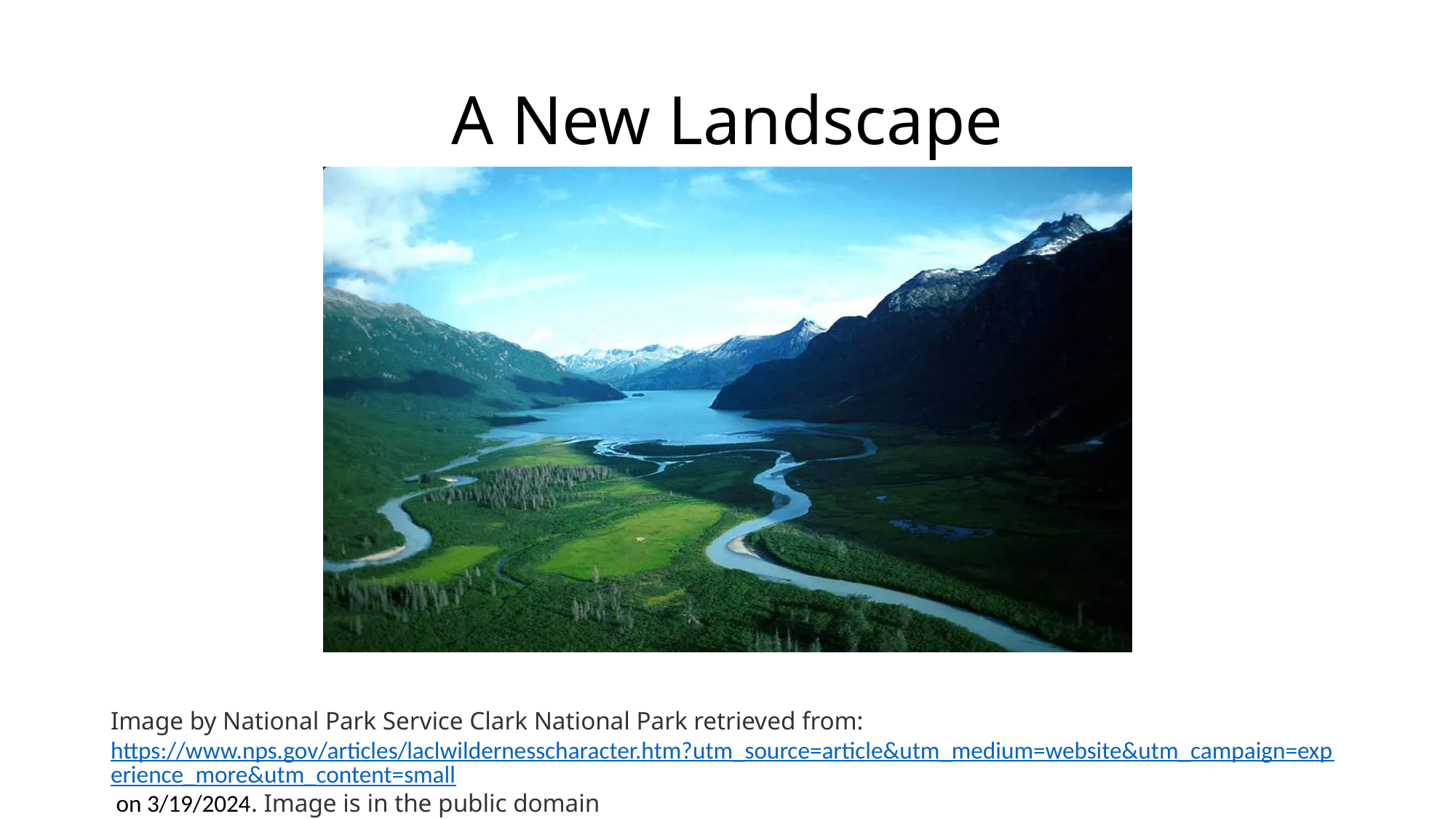

# A New Landscape
Image by National Park Service Clark National Park retrieved from: https://www.nps.gov/articles/laclwildernesscharacter.htm?utm_source=article&utm_medium=website&utm_campaign=experience_more&utm_content=small on 3/19/2024. Image is in the public domain

## Slide 5
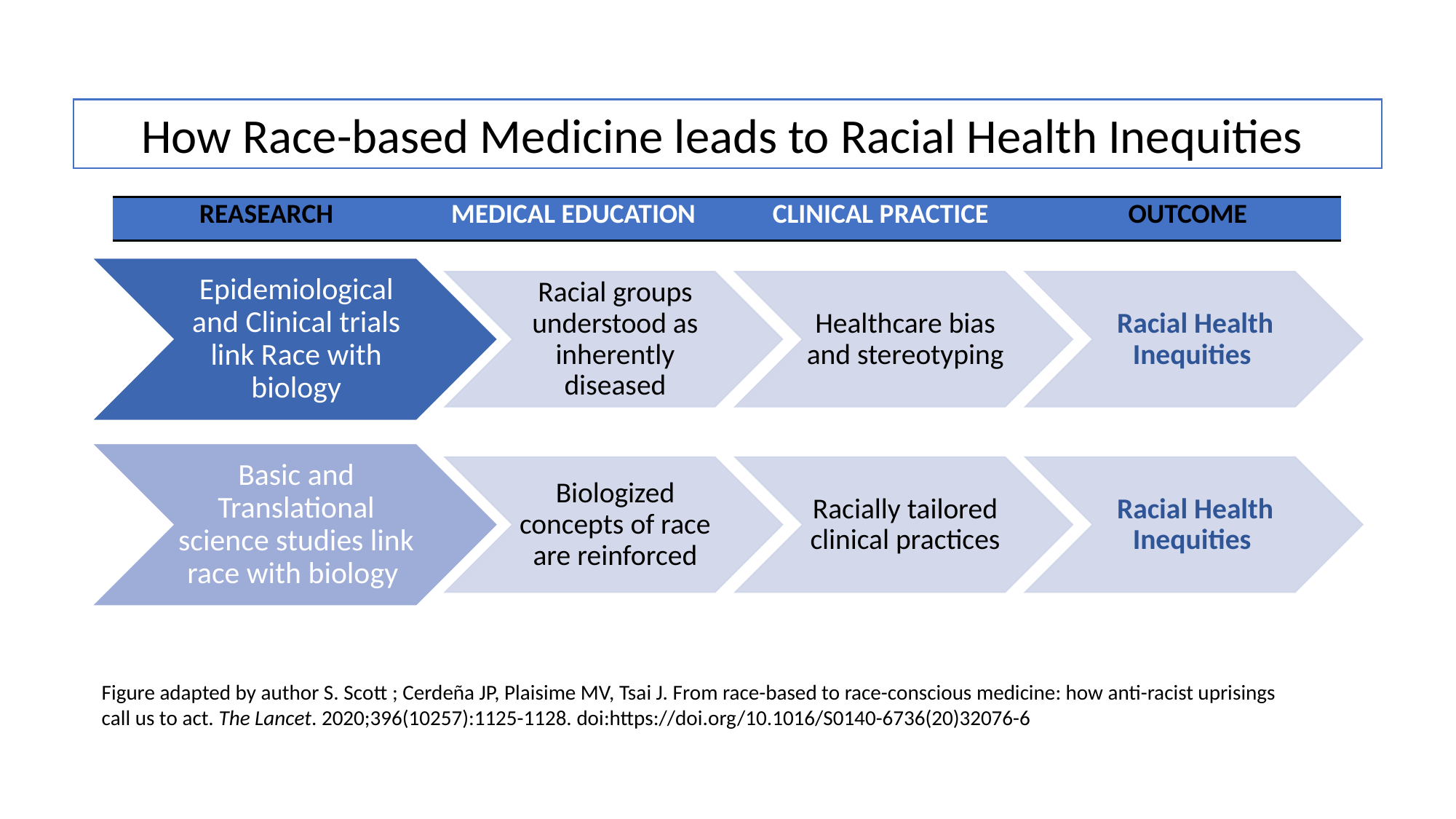

How Race-based Medicine leads to Racial Health Inequities
| REASEARCH | MEDICAL EDUCATION | CLINICAL PRACTICE | OUTCOME |
| --- | --- | --- | --- |
Figure adapted by author S. Scott ; Cerdeña JP, Plaisime MV, Tsai J. From race-based to race-conscious medicine: how anti-racist uprisings call us to act. The Lancet. 2020;396(10257):1125-1128. doi:https://doi.org/10.1016/S0140-6736(20)32076-6
‌

## Slide 6
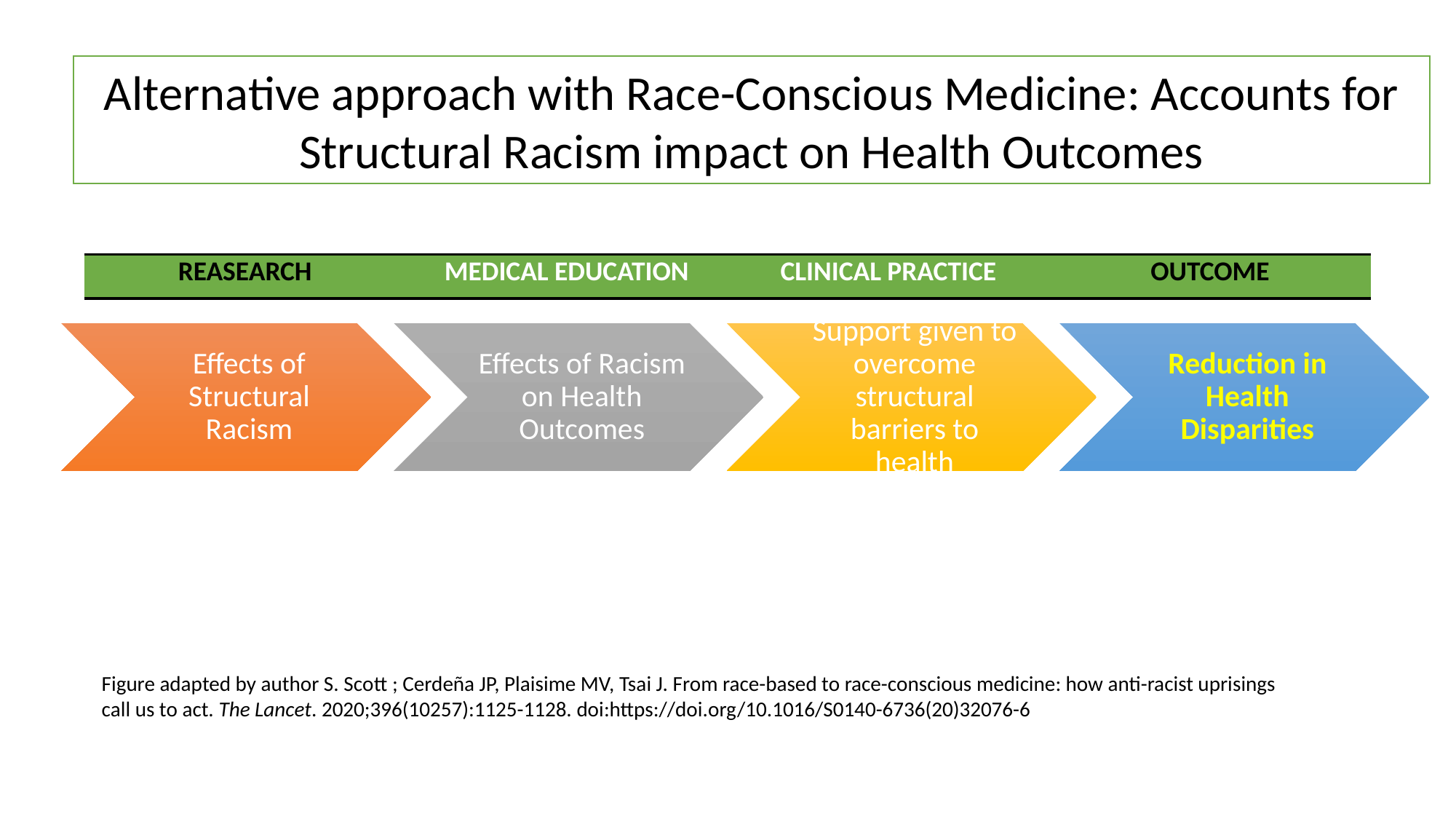

Alternative approach with Race-Conscious Medicine: Accounts for Structural Racism impact on Health Outcomes
| REASEARCH | MEDICAL EDUCATION | CLINICAL PRACTICE | OUTCOME |
| --- | --- | --- | --- |
Figure adapted by author S. Scott ; Cerdeña JP, Plaisime MV, Tsai J. From race-based to race-conscious medicine: how anti-racist uprisings call us to act. The Lancet. 2020;396(10257):1125-1128. doi:https://doi.org/10.1016/S0140-6736(20)32076-6
‌

## Slide 7
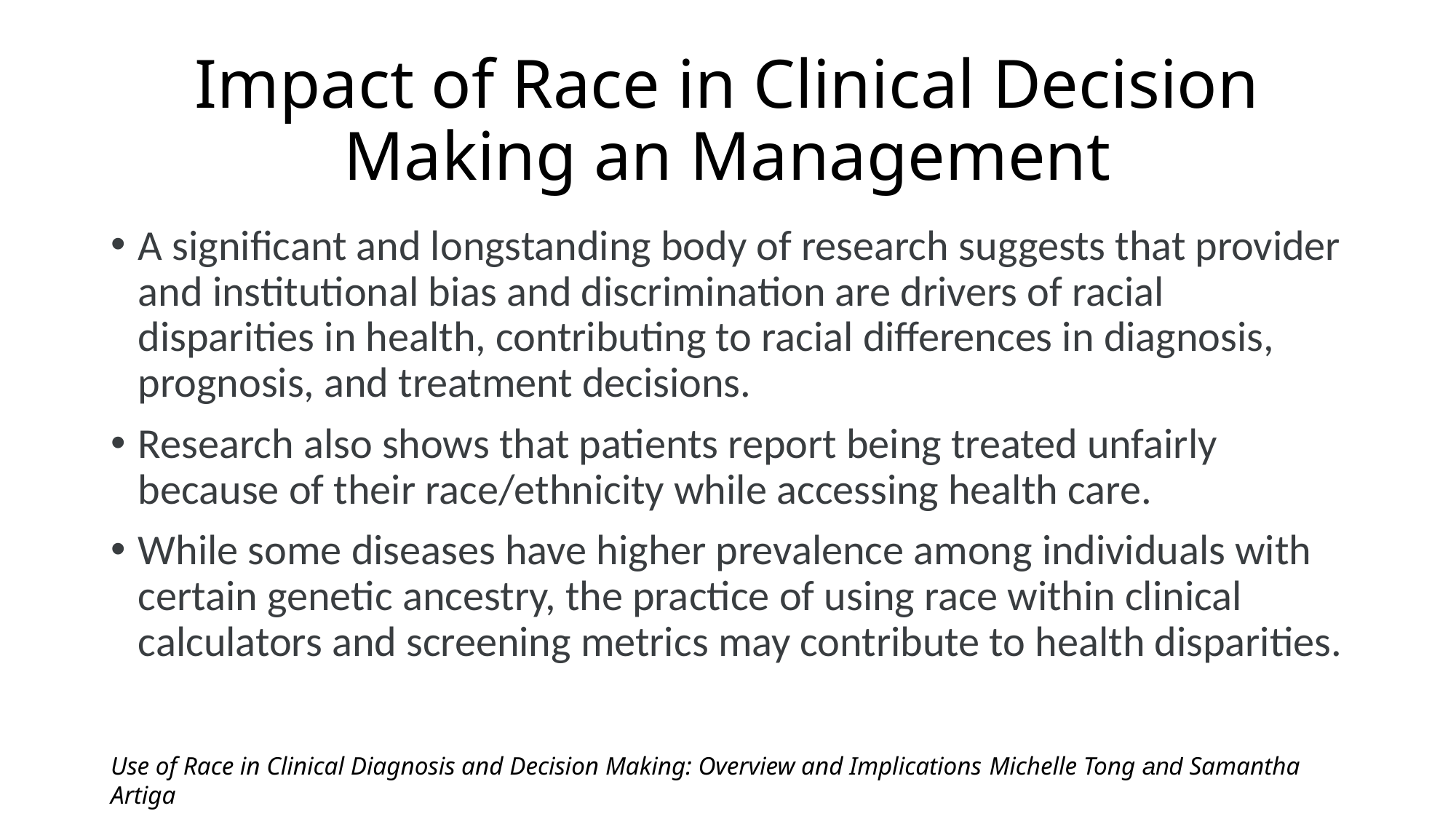

# Impact of Race in Clinical Decision Making an Management
A significant and longstanding body of research suggests that provider and institutional bias and discrimination are drivers of racial disparities in health, contributing to racial differences in diagnosis, prognosis, and treatment decisions.
Research also shows that patients report being treated unfairly because of their race/ethnicity while accessing health care.
While some diseases have higher prevalence among individuals with certain genetic ancestry, the practice of using race within clinical calculators and screening metrics may contribute to health disparities.
Use of Race in Clinical Diagnosis and Decision Making: Overview and Implications Michelle Tong and Samantha Artiga

## Slide 8
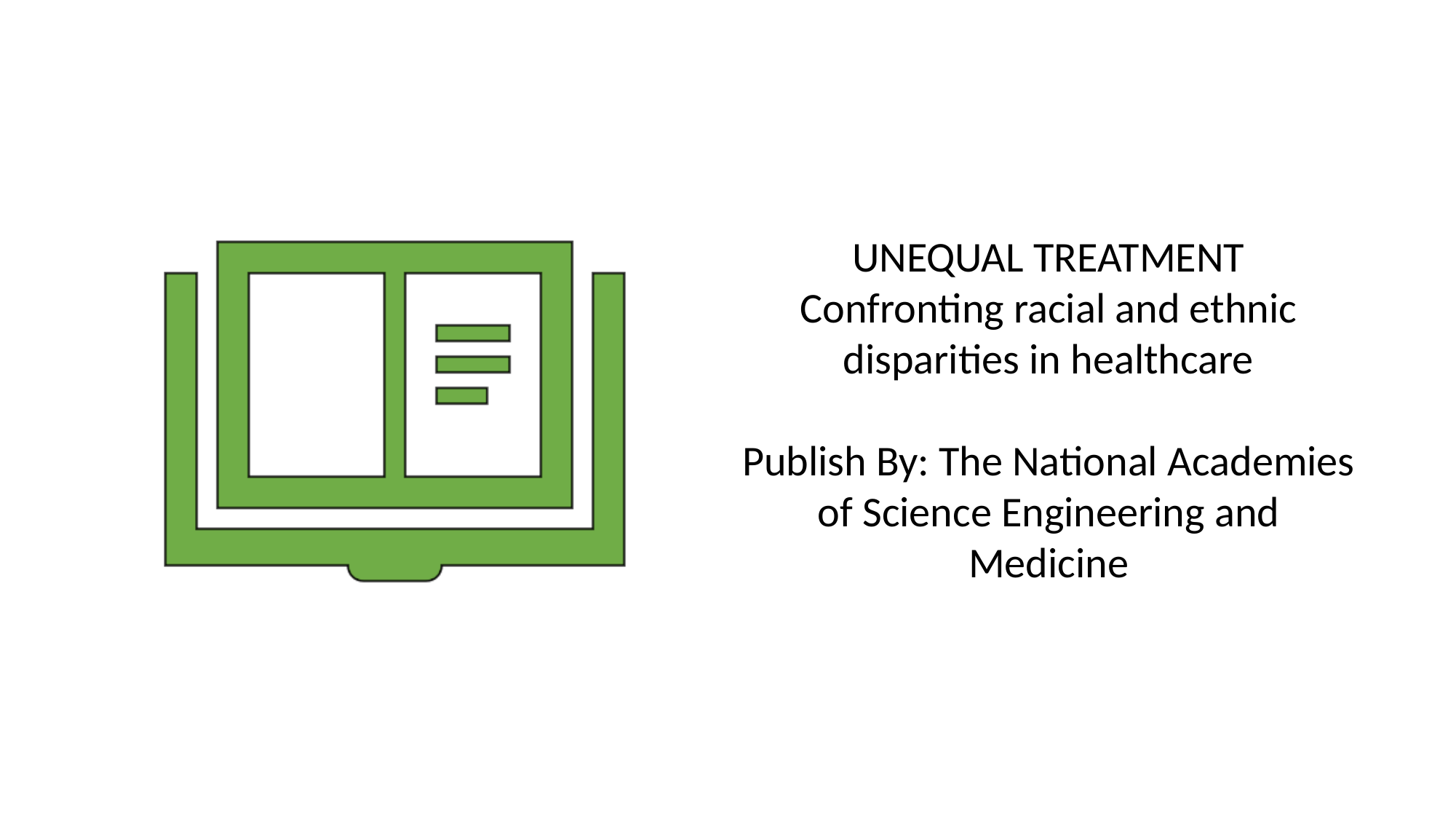

UNEQUAL TREATMENT
Confronting racial and ethnic disparities in healthcare
Publish By: The National Academies of Science Engineering and Medicine

## Slide 9
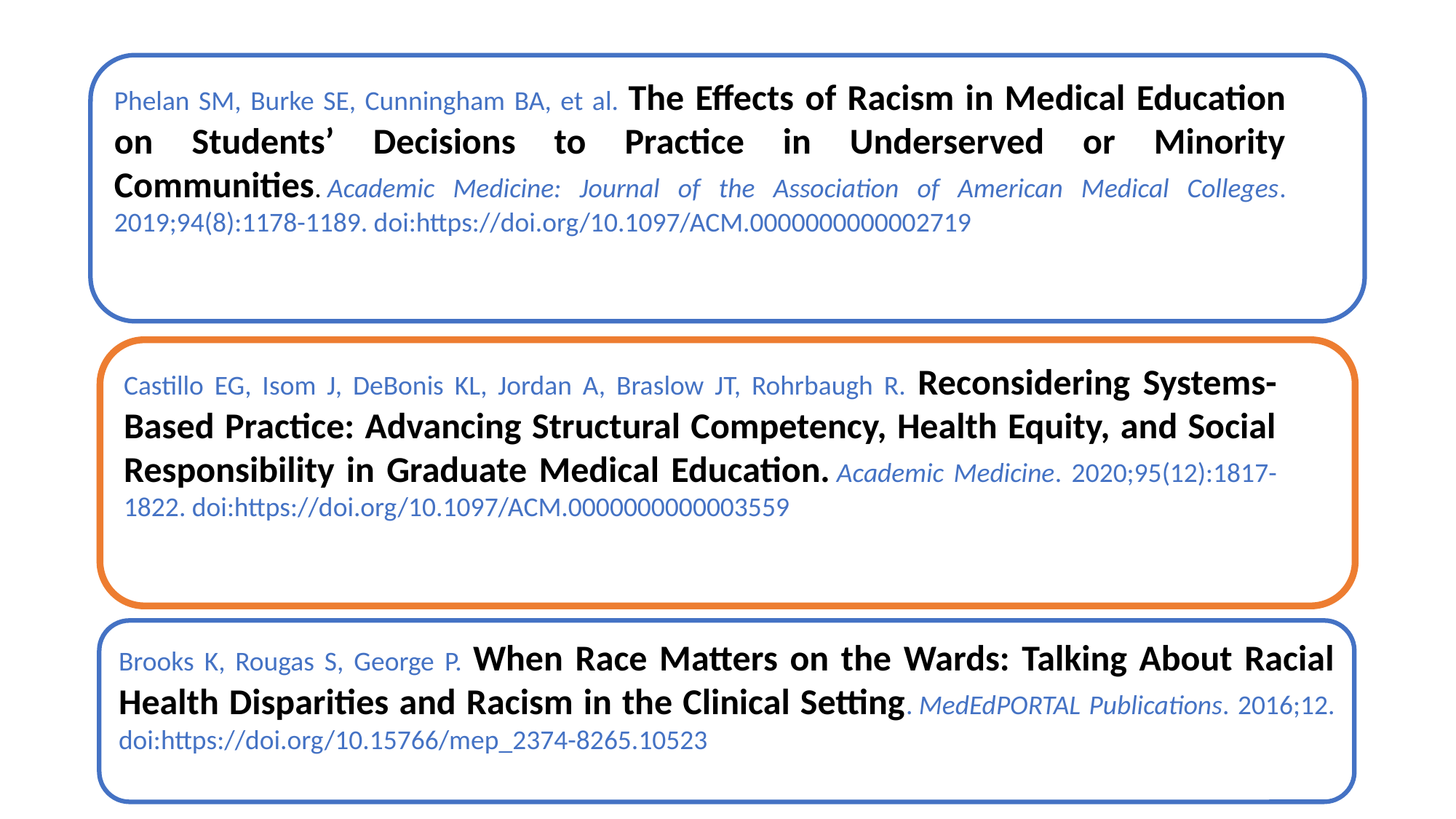

Phelan SM, Burke SE, Cunningham BA, et al. The Effects of Racism in Medical Education on Students’ Decisions to Practice in Underserved or Minority Communities. Academic Medicine: Journal of the Association of American Medical Colleges. 2019;94(8):1178-1189. doi:https://doi.org/10.1097/ACM.0000000000002719
Castillo EG, Isom J, DeBonis KL, Jordan A, Braslow JT, Rohrbaugh R. Reconsidering Systems-Based Practice: Advancing Structural Competency, Health Equity, and Social Responsibility in Graduate Medical Education. Academic Medicine. 2020;95(12):1817-1822. doi:https://doi.org/10.1097/ACM.0000000000003559
‌
Brooks K, Rougas S, George P. When Race Matters on the Wards: Talking About Racial Health Disparities and Racism in the Clinical Setting. MedEdPORTAL Publications. 2016;12. doi:https://doi.org/10.15766/mep_2374-8265.10523

## Slide 10
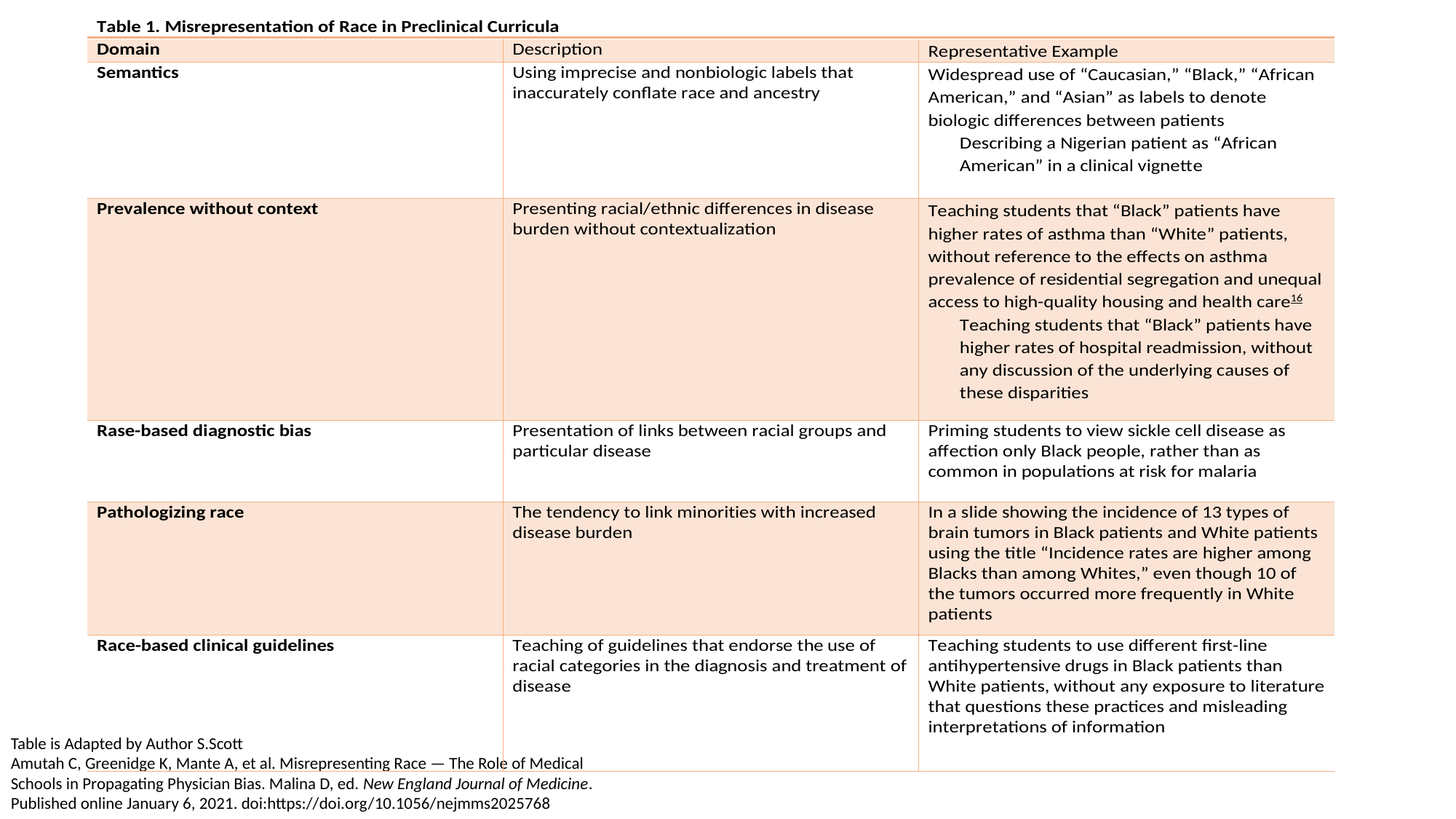

Table is Adapted by Author S.Scott
Amutah C, Greenidge K, Mante A, et al. Misrepresenting Race — The Role of Medical Schools in Propagating Physician Bias. Malina D, ed. New England Journal of Medicine. Published online January 6, 2021. doi:https://doi.org/10.1056/nejmms2025768
‌

## Slide 11
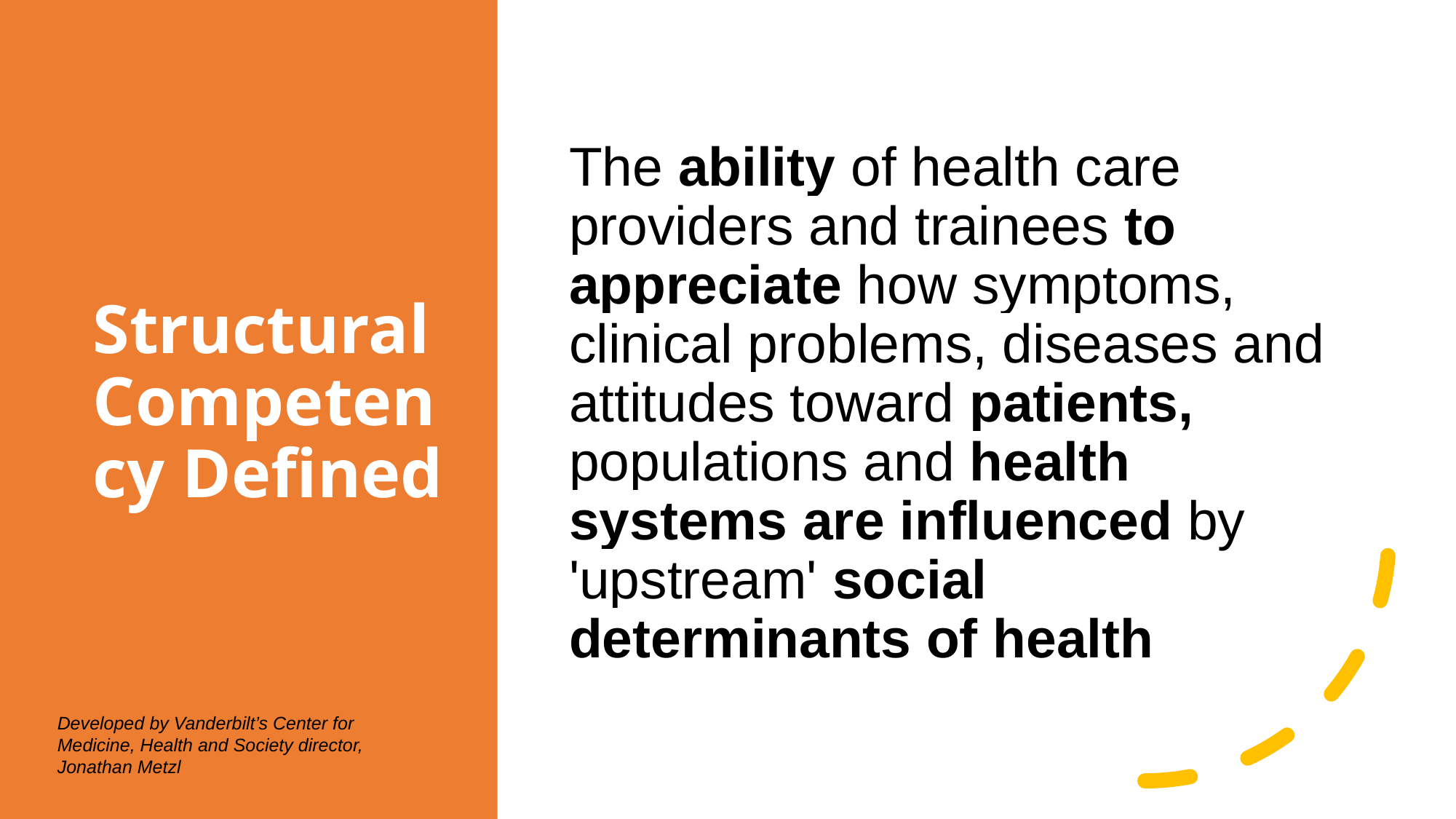

# Structural Competency Defined
The ability of health care providers and trainees to appreciate how symptoms, clinical problems, diseases and attitudes toward patients, populations and health systems are influenced by 'upstream' social determinants of health
Developed by Vanderbilt’s Center for Medicine, Health and Society director, Jonathan Metzl

## Slide 12
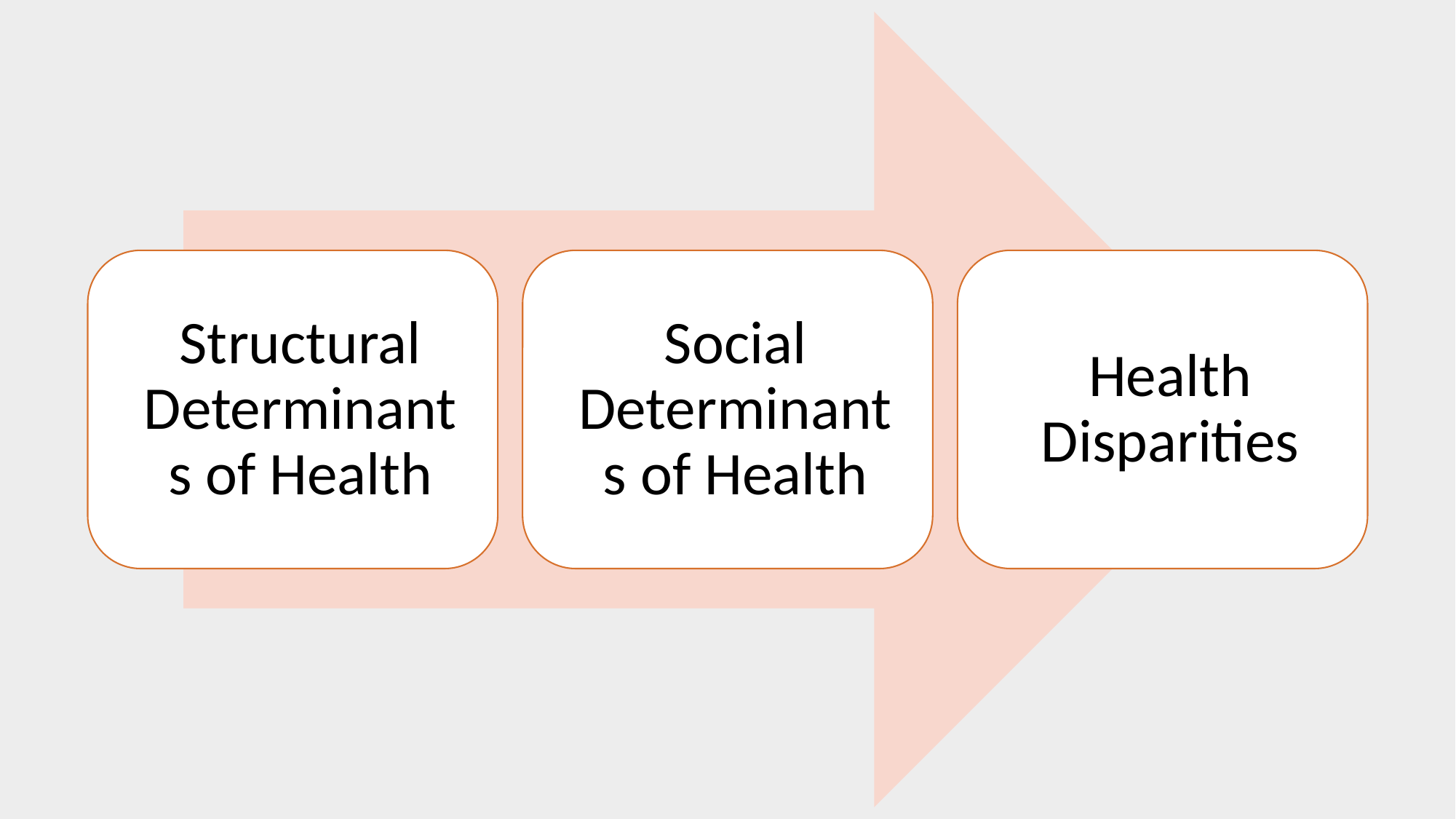

## Slide 13
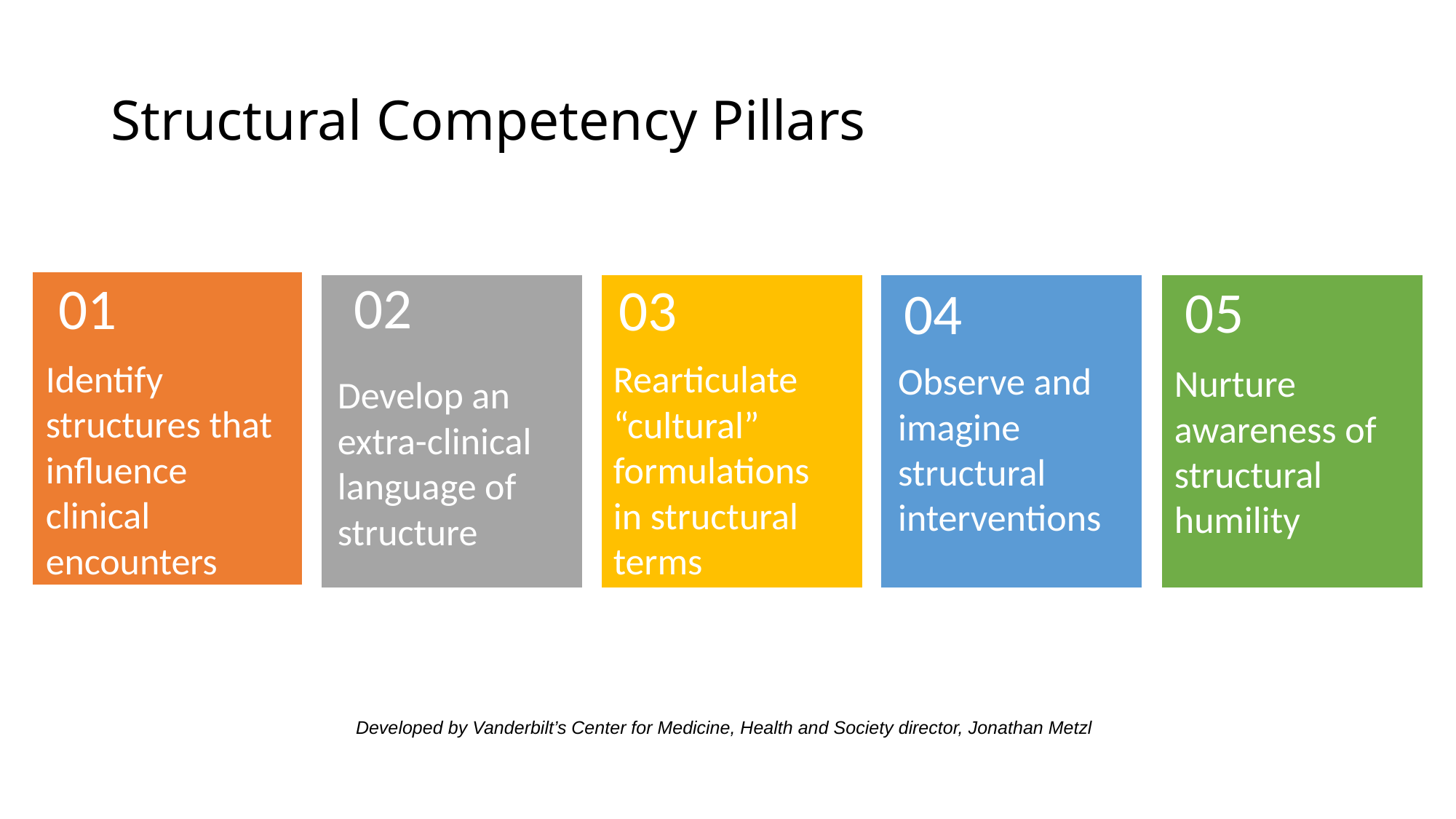

# Structural Competency Pillars
Identify structures that influence clinical encounters
Rearticulate “cultural” formulations in structural terms
Observe and imagine structural interventions
Nurture awareness of structural humility
Develop an extra-clinical language of structure
Developed by Vanderbilt’s Center for Medicine, Health and Society director, Jonathan Metzl

## Slide 14
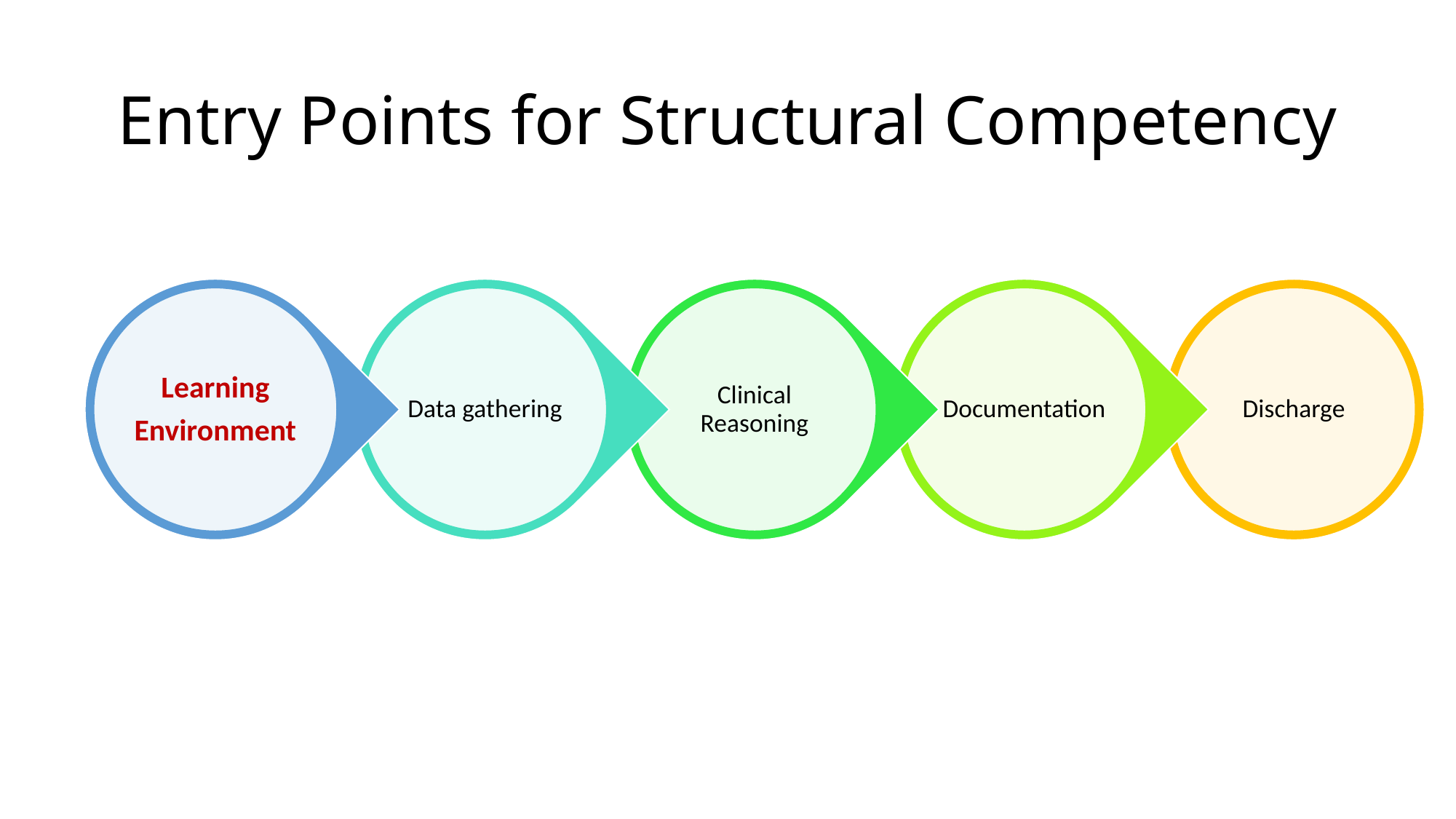

# Entry Points for Structural Competency

## Slide 15
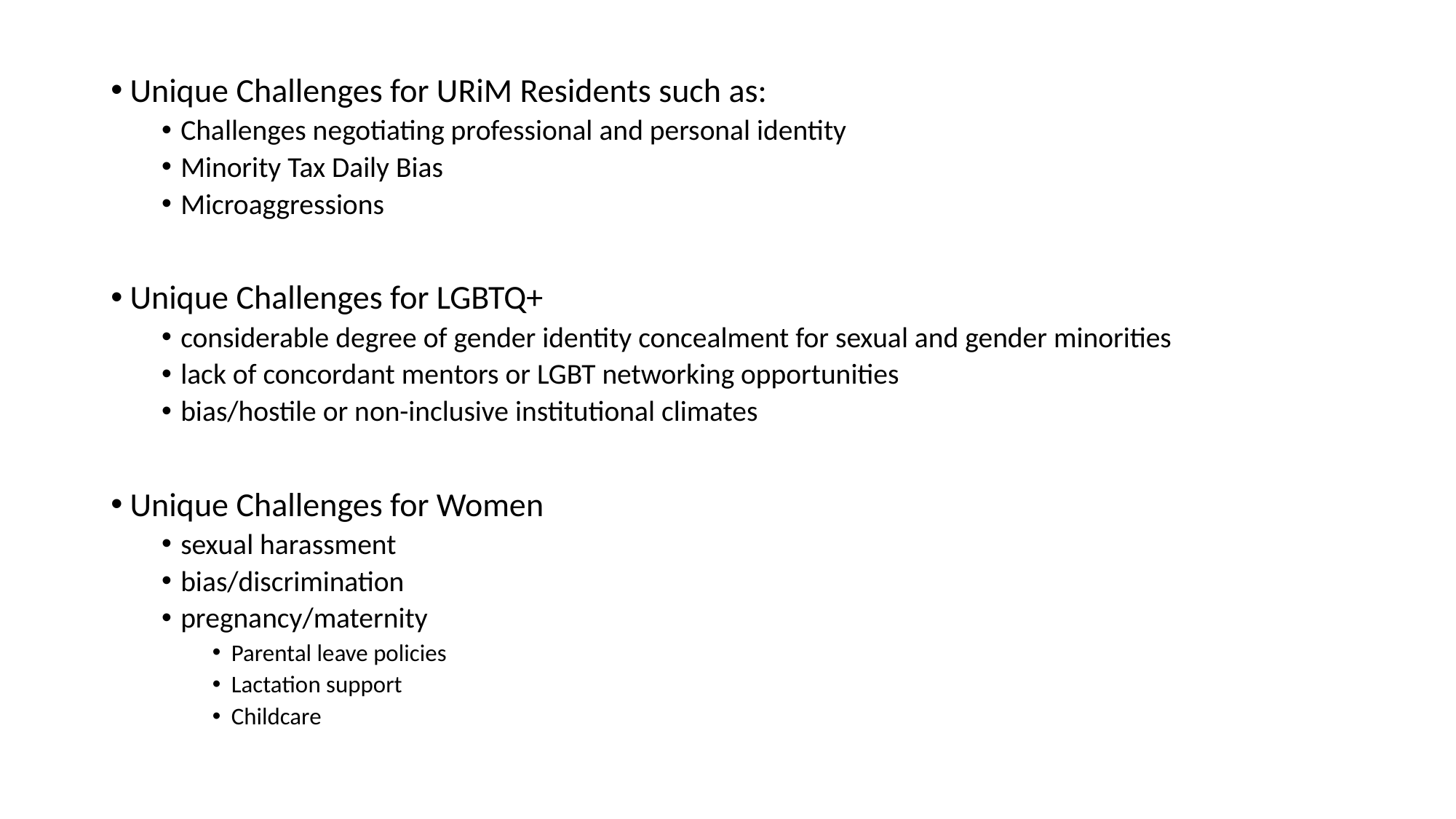

Unique Challenges for URiM Residents such as:
Challenges negotiating professional and personal identity
Minority Tax Daily Bias
Microaggressions
Unique Challenges for LGBTQ+
considerable degree of gender identity concealment for sexual and gender minorities
lack of concordant mentors or LGBT networking opportunities
bias/hostile or non-inclusive institutional climates
Unique Challenges for Women
sexual harassment
bias/discrimination
pregnancy/maternity
Parental leave policies
Lactation support
Childcare

## Slide 16
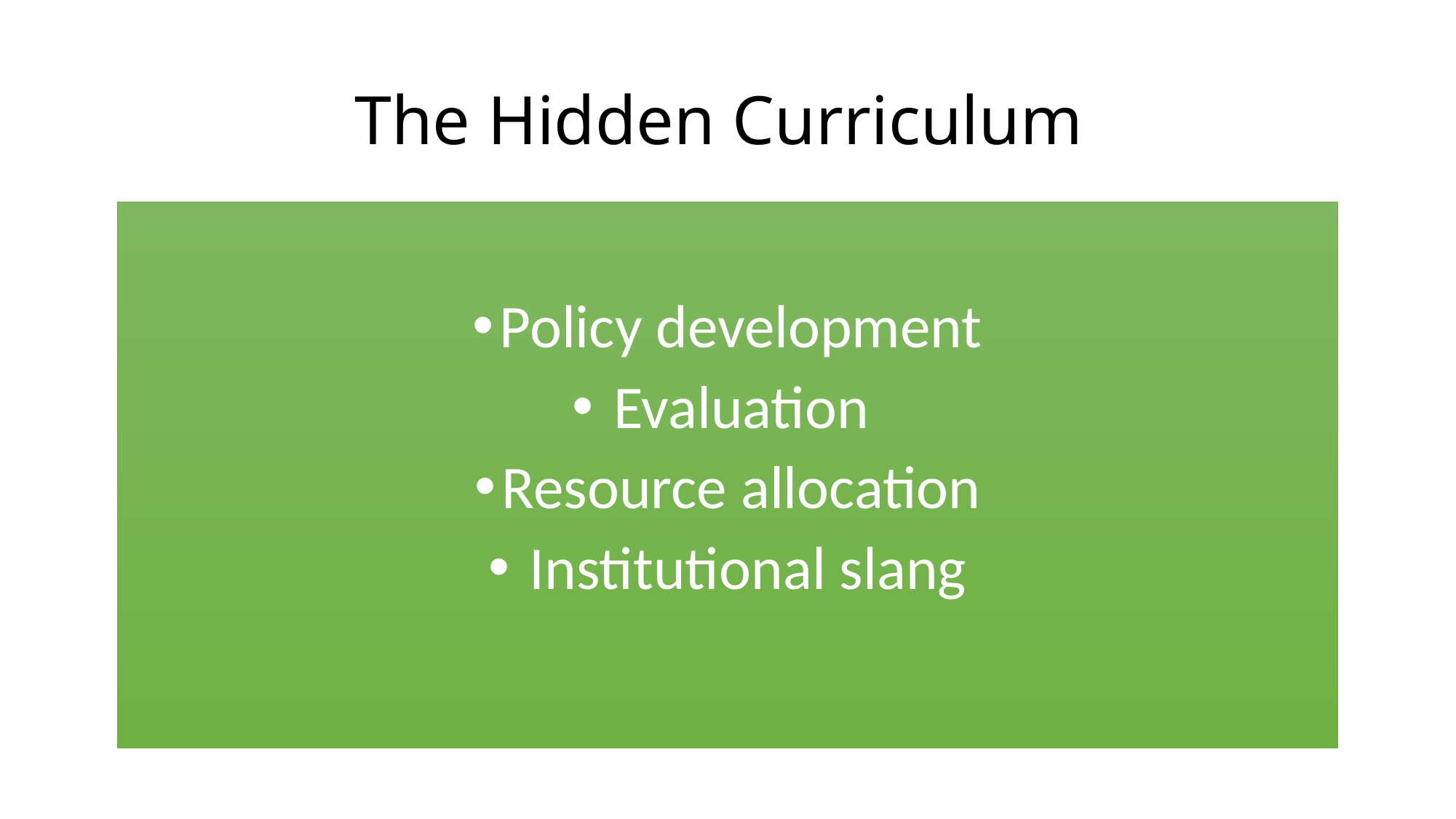

# The Hidden Curriculum
Policy development
 Evaluation
Resource allocation
 Institutional slang
“Set of influences that function at the level of organizational structure and culture.[He described the hidden curriculum generally as the] ‘understandings,’ customs, rituals, and taken-for-granted aspects of what goes on in the life-space we call medical education”

## Slide 17
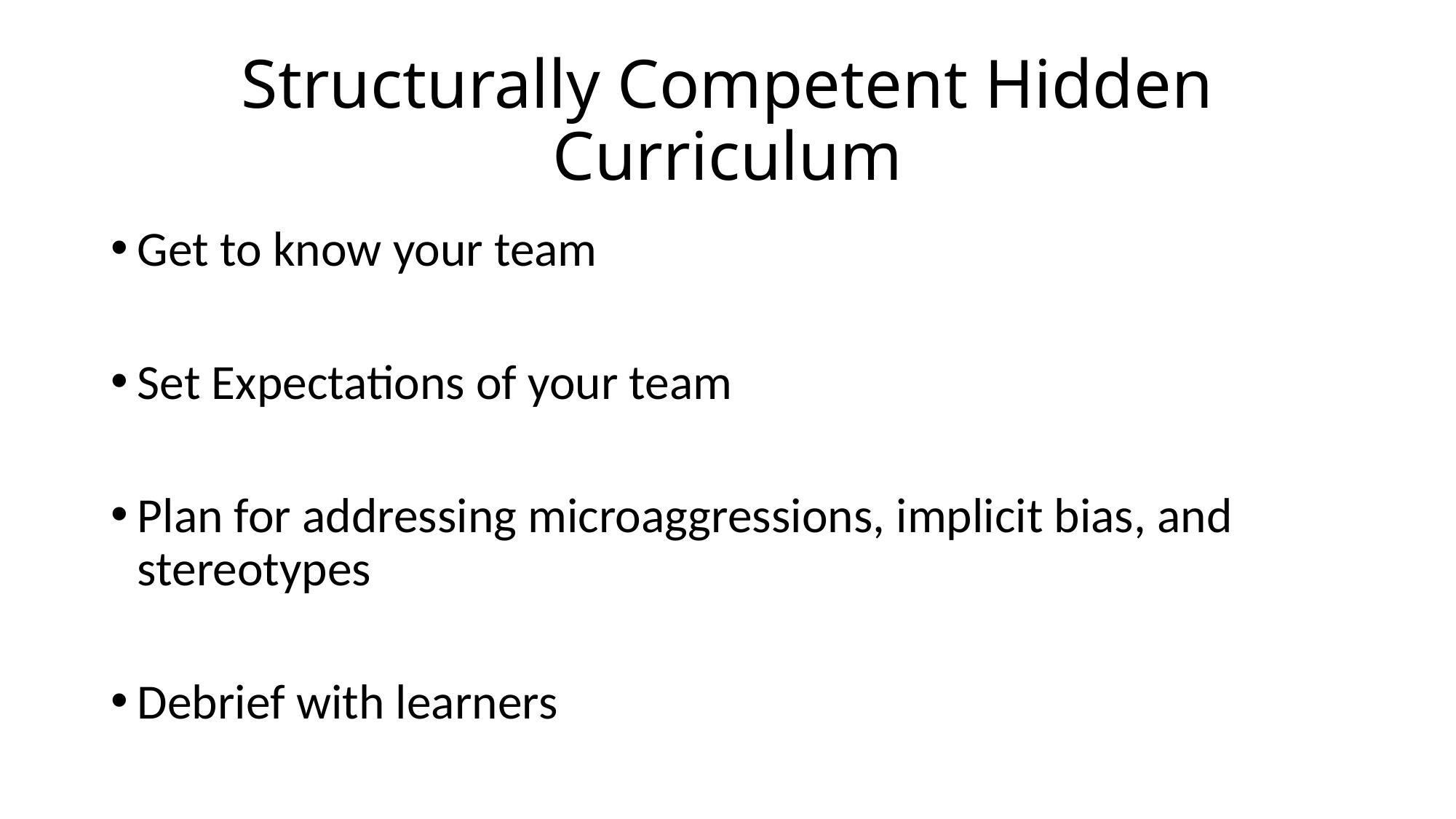

# Structurally Competent Hidden Curriculum
Get to know your team
Set Expectations of your team
Plan for addressing microaggressions, implicit bias, and stereotypes
Debrief with learners

## Slide 18
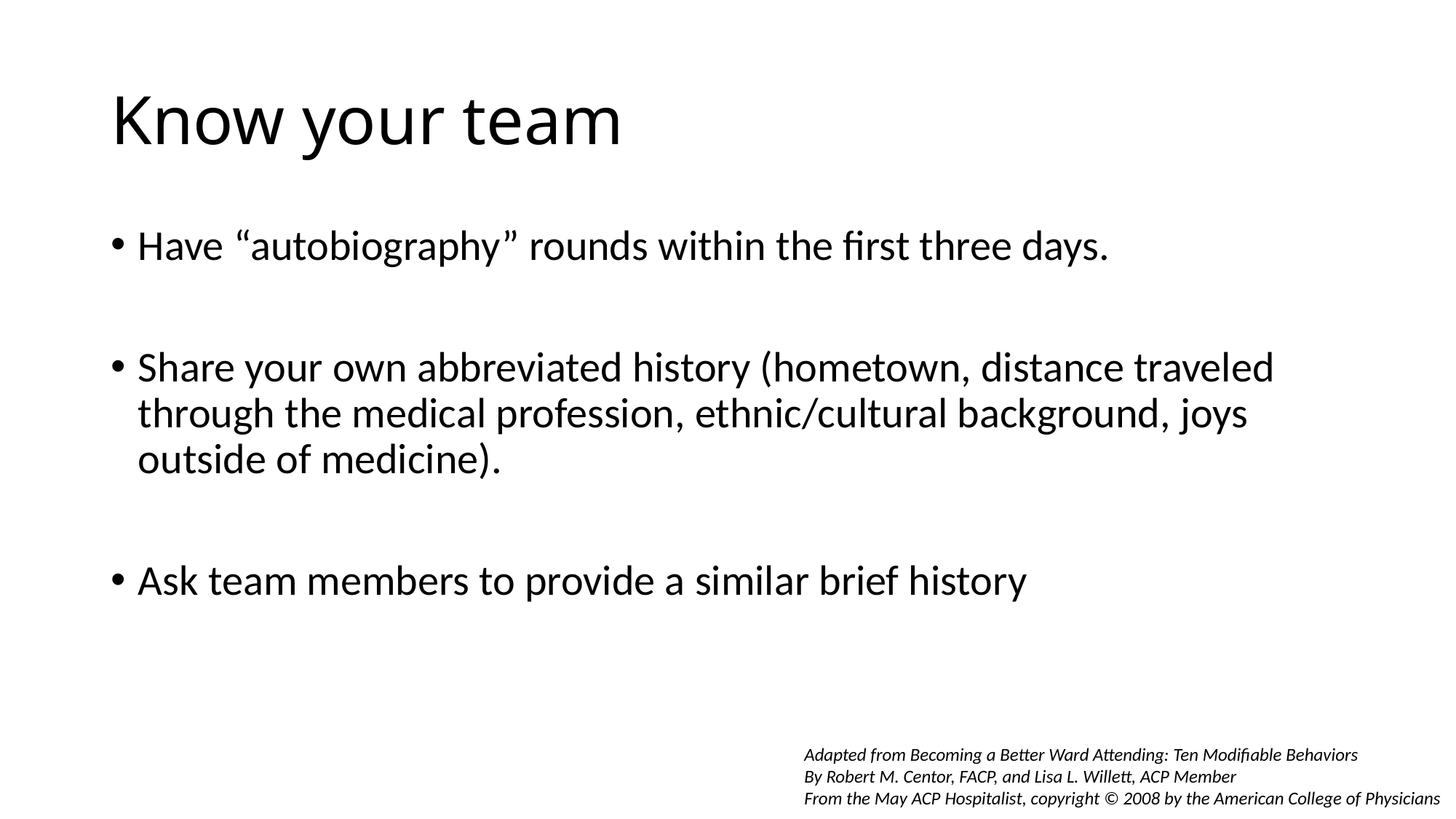

# Know your team
Have “autobiography” rounds within the first three days.
Share your own abbreviated history (hometown, distance traveled through the medical profession, ethnic/cultural background, joys outside of medicine).
Ask team members to provide a similar brief history
Adapted from Becoming a Better Ward Attending: Ten Modifiable Behaviors
By Robert M. Centor, FACP, and Lisa L. Willett, ACP Member
From the May ACP Hospitalist, copyright © 2008 by the American College of Physicians

## Slide 19
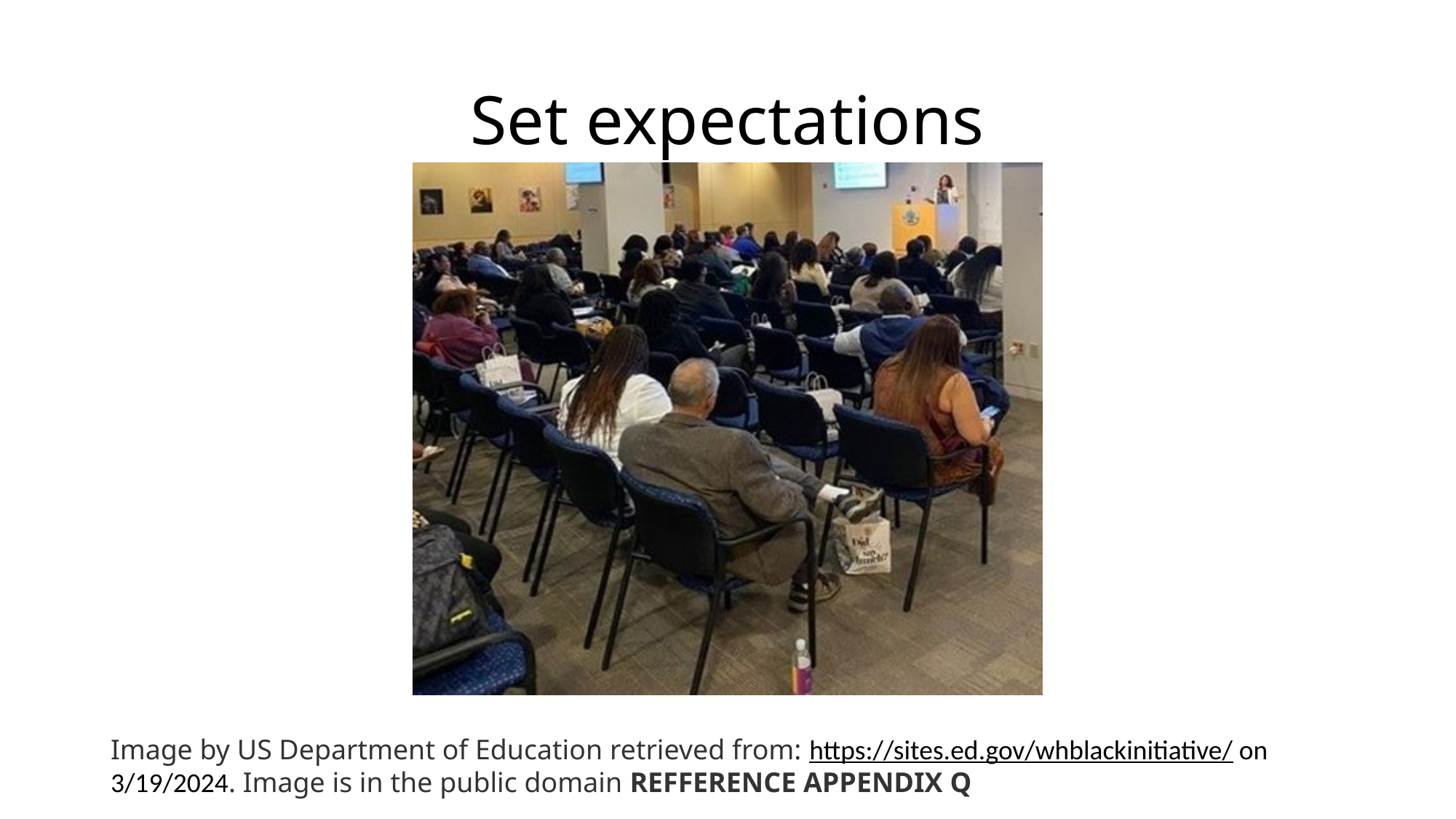

# Set expectations
Image by US Department of Education retrieved from: https://sites.ed.gov/whblackinitiative/ on 3/19/2024. Image is in the public domain REFFERENCE APPENDIX Q

## Slide 20
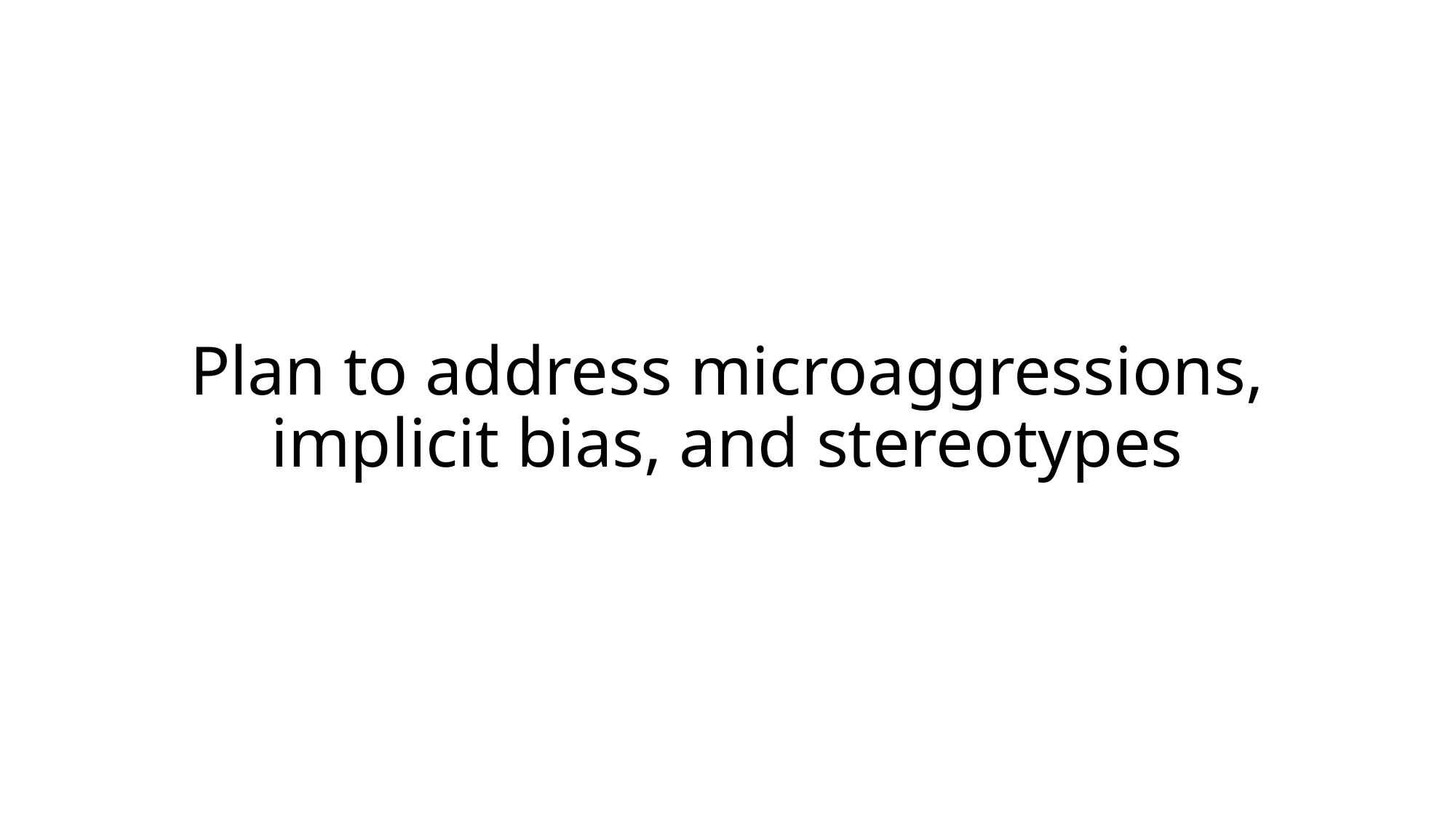

# Plan to address microaggressions, implicit bias, and stereotypes

## Slide 21
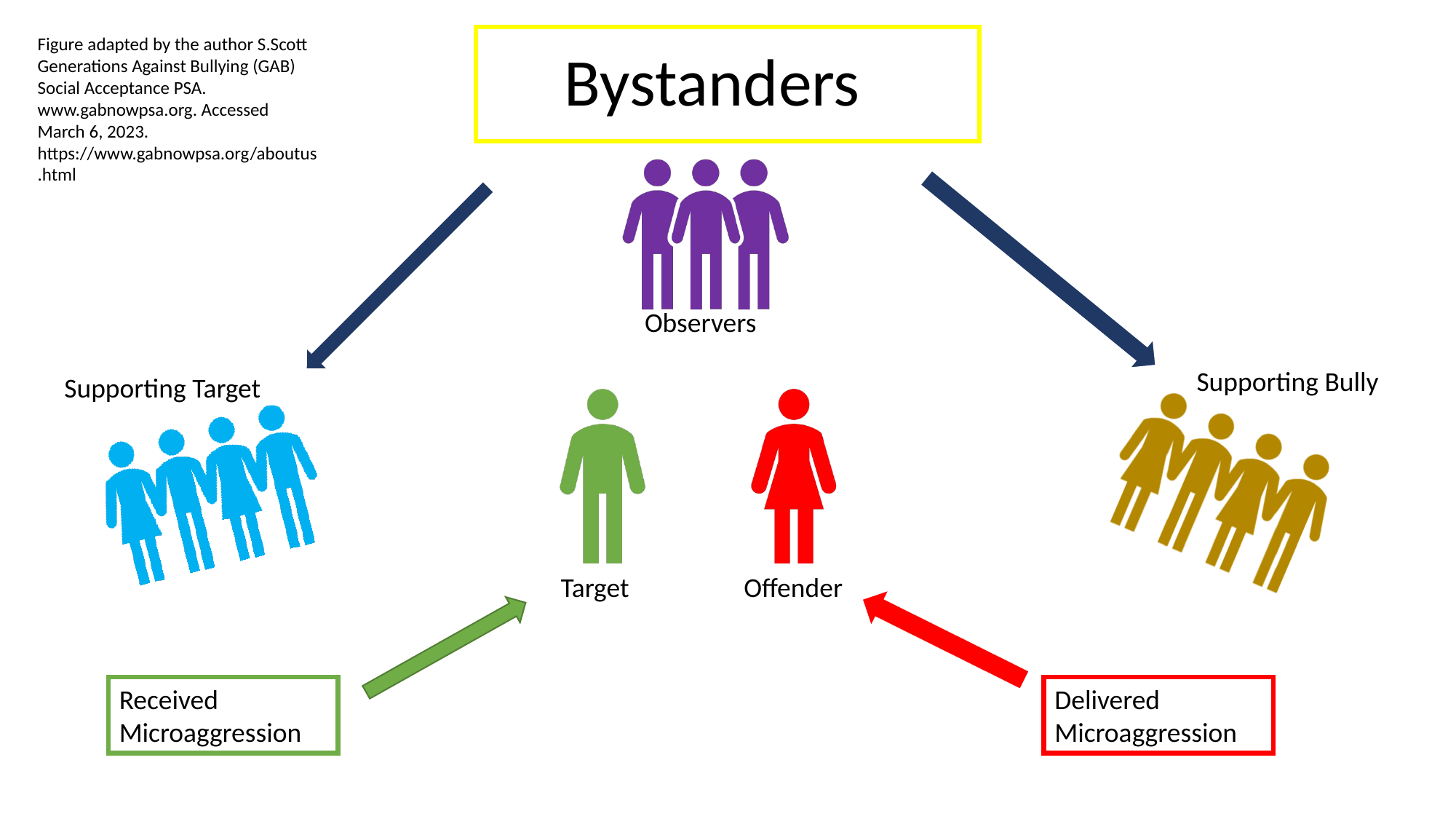

Figure adapted by the author S.Scott Generations Against Bullying (GAB) Social Acceptance PSA. www.gabnowpsa.org. Accessed March 6, 2023. https://www.gabnowpsa.org/aboutus.html
‌
# Bystanders
Observers
Supporting Bully
Supporting Target
Target
Offender
Received Microaggression
Delivered Microaggression

## Slide 22
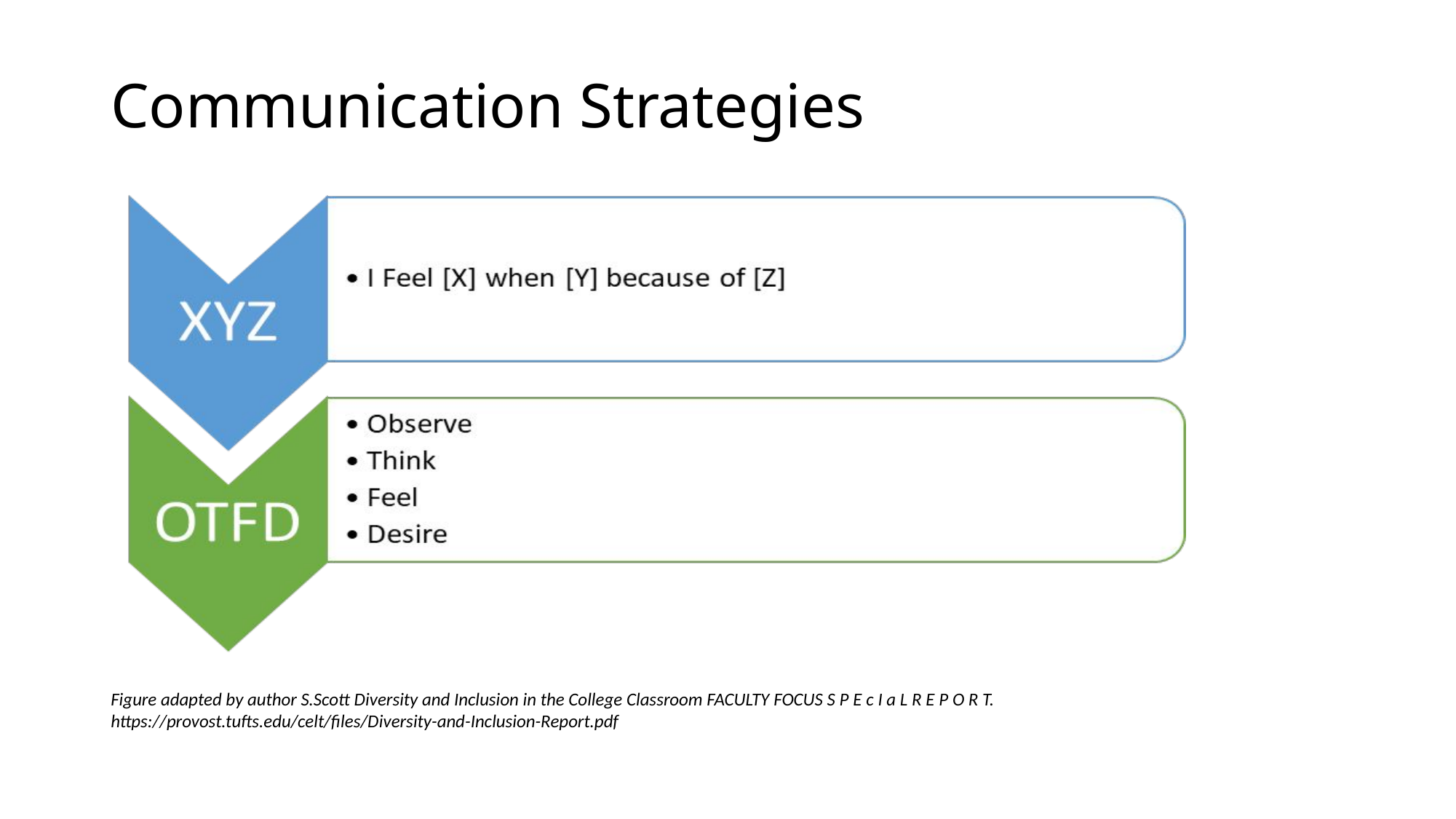

# Communication Strategies
Figure adapted by author S.Scott Diversity and Inclusion in the College Classroom FACULTY FOCUS S P E c I a L R E P O R T. https://provost.tufts.edu/celt/files/Diversity-and-Inclusion-Report.pdf
‌

## Slide 23
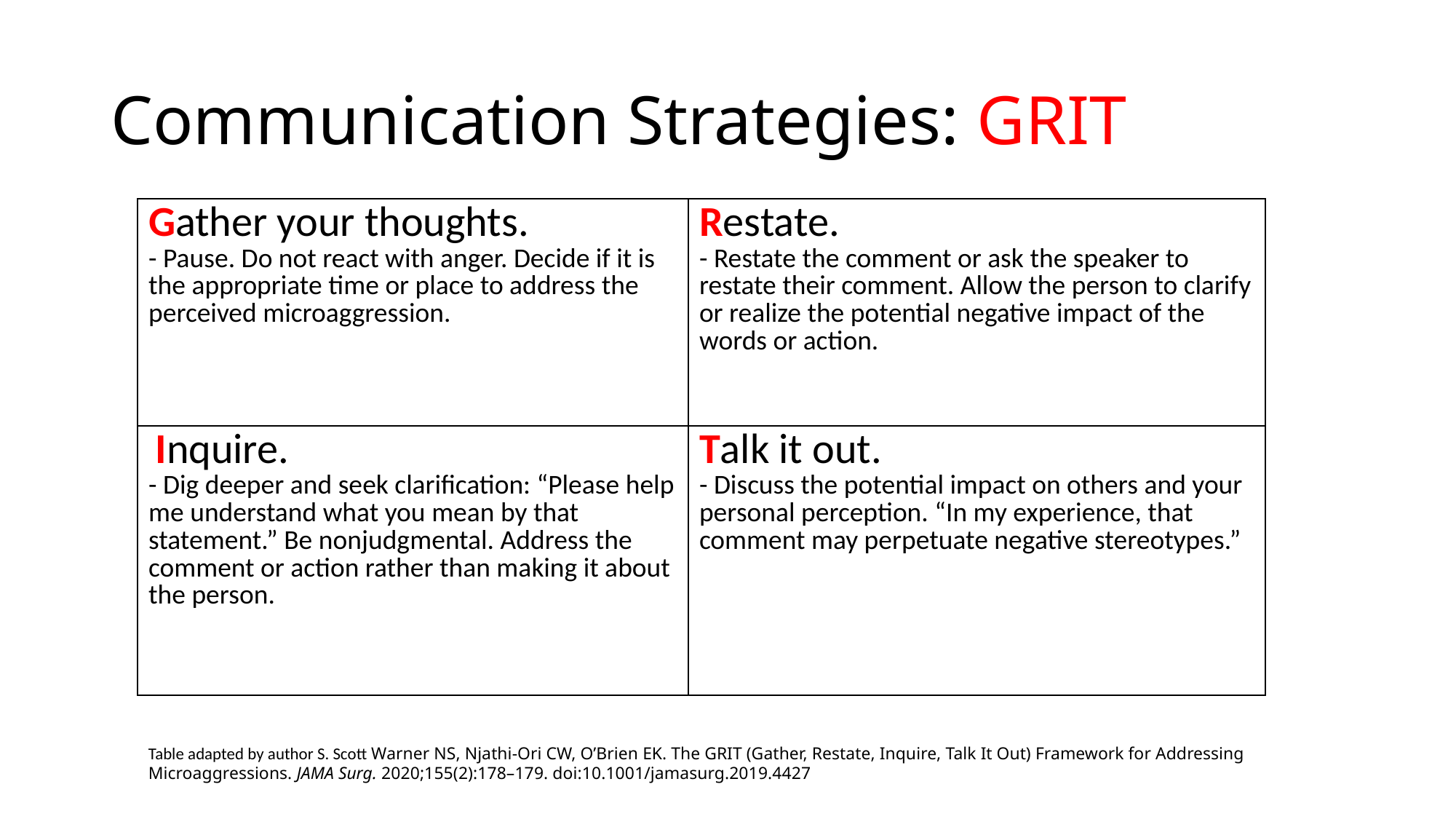

# Communication Strategies: GRIT
| Gather your thoughts. - Pause. Do not react with anger. Decide if it is the appropriate time or place to address the perceived microaggression. | Restate. - Restate the comment or ask the speaker to restate their comment. Allow the person to clarify or realize the potential negative impact of the words or action. |
| --- | --- |
| Inquire. - Dig deeper and seek clarification: “Please help me understand what you mean by that statement.” Be nonjudgmental. Address the comment or action rather than making it about the person. | Talk it out. - Discuss the potential impact on others and your personal perception. “In my experience, that comment may perpetuate negative stereotypes.” |
Table adapted by author S. Scott Warner NS, Njathi-Ori CW, O’Brien EK. The GRIT (Gather, Restate, Inquire, Talk It Out) Framework for Addressing Microaggressions. JAMA Surg. 2020;155(2):178–179. doi:10.1001/jamasurg.2019.4427

## Slide 24
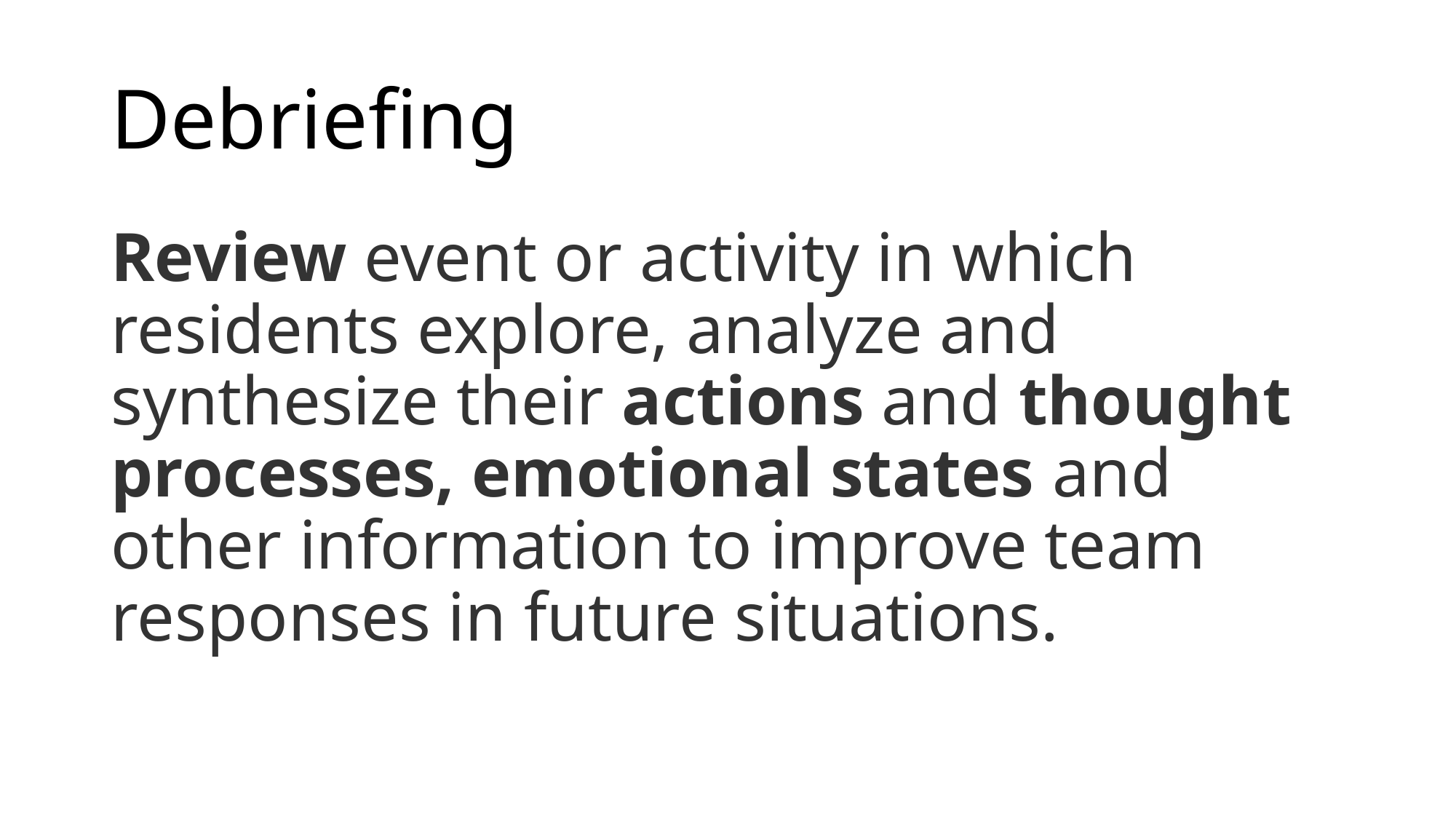

# Debriefing
Review event or activity in which residents explore, analyze and synthesize their actions and thought processes, emotional states and other information to improve team responses in future situations.

## Slide 25
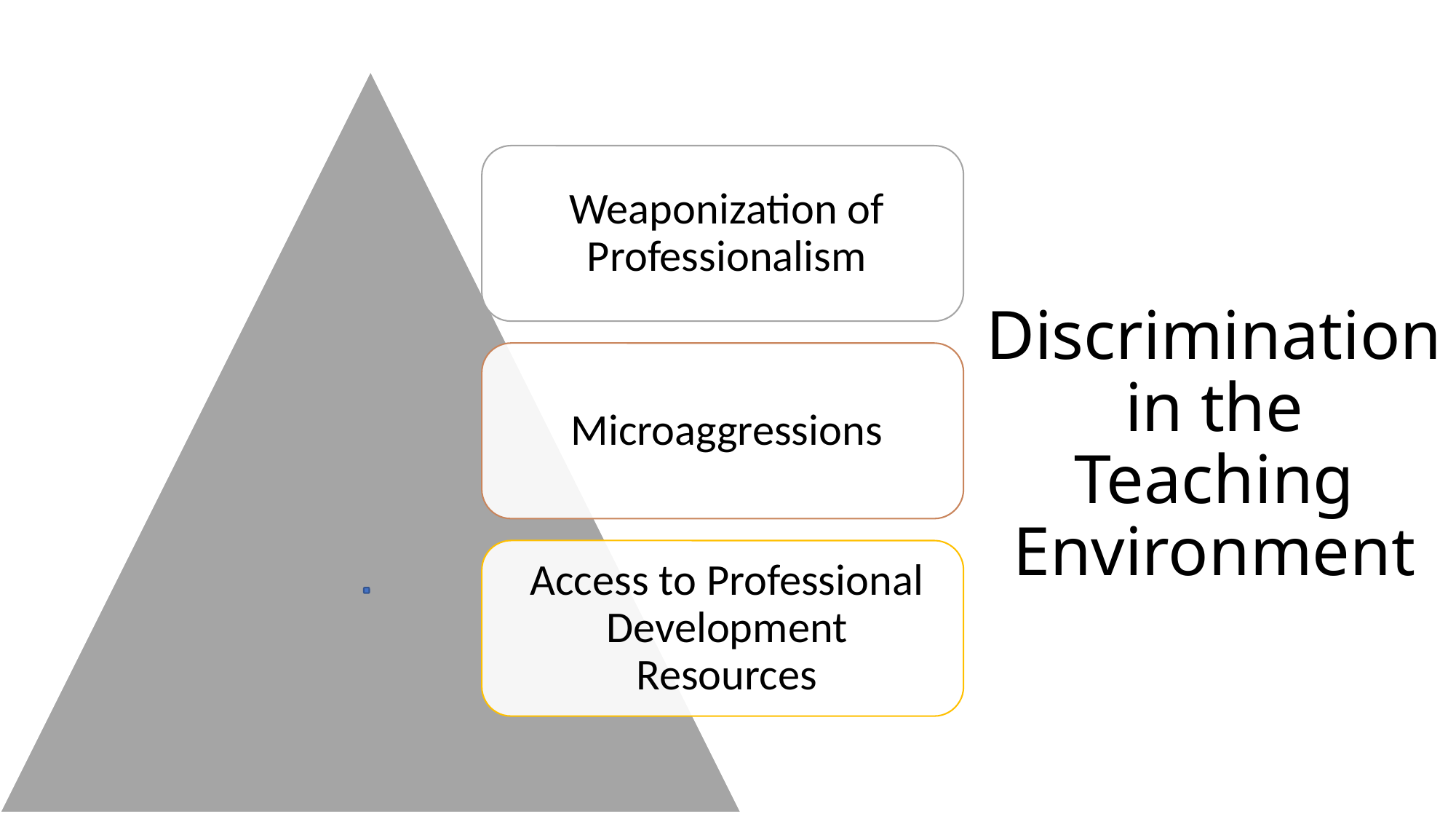

# Discrimination in the Teaching Environment

## Slide 26
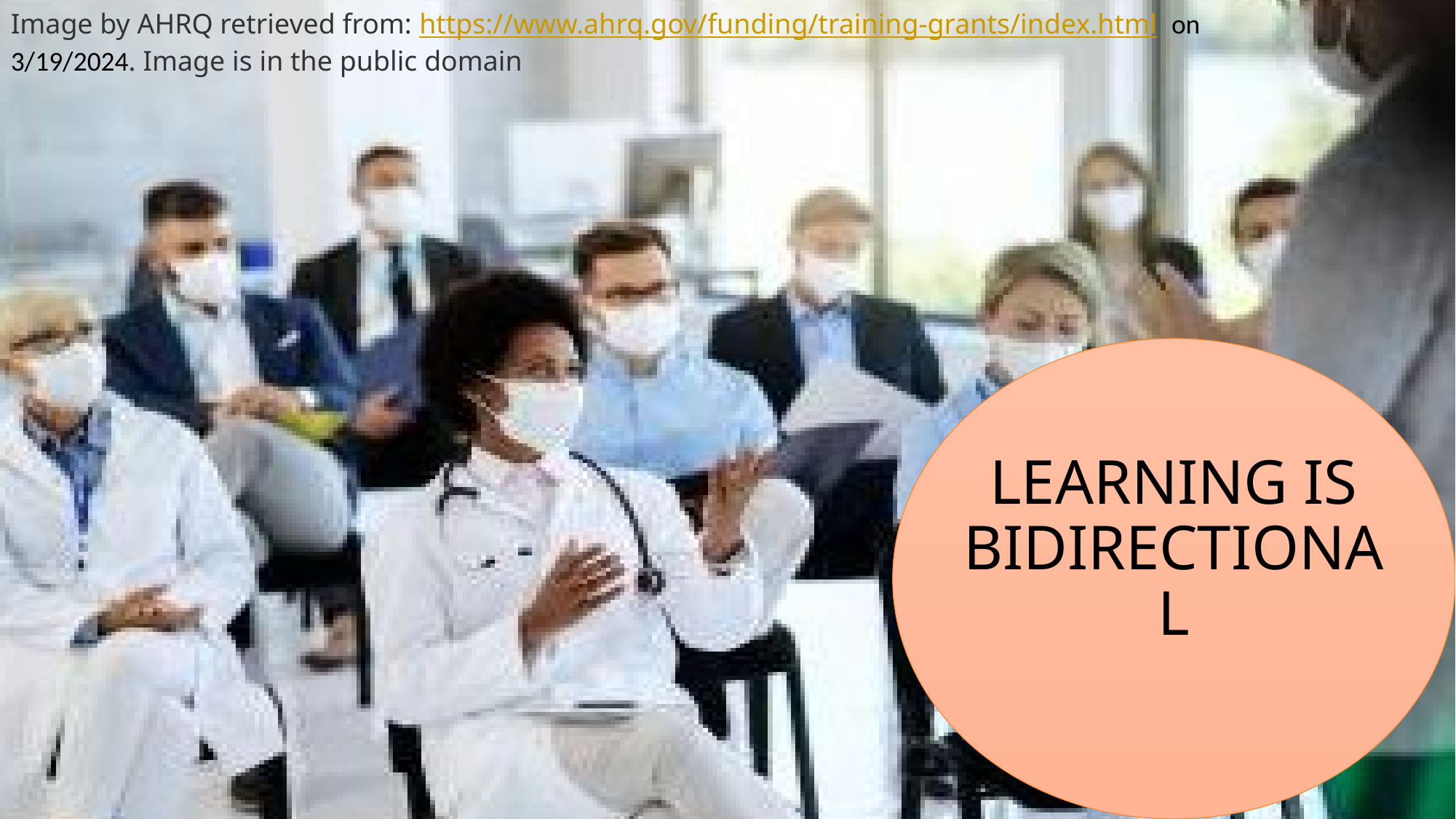

Image by AHRQ retrieved from: https://www.ahrq.gov/funding/training-grants/index.html on 3/19/2024. Image is in the public domain
LEARNING IS BIDIRECTIONAL

## Slide 27
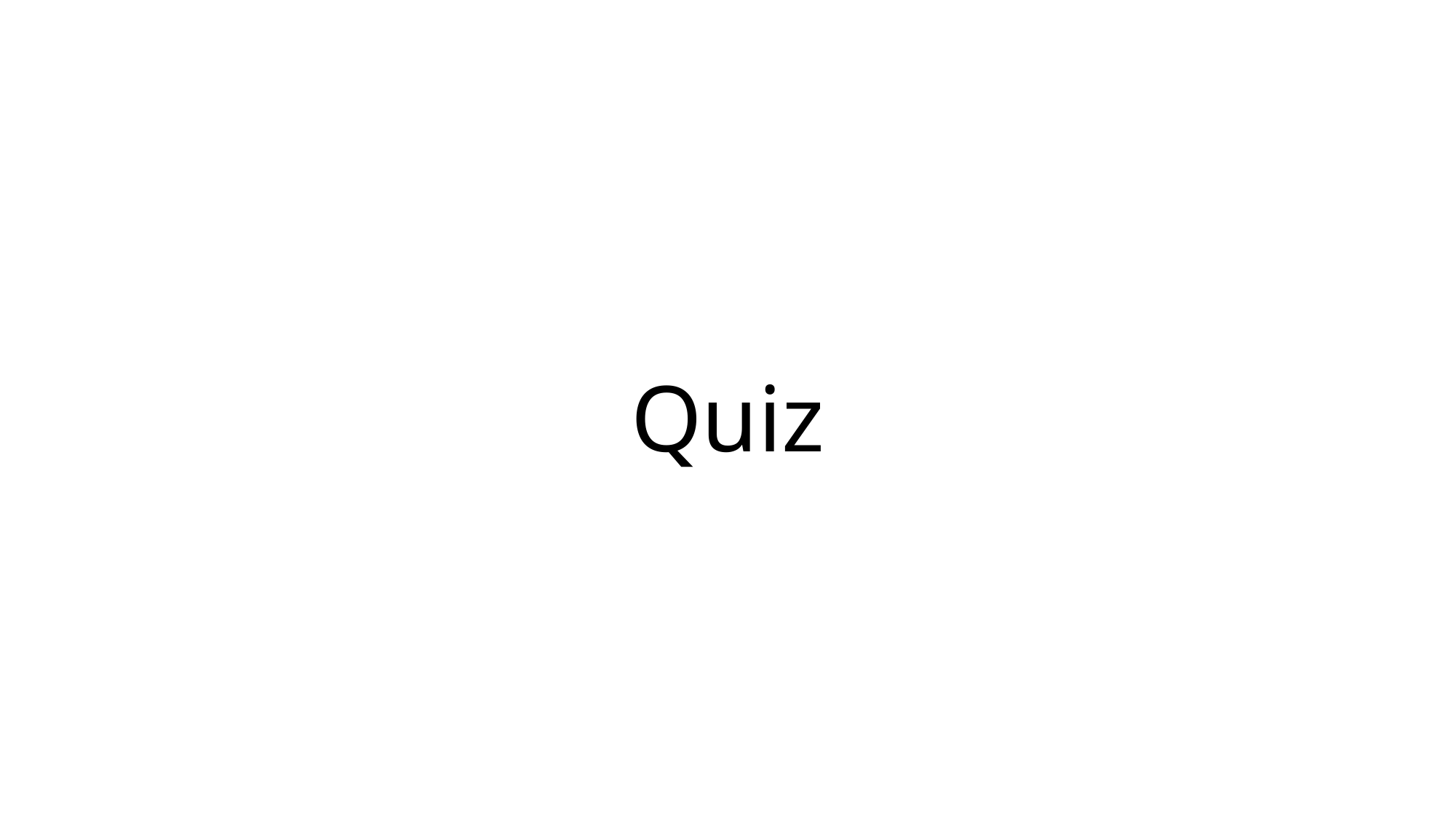

# Quiz

## Slide 28
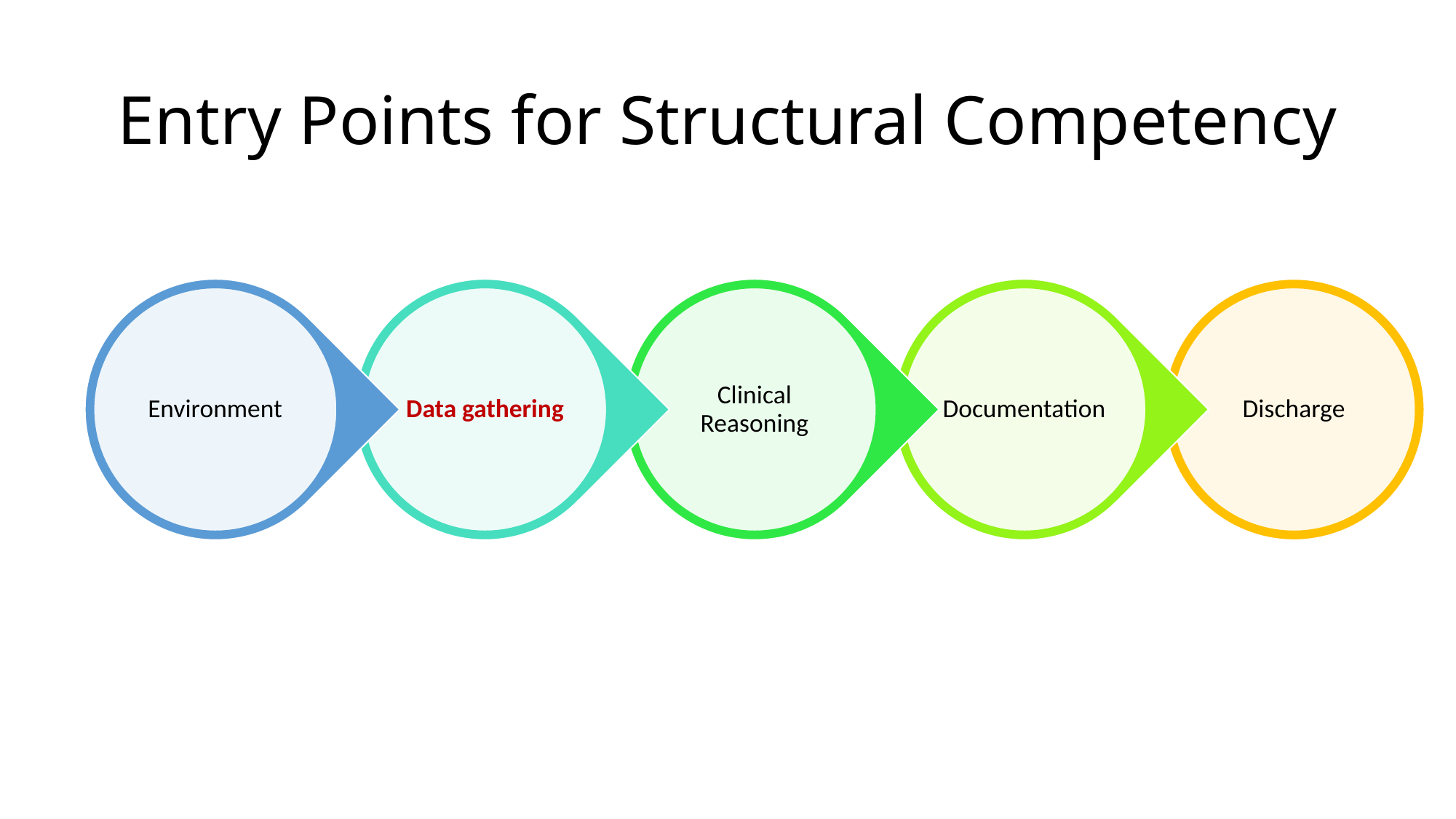

# Entry Points for Structural Competency

## Slide 29
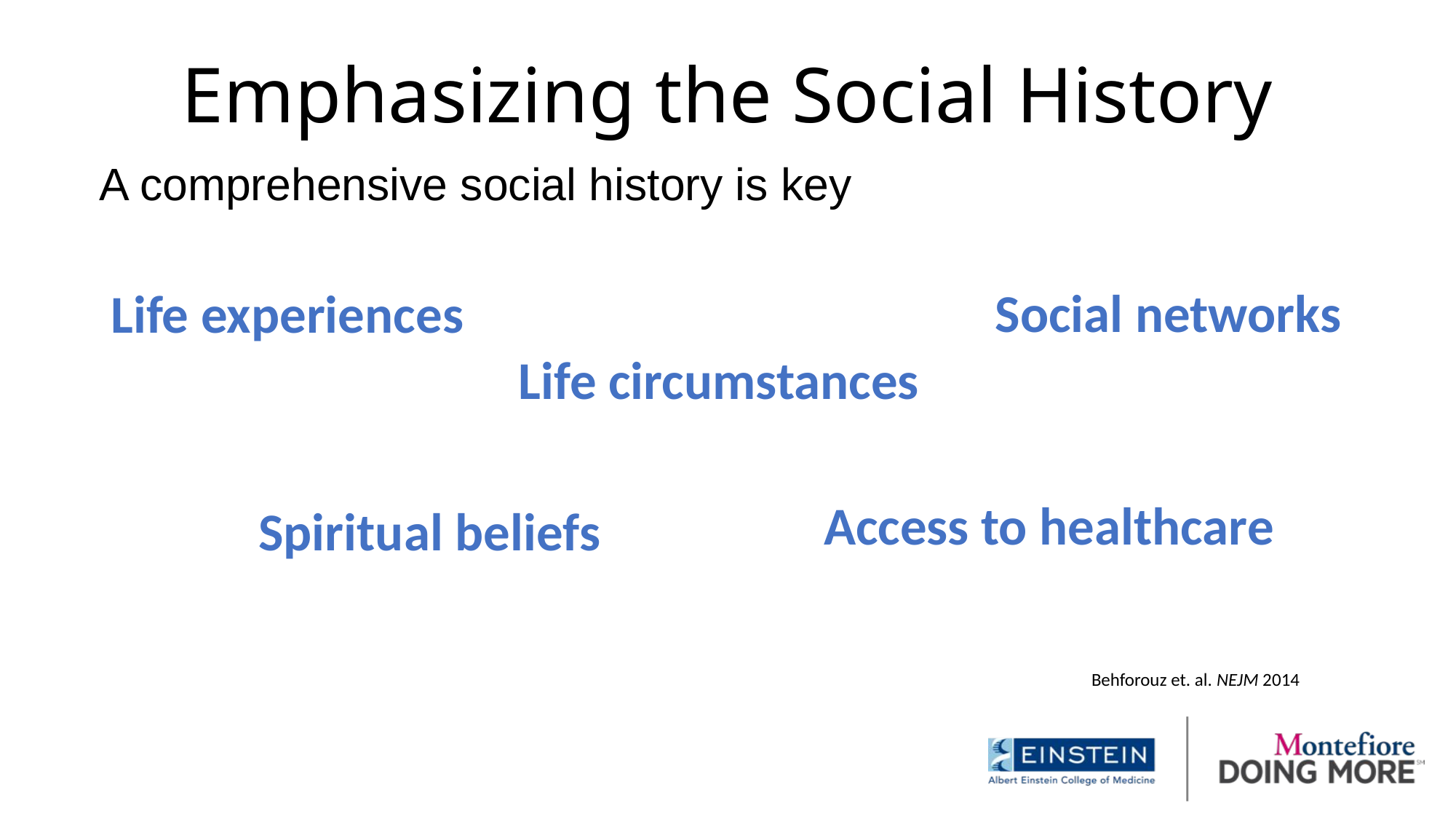

# Emphasizing the Social History
A comprehensive social history is key
Social networks
Life experiences
Life circumstances
Access to healthcare
Spiritual beliefs
Behforouz et. al. NEJM 2014

## Slide 30
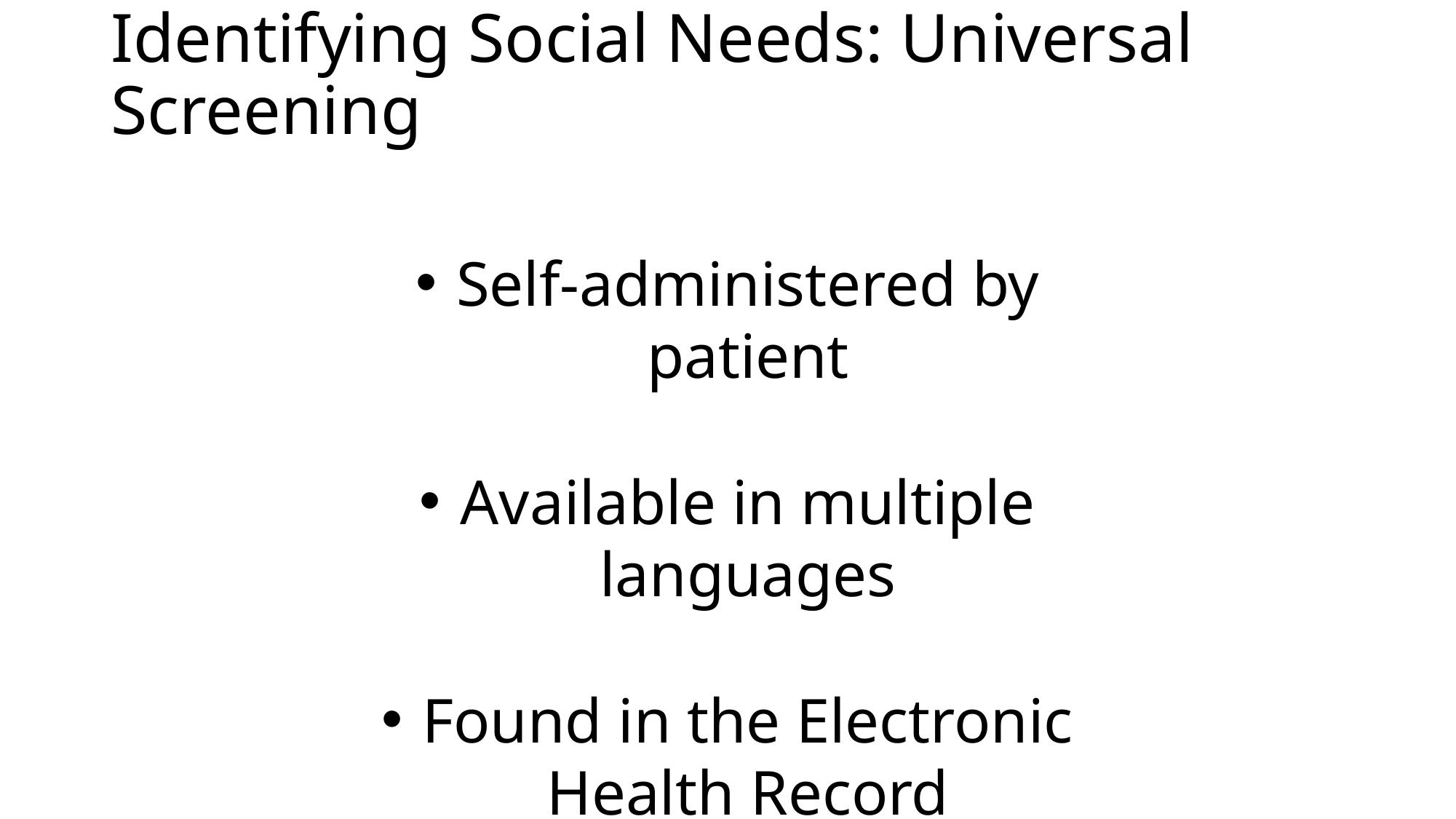

# Identifying Social Needs: Universal Screening
Self-administered by patient
Available in multiple languages
Found in the Electronic Health Record

## Slide 31
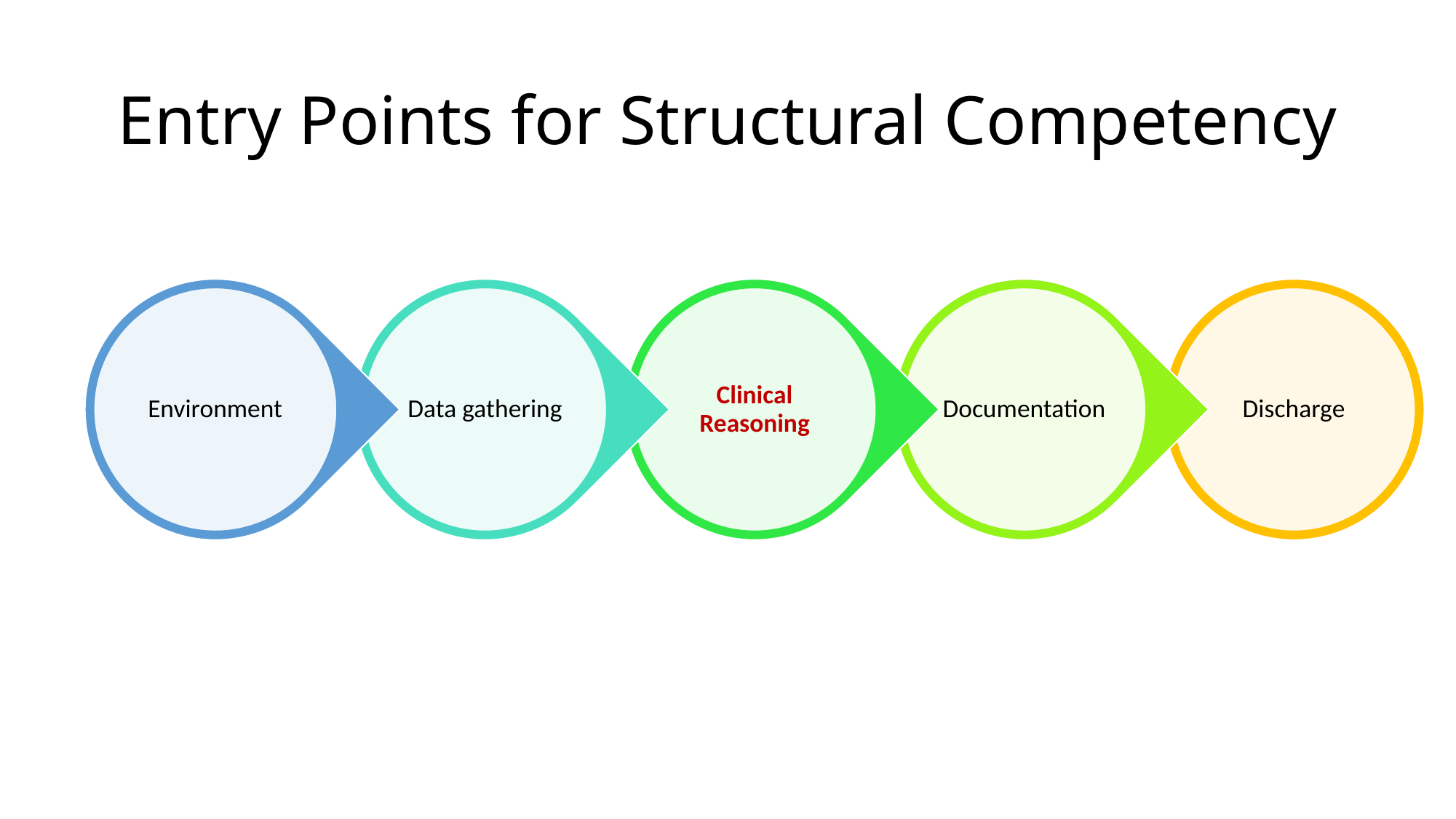

# Entry Points for Structural Competency

## Slide 32
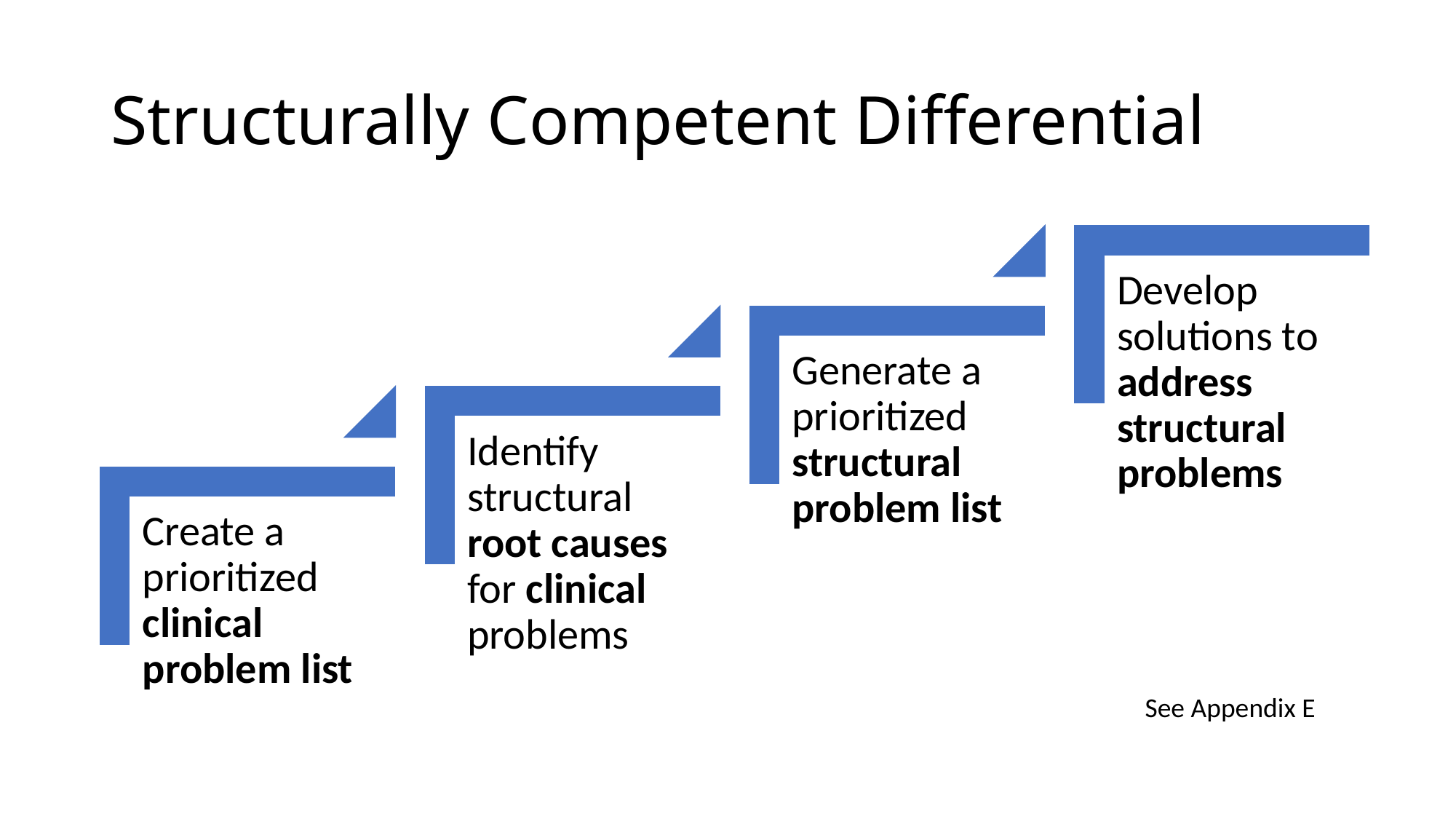

# Structurally Competent Differential
See Appendix E

## Slide 33
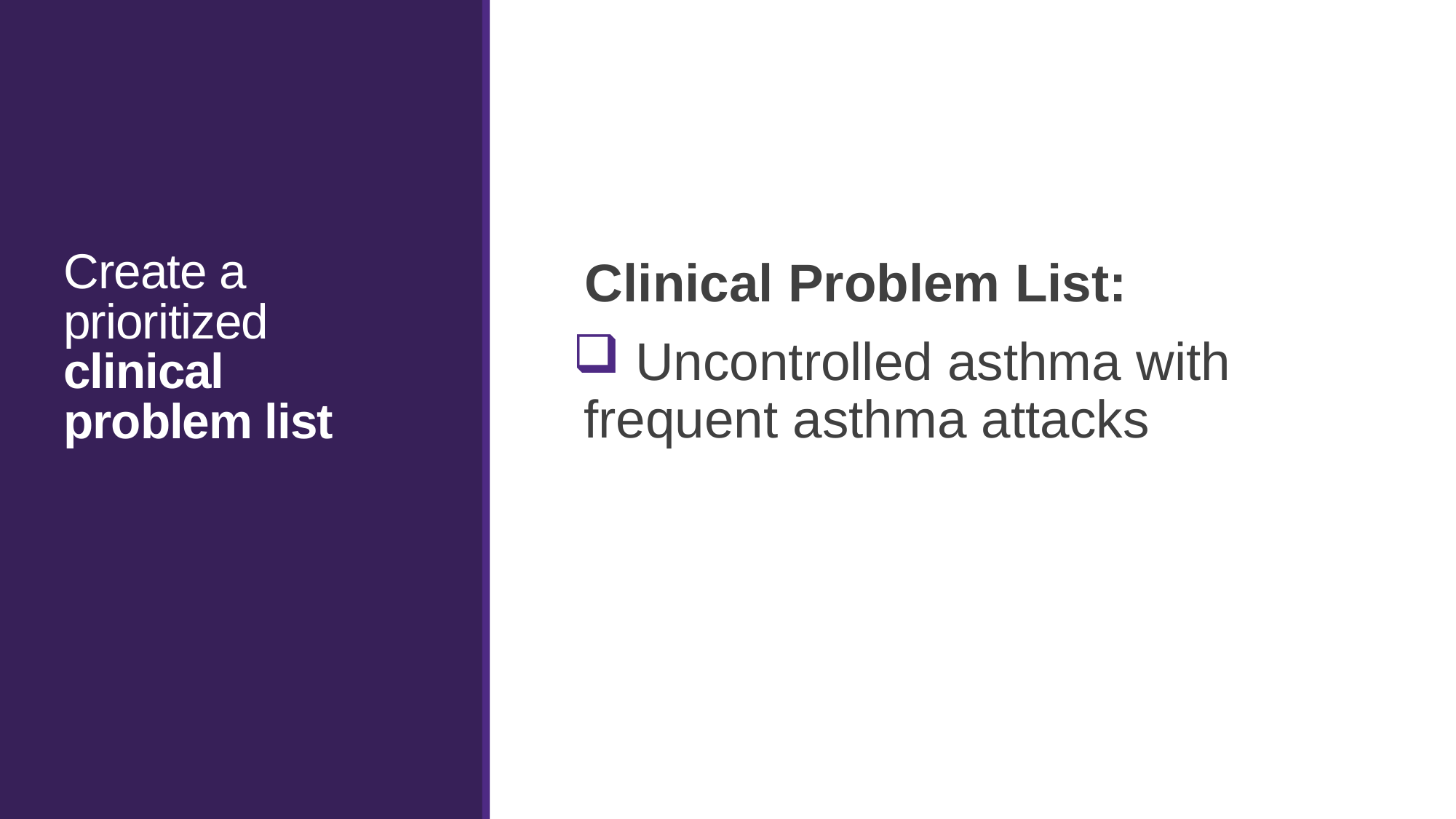

Clinical Problem List:
 Uncontrolled asthma with frequent asthma attacks
# Create a prioritized clinical problem list

## Slide 34
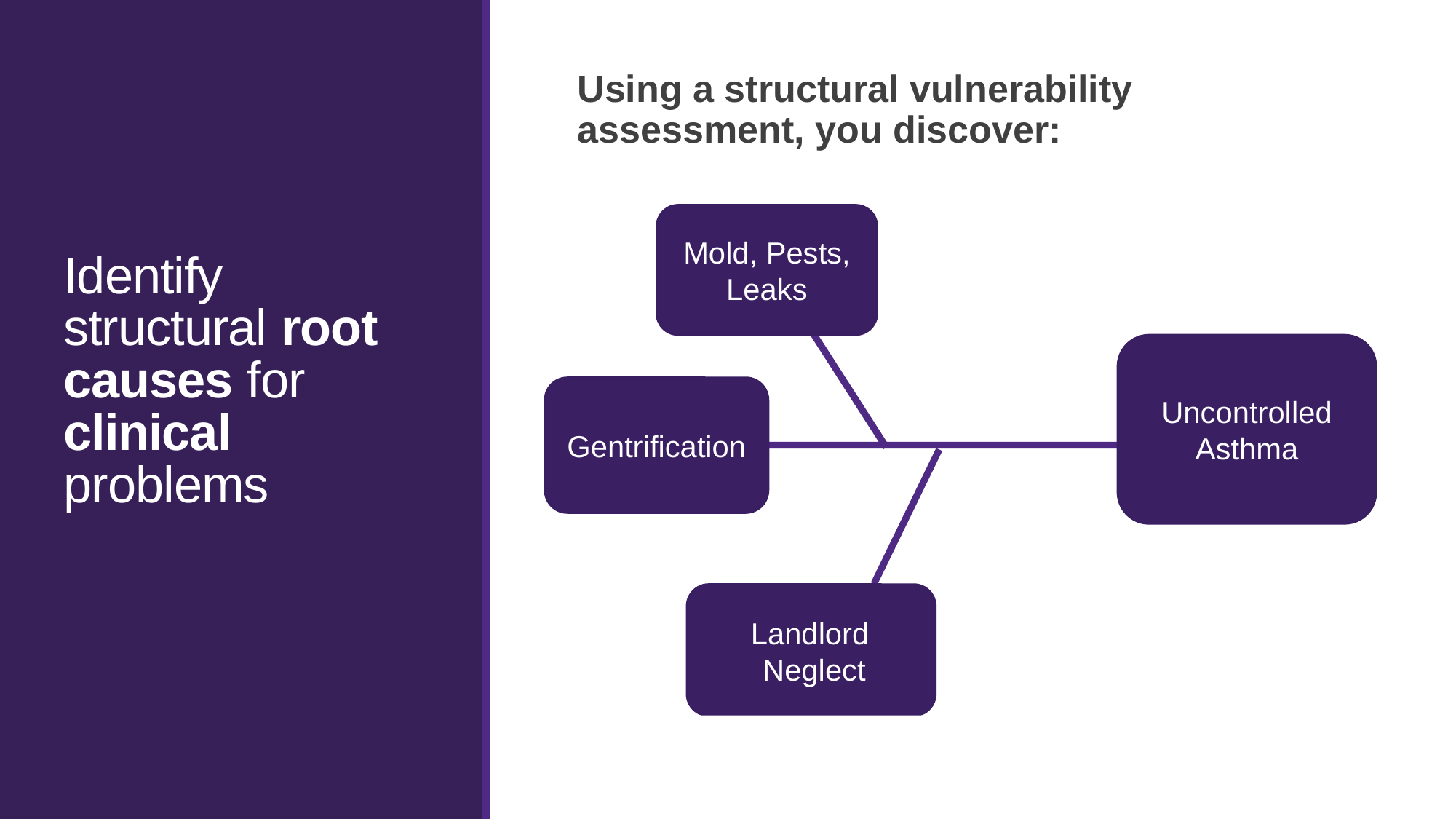

Using a structural vulnerability assessment, you discover:
Mold, Pests, Leaks
# Identify structural root causes for clinical problems
Uncontrolled Asthma
Gentrification
Landlord
Neglect

## Slide 35
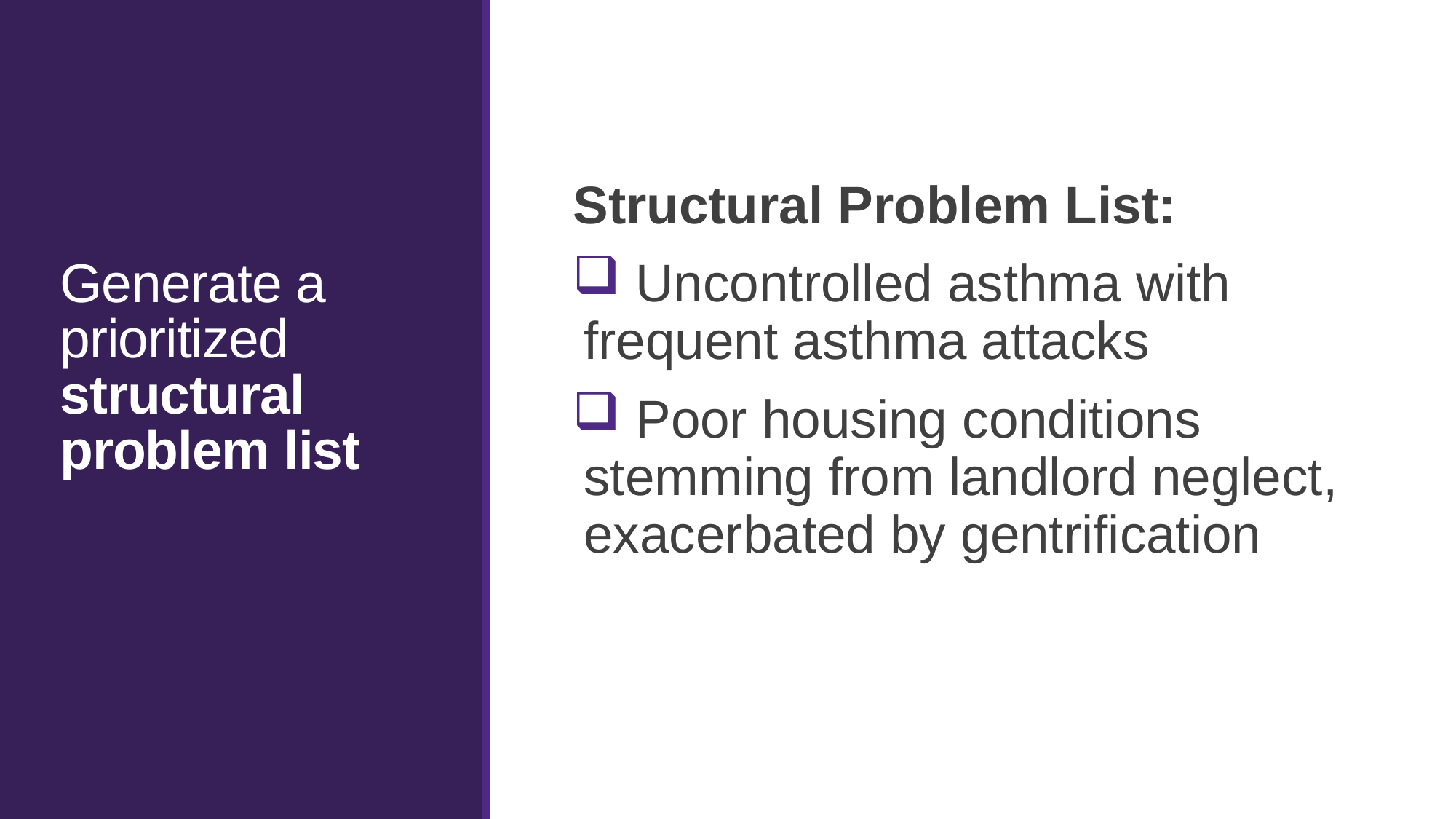

Structural Problem List:
 Uncontrolled asthma with frequent asthma attacks
 Poor housing conditions stemming from landlord neglect, exacerbated by gentrification
# Generate a prioritized structural problem list

## Slide 36
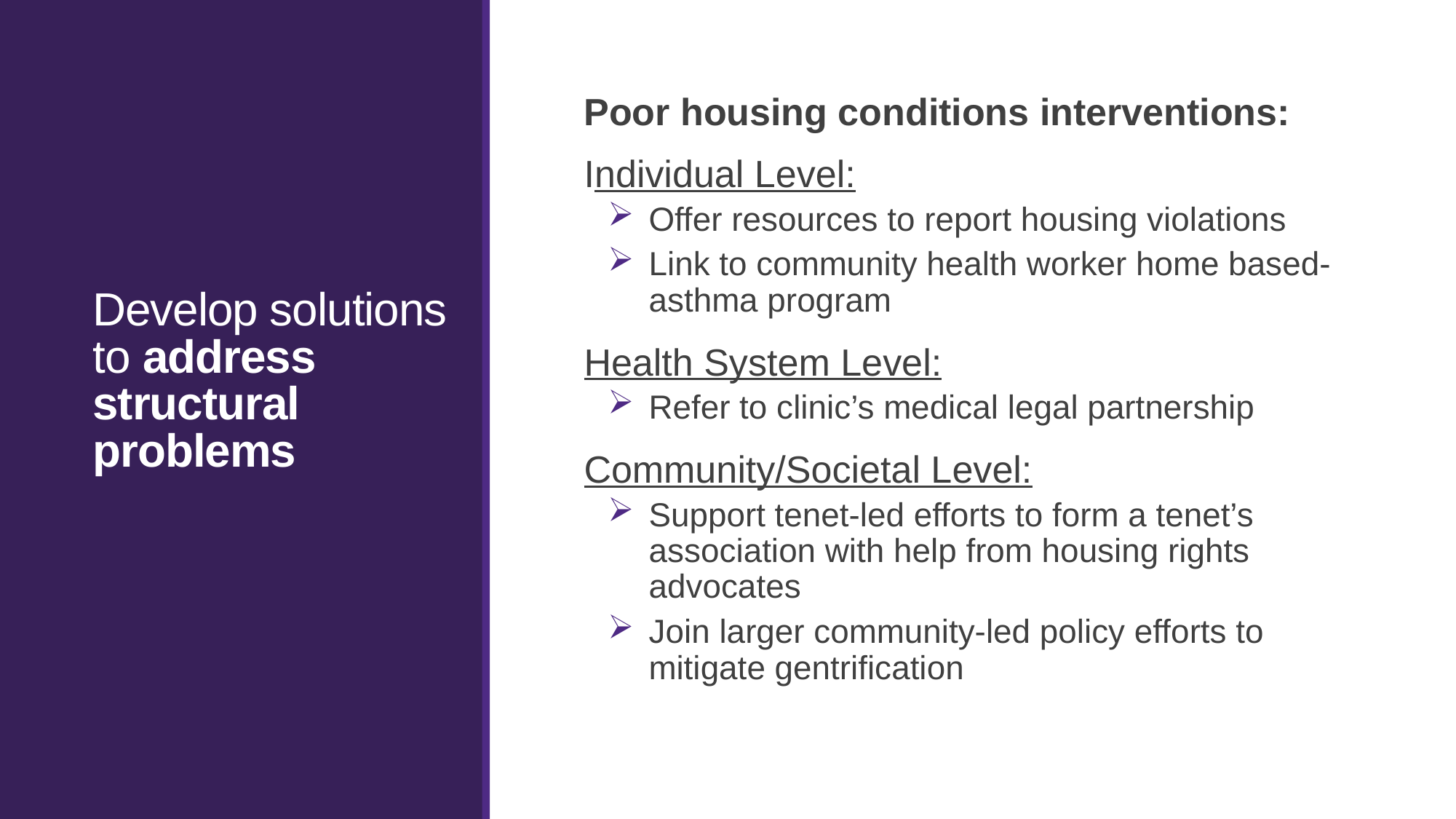

Poor housing conditions interventions:
Individual Level:
Offer resources to report housing violations
Link to community health worker home based-asthma program
Health System Level:
Refer to clinic’s medical legal partnership
Community/Societal Level:
Support tenet-led efforts to form a tenet’s association with help from housing rights advocates
Join larger community-led policy efforts to mitigate gentrification
# Develop solutions to address structural problems

## Slide 37
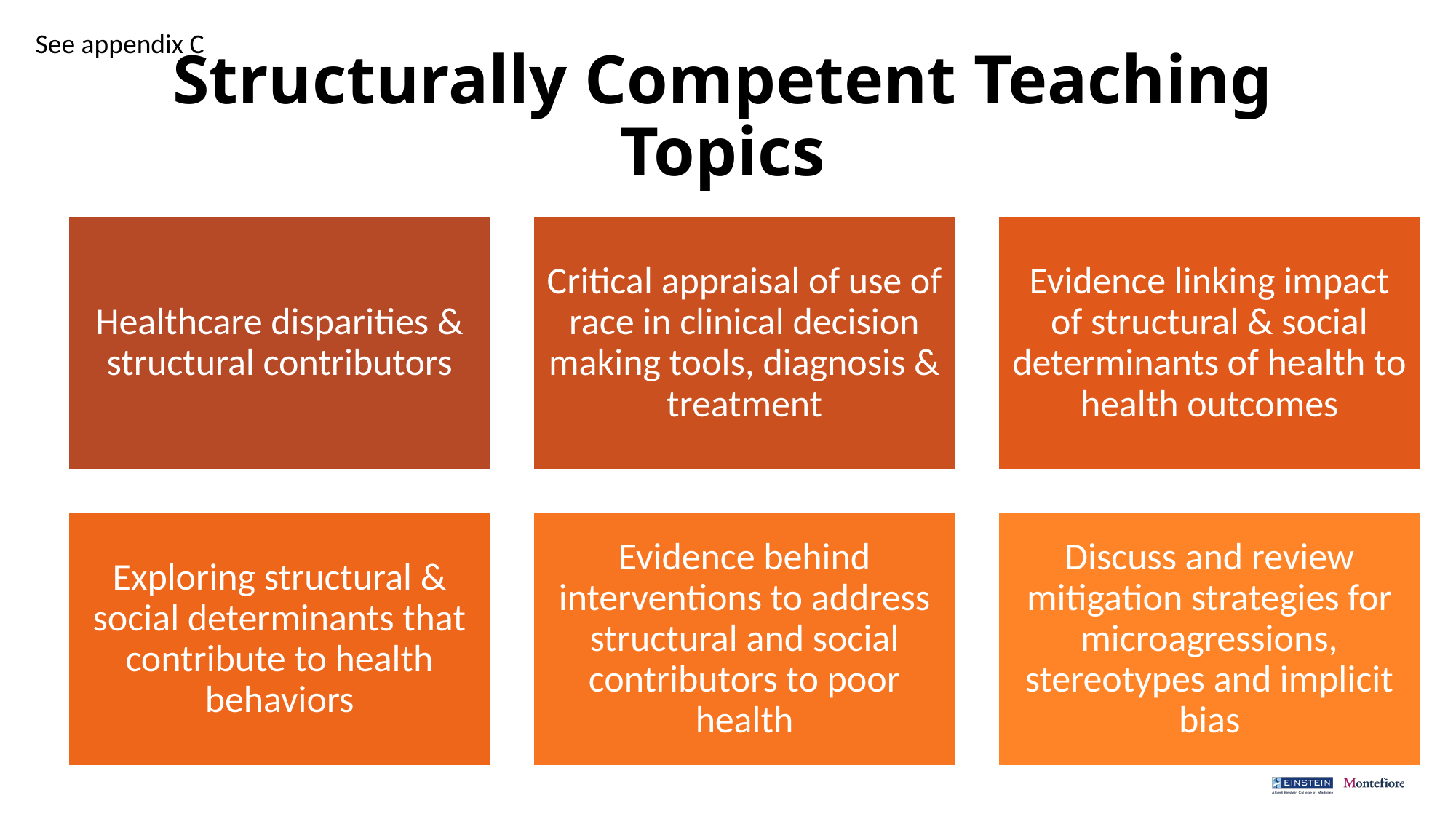

See appendix C
# Structurally Competent Teaching Topics

## Slide 38
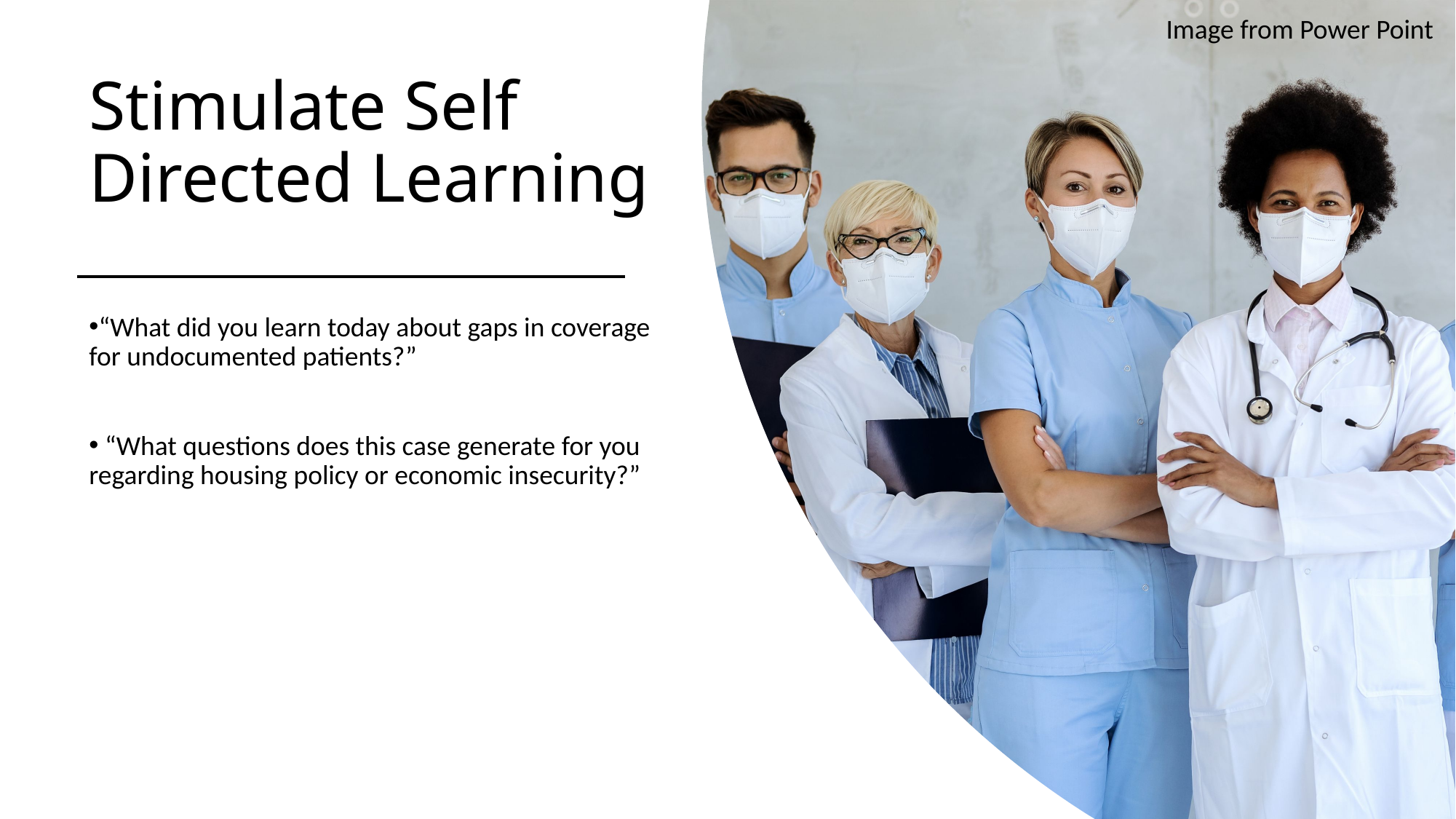

Image from Power Point
# Stimulate Self Directed Learning
“What did you learn today about gaps in coverage for undocumented patients?”
 “What questions does this case generate for you regarding housing policy or economic insecurity?”

## Slide 39
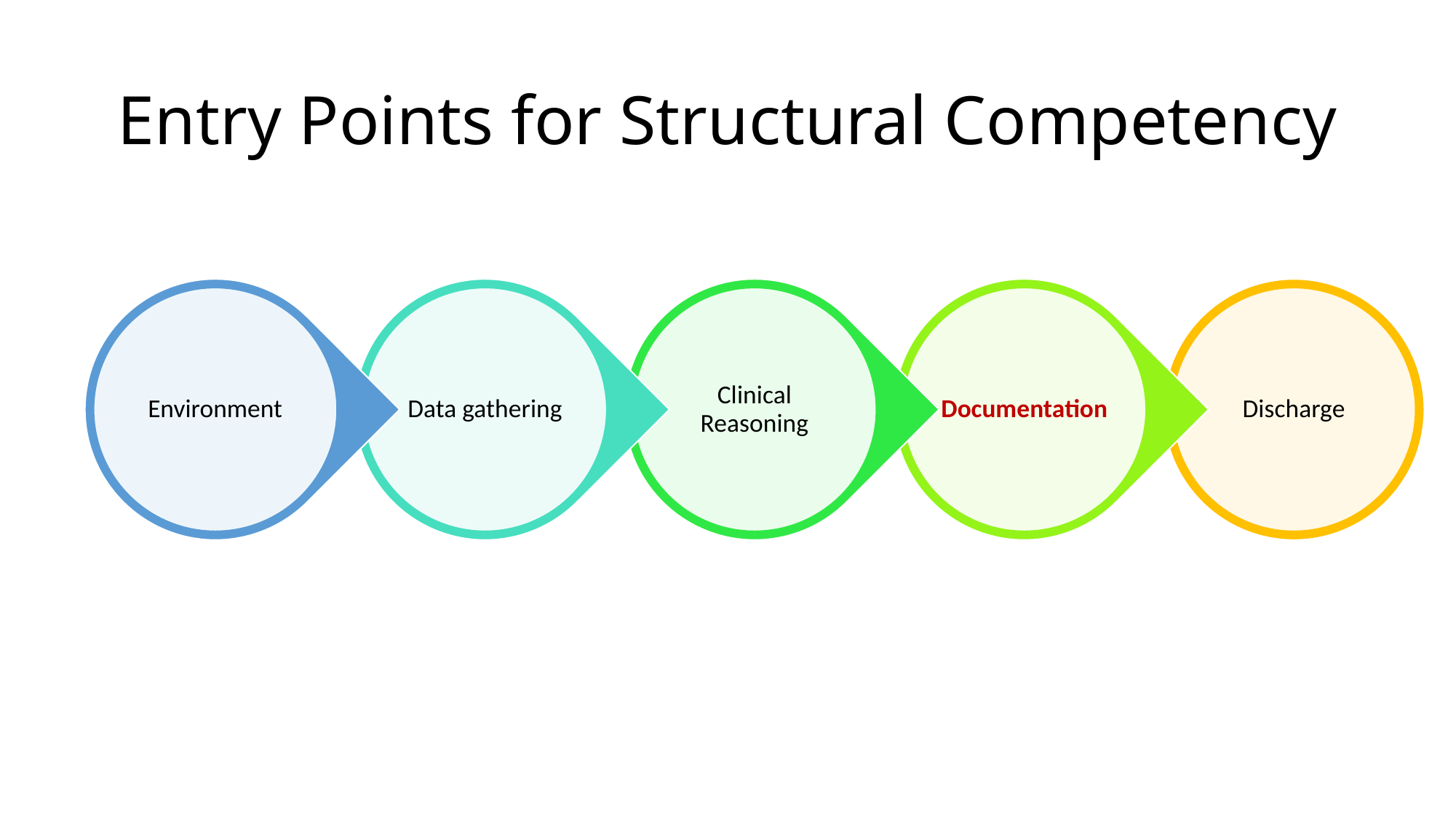

# Entry Points for Structural Competency

## Slide 40
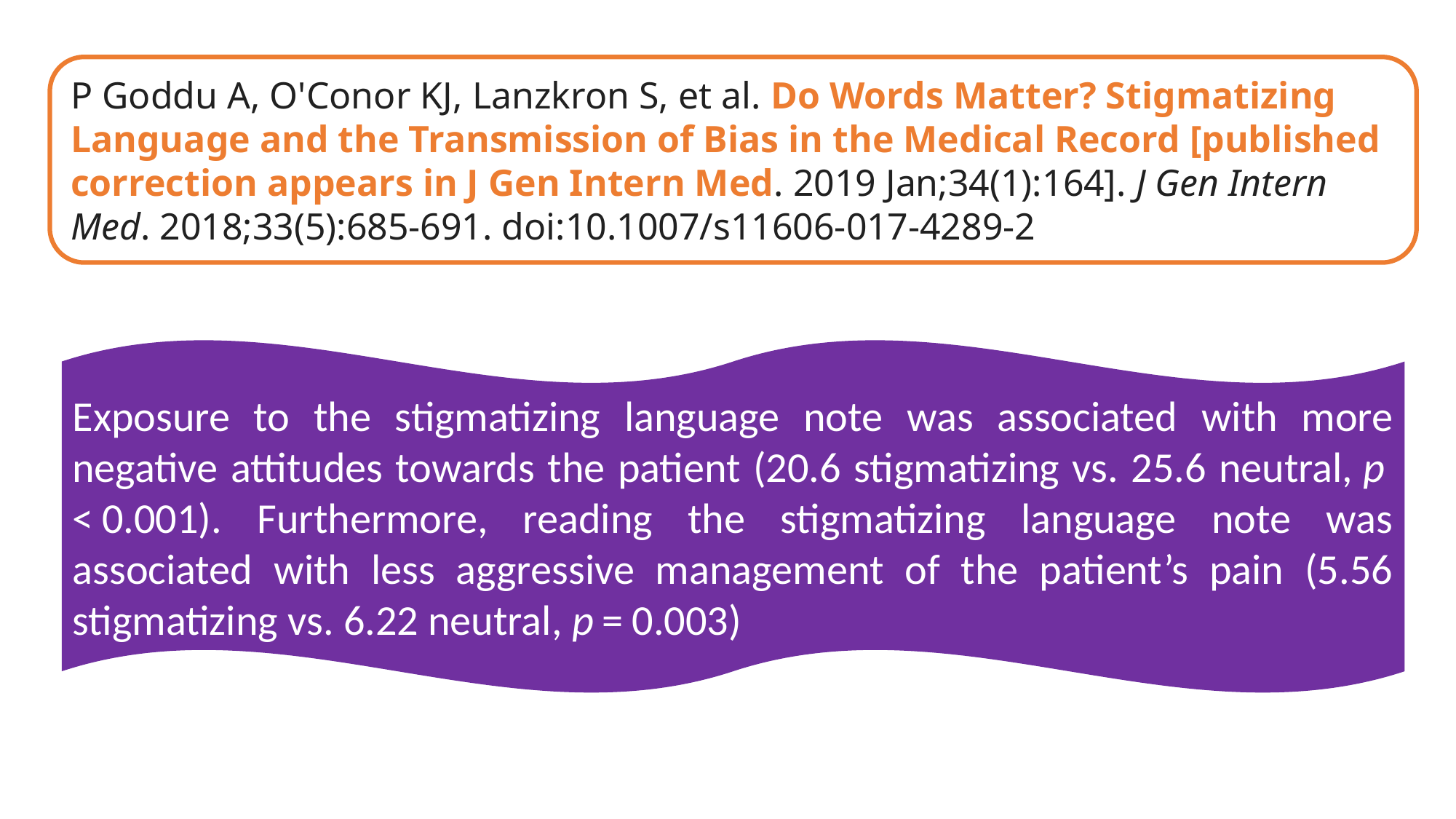

P Goddu A, O'Conor KJ, Lanzkron S, et al. Do Words Matter? Stigmatizing Language and the Transmission of Bias in the Medical Record [published correction appears in J Gen Intern Med. 2019 Jan;34(1):164]. J Gen Intern Med. 2018;33(5):685-691. doi:10.1007/s11606-017-4289-2
Exposure to the stigmatizing language note was associated with more negative attitudes towards the patient (20.6 stigmatizing vs. 25.6 neutral, p < 0.001). Furthermore, reading the stigmatizing language note was associated with less aggressive management of the patient’s pain (5.56 stigmatizing vs. 6.22 neutral, p = 0.003)

## Slide 41
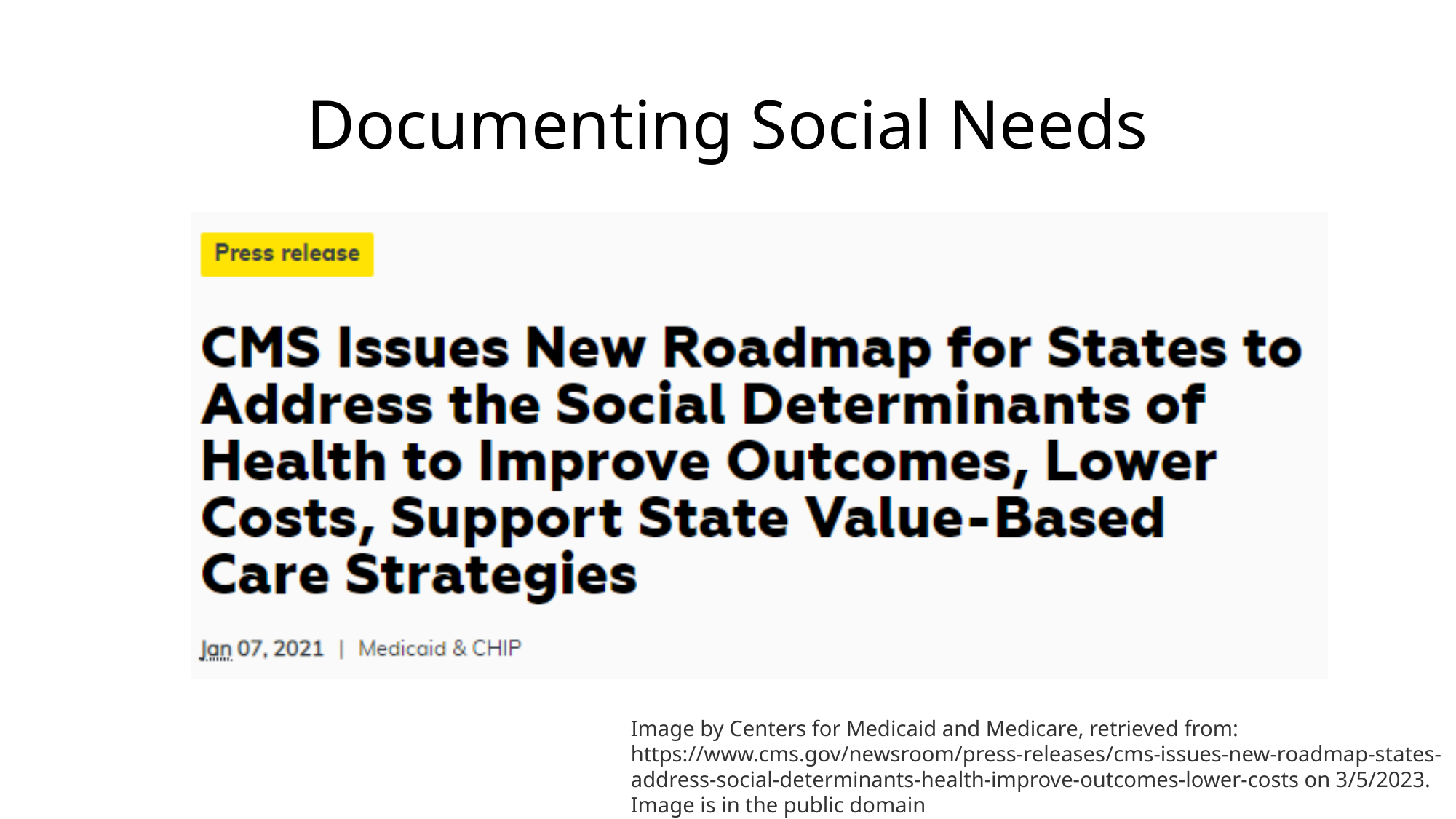

# Documenting Social Needs
Image by Centers for Medicaid and Medicare, retrieved from: https://www.cms.gov/newsroom/press-releases/cms-issues-new-roadmap-states-address-social-determinants-health-improve-outcomes-lower-costs on 3/5/2023. Image is in the public domain

## Slide 42
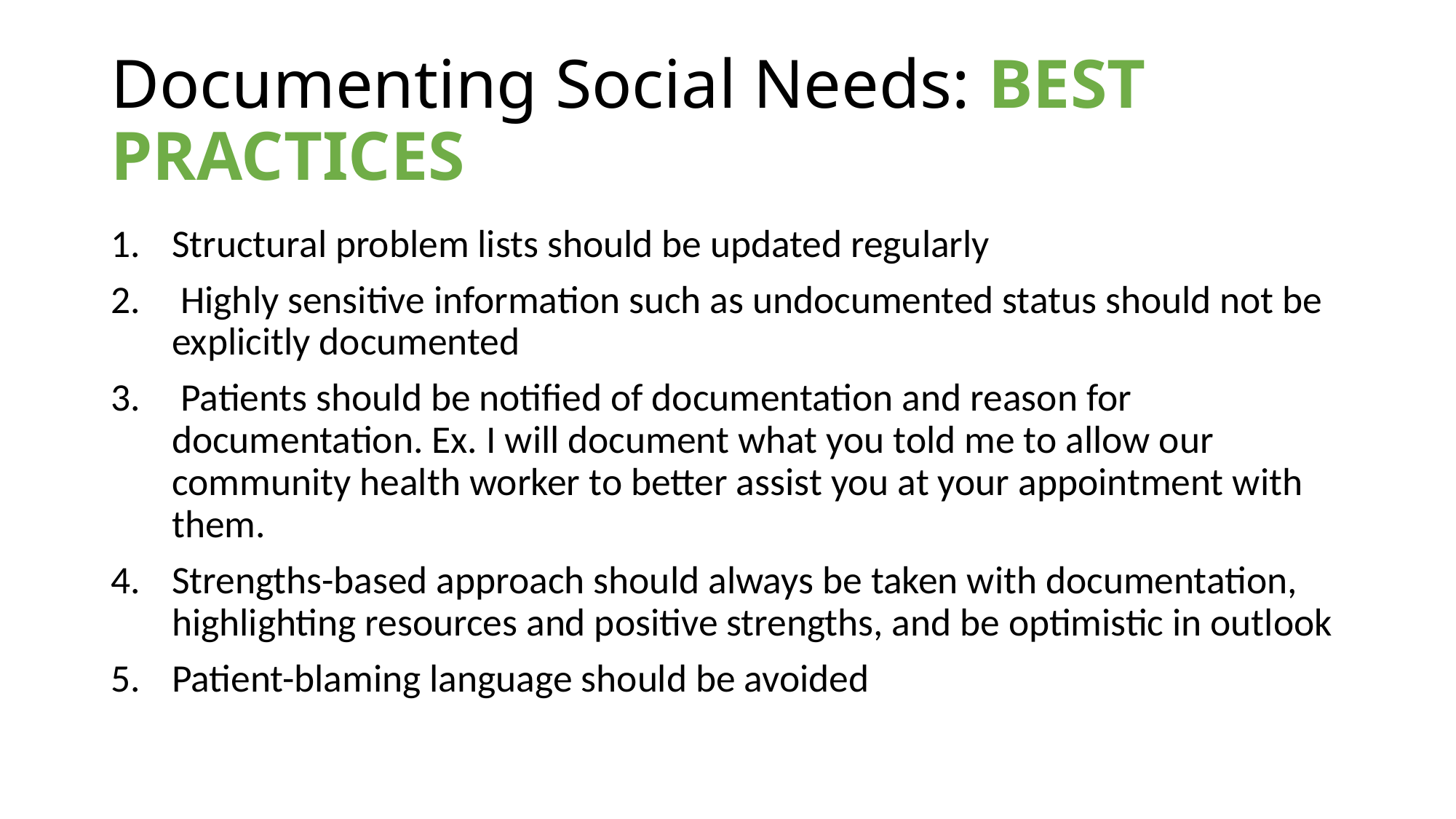

# Documenting Social Needs: BEST PRACTICES
Structural problem lists should be updated regularly
 Highly sensitive information such as undocumented status should not be explicitly documented
 Patients should be notified of documentation and reason for documentation. Ex. I will document what you told me to allow our community health worker to better assist you at your appointment with them.
Strengths-based approach should always be taken with documentation, highlighting resources and positive strengths, and be optimistic in outlook
Patient-blaming language should be avoided

## Slide 43
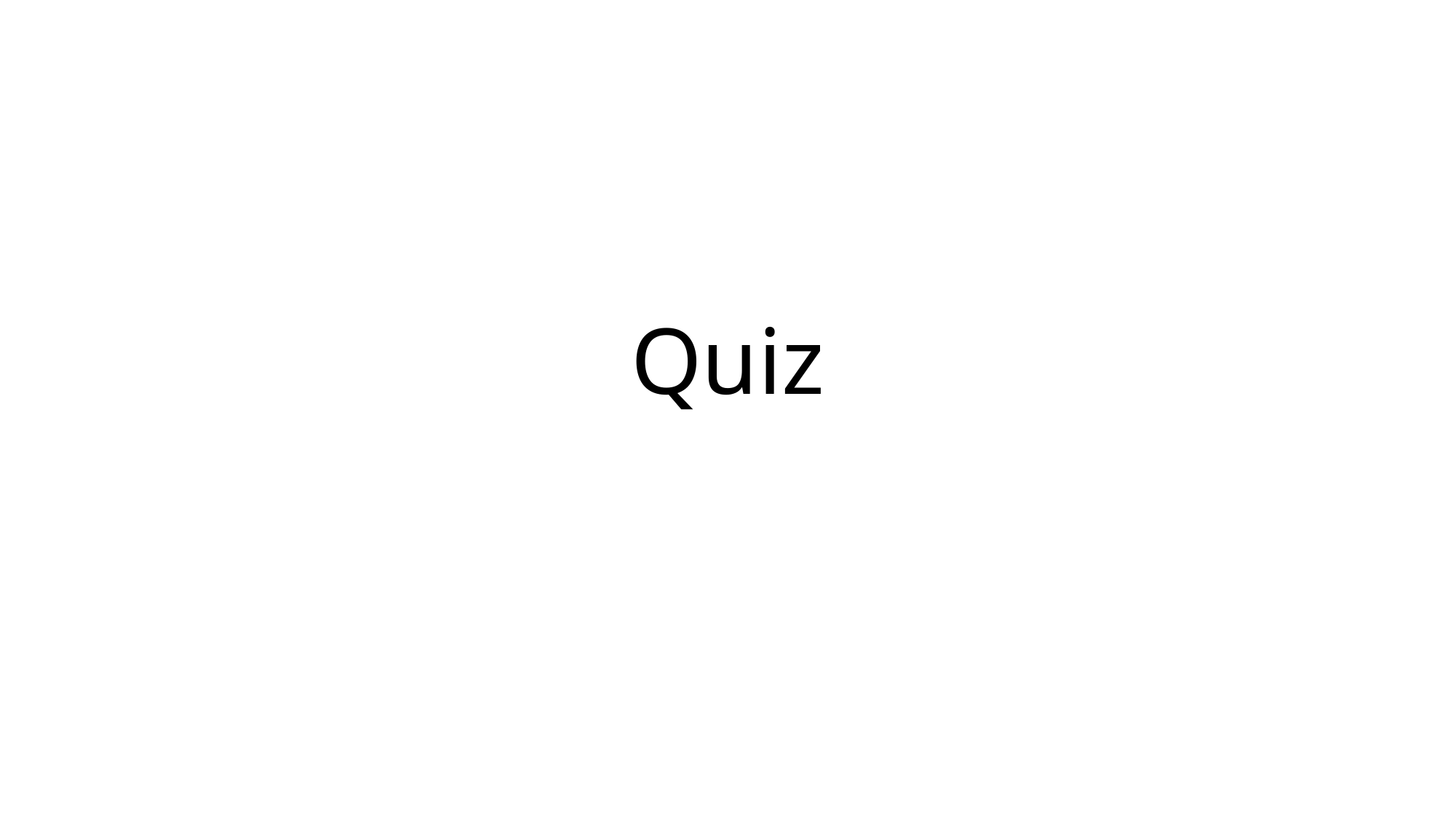

# Quiz

## Slide 44
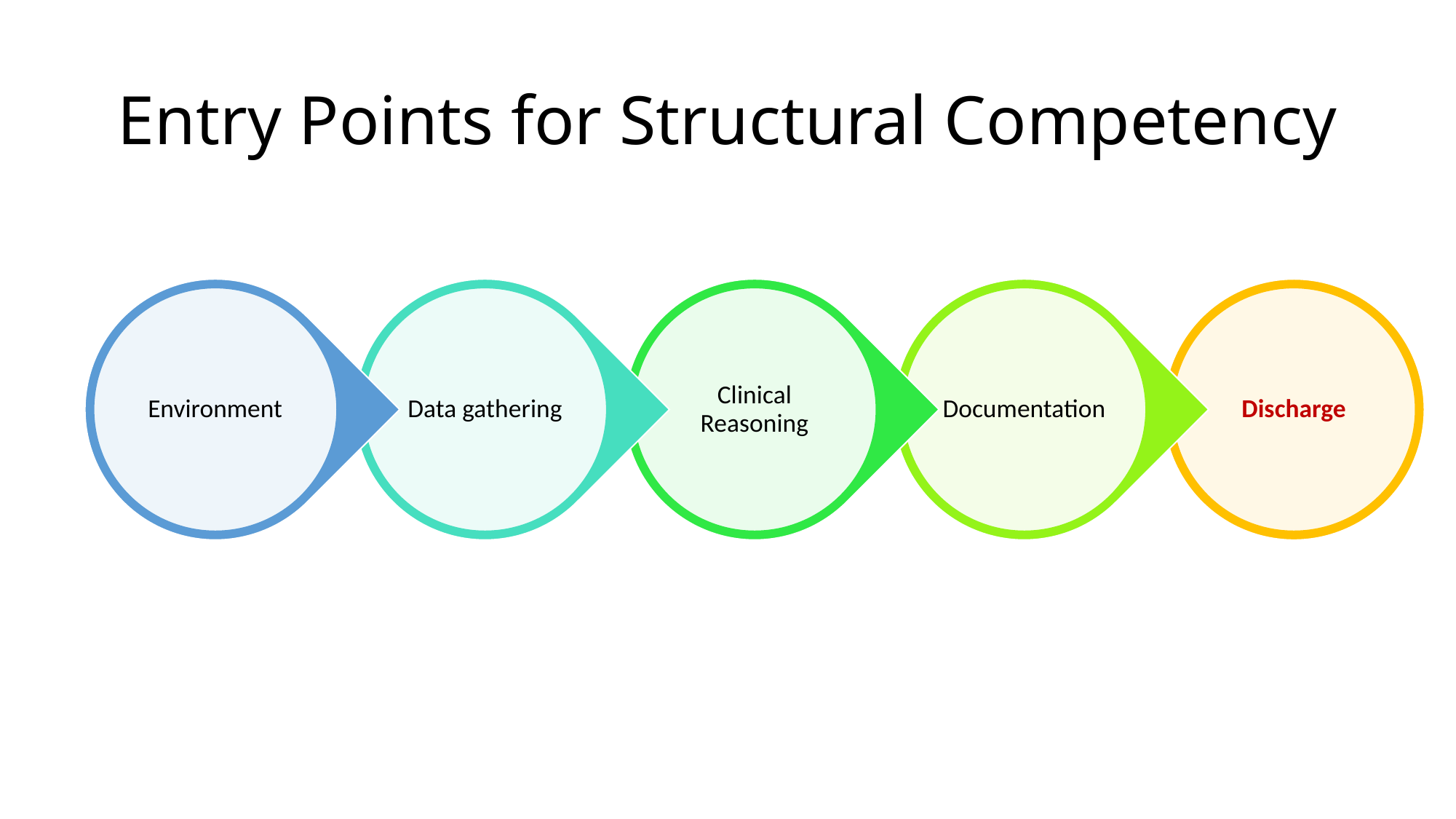

# Entry Points for Structural Competency

## Slide 45
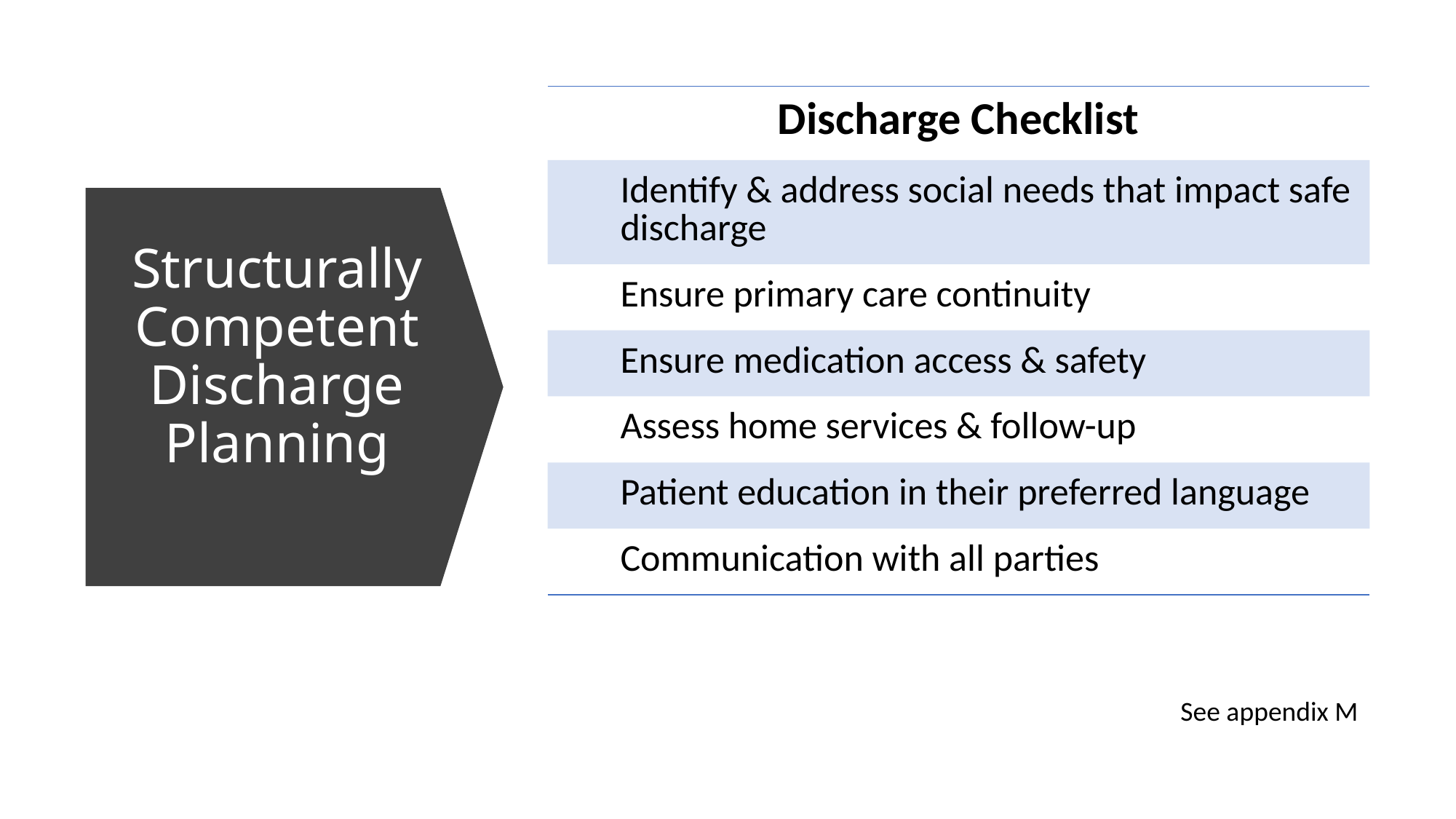

| Discharge Checklist |
| --- |
| Identify & address social needs that impact safe discharge |
| Ensure primary care continuity |
| Ensure medication access & safety |
| Assess home services & follow-up |
| Patient education in their preferred language |
| Communication with all parties |
# Structurally Competent Discharge Planning
See appendix M

## Slide 46
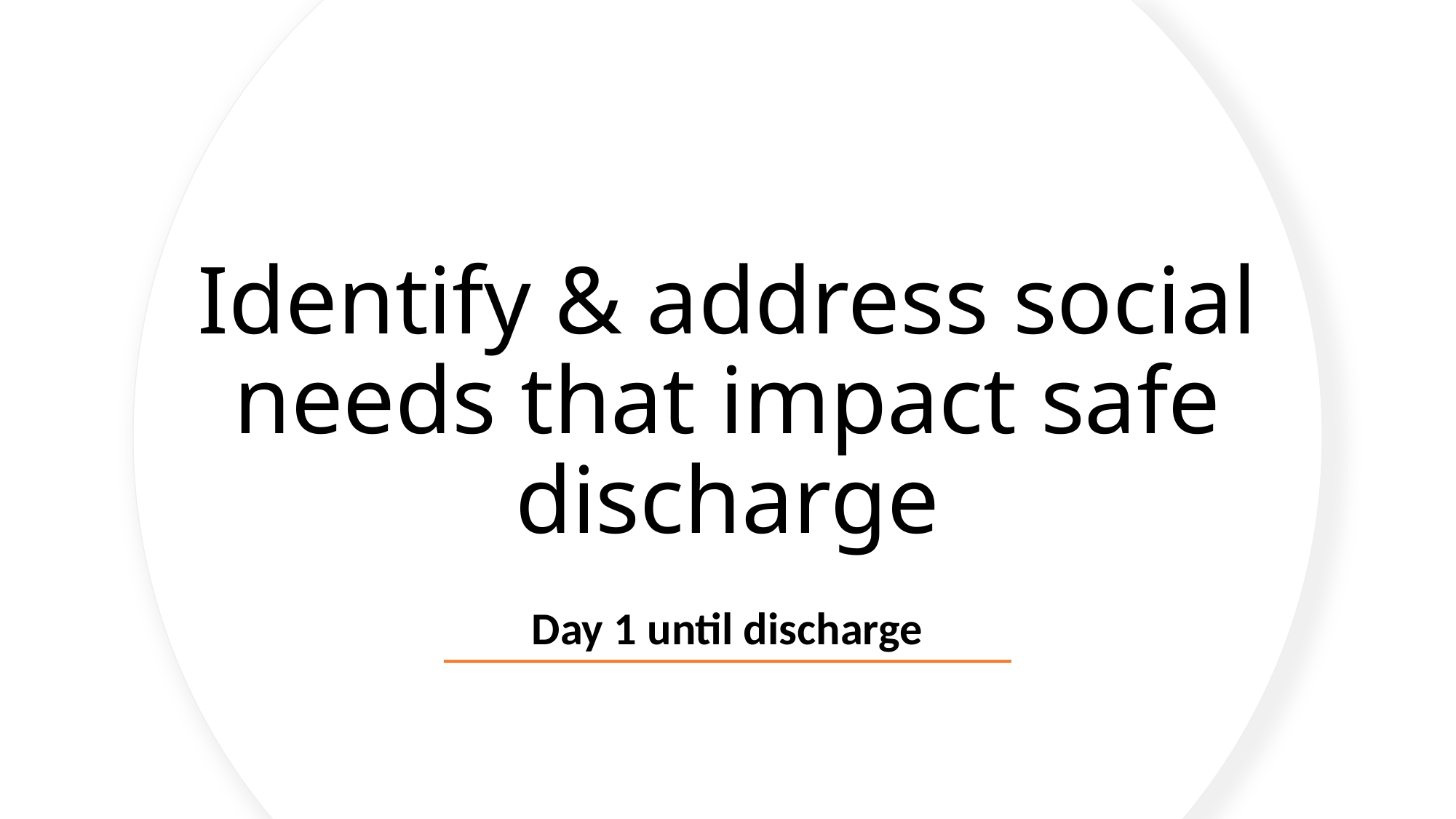

# Identify & address social needs that impact safe discharge
Day 1 until discharge

## Slide 47
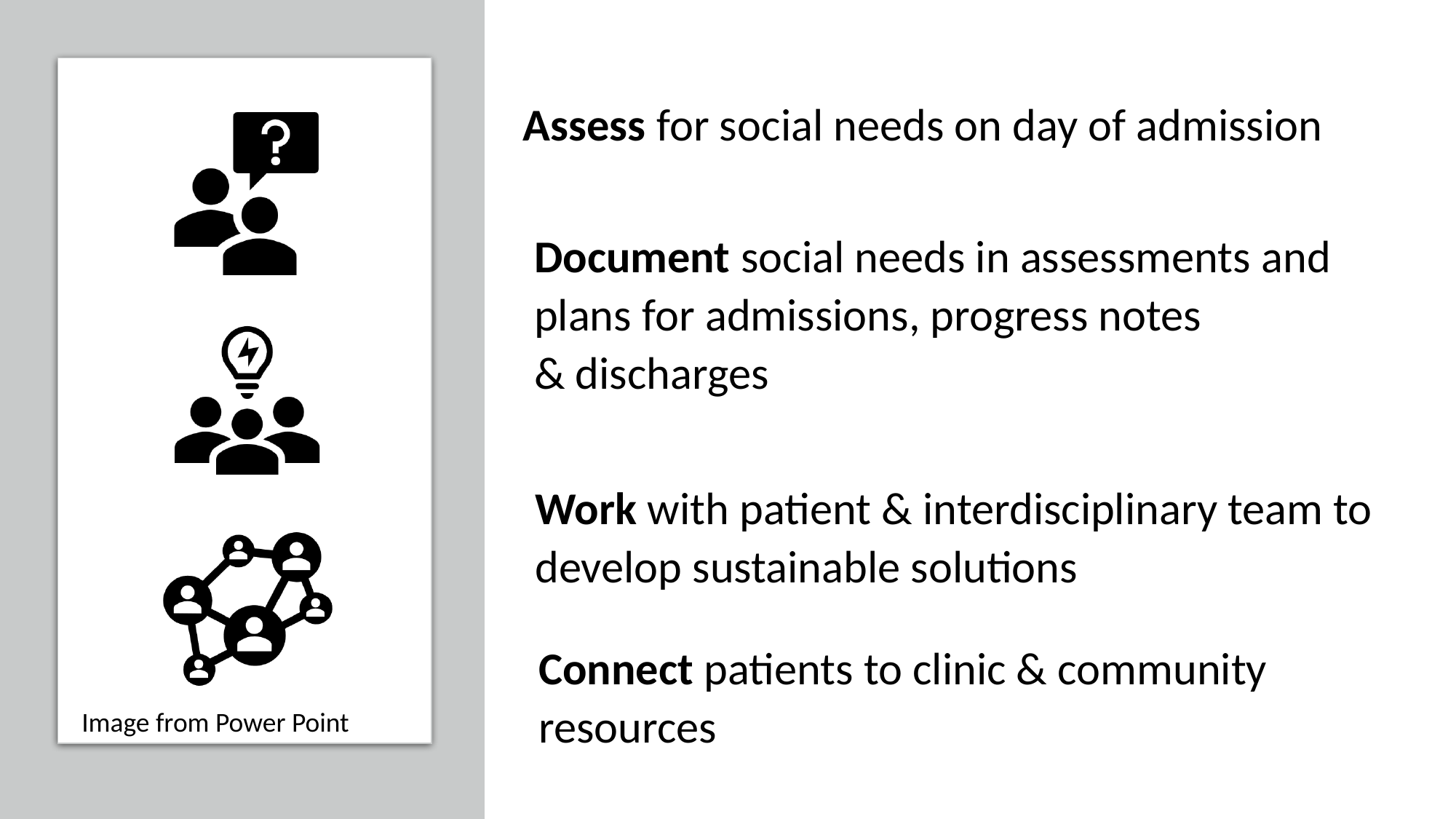

Assess for social needs on day of admission
Document social needs in assessments and
plans for admissions, progress notes
& discharges
Work with patient & interdisciplinary team to
develop sustainable solutions
Connect patients to clinic & community
resources
Image from Power Point

## Slide 48
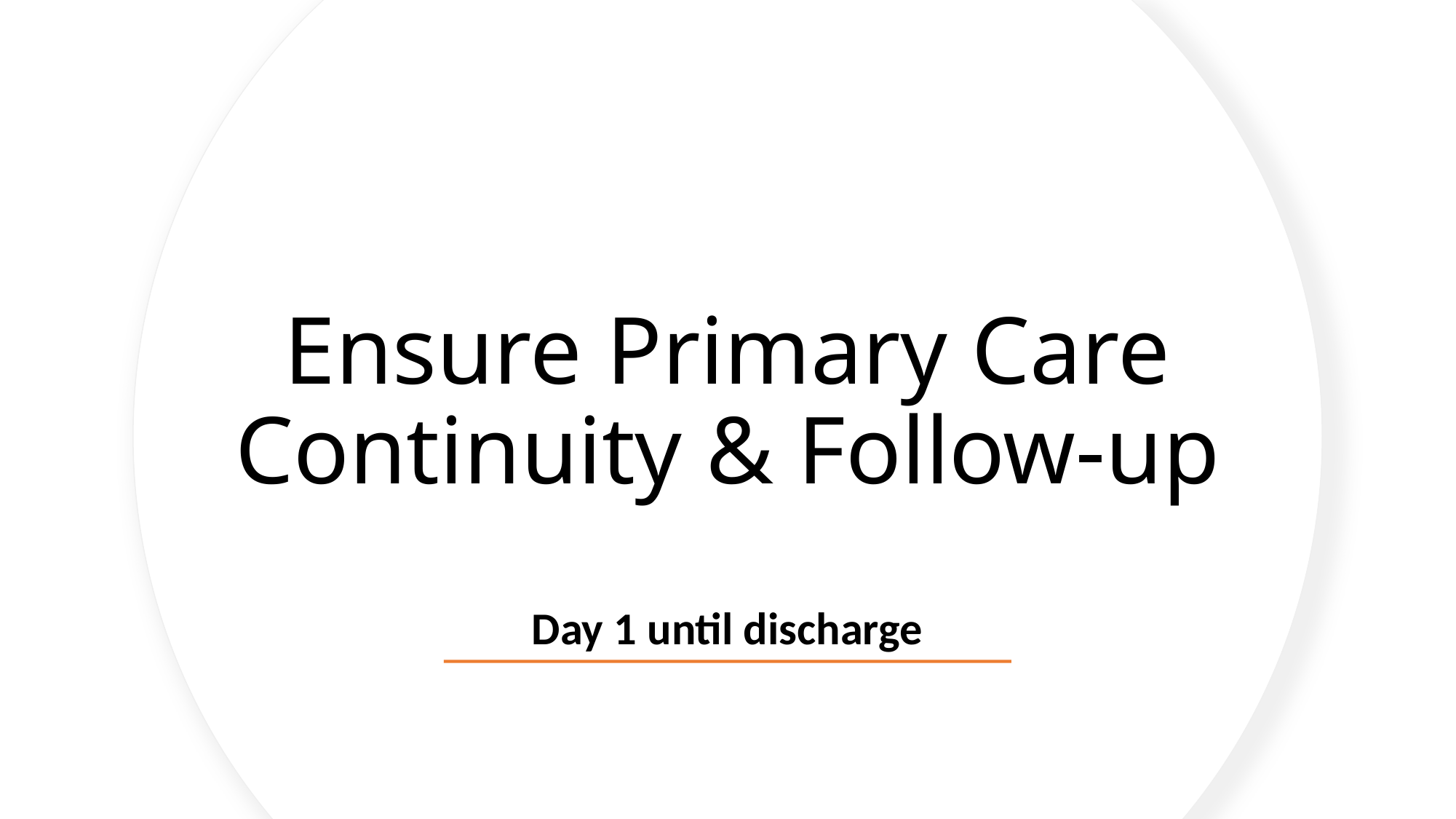

# Ensure Primary Care Continuity & Follow-up
Day 1 until discharge

## Slide 49
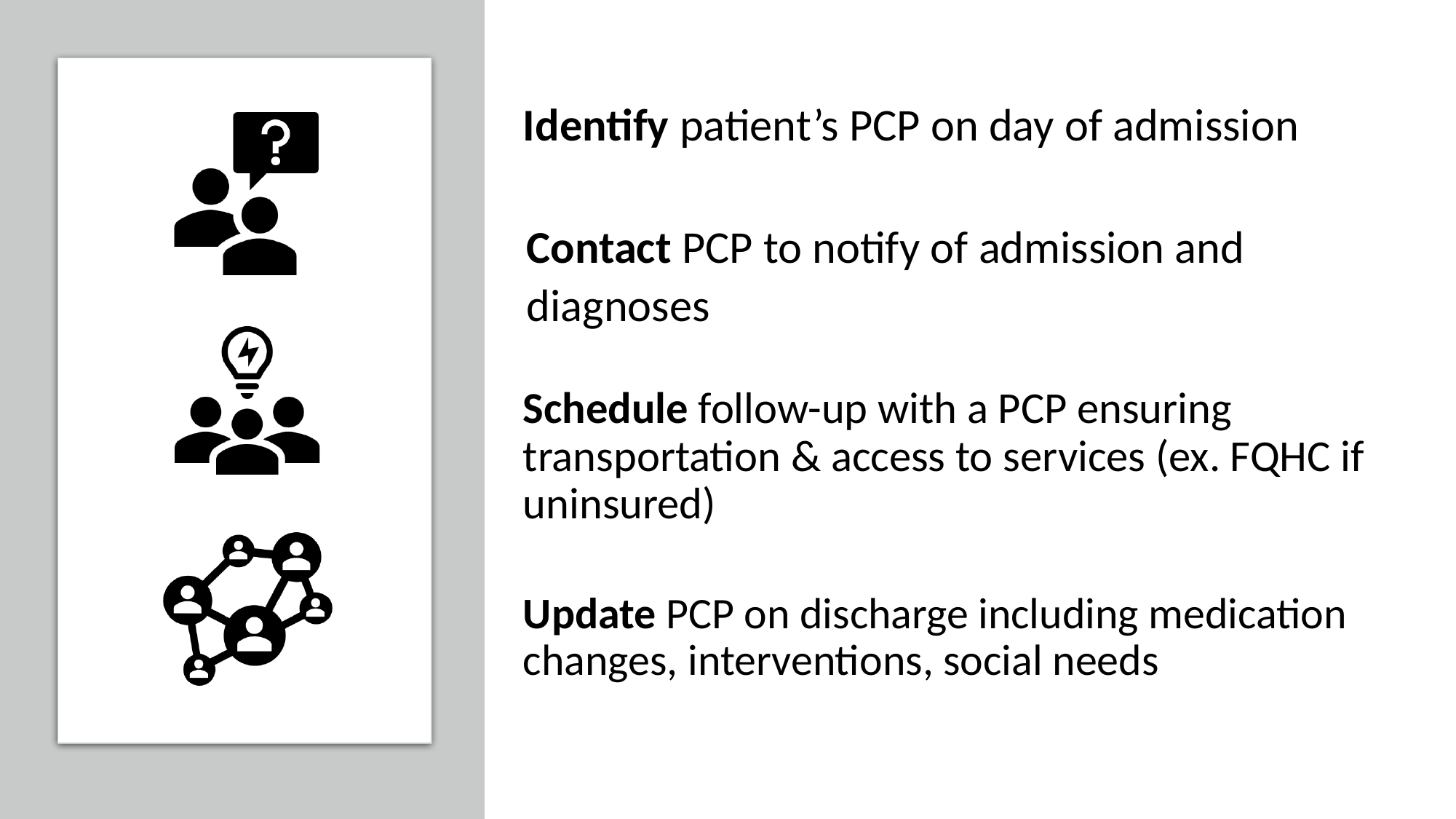

Identify patient’s PCP on day of admission
Contact PCP to notify of admission and
diagnoses
Schedule follow-up with a PCP ensuring transportation & access to services (ex. FQHC if uninsured)
Update PCP on discharge including medication changes, interventions, social needs

## Slide 50
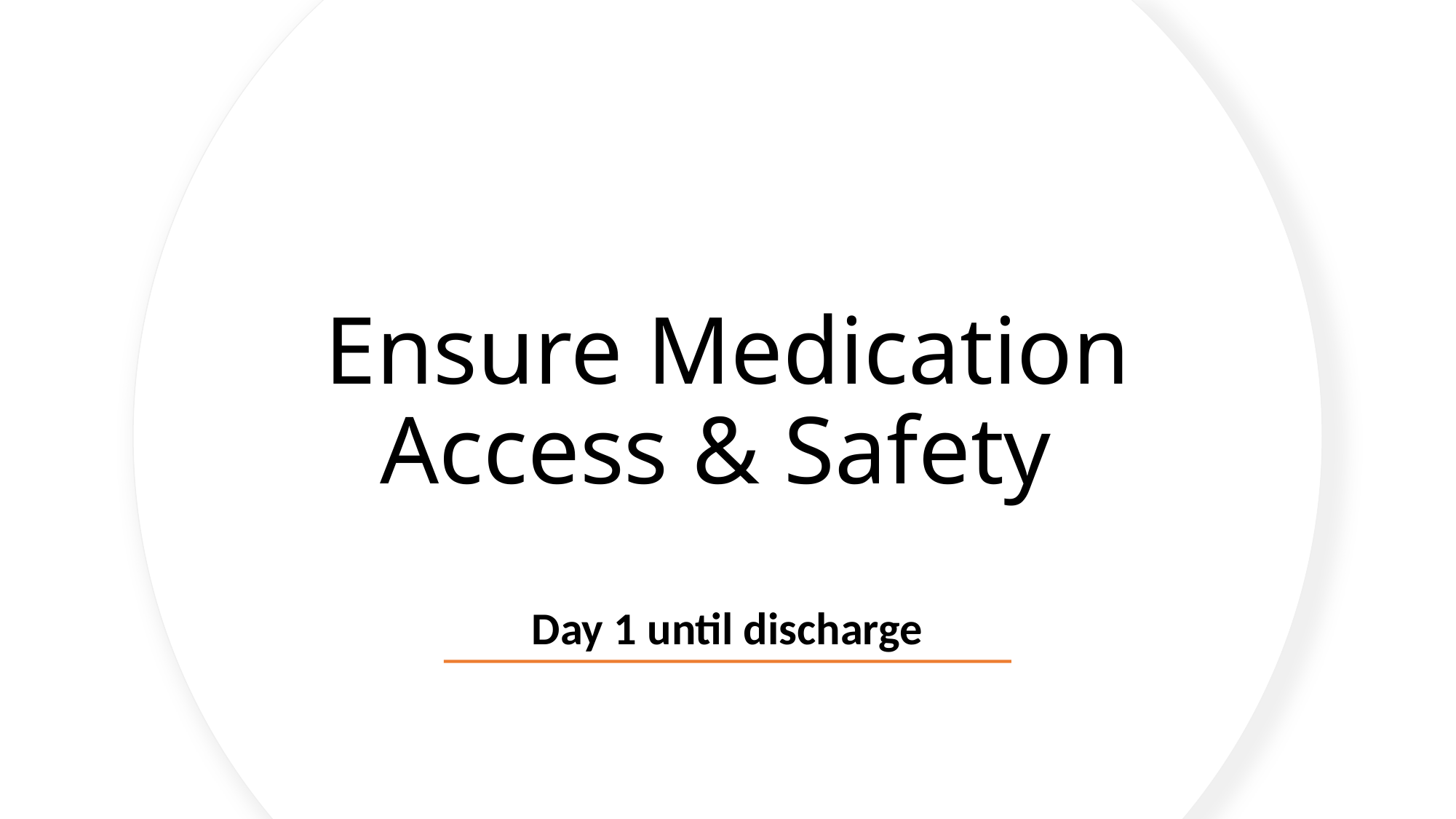

# Ensure Medication Access & Safety
Day 1 until discharge

## Slide 51
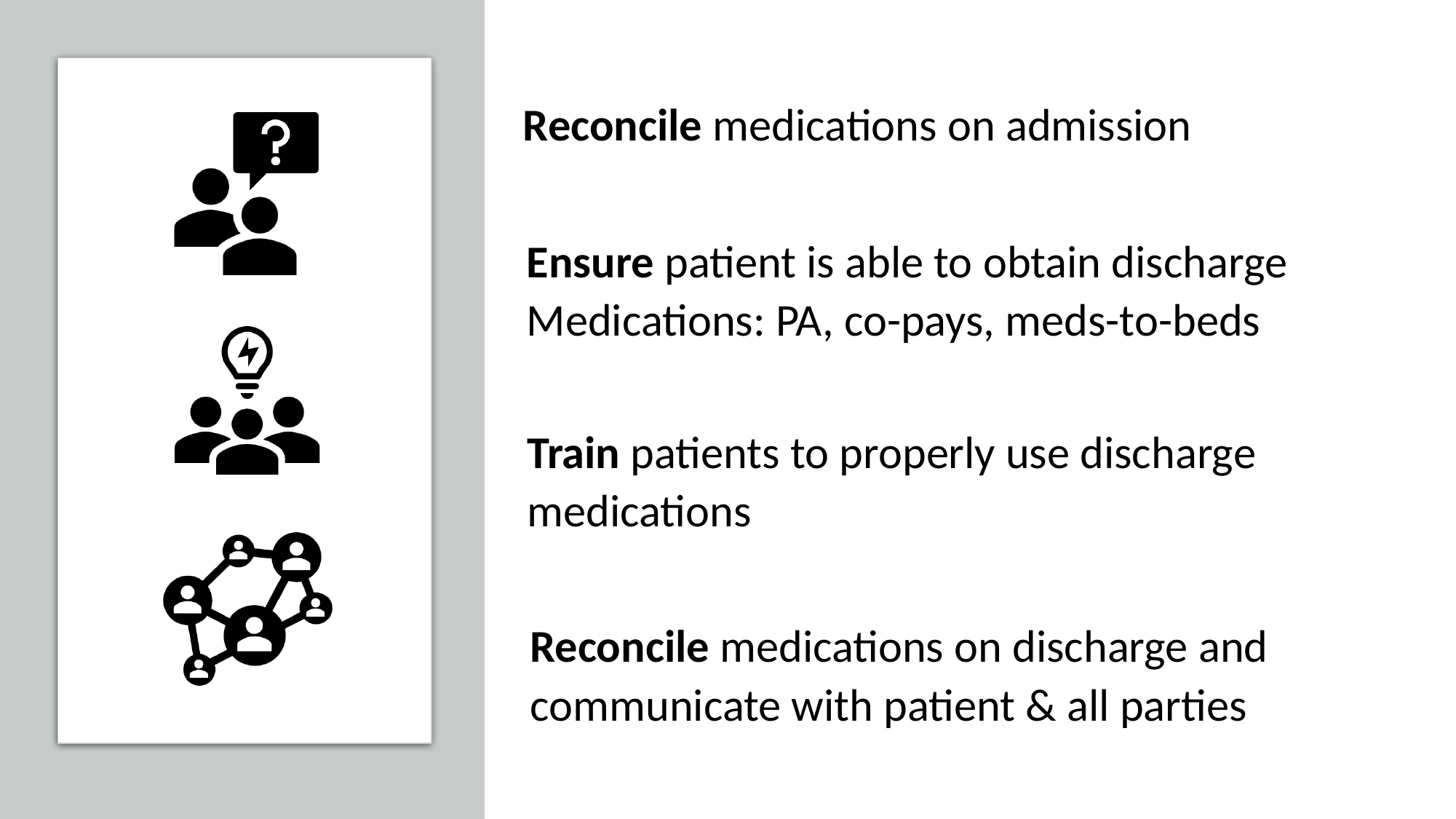

Reconcile medications on admission
Ensure patient is able to obtain discharge
Medications: PA, co-pays, meds-to-beds
Train patients to properly use discharge
medications
Reconcile medications on discharge and
communicate with patient & all parties

## Slide 52
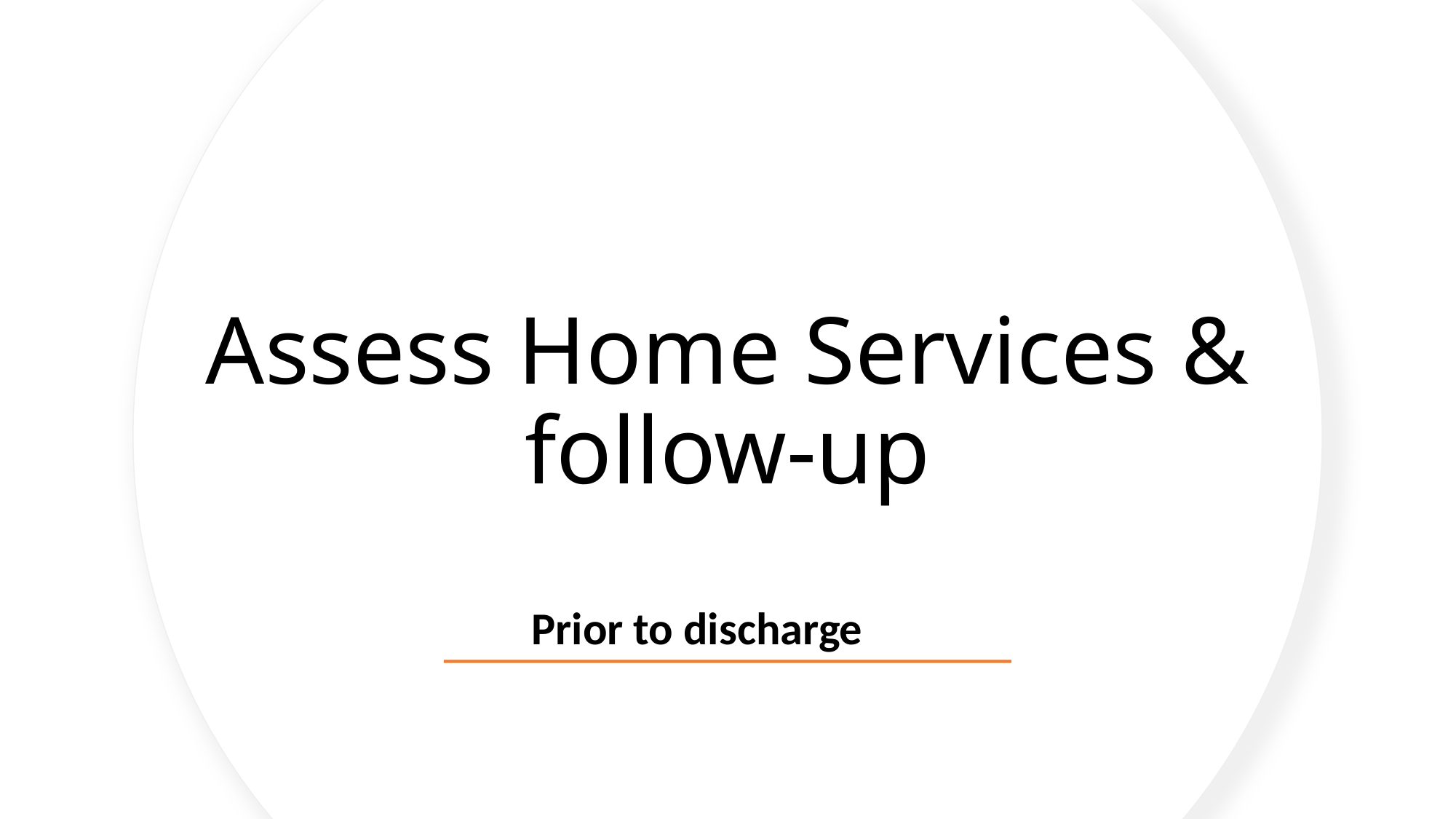

# Assess Home Services & follow-up
Prior to discharge

## Slide 53
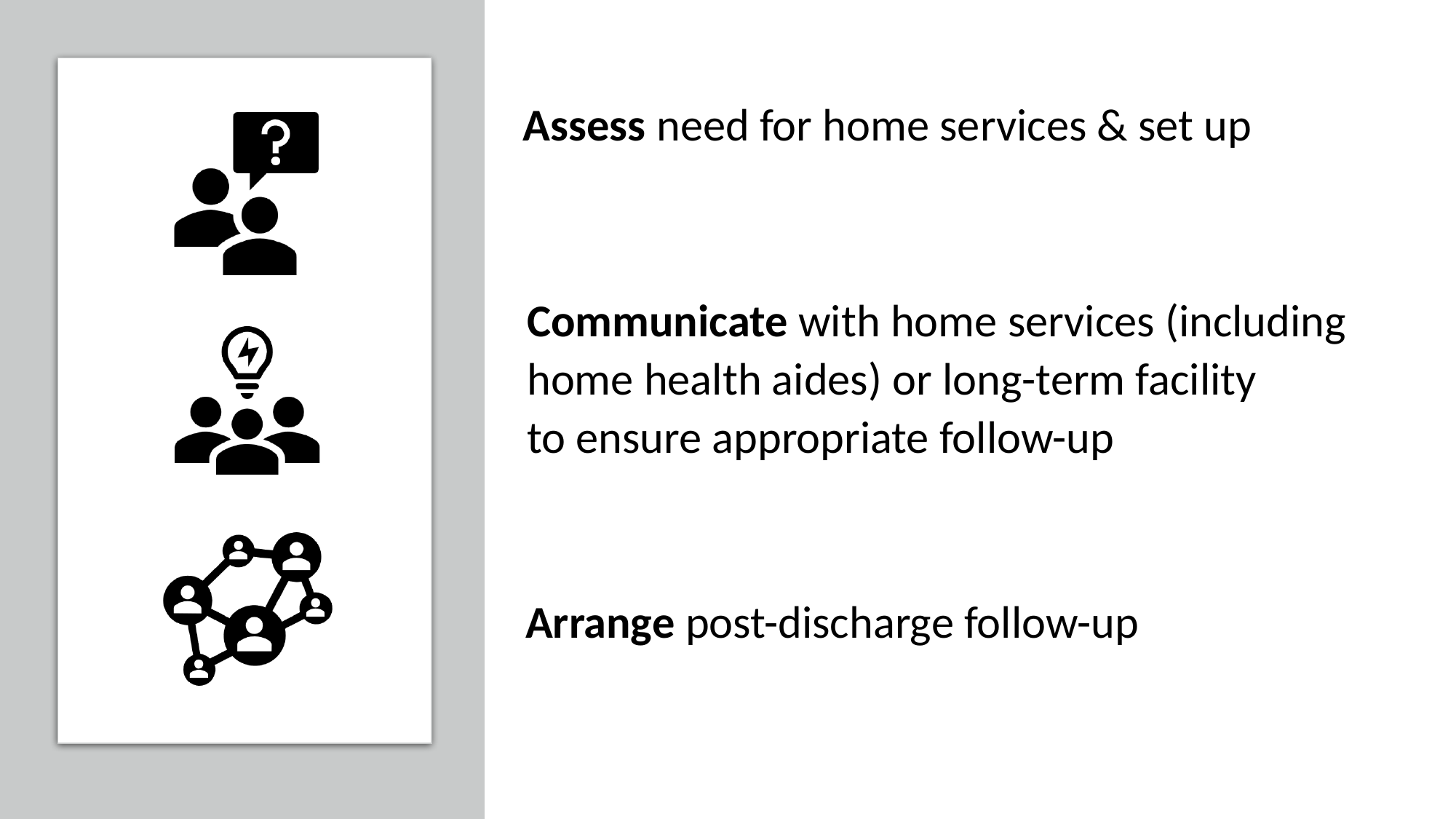

Assess need for home services & set up
Communicate with home services (including
home health aides) or long-term facility
to ensure appropriate follow-up
Arrange post-discharge follow-up

## Slide 54
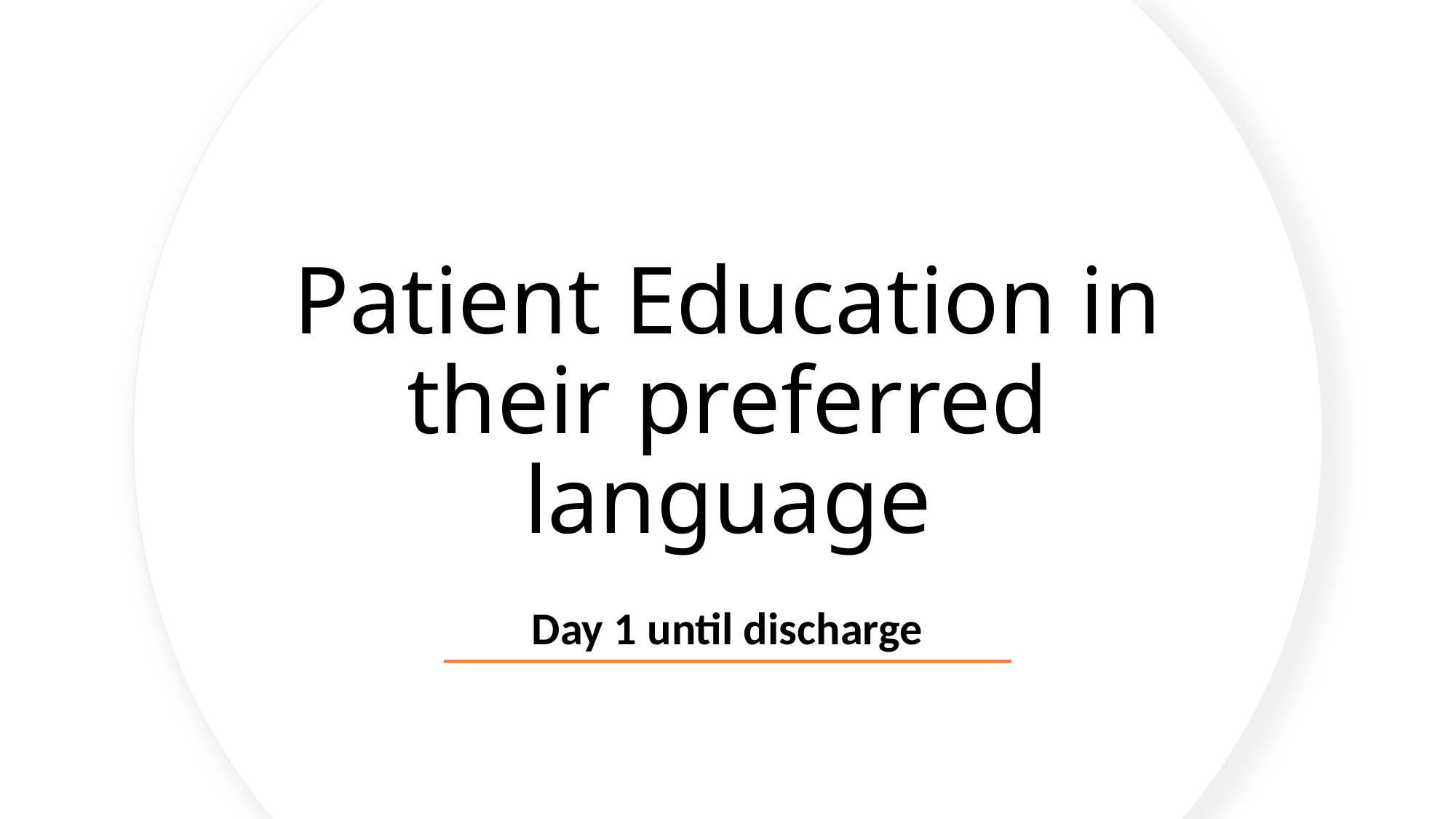

# Patient Education in their preferred language
Day 1 until discharge

## Slide 55
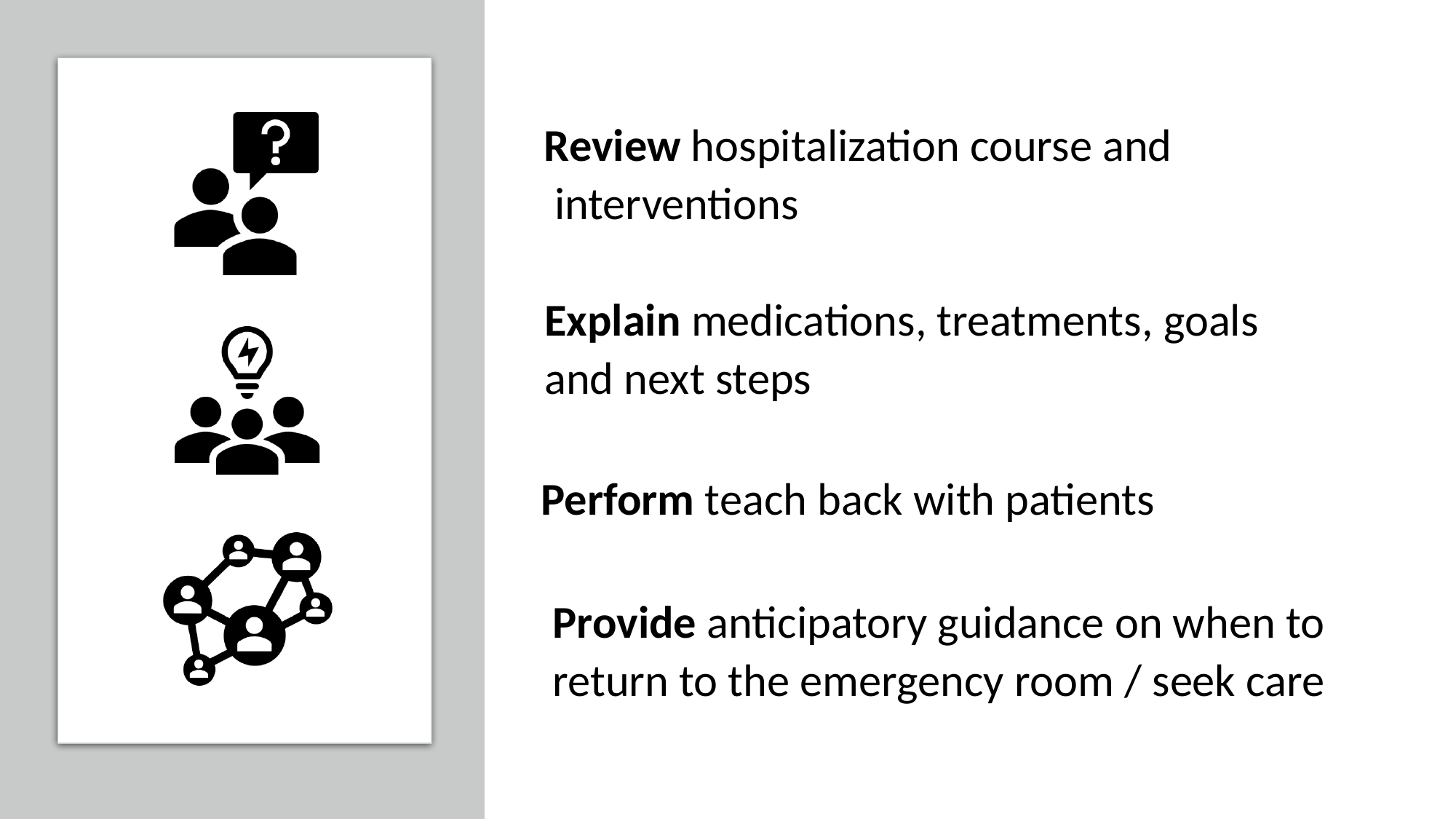

Review hospitalization course and
 interventions
Explain medications, treatments, goals
and next steps
Perform teach back with patients
Provide anticipatory guidance on when to
return to the emergency room / seek care

## Slide 56
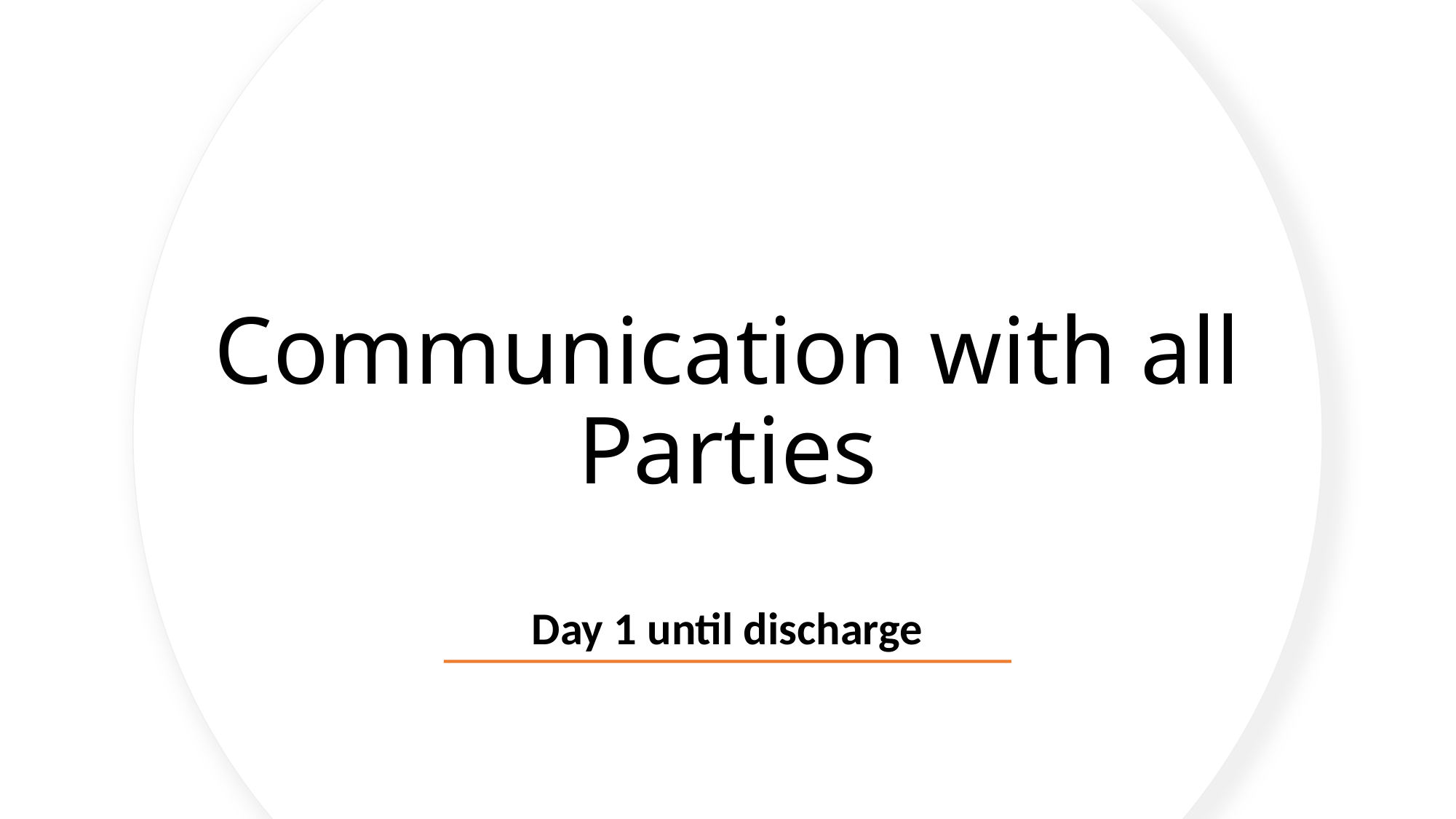

# Communication with all Parties
Day 1 until discharge

## Slide 57
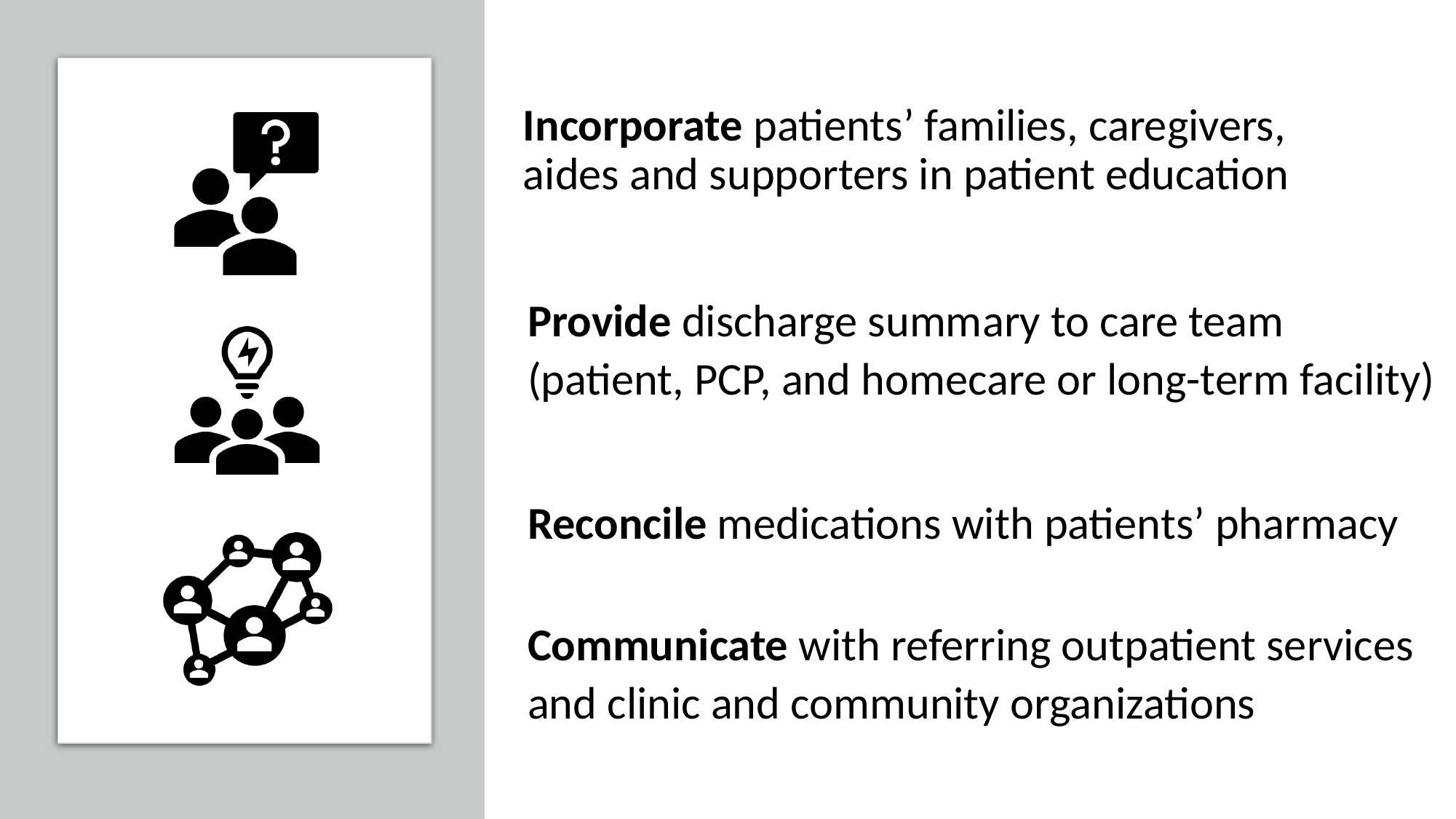

Incorporate patients’ families, caregivers, aides and supporters in patient education
Provide discharge summary to care team
(patient, PCP, and homecare or long-term facility)
Reconcile medications with patients’ pharmacy
Communicate with referring outpatient services
and clinic and community organizations

## Slide 58
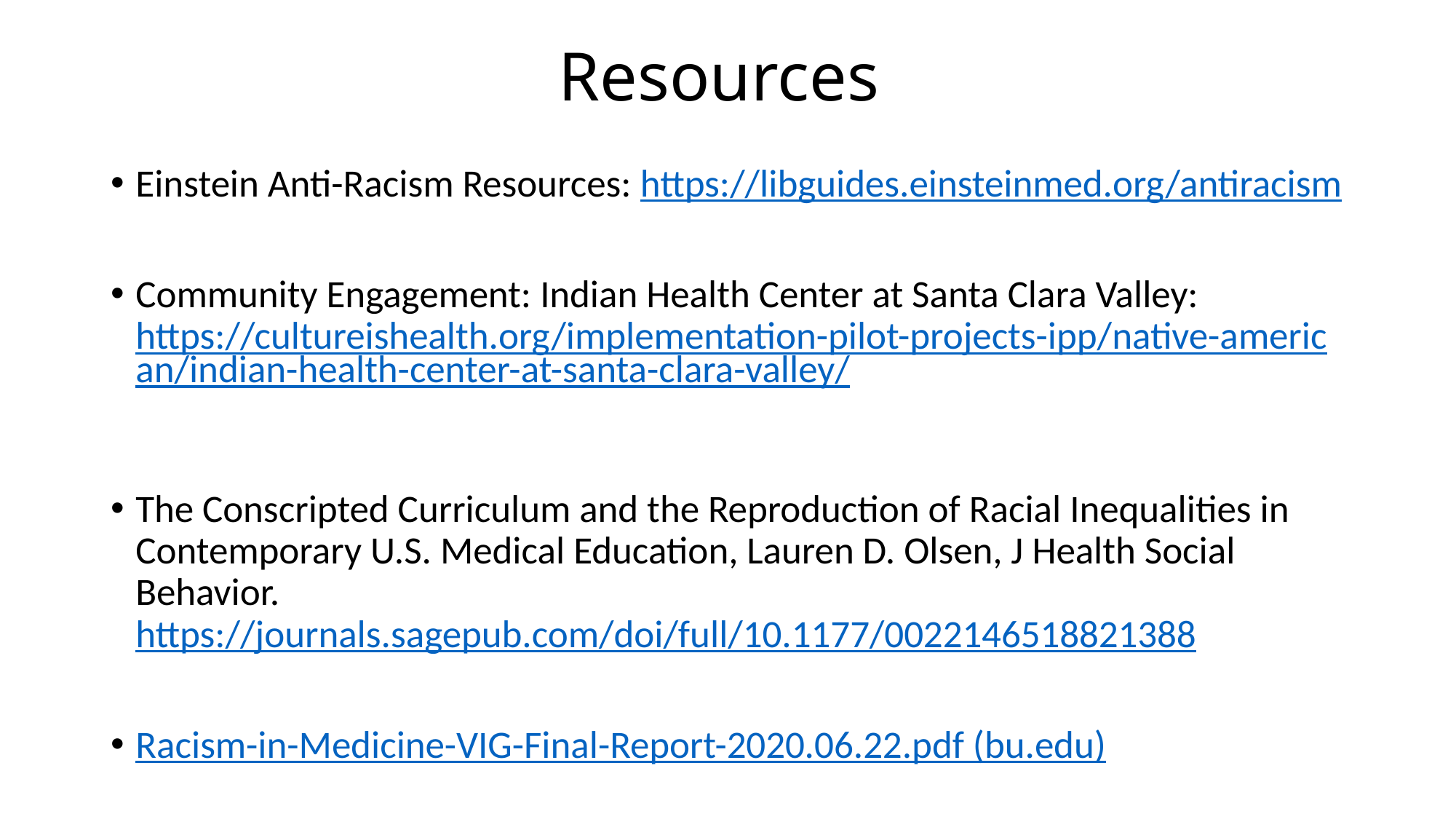

# Resources
Einstein Anti-Racism Resources: https://libguides.einsteinmed.org/antiracism
Community Engagement: Indian Health Center at Santa Clara Valley: https://cultureishealth.org/implementation-pilot-projects-ipp/native-american/indian-health-center-at-santa-clara-valley/
The Conscripted Curriculum and the Reproduction of Racial Inequalities in Contemporary U.S. Medical Education, Lauren D. Olsen, J Health Social Behavior. https://journals.sagepub.com/doi/full/10.1177/0022146518821388
Racism-in-Medicine-VIG-Final-Report-2020.06.22.pdf (bu.edu)
