# Supplementary material for: Structural Competency: A Faculty Development Workshop Series for Anti-racism in Medical Education
Source: MedEdPORTAL. 2025 Feb 7;21:11492. doi: 10.15766/mep_2374-8265.11492 (PMC11802914; doi:10.15766/mep_2374-8265.11492)
Supplement: Supplementary file 1 — 1 - Introduction to SC.pptx1 - Facilitator Guide.docx1 - SC Rubric Handout.docx1 - Sample SC Learning Goals.docx2 - Resident Reports & Case-Based Presentations.pptx2 - Facilitator Guide.docx2 - Structural Differential Handout.docx2 - Small-Group Handout.docx3 - Demystifying SC.pptx3 - Facilitator Guide.docx3 - SC One-Minute Preceptor Handout.docx3 - SC SNAPPS Handout.docx3 - Role-Play Scenarios.docx4 - SC Hospital-Based Teaching.pptx4 - Facilitator Guide.docx4 - Daily Inpatient Checklist.docx4 - SC Discharge Checklist.docx4 - Small-Group Scenarios.docxPre- and Postsurveys.docx [file mep_2374-8265.11492-s001.zip › O. 4 - Facilitator Guide.docx]

Appendix O - WS 4 Slides and Talking Points

| **Slide 1**  Welcome Slide Time Check 00:00 | **Big Picture**: Set a welcoming learning environment. |  |
| --- | --- | --- |
| 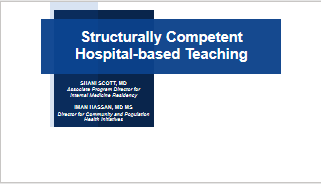 |  |  |
| **Slide 2**  Learning Objectives | **Big Picture**: Overview of learning goals for the session |  |
| 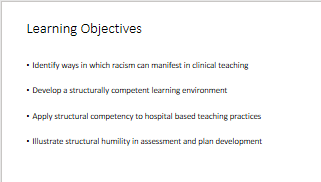 |  |  |
| *Suggested script/talking points:* Read learning objectives off slide. | |  |
| **Slide 3**  Terms | **Big Picture**: Review key terms. Pay attention to the bolded orange items on the slide |  |
| 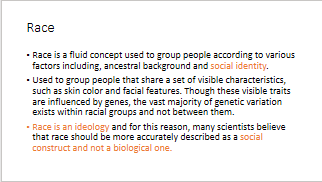 |  |  |
| *Suggested script/talking points:*  I would like to take a moment to discuss the definition of race how it influences our practice of structural competency and teaching practices.  Therefore I want to present a uniform definition of race outlined by the NIH Nation Human Genome Research Institute | |  |
| **Slide 4**  New ACGME Policy | **Big Picture**: Review new expectations of training institutions |  |
| 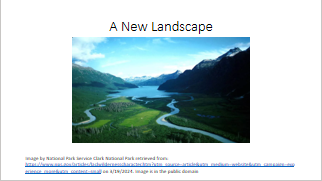 |  |  |
| *Suggested script/talking points:*  The new ACGME Common Program Requirements emphasizing the recruitment and retention of a diverse and inclusive workforce, the time is right for this new competency.^1^ Resident-led education on topics of diversity, health disparities, health equity, and social justice reveal an unmet need within traditional curricula. In addition to preparing residents and fellows to better serve diverse patients and communities, our new competency has the potential to enhance the recruitment and work satisfaction of trainees from minority groups historically underrepresented in medicine by creating educational environments that are more critically reflective of topics like implicit bias, racism, personal histories, and privilege, among others  Ref: Castillo EG, Isom J, DeBonis KL, Jordan A, Braslow JT, Rohrbaugh R. Reconsidering Systems-Based Practice: Advancing Structural Competency, Health Equity, and Social Responsibility in Graduate Medical Education. Acad Med. 2020 Dec;95(12):1817-1822. doi: 10.1097/ACM.0000000000003559. PMID: 32590465; PMCID: PMC8279228. | |  |
| **Slide 5**  Race-based Medicine and Inequities Time Check 05:00 minutes | **Big Picture**: Discuss how the influence of race-based medicine impacts all stages of knowledge acquisition to dissemination and practice. Spend up to five minutes on this slide considering it reviews key concepts. |  |
| 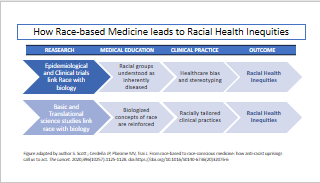 |  |  |
| *Suggested script/talking points:*  **Step 1: Research** **Remarks:** "We begin with the role of research. Historically, medical research has often used race as a variable, assuming it represents genetic differences. This has led to the development of race-based guidelines and tools, such as race-adjusted algorithms for kidney function or cardiovascular risk. These flawed research practices reinforce the idea that race is biologically significant, rather than a social construct influenced by external factors."  **Step 2: Medical Education** **Remarks:** "Next, these research findings are integrated into medical education. Medical students and trainees are taught to view race as a key factor in diagnosis and treatment decisions. This perpetuates the belief that certain racial groups are predisposed to specific diseases or have different physiological norms. As a result, future healthcare providers internalize and propagate these biased practices."  **Step 3: Clinical Practice** **Remarks:** "In clinical practice, race-based medicine manifests in the form of differential treatment based on a patient's racial background. For instance, clinicians might use race-specific criteria for prescribing medications or assessing disease risk, without considering the individual patient's social context or environmental exposures. This leads to inconsistent and often inequitable care."  **Step 4: Outcome** **Remarks:** "Finally, the culmination of race-based research, education, and clinical practice results in significant racial health inequities. Marginalized groups may receive suboptimal care, leading to worse health outcomes, such as higher rates of chronic illness, untreated pain, and preventable mortality. These disparities are not just the result of individual biases but are embedded in the very structure of our healthcare system.  To address these inequities, we must critically examine and reform the entire process—from how research is conducted, to what is taught in medical schools, to how care is delivered in clinical settings. Only by dismantling race-based medicine can we move towards truly equitable healthcare. | |  |
| **Slide 6**  Race-conscious medicine combats health inequities. Time Check 08:00 minutes | **Big Picture**: Race-conscious medicine stops the pathologizing communities based upon race and refocuses on the structural and social determinants of health. |  |
| 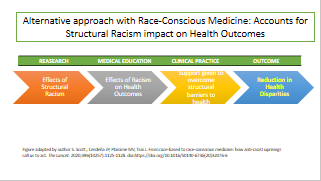 |  |  |
| *Suggested script/talking points:*  It's important to note that while race has no biological basis, it has real and profound effects on people's lives because of how society interprets and responds to these constructed categories. For example, racial disparities in healthcare, education, and criminal justice are not due to genetic differences but are the result of systemic biases and discrimination rooted in these social constructs.  Understanding race as a social and political construct allows us to challenge and dismantle the systems of inequality that are built upon it. It shifts the focus from individual differences to the broader societal structures that perpetuate racial inequities. | |  |
| **Slide 7**  Literature review of the effects of race-based medicine. Time Check 20:00 minutes | **Big Picture**: Highlight research that demonstrates the downstream effects of race based medicine. Spend three minutes |  |
| 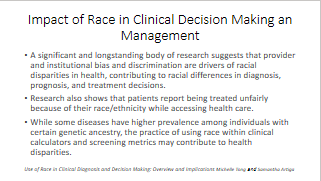 |  |  |
| *Suggested script/talking points:*  **A significant and longstanding body of research suggests that provider and institutional bias and discrimination are drivers of racial disparities in health, contributing to racial differences in diagnosis, prognosis, and treatment decisions.**  -  A 2015 systematic review of published studies showed that most health care providers appear to have [implicit bias](https://www.ncbi.nlm.nih.gov/pmc/articles/PMC4638275/pdf/AJPH.2015.302903.pdf) in terms of positive attitudes towards White people and negative attitudes towards people of color. Providers who endorse [false beliefs about biological differences](https://www.ncbi.nlm.nih.gov/pmc/articles/PMC4843483/) by race report lower pain for Black patients compared to White patients, which has been linked to systematic [undertreatment for pain](https://www.pnas.org/content/113/16/4296) of Black patients. Similarly, compared to White patients in emergency departments, Hispanic and Asian patients are [less likely to receive pain assessments](https://journals.lww.com/lww-medicalcare/Abstract/2019/12000/Racial_Ethnic_Disparities_in_Pain_Treatment_.2.aspx) and appropriate pain medication.  **Research also shows that patients report being treated unfairly because of their race/ethnicity while accessing health care.**   - **2020 KFF/The Undefeated Survey revealed that** Black adults also are more likely than White adults to report negative experiences with health care providers, including feeling a provider did not believe they were telling the truth, being refused a test or treatment they thought they needed, and being refused pain medication. In addition, Black and Hispanic adults are more likely than their White counterparts to say it is difficult to find a doctor who shares their background and experiences and one who treats them with dignity and respect.   **While some diseases have higher prevalence among individuals with certain genetic ancestry, the practice of using race within clinical calculators and screening metrics may contribute to health disparities.**  - One of the most well-known examples of this practice is within nephrology, where separate measures of kidney function (i.e., estimated glomerular filtration rates, eGFRs) are applied to Black patients compared to non-Black patients. pulmonary function tests have a race correction factor, East Asian race is considered a major risk factor for neonatal jaundice, and a different Body Mass Index threshold is used to recommend diabetes testing among asymptomatic Asian and Pacific Islander patients. | |  |
| **Slide 8**  Unequal Treatment | **Big Picture**: Promote the reading of Unequal Treatment |  |
| 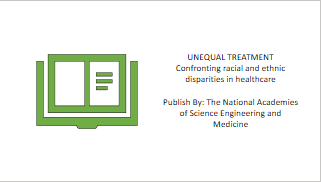 |  |  |
| *Suggested script/talking points:*  In *Unequal Treatment*, a panel of experts documents this evidence and explores how persons of color experience the health care environment. The book examines how disparities in treatment may arise in health care systems and looks at aspects of the clinical encounter that may contribute to such disparities. Patients' and providers' attitudes, expectations, and behavior are analyzed. | |  |
| **Slide 9**  Evidence of disparities influenced by bias | **Big Picture**: Implicit bias among physicians may impact clinical decision-making in ways that perpetuate health care disparities. Indoctrination starts in medical school |  |
| 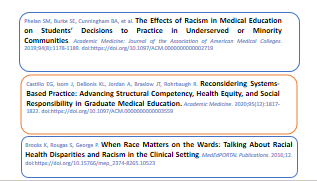 |  |  |
| *Suggested script/talking points:*  A number of studies have demonstrated that physicians are implicitly biased, which is likely to impact their clinical decision-making. Research suggests that implicit bias may contribute to health care disparities by shaping physician behavior and producing differences in medical treatment along the lines of race, ethnicity, gender or other characteristics. Because populations in the U.S. that experience the greatest health disparities also suffer from negative cultural stereotypes, implicit bias among physicians may impact clinical decision-making in ways that perpetuate health care disparities. (JGIM)  Phenomenon could be occurring as early as medical school: These medical school factors may operate at multiple intersecting levels.^17^ For example, a lack of emphasis in the curriculum on minority health or health equity may reflect a structural, or institutionalized, form of racism that can potentially influence medical students’ interest in working with underserved populations.^4,18^ In addition, less formal curricula and the learning environment,^3,19–21^ as well as personal attitudes regarding race, can influence students’ intentions to practice in underserved areas. | |  |
| **Slide 10**  Example of misrepresentation of race in preclinical curricula | **Big Picture**: NEJM article that highlights how biased clinical reasoning starts in preclinical curricula |  |
| 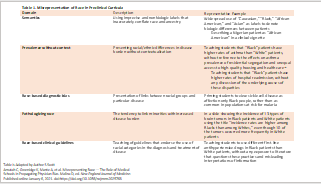 |  |  |
| *Suggested script/talking points:*  This table, adapted from the study by Amutah and colleagues published in the New England Journal of Medicine, highlights the critical ways in which medical education can perpetuate racial biases among physicians. The study underscores that medical schools often misrepresent race by teaching it as a biological factor, rather than as a social and political construct.  Key points from the table illustrate how race is frequently linked to disease prevalence, diagnostic criteria, and treatment protocols without sufficient context. For instance, the use of race-specific guidelines in areas such as kidney function and cardiovascular risk is widespread, despite evidence that these practices are based on flawed assumptions.  The table also sheds light on how this misrepresentation influences medical students' and trainees' perceptions, leading to biased clinical decision-making. When race is incorrectly emphasized as a determinant of health, it not only reinforces stereotypes but also contributes to the persistent racial health inequities we see in clinical outcomes | |  |
| **Slide 11**  Structural Competency | **Big Picture**: Duplicate slide from previous presentations to emphasize the definition of structural competency. |  |
| 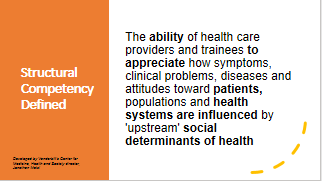 |  |  |
| *Suggested script/talking points:*  Structural competency refers to the trained ability to recognize and respond to health and illness as the downstream effects of broad social, economic, and political structures. Unlike cultural competency, which focuses on individual beliefs and behaviors, structural competency emphasizes understanding how systemic factors—such as policies, institutional practices, and social norms—shape health outcomes. This concept encourages healthcare professionals to consider these larger structural influences when diagnosing, treating, and preventing illness, ultimately aiming to address and reduce health disparities. | |  |
| **Slide 12**  Overview of Structural and Social Determinants of Health | **Big Picture**: Describe how the social and structural determinants of health impact health disparities. |  |
| 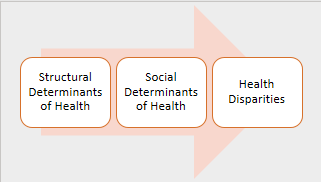 |  |  |
| *Suggested script/talking points:*  Structural Determinants of Health and the “social and political mechanism[s] that generate, configure and maintain social hierarchies”, for example, racism, labor markets and political institutions. These in turn impact the conditions in which people are *born, grow, work, live, and age,* also known as the Social Determinants of Health. Examples include economic stability, neighborhood and physical environment, education, food, community and social context, and health care system. Social determinants of health contribute to health outcomes and to the health disparities we see across social hierarchies, ex. across races/ethnicities.  Sources:  1. Solar O, Irwin A. “A conceptual framework for action on the social determinants of health. Social Determinants of Health Discussion Paper 2 (Policy and Practice).” *World Health Organization.* 2010  2. Artiga S and Hinton E. “Beyond Health Care: The Role of Social Determinants in Promoting Health and Health Equity” *Kaiser Family Foundation Issue Brief*. May 2018. Accessed Feb 17 2020: https://www.kff.org/disparities-policy/issue-brief/beyond-health-care-the-role-of-social-determinants-in-promoting-health-and-health-equity/ | |  |
| **Slide 13**  Pillars of Structural Competency | **Big Picture**: Duplicate slide to describe the five pillar of structural competency. Focus on the bolded language. |  |
| 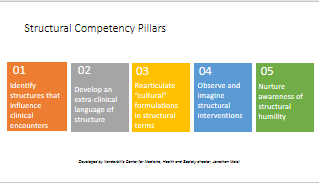 |  |  |
| *Suggested script/talking points:*   1. **Identify structures that influence clinical encounters:** economic, physical, and socio-political forces impact medical decisions 2. **Develop an extra-clinical language of structure:** recognition of language used by anthropologists, sociologists, economists, urban planners, etc. and their contributions 3. **Rearticulate “cultural” presentations in structural terms:** avoid using ”culture” to describe what are in fact structural barriers 4. **Observe and imaging structural interventions**: structures are subject to change and not immutable   (5) **Nurture awareness of structural humility:** In other words, structural humility seeks to acknowledge the complexity of structure and recognize that physicians cannot act alone or even principally to change institutions or structures. Structural change requires the concerted effort and leadership of those outside the medical field.  When teaching through the framework of structural competency physicians should evaluate their learners base on these four objectives. (Read)  Understand structure via sociology, urban planning, and economics. Recasting case presentations to acknowledge these structural barriers to health  Develop interventions to address health infrastructures  Nurture a critical awareness of structural humility | |  |
| **Slide 14**  Entry Points | **Big Picture**: Duplicate slide to emphasize the entry points to discuss structural competency within medical education. Spend three minutes. |  |
| 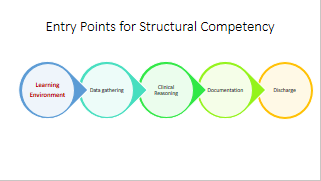 |  |  |
| *Suggested script/talking points:*  Understanding the patient's life context is crucial for building a robust therapeutic relationship and devising effective health management plans. Historically, terms like "cultural competence" have been used to emphasize the importance of integrating a patient's culture into clinical reasoning. However, these terms have evolved over time because they can inadvertently perpetuate bias and harmful stereotypes, potentially pathologizing cultural norms. This evolution reflects a growing awareness of the limitations and potential pitfalls of focusing solely on cultural factors.  Progressing toward structural competency represents an important shift. It empowers health professionals to consider the broader social, economic, and political structures that impact patient health. By understanding these factors, healthcare providers can create more accurate health recommendations and set achievable health outcome goals that align with the patient's unique life context. This approach moves beyond individual cultural considerations, aiming to address systemic issues and promote equity in healthcare. | |  |
| **Slide 15**  Challenges for Residents | **Big Picture**: Overview of workplace dynamics that can influence the safety of a clinical learning environment. Slide may Take up to three minutes due to content. |  |
| 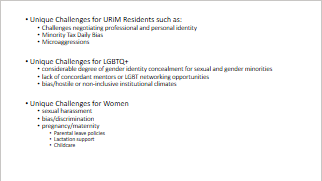 |  |  |
| Medical training is demanding for all residents, but there are unique challenges that disproportionately affect certain groups, including Underrepresented in Medicine (URiM) residents, LGBTQ+ individuals, and women.  **For URiM Residents:** URiM residents often face the difficult task of negotiating their professional and personal identities within environments where they may feel underrepresented or isolated. This can lead to what is often referred to as the 'minority tax'—the additional burden of being expected to represent their race or ethnicity in various settings, such as diversity initiatives or mentorship roles, on top of their regular duties. Daily biases and microaggressions are also common experiences, contributing to a sense of being undervalued or misunderstood within the clinical environment.  **For LGBTQ+ Individuals:** LGBTQ+ residents frequently encounter a different set of challenges. Many find themselves having to conceal their gender identity or sexual orientation due to fears of discrimination or bias in the workplace. The lack of concordant mentors or LGBTQ+ networking opportunities can make it difficult for them to find support and guidance, further exacerbating feelings of isolation. Additionally, the presence of hostile or non-inclusive institutional climates can lead to significant stress and mental health concerns.  **For Women:** Women in medical training face challenges that include sexual harassment, bias, and discrimination. These issues can hinder their professional growth and contribute to a hostile work environment. Additionally, women often have to navigate pregnancy and maternity challenges, such as negotiating parental leave policies, securing lactation support, and finding adequate childcare. These challenges can create significant barriers to career progression and work-life balance.  **Conclusion:** Addressing these unique challenges requires targeted strategies and institutional commitment to fostering an inclusive, supportive, and equitable environment for all residents. By understanding and addressing these specific issues, we can create a healthcare system that better supports the diversity of its workforce. | |  |
| **Slide 16**  Hidden Curriculum | **Big Picture**: Explaining the concept of the hidden curriculum of clinical learning environments |  |
| 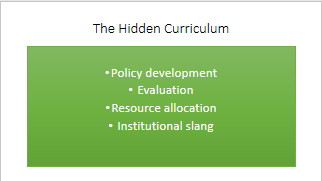 |  |  |
| *Suggested script/talking points:*  Frederic Hafferty originally defined the term with respect to medical education as the “set of influences that function at the level of organizational structure and culture.”4 He described the hidden curriculum generally as the “‘understandings,’ customs, rituals, and taken-for-granted aspects of what goes on in the life-space we call medical education” and viewed the hidden curriculum in medical education as an institutional-level concept best visible in “(1) policy development, (2) evaluation, (3) resource allocation, and (4) institutional slang.” | |  |
| **Slide 17**  Structurally Competent Hidden Curriculum | **Big Picture**: Applying structural competency to the hidden curriculum. Spend three minutes. |  |
| 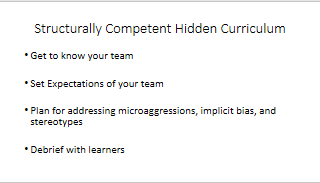 |  |  |
| *Suggested script/talking points:*  **1.Get to Know Your Team** **Remarks:** "Start by getting to know your team. Understanding the diverse backgrounds, experiences, and perspectives of your team members is crucial for fostering a supportive and inclusive environment. Take time to learn about their strengths, challenges, and what motivates them. Building these relationships helps create trust and sets the stage for effective collaboration."  **2. Set Expectations of Your Team** **Remarks:** "Clear expectations are essential for any team. Communicate your expectations around professionalism, collaboration, and inclusivity from the outset. Make it clear that respect, open communication, and support for one another are non-negotiable. This will help set the tone for a positive and productive working and learning environment."  **3. Plan for Addressing Microaggressions, Implicit Bias, and Stereotypes** **Remarks:** "It's important to have a plan in place for addressing microaggressions, implicit bias, and stereotypes when they arise. Create a safe space where team members feel comfortable discussing these issues, and ensure that there are clear protocols for addressing them. Encourage everyone to speak up if they witness or experience bias, and be prepared to intervene constructively when necessary. This proactive approach helps maintain a respectful and inclusive environment."  **4. Debrief with Learners** **Remarks:** "Regular debriefing sessions are key to reinforcing learning and addressing any concerns that may have arisen. Use these opportunities to reflect on the team's experiences, discuss any challenges related to bias or inclusivity, and celebrate successes. Debriefing allows for continuous improvement and ensures that everyone feels heard and supported." | |  |
| **Slide 18**  Approach to knowing your team | **Big Picture**: Describe steps to knowing the learners on your team. |  |
| 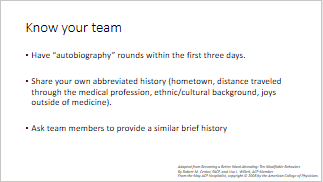 |  |  |
| *Suggested script/talking points:* Ask team members to provide a similar brief history, including their future career plans, hometown, distance traveled through the medical profession, ethnic/cultural background, and joys outside of medicine. | |  |
| **Slide 19**  Setting expectations | **Big Picture**: Explain the value of setting expectations with team |  |
| 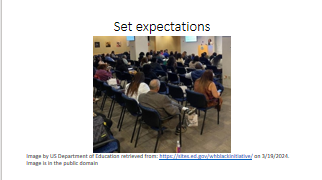 |  |  |
| *Suggested script/talking points:*  **1. Expectations of Learners:** "The first section of the guide covers expectations for how learners should act and behave in the clinical environment. This includes professionalism, communication with colleagues and patients, and maintaining a respectful and inclusive atmosphere. We emphasize the importance of teamwork, ethical behavior, and continuous learning as core components of their professional development."  **2. Social History Expectations:** "Taking an accurate and comprehensive social history is critical for providing patient-centered care. The guide outlines the key elements to cover when taking a social history, including housing, employment, substance use, and social support systems. We want to ensure that residents understand how these factors impact patient health and tailor their care accordingly."  **3. Documentation Practices:** "Proper documentation is essential for patient safety and continuity of care. The guide provides detailed instructions on best practices for documenting patient encounters, including SOAP notes, progress notes, and discharge summaries. Emphasizing accuracy, clarity, and timeliness in documentation helps prevent errors and improves communication within the healthcare team."  **4. Discharge Checklist:** "The discharge process is a critical moment in patient care. To streamline this process and ensure no steps are missed, the guide includes a comprehensive discharge checklist. This checklist, referenced as Appendix Q in the guide, covers all the necessary steps, from medication reconciliation to patient education and follow-up appointments."  **5. Evaluate on Ability to Meet Expectations:** "Finally, it's important to evaluate our residents and fellows on their ability to meet these expectations. Regular feedback and assessments will be conducted to ensure that they are not only following the guidelines but also growing as clinicians. By aligning evaluations with these clearly defined expectations, we can provide targeted support and help them excel in their training.  Discharge checklist -🡪 **Reference Appx Q**  EVALUATE ON ABILITY TO MEET those expectations  This pocket manual is designed to be a practical, accessible tool that residents and fellows can refer to throughout their training. It’s more than just a checklist; it’s a roadmap for success in clinical practice, ensuring that our learners are well-prepared to provide high-quality, compassionate care  . | |  |
| **Slide 20**  Addressing Disrespect in the Clinical Learning Environment | **Big Picture**: Explain to participants how educators can equally support the dignity of all the learners on their team to mitigate inequities of cognitive load. |  |
| 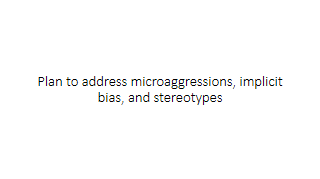 |  |  |
| *Suggested script/talking points:*  "Understanding the impact of racial stress is crucial for fostering a supportive clinical learning environment.  First, **Racial Battle Fatigue** refers to the cumulative emotional and physiological toll from enduring racial microaggressions. These repeated stressors can lead to traumatic psychological and physical symptoms.  Smith et al. have shown that this fatigue profoundly affects the clinical learning environment, contributing to increased stress among learners and impairing their ability to thrive.  Similarly, **Weathering**, as described by Dr. Arline Geronimus, illustrates how chronic exposure to stressors, including racial bias, accelerates health deterioration.  Together, these factors lead to increased cognitive load, which negatively impacts job performance and overall well-being, making it essential to address these issues in our clinical settings. | |  |
| **Slide 21**  Bystander dynamics | **Big Picture**: Discuss how GAB has depicted a figure that shows everyone is involved when microaggression/bullying event occurs. |  |
| 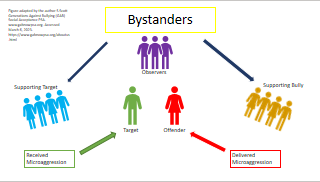 |  |  |
| *Suggested script/talking points:*  This figure has been adapted from the Generations Against Bullying (GAB) Social Acceptance PSA. GAB focuses on promoting social acceptance and addressing bullying through various educational campaigns and resources. You can explore more about their initiatives and resources on their website, [www.gabnowpsa.org](http://www.gabnowpsa.org), which was accessed on March 6, 2023. This figure is used to illustrate key points about fostering a respectful and inclusive environment. | |  |
| **Slide 22**  Communication Strategies for addressing microaggressions/bias | **Big Picture**: Overview of communication frameworks to share with learners to talk about their experiences in the clinical learning environment when targeted by discrimination/bias. |  |
| 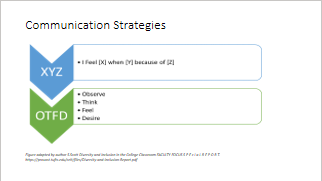 |  |  |
| *Suggested script/talking points:*  This figure is adapted from the report titled *Diversity and Inclusion in the College Classroom*, a special report by the Faculty Focus. You can access the full report here: [Diversity and Inclusion Report](https://provost.tufts.edu/celt/files/Diversity-and-Inclusion-Report.pdf).  The report provides valuable insights into best practices for promoting diversity and inclusion within the college classroom. It outlines effective strategies for creating an inclusive learning environment, addressing common challenges, and implementing supportive policies for diverse student populations.  Incorporating frameworks like XYZ and OTFD into our educational practices can enhance how we support our learners. The XYZ framework focuses on creating clear, actionable objectives and strategies to meet learners' diverse needs, while the OTFD framework emphasizes open communication, feedback, and continuous improvement.  By adapting this figure, we aim to highlight key findings and recommendations from the report and illustrate how these frameworks support inclusive teaching practices. This underscores the importance of integrating these strategies into our methods and policies to ensure all students have equitable opportunities to succeed. | |  |
| **Slide 23**  Communication Strategies to address microaggressions/bias | **Big Picture**: Overview of communication frameworks to share with learners to talk about their experiences in the clinical learning environment when targeted by discrimination/bias. |  |
| 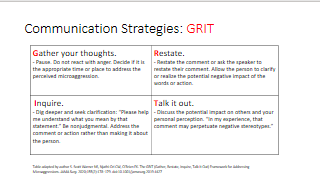 |  |  |
| *Suggested script/talking points:*  This table is adapted from the article by Warner, Njathi-Ori, and O’Brien titled 'The GRIT (Gather, Restate, Inquire, Talk It Out) Framework for Addressing Microaggressions,' published in JAMA Surgery in 2020. The GRIT framework provides a structured approach for effectively addressing and managing microaggressions. For more details, refer to the full article here. This table highlights the key components of the framework, offering practical strategies to handle microaggressions in clinical settings. | |  |
| **Slide 24**  Roles of Debriefing in the Learning Environment | **Big Picture**: Explain the role of debriefing |  |
| 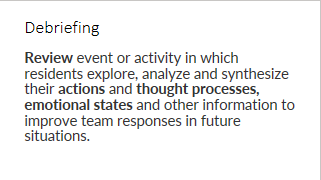 |  |  |
| *Suggested script/talking points:* Debriefing is a type of retrospective analysis of critical incidents in health professions. | |  |
| **Slide 25**  Discrimination of Teaching Environment | **Big Picture**: Explain how learners from minoritized backgrounds experience a disproportionate amount of disrespect starting with matriculation into medical school. Highlight all the manifestations of bias in the learning environment |  |
| 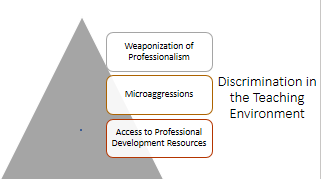 |  |  |
| *Suggested script/talking points:*  Structural racism impacts every stage of medical education.  Upon matriculation, medical students from URM backgrounds may experience an accumulation of microaggressions (or racist abuse, as described by scholar Ibram X. Kendi^15^) from patients, other students, health care professionals, and the faculty that evaluate them.^16^  Evaluations of medical students can result in the “weaponization of professionalism,”^17^ where those who do not conform to traditional White male standards of behavior are accused of being “unprofessional” and forced to “remediate” to be promoted. Also, as students progress through medical school, Black and Asian students are less likely than White students to be inducted into the Alpha Omega Alpha Honor Medical Society.^18^ | |  |
| **Slide 26**  Openness to Bidirectional Learning | **Big Picture**: Educators can also learn from their students regarding how to create a safe structurally competent clinical learning environment. |  |
| 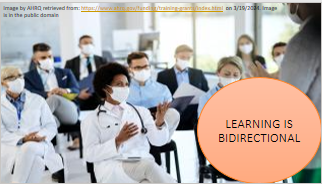 |  |  |
| *Suggested script/talking points:*  Listen to what your learners are saying  Learning is bidirectional  Open to diverse input from learners | |  |
| **Slide 27**  Quiz Time Check 35:00 minutes | **Big Picture**: Asses knowledge acquisition reset focus on content. Spend three minutes. |  |
| 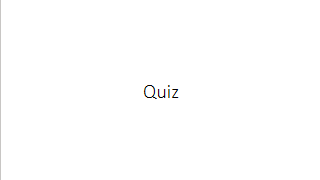 |  |  |
| *Suggested script/talking points:*  The following quiz was administered via a zoom poll. Correct answers are bolded:  Which of the following exemplifies the best practices of a ward attending? (Select all that apply)   1. **Shares experience as being first generation physician, used to be a hostess throughout college and medical school, now writes comic for fun** 2. **Pulls aside resident to discuss thoughts and emotions after patient complains about roommate “not speaking English”** 3. Describes patient admitted with cirrhosis as an alcoholic 4. Asks resident to “stay on topic” when they discuss the history of discrimination within communities of color | |  |
| **Slide 28**  Entry Points | **Big Picture**: Highlight additional entry points to incorporate structural competency. |  |
| 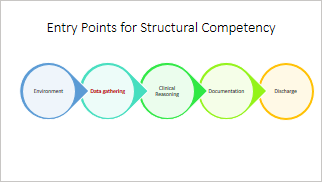 |  |  |
| *Suggested script/talking points:*  Once you have established a structurally competent learning environment, encouraging your learners to gather comprehensive social data from patients can further promote structural competency on the wards. | |  |
| **Slide 29**  Social History in Presentation | **Big Picture**: Highlight how the social history is a critical aspect of clinical case presentations. |  |
| 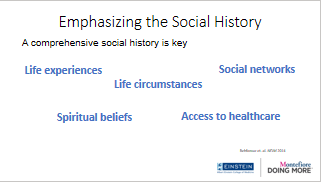 |  |  |
| *Suggested script/talking points:*  It is essential to set the expectation that gathering and emphasizing a comprehensive social history is crucial for a competent admission process. Understanding a patient’s social history provides valuable context that impacts their overall health and well-being.  **Components of a Comprehensive Social History Include:**  **1. Life Experiences:**   - **Education:** The patient’s educational background can influence their health literacy and understanding of medical information. - **Job History:** Employment status and history can affect access to healthcare and impact stress levels. - **Military Service:** Military experience may contribute to unique health concerns or needs. - **Incarceration:** Previous incarceration can have implications for mental health and access to care. - **Immigration:** Immigration status and experiences can affect health outcomes and access to services. - **Key Experiences:** Significant life events, such as trauma or major transitions, are important to consider.   **2. Life Circumstances:**   - **Family Structures:** Understanding family dynamics and support systems can provide insight into the patient’s social support. - **Obligations:** Responsibilities like caregiving or financial pressures can impact health. - **Housing:** Housing stability affects health conditions and access to care. - **Neighborhood:** The safety and resources available in the neighborhood influence health outcomes. - **Food Security:** Access to sufficient and nutritious food is a critical aspect of health.   **3. Social Networks:**   - **Spiritual Beliefs:** Spirituality can play a role in coping strategies and overall health.   **4. Access to Healthcare:**   - **Health Literacy:** The patient’s ability to understand health information is essential for effective care. - **Health Insurance:** Insurance status affects access to and affordability of care. - **Barriers to Healthcare Access:** Identifying obstacles such as transportation or language barriers is important. - **Medication Affordability:** Ensuring medications are affordable is crucial for adherence and health management.   By thoroughly exploring these areas, we ensure a holistic understanding of our patients, which is vital for providing personalized and effective care. | |  |
| **Slide 30**  SDoH Screeners | **Big Picture**: Encourage educators to instruct their learners to routinely incorporates SDoH screeners in admission work flows. |  |
| 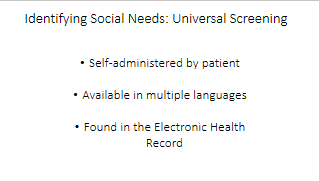 |  |  |
| *Suggested script/talking points:*  Universal screening for social needs can also be incorporated into your social history gathering. This is an example of a social needs screener used at Montefiore/Einstein which asks about safe/stable housing, housing conditions, utilities, food, transportations, medication adorability, child/elder care, legal needs and personal safety. It is meant to be self administered by the patient, available in multiple languages and found in the electronic health record.  To avoid unintended consequences when screening for social needs:  Ensure you know how to refer patients to appropriate resources  Avoid making assumptions about patients’ experiences or needs  Ask permission, delve deeper into unmet social needs  Always elicit patient’s concerns, opinions and priorities before referring (shared decision making)  Maintain a strengths based approach | |  |
| **Slide 31**  Entry Points | **Big Picture**: Clinical Reasoning exercises are opportunities to discuss structural competency |  |
| 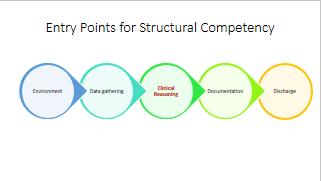 |  |  |
| *Suggested script/talking points:*  Next, incorporate structural competency into clinical reasoning during presentations for admissions and daily rounds. | |  |
| **Slide 32**  Structural Differential | **Big Picture**: Duplicate slide from previous presentations that discusses framework for how educators can instruct learners on how to generate structural differentials. |  |
| 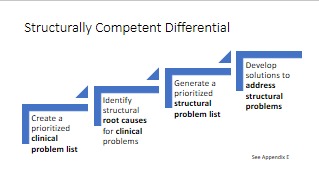 |  |  |
| *Suggested script/talking points:*  The structural differential is a step-by-step guide to teaching your learners how to create a structurally competent assessment and plan.  **Create** a prioritized clinical problem list  Ensure congruity between patient and clinician problem lists.  **Identify** structural root causes for clinical problems  Elicit upstream structural and social determinants of health that contribute to clinical problems.  Integrate historical context.  **Generate** a prioritized structural problem list  Incorporate patient priorities, preferences, and concerns.  Prioritize urgent problems and problems for which clinical and community resources are available.  **Develop** solutions to address structural problems  Driven by patients  Imagine individual-level, health system-level, community level and population-level solutions.  Consider individual and community strengths/assets.  Partner with an interdisciplinary team participating in community-led efforts | |  |
| **Slide 33**  Example of Structural Differential | **Big Picture**: Walk through an example of developing a structural differential |  |
| 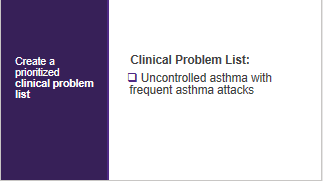 |  |  |
| *Suggested script/talking points:*  As an example, a patient might present with uncontrolled asthma with frequent asthma attacks. A traditional clinical problem list might state “uncontrolled asthma with frequent asthma attacks.” | |  |
| **Slide 34**  Structural Differential: Root Cause Analysis | **Big Picture**: Explain sequential steps to creating a root cause analysis |  |
| 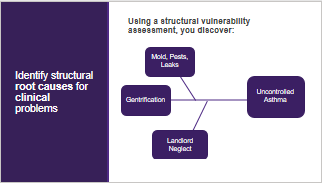 |  |  |
| *Suggested script/talking points:*  Exploring social and structural root causes using a root cause diagram (incorporating information your patient’s social history and from literature and sources outside the clinic) revels that your patient has poor housing conditions and numerous triggers in their home such as leaks, mold and pests. Your patient’s building has been cited in an increasing number of housing violations as the neighborhood is starting to gentrify. All of these are contributing root causes to the patient’s uncontrolled asthma. | |  |
| **Slide 35**  Structural Differential: Problem List | **Big Picture**: Explaining Sequential Steps to creating a structural differential |  |
| 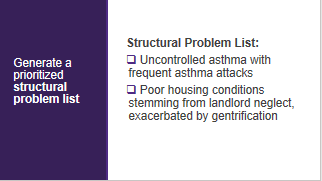 |  |  |
| *Suggested script/talking points:*  A structural problem list identifies these root causes explicitly and prioritizes root causes for which clinic and community resources are available, and needs are more urgent as prioritized by patients. | |  |
| **Slide 36**  Structural Differential: Address structural Problems | **Big Picture**: Explain sequential steps to creating a structural differential |  |
| 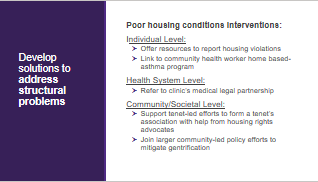 |  |  |
| *Suggested script/talking points:*  Solutions to the structural problem identified could be the following individual level solutions, health system level solutions or community/societal level solutions. Notice that at the community/societal level, the role of physicians is decentered, serving in a supportive capacity to ongoing advocacy efforts that are community led. | |  |
| **Slide 37**  Teaching Topics/Discussions | | **Big Picture**: Overview of examples of teaching topics |
| 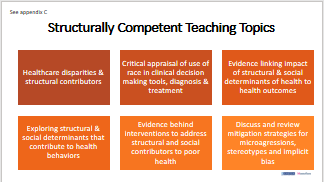 | |  |
| *Suggested script/talking points:*  Clinical presentations during admissions or daily rounds on patients is a great time to introduce structurally competent teaching topics. Here, data can be presented to learners or alternatively learners can be encouraged to research one of these topics and include them in their presentations during the assessment section.  Examples of teaching topics that are structurally competent:   - *Why are Black, Latinx and Native American individuals more likely to be diagnosed with asthma, diabetes, etc.?* - *Critically appraise use of race in eGFR assessments* - *Highlight evidence linking environmental pollutant exposure to asthma* - *In instances of “non-compliance,” inquire about etiology, ex. medication affordability, stressors, etc.* - *Highlight evidence linking housing first programs to improved mental health* - *Acknowledge potential for implicit bias when addressing a patient who previously used injection drugs* | | |
| **Slide 38**  Self Directed Learning | | **Big Picture**: Goal of educator is to instill confidence in learners to internalize independent/self-directed learning. |
| 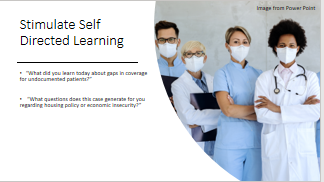 | |  |
| *Suggested script/talking points:*  When learners are allowed to reflect on what they learned, it reinforces their knowledge for next time.  Example prompts Ask:  What are the data linking socioeconomic status to cardiovascular outcomes?  What individual level, community level and societal level interventions have been shown to improve this health outcome?  How did redlining contribute to the concentration of poor health in this community?  Stimulate self-directed learning regarding health and structural disparities. | | |
| **Slide 39**  Entry Point | | **Big Picture**: Documentation is a way to combat perpetuating stigma. Allot 10 minutes for Appendix R via pair and share small groups breakout rooms. |
| 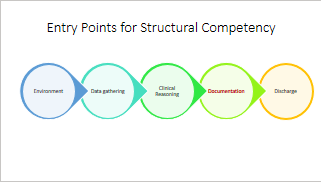 | |  |
| *Suggested script/talking points:*  Next on the progressive depiction of structural competency within medical education is a focus on encouraging the learner documentation while on the wards. | | |
| **Slide 40**  Evidence of Stigmatizing language impacting healthcare | | **Big Picture**: Stigmatizing language impacts provider attitude towards patients |
| 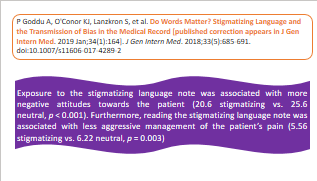 | |  |
| *Suggested script/talking points:*  What and how we document has important clinical implications. A paper in JGIM found that stigmatizing language led to more negative attitudes and less aggressive pain management than non-stigmatizing language.  Reference: P Goddu A, O'Conor KJ, Lanzkron S, et al. Do Words Matter? Stigmatizing Language and the Transmission of Bias in the Medical Record [published correction appears in J Gen Intern Med. 2019 Jan;34(1):164]. *J Gen Intern Med*. 2018;33(5):685-691. doi:10.1007/s11606-017-4289-2 | | |
| **Slide 41**  Social Need Documentation | | **Big Picture**: Additionally, CMS requiring documentation of social needs is a required skill |
| 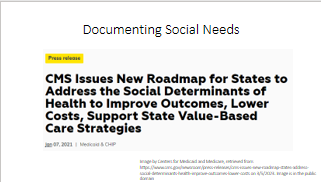 | |  |
| *Suggested script/talking points:*  Explicitly documenting social and structural problems in problem lists is important because it moves focus from personal blame and patient-level factors, and reorients focus on the broader factors that influence patient’s decisions, behaviors and circumstances. A number of ICD codes exist that can help document the structural and social factors affecting patients. A general ICD code that is easy to remember is “Problems related to the social environment.” | | |
| **Slide 42**  Best Practices | | **Big Picture**: Overview of best practices for documentation |
| 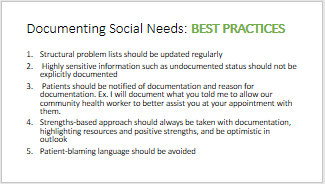 | |  |
| *Suggested script/talking points:*  Documenting structural problems in this manner ensures that these critical factors are not overlooked in patient care. It also helps in creating a more comprehensive and effective treatment plan that considers both medical and social determinants of health. By acknowledging and addressing these issues directly in our documentation, we contribute to more equitable and holistic patient care. | | |
| **Slide 43**  Quiz Time Check 60:00 minutes | | **Big Picture**: Assessment of knowledge acquisition. Spend three minutes. |
| 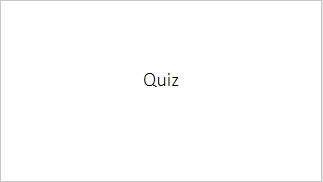 | |  |
| *Suggested script/talking points:*  The following zoom poll was included. Bolded answers are correct.  Studies have shown which of the following is true? [select all that apply]   1. **Having one social need nearly tripled 90-day mortality after hospitalization for heart failure** 2. **Patients from low SES neighborhoods are 24 percent more likely to be readmitted** 3. **A community health worker intervention has been shown to reduce multiple 30-day readmissions** | | |
| **Slide 44**  Entry Point: Discharge | | **Big Picture**: Discharge planning is another opportunity to incorporate structural competency. |
| 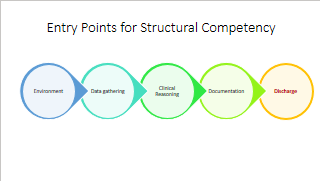 | |  |
| *Suggested script/talking points:*  Finally, using a structurally competent discharge checklist can help with ensuring that discharges are structurally competent. Structurally competent discharge planning starts on day 1 of admission. | | |
| **Slide 45**  Structurally Competent Discharge Checklist | | **Big Picture**: Encourage participants to incorporate a structurally competent discharge check list into work flow |
| 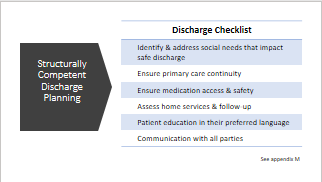 | |  |
| *Suggested script/talking points:*  We suggest providing your learners with our structurally competent discharge checklist to systematize and streamline structural determinants of health into discharge planning.  The components of a structurally competent discharge checklist are: identify and address social needs that impact safe discharge, ensure primary care continuity, ensure medication access and safety, assess home services and follow-up, patient education in their preferred language, and communication with all parties.  Adapted from a safe discharge planning checklist: Soong C, Daub S, Lee J, Majewski C, Musing E, Nord P, Wyman R et. al. Developing of a checklist of safe discharge practices for hospital patients. *Journal of Hospital Medicine*. 2013;8:444-419. | | |
| **Slide 46**  Discharge Checklist | | **Big Picture**: Walk through sequential steps to the structurally competent discharge checklist. |
| 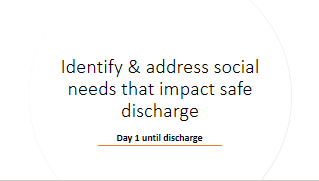 | |  |
| *Suggested script/talking points:*  Identifying and addressing social needs that impact safe discharge starts on day 1.  . | | |
| **Slide 47**  Discharge Checklist | | **Big Picture**: Walk through sequential steps to the structurally competent discharge checklist. |
| 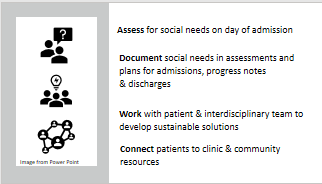 | |  |
| *Suggested script/talking points:*  Components of this item include:  Social needs should be assessed on admission; this can be done using a social needs screening tool.  Documenting a structural problem list that is updated daily.  Working with patients and interdisciplinary teams to develop solutions.  Connecting to clinic and community-based resources in partnership with and guided by patient preferences and priorities. | | |
| **Slide 48**  Discharge Checklist | | **Big Picture**: Walk through sequential steps to the structurally competent discharge checklist. |
| 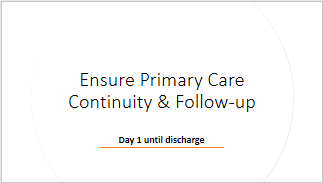 | |  |
| *Suggested script/talking points:*  Ensuring primary care continuity and follow-up also begins on day 1. | | |
| **Slide 49**  Discharge Checklist | | **Big Picture**: Walk through sequential steps to the structurally competent discharge checklist. |
| 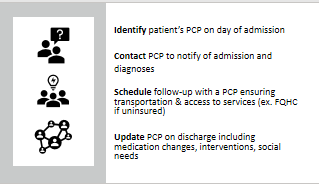 | |  |
| *Suggested script/talking points:*  Here, it’s important to make sure that patients have primary care physicians (PCPs), that their financial circumstances, transportation resources and neighborhood location is taken into account when establishing primary care (i.e. that clinic resources are able to meet patient needs and resources are available to assist patients in making it to appointments), and that the primary care physician is updated on both structural/social problems and interventions. Specific interventions may include scheduling patients with follow-up at a federally qualified health center, and arranging for insurance-based transportation or providing information on how to access transportation assistance for those with disabilities. | | |
| **Slide 50**  Discharge Checklist | | **Big Picture**: Walk through sequential steps to the structurally competent discharge checklist. |
| 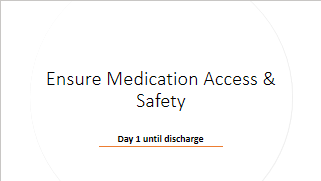 | |  |
| *Suggested script/talking points:* Ensuring medication access and safety begins on day 1. | | |
| **Slide 51**  Discharge Checklist | | **Big Picture**: Walk through sequential steps to the structurally competent discharge checklist. |
| 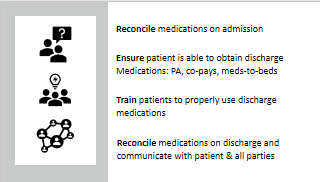 | |  |
| *Suggested script/talking points:*  Here, it’s helpful to elicit if patients can afford medications being prescribed, that they can easily access medications, and that they are aware of medications prescribed. Interventions to assist patients with medication access might include coupons (ex. goodrx.com) or clinic-based medication assistance programs, submitting prior authorizations before discharge, bringing medications to the bedside prior to discharge, and or arranging for blister packing and delivery of medications through outpatient pharmacies. | | |
| **Slide 52**  Discharge Checklist | | **Big Picture**: Walk through sequential steps to the structurally competent discharge checklist. |
| 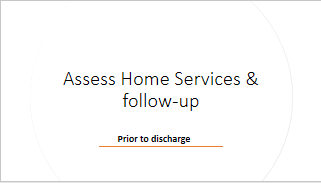 | |  |
| *Suggested script/talking points:*  It’s important to assess home services prior to discharge. | | |
| **Slide 53**  Discharge Checklist | | **Big Picture**: Walk through sequential steps to the structurally competent discharge checklist. |
| 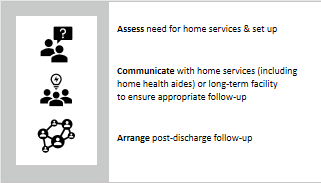 | |  |
| *Suggested script/talking points:*  Patients may need new or increased home services especially after a hospitalization. They may benefit from disease-specific home-based treatment/evaluation programs like home-based asthma programs to assess housing conditions triggering exacerbations, home-based heart failure management programs, etc. Patients may also benefit from home nursing assessments, home health aides, or care management through their health insurance. If patients are already enrolled in home-based services, then communication with those services can help in transitioning from inpatient to outpatient care and ensuring medication treatment changes or recommendations are reinforced. | | |
| **Slide 54**  Discharge Checklist | | **Big Picture**: Walk through sequential steps to the structurally competent discharge checklist. |
| 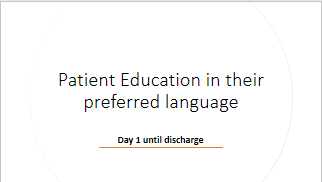 | |  |
| *Suggested script/talking points:*  Patient education in their preferred language starts on day 1. | | |
| **Slide 55**  Discharge Checklist | | **Big Picture**: Walk through sequential steps to the structurally competent discharge checklist. |
| 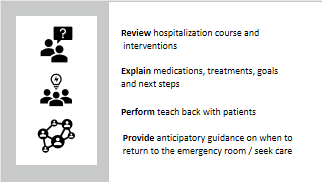 | |  |
| *Suggested script/talking points:*  Making sure patients are being communicated with in their preferred language, with the use of interpreters, is vital. Some patients may prefer family members be present when education is given to help reinforce changes/provide additional support in treatment management. Patient’s level of education should be taken into account to assess health literacy. It’s important that written patient education materials be provided at an appropriate reading level in the language preferred by the patient. Additionally, interventions related to social needs should be actively discussed with patients and all questions answered. | | |
| **Slide 56**  Discharge Checklist | | **Big Picture**: Walk through sequential steps to the structurally competent discharge checklist. |
| 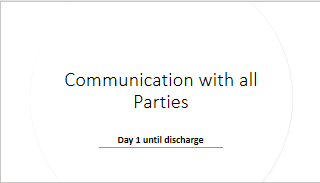 | |  |
| *Suggested script/talking points:* Read Slide | | |
| **Slide 57**  Discharge Checklist Time Check 88:00 Minutes | | **Big Picture**: Walk through sequential steps to the structurally competent discharge checklist. |
| 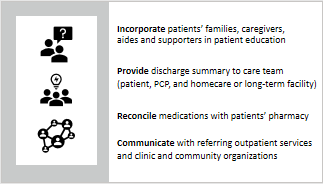 | |  |
| *Suggested script/talking points:*  It’s important to inform patients about who you/the team is communicating with, what they are communicating and why, and to get input from patients regarding who they want involved in their care. Solutions to social needs often involve interdisciplinary teams and communication with them and with patients is important. Interdisciplinary team rounds is a good venue to make sure everyone is on the same page. Additionally, if referrals are being made to community service organizations outside the hospital, communication with those organizations can help make it easier to ensure patients will receive follow-up and connect to services. It’s helpful to include social needs interventions in discharge summaries to make outpatient providers aware of the needs and interventions planned and allow them to follow-up with patients.  . | | |
| **Slide 58**  Resources | | **Big Picture**: Highlight resources for continued independent learning |
| 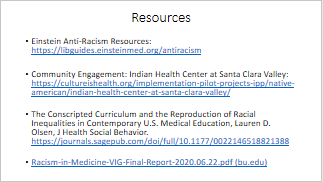 | |  |
| *Suggested script/talking points:*  Listed are some resources that can help you incorporate structural competency while on the wards. | | |
