# Supplementary material for: Structural Competency: A Faculty Development Workshop Series for Anti-racism in Medical Education
Source: MedEdPORTAL. 2025 Feb 7;21:11492. doi: 10.15766/mep_2374-8265.11492 (PMC11802914; doi:10.15766/mep_2374-8265.11492)
Supplement: Supplementary file 1 — 1 - Introduction to SC.pptx1 - Facilitator Guide.docx1 - SC Rubric Handout.docx1 - Sample SC Learning Goals.docx2 - Resident Reports & Case-Based Presentations.pptx2 - Facilitator Guide.docx2 - Structural Differential Handout.docx2 - Small-Group Handout.docx3 - Demystifying SC.pptx3 - Facilitator Guide.docx3 - SC One-Minute Preceptor Handout.docx3 - SC SNAPPS Handout.docx3 - Role-Play Scenarios.docx4 - SC Hospital-Based Teaching.pptx4 - Facilitator Guide.docx4 - Daily Inpatient Checklist.docx4 - SC Discharge Checklist.docx4 - Small-Group Scenarios.docxPre- and Postsurveys.docx [file mep_2374-8265.11492-s001.zip › G. 2 - Structural Differential Handout.docx]

Structural Differential

Create a prioritized **clinical problem list**

Identify structural **root causes** for **clinical** problems

Generate a prioritized **structural problem list**

Develop solutions to **address structural problems**

# **Create** a prioritized clinical problem list

Ensure congruity between patient and clinician problem lists.

**Identify** structural root causes for clinical problems

Elicit upstream structural and social determinants of health that contribute to clinical problems.

Integrate historical context.

# **Generate** a prioritized structural problem list

Incorporate patient priorities, preferences, and concerns.

Prioritize urgent problems and problems for which clinical and community resources are available.

# **Develop** solutions to address structural problems

Imagine individual-level, health system-level, community level and population-level solutions.

Consider individual and community strengths/assets.

Partner with community-led efforts.

# **Create** a prioritized clinical problem list

*Clinical Problem List:*

*(1) Uncontrolled asthma with frequent asthma attacks*

**Identify** structural root causes for clinical problems

Using a structural vulnerability assessment, you discover that your patient has poor housing conditions and numerous triggers in their home such as leaks, mold and pests. Your patient’s building has been cited in an increasing number of housing violations as the neighborhood is starting to gentrify.

# **Generate** a prioritized structural problem list

**Develop** solutions to address structural problems

Structural Problem List:

1. *Poor housing conditions stemming from landlord neglect, exacerbated by gentrification*
2. *Uncontrolled asthma with frequent asthma attacks*

In addition to medications, offer resources to report housing violations to local agencies, links to a community health worker home-based asthma program, and information for the clinic’s medical-legal partnership.

Support community organizer and tenet-led efforts to form a tenet’s association and join larger community-led efforts mitigating gentrification.
