# Supplementary material for: Structural Competency: A Faculty Development Workshop Series for Anti-racism in Medical Education
Source: MedEdPORTAL. 2025 Feb 7;21:11492. doi: 10.15766/mep_2374-8265.11492 (PMC11802914; doi:10.15766/mep_2374-8265.11492)
Supplement: Supplementary file 1 — 1 - Introduction to SC.pptx1 - Facilitator Guide.docx1 - SC Rubric Handout.docx1 - Sample SC Learning Goals.docx2 - Resident Reports & Case-Based Presentations.pptx2 - Facilitator Guide.docx2 - Structural Differential Handout.docx2 - Small-Group Handout.docx3 - Demystifying SC.pptx3 - Facilitator Guide.docx3 - SC One-Minute Preceptor Handout.docx3 - SC SNAPPS Handout.docx3 - Role-Play Scenarios.docx4 - SC Hospital-Based Teaching.pptx4 - Facilitator Guide.docx4 - Daily Inpatient Checklist.docx4 - SC Discharge Checklist.docx4 - Small-Group Scenarios.docxPre- and Postsurveys.docx [file mep_2374-8265.11492-s001.zip › M. 3 - Role-Play Scenarios.docx]

**In small groups, one faculty member will role-play the preceptor, one will role-play the learner, and the remaining will be observers. Below are worksheets for each role.**

**Preceptor Version**

*Instructions:* Take a minute to read through the case stem below. When you reach the end of the case, utilize prompts from the one-minute preceptor and steps of the structural differential to prompt your learner to develop a structurally competent assessment and plan for this patient. Refer to pages 2-4 for a reminder of the precepting tools presented.

*Case stem:*

Mr. K who is 53 years old and has a past medical history of diet-controlled diabetes type II, hypertension, chronic kidney disease stage III, bilateral knee osteoarthritis, obesity and asthma who is presenting for a follow-up visit. This is his first visit in nine months due to the COVID-19 pandemic. He had COVID-19 two months ago but has since recovered and denies shortness of breath, cough, fever, chest pain or other symptoms at this time. He reports he has been unable to take his blood pressure medications for the past month and half.

His blood pressure today is 172/85, his BMI is 37, and he is afebrile. His lung and heart exam are normal. His point-of-care A1c is 8.1.

**Learner Version**

*Instructions:* Take a minute to read through the case stem below to familiarize yourself with the patient you are presenting. Your preceptor will utilize prompts from the one-minute preceptor and steps of the structural differential to help you develop a structurally competent assessment and plan for this patient. If prompted by your preceptor, provide additional social history as listed below.

*Case stem:*

Mr. K who is 53 years old and has a past medical history of diet-controlled diabetes type II, hypertension, chronic kidney disease stage III, bilateral knee osteoarthritis, obesity and asthma who is presenting for a follow-up visit. This is his first visit in nine months due to the COVID-19 pandemic. He had COVID-19 two months ago but has since recovered and denies shortness of breath, cough, fever, chest pain or other symptoms at this time. He reports he has been unable to take his blood pressure medications for the past month and half.

His blood pressure today is 172/85, his BMI is 37, and he is afebrile. His lung and heart exam are normal. His point-of-care A1c is 8.1.

*If the preceptor asks for or elicits additional social history:*

Mr. K’s household contracted COIVD-19 and his mother passed away from COVID-19 shortly after. He has not been able to find work since he got sick. Mr. K is grieving over the loss of his mom, who was the main source of support for him. It seems almost everyone in his building got sick from COVID-19. He expressed really wanting to take care of himself and his health now that his mom is gone. However, he’s been told his health insurance is not active, and now he is worried about paying for his medications.

**Observer Version**

*Instructions:* Take a minute to read through the case stem below. When you reach the end of the case, utilize the table which lists out the microskills of the one-minute preceptor and steps of the structural differential to identify if the preceptor appropriately guides the learner to develop a structurally competent assessment and plan for this patient.  Be prepared to give feedback to the preceptor

*Case Stem for reference: the preceptor only has access to the first two paragraphs*

Mr. K who is 53 years old and has a past medical history of diet-controlled diabetes type II, hypertension, chronic kidney disease stage III, bilateral knee osteoarthritis, obesity and asthma who is presenting for a follow-up visit. This is his first visit in nine months due to the COVID-19 pandemic. He had COVID-19 two months ago but has since recovered. He reports he has been unable to take his blood pressure medications for the past month and half.

His blood pressure today is 172/85, his BMI is 37, and he is afebrile. His lung and heart exam are normal. His point-of-care A1c is 8.1.

*The learner will give additional information if the preceptor asks for or elicits additional social history:*

Mr. K’s household contracted COVID-19 and his mother passed away from COVID-19 shortly after. He has not been able to find work since he got sick. Mr. K is grieving over the loss of his mom, who was the main source of support for him. It seems almost everyone in his building got sick from COVID-19. He expressed really wanting to take care of himself and his health now that his mom is gone. However, he’s been told his health insurance is not active, and now he is worried about paying for his medications.

| ***The preceptor may not use every skill; the goal is to reinforce which skills they are already using well, and to highlight areas for growth if there are any.* | | | |
| --- | --- | --- | --- |
| **Microskills** | **Example Probes** | **Check if done** | **Notes on wording used by preceptor** |
| **Get a commitment to a structural problem related to the case** | “What structural contributors are affecting this patient’s health?”  “Why might this patient be disproportionately experiencing health inequity? How does that change your problem list?” |  |  |
| **Probe for supporting evidence & address implicit bias** | “Did you ask the patient about barriers to taking his medications?”  “What are the patient’s perceptions on their current medical condition?” |  |  |
| **Reinforce what was done well** | “That was a comprehensive social history, and I am glad you used the social determinants of health screening tool!”  “Nice job on identifying the insurance challenges Mr. K is facing!” |  |  |
| **Give guidance about errors/omissions** | “I see you mentioned that he has not been seen in 9 months, other than pandemic restrictions, were there any other contributing factors?  “During your presentation, the social history was limited and only reported symptoms. How can you expand/ strengthen this part of the presentation?” |  |  |
| **Teach core structural differential steps** | **Create** a prioritized clinical problem list including patient concerns/symptoms  **Identify** structural root causes for clinical problems such as SDoH  **Generate** a prioritized structural problem list that incorporates patient priorities, preferences, and concerns and prioritize problems for which clinical and community resources are available.  **Develop** solutions to address structural problems such as interdisciplinary team approaches and engaging community organizations |  |  |

Chart compiled by Dr. Eloho Ufomata MD MS, University of Pittsburgh
