# Supplementary material for: Structural Competency: A Faculty Development Workshop Series for Anti-racism in Medical Education
Source: MedEdPORTAL. 2025 Feb 7;21:11492. doi: 10.15766/mep_2374-8265.11492 (PMC11802914; doi:10.15766/mep_2374-8265.11492)
Supplement: Supplementary file 1 — 1 - Introduction to SC.pptx1 - Facilitator Guide.docx1 - SC Rubric Handout.docx1 - Sample SC Learning Goals.docx2 - Resident Reports & Case-Based Presentations.pptx2 - Facilitator Guide.docx2 - Structural Differential Handout.docx2 - Small-Group Handout.docx3 - Demystifying SC.pptx3 - Facilitator Guide.docx3 - SC One-Minute Preceptor Handout.docx3 - SC SNAPPS Handout.docx3 - Role-Play Scenarios.docx4 - SC Hospital-Based Teaching.pptx4 - Facilitator Guide.docx4 - Daily Inpatient Checklist.docx4 - SC Discharge Checklist.docx4 - Small-Group Scenarios.docxPre- and Postsurveys.docx [file mep_2374-8265.11492-s001.zip › S. Pre- and Postsurveys.docx]

Faculty Development in Structural Competency Survey

Definitions:

Structural competency: “A shift in medical education toward attention to forces that influence health outcomes at levels above individual interactions” and the capacity for health professionals to recognize and respond to health and illness as the downstream effects of broad social, political, and economic structures (Metzl and Hansen).

Attitudes Questions: Rate how much you agree or disagree with the following statements:

|  | Strongly Disagree (1) | Somewhat Disagree (2) | Somewhat Agree (3) | Strongly Agree (4) |
| --- | --- | --- | --- | --- |
| Race is a risk factor for disease (1 - RC) |  |  |  |  |
| Providers should be critical of clinical prediction tools that utilize race as a risk factor (2) |  |  |  |  |
| Structural and social determinants of health account for the majority of health outcomes (3) |  |  |  |  |
| Providers should discuss social needs with their patients (4) |  |  |  |  |
| Providers should help patients address social needs as part of the clinical encounter (5) |  |  |  |  |

**[Postintervention Only]** Questions: Please select the faculty development sessions you attended (select all that apply):

- Session I: Introduction to Structural Competency & Revising Existing Curricula (1)
- Session II: Transforming Resident Report and Case Based Presentations Using the Structural Differential (2)
- Session III: Ambulatory Teaching: The Structurally Competent Preceptor (3)
- Session IV: Inpatient Teaching: The Structurally Competent Hospital-Based Care (4)

**[Postintervention Only]** Confidence Questions: **Prior to this curriculum**, how **confident** did you feel incorporating the following into your teaching (includes lectures, case-based sessions, precepting, rounding, etc.)?

|  | Not confident at all (1) | Slightly confident (2) | Somewhat confident (3) | Fairly confident (4) | Completely confident (5) |
| --- | --- | --- | --- | --- | --- |
| Discussing racial/ethnic health disparities |  |  |  |  |  |
| Discussing structural/social determinants of health that contribute to health disparities |  |  |  |  |  |
| Discussing structural/social determinants of health when discussing health behaviors |  |  |  |  |  |
| Offering solutions for social needs |  |  |  |  |  |

**[Postintervention Only]** Confidence Questions: After this faculty development curriculum, how confident do you feel incorporating the following into your teaching (includes lectures, case-based sessions, precepting, rounding, etc.)?

|  | Not confident at all (1) | Slightly confident (2) | Somewhat confident (3) | Fairly confident (4) | Completely confident (5) |
| --- | --- | --- | --- | --- | --- |
| Discussing racial/ethnic health disparities |  |  |  |  |  |
| Discussing structural/social determinants of health that contribute to health disparities |  |  |  |  |  |
| Discussing structural/social determinants of health when discussing health behaviors |  |  |  |  |  |
| Offering solutions for social needs |  |  |  |  |  |

**[Postintervention Only]** Please take a moment to describe the impact this faculty development series has had on your clinical care or practice:

________________________________________________________________

________________________________________________________________

________________________________________________________________

________________________________________________________________

________________________________________________________________

Demographics Questions: How many years have you been a practicing provider?

- Less than 5 years (1)
- 5-10 years (2)
- 11-15 years (3)
- 16-20 years (4)
- 21-25 years (5)
- > 25 years (6)

What is your medical specialty?

________________________________________________________________

Where did you receive your medical school training?

- Within the United States (1)
- Outside the United States (2)
- Prefer not to answer (3)

List any additional degrees or special training

________________________________________________________________

________________________________________________________________

________________________________________________________________

________________________________________________________________

________________________________________________________________

How do you self-identify with regards to gender? [select all that apply]

- Cisgender Female (1)
- Cisgender Male (2)
- Transgender Female (3)
- Transgender Male (4)
- Non-binary/Third Gender (5)
- Prefer not to answer (6)

How do you self-identify with regards to sexual orientation? [select all that apply]

- Gay/Lesbian (1)
- Straight (2)
- Bisexual (3)
- Pansexual/Fluid (4)
- Asexual (5)
- Other (6) ________________________________________________
- Prefer not to answer (7)

How do you self-identify with regards to racial/ethnic identity [select all that apply]

- White (Eg: German, Irish, English, Italian, Polish, French, etc) (1)
- Hispanic, Latino or Spanish origin (Eg: Mexican or Mexican American, Puerto Rican, Cuban, Salvadoran, Dominican, Colombian, etc) (2)
- Black or African American (Eg: African American, Jamaican, Haitian, Nigerian, Ethiopian, Somalian, etc) (3)
- Asian (Eg: Chinese, Filipino, Asian Indian, Vietnamese, Korean, Japanese, etc) (4)
- American Indian or Alaska Native(Eg: Navajo nation, Blackfeet tribe, Mayan, Aztec, Native Village or Barrow Inupiat Traditional Government, Nome Eskimo Community, etc) (5)
- Middle Eastern or North African (Eg: Lebanese, Iranian, Egyptian, Syrian, Moroccan, Algerian, etc) (6)
- Native Hawaiian or Other Pacific Islander (Eg: Native Hawaiian, Samoan, Chamorro, Tongan, Fijian, etc) (7)
- Some other race, ethnicity or origin (8)
- Prefer not to answer (9)
