# Supplementary material for: Structural Competency: A Faculty Development Workshop Series for Anti-racism in Medical Education
Source: MedEdPORTAL. 2025 Feb 7;21:11492. doi: 10.15766/mep_2374-8265.11492 (PMC11802914; doi:10.15766/mep_2374-8265.11492)
Supplement: Supplementary file 1 — 1 - Introduction to SC.pptx1 - Facilitator Guide.docx1 - SC Rubric Handout.docx1 - Sample SC Learning Goals.docx2 - Resident Reports & Case-Based Presentations.pptx2 - Facilitator Guide.docx2 - Structural Differential Handout.docx2 - Small-Group Handout.docx3 - Demystifying SC.pptx3 - Facilitator Guide.docx3 - SC One-Minute Preceptor Handout.docx3 - SC SNAPPS Handout.docx3 - Role-Play Scenarios.docx4 - SC Hospital-Based Teaching.pptx4 - Facilitator Guide.docx4 - Daily Inpatient Checklist.docx4 - SC Discharge Checklist.docx4 - Small-Group Scenarios.docxPre- and Postsurveys.docx [file mep_2374-8265.11492-s001.zip › _Educational Summary Report_11492.pdf]

MedEdPORTAL® | The AAMC Journal of Teaching and Learning Resources

Original Publication

Open Access

Structural Competency: A Faculty Development Workshop Series for Anti-racism in Medical Education

Shani R. Scott, MD*, Cristina M. Gonzalez, MD, MEd, Chenshu Zhang, PhD, Iman Hassan, MD, MS

*Corresponding author: shanscot@montefiore.org

Abstract

Introduction: In response to accreditation bodies requiring health disparities curricula, medical educators are tasked with incorporating structural competency, the understanding of how social and structural barriers like structural racism impact health, into their teaching. Most have not received training in this area, yet there remains a scarcity of faculty development curricula to address this gap. We describe the creation, implementation, and evaluation of a faculty development workshop series rooted in the framework of structural competency. **Methods:** We delivered four 90-minute workshops at an urban academic medical center from March through April of 2021. Workshops were offered to interdisciplinary faculty. We evaluated this workshop series with a pre- and postintervention survey assessing attitudes and confidence, and a postintervention satisfaction survey. Data analysis was conducted using a paired *t* test. **Results:** A total of 206 participants attended at least one workshop within the series, and 20 participants completed both pre- and postintervention surveys. Participants overwhelmingly recommended these workshops to their colleagues and had significant increases in overall attitudes (3.3 vs. 3.6, $p = .001$) and level of confidence (3.2 vs. 3.9, $p < .001$) incorporating structural competency. **Discussion:** Our application of structural competency to faculty workshops and teaching tools feasibly engages faculty in instruction to incorporate concepts of structural racism and the downstream effects of social determinants of health into clinical teaching. It represents an innovative tool as we seek to enhance clinical teaching to improve care for racially and ethnically minoritized communities.

Keywords
Faculty Development, Social and Structural Determinants of Health, Anti-racism, Case-Based Learning, Clinical Teaching/Bedside Teaching, Diversity, Equity, Inclusion

Educational Objectives

By the end of this activity, learners should be able to:

1. Explain structural competency and its principles.
2. Apply a structurally competent/anti-racism rubric to existing curricula.
3. Construct a structural differential for case-based presentations.
4. Integrate structural and social determinants of health into precepting encounters.
5. Apply structural competency to inpatient admissions and discharge planning.

Citation:
Scott SR, Gonzalez CM, Zhang C, Hassan I. Structural competency: a faculty development workshop series for anti-racism in medical education. *MedEdPORTAL*. 2025;21:11492.
https://doi.org/10.15766/mep_2374-8265.11492

Introduction

Structural racism is defined as the cumulative effects of policies, institutional practices, cultural representations, and other norms that work together to perpetuate racial inequities in access to social resources.[1] Teaching faculty who are unable to conceptualize structural racism in medicine risk indoctrinating learners with an ideology that pathologizes race and perpetuates racial health disparities, rather than acknowledging the impact of historic and present day structural inequities.[2-4] Accreditation bodies, in response to this widespread teaching practice, now require institutions to teach residents and medical students about structural and social determinants of health (SDOH) and their influence on health disparities.[5,6]

However, academic institutions compensate by tasking ethnically and racially minoritized faculty members to radically change crystalized institutional cultural attitudes, develop innovative education models, and implement program interventions which are typically underfunded. This approach saddles these

Copyright © 2025 Scott et al. This is an open-access publication distributed under the terms of the Creative Commons Attribution license.

faculty members with disproportionate levels of emotionally, mentally, and physically taxing teaching labor to bridge these gaps in education, potentially hindering their own professional development (referred to as a minority tax).[7]

In response to these problems, anti-racist faculty development frameworks have emerged that center on building an inclusive learning environment, broadening knowledge of anti-racism terminology, and strengthening capacity for clinical reasoning that reflects the impact of racism on access to health care resources.[8,9] Despite these advancements, there remains a gap in providing concrete teaching tools for educators to implement anti-racist strategies effectively. While many resources address general curricular frameworks, they often overlook the need for specific anti-racist approaches.[10-12] Existing publications may detail teaching methods and evaluation techniques but fail to offer teaching methods and supportive tools for deconstructing systemic racism within curricula. This gap is especially pronounced in addressing local needs where racism affects health disparities and educational equity. The lack of comprehensive anti-racist curricular guidelines highlights the need for targeted research and development to ensure medical education actively confronts racial inequities in health care systems and communities.

Structural competency can facilitate the active process of anti-racism through the identification and ultimate elimination of racism in systems, policies, and practices. Structural competency is the ability of providers to conceptualize social and structural barriers to health and incorporate awareness of these barriers into clinical care. Specifically, structural competency is comprised of five pillars that collectively improve the ability of health care providers to recognize and respond to health and illness as the downstream effects of social, political, and economic structures. These pillars are (1) recognizing structures shaping clinical encounters, (2) developing an extra-clinical language, (3) rearticulating cultural presentations in structural terms, (4) observing and imagining structural interventions, and (5) developing structural humility.[11] We utilized the framework of structural competency to develop and evaluate a faculty development workshop series with associated concrete teaching tools for use in the clinical learning environment.

Methods

Population and Sample

Our faculty development workshop series was held at a large, urban academic medical center in the Bronx, New York. We invited faculty from all disciplines to participate in sessions focused on structural competency. These sessions required no prior knowledge and were open to all clinician educators with no participant limits. Facilitated by authors Shani R. Scott and Iman Hassan via Zoom, the workshops took place between March and April 2021. Participants preregistered and were encouraged, but not required, to attend all four sessions.

The workshops were hosted by various departments: weekly sessions organized by the Albert Einstein College of Medicine's Office of Faculty Development, a grand rounds presentation by the Division of General Internal Medicine and the Department of Geriatric Medicine, and two half-day educational conferences presented to the Department of Psychiatry, which occurred over the course of 1 year.

The one-time or two-time standalone presentations, including the grand rounds and half-day conferences, were designed to reach a broader audience and provide targeted education on structural competency. These sessions, which were conducted at the enthusiastic request of institutional colleagues following the feedback from the initial workshops, were included in the evaluation data. This inclusion contributed to the overall participant numbers. However, the separate scheduling of these standalone sessions likely explains the lower number of participants who attended all four sessions, as these standalone presentations attracted different groups from those attending the weekly series.

Intervention

The faculty development series comprised four 90-minute workshops aimed at training faculty to integrate structural competency into curricula, case presentations, ambulatory precepting, and inpatient care. Each workshop was informed by a literature review and existing *MedEdPORTAL* curricula, with detailed resources provided in the appendices to assist faculty with varying levels of familiarity with structural competency.[9-14]

The first workshop, titled Introduction to Structural Competency and Revising Curricula, introduced structural competency principles, and the historical context of racism in academic medicine including anti-racist terminology (Appendices A & B). Participants used a structurally competent anti-racist rubric (Appendix C), developed from Krishnan et al.'s research, to revise their teaching materials.[15] They practiced applying the rubric to sample content and their own lectures, using Qualtrics XM (v. 2021) platform for tailored feedback. As part of the rubric, participants were encouraged to incorporate at least one structurally competent learning goal into their existing teaching sessions, regardless of topic, based on sample goals

Copyright © 2025 Scott et al. This is an open-access publication distributed under the terms of the Creative Commons Attribution license.

(Appendix D). Participants conducted a small-group exercise in which they practiced using the rubric to review a sample PowerPoint didactic and discussed areas for improvement based on the results.

The second workshop in the series, Transforming Resident Report and Case Based Presentations, introduced the structural differential, a four-step guide for creating structurally competent care plans (Appendices E, F, & G).[16] Participants learned to incorporate historical context into clinical teaching using COVID-19 as an example and practiced developing a structural differential with a fishbone diagram in small-group breakouts (Appendix H).

In the third workshop of the series, Demystifying Structural Competency: Promoting Anti-racist Training in Ambulatory Medical Education, we discussed institutional racism in ambulatory care, using segregated health clinics as a case study (Appendices I & J). We introduced our structural competency adaptations of the One Minute Preceptor (Appendix K) and SNAPPS models (Appendix L), and participants practiced these precepting tools in role-play scenarios (Appendix M).[14,17,18]

The fourth and final session of the series was Structurally Competent Hospital-Based Teaching. This session focused on creating a structurally competent learning environment in inpatient settings (Appendices N & O) using checklists (Appendices P & Q).[19,20] Participants worked in small groups to review and revise a sample progress note (Appendix R) to eliminate documentation bias using the structural competency checklists.

Data Collection and Analysis

Our preliminary evaluation of this intervention assessed participants' reaction and learning. An amalgamation of participants responses derived from the workshop venues completed a two-question satisfaction survey, during which they rated on a 4-point Likert scale (1 = *disagree*, 4 = *strongly agree*) whether the sessions would result in practice changes, with an option for *not applicable* responses. They were also asked to give feedback on whether they would recommend the workshops to a colleague using a 5-point Likert scale (1 = *strongly disagree*, 5 = *strongly agree*).

Using a pre- and postsurvey (Appendix S), we assessed attitude changes and collected participant demographic information. Attitudes about race and structural and social determinants of health were assessed using a 4-point Likert scale (1 = *strongly disagree*, 4 = *strongly agree*). The postintervention survey include some specific questions on session attendance and impact, and retrospectively assessed pre/postintervention changes in self-reported confidence on a 5-point Likert scale (1 = *not confident at all*, 5 = *completely confident*). Surveys were matched via anonymous coding, using each participant's unique login based upon their initials; birth day and month. Overall confidence was determined based upon the mean of confidence survey questions and assessed using paired *t* tests.

This study was deemed exempt by the Albert Einstein College of Medicine Institutional Review Board (Reference # 2020-12238; February 18, 2021).

Descriptive statistics using means and frequencies were computed to analyze demographic data. Pre- and postintervention survey questions and retrospective pre- and postsurvey questions were assessed using paired *t* tests. Data analysis was conducted using SAS v9.4 (SAS Institute).

Results

Two hundred and six participants attended at least one workshop. Of those, 18 (9%) attended all four workshops, 36 (17%) attended three workshops, 67 (33%) attended two workshops, and 85 (41%) attended one workshop.

Eighty-one participants attended any of the four the workshops through our Office of Faculty Development. Twenty-three (35%) of 64 workshop 1 participants, 15 (30%) of 50 workshop 2 participants, 14 (39%) of 36 workshop 3 participants, and nine (39%) of 23 workshop 4 participants completed the two-question satisfaction survey. Results from all four workshops showed that 100% of participants selected *agree* or *strongly agree* when asked if attending the activity increased their confidence in their medical knowledge, procedural or cognitive skills, patient clinical outcomes, and practice behavior. When asked if they would recommend the workshop, 91%, 93%, 100%, and 78% strongly agreed for the first, second, third, and fourth workshops, respectively. Notably, no participants disagreed.

Of the 206 participants across all workshop venues, 94 completed the preintervention survey (response rate of 46%), 33 participants completed the postintervention survey, and 20 matched participants completed both the pre- and postintervention surveys, resulting in a 10% response rate overall. Among those who completed both surveys, the majority attended only one session (*n* = 12), professionally trained with the US (*n* = 17), worked in the Department of Medicine (*n* = 12), and had been in practice 5 to 10 years (*n* = 8).

Copyright © 2025 Scott et al. This is an open-access publication distributed under the terms of the Creative Commons Attribution license.

Also notably, three participants attended all four sessions (Table 1).

Analysis of matched surveys ($N = 20$) revealed significant improvements in knowledge/attitudes, and confidence. Improvement in overall attitudes (3.3 to 3.6, $p = .001$) was primarily driven by participants disagreeing (item was reverse-coded) more with the statement "Race is a risk factor for disease" and agreeing more with the statement "Providers should be critical of clinical prediction tools that utilize race as a risk factor" from pre- to postintervention (Table 2). When assessed retrospectively, participants reported a significant increase in overall confidence after the training (3.2 to 3.9, $p < .001$) and a significant increase in confidence in all items assessed (Table 3).

Table 1. Demographics of Participants Who Completed the Preintervention Survey ($N = 94$) and Both Pre- and Postintervention Surveys ($N = 20$)

Item	Preintervention No. (%)	Pre/Post Matched No. (%)
Specialty		
Medicine/medicine subspecialty	43 (46)	12 (60)
Psychiatry	22 (23)	5 (25)
Neurology	8 (9)	0 (0)
Pediatrics	7 (7)	2 (10)
Obstetrics/gynecology	3 (3)	0 (0)
Pathology	2 (2)	0 (0)
Family medicine	2 (2)	0 (0)
Other (radiology, vascular surgery, researcher)	7 (7)	1 (5)
Number of sessions attended		
1		12 (60)
2		3 (15)
3		2 (10)
4		3 (15)
Number of years in practice		
<5	16 (17)	5 (25)
5-10	29 (31)	8 (40)
11-15	16 (17)	3 (15)
16-20	7 (7)	1 (5)
21-25	5 (5)	2 (10)
>25	21 (22)	1 (5)
Location of medical school training		
Within the US	78 (83)	17 (85)
Outside the US	11 (12)	2 (10)
Prefer not to specify	5 (5)	1 (5)
Gender		
Cisgender female	61 (65)	16 (80)
Cisgender male	24 (26)	1 (5)
Nonbinary/third gender	1 (1)	1 (5)
Prefer not to specify	8 (9)	2 (10)
Sexual orientation		
Gay/lesbian	3 (3)	0 (0)
Straight	77 (82)	18 (90)
Bisexual	3 (3)	0 (0)
Other	1 (1)	0 (0)
Prefer not to specify	10 (11)	2 (10)
Race or Ethnicity		
Asian	18 (19)	4 (20)
Black/African American	7 (7)	0 (0)
Hispanic, Latino, or Spanish origin	6 (6)	1 (5)
Middle Eastern or North African	2 (2)	0 (0)
White	39 (41)	12 (60)
Multiracial	8 (9)	1 (5)
Some other race, ethnicity, or origin	5 (5)	0 (0)
Prefer not to specify	9 (10)	2 (10)

Discussion

Historically, faculty development is recognized by medical education organizations as an essential support provided to faculty members to assist them in responding to the challenges of evolving responsibilities.[21-23] Our faculty development workshop series and adapted teaching tools contribute to the literature by using structural competency as a guiding framework to incorporate structural influences on health disparities into teaching. Within 1 month, we successfully delivered four 90-minute workshops to an interdisciplinary audience. We engaged 206 faculty spanning multiple training levels, disciplines, and departments in small-group discussions to critically think and reflect on structural barriers to care with an emphasis on structural racism. Furthermore, our assessment captures improvements in attitudes and confidence regarding structural competency, specifically increased confidence among teaching faculty in reflecting on and modifying their teaching and clinical practices using structural competency.

Educating a broad range of faculty is necessary for institutional culture change and mitigates the unfair reliance on a limited number of volunteer faculty—frequently people of color—to teach on these topics.[24] Expanding the skillset of the greater faculty body can help mitigate isolation, and reduce the minority tax for faculty, potentially enhancing diversity efforts.[25] Results of our intervention are consistent with a prior, well-received effort to instruct a group medical students, residents, and faculty in the concept of structural competency.[11] Our approach expands the existing literature by moving beyond structural competency as a concept to providing faculty with structurally competent adaptations of familiar tools, allowing for concrete implementation into their clinical teaching.

Facilitating a four-part series on anti-racist approaches to faculty development provided invaluable insights into the complexities and nuances of teaching such sensitive and critical content. One of the significant lessons learned from the four-part series on anti-racist approaches to faculty development is the critical need for faculty to have consistent exposure and practice with a unifying terminology and language when discussing structural racism in health care and medical education. This unifying language serves as a foundation for clear and effective communication, enabling faculty to engage in meaningful dialogue and reflection on these

Copyright © 2025 Scott et al. This is an open-access publication distributed under the terms of the Creative Commons Attribution license.

Table 2. Reported Knowledge and Attitudes for Matched Pre- and Postintervention Participants ($N = 20$)

Knowledge/Attitude Item[a]	Preintervention *M (SD)*	Postintervention *M (SD)*	*p*
Race is a risk factor for disease (reverse coded)	2.3 (1.1)	3.2 (1.0)	<.001
Providers should be critical of clinical prediction tools that utilize race as a risk factor	3.5 (0.6)	3.8 (0.4)	.003
Structural and social determinants of health account for the majority of health outcomes	3.4 (0.8)	3.6 (0.5)	.16
Providers should discuss social needs with their patients	3.8 (0.7)	3.9 (0.3)	.42
Providers should address social needs as part of the clinical encounter	3.6 (0.8)	3.8 (0.4)	.42
Overall attitudes	3.3 (0.5)	3.6 (0.3)	.001

[a]Rated based on a 4-point Likert-like scale (1 = *strongly disagree*, 4 = *strongly agree*).

complex issues. Without a shared vocabulary, discussions can become fragmented, and misunderstandings may arise, hindering progress toward addressing racial inequities. By providing faculty with the tools and language to articulate the nuances of structural racism, we empower them to confidently navigate conversations, challenge existing biases, and implement anti-racist practices in their teaching and clinical environments.

Additionally, the importance of creating an emotionally and intellectually safe and open environment where faculty members felt comfortable discussing race, bias, and systemic inequities was vital to sustaining discourse on the workshop subject matter. Engagement with faculty in postworkshop Q & A sessions repeatedly revealed that participants had feelings of shame or avoidance, or inexperience and insecurity in speaking about race and racism. We found that structured small-group discussions were effective in fostering deeper engagement and reflection, allowing participants to share personal experiences and explore the structural factors contributing to health disparities. The series also underscored the value of interdisciplinary collaboration, as it enriched discussions and broadened perspectives. Overall, the experience reinforced the importance of continuous learning and adaptability in facilitating such workshops, as well as the need for ongoing support and resources to sustain the impact of anti-racist training initiatives. Our assessment demonstrates that workshops are a feasible vehicle to equitably distribute knowledge, tools, and teaching skills for concepts such as race and structural racism. Incorporation of facilitated discussions enabled faculty to engage in safe discourse around these concepts. This practice of reflection and discourse is supported by the literature and aides in what Sotto-Santiago et al. refer to as the (re)learning of racism as a structural determinant of health.[12]

Table 3. Retrospective Self-Reported Confidence for Matched Pre- and Postintervention Participants ($N = 33$)

Confidence Item[a]	Retrospective Preintervention *M (SD)*	Postintervention *M (SD)*	*p*
Discussing racial/ethnic health disparities	3.3 (0.8)	3.8 (0.7)	<.001
Discussing structural/social determinants of health that contribute to health disparities	3.2 (0.9)	3.9 (0.6)	<.001
Discussing structural/social determinants of health when discussing health behaviors	3.4 (1.0)	4.0 (0.7)	<.001
Offering solutions for social needs	3.0 (1.2)	3.7 (0.9)	<.001
Overall confidence	3.2 (0.8)	3.9 (0.6)	<.001

[a]Rated based on a 5-point Likert scale (1 = *not confident at all*, 5 = *completely confident*).

Our study had several limitations, including a small number of matched survey responses, lack of a comparison group, and absence of a validated assessment tool. Most matched participants attended only one workshop, limiting our ability to assess the impact of attending all four sessions. This may reflect participants tailoring attendance to their clinical and teaching duties, contributing to overall numbers but resulting in fewer who attended all sessions. The study was conducted in an urban academic center, so perspectives from rural and suburban locations remain unexplored. Additionally, the virtual format introduced challenges like connectivity issues and limited facilitation in breakout rooms, though it likely allowed us to reach a larger number of faculty participants. Future research should address attrition by identifying and mitigating factors like scheduling conflicts and explore diverse medical centers to improve generalizability. To enhance the generalizability of this research, future studies should focus on addressing the high attrition rates within the workshop series by identifying and mitigating factors that contribute to participant drop-off, such as scheduling conflicts or workshop format. Additionally, expanding the intervention to include a more diverse range of medical centers and a larger, more varied sample of participants would improve the broader applicability of the findings across different health care environments.

Our faculty development workshop series aims to instill the attitudes and confidence needed for teaching with an awareness of racism in medical education. It goes further by equipping faculty with concrete anti-racist teaching tools adaptable to

Copyright © 2025 Scott et al. This is an open-access publication distributed under the terms of the Creative Commons Attribution license.

various clinical and didactic environments. The modular design allows sessions to be customized to fit the specific needs, demographics, and cultural contexts of different institutions. Institutions can choose relevant case studies and examples, and deliver the series in in-person, virtual, or hybrid formats, accommodating different logistical and technological capabilities. The workshops can be adjusted in complexity to suit various levels of familiarity with structural racism and health disparities and can be tailored in pacing and duration to fit scheduling needs and integrate with existing professional development frameworks. This flexibility would enhance relevance and impact across diverse educational settings, from large academic centers to smaller community programs. Future studies should focus on assessing educational behaviors, analyzing responses to individual workshop sessions, and evaluating the sustainability of behavioral changes.

Appendices

A. 1 - Introduction to SC.pptx
B. 1 - Facilitator Guide.docx
C. 1 - SC Rubric Handout.docx
D. 1 - Sample SC Learning Goals.docx
E. 2 - Resident Reports & Case-Based Presentations.pptx
F. 2 - Facilitator Guide.docx
G. 2 - Structural Differential Handout.docx
H. 2 - Small-Group Handout.docx
I. 3 - Demystifying SC.pptx
J. 3 - Facilitator Guide.docx
K. 3 - SC One-Minute Preceptor Handout.docx
L. 3 - SC SNAPPS Handout.docx
M. 3 - Role-Play Scenarios.docx
N. 4 - SC Hospital-Based Teaching.pptx
O. 4 - Facilitator Guide.docx
P. 4 - Daily Inpatient Checklist.docx
Q. 4 - SC Discharge Checklist.docx
R. 4 - Small-Group Scenarios.docx
S. Pre- and Postsurveys.docx

All appendices are peer reviewed as integral parts of the Original Publication.

Shani R. Scott, MD: Assistant Professor, Department of Medicine, Albert Einstein College of Medicine; Associate Program Director, Moses-Weiler Internal Medicine Residency Program, Montefiore Medical Center; Director of Diversity Affairs, Department of Medicine, Montefiore Medical Center; ORCID: https://orcid.org/0000-0001-5880-4456

Cristina M. Gonzalez, MD, MEd: Professor, Departments of Medicine and Population Health, New York University Grossman School of Medicine; Associate Director for the Institute for Excellence in Health Equity, New York University Grossman School of Medicine

Chenshu Zhang, PhD: Research Associate Professor, Department of Medicine, Albert Einstein College of Medicine

Iman Hassan, MD, MS: Associate Professor, Department of Medicine, Albert Einstein College of Medicine; Director for Community and Population Health Initiatives, Moses-Weiler Internal Medicine Residency Program, Montefiore Medical Center

Disclosures
None to report.

Funding/Support
None to report.

Ethical Approval
The Albert Einstein College of Medicine Institutional Review Board deemed further review of this project not necessary.

References

1. 11 terms you should know to better understand structural racism. Aspen Institute. July 11, 2016. Accessed January 23, 2025. https://www.aspeninstitute.org/blog-posts/structural-racism-definition/
2. Hardeman RR, Murphy KA, Karbeah J, Kozhimannil KB. Naming institutionalized racism in the public health literature: a systematic literature review. *Public Health Rep*. 2018;133(3):240-249. https://doi.org/10.1177/0033354918760574
3. Castle B, Wendel M, Kerr J, Brooms D, Rollins A. Public health's approach to systemic racism: a systematic literature review. *J Racial Ethn Health Disparities*. 2019;6(1):27-36. https://doi.org/10.1007/s40615-018-0494-x
4. Printz C. Racism and racial inequities in health care rise to the forefront: amid the country's national reckoning on race, experts are renewing their focus on systemic racism in the health care system. *Cancer*. 2020;126(18):4081-4082. https://doi.org/10.1002/cncr.33157
5. *Functions and Structure of a Medical School: Standards for Accreditation of Medical Education Programs Leading to the MD Degree*. Liaison Committee on Medical Education; 2023. Accessed January 23, 2025. https://www.google.com/url?sa=t&source=web&rct=j&opi=89978449&url=https://lcme.org/wp-content/uploads/2023/10/2023-24_Functions-and-Structure_2023-10-23.docx&ved=2ahUKEwjH0-qPnquKAxWUmYkEHTchN8QQFnoECBYQAQ&usg=AOvVaw3PrmXHKBsd8swSmqjbqEIZ

Copyright © 2025 Scott et al. This is an open-access publication distributed under the terms of the Creative Commons Attribution license.

6. Wagner R, Koh N, Bagian JP, Weiss KB; CLER Program. *National Report of Findings 2016: Issue Brief No. 4: Health Care Disparities*. Accreditation Council for Graduate Medical Education; 2017. Accessed January 23, 2025. https://www.acgme.org/globalassets/pdfs/cler/cler_health_care_disparities_issue_brief.pdf

7. Ziegelstein RC, Crews DC. The majority subsidy. *Ann Intern Med*. 2019;171(11):845-846. https://doi.org/10.7326/M19-1923

8. Cerdeña JP, Plaisime MV, Tsai J. From race-based to race-conscious medicine: how anti-racist uprisings call us to act. *Lancet*. 2020;396(10257):1125-1128. https://doi.org/10.1016/S0140-6736(20)32076-6

9. Jowell AR, James AK, Jasrasaria R, et al. DARE training: teaching educators how to revise internal medicine residency lectures by using an anti-racism framework. *MedEdPORTAL*. 2023;19:11351. https://doi.org/10.15766/mep_2374-8265.11351

10. Falusi O, Chun-Seeley L, de la Torre D, et al. Teaching the teachers: development and evaluation of a racial health equity curriculum for faculty. *MedEdPORTAL*. 2023;19:11305. https://doi.org/10.15766/mep_2374-8265.11305

11. Neff J, Holmes SM, Knight KR, et al. Structural competency: curriculum for medical students, residents, and interprofessional teams on the structural factors that produce health disparities. *MedEdPORTAL*. 2020;16:10888. https://doi.org/10.15766/mep_2374-8265.10888

12. Sotto-Santiago S, Poll-Hunter N, Trice T, et al. A framework for developing antiracist medical educators and practitioner-scholars. *Acad Med*. 2022;97(1):41-47. https://doi.org/10.1097/ACM.0000000000004385

13. Metzl JM, Hansen H. Structural competency: theorizing a new medical engagement with stigma and inequality. *Soc Sci Med*. 2014;103:126-133. https://doi.org/10.1016/j.socscimed.2013.06.032

14. Neff J, Knight KR, Satterwhite S, Nelson N, Matthews J, Holmes SM. Teaching structure: a qualitative evaluation of a structural competency training for resident physicians. *J Gen Intern Med*. 2017;32(4):430-433. https://doi.org/10.1007/s11606-016-3924-7

15. Krishnan A, Rabinowitz M, Ziminsky A, Scott SM, Chretien KC. Addressing race, culture, and structural inequality in medical education: a guide for revising teaching cases. *Acad Med*. 2019;94(4):550-555. https://doi.org/10.1097/ACM.0000000000002589

16. Hassan IF, Bui T. The structural analysis: incorporating structurally competent clinical reasoning into case-based presentations. *J Gen Intern Med*. 2022;37(13):3465-3470. https://doi.org/10.1007/s11606-022-07751-7

17. Neher JO, Gordon KC, Meyer B, Stevens N. A five-step “microskills” model of clinical teaching. *J Am Board Fam Pract*. 1992;5(4):419-424.

18. Wolpaw TM, Wolpaw DR, Papp KK. SNAPPS: a learner-centered model for outpatient education. *Acad Med*. 2003;78(9):893-898. https://doi.org/10.1097/00001888-200309000-00010

19. Soong C, Daub S, Lee J, et al. Development of a checklist of safe discharge practices for hospital patients. *J Hosp Med*. 2013;8(8):444-449. https://doi.org/10.1002/jhm.2032

20. Goddu AP, O’Conor KJ, Lanzkron S, et al. Do words matter? Stigmatizing language and the transmission of bias in the medical record. *J Gen Intern Med*. 2018;33(5):685-691. https://doi.org/10.1007/s11606-017-4289-2

21. Kirkpatrick DL. *Evaluating Training Programs: The Four Levels*. Berrett-Koehler; 1994.

22. Leslie K, Baker L, Egan-Lee E, Esdaile M, Reeves S. Advancing faculty development in medical education: a systematic review. *Acad Med*. 2013;88(7):1038-1045. https://doi.org/10.1097/ACM.0b013e318294fd29

23. Kumagai AK, White CB, Ross PT, Perlman RL, Fantone JC. The impact of facilitation of small-group discussions of psychosocial topics in medicine on faculty growth and development. *Acad Med*. 2008;83(10):976-981. https://doi.org/10.1097/ACM.0b013e3181850aeb

24. Wear D, Zarconi J, Aultman JM, Chyatte MR, Kumagai AK. Remembering Freddie Gray: medical education for social justice. *Acad Med*. 2017;92(3):312-317. https://doi.org/10.1097/ACM.0000000000001355

25. Campbell KM, Rodríguez JE. Addressing the minority tax: perspectives from two diversity leaders on building minority faculty success in academic medicine. *Acad Med*. 2019;94(12):1854-1857. https://doi.org/10.1097/ACM.0000000000002839

Received: April 8, 2024
Accepted: November 21, 2024
Published: February 7, 2025

Copyright © 2025 Scott et al. This is an open-access publication distributed under the terms of the Creative Commons Attribution license.
